# Suppressed Expression of T-Box Transcription Factors Is Involved in Senescence in Chronic Obstructive Pulmonary Disease

**DOI:** 10.1371/journal.pcbi.1002597

**Published:** 2012-07-19

**Authors:** George K. Acquaah-Mensah, Deepti Malhotra, Madhulika Vulimiri, Jason E. McDermott, Shyam Biswal

**Affiliations:** 1 Department of Pharmaceutical Sciences, Massachusetts College of Pharmacy and Health Sciences, Worcester, Massachusetts, United States of America; 2 Department of Environmental Health Sciences, Bloomberg School of Public Health, Johns Hopkins University, Baltimore, Maryland, United States of America; 3 Genetic Disease Research Branch, National Human Genome Research Institute, National Institutes of Health, Bethesda, Maryland, United States of America; 4 University of North Carolina at Chapel Hill, Raleigh-Durham, North Carolina, United States of America; 5 Computational Biology and Bioinformatics Group Pacific Northwest National Laboratory, Richland, Washington, United States of America; Ottawa University, Canada

## Abstract

Chronic obstructive pulmonary disease (COPD) is a major global health problem. The etiology of COPD has been associated with apoptosis, oxidative stress, and inflammation. However, understanding of the molecular interactions that modulate COPD pathogenesis remains only partly resolved. We conducted an exploratory study on COPD etiology to identify the key molecular participants. We used information-theoretic algorithms including Context Likelihood of Relatedness (CLR), Algorithm for the Reconstruction of Accurate Cellular Networks (ARACNE), and Inferelator. We captured direct functional associations among genes, given a compendium of gene expression profiles of human lung epithelial cells. A set of genes differentially expressed in COPD, as reported in a previous study were superposed with the resulting transcriptional regulatory networks. After factoring in the properties of the networks, an established COPD susceptibility locus and domain-domain interactions involving protein products of genes in the generated networks, several molecular candidates were predicted to be involved in the etiology of COPD. These include COL4A3, CFLAR, GULP1, PDCD1, CASP10, PAX3, BOK, HSPD1, PITX2, and PML. Furthermore, T-box (TBX) genes and cyclin-dependent kinase inhibitor 2A (CDKN2A), which are in a direct transcriptional regulatory relationship, emerged as preeminent participants in the etiology of COPD by means of senescence. Contrary to observations in neoplasms, our study reveals that the expression of genes and proteins in the lung samples from patients with COPD indicate an increased tendency towards cellular senescence. The expression of the anti-senescence mediators TBX transcription factors, chromatin modifiers histone deacetylases, and sirtuins was suppressed; while the expression of TBX-regulated cellular senescence markers such as CDKN2A, CDKN1A, and CAV1 was elevated in the peripheral lung tissue samples from patients with COPD. The critical balance between senescence and anti-senescence factors is disrupted towards senescence in COPD lungs.

## Author Summary

Chronic obstructive pulmonary disease or COPD is among the most lethal of respiratory diseases. While this disease has been well characterized, more studies are needed to learn the interaction of macromolecules involved in the progression towards illness. We explored possible interactions involved in the disease process using a compendium of gene expression data from frontline cells of the respiratory airways of the lung. The gene expression data were generated under a variety of experimental conditions. Application of computational schemes, which robustly detect enduring patterns, among sections of the genes represented across the varying experimental perturbations, revealed important regulatory relationships. When gene expression data from lungs of patients with COPD were factored into these networks of regulatory relationships, certain highly connected nodes (hubs) representing differentially expressed genes emerged. Notably included are members of the T-box (TBX) family of genes and CDKN2A, which regulate cellular aging. These findings were confirmed in studies using lung samples from COPD patients. Novel genes linked to TBX and CDKN2A include COL4A3, CFLAR, GULP1, PDCD1, CASP10, PAX3, BOK, HSPD1, PITX2, and PML, which were thus predicted to be involved in the disease process. The balance between senescence and anti-senescence factors is disrupted towards senescence in COPD lungs.

## Introduction

Chronic obstructive pulmonary disease (COPD) is characterized by a progressive decline in lung function, with an irreversible airflow obstruction, caused either by chronic bronchitis, emphysema or both [1]. It is a leading cause of morbidity and mortality worldwide, and thus a global health problem [2], [3]. COPD affects around 210 million people worldwide and is predicted to become the third leading cause of death worldwide by the year 2020 [4], [5]. The pathogenesis of COPD involves chronic inflammatory response in airways and lung parenchyma that results in pulmonary tissue injury, repair, and abnormal remodeling processes [6]–[9]. The pathobiology of COPD involves persistent inflammation, oxidative, and nitrosative stress, impaired cell repair and cell death manifested as senescence and apoptosis, and destruction of extracellular matrix due to protease-antiprotease imbalance in the lung tissues [1], [10].

Cigarette smoking remains the primary preventable environmental risk factor for COPD [11]. Other factors such as air pollution, respiratory infections, and aging are being recognized as critical environmental contributors to disease pathogenesis as many more people are exposed to biomass pollutants compared to tobacco smoke [10], [12]. It is unknown why only a subset of smokers as well as people exposed to similar amount of other environmental lung toxicants develops the disease. Furthermore, the disease progresses at different rates in different people exposed to similar amounts of pollutants. COPD and lung cancer are the fourth and second leading cause of deaths in the US, respectively, and it is important to understand the genes and processes that may define the bifurcations for both these debilitating diseases with high lethality [13]. A better comprehension of the host genetic susceptibility and consequent differential regulation of pathogenic processes is required to make advances in directed therapy of COPD.

Oxidative and nitrosative stress induced by cigarette smoking is thought to be responsible for corticosteroid resistance in COPD [14]–[18] [19] [20]. Oxidant–antioxidant imbalance in the lungs has been strongly implicated in COPD severity and resistance to corticosteroids [21]–[23]. Strong epidemiologic and genetic evidence indicates that an individual's ability to defend against cigarette smoke–induced oxidative stress through up-regulation of lung antioxidant defenses is important, presenting oxidative stress as a critical event in the pathogenesis of COPD [24].

Although, the understanding of the underlying mechanisms of COPD is constantly evolving, the absence of any novel or effective therapy aimed at this irreversible disease presents a significant challenge [8], [9]. There are limited effective therapies for COPD [25], [26]. Therapies such as bronchodilators provide temporary symptomatic relief, while corticosteroids are not completely effective [27] [28], [29]. Interestingly, recently, it is reported that the risk of pneumonia in patients with COPD increases with corticosteroid treatments [27]. There is no current therapy to arrest the long-term decline in lung function seen in COPD. Current therapy has not significantly decreased the mortality noted in susceptible former smokers even after years of smoking cessation. Hence, the understanding of the irreversible processes important in the pathogenesis of COPD seen in smokers may provide a means of exploiting these destructive processes and genes associated with the disease process.

To explore the molecular definition of COPD, transcriptional regulatory networks were derived from airway gene expression data. Large collections of gene expression data provide regulatory patterns that potentially bear valuable insights regarding disease mechanisms. A number of predicted molecular participants involved in COPD etiology are identified in this report, concurring with the aging hypothesis for COPD [30]. This hypothesis, based on empirical evidence as presented by Aoshiba and Nagai (2009) [30], highlights the involvement of cellular senescence in key processes that characterize COPD such as chronic inflammation, increased susceptibility to infection, emphysematous lesions, and arrested tissue repair. Notable among the identified genes and their corresponding products are members of the T-box (TBX) family transcription factors and CDKN2A, which are associated with senescence [31], [32] [33], [34]. The findings suggest that the balance between senescence and anti-senescence factors in normal smokers is disrupted towards senescence in COPD lungs.

## Results

**Figure 1:**
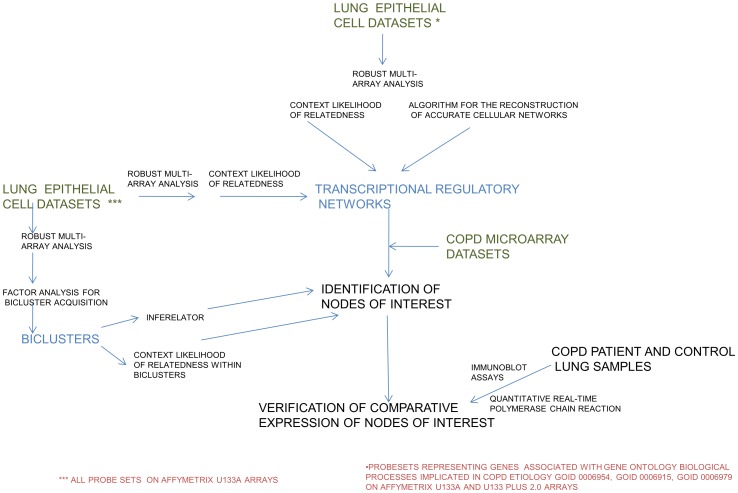
A view of the outline of the approaches used in this study. A combination of network inference and other algorithms applied to the datasets as described in the Materials and Methods section, led to the nodes of interest identified in the networks.

The experimental approach is summarized in Figure 1. Using the Context Likelihood of Relatedness (CLR) algorithm [35], data from both the U133A and the U133Plus_2 Affymetrix platforms were used to generate a transcriptional regulatory network consisting of genes involved in apoptosis, response to oxidative stress, and inflammatory response. The rationale for the focus on these processes is that they have been identified as key players in molecular pathogenesis of COPD [36] [37], [38]. A union of the generated network consisted of 535 genes (represented at the nodes in Figure S1A) and 1474 interactions (represented as connections or edges between the nodes in Figure S1). Further details are presented in Table S1.

For the purpose of identifying the most influential nodes within the overall network, two features were used. First, the size of each node is an indication of its connectedness within the network: the large size nodes are more connected in the network. The larger nodes include HMOX1, TGFB1, TBX3, CDKN2A, PML, NME1, NPM1, SMAD3, RELA, FOXL2, STAT1, IL1B, TP63, NOTCH2, NFX1, ELF3, HIF1A, NLRP3, NFRKB, E2F1, TIAL1, AATF, TBX5, TCF7L2, HTAPIP2, TNF, ITCH, NFAM1, and CREB1. Second, the color of each node is an indication of the alterations in gene expression in COPD. For this purpose, a study on the differential gene expression in 15 COPD cases and 18 controls was used [39]. As several genes were represented by multiple probe sets on the arrays, the probe set with the median gene expression level was used. Pink nodes represent genes whose expression levels were unchanged in the study. Olive-green nodes represent genes whose median probe set expressions are suppressed in COPD. These include SMAD3, TBX3, TBX5, AATF, TCF7L2, NFX1, SEMA6A, HIP1, TNFRSF19, TNFSF10, DNAJB6, AGER, PRDX5, RAC1, NFATC3, PAWR, MGLL, SCYE1, NAE1, GPX3, SIRT2, HDAC6, HDAC1, and CAT. White nodes represent genes whose median probe set expressions are elevated in COPD. These include SOCS3, MCL1, IL1B, IL6, IL8, IL24, IL1RN, TNFAIP6, CCL3, CCL4, PTX3, ADORA3, NFAM1, NLRP3, BCL10, PPARD, FAIM3, PAX3, MAPK1, PRKCA, CASP2, SERPINB9, BCL2L11, TRADD, CAV1, and CDKN2A.

**Figure 2:**
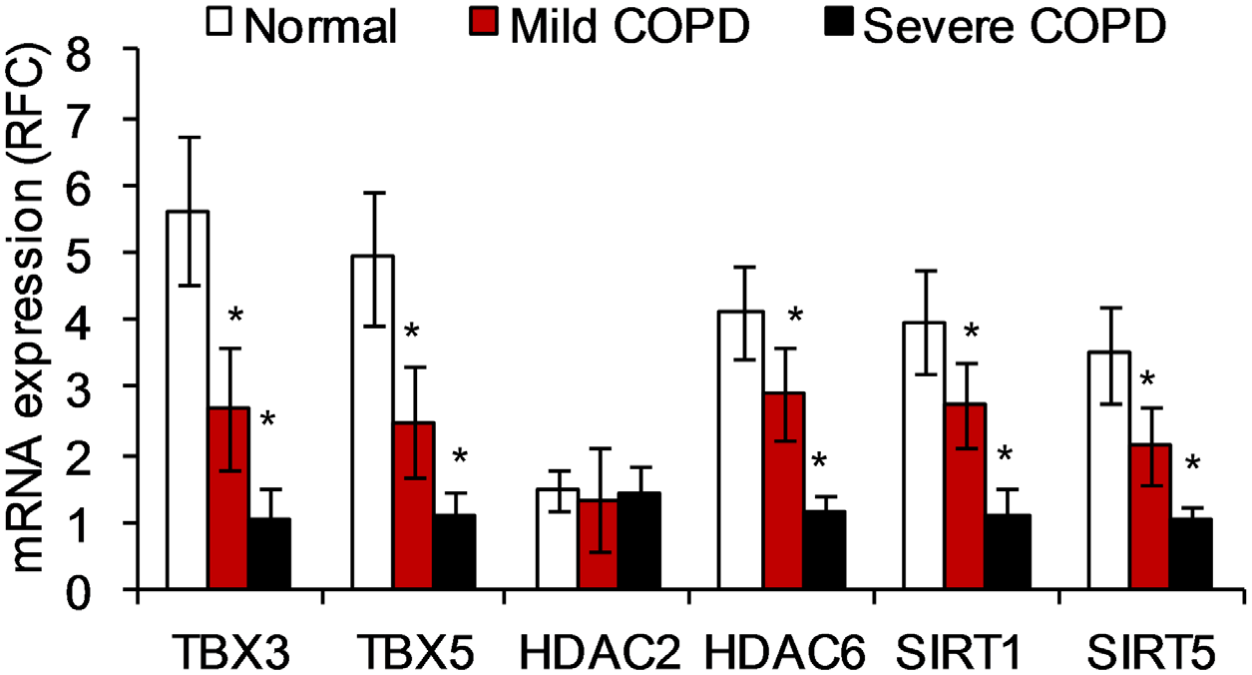
Quantitative PCR data indicate TBX3, TBX5, HDAC6, SIRT1, SIRT6 gene expression is suppressed in the lungs of patients with COPD, while mRNA expression of HDAC2 does not change compared to lung tissue from normal smokers. The extent of the suppression is highly significant between patients with mild and those with severe COPD. Patient diagnosis was based on the National Heart, Lung, and Blood Institute/World Health Organization Global Initiative for Chronic Obstructive Lung Disease (GOLD) [146]. Fifteen normal, nine mild COPD, and six severe COPD samples were used for this analysis. The data is represented as Mean ± S.D. The data was analyzed using student's t-test for comparing mRNA expression in the respective groups. *represents a significance of p-value<0.01.

Combining these two features facilitated the identification of the most connected nodes that were also differentially expressed in COPD. We hypothesize that these nodes represent genes that may be critical regulators in the etiology of the disease. Among these, the T-box (TBX) transcription factors, TBX3 and TBX5 (Figure S1B) along with the cell cycle inhibitor, CDKN2A (Figure S1B, C) were noteworthy for the extent of their connectedness. An enrichment analysis showed that genes dependent on TBX3 in the network are involved in apoptosis. However they also included genes associated with cell development, cell proliferation, and signal transduction. Of the 83 genes, 19 genes (e.g. TBX3) are involved in the cell cycle process. Similarly, of the 46 genes dependent on TBX5, 11 are involved in the regulation of the cell cycle. Quantitative real-time PCR (qRT-PCR) analysis of samples from smokers with or without COPD confirmed that relative to normal smokers without COPD, the expression of TBX3, TBX5, HDAC6, SIRT1, SIRT5, is suppressed in severe cases of COPD (Figure 2).

For confirmation purposes, a second transcriptional regulatory network was generated using the same data and an alternative algorithm, the Algorithm for the Reconstruction of Accurate Cellular Networks (ARACNE) [40]. Like CLR, ARACNE uses mutual information computed on the basis of gene expression data. ARACNE and CLR differ in their modes of discretizing and eliminating false edges. Unlike ARACNE, CLR uses B-spline functions for discretization of gene expression data [41]. Both, nonetheless, assert high connectivity for the same nodes of interest when direct as well as indirect close neighbors are considered. As summarized in Table 1, CLR was the more conservative of the two algorithms; most of the edges it predicted were predicted by ARACNE. For example, both algorithms infer the following as *direct* neighbors of TBX5 in the network generated: KNG1, SNCA, RHOB, ALOX15B, SOCS3, RHOT1, MPO, AGTR2, BCL2, MAPK1, TBX3, SEMA6A, BBC3, PRKCA, BOK, LYST, PDCD6, PTEN, ADORA1, CD74, ACTN3, NLRP12, DNM2, PDIA2, BCL2L1, CDK5R1, TRAF7, CECR2, COL4A3, PAX3, GRM4, ACTN1, HSPD1, MAPK8, CDKN2D, TIA1, and PDCD1.

**Table 1:** Proportion and list of direct neighbors also asserted in initial networks generated via other mutual information—based algorithm.

GENE	CLR	ARACNE	DIRECT NETWORK NEIGHBORS FOUND BY BOTH ALGORITHMS
**TBX3**	80/84	80/347	ACTN1 ACTN3 ACVR1B AIFM2 AVEN BAX BCL2L1 BCL2L10 BCL3 BCLAF1 BID BIRC7 BOK CASP10 CD74 CDK5R1 CDKN2D CECR2 CIDEA CLCF1 COL4A3 CRYAA DAPK2 DAPK3 DIABLO DLC1 DOCK1 F2 FOXO1 FURIN GLRX2 HIP1 IGF1R IL3 IL4I1 KIAA1967 KNG1 KRT18 LYST MAP3K10 MAPK1 MAPK8IP2 NCR1 NLRP12 NME1 NME2 NME5 NOL3 NPM1 PAX3 PCSK6 PDCD5 PDIA2 PHLDA2 PRKAA1 PRKCA PRKCZ PRLR RNF7 RTKN SCIN SEMA6A SERPINB2 SERPINB9 SFRP1 SMAD3 SOCS3 SPATA3 SST SSTR3 TBX5 TGFB1 TGFB2 TIA1 TIAL1 TLR2 TNFRSF19 TNFSF10 TP53 TRIAP1
**TBX5**	37/45	37/180	ACTN1 ACTN3 ADORA1 AGTR2 ALOX15B BBC3 BCL2 BCL2L1 BOK CD74 CDK5R1 CDKN2D CECR2 COL4A3 DNM2 GRM4 HSPD1 KNG1 LYST MAPK1 MAPK8 MPO NLRP12 PAX3 PDCD1 PDCD6 PDIA2 PRKCA PTEN RHOB RHOT1 SEMA6A SNCA SOCS3 TBX3 TIA1 TRAF7
**CDKN2A**	44/60	44/189	ADAMTSL4 ANXA1 ANXA4 API5 BBC3 BCL2 BCL2A1 BCLAF1 BTG1 CADM1 CASP8AP2 CD38 CDC2L2 CFLAR CRADD CYFIP2 DAPK2 DAXX DEDD2 EIF5A FASLG GCLC GULP1 HDAC3 HMGB1 IL1A MAPK8IP2 MCL1 NLRP1 NOTCH2 NOX5 PCSK6 PDCD1 PDIA2 PIK3R2 PML PRKCA PRKCE SPHK1 TAOK2 TGFB1 TNFRSF25 TNFRSF6B TP63

**Figure 3:**
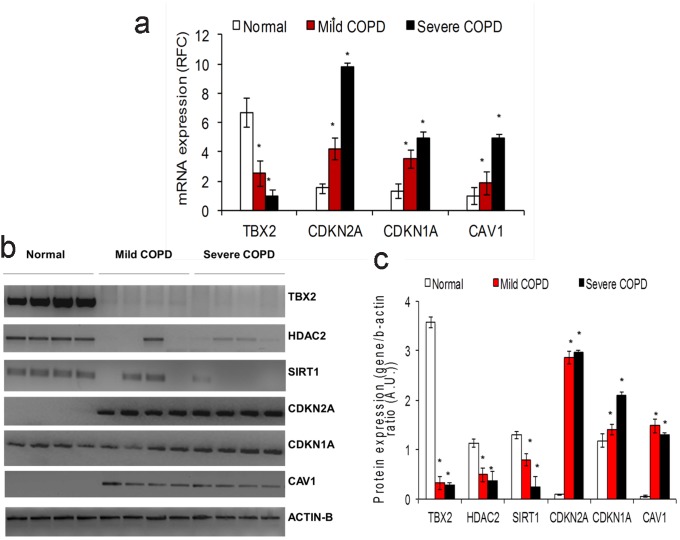
Patients with COPD have suppressed TBX2 and increased CDKN2A, CDKN1A, and caveolin-1 mRNA and protein expression. A) Quantitative PCR data indicate TBX2 gene expression is suppressed, while senescence factors, CDKN2A, CDKN1A, and caveolin-1 are induced in the lungs of patients with COPD compared to lung tissue from normal smokers. Fifteen normal, nine mild COPD, and six severe COPD samples were used for this analysis. The data is represented as Mean ± S.D. The data was analyzed using student's t-test for comparing mRNA expression in the respective groups. B) Representative Western blots showing suppressed TBX2, HDAC2, SIRT1 proteins and increased expression of CDKN2A, CDKN1A and caveolin-1 proteins in samples from patients with COPD. C) Densitometry analysis of Western blot data. Four normal, four mild COPD, and four severe COPD samples were used for this analysis. Densitometry analysis was carried out using image-J software. The data is represented as Mean ± S.D. The data was analyzed using student's t-test for comparing protein expression in the respective groups. *represents a significance of p-value<0.01.

The exploratory study was then expanded to involve all probe sets available on the Affymetrix U133A platform (removing the focus away from apoptosis, oxidative stress, and inflammation genes). All 22,283 probe sets represented after robust multi-array analysis [42] on 109 human lung epithelia arrays were used. Using the CLR likelihood estimate cut-off of 2.5 (a threshold that captured known biological associations described as part of the Discussion section), a network consisting of 17,396 nodes and 127,331 edges was generated. At this cut-off, the previously established dependencies were detected along with additional ones. Using the study by Bhattacharya et al. [39], the same coloring scheme as indicated above was used for differentially expressed genes. The T-box transcription factors remained central in this enlarged network, with the TBX2 gene emerging as one of the most connected to other genes in the network (Figure S2A). The CDKN2A node remained highly connected in this network as well (Figure S2B). It is noteworthy that a large cross-section of the genes differentially expressed in COPD was found to be dependent on TBX2 and CDKN2A in this network. (Further details are presented in Tables S2 and S3 respectively.) This expanded study revealed links for additional genes such as CAV1 and certain histone deacetylases (including certain sirtuins), whose probable roles in COPD were indicated by altered expression in COPD lung samples (Figures 2 and 3).

For confirmatory purposes, the entire dataset of 22,283 probe sets and 109 observations (Gene Expression Omnibus datasets GDS534 and GDS999) was also subjected to a bicluster analysis. Each bicluster consists of a subset of probe sets and a collection of the observations (conditions) within which they are similar [43]. Members of the observation set are similar only when the given subset of probe sets is considered and vice versa. This unsupervised learning of subsets within the larger dataset constitutes an unbiased mechanism of identifying functional associations within the data. By helping to identify relationships among the probe sets, the biclusters provided a means for re-examining the observations made in Figures S1 and S2 for possible corroboration. Using the Factor Analysis for Bicluster Acquisition (FABIA) algorithm [44], five, ten, then twenty biclusters were identified. Several probe sets grouped together in this way were also in transcriptional regulatory relationships per the CLR and ARACNE learning. As depicted in Table 2, CDKN2A, CDKN2B, COL4A3, COL4A3BP, CTNNA1, FOXJ2, FOXK2, FOXL1, FOXN3, HDAC6, HDAC9, IGF1, IGF2, IGF2BP3, PML, TBX1, TBX2, and TBX3 are all contained in the same bicluster (bicluster number 3) when ten biclusters were identified. Several of these (and related) genes also fall in the same bicluster when five and twenty biclusters are identified (Table 2). Tables S4A, S4B, and S4C contain complete listings of the membership of the various biclusters. Within-bicluster only CLR runs affirmed the regulatory relationship between TBX2 and several genes differentially expressed in COPD, as found before (Figure S3).

**Table 2:** Examples of genes within Biclusters (of five, ten, and twenty Biclusters identified using FABIA).

Number of Biclusters Learned	Bicluster Number	Probe sets Present	Arrays Present	Some Genes Present	Description of Array Samples In Bicluster
**5**	Bicluster #4	924	35	**CTNNA1, FOXO3, HDAC5, IGFBP2, IGFBP5, TBX2, WNT6**	*Thirty four bronchoalveolar lavage samples from lung transplant recipients (7 acute rejection; 27 with no rejection). In addition, there is one array from a sample obtained from one **former smoker.
**10**	Bicluster #3	1749	30	**CDKN2A, CDKN2B, COL4A3, COL4A3BP, CTNNA1, FOXJ2, FOXK2, FOXL1, FOXN3, HDAC6, HDAC9, IGF1, IGF2, IGF2BP3, PML, TBX1, TBX2, TBX3**	*Bronchoalveolar lavage samples only. The samples were from 27 lung transplant recipients who had no rejection, and 3 who had acute rejection.
**20**	Bicluster #2	2096	30	**CDKN2D, COL4A3, CTNNA1, CTNND2, FOXG1, FOXK2, FOXM1, FOXO3, HDAC2, IGFBP6, TBX2, WNT16**	*Bronchoalveolar lavage samples only. The samples were from 27 lung transplant recipients who had no rejection, and 3 who had acute rejection.
**20**	Bicluster #8	1991	44	**CDKN2A, CDKN2C, CIZ1, CTNNA1, FOXB1, FOXG1, FOXL2, FOXN3, HDAC5, IGF1, IGFBP2, IGFBP5, IGFBP7, IGF2BP3, PML, TBX1**	*Thirty four bronchoalveolar lavage samples from lung transplant recipients (7 acute rejection; 27 with no rejection). This set also includes three arrays from samples from former smokers, five from current smokers, and two from “never smokers”**.
**20**	Bicluster #13	567	56	**COL4A3BP, FOXO3, IGFBP3, TBX2**	*Thirty bronchoalveolar lavage samples from lung transplant recipients (3 acute rejection; 27 with no rejection). This set also includes seven arrays from samples from former smokers, eight from current smokers, and eleven from “never smokers”**.

*: Bronchoalveolar lavage samples obtained from lung transplant recipients whose biopsies had a perivascular score of between 0 and 2, and a bronchiolar score of between 0 and 1 [147].

**: Lung epithelial cell transcriptome study of 34 current smokers, 18 former smokers, and 23 subjects who had never smoked [148].

Furthermore, the Inferelator algorithm [45] was used to infer the transcription regulators targeting the FABIA-derived biclusters. Inferelator uses model shrinkage and standard regression to select predictive models for the expression of a gene or gene cluster, on the basis of expression levels of previously identified transcription regulators and interactions between them. Inferelator has been judged among the best performing network inference algorithms [46], [47]. As shown in Table 3 (the output when twenty FABIA-generated biclusters were fed into Inferelator), TBX3 was a predicted positive regulator of bicluster 1. TBX5 was predicted to negatively regulate biclusters 2 and 7; it also negatively regulates bicluster 20 (along with HSF1). TBX2 cooperates with PML in a predicted negative regulation of bicluster 7; it also cooperates with EP300 in a predicted negative regulation of bicluster 11. Besides the TBX family members, other regulators of note identified in Table 3 include STAT1, STAT5A, STAT5B, RUNX3, SMAD3, PML, HSF1, JUN, NFKBIB, TP53, TP63, NFE2L2, PAX3, PAX7, ATF1, ATF2, CDKN1A and FOXO3. Similar outcomes were obtained when ten FABIA-generated biclusters were fed into Inferelator; four of the ten biclusters were predicted to be TBX gene product-regulated (Figure S4).

**Table 3:** Inferelator predictions of regulators of twenty Biclusters generated using FABIA.

BICLUSTER∧	NUMBER OF PROBESETS	NUMBER OF OBSERVATIONS	CLUSTER PROBABLY POSITIVELY REGULATED BY***	CLUSTER PROBABLY NEGATIVELY REGULATED BY***
**1**	2084	32	TBX3, ELF3	ATF2_with_HSF1
**2**	2096	30	CEBPG, ATF1	PML, TP63, TBX5, TBX5_with_NFKBIB, PPARD_with_SMAD3
**3**	1560	31		CEBPG_with_TP63, NME1-NME2, TCF7L2, TIAL1
**4**	1569	38	NFE2L2_with_CDKN1A, STAT1	TCF7L2
**5**	2619	35	STAT5B_with_TP63, SMAD3	HTATIP2
**6**	1705	35	RUNX3	E2F1_and_TCFL2 , HTATIP2
**7**	1381	38	STAT1, CREBBP	E2F1, LOC652346, PML_with_TBX2, TBX5
**8**	1991	44	STAT5B, NFATC3	TCF7L2, TCF7L2_with_TIAL1
**9**	1089	33	NLRP3, SMAD3, RUNX3	
**10**	967	47	SIGIRR	
**11**	806	32	RUNX3, RUNX3_with_SMAD3	ATF2, EP300_with_TBX2, TCF7L2
**12**	566	34	TP63_and_STAT1	CEBPB, TCF7L2
**13**	567	56	FOXO3, SMAD3_with_ERC1, ELF3	STAT5A
**14**	493	50	ARHGDIA_with_RUNX3, RUNX3	PML, E2F1
**15**	754	45	ELF3	CREB1_with_TIAL1
**16**	356	43	PML_with_HSF1, LOC161527, HSF1, SBNO2	
**17**	510	46	SBNO2, JUN, RUNX3_with_CREB1,JUN_with_TP53, TP63	NFATC4_with_HTATIP2
**18**	380	61	ELF3_with_BCL10	ATF1
**19**	347	47	SMAD3	TGFB1_with_GOLGA6L4, STAT5A
**20**	497	50	HIF1A, STAT1, STAT5B, RELA	HSF1_with_TBX5
	∧Obtained using FABIA algorithm			****Per Inferelator predictions

On the basis of these results, samples obtained from patients with COPD and normal subjects without COPD were examined for the relative levels of TBX2 and CDKN2A mRNA and protein expression. As shown in Figure 3, patients with COPD had elevated expression of CDKN2A and suppressed expression of TBX2 mRNA and protein. These findings are consistent with the findings from our exploratory studies (Figures S1 and S2). In addition, other senescence factors such as CDKN1A [48] and caveolin-1 (CAV-1) [49], [50] also showed enhanced expression in samples from patients with COPD (Figures 3A, B, and C). Both these genes have critical roles in senescence pathway activation. In our regulatory network, CDKN1A is connected to TBX2 via TP53 and MMP12; TBX3 via TP53 and FH; TBX5 via TP53 and SEC14L1. A number of previous reports have shown TBX2- and TBX3-mediated regulation of senescence factor, CDKN1A [51]–[53]. Interestingly, we found that CAV-1 is connected to TBX2 via ARHGDIA and TMED2. Both ARHGDIA and TMED2 are directly linked to the master transcriptional regulator of the anti-oxidant response, nuclear factor erythroid 2-related factor 2 (NRF2), i.e. NFE2L2, which was predicted to be a bicluster regulator along with CDKN1A (Table 3; also a regulator in one of ten FABIA-generated biclusters, Figure S4).

**Figure 4:**
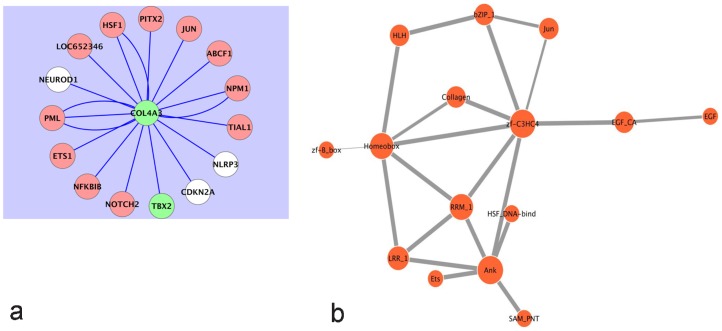
A) The state of the Type IV collagen alpha 3 subunit, COL4A3, depends on the states of both TBX2 and CDKN2A in human lung epithelial cells. Following Robust Multi-Array Analysis of a compendium of 109 Affymetrix arrays on the U133A platform, the Context Likelihood of Relatedness (CLR) algorithm was used to generate a transcriptional regulatory network involving all available probe sets (at a CLR likelihood estimate cut-off of 2.5). Olive-green nodes represent genes whose median probe set expressions are suppressed in COPD. White nodes represent genes whose median probe set expressions are elevated in COPD. COL4A3, whose expression is suppressed in the COPD lung, is thus statistically dependent on both TBX2 and CDKN2A. B) Evolutionarily conserved probable protein domain-domain interactions corresponding to the predictions of Table 5. The thickness of each edge is commensurate with the corresponding computed probabilities. The COL4A3 protein has the Collagen domain (Collagen in Pfam database; InterPro Database Accession IPR008160) and probably engages PML via its zf-C3HC4 domain (zf-C3HC4 in Pfam database; InterPro Database Accession IPR001841). By way of its Collagen domain, COL4A3 interacts with PITX2 via its Homeobox domain (Homeobox in Pfam database; InterPro Database Accession IPR001356). Among others, there is also a probable interaction between the zf-C3HC4 of PML and the Ankyrin repeat (Ank in Pfam database; InterPro Database Accession IPR002110) domain of CDKN2A that could impact the COPD etiology.

It is well known that COPD clusters in families [54]. Details of the genetic susceptibility loci continue to be studied. It has been determined that a polymorphism of the type IV collagen alpha3 (COL4A3) gene is associated with the risk of developing COPD [55]. Subjects with carriers of 451HH with at least one 451R allele had a higher COPD risk, which is more pronounced in younger subjects. Interestingly, in our analysis, COL4A3 expression was found to be suppressed in COPD. An examination of its expression within the networks generated indicated dependence on TBX genes (Figure 4A and Figures S1B, and S1C). Further, Figure 4A shows that the expression of COL4A3 depends on both CDKN2A and TBX2.

Taken together, these results indicate a critical balance between senescence and anti-senescence factors in normal smokers, which is disrupted towards senescence in COPD lungs. There are previous reports of decline in telomere length, which is a hallmark of senescence in samples from patients with COPD [49], [56]–[58]. On the other hand, increase in anti-senescence activity is reported as a hallmark of cancer [59]–[64] including lung cancer which, like COPD, is associated with smoking. These observations are in agreement with recent reports that highlight aging and associated senescence pathway as the key pathogenic molecular pathways involved in chronic lung diseases including lung cancer [65] and COPD [66]–[69].

## Discussion

Using a variety of computational approaches, a number of regulatory genes important in COPD have been identified in these studies. First, using gene expression data of genes associated with three Gene Ontology biological processes implicated in COPD, transcriptional regulatory networks were learned by way of CLR and ARACNE. The most highly connected nodes of the networks which simultaneously represented genes differentially expressed in COPD were noted as important. The basic findings were also present in an expanded CLR study of all 22,283 probesets on the U133A Affymetrix platform. Differentially expressed and highly connected nodes of note included TBX2, TBX3, TBX5, and CDKN2A.

Bicluster analyses of the entire U133A platform dataset, which were unbiased in terms of a prior determination of genes of focus, found that the genes related to those noted in the CLR and ARACNE studies clustered together, often co-occurring in more than one bi-cluster (Table 2). CLR-generated regulatory networks involving only genes occurring within the same biclusters affirmed a central regulatory role for TBX2 (Figure S3). In addition, Inferelator, which uses a very different approach from CLR and ARACNE, predicted that TBX gene products are involved in the overall regulation of between 25% and 40% of identified biclusters (Table 3, Figure S4). Thus, among a variety of computational approaches, there was a consensus regarding a regulatory role for TBX gene products in COPD.

T-box proteins are an important family of transcription factors. Over 20 genes in vertebrates have a region of homology to the DNA-binding domain of the transcription factor encoded by Brachyury, the T gene [70]. The region of homology in the gene product, called the T-box, has approximately 180 amino acid residues and is highly conserved across several species [71]. TBX transcription factors are characterized by a highly conserved DNA-binding T-box domain, which recognizes a consensus core sequence GGTGTGA called the T-sites, and this domain is different from any other known DNA-binding motif [72]. A variety of T-box proteins serve as activators or repressors of their target genes, depending on cofactors involved [70], [73] [74], [75]. T-box proteins are critically important during development [76]. Importantly, with regard to COPD, T-box proteins are inhibitors of senescence [31], [32]. TBX3, for instance, inhibits senescence in a process involving p53-dependent proliferation arrest [32].

CDKN2A is a mechanistic marker for cellular senescence [33], [34]. The CDKN2A gene generates transcript variants that include p16INK4A and ARF. The cyclin-dependent kinase, CDK4, which is critical for cell cycle turnover, is inhibited by the p16INK4A product [77]. The other CDKN2A product, ARF, promotes the degradation of the p53 inhibitor, MDM2 and consequently promotes the accumulation and stabilization of p53 [78]. The accumulated p53 is subsequently able to activate the expression of genes involved in the arrest of the cell cycle at G1 or in apoptosis [79]. Thus, by arresting the progression of the cell cycle to promote repair of damaged DNA or by inducing apoptosis; p53 prevents accumulation of mutations that can be oncogenic.

Thus, the activities of both T-box proteins and the CDKN2A products converge on the p53 pathway. Our findings (Figure S1C) underscore the roles of T-box proteins and CDKN2A in the etiology of COPD and indicate that the expressions of these genes are linked in the human lung epithelium. Of note are the changes in expressions of TBX (-2, -3) and CDKN2A occur in opposite directions in cancer. T-box genes/proteins such as TBX2 and TBX3 are overexpressed in several neoplasms, including melanomas, breast, and pancreatic cancer [53], [80], [81]. On the other hand, because of its effect on MDM2, the CDKN2A product, ARF, acts as a tumor suppressor; consequently its loss is associated with neoplasms [82]. The other CDKN2A product, p16INK4A, also suppresses tumors, and is itself suppressed in neoplasms [77]. Secondly, in COPD, we show that expressions of TBX (-3, -5) are suppressed and expression of CDKN2A increases (Figures 2 and 3A and Figures S1B, and S1C). This is in distinct contrast to prior observations made in cancer. TBX2 belongs to the TBX2 sub-family of TBX transcription factors that include TBX3, TBX4, and TBX5 genes [71]. Protein expression of TBX2 also declines in the lungs of patients with COPD compared to control subjects, while the expression of CDKN2A protein is elevated in the lungs of patients with COPD compared to control subjects (Figures 3B and 3C). We also show an association between expression levels of TBX3, TBX5, and CDKN2A, an indication they are statistically associated (Figure S1C). TBX3 down-regulates the expression of the CDKN2A product, ARF [32]. Similarly, via the stress-activated p38 MAP kinase, the activated TBX2 localizes in the nucleus and represses the closely related CDKN1A (p21) promoter [51]; CDKN1A is an inhibitor of DNA repair [83], [84]. In Figures S2A and S2B, we show that both TBX2 and CDKN2A are highly connected in the human lung epithelium transcriptional regulatory network. We also show that many of the genes differentially expressed in the COPD lungs are statistically dependent on the expression of TBX2 and CDKN2A.

TBX2 and its close relative, TBX3, negatively regulate cell cycle control genes and CDKNs, specifically CDKN2A, CDKN2B, and CDKN1A [51], [53] and are often upregulated in several cancers [53], [80], [85]. Additionally, in carcinogenesis, TBX2 destabilizes p53 by inhibition of ARF [86], while the retinoblastoma protein (Rb1) is an important modifier of TBX2 function [87]. Taken together, TBX2 and TBX3 act as suppressors of senescence factors such as CDKNs, and hence, suppress senescence especially in cancer cells; thus, TBX2 and TBX3 are thought to be critical anti-senescence factors [51], [52], [88], [89].

As reported here, the expression of the anti-senescence T-box transcription factors are suppressed in the COPD lungs, and there is a concomitant rise in the expression of the cellular senescence markers, CDKN2A, CDKN1A, and CAV-1 (Figure 3). CDKN1A mediates cigarette smoke-induced inflammation [48]; cigarette smoke increases expression of CAV-1 which is involved in senescence and pulmonary emphysema induction [49], [50]. Thus these findings support the aging hypothesis for COPD [30]. The two important relevant COPD risk factors converge. First, the incidence of COPD increases with age [90]. Secondly, cigarette smoking has been associated with the incidence of COPD. These associations suggest senescence in the lungs is probably due to the exposure to cigarette smoke. Indeed, studies in human lung epithelial cells and mice indicate that cigarette smoke induces senescence [91]. Cellular senescence is often associated with shortening of telomeres and is relevant to tissue aging [92]. One of the hallmarks of senescent cells is their tendency to generate a number of pro-inflammatory cytokines [93].

### COPD Susceptibility Locus

Genome-wide studies in patients with COPD indicate that the chromosomal locus spanning 2q33.3–2q37.2 is associated with COPD [94] [95]. Among the genes linked by the CLR algorithm to TBX5 (Figure S1C), HSPD1, COL4A3, and PAX3 fall within this region, and BOK and PDCD1 lie on the edge of this locus (Table 4). HSPD1, also known as HSP60 or HSP65, is a member of the heat shock protein family. As shown in Figure S1C HSPD1, which has elevated expression in COPD is linked to the expression of TBX5, one of the central transcription factors with suppressed expression in COPD. Notably, HSF1 [96], a transcription factor responsible for the transcriptional activation of several heat shock proteins, is linked to COL4A3 (Figure 4A), which falls within a chromosomal locus of interest. Also, a polymorphism of the COL4A3 gene is associated with the risk of developing COPD [55].

**Table 4:** Several chromosome 2 genes linked to senescence hubs in transcriptional regulatory network fall within COPD susceptibility locus, 2q33.3–2q37.2.

Hub	Direct Neighbor	Chromosomal Location
CDKN2A	ANXA4	2p13
	**CFLAR**	**2q33–34**
	**GULP1**	**2q32.3–q33**
	IL1A	2q14
	**PDCD1**	**2q37.3**
TBX3	**CASP10**	**2q33–34**
	**PAX3**	**2q35**
	RTKN	2p13.1
	TIA1	2p13
TBX5	**BOK**	**2q37.3**
	**COL4A3**	**2q36–37**
	**HSPD1**	**2q33.1**
	**PAX3**	**2q35**
	**PDCD1**	**2q37.3**
	PRKCE	2p21
	RHOB	2p24
	TIA1	2p13

Genes in bold character are in or border the region of the susceptibility locus.

COL4A3 [97] has suppressed expression in COPD (Figure S1 and Figure 4A), and is tied by the CLR algorithm to the transcription factors PML (also a tumor suppressor) and PITX2. PML and PITX2 have protein domains that likely interact with COL4A3 (Figure 4B, Table 5). By way of its Collagen domain, COL4A3 probably interacts with PITX2 via the PITX2 Homeobox domain (Homeobox in Pfam database; InterPro Database Accession IPR001356). PITX2 acts downstream of the Wnt-β-catenin pathway and responds to the activation of that pathway, by regulating the transcription of G1 cell cycle control genes such as cyclin D1 and c-Myc [98]. The dependence between PML and COL4A3 (per the CLR runs) is similarly interesting, because PML, a regulator of the cell cycle, plays a critical role in the regulation of cell proliferation, apoptosis, and senescence [99]. It induces a block of the G1 phase of the cell cycle in tumor cell lines [100] and enhances the transcriptional activity of the key tumor suppressors such as p53 and Rb [99]. The COL4A3 protein has the Collagen domain (Collagen in Pfam database; InterPro Database Accession IPR008160) and probably engages PML via its zf-C3HC4 domain (zf-C3HC4 in Pfam database; InterPro Database Accession IPR001841) (Figure 4B, Table 5). Our results also show a transcriptional regulatory relationship between PML and CDKN2A (probably by an interaction between the zf-C3HC4 of PML and the Ankyrin repeat domain of CDKN2A (Figure 4B, Table 5)). However, the significance of this observation for COPD remains unclear.

**Table 5:** Maximum likelihood estimation indicating probabilities of protein-protein interactions based on evolutionarily conserved domain-domain interactions.

	MLE Probability	
CDKN2A	0.99	ETS1
CDKN2A	1	NFKBIB
CDKN2A	0.94	NLRP3
CDKN2A	1	NOTCH2
CDKN2A	1	PML
CDKN2A	0.99	TIAL1
**COL4A3**	**0.68**	**PITX2**
**COL4A3**	**0.9**	**PML**
ETS1	0.99	NFKBIB
ETS1	0.99	NOTCH2
HSF1	0.9	NFKBIB
HSF1	0.9	NOTCH2
JUN	0.99	NEUROD1
NEUROD1	0.99	PITX2
NLRP3	0.94	NFKBIB
NLRP3	0.94	NOTCH2
NLRP3	0.99	PITX2
NLRP3	1	TIAL1
NOTCH2	0.99	ETS1
NOTCH2	1	NFKBIB
NOTCH2	1	PML
PML	1	JUN
PML	1	NFKBIB
PML	1	NOTCH2
PML	1	PITX2
TIAL1	0.99	NFKBIB
TIAL1	0.99	NOTCH2
TIAL1	0.99	NOTCH2
TIAL1	1	PITX2
TIAL1	1	PITX2
TIAL1	1	PML

MLE = Maximum likelihood estimation.

Probable interactions involving COL4A3, which is in the susceptibility locus, are in bold character.

#### Other senescence genes

The importance of TBX2 and CDKN2A in the network has been highlighted. Other gene products associated with senescence were found via the CLR (using all available probe sets) to be statistically associated with, and thus probably dependent on, TBX2 and/or CDKN2A (Table 6). They include insulin/IGF-1 signaling genes which promote aging [101]–[103]; members of the forkhead family [104] [105] [106]; members of the WNT family and the bipartite transcription factor β-catenin/TCF [107] [108] [109] [110] [111] [106]; as well as histone deacetylases (HDACs) and sirtuins (SIRTs) [112] [113]. Furthermore, the association of TGBF1 with senescence-related genes in our study (Table 6) is an indication of a probable role in the etiology of COPD [114] [115].

**Table 6:** Several aging-related genes are statistically linked to the expressions of TBX2, CDKN2A, and TGFB1.

	TBX2	CDKN2A	TGFB1
**Catenin-related genes**	CTNNA1, CTNND1		CTNNBIP1
**Forkhead transcription factor-related genes**	FOXA2, FOXB1, FOXD3, FOXH1, FOX01, FOXO3		FOXA1, FOXJ1, FOXO4
**Insulin Growth factor-related genes**	IGF2, IGF2BP3, IGFBP7, IGFBP5	IGFALS	IGFALS, IGFBP4, IGF2PB2, IGFBP7, IGFBP5, IGF2R, IGFBP3
**Interleukin-related genes**	IL4, IL13, IL13RA1, IL27RA, IL1RL1, IL17RC, IL10RB, IL2RA, IL12RB1	IL1RN, IL6ST, IL1RAPL1	IL20RA, IL27RA, IL13RA1, IL2RG, IL10RA, IL4I1
**Wnt-related genes**	WNT3, WNT4, WNT6, WNT7B, WNT11	WNT10B	

Our study furthers the paradigm on cellular senescence as its effectors and their regulation are the cross-roads of smoking-induced lung cancer or COPD [13], [116], [117]. Along with T-box transcription factors, other anti-aging molecules such as HDACs and SIRTs are decreased in the lungs of patients with COPD compared with smokers without COPD. This results in enhanced inflammation that furthers the progression of COPD [118], [119]. The genes identified here may serve as mechanistic biomarkers for detecting impending physiological changes during the disease process. Further, this understanding can help in designing directed therapies with further understanding of genomic, molecular, and physiological changes in patients with emphysema or COPD [120], [121].

## Materials and Methods

### Ethics Statement

The study protocols were approved by the Institutional Review Board for human studies, and patients' lung function data from each of the contributing centers were obtained for this study.

### Etiology of COPD and Microarray

The overall experimental approach is summarized in Figure 1. The etiology of COPD has been associated with apoptosis [122], [123], oxidative stress [124], and inflammation [125]. The computational aspects of these studies were conducted in two phases. During the first phase, transcriptional regulatory networks of the human lung epithelium were reverse-engineered from publicly available gene expression data, using the CLR network inference algorithm [35]. Subsets of a compilation of publicly available gene expression data were generated based on genes classified under the Gene Ontology [126] term “inflammatory response” (GOID 0006954), “apoptosis” (GOID 0006915), and “response to oxidative stress” (GOID 0006979), using a program written for that purpose in lisp [127]. Thus, for each microarray platform (Affymetrix U133A and U133Plus_2), a transcription regulatory network was generated that consists of a union of statistical dependencies among the genes involved in inflammatory response, apoptosis, and response to oxidative stress. For comparison and confirmation purposes, a second transcriptional regulatory network was generated using ARACNE and the same datasets. Like CLR, ARACNE uses mutual information computed on the basis of gene expression data, as discussed below. ARACNE and CLR differ in their modes of binning and eliminating false edges. CLR was the more conservative of the two algorithms, most of the edges it asserted also having been asserted by ARACNE (Table 1).

Expanding on the findings of the first phase, the entire set of probe sets represented on 109 arrays of the U133A platform was used during the second phase in which the CLR algorithm was executed (Gene Expression Omnibus datasets GDS534 and GDS999). Subsequently to assure the reliability of the regulatory relationships just described, biclusters were identified within the dataset using the FABIA algorithm. The Inferelator algorithm was then also used to predict regulators of those biclusters.

### Public Gene Expression Data

A compendium of microarray data was generated from the Gene Expression Omnibus (GEO). GEO record numbers GDS534, GDS999, GDS2604, and GDS2486 (http://www.ncbi.nlm.nih.gov/geo/) and contains the gene expression microarray experiment data from human lung epithelial cells under a variety of conditions. These arrays are based on the Affymetrix (http://www.affymetrix.com) human U133A and U133Plus_2 platforms. In all, there were 109 arrays from the human U133A and 49 arrays from the human U133Plus_2 platforms, respectively. For each platform, gene expression data files in the .CEL format were downloaded and subjected to Robust Multiarray Analysis [42], using the Bioconductor (http://www.bioconductor.org) package *affy* in R (http://cran.r-project.org/).

### CLR

The CLR algorithm [35] is an improvement on the Relevance Networks algorithm [128]. Both use the concept known as mutual information to infer the state of one member of a given gene pair, given the state of the other member of the pair [129], [130]. There is a need to capture biologically relevant links within the resulting reverse-engineered networks. In relevance networks, mutual information score thresholds are applied. Low thresholds tend to capture dense networks with many false positives which inevitably include misrepresentations of indirect dependencies as direct interactions. On the other hand, high thresholds result in much smaller networks albeit with fewer false positives. The CLR uses an adaptive background correction step to remove indirect influences and false correlations. It compares the mutual information value for a given pair of genes to a background distribution of mutual information scores.

Thus a likelihood estimate:
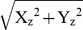
is used (where X_z_ is the z-score of the mutual information between gene X and gene Y in gene X's mutual information score distribution, and Y_z_ is the z-score of the mutual information between gene X and gene Y in gene Y's mutual information score distribution).

#### Mutual information

Given two random variables, X and Y, the mutual information between them is given by:
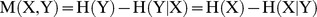


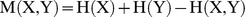
(Here the Shannon entropy [130], the probability of observing a particular symbol or event, p_i_,
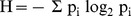
is used as a measure of quantitative information).

In other words, the shared information between X and Y corresponds to the remaining information of one party if we remove the information of that party that is not shared with the other party. For two genes, X and Y, the mutual information is given by:
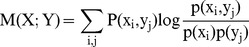
x_i_ and y_i_ represent specific expression levels across a given set of measurements. The mutual information thus ranges between 0 and 1, and is a measure for dependencies in the data: negative or positive, nonlinear or linear [131], [132]. The higher the mutual information score between the two genes, the greater the information inferred on the states of the first gene from the pattern of states in the second.

#### CLR execution

The code implementation provided by Faith et al. was used from a Linux command line [35]. During the first phase, the CLR runs were conducted on the data subsets outlined above. For each data subset, gene subsets that are also identified in the Gene Ontology as transcription factors using GeneInfoViz [133] were designated as transcription regulators as part of the execution of the algorithm. Other details of this CLR run are in Table S5.

In the second phase, all 22,283 probe sets represented after background correction and normalization on the U133A platform were used (Gene Expression Omnibus datasets GDS534 and GDS999). A likelihood estimate cut-off value of 2.5 used generated a network consisting of 17,396 nodes and 127, 331 transcription regulatory links.

### ARACNE

Like CLR, ARACNE uses mutual information [40]. Unlike CLR, it uses the Data Processing Inequality (DPI) to retain only those regulatory relationships that are direct (rather than indirect) [134]. In other words, if genes g1 and g3 interact only through a third gene, g2, then DPI indicates:
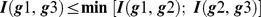
Thus of the trio, the edge with the least value gets eliminated. The “DPI tolerance” used for ranking of *I* values, to minimize the impact of I value variance was set at 0.15 in this study. DPI tolerance values of greater than 0.2 have been determined to yield high false positive edges by the developers of ARACNE. Furthermore, the threshold p-value for establishing that the mutual information between gene pairs was significant in this study was set at 10^−7^.

### Factor Analysis for Bicluster Acquisition (FABIA)

The algorithm [44] was run using the entire dataset derived from the 109 U133A arrays. For *p* biclusters and additive noise, the model for the matrix *X* (input to biclustering method) is:
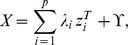
where the real numbers λ, *z*_i,_ and Υ, are the sparse prototype column vector, the sparse vector of factors (transposed as rows) with which the prototype vector is scaled for the *i*th bicluster, and the additive noise respectively. For each of the bicluster sets (where *p* is 5, 10 or 20), there were 500 iterations, and a sparseness factor of 0.1. All parameters were set at the default values.

### Inferelator

The Inferelator [45] version 1.0 was used to infer a minimal set of regulators that explains the expression levels of each of the 10 and 20 biclusters identified. Potential regulators were defined (as was done for the CLR execution), and the expression data was treated as equilibrium observations (i.e. no information about temporal relationships between observations was incorporated into the inference process). The Inferelator was run with default settings.

### Previous COPD Studies and Network Graphics

The human lung transcription regulatory networks generated were subsequently analyzed in the light of GEO datasets GSE1122, GSE1650, and GSE8581, representing studies on changes in gene expression between emphysema subjects and control subjects [135], patients with severe COPD and patients with symptoms ranging from mild COPD to normal [136], and patients with COPD and control patients [39]. CEL files were downloaded, and in each case, the data analyzed for differential gene expression using Partek workbench [137] after Robust Multiarray Analysis [42] processing (p-value = 0.01 and false discovery rate = 0.01) [138]. For each gene represented by differentially expressed probe sets, the median probe set value was used to represent the level of expression during visualization in Cytoscape [139]. Genes suppressed in COPD (compared to controls) were depicted using olive green-colored nodes and up-regulated genes had white-colored nodes. In our studies GSE8581 data [39] were used.

### Domain Analysis

Proteins interact with each other via their component domains. An accurate prediction of domain-domain interactions would facilitate the prediction of protein-protein interactions. The Pfam database [140] contains a large collection of evolutionarily conserved protein domains and empirically determined interactions they are involved in. Based on relevant Pfam domain family data, a Maximum Likelihood Estimation method was used to infer protein-protein interactions among connected nodes of the transcriptional regulatory networks generated as described above [141] [142].Although the networks were generated on the basis of gene array data, they are proxies for interactions among corresponding gene products (proteins). Indeed genes with similar expression patterns often generate proteins that interact in some fashion [143], [144]. An implementation of the maximum likelihood estimation in Cytoprophet [145] was used, and probabilities of domain-domain (protein-protein) interactions were computed.

### COPD Patient and Normal Lung Samples

Frozen peripheral lung tissue samples used in this study were obtained from two tissue banks: (1) the NHLBI Lung Tissue Research Consortium (University of Colorado Health Sciences Center, Denver, CO); and (2) the iCAPTURE (James Hogg iCAPTURE Centre for Cardiovascular and Pulmonary Research, St. Paul's Hospital, University of British Columbia, Vancouver, BC, Canada). We obtained data on patients' lung function from both established patient registries. Clinical information, samples size, and classification based on Global Initiative for Obstructive Lung Disease (GOLD) for Chronic Obstructive Lung Disease stages of patients with COPD and normal control subjects are summarized in Table 7. Participants in the COPD groups who smoked had similar pack-year smoking histories, where smoking for 1 pack-year refers to smoking one pack of cigarettes per day each year.

**Table 7:** Patient characteristics.

Characteristic	Normal Samples	COPD Samples
GOLD Stage (1/2/3/4)	15	0/9/6/0
Sex (Male/Female)	15/0	15/0
Age (mean ± SD), years	69.9±14.4	68.4±15.1
Pack years smoked, (mean ± SD)	51.4±11.1	58.3±8.3
FEV_1_ % predicted, (mean ± SD)	95.1±9.3	31.3±29.9
FVC % predicted, (mean ± SD)	88.7±8.9	60.1±18.1

FEV_1_ = Forced expiratory volume at 1 sec; FVC = Function vital capacity; SD = Standard deviation; COPD = Chronic obstructive pulmonary disease.

GOLD (Global Initiative for Chronic Obstructive Lung Disease) Stages:

1 – mild COPD: FEV_1_≥80% predicted, FEV_1_:FVC<70%.

2 – moderate COPD: 50%≤FEV_1_≤80% predicted, FEV_1_:FVC<70%.

3 – severe COPD: 30%≤FEV_1_≤50% predicted, FEV_1_:FVC<70%.

4 – very severe COPD: FEV1<30%predicted or FEV_1_<50% predicted with chronic respiratory failure, FEV_1_:FVC<70%.

Pack years: (Packs smoked per day)×(years as a smoker).

### Quantitative Real-Time Polymerase Chain Reaction (qRT-PCR)

Selected genes (TBX2, TBX3, TBX5, CDKN2A, CDKN1A, HDAC2, HDAC5, SIRT1, SIRT5, and CAV1) from our analysis were validated by qRT-PCR. Total mRNA from the peripheral lung tissues from patients with COPD and non-COPD individual's lungs were purified using the Qiagen RNeasy kit (Qiagen, Valencia, CA). qRT-PCR was then performed using inventoried Assay-on-Demand primers and probe sets from Applied Biosystems (Foster City, CA). We used the ABI 7000 Taqman system (Applied Biosystems) to perform these assays. β-actin was used as a normalization control. The analysis was run as previously described [19].

### Immunoblot Assay

Immunoblots were performed using antibodies for TBX2, CDKN2A, CDKN1A, SIRT1, CAV1, HDAC2, and ACTIN-B (Santa Cruz Biotechnology, Santa Cruz, CA). ACTIN-B was used as a loading control. These immunoblots were performed using protocols as described previously [19].

### Statistical Analyses for q-RT-PCR and Immunoblot Analysis

Fifteen normal, nine mild COPD, and six severe COPD samples were used for q-RTPCR analysis. Four samples per group were used for immunonoblots. All immunoblots were quantified by measuring scanned photographs in ImageJ software (NIH). All statistical analyses were done with student's t-test for comparisons of COPD groups with normal samples as control. Data in graphs were represented as mean values and error bars in the graphs represent standard deviation (SD).

## Supporting Information

Figure S1 CLR-Generated transcriptional regulatory network of human lung epithelial cells. Following Robust Multi-Array Analysis of a compendium of 158 Affymetrix arrays, the Context Likelihood of Relatedness (CLR) algorithm was used to generate a transcriptional regulatory network (false discovery rate, 0.05). A) A synoptic view of the overall lung epithelial transcriptional regulatory network generated using Gene Ontology genes associated with apoptosis, response to inflammation, and response to oxidative stress. B) An up-close view of TBX3 and nodes directly connected to it in the network generated. C) An up-close view of TBX5 and nodes directly connected to it in the network generated. Larger-sized nodes represent hubs within the network, i.e. human lung epithelium cell genes more highly connected to other genes associated with apoptosis, response to inflammation, and response to oxidative stress. Olive-green nodes represent genes whose median probe set expressions are suppressed in COPD. White nodes represent genes whose median probe set expressions are elevated in COPD.
(TIF)

Figure S2 The states of a large cross-section of human epithelial cell genes differentially expressed in COPD depend on the states of (A) TBX2 and (B) CDKN2A. Following Robust Multi-Array Analysis of a compendium of 109 Affymetrix arrays on the U133A platform, the Context Likelihood of Relatedness (CLR) algorithm was used to generate a transcriptional regulatory network involving all available probe sets (at a CLR likelihood estimate cut-off of 2.5). Olive-green nodes represent genes whose median probe set expressions are suppressed in COPD. White nodes represent genes whose median probe set expressions are elevated in COPD. TBX2 gene expression is suppressed while CDKN2A gene expression is elevated in COPD.
(TIF)

Figure S3 TBX2 is statistically associated with a large cross-section of genes differentially expressed in the COPD lung. Following Robust Multi-Array Analysis, ten biclusters were identified using FABIA from a compendium of 109 Affymetrix human lung epithelial cell microarrays. Focusing only on genes present in each cluster, the Context Likelihood of Relatedness (CLR) algorithm was used to generate Transcriptional Regulatory Networks (false discovery rate, 0.05). The ten networks are merged in this figure. Olive-green nodes represent genes whose median probe set expressions are suppressed in COPD. White nodes represent genes whose median probe set expressions are elevated in COPD. TBX2, the olive node in the center, is either directly or indirectly (by way of one or two intervening nodes) linked with a significant cross-section of genes differentially expressed in the COPD lung.
(TIF)

Figure S4 TBX gene products (captured by red arrows in figure) are predicted to be involved in the direct regulation of 40% of biclusters in the dataset. Following Robust Multi-Array Analysis, ten biclusters were identified using FABIA from a compendium of 109 Affymetrix human lung epithelial cell microarrays. Inferelator version 1.0 was used to infer a minimal set of regulators that explain the expression levels of each of 10 biclusters. TBX2 was predicted to be involved in the direct regulation of biclusters 6 and 10. TBX3 was predicted to be involved in the direct regulation of bicluster 10. TBX5 was predicted to be involved in the direct regulation of biclusters 2 and 8.
(TIF)

Table S1 Interacting nodes in CLR-generated network and their corresponding likelihood estimates. The data in this table correspond to Figure S1.
(DOC)

Table S2 Direct Connections to TBX2 in the CLR-Generated Network. The data in this table correspond to Figure S2A.
(DOC)

Table S3 Direct Connections to CDKN2A in the CLR-Generated Network. The data in this table correspond to Figure S2B.
(DOC)

Table S4 A: Membership of *Five* Biclusters Learned Using Factor Analysis for Bicluster Acquisition (FABIA). This table identifies the genes and phenotypes that clustered together when the algorithm was applied to learn *five* biclusters from the experiments in the compendium. B: Membership of *Ten* Biclusters Learned Using Factor Analysis for Bicluster Acquisition (FABIA). This table identifies the genes and phenotypes that clustered together when the algorithm was applied to learn *ten* biclusters from the experiments in the compendium. C: Membership of *Twenty* Biclusters Learned Using Factor Analysis for Bicluster Acquisition (FABIA). This table identifies the genes and phenotypes that clustered together when the algorithm was applied to learn *twenty* biclusters from the experiments in the compendium.
(DOC)

Table S5 Details of CLR Execution that Generated Results Found in Figure S1 and Table S1.
(DOC)
