# Correction: Suppressed Expression of T-Box Transcription Factors Is Involved in Senescence in Chronic Obstructive Pulmonary Disease

**DOI:** 10.1371/journal.pcbi.1005263

**Published:** 2017-01-27

**Authors:** The PLOS Computational Biology Staff

At the recommendation of the Johns Hopkins Bloomberg School of Public Health following an internal review, this article is being corrected.

The following references cited within this article have been retracted:

17. Malhotra D, Thimmulappa R, Navas-Acien A, Sandford A, Elliott M, et al. (2008) Decline in NRF2-regulated antioxidants in chronic obstructive pulmonary disease lungs due to loss of its positive regulator, DJ-1. *Am J Respir Crit Care Med* 178: 592–604. 10.1164/rccm.200803-380OC.

19. Malhotra D, Thimmulappa R, Vij N, Navas-Acien A, Sussan T, et al. (2009) Heightened endoplasmic reticulum stress in the lungs of patients with chronic obstructive pulmonary disease: The role of Nrf2-regulated proteasomal activity. *Am J Respir Crit Care Med* 180: 1196–1207. 10.1164/rccm.200903-0324OC.

20. Malhotra D, Thimmulappa RK, Mercado N, Ito K, Kombairaju P, et al. (2011) Denitrosylation of HDAC2 by targeting Nrf2 restores glucocorticosteroid sensitivity in macrophages from COPD patients. *J Clin Invest* 121: 4289–4302. 10.1172/JCI45144.

Corrections to this article are as follows:

Fig 3 is incorrect because of errors made in the preparation of the images in panels B and C. The corrected figure and legend appear below.

**Fig 3:**
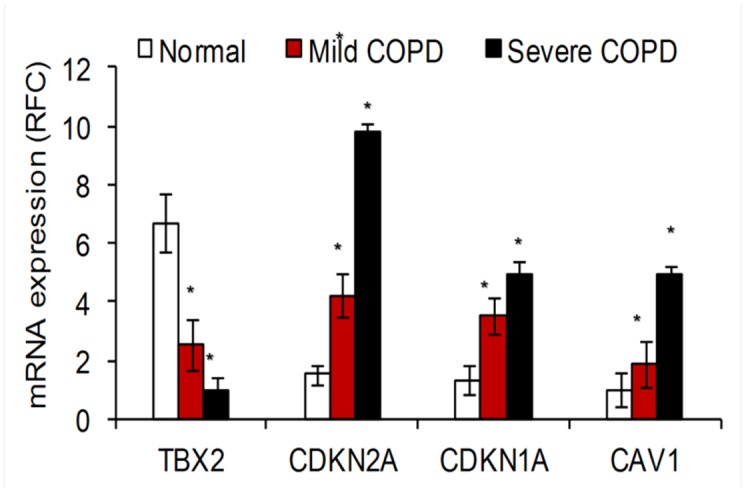
Patients with COPD have suppressed TBX2 and increased CDKN2A, CDKN1A, and CAV1 mRNA expression. Quantitative PCR data indicate TBX2 gene expression is suppressed, while senescence factors, CDKN2A, CDKN1A, and CAV1 are induced in the lungs of patients with COPD compared to lung tissue from normal smokers. Fifteen normal, nine mild COPD, and six severe COPD samples were used for this analysis. The data is represented as Mean ± S.D. The data was analyzed using student’s t-test for comparing mRNA expression in the respective groups. *represents a significance of p-value<0.01.

In the Results section, paragraph eight is corrected to:

“On the basis of these results, samples obtained from patients with COPD and normal subjects without COPD were examined for the relative levels of TBX2 and CDKN2A mRNA expression. As shown in Fig 3, patients with COPD had elevated expression of CDKN2A and suppressed expression of TBX2 mRNA. These findings are consistent with the findings from our exploratory studies (Figures S1 and S2). In addition, other senescence factors such as CDKN1A [48] and caveolin-1 (CAV-1) [49,50] also showed enhanced expression in samples from patients with COPD (Fig 3). Both these genes have critical roles in senescence pathway activation. In our regulatory network, CDKN1A is connected to TBX2 via TP53 and MMP12; TBX3 via TP53 and FH; TBX5 via TP53 and SEC14L1. A number of previous reports have shown TBX2- and TBX3-mediated regulation of senescence factor, CDKN1A [51]–[53]. Interestingly, we found that CAV-1 is connected to TBX2 via ARHGDIA and TMED2. Both ARHGDIA and TMED2 are directly linked to the master transcriptional regulator of the anti-oxidant response, nuclear factor erythroid 2-related factor 2 (NRF2), i.e. NFE2L2, which was predicted to be a bicluster regulator along with CDKN1A (Table 3; also a regulator in one of ten FABIA-generated biclusters, Figure S4).

In the Discussion section, the fifth paragraph is corrected to:

“Thus, the activities of both T-box proteins and the CDKN2A products converge on the p53 pathway. Our findings (Figure S1C) underscore the roles of T-box proteins and CDKN2A in the etiology of COPD and indicate that the expressions of these genes are linked in the human lung epithelium. Of note are the changes in expressions of TBX (-2, -3) and CDKN2A occur in opposite directions in cancer. T-box genes/proteins such as TBX2 and TBX3 are overexpressed in several neoplasms, including melanomas, breast, and pancreatic cancer [53], [80], [81]. On the other hand, because of its effect on MDM2, the CDKN2A product, ARF, acts as a tumor suppressor; consequently its loss is associated with neoplasms [82]. The other CDKN2A product, p16INK4A, also suppresses tumors, and is itself suppressed in neoplasms [77]. Secondly, in COPD, we show that expressions of TBX (-3, -5) are suppressed and expression of CDKN2A increases (Figures 2 and 3A and Figures S1B, and S1C). This is in distinct contrast to prior observations made in cancer. TBX2 belongs to the TBX2 sub-family of TBX transcription factors that include TBX3, TBX4, and TBX5 genes [71]. We also show an association between expression levels of TBX3, TBX5, and CDKN2A, an indication they are statistically associated (Figure S1C). TBX3 down-regulates the expression of the CDKN2A product, ARF [32]. Similarly, via the stress-activated p38 MAP kinase, the activated TBX2 localizes in the nucleus and represses the closely related CDKN1A (p21) promoter [51]; CDKN1A is an inhibitor of DNA repair [83],[84]. In Figures S2A and S2B, we show that both TBX2 and CDKN2A are highly connected in the human lung epithelium transcriptional regulatory network. We also show that many of the genes differentially expressed in the COPD lungs are statistically dependent on the expression of TBX2 and CDKN2A.”

In the Materials and Methods section, the following subsection should be removed:

**“Immunoblot Assay**. Immunoblots were performed using antibodies for TBX2, CDKN2A, CDKN1A, SIRT1, CAV1, HDAC2, and ACTIN-B (Santa Cruz Biotechnology, Santa Cruz, CA). ACTIN-B was used as a loading control. These immunoblots were performed using protocols as described previously [19].”

The journal editors and authors confirm that these corrections do not affect the conclusions reported in the article.

The following authors agree to this correction: Acquaah-Mensah GK, Biswal S, Vulimiri M, Malhotra D

No response was received from the following authors: McDermott JE
