# Supplementary figures and images for: Suppressed Expression of T-Box Transcription Factors Is Involved in Senescence in Chronic Obstructive Pulmonary Disease
Source: PLoS Comput Biol. 2012 Jul 19;8(7):e1002597. doi: 10.1371/journal.pcbi.1002597 (PMC3400575; doi:10.1371/journal.pcbi.1002597)

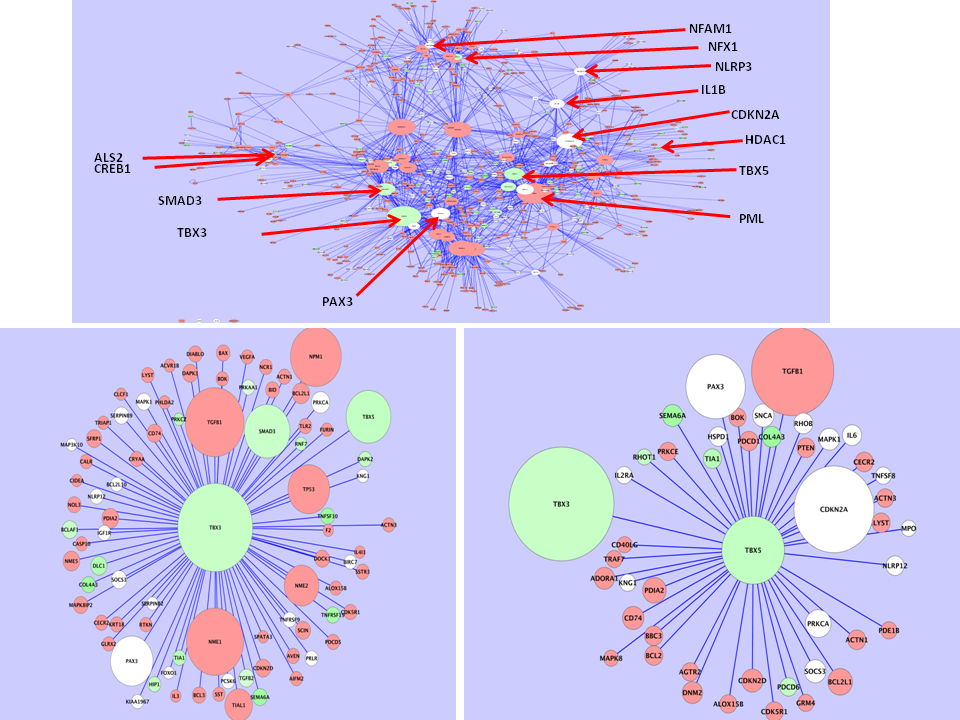

Supplement: Figure S1 — CLR-Generated transcriptional regulatory network of human lung epithelial cells. Following Robust Multi-Array Analysis of a compendium of 158 Affymetrix arrays, the Context Likelihood of Relatedness (CLR) algorithm was used to generate a transcriptional regulatory network (false discovery rate, 0.05). A) A synoptic view of the overall lung epithelial transcriptional regulatory network generated using Gene Ontology genes associated with apoptosis, response to inflammation, and response to oxidative stress. B) An up-close view of TBX3 and nodes directly connected to it in the network generated. C) An up-close view of TBX5 and nodes directly connected to it in the network generated. Larger-sized nodes represent hubs within the network, i.e. human lung epithelium cell genes more highly connected to other genes associated with apoptosis, response to inflammation, and response to oxidative stress. Olive-green nodes represent genes whose median probe set expressions are suppressed in COPD. White nodes represent genes whose median probe set expressions are elevated in COPD. (TIF) [file pcbi.1002597.s001.tif]

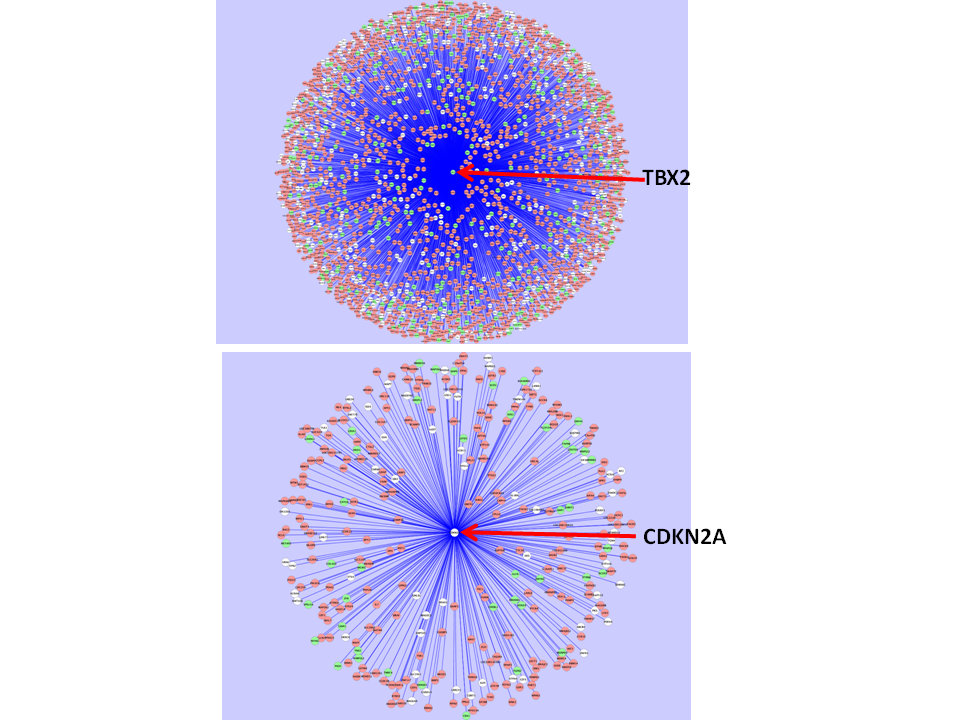

Supplement: Figure S2 — The states of a large cross-section of human epithelial cell genes differentially expressed in COPD depend on the states of (A) TBX2 and (B) CDKN2A. Following Robust Multi-Array Analysis of a compendium of 109 Affymetrix arrays on the U133A platform, the Context Likelihood of Relatedness (CLR) algorithm was used to generate a transcriptional regulatory network involving all available probe sets (at a CLR likelihood estimate cut-off of 2.5). Olive-green nodes represent genes whose median probe set expressions are suppressed in COPD. White nodes represent genes whose median probe set expressions are elevated in COPD. TBX2 gene expression is suppressed while CDKN2A gene expression is elevated in COPD. (TIF) [file pcbi.1002597.s002.tif]

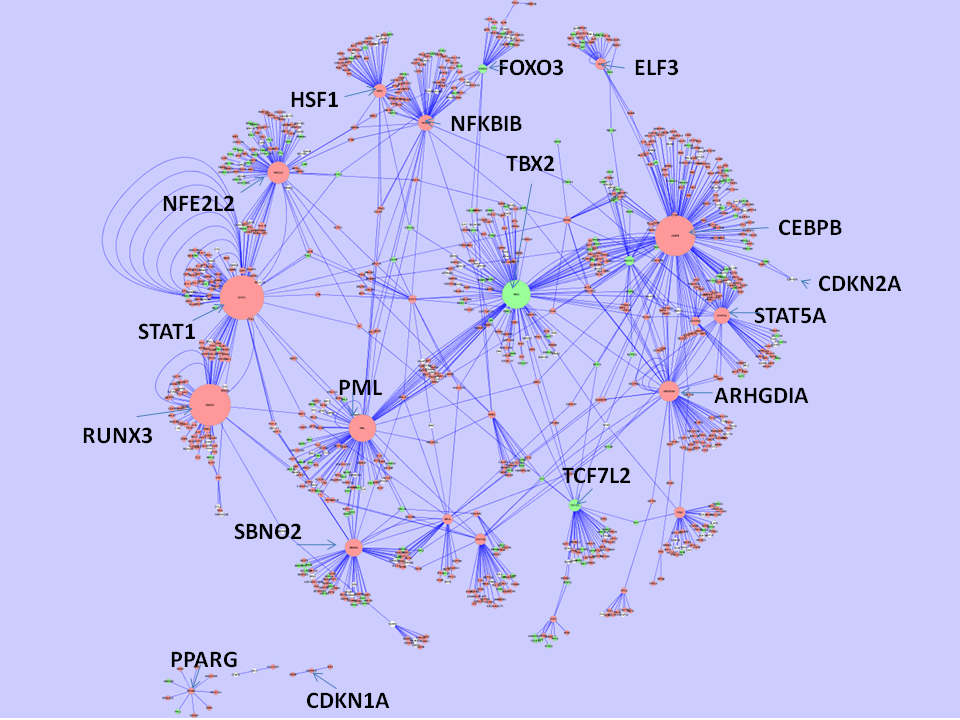

Supplement: Figure S3 — TBX2 is statistically associated with a large cross-section of genes differentially expressed in the COPD lung. Following Robust Multi-Array Analysis, ten biclusters were identified using FABIA from a compendium of 109 Affymetrix human lung epithelial cell microarrays. Focusing only on genes present in each cluster, the Context Likelihood of Relatedness (CLR) algorithm was used to generate Transcriptional Regulatory Networks (false discovery rate, 0.05). The ten networks are merged in this figure. Olive-green nodes represent genes whose median probe set expressions are suppressed in COPD. White nodes represent genes whose median probe set expressions are elevated in COPD. TBX2, the olive node in the center, is either directly or indirectly (by way of one or two intervening nodes) linked with a significant cross-section of genes differentially expressed in the COPD lung. (TIF) [file pcbi.1002597.s003.tif]

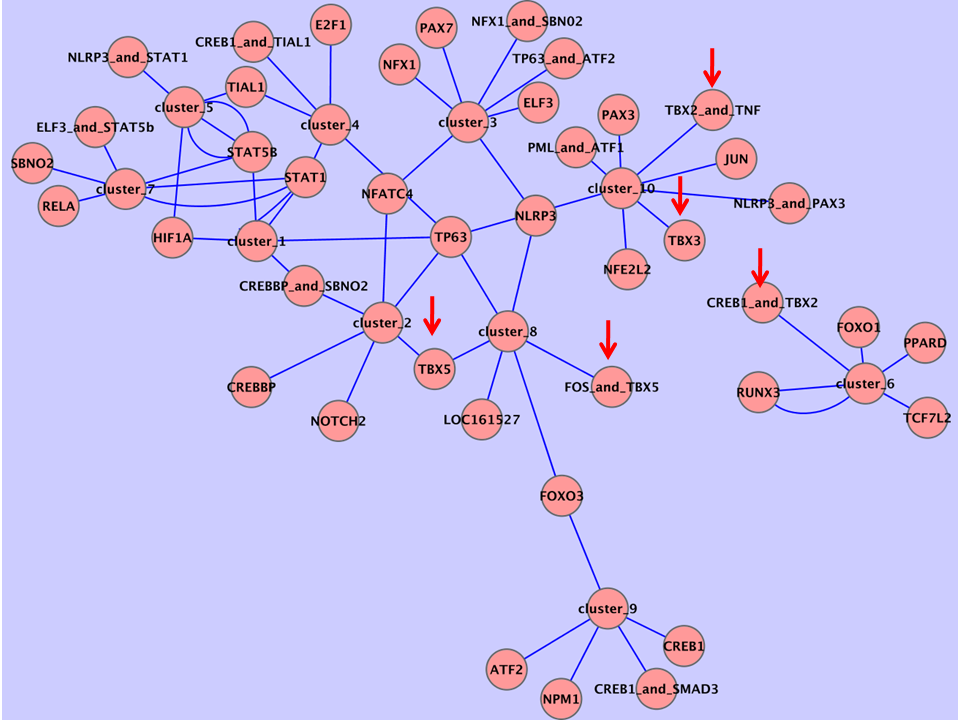

Supplement: Figure S4 — TBX gene products (captured by red arrows in figure) are predicted to be involved in the direct regulation of 40% of biclusters in the dataset. Following Robust Multi-Array Analysis, ten biclusters were identified using FABIA from a compendium of 109 Affymetrix human lung epithelial cell microarrays. Inferelator version 1.0 was used to infer a minimal set of regulators that explain the expression levels of each of 10 biclusters. TBX2 was predicted to be involved in the direct regulation of biclusters 6 and 10. TBX3 was predicted to be involved in the direct regulation of bicluster 10. TBX5 was predicted to be involved in the direct regulation of biclusters 2 and 8. (TIF) [file pcbi.1002597.s004.tif]
