# Supplementary material for: Suppressed Expression of T-Box Transcription Factors Is Involved in Senescence in Chronic Obstructive Pulmonary Disease
Source: PLoS Comput Biol. 2012 Jul 19;8(7):e1002597. doi: 10.1371/journal.pcbi.1002597 (PMC3400575; doi:10.1371/journal.pcbi.1002597)
Supplement: Table S1 — Interacting nodes in CLR-generated network and their corresponding likelihood estimates. The data in this table correspond to Figure S1. (DOC) [file pcbi.1002597.s005.doc]

**Table S1. Interacting Nodes in CLR-Generated Network and Their Corresponding Likelihood Estimates (See Figure S1)**

Note: Certain genes are represented by multiple probe sets and so occur more than once in the network.

| **GENE** | **CLR_LIKELIHOOD_ESTIMATE_(ABSOLUTE)** | **GENE** |  | **GENE** | **CLR_LIKELIHOOD_ESTIMATE_(ABSOLUTE)** | **GENE** |  | **GENE** | **CLR_LIKELIHOOD_ESTIMATE_(ABSOLUTE)** | **GENE** |
| --- | --- | --- | --- | --- | --- | --- | --- | --- | --- | --- |
| AATF | 4 | FOXL2 |  | DFFA | 3.89 | ETS1 |  | NPM1 | 3 | TP53 |
| AATF | 3 | SMAD3 |  | DFFA | 3.75 | PML |  | NUDT15 | 2.88 | ATF1 |
| ABL1 | 3.54 | TP53 |  | DHCR24 | 3.51 | CEBPG |  | NUDT15 | 3 | CREB1 |
| ABL1 | 3.65 | NME1 |  | DHCR24 | 4.9 | HMOX1 |  | NUDT2 | 4.51 | TP63 |
| ACE2 | 3.83 | HIF1A |  | DHCR24 | 5.26 | FOXL2 |  | NUP62 | 3.98 | RELA |
| ACTN1 | 3 | ARHGDIA |  | DHCR24 | 3.53 | HTATIP2 |  | NUP62 | 3.62 | TP63 |
| ACTN1 | 3.73 | NME1 |  | DHCR24 | 4.1 | AATF |  | NUP62 | 3.81 | ERC1 |
| ACTN1 | 4.5 | TBX3 |  | DHCR24 | 3.51 | STAT1 |  | NUP62 | 3.54 | NME1 |
| ACTN1 | 3.53 | TBX5 |  | DHCR24 | 3.68 | SMAD3 |  | NUP62 | 4.23 | NPM1 |
| ACTN2 | 3.96 | PAX3 |  | DIABLO | 4 | SMAD3 |  | OPA1 | 3.48 | TIAL1 |
| ACTN2 | 4.63 | PML |  | DIABLO | 3.56 | FOXL2 |  | ORM1 | 2.82 | NFX1 |
| ACTN3 | 4 | TBX5 |  | DIABLO | 4.24 | HMOX1 |  | OXSR1 | 3.1 | FOS |
| ACTN3 | 4.48 | TBX3 |  | DIABLO | 4 | TBX3 |  | OXSR1 | 3.18 | NFE2L2 |
| ACTN3 | 4 | PAX3 |  | DIABLO | 3.92 | AATF |  | P2RX4 | 4.21 | TP63 |
| ACTN4 | 3.65 | TGFB1 |  | DLC1 | 3 | TP53 |  | P2RX7 | 3.96 | PML |
| ACVR1B | 4 | TBX3 |  | DLC1 | 3.9 | JMY |  | PARK7 | 4.72 | STAT3 |
| ACVR1B | 3.51 | NPM1 |  | DLC1 | 3.7 | PML |  | PARK7 | 4.75 | AP1GBP1 |
| ACVR1C | 4 | NOTCH2 |  | DLC1 | 3 | NPM1 |  | PARK7 | 4.17 | NFKBIB |
| ADAMTSL4 | 5.14 | NLRP3 |  | DLC1 | 4 | NME2 |  | PAWR | 3.53 | RUNX3 |
| ADAMTSL4 | 5 | CDKN2A |  | DLC1 | 4.03 | TP63 |  | PAX3 | 4 | PAX7 |
| ADORA1 | 4.06 | CDKN2A |  | DLC1 | 4 | NME1 |  | PAX3 | 3 | TBX3 |
| ADORA1 | 4.16 | TGFB1 |  | DLC1 | 3 | TBX3 |  | PCBP4 | 4.05 | E2F1 |
| ADORA1 | 3.52 | NFAM1 |  | DNAJA3 | 3.86 | FOXL2 |  | PCBP4 | 4.07 | PYCARD |
| ADORA1 | 4.11 | TIAL1 |  | DNAJA3 | 3.86 | SMAD3 |  | PCBP4 | 3.73 | RUNX3 |
| ADORA1 | 3.85 | TBX5 |  | DNAJA3 | 3.48 | HMOX1 |  | PCBP4 | 3.65 | PML |
| ADORA2A | 2.88 | HIF1A |  | DNAJA3 | 3.6 | AATF |  | PCSK6 | 3.94 | CDKN2A |
| ADORA2A | 4.83 | HTATIP2 |  | DNAJB13 | 3.49 | TP73 |  | PCSK6 | 3.64 | TBX3 |
| ADORA2A | 4.38 | PYCARD |  | DNAJB13 | 3.97 | NME2 |  | PCSK6 | 3.7 | TGFB1 |
| ADORA2A | 3.48 | RUNX3 |  | DNAJB13 | 3.93 | TP53 |  | PCSK6 | 5.41 | NEUROD1 |
| ADORA2A | 2.79 | NFRKB |  | DNAJB13 | 4 | NPM1 |  | PCSK9 | 6 | TP63 |
| ADORA3 | 3.45 | NFATC4 |  | DNAJB13 | 4.43 | NME1 |  | PCSK9 | 6 | IL1B |
| ADRA1A | 3.56 | RELA |  | DNAJB6 | 4.1 | BCL10 |  | PDCD1 | 4.57 | TGFB1 |
| AGER | 3.87 | TGFB1 |  | DNM1L | 3.56 | TP73 |  | PDCD1 | 3.58 | RUNX3 |
| AGT | 4.15 | PAX3 |  | DNM2 | 3.84 | RELA |  | PDCD1 | 4.67 | TBX5 |
| AGT | 3.59 | JMY |  | DNM2 | 3.8 | ARHGDIA |  | PDCD1 | 4.17 | CDKN2A |
| AGTR1 | 4 | RIPK2 |  | DNM2 | 5.33 | ERC1 |  | PDCD2 | 3.66 | NPM1 |
| AGTR1 | 4.87 | HMOX1 |  | DNM2 | 4.24 | PML |  | PDCD5 | 3.93 | HMOX1 |
| AGTR1 | 2.73 | NFRKB |  | DNM2 | 3.49 | HTATIP2 |  | PDCD5 | 3.79 | TBX3 |
| AGTR2 | 4.96 | PML |  | DNM2 | 3.83 | TBX5 |  | PDCD5 | 3.8 | NME1 |
| AGTR2 | 3.48 | NFKB1 |  | DOCK1 | 3.54 | STAT1 |  | PDCD5 | 3.94 | AATF |
| AGTR2 | 3.71 | TBX5 |  | DOCK1 | 3.71 | TBX3 |  | PDCD6 | 5.38 | TCF7L2 |
| AIF1 | 2 | HMOX1 |  | DOCK1 | 4.04 | FOXL2 |  | PDCD6 | 3.71 | PML |
| AIF1 | 2 | RIPK2 |  | DOCK1 | 4.14 | PAX3 |  | PDCD6 | 3.88 | TBX5 |
| AIF1 | 2 | CEBPB |  | DOCK1 | 4.41 | HMOX1 |  | PDE1B | 4.13 | NLRP3 |
| AIF1 | 2 | ELF3 |  | DOCK1 | 3.63 | PAX7 |  | PDE1B | 4.41 | TBX5 |
| AIF1 | 2.83 | TGFB1 |  | DPF1 | 4.14 | ERC1 |  | PDIA2 | 4.08 | TBX3 |
| AIFM1 | 4.29 | AATF |  | DPF1 | 3.65 | PML |  | PDIA2 | 4.38 | CDKN2A |
| AIFM1 | 3 | SMAD3 |  | DPF1 | 3.59 | TIAL1 |  | PDIA2 | 4.42 | PML |
| AIFM1 | 4.54 | HMOX1 |  | DPF2 | 3.49 | HTATIP2 |  | PDIA2 | 4.15 | TGFB1 |
| AIFM1 | 4.18 | FOXL2 |  | DUSP22 | 3.5 | TGFB1 |  | PDIA2 | 3.52 | PAX3 |
| AIFM2 | 3.54 | NME2 |  | DUSP22 | 3.54 | NME1 |  | PDIA2 | 3.86 | RUNX3 |
| AIFM2 | 3.58 | TP53 |  | DYRK2 | 3.97 | PML |  | PDIA2 | 5 | TBX5 |
| AIFM2 | 3 | TBX3 |  | E2F1 | 4.82 | AHR |  | PDIA2 | 3.77 | ERC1 |
| AKT1 | 4 | ARHGDIA |  | E2F1 | 4 | PML |  | PEA15 | 3.66 | E2F1 |
| AKT1 | 3.79 | RELA |  | E2F1 | 4 | PPARD |  | PEA15 | 4 | PYCARD |
| AKT1S1 | 4 | TP53 |  | EIF5A | 3.55 | TP63 |  | PEA15 | 3 | ERC1 |
| AKT1S1 | 4 | SMAD3 |  | EIF5A | 4 | CDKN2A |  | PEA15 | 3.62 | RUNX3 |
| AKT1S1 | 5 | ARHGDIA |  | ELA2 | 3.31 | SBNO2 |  | PHB | 3.76 | NPM1 |
| AKT1S1 | 5 | RELA |  | ELF3 | 2 | HMOX1 |  | PHB | 3.91 | NME1 |
| ALOX15 | 4.43 | HIF1A |  | ELF3 | 2 | CEBPB |  | PHF17 | 3.83 | PPARD |
| ALOX15 | 3.66 | ABCF1 |  | ELF3 | 3 | NFRKB |  | PHF17 | 4 | IL1B |
| ALOX15 | 2 | ELF3 |  | EP300 | 5.18 | EP300 |  | PHF17 | 3 | PML |
| ALOX15B | 3.92 | TBX5 |  | EPAS1 | 3.1 | ATF2 |  | PHF17 | 3.6 | HMOX1 |
| ALOX15B | 4.11 | PAX3 |  | EPAS1 | 3.14 | AP1GBP1 |  | PHF17 | 3.95 | FOXL2 |
| ALOX15B | 3.57 | TBX3 |  | EPAS1 | 3 | CREBBP |  | PHF17 | 3.76 | SMAD3 |
| ALOX5AP | 2.84 | CEBPB |  | EPAS1 | 3.27 | JUN |  | PHF17 | 3.92 | CEBPG |
| ALOX5AP | 3.25 | FOS |  | EPAS1 | 5.19 | STAT1 |  | PHLDA2 | 3.78 | NME1 |
| ALOX5AP | 2.93 | HMOX1 |  | EPHX2 | 3.23 | NFRKB |  | PHLDA2 | 3.85 | TBX3 |
| ALOX5AP | 3 | ELF3 |  | EPHX2 | 3.45 | ITCH |  | PIK3R2 | 5.52 | PML |
| ALS2 | 3 | RELA |  | EPHX2 | 3.41 | ABCF1 |  | PIK3R2 | 3.75 | RUNX3 |
| ALS2 | 3 | CREB1 |  | EPHX2 | 3 | NFX1 |  | PIK3R2 | 3.53 | CDKN2A |
| ALS2 | 3.17 | ATF2 |  | ERC1 | 3 | HTATIP2 |  | PLA2G4C | 2.82 | NLRP3 |
| ALS2 | 3 | CREBBP |  | ERC1 | 3 | TIAL1 |  | PLAGL1 | 4 | TCF7L2 |
| ALS2 | 3 | ATF1 |  | ERC1 | 3 | RELA |  | PLAGL1 | 4 | PYCARD |
| ANGPTL4 | 3 | NLRP3 |  | ERC1 | 3 | PML |  | PLAGL1 | 4 | TP63 |
| ANGPTL4 | 4 | IL1B |  | ERCC2 | 3.1 | RELA |  | PLAGL1 | 4 | HTATIP2 |
| ANGPTL4 | 4 | TP63 |  | ERCC2 | 3 | HSF1 |  | PLAGL1 | 3.82 | EP300 |
| ANXA1 | 5 | CDKN2A |  | ERCC2 | 4.17 | PYCARD |  | PLAGL1 | 4 | ERC1 |
| ANXA1 | 3.6 | PPARD |  | ERCC2 | 2.94 | STAT3 |  | PMAIP1 | 3.64 | ERC1 |
| ANXA1 | 3.98 | NFE2L1 |  | ERCC2 | 3.68 | AP1GBP1 |  | PMAIP1 | 3.62 | HTATIP2 |
| ANXA1 | 3.3 | ITCH |  | ERCC2 | 4 | PML |  | PML | 4 | RELA |
| ANXA1 | 4.3 | NFRKB |  | ERCC2 | 4.85 | TCF7L2 |  | PML | 4 | CDKN2A |
| ANXA1 | 4.01 | E2F1 |  | ERN2 | 3 | TP73 |  | PPP1R13B | 3.57 | AATF |
| ANXA1 | 3.93 | SBNO2 |  | ERN2 | 3.71 | NME1 |  | PPP1R13B | 3.81 | SMAD3 |
| ANXA1 | 3.67 | PML |  | ERN2 | 3 | NPM1 |  | PPP1R13B | 4.21 | FOXL2 |
| ANXA1 | 3.92 | HTATIP2 |  | ESPL1 | 4 | AHR |  | PPP1R13B | 4.06 | TP73 |
| ANXA1 | 3.57 | NOTCH2 |  | ESPL1 | 3 | NPM1 |  | PPP1R13B | 3.83 | HMOX1 |
| ANXA4 | 4 | NOTCH2 |  | ESPL1 | 3 | NME1 |  | PPP1R15A | 3 | E2F1 |
| ANXA4 | 7 | CDKN2A |  | ETS1 | 3 | CDKN2A |  | PPP1R15B | 3.14 | ATF1 |
| ANXA4 | 3.97 | NPM1 |  | F11R | 3 | HMOX1 |  | PPP1R15B | 3.65 | CREB1 |
| ANXA4 | 3.5 | TP63 |  | F11R | 3 | NFX1 |  | PPP1R15B | 3.27 | AP1GBP1 |
| ANXA4 | 3.9 | PML |  | F11R | 3 | RIPK2 |  | PPP1R15B | 4.26 | RELA |
| AOC3 | 2.78 | ELF3 |  | F11R | 2.77 | NFE2L1 |  | PPP1R15B | 3 | CREBBP |
| AP1GBP1 | 3 | CREBBP |  | F11R | 3.67 | TGFB1 |  | PPP2R1A | 4.51 | ARHGDIA |
| APAF1 | 4.15 | TIAL1 |  | F2 | 4 | TBX3 |  | PPP2R1A | 4.17 | SMAD3 |
| APCS | 3.2 | NFRKB |  | F2R | 3.29 | TGFB1 |  | PPP3CC | 4 | STAT1 |
| APCS | 3.17 | TNF |  | F2R | 4.03 | HIF1A |  | PPP3CC | 4.14 | TP63 |
| API5 | 4 | BCL10 |  | F2R | 3 | TP53 |  | PPP3CC | 3.76 | TIAL1 |
| API5 | 3.77 | PML |  | F2R | 3 | NPM1 |  | PRDX2 | 5.39 | AATF |
| API5 | 5.23 | TP63 |  | F2R | 3.85 | NME2 |  | PRDX2 | 3.89 | JMY |
| API5 | 3.69 | CDKN2A |  | F2R | 4.07 | NME1 |  | PRDX2 | 2.86 | STAT5B |
| API5 | 4 | IL1B |  | F2R | 3.28 | ABCF1 |  | PRDX2 | 2.89 | CREBBP |
| APOE | 3.67 | PAX3 |  | F2R | 3.24 | NFX1 |  | PRDX2 | 5.95 | HMOX1 |
| APOE | 3.28 | ATF1 |  | FADD | 3.81 | SMAD3 |  | PRDX2 | 6.19 | FOXL2 |
| APOE | 2.85 | CREBBP |  | FAF1 | 3 | ARHGDIA |  | PRDX2 | 5 | SMAD3 |
| APOE | 2.86 | TGFB1 |  | FAF1 | 4.3 | PML |  | PRDX2 | 5 | HTATIP2 |
| APOE | 4 | NFATC4 |  | FAF1 | 3.88 | RELA |  | PRDX2 | 2.75 | AP1GBP1 |
| APOE | 3.7 | TNF |  | FAF1 | 3.84 | ERC1 |  | PRDX5 | 3.14 | TGFB1 |
| APOH | 4.39 | HMOX1 |  | FAF1 | 3.97 | E2F1 |  | PRDX5 | 3 | HIF1A |
| APOH | 4 | SMAD3 |  | FAIM3 | 4 | HTATIP2 |  | PRDX5 | 3 | ABCF1 |
| APOH | 3.99 | AATF |  | FAIM3 | 6.13 | PML |  | PRDX6 | 4.49 | AP1GBP1 |
| APOH | 4.43 | FOXL2 |  | FAIM3 | 4.21 | PPARD |  | PRF1 | 5 | RUNX3 |
| APOL2 | 4.64 | NFATC4 |  | FANCG | 3.64 | NME1 |  | PRF1 | 3.83 | TNF |
| APOL2 | 4.92 | TGFB1 |  | FANCG | 3.51 | TIAL1 |  | PRKAA1 | 3.84 | TIAL1 |
| ARHGDIA | 4 | ARHGDIA |  | FAS | 3.8 | RELA |  | PRKAA1 | 3.68 | EP300 |
| ARHGDIA | 5 | RELA |  | FASLG | 3.74 | CDKN2A |  | PRKAA1 | 4 | TBX3 |
| ARHGDIA | 4 | PML |  | FEM1B | 3.89 | NOTCH2 |  | PRKAA1 | 3.52 | JMY |
| ARHGDIA | 4 | ERC1 |  | FOXL2 | 3 | HTATIP2 |  | PRKCA | 3.74 | NME1 |
| ARHGEF6 | 3.51 | NME2 |  | FOXL2 | 3 | STAT1 |  | PRKCA | 3.76 | TBX3 |
| ARHGEF6 | 3 | NPM1 |  | FOXL2 | 5 | HMOX1 |  | PRKCA | 3.85 | CDKN2A |
| ARHGEF6 | 4.02 | NME1 |  | FOXO1 | 3.56 | TBX3 |  | PRKCA | 3.99 | TBX5 |
| ATF1 | 4 | CREB1 |  | FOXO3 | 3 | SMAD3 |  | PRKCA | 3.74 | HTATIP2 |
| ATF2 | 3 | ATF1 |  | FOXO3 | 4.1 | PML |  | PRKCA | 3.72 | NME2 |
| ATF2 | 5 | STAT1 |  | FOXO3 | 3.49 | AATF |  | PRKCA | 4.34 | TGFB1 |
| ATF2 | 4 | CREB1 |  | FPR2 | 2 | ELF3 |  | PRKCA | 5.23 | FOXL2 |
| ATP7A | 3.6 | JMY |  | FURIN | 3.65 | TBX3 |  | PRKCA | 4 | SMAD3 |
| AVEN | 4.78 | HMOX1 |  | FURIN | 3.86 | PPARD |  | PRKCA | 4.48 | AATF |
| AVEN | 4.43 | FOXL2 |  | FXR1 | 5.05 | EP300 |  | PRKCA | 4.95 | HMOX1 |
| AVEN | 4.25 | AATF |  | GAB1 | 4.18 | NFKBIB |  | PRKCE | 4.74 | CDKN2A |
| AVEN | 3 | TBX3 |  | GAS2 | 3.51 | TGFB1 |  | PRKCE | 4.4 | TBX5 |
| BAD | 3.97 | NOTCH2 |  | GCLC | 3.59 | NOTCH2 |  | PRKCE | 5.35 | TGFB1 |
| BAD | 3.67 | ARHGDIA |  | GCLC | 6 | CDKN2A |  | PRKCZ | 3 | TBX3 |
| BAD | 3 | ERC1 |  | GCLC | 4 | PML |  | PRLR | 4.5 | PAX3 |
| BAG4 | 3.69 | TIAL1 |  | GHRL | 4.39 | NLRP3 |  | PRLR | 3.71 | PAX7 |
| BAK1 | 4.07 | PYCARD |  | GHRL | 3.18 | NFAM1 |  | PRLR | 3.5 | EP300 |
| BAK1 | 4 | PML |  | GHRL | 3.43 | TGFB1 |  | PRLR | 4.2 | TBX3 |
| BAX | 3.79 | TBX3 |  | GLO1 | 3 | CDKN2A |  | PRNP | 3.12 | STAT5B |
| BAX | 4 | SMAD3 |  | GLRX2 | 3.67 | TBX3 |  | PRNP | 3.37 | FOS |
| BAX | 4 | NME1 |  | GLRX2 | 3 | NPM1 |  | PRODH | 4 | TP73 |
| BAX | 4 | TP53 |  | GLRX2 | 3.93 | NME1 |  | PSEN1 | 4.06 | TCF7L2 |
| BAX | 3.52 | NME2 |  | GML | 4.37 | PML |  | PSEN1 | 3 | STAT1 |
| BBC3 | 4.08 | TBX5 |  | GML | 4.32 | PAX3 |  | PSEN2 | 3 | HTATIP2 |
| BBC3 | 4.04 | TGFB1 |  | GPR68 | 3.17 | NFATC4 |  | PTAFR | 3.17 | NFE2L1 |
| BBC3 | 3.6 | CDKN2A |  | GPX1 | 3.46 | AP1GBP1 |  | PTAFR | 3.22 | NFAM1 |
| BBC3 | 4.31 | RUNX3 |  | GPX1 | 4.07 | HTATIP2 |  | PTAFR | 5.58 | NFATC4 |
| BCL2 | 4 | CDKN2A |  | GPX1 | 3.68 | STAT3 |  | PTEN | 3.48 | NME1 |
| BCL2 | 4 | TGFB1 |  | GPX3 | 3.69 | STAT3 |  | PTEN | 4 | TIAL1 |
| BCL2 | 4 | TBX5 |  | GRIK2 | 4.75 | PAX3 |  | PTEN | 4.22 | TBX5 |
| BCL2 | 4 | RUNX3 |  | GRM4 | 3.74 | TIAL1 |  | PTEN | 3.89 | TCF7L2 |
| BCL2A1 | 6 | CDKN2A |  | GRM4 | 3.59 | TBX5 |  | PTGS2 | 4 | HMOX1 |
| BCL2A1 | 4 | NOTCH2 |  | GSK3B | 4.05 | TP63 |  | PTGS2 | 2 | IL1B |
| BCL2L1 | 4 | SMAD3 |  | GSK3B | 4 | IL1B |  | PTGS2 | 4 | RIPK2 |
| BCL2L1 | 4 | ARHGDIA |  | GSTA1 | 2.81 | MAF |  | PTK2B | 4.09 | PML |
| BCL2L1 | 3.79 | TBX5 |  | GSTA1 | 3.09 | NFKBIB |  | PTK2B | 5 | IL1B |
| BCL2L1 | 4 | TBX3 |  | GSTA1 | 3.69 | NFE2L2 |  | PTK2B | 4.12 | ARHGDIA |
| BCL2L1 | 4 | ERC1 |  | GSTA1 | 3.03 | CREBBP |  | PTK2B | 7 | PPARD |
| BCL2L1 | 4.12 | RELA |  | GSTA1 | 3.75 | FOS |  | PTK2B | 5.66 | TP63 |
| BCL2L1 | 4.42 | TGFB1 |  | GSTT2 | 3.81 | HSF1 |  | PTX3 | 4 | ITCH |
| BCL2L1 | 3.54 | CDKN2A |  | GULP1 | 6 | CDKN2A |  | PTX3 | 4 | NFE2L1 |
| BCL2L1 | 5.78 | PML |  | GULP1 | 3.5 | PML |  | PTX3 | 3.22 | NFRKB |
| BCL2L10 | 4.28 | TBX3 |  | GULP1 | 3.47 | PAX3 |  | PTX3 | 4 | ELF3 |
| BCL2L10 | 3.47 | ERC1 |  | GULP1 | 3.82 | TCF7L2 |  | PXK | 3.2 | NFRKB |
| BCL2L10 | 3.48 | TGFB1 |  | GULP1 | 5 | NOTCH2 |  | PXK | 3.33 | NFATC3 |
| BCL2L11 | 3.48 | ERC1 |  | GZMA | 7 | RUNX3 |  | PYCARD | 3 | PML |
| BCL2L11 | 4.28 | PML |  | GZMA | 5.4 | STAT1 |  | RAC1 | 3.45 | SIGIRR |
| BCL2L11 | 5.59 | TCF7L2 |  | GZMB | 5.74 | STAT1 |  | RAC1 | 3.22 | HMOX1 |
| BCL2L11 | 4.21 | EP300 |  | GZMB | 7 | RUNX3 |  | RAF1 | 3.85 | NOTCH2 |
| BCL2L13 | 3.83 | ARHGDIA |  | H6PD | 3.48 | CREB1 |  | RAF1 | 3 | HTATIP2 |
| BCL2L13 | 4.12 | PML |  | H6PD | 3 | STAT3 |  | RAF1 | 3.82 | PYCARD |
| BCL2L2 | 3.94 | TIAL1 |  | H6PD | 2.74 | ATF2 |  | RAF1 | 3.47 | EP300 |
| BCL2L2 | 4.96 | JMY |  | H6PD | 3.53 | MAF |  | RAF1 | 4.04 | PML |
| BCL3 | 3.8 | TBX3 |  | HBXIP | 3.47 | CDKN2A |  | RAF1 | 3.79 | ERC1 |
| BCL3 | 4.03 | TGFB1 |  | HBXIP | 4 | NOTCH2 |  | RASA1 | 5 | HTATIP2 |
| BCL3 | 4.05 | HMOX1 |  | HDAC1 | 4 | CDKN2A |  | RASA1 | 4 | ERC1 |
| BCL3 | 3.49 | FOXL2 |  | HDAC1 | 4 | TP63 |  | RELA | 2 | ATF1 |
| BCL3 | 3.58 | AATF |  | HDAC3 | 4 | CDKN2A |  | RELA | 3 | TP53 |
| BCL6 | 3 | NME1 |  | HDAC3 | 3 | NOTCH2 |  | RELA | 3 | STAT5B |
| BCL6 | 3.5 | TP73 |  | HDAC4 | 3.84 | NFRKB |  | RELA | 3 | AP1GBP1 |
| BCL6 | 3 | NPM1 |  | HDAC4 | 3.73 | NFATC4 |  | RELA | 5 | HSF1 |
| BCL6 | 3.49 | TP53 |  | HDAC5 | 2.78 | HIF1A |  | RHOB | 4.72 | ERC1 |
| BCLAF1 | 3.91 | STAT1 |  | HDAC5 | 4.01 | TNF |  | RHOB | 6 | STAT1 |
| BCLAF1 | 3.78 | RELA |  | HDAC5 | 3.05 | NFRKB |  | RHOB | 3.58 | TGFB1 |
| BCLAF1 | 5.69 | NPM1 |  | HDAC5 | 2.99 | SIGIRR |  | RHOB | 4.75 | TBX5 |
| BCLAF1 | 4.32 | TBX3 |  | HDAC7 | 3.73 | TGFB1 |  | RHOB | 3.85 | NOTCH2 |
| BCLAF1 | 3.54 | NEUROD1 |  | HDAC7 | 3.81 | SBNO2 |  | RHOT1 | 3.8 | TBX5 |
| BCLAF1 | 3.81 | JMY |  | HDAC7 | 4.39 | NFX1 |  | RNF7 | 3 | TBX3 |
| BCLAF1 | 3 | CDKN2A |  | HDAC9 | 3.32 | HMOX1 |  | RNF7 | 4 | SMAD3 |
| BDKRB2 | 2.83 | SIGIRR |  | HDAC9 | 3.23 | RIPK2 |  | RPS3 | 3.53 | NPM1 |
| BDKRB2 | 2 | ELF3 |  | HIF1A | 3 | RIPK2 |  | RPS3 | 3.49 | TP53 |
| BDKRB2 | 2.96 | HMOX1 |  | HIF1A | 3 | ABCF1 |  | RPS3 | 3.57 | NME1 |
| BDKRB2 | 2.74 | CEBPB |  | HIF1A | 3 | ITCH |  | RPS3A | 4.67 | NPM1 |
| BECN1 | 4.3 | PYCARD |  | HIF1A | 3 | TGFB1 |  | RRAGC | 4 | PYCARD |
| BECN1 | 3.53 | PML |  | HIP1 | 3 | TP53 |  | RRAGC | 4 | PML |
| BECN1 | 4.38 | TCF7L2 |  | HIP1 | 3 | NPM1 |  | RRAGC | 3.7 | ERC1 |
| BECN1 | 4 | HTATIP2 |  | HIP1 | 3 | TBX3 |  | RRAGC | 3.8 | HTATIP2 |
| BECN1 | 4.22 | NPM1 |  | HIP1 | 3 | NME1 |  | RTKN | 3 | TBX3 |
| BID | 3.72 | TP53 |  | HIPK2 | 4 | PML |  | RTKN | 3.7 | TP63 |
| BID | 4.04 | NME1 |  | HIPK2 | 4 | STAT1 |  | RTKN | 4.06 | PPARD |
| BID | 3 | TBX3 |  | HIPK2 | 3.83 | TCF7L2 |  | RYR2 | 5.59 | PAX3 |
| BID | 3 | NPM1 |  | HIPK2 | 4 | PYCARD |  | RYR2 | 3.9 | PAX7 |
| BID | 3.73 | NME2 |  | HIPK2 | 3.96 | JMY |  | S100A8 | 2.88 | IL1B |
| BIRC3 | 3.7 | HMOX1 |  | HIPK2 | 4.32 | PAX3 |  | S100A8 | 3.18 | HMOX1 |
| BIRC3 | 4.91 | RUNX3 |  | HIPK2 | 4 | HTATIP2 |  | S1PR3 | 3.87 | HIF1A |
| BIRC3 | 5.97 | STAT1 |  | HIPK3 | 4.14 | NEUROD1 |  | S1PR3 | 3.14 | HMOX1 |
| BIRC3 | 4.11 | FOXL2 |  | HMGB1 | 4 | CDKN2A |  | S1PR3 | 3.39 | TGFB1 |
| BIRC5 | 3.49 | TP53 |  | HMOX1 | 4 | NFATC3 |  | S1PR3 | 3 | NFX1 |
| BIRC5 | 3.61 | PML |  | HMOX1 | 4 | AATF |  | S1PR3 | 3.73 | ITCH |
| BIRC5 | 3 | NPM1 |  | HMOX1 | 5 | RIPK2 |  | S1PR3 | 3.65 | RIPK2 |
| BIRC5 | 4 | NME1 |  | HMOX1 | 3 | HTATIP2 |  | S1PR3 | 3 | ELF3 |
| BIRC5 | 4 | AHR |  | HRH1 | 3.12 | NFX1 |  | S1PR3 | 3.64 | ABCF1 |
| BIRC7 | 3.63 | TBX3 |  | HRH1 | 4.03 | HIF1A |  | SAA1 | 3.49 | NFX1 |
| BIRC7 | 3.48 | PAX3 |  | HRH1 | 3.48 | ABCF1 |  | SAA1 | 3 | HMOX1 |
| BIRC7 | 4.5 | JMY |  | HRH1 | 3 | ELF3 |  | SBNO2 | 7 | RELA |
| BLNK | 3.35 | TGFB1 |  | HRH1 | 3.23 | ITCH |  | SBNO2 | 2 | NFRKB |
| BLNK | 3.17 | FOS |  | HRK | 3 | STAT1 |  | SBNO2 | 3 | SBNO2 |
| BNIP1 | 3.5 | TP63 |  | HSF1 | 2 | CREB1 |  | SCG2 | 3.48 | STAT1 |
| BNIPL | 3.8 | NME1 |  | HSP90B1 | 3.54 | IL1B |  | SCIN | 3.71 | SMAD3 |
| BOK | 3.72 | TBX5 |  | HSP90B1 | 3.84 | TP63 |  | SCIN | 4 | TBX3 |
| BOK | 3 | ARHGDIA |  | HSPA1A | 4.4 | NPM1 |  | SCIN | 3.53 | TP53 |
| BOK | 3.69 | TBX3 |  | HSPA5 | 3.67 | JMY |  | SCIN | 3.72 | NPM1 |
| BRAF | 6.7 | JMY |  | HSPA5 | 3.84 | ERC1 |  | SCIN | 3.5 | NME1 |
| BRCA1 | 4.4 | PML |  | HSPA9 | 3.54 | CEBPG |  | SCIN | 3.89 | TGFB1 |
| BRCA1 | 3.53 | TIAL1 |  | HSPD1 | 4.33 | TBX5 |  | SCIN | 3.5 | NME2 |
| BRE | 4 | HTATIP2 |  | HSPD1 | 3.88 | TCF7L2 |  | SCYE1 | 3.13 | NFAM1 |
| BRE | 4 | SMAD3 |  | HSPD1 | 3.58 | SMAD3 |  | SELS | 3.53 | HTATIP2 |
| BRE | 4 | HMOX1 |  | HSPD1 | 4.47 | PAX3 |  | SELS | 4.62 | NLRP3 |
| BRE | 4.95 | NEUROD1 |  | HSPE1 | 4.93 | SMAD3 |  | SELS | 3.62 | PML |
| BRE | 4 | AATF |  | HTATIP2 | 3 | PYCARD |  | SEMA4D | 5.13 | NOTCH2 |
| BRE | 5 | FOXL2 |  | IDH1 | 3.02 | STAT3 |  | SEMA6A | 4 | TBX3 |
| BTG1 | 3 | CDKN2A |  | IFI16 | 4 | HMOX1 |  | SEMA6A | 4 | PAX3 |
| C11ORF17 | 4 | SMAD3 |  | IFI16 | 4 | SMAD3 |  | SEMA6A | 4.01 | CDKN2A |
| C16ORF5 | 4 | RUNX3 |  | IFI16 | 4 | FOXL2 |  | SEMA6A | 4.01 | TGFB1 |
| C16ORF5 | 4 | E2F1 |  | IFI16 | 3 | AATF |  | SEMA6A | 4.04 | TBX5 |
| C16ORF5 | 3.56 | ERC1 |  | IFI30 | 3.66 | SMAD3 |  | SEMA6A | 3.82 | TCF7L2 |
| C16ORF5 | 4 | PML |  | IFI6 | 3.67 | HTATIP2 |  | SERPINB2 | 4.18 | TBX3 |
| C16ORF5 | 4 | PYCARD |  | IFNB1 | 3.9 | PAX3 |  | SERPINB2 | 3 | SMAD3 |
| C3AR1 | 2.77 | CEBPB |  | IFNG | 4.36 | TCF7L2 |  | SERPINB9 | 3.49 | TIAL1 |
| C3AR1 | 2.82 | ELF3 |  | IFNG | 3.48 | RUNX3 |  | SERPINB9 | 3.65 | ERC1 |
| C5 | 4 | RIPK2 |  | IFNG | 3.62 | PML |  | SERPINB9 | 3.85 | PML |
| C5 | 4.86 | HMOX1 |  | IFT57 | 3 | TP53 |  | SERPINB9 | 4.45 | RUNX3 |
| CADM1 | 4 | CDKN2A |  | IFT57 | 4 | NPM1 |  | SERPINB9 | 3.66 | NME1 |
| CADM1 | 4 | AATF |  | IFT57 | 3 | NME1 |  | SERPINB9 | 3 | TBX3 |
| CADM1 | 3 | SMAD3 |  | IFT57 | 3.63 | AHR |  | SFN | 4 | TP73 |
| CADM1 | 4 | HMOX1 |  | IFT57 | 3 | NME2 |  | SFN | 4.09 | TP63 |
| CADM1 | 5 | FOXL2 |  | IGF1 | 3.95 | TNF |  | SFN | 4 | E2F1 |
| CADM1 | 3.58 | PML |  | IGF1R | 3.97 | SMAD3 |  | SFRP1 | 3.53 | PML |
| CALR | 4 | PML |  | IGF1R | 4.2 | TBX3 |  | SFRP1 | 4 | NME2 |
| CALR | 4 | ARHGDIA |  | IGF1R | 5 | STAT1 |  | SFRP1 | 4 | TP53 |
| CALR | 3.91 | TBX3 |  | IGF1R | 3.77 | TCF7L2 |  | SFRP1 | 4 | NME1 |
| CARD8 | 3.75 | FOXL2 |  | IGF2 | 3.19 | NFAM1 |  | SFRP1 | 4 | TBX3 |
| CARD8 | 3.69 | HMOX1 |  | IGFBP3 | 3 | SMAD3 |  | SFRP1 | 4 | NPM1 |
| CARD8 | 3.5 | NOTCH2 |  | IGFBP3 | 3.93 | FOXL2 |  | SFRP1 | 3 | ARHGDIA |
| CARD8 | 3.7 | AATF |  | IGFBP3 | 3.57 | AATF |  | SGPL1 | 4.25 | JMY |
| CARD8 | 3.65 | STAT1 |  | IGFBP3 | 3.81 | HMOX1 |  | SIAH1 | 3.88 | CEBPG |
| CASP10 | 3.73 | TGFB1 |  | IHPK2 | 3.64 | HMOX1 |  | SIAH1 | 4 | STAT1 |
| CASP10 | 3.55 | RELA |  | IHPK2 | 3.93 | FOXL2 |  | SIGIRR | 3 | NLRP3 |
| CASP10 | 4 | TBX3 |  | IKBKG | 3.76 | TGFB1 |  | SIGLEC1 | 2.87 | CEBPB |
| CASP2 | 4.23 | RELA |  | IL10 | 4.26 | NFRKB |  | SIGLEC1 | 3.96 | TNF |
| CASP2 | 5 | TIAL1 |  | IL10 | 3.85 | PAX3 |  | SIGLEC1 | 2.77 | RIPK2 |
| CASP2 | 3.72 | PAX7 |  | IL10 | 4 | TIAL1 |  | SIVA1 | 3.96 | PML |
| CASP2 | 4 | ARHGDIA |  | IL12A | 4.21 | E2F1 |  | SIVA1 | 4 | E2F1 |
| CASP2 | 4 | ERC1 |  | IL17C | 3.84 | NFATC4 |  | SIVA1 | 3.72 | ERC1 |
| CASP2 | 4.19 | PML |  | IL17C | 4.01 | TGFB1 |  | SMAD3 | 3 | RELA |
| CASP3 | 4.33 | E2F1 |  | IL18RAP | 2.82 | NLRP3 |  | SMAD3 | 3 | TP53 |
| CASP3 | 3.98 | NME1 |  | IL1A | 5 | IL1B |  | SNCA | 3.59 | TBX5 |
| CASP4 | 3.9 | HMOX1 |  | IL1A | 4.1 | PML |  | SNCA | 3.7 | HMOX1 |
| CASP4 | 4.02 | AATF |  | IL1A | 3.57 | NFRKB |  | SNCA | 5 | PML |
| CASP4 | 4.1 | JMY |  | IL1A | 3.84 | BCL10 |  | SNCA | 3.5 | IL1B |
| CASP4 | 3 | SMAD3 |  | IL1A | 4 | CDKN2A |  | SNCA | 4.52 | ETS1 |
| CASP4 | 4.03 | FOXL2 |  | IL1A | 6 | TP63 |  | SNCA | 3 | CDKN2A |
| CASP6 | 3.5 | PML |  | IL1A | 4 | NOTCH2 |  | SNCA | 3.64 | TGFB1 |
| CASP8 | 6 | STAT1 |  | IL1B | 5.47 | IL1B |  | SOCS2 | 3 | NME1 |
| CASP8 | 3.73 | TCF7L2 |  | IL1B | 4 | PPARD |  | SOCS2 | 3 | TP53 |
| CASP8AP2 | 3.52 | TIAL1 |  | IL1B | 5 | TP63 |  | SOCS2 | 3 | NME2 |
| CASP8AP2 | 4 | NOTCH2 |  | IL1B | 3 | SIGIRR |  | SOCS2 | 4 | NPM1 |
| CASP8AP2 | 4 | CDKN2A |  | IL1RAP | 3.17 | ITCH |  | SOCS3 | 4.1 | TBX3 |
| CASP9 | 5.04 | NLRP3 |  | IL1RAP | 4 | IL1B |  | SOCS3 | 3.79 | RUNX3 |
| CASP9 | 3.75 | TCF7L2 |  | IL1RAP | 3 | ELF3 |  | SOCS3 | 3.64 | JMY |
| CAT | 3.57 | STAT5A |  | IL1RAP | 3.77 | NFRKB |  | SOCS3 | 3.79 | TGFB1 |
| CAT | 3 | NFE2L2 |  | IL1RN | 2.83 | NLRP3 |  | SOCS3 | 3.84 | PPARD |
| CBX4 | 4.19 | TIAL1 |  | IL1RN | 4.12 | NFAM1 |  | SOCS3 | 3.5 | TNF |
| CBX4 | 3.61 | CEBPG |  | IL1RN | 2.77 | NFX1 |  | SOCS3 | 4 | TBX5 |
| CBX4 | 4.15 | RELA |  | IL1RN | 3 | IL1B |  | SON | 3.72 | NOTCH2 |
| CBX4 | 3.51 | SMAD3 |  | IL2 | 3.5 | CDKN2A |  | SON | 3.54 | HMOX1 |
| CBX4 | 6.35 | PML |  | IL20 | 3.17 | TNF |  | SON | 3 | SMAD3 |
| CBX4 | 3.55 | TP53 |  | IL20 | 3.4 | NFAM1 |  | SON | 3.82 | EP300 |
| CCL21 | 3.2 | NFAM1 |  | IL24 | 6 | TP63 |  | SON | 3.89 | BCL10 |
| CCL22 | 3.15 | NFAM1 |  | IL24 | 3.52 | PPARD |  | SON | 4.63 | JMY |
| CCL3 | 3 | IL1B |  | IL24 | 6 | IL1B |  | SON | 4.09 | FOXL2 |
| CCL4 | 2.76 | TNF |  | IL24 | 3.68 | NLRP3 |  | SORT1 | 4.73 | HTATIP2 |
| CCL4 | 3 | IL1B |  | IL2RA | 3.95 | TBX5 |  | SORT1 | 5 | SMAD3 |
| CCR1 | 2.74 | HMOX1 |  | IL2RA | 3.88 | TNF |  | SORT1 | 5 | FOXL2 |
| CCR1 | 2.78 | CEBPB |  | IL2RB | 6 | RUNX3 |  | SORT1 | 5 | AATF |
| CCR1 | 2 | ELF3 |  | IL3 | 3 | TBX3 |  | SORT1 | 5 | HMOX1 |
| CCR3 | 4.29 | NLRP3 |  | IL31RA | 3.61 | PML |  | SORT1 | 3.78 | PPARD |
| CCR4 | 3.25 | NLRP3 |  | IL31RA | 3.76 | IL1B |  | SPATA3 | 4.11 | TBX3 |
| CD14 | 4.35 | PAX3 |  | IL31RA | 3.96 | TP63 |  | SPATA4 | 3.96 | NME2 |
| CD2 | 5.86 | STAT1 |  | IL31RA | 3.74 | PAX7 |  | SPATA4 | 4.44 | NME1 |
| CD24 | 4 | ERC1 |  | IL4 | 4.87 | ERC1 |  | SPATA4 | 4 | TP53 |
| CD24 | 4 | PYCARD |  | IL4 | 4.22 | PAX7 |  | SPATA4 | 4 | NPM1 |
| CD24 | 3.51 | HTATIP2 |  | IL4 | 3 | PML |  | SPHK1 | 4 | CDKN2A |
| CD24 | 3 | AHR |  | IL4 | 4 | PAX3 |  | SPHK1 | 3.71 | ETS1 |
| CD28 | 4.84 | RUNX3 |  | IL4I1 | 3.68 | TBX3 |  | SPHK2 | 3.61 | TIAL1 |
| CD28 | 3.53 | ERC1 |  | IL5 | 3.12 | NLRP3 |  | SRA1 | 3.66 | TP53 |
| CD38 | 4 | CDKN2A |  | IL5 | 3.34 | ITCH |  | SRA1 | 3.63 | NME2 |
| CD3E | 6.26 | RUNX3 |  | IL6 | 3.29 | ELF3 |  | SRGN | 4.18 | EP300 |
| CD40LG | 3.66 | TBX5 |  | IL6 | 3.24 | IL1B |  | SRGN | 3.7 | TCF7L2 |
| CD70 | 3.86 | AATF |  | IL6 | 2.81 | NLRP3 |  | SRGN | 5.47 | NEUROD1 |
| CD70 | 3.84 | HMOX1 |  | IL6 | 3 | CDKN2A |  | SRXN1 | 2.73 | FOS |
| CD70 | 3.72 | FOXL2 |  | IL6 | 3.59 | TBX5 |  | SRXN1 | 2.76 | STAT5B |
| CD74 | 3.51 | NME2 |  | IL7 | 3.55 | NME1 |  | SST | 4.44 | PAX3 |
| CD74 | 4 | TBX3 |  | IL7 | 3 | NPM1 |  | SST | 3.99 | PAX7 |
| CD74 | 3 | NME1 |  | IL7 | 3.47 | TGFB1 |  | SST | 3.76 | TBX3 |
| CD74 | 3 | NPM1 |  | IL7 | 3.64 | NME2 |  | SSTR3 | 3.55 | TGFB1 |
| CD74 | 3 | TP53 |  | IL8 | 3 | IL1B |  | SSTR3 | 3.69 | PAX7 |
| CD74 | 4.3 | TBX5 |  | IL8 | 3 | ELF3 |  | SSTR3 | 3.8 | TBX3 |
| CD74 | 4.24 | TCF7L2 |  | IL8 | 3.3 | NFAM1 |  | SSTR3 | 3.7 | PAX3 |
| CD97 | 3.68 | HIF1A |  | IL8 | 3 | NFRKB |  | STAT1 | 3.35 | STAT1 |
| CD97 | 3.16 | ELF3 |  | IL8RB | 2 | IL1B |  | STAT3 | 3 | NFE2L2 |
| CDC2L1 | 4 | NOTCH2 |  | IL9 | 2.96 | NLRP3 |  | STAT3 | 4 | AP1GBP1 |
| CDC2L1 | 4.51 | PML |  | ING4 | 5.13 | TIAL1 |  | STAT5A | 4 | STAT3 |
| CDC2L2 | 3 | CDKN2A |  | INHBA | 7 | IL1B |  | STAT5B | 3.76 | STAT5B |
| CDC2L2 | 4 | NOTCH2 |  | IRAK2 | 3.76 | NFRKB |  | STK17A | 4.56 | STAT1 |
| CDK5 | 3.52 | SMAD3 |  | IRAK2 | 3.15 | TGFB1 |  | STK17A | 4.02 | RUNX3 |
| CDK5R1 | 4.33 | TBX3 |  | IRAK2 | 5 | RIPK2 |  | STK3 | 4 | PML |
| CDK5R1 | 4.09 | PAX7 |  | IRAK2 | 3.62 | NFATC3 |  | STK3 | 4.22 | TCF7L2 |
| CDK5R1 | 4.19 | PAX3 |  | IRAK2 | 5.25 | HMOX1 |  | STK4 | 3.48 | PML |
| CDK5R1 | 3.49 | TBX5 |  | ITCH | 4 | ELF3 |  | STK4 | 4 | TCF7L2 |
| CDKN1A | 3.58 | TP53 |  | ITCH | 4 | NFRKB |  | STK4 | 3.69 | PPARD |
| CDKN1A | 3.52 | PAX7 |  | ITGAL | 3.48 | FOS |  | STK4 | 3.52 | STAT1 |
| CDKN1A | 4.3 | PML |  | ITGAL | 3.73 | HIF1A |  | STK4 | 4.17 | EP300 |
| CDKN1A | 3.6 | NME2 |  | ITGAL | 3.21 | NFAM1 |  | STK4 | 3.54 | E2F1 |
| CDKN1A | 3.75 | NME1 |  | ITGB2 | 2.89 | CEBPB |  | STK4 | 4.11 | PYCARD |
| CDKN1A | 3.64 | PAX3 |  | ITGB2 | 2.85 | TGFB1 |  | TACR1 | 3.19 | TGFB1 |
| CDKN1B | 4.37 | E2F1 |  | ITGB2 | 4.21 | NFATC4 |  | TACR1 | 4.08 | RELA |
| CDKN1B | 3.58 | NLRP3 |  | ITGB2 | 2 | ELF3 |  | TACR1 | 3.61 | NFAM1 |
| CDKN2A | 3 | TBX5 |  | ITGB2 | 2.96 | HMOX1 |  | TAOK2 | 3 | RELA |
| CDKN2C | 3.64 | HTATIP2 |  | ITGB2 | 2.79 | RIPK2 |  | TAOK2 | 3.74 | CDKN2A |
| CDKN2C | 4.15 | PML |  | JMY | 3 | PAX3 |  | TAOK2 | 3.53 | RUNX3 |
| CDKN2D | 3.64 | TBX3 |  | JUN | 10 | JUN |  | TAOK2 | 4.16 | TGFB1 |
| CDKN2D | 3.91 | PYCARD |  | KCNMA1 | 3.7 | EP300 |  | TAOK2 | 4 | ARHGDIA |
| CDKN2D | 3 | TBX5 |  | KCNMA1 | 3.75 | FOXL2 |  | TAX1BP1 | 3 | TP63 |
| CDKN2D | 3 | PAX3 |  | KCNMA1 | 3.73 | HMOX1 |  | TAX1BP1 | 4.05 | IL1B |
| CDKN2D | 3.73 | PML |  | KIAA1967 | 3.59 | PML |  | TAX1BP1 | 4.04 | TCF7L2 |
| CDKN2D | 3.56 | TCF7L2 |  | KIAA1967 | 3.62 | TBX3 |  | TBX3 | 3 | SMAD3 |
| CDKN2D | 4.31 | ERC1 |  | KIAA1967 | 3.7 | PAX3 |  | TBX3 | 4 | TBX5 |
| CEBPG | 4 | CEBPG |  | KLRG1 | 3.73 | HMOX1 |  | TBX3 | 3 | TP53 |
| CECR2 | 5.1 | TBX3 |  | KNG1 | 4.08 | TBX5 |  | TBX3 | 3 | TIAL1 |
| CECR2 | 3.53 | PAX7 |  | KNG1 | 3.66 | TBX3 |  | TBX3 | 3 | TGFB1 |
| CECR2 | 4.47 | TBX5 |  | KRT18 | 4.02 | HMOX1 |  | TBX5 | 3 | PAX3 |
| CECR2 | 4.41 | PAX3 |  | KRT18 | 5 | SMAD3 |  | TCF7L2 | 3 | PML |
| CFB | 4.56 | TGFB1 |  | KRT18 | 3.86 | AATF |  | TCF7L2 | 3 | STAT1 |
| CFB | 3.23 | NFAM1 |  | KRT18 | 3 | TBX3 |  | TGFB1 | 4 | CDKN2A |
| CFB | 3.32 | HIF1A |  | LGALS1 | 3.74 | NME2 |  | TGFB1 | 3 | NME2 |
| CFD | 3.75 | NFRKB |  | LGALS1 | 3.82 | NME1 |  | TGFB1 | 3 | RUNX3 |
| CFH | 3 | ITCH |  | LGALS12 | 4.11 | NLRP3 |  | TGFB1 | 3 | TP53 |
| CFH | 3 | ELF3 |  | LIG4 | 4.53 | JMY |  | TGFB1 | 3 | TBX5 |
| CFH | 2 | SIGIRR |  | LIG4 | 4.04 | SMAD3 |  | TGFB2 | 4 | SMAD3 |
| CFH | 3.27 | ABCF1 |  | LTA4H | 3 | ELF3 |  | TGFB2 | 5 | TBX3 |
| CFL1 | 3.54 | SMAD3 |  | LTA4H | 2.92 | HMOX1 |  | TGFB2 | 3.73 | TGFB1 |
| CFL1 | 5.14 | RELA |  | LTB4R | 4.37 | NFE2L1 |  | TGFB2 | 3.6 | RELA |
| CFL1 | 5 | ARHGDIA |  | LTB4R | 3.15 | CEBPB |  | TGFB2 | 4.09 | TP53 |
| CFL1 | 4.35 | TP53 |  | LY75 | 3.49 | HIF1A |  | TGFB2 | 3.5 | HMOX1 |
| CFLAR | 3.48 | PPARD |  | LY75 | 4.49 | TGFB1 |  | TGFB2 | 3.6 | ETS1 |
| CFLAR | 4.34 | FOXL2 |  | LY75 | 3.47 | RIPK2 |  | TIA1 | 6.16 | PML |
| CFLAR | 3.6 | NME1 |  | LY75 | 3.22 | NFATC4 |  | TIA1 | 3.5 | TBX3 |
| CFLAR | 3.5 | NPM1 |  | LY75 | 2.84 | NLRP3 |  | TIA1 | 4 | TBX5 |
| CFLAR | 4 | AATF |  | LY75 | 3.42 | NFX1 |  | TIA1 | 3.54 | JMY |
| CFLAR | 6 | CDKN2A |  | LYST | 4.65 | JMY |  | TKT | 2.79 | NFE2L2 |
| CFLAR | 3.53 | SMAD3 |  | LYST | 4 | PAX3 |  | TLR2 | 4 | TBX3 |
| CFLAR | 3.56 | NME2 |  | LYST | 4.38 | TBX3 |  | TLR2 | 3 | SMAD3 |
| CFLAR | 4 | HMOX1 |  | LYST | 3.77 | TBX5 |  | TLR2 | 4.2 | PAX3 |
| CFLAR | 4 | NOTCH2 |  | LYZ | 3.15 | NFX1 |  | TLR2 | 3.52 | HMOX1 |
| CFP | 4.04 | HIF1A |  | LYZ | 4 | HIF1A |  | TNFAIP6 | 2 | IL1B |
| CFP | 3.12 | ABCF1 |  | LYZ | 3.62 | ABCF1 |  | TNFAIP6 | 3.22 | HMOX1 |
| CGB | 3.62 | PPARD |  | MAEA | 4.89 | TP63 |  | TNFAIP8 | 4 | PML |
| CHEK2 | 3.53 | TP53 |  | MAL | 4.58 | TNF |  | TNFAIP8 | 3.5 | TCF7L2 |
| CHEK2 | 3 | NPM1 |  | MALT1 | 3 | NPM1 |  | TNFAIP8 | 3.6 | E2F1 |
| CHEK2 | 3.91 | NME1 |  | MALT1 | 3.94 | NME1 |  | TNFAIP8 | 4.04 | ERC1 |
| CHRFAM7A | 3.45 | RIPK2 |  | MAP1S | 3 | ARHGDIA |  | TNFRSF10A | 3.48 | TP73 |
| CHST2 | 5.05 | NFRKB |  | MAP1S | 4.5 | RELA |  | TNFRSF10A | 3.51 | TP63 |
| CIAPIN1 | 3.5 | NME2 |  | MAP3K10 | 4 | TBX3 |  | TNFRSF10A | 3.52 | TCF7L2 |
| CIAPIN1 | 4 | NME1 |  | MAPK1 | 3.66 | BCL10 |  | TNFRSF10B | 3 | NME1 |
| CIAPIN1 | 3.67 | TP53 |  | MAPK1 | 5 | STAT1 |  | TNFRSF10B | 3.6 | NPM1 |
| CIAPIN1 | 3 | NPM1 |  | MAPK1 | 4 | ERC1 |  | TNFRSF10D | 3.52 | TP73 |
| CIB1 | 3.52 | NPM1 |  | MAPK1 | 3.68 | TBX3 |  | TNFRSF10D | 3.92 | NME1 |
| CIB1 | 3.63 | NME1 |  | MAPK1 | 3.7 | TCF7L2 |  | TNFRSF10D | 3 | NPM1 |
| CIDEA | 4 | TBX3 |  | MAPK1 | 3.64 | TBX5 |  | TNFRSF18 | 3.6 | PAX3 |
| CIDEB | 3.52 | TP53 |  | MAPK8 | 4.04 | TBX5 |  | TNFRSF19 | 4 | NME1 |
| CIDEB | 3 | NPM1 |  | MAPK8IP2 | 4.23 | PAX3 |  | TNFRSF19 | 4.1 | TP53 |
| CIDEB | 3.91 | NME1 |  | MAPK8IP2 | 4 | TBX3 |  | TNFRSF19 | 4 | NPM1 |
| CLCF1 | 3.49 | TBX3 |  | MAPK8IP2 | 3.96 | TIAL1 |  | TNFRSF19 | 3 | NME2 |
| CLN8 | 5 | NFKBIB |  | MAPK8IP2 | 5.02 | TGFB1 |  | TNFRSF19 | 3.54 | TBX3 |
| CLN8 | 3.22 | RELA |  | MAPK8IP2 | 3.96 | CDKN2A |  | TNFRSF1A | 3.33 | HMOX1 |
| CLN8 | 4.33 | AP1GBP1 |  | MAPK8IP2 | 6.12 | RUNX3 |  | TNFRSF1A | 3.83 | TGFB1 |
| COL4A3 | 3.6 | TBX3 |  | MASP1 | 3.74 | RELA |  | TNFRSF1A | 3.24 | RIPK2 |
| COL4A3 | 3 | FOXL2 |  | MCL1 | 4 | TP63 |  | TNFRSF25 | 3.56 | PAX3 |
| COL4A3 | 4.56 | EP300 |  | MCL1 | 3.98 | STAT1 |  | TNFRSF25 | 4 | NOTCH2 |
| COL4A3 | 4 | PAX3 |  | MCL1 | 4 | BCL10 |  | TNFRSF25 | 4 | PML |
| COL4A3 | 3.58 | TBX5 |  | MCL1 | 3.85 | IL1B |  | TNFRSF25 | 4 | CDKN2A |
| CRADD | 3 | CDKN2A |  | MCL1 | 4.8 | ETS1 |  | TNFRSF6B | 4 | ERC1 |
| CRADD | 4.49 | TCF7L2 |  | MCL1 | 3 | CDKN2A |  | TNFRSF6B | 4 | PML |
| CREB1 | 3 | STAT1 |  | MDH1 | 2.87 | CREB1 |  | TNFRSF6B | 3.51 | IL1B |
| CREB1 | 3 | RELA |  | MEFV | 3.55 | NLRP3 |  | TNFRSF6B | 4 | CDKN2A |
| CREBBP | 3 | RELA |  | MGLL | 5 | NFRKB |  | TNFRSF6B | 3.49 | RELA |
| CROP | 4.59 | NPM1 |  | MGLL | 4 | ITCH |  | TNFRSF6B | 4.84 | TP63 |
| CROP | 4.44 | NLRP3 |  | MGST3 | 3.99 | CREB1 |  | TNFRSF6B | 3.49 | TCF7L2 |
| CRYAA | 3.99 | TBX3 |  | MGST3 | 2.92 | CREBBP |  | TNFRSF6B | 3.62 | ARHGDIA |
| CRYAA | 3.81 | EP300 |  | MGST3 | 2.91 | ATF1 |  | TNFRSF8 | 3.66 | PAX3 |
| CRYAA | 3.83 | PML |  | MIF | 4.7 | NME1 |  | TNFRSF8 | 3.58 | JMY |
| CRYAA | 3.68 | PAX3 |  | MIF | 3.66 | ARHGDIA |  | TNFRSF9 | 4.19 | HMOX1 |
| CRYAB | 4.02 | FOXL2 |  | MIF | 3 | PML |  | TNFRSF9 | 3.7 | AATF |
| CRYAB | 3.8 | AATF |  | MMP25 | 3 | TGFB1 |  | TNFRSF9 | 3 | TBX3 |
| CRYAB | 4.55 | HMOX1 |  | MPO | 4.26 | TBX5 |  | TNFRSF9 | 3.53 | FOXL2 |
| CSE1L | 3.88 | NME1 |  | MTCH1 | 3.49 | PML |  | TNFSF10 | 4 | NME2 |
| CTSB | 4 | PAX3 |  | MTCH1 | 3 | STAT1 |  | TNFSF10 | 3 | TBX3 |
| CTSB | 3.52 | ERC1 |  | MTCH1 | 4 | HTATIP2 |  | TNFSF10 | 3 | TP53 |
| CTSB | 2.85 | STAT3 |  | MTF1 | 2.78 | NFKBIB |  | TNFSF10 | 4 | NME1 |
| CTSB | 2.78 | CREB1 |  | MTF1 | 2.74 | STAT5B |  | TNFSF10 | 4 | NPM1 |
| CTSB | 3 | MAF |  | MTF1 | 3.39 | NFE2L2 |  | TNFSF12 | 3 | NPM1 |
| CUL1 | 3.48 | CDKN2A |  | MTF1 | 3 | FOS |  | TNFSF12 | 3 | NME1 |
| CUL1 | 3.68 | TGFB1 |  | MX1 | 3.5 | HMOX1 |  | TNFSF12 | 3.5 | TP73 |
| CUL2 | 5.15 | TIAL1 |  | MX1 | 3.73 | HTATIP2 |  | TNFSF15 | 3.86 | TP63 |
| CUL4A | 3.57 | E2F1 |  | MX1 | 3.53 | FOXL2 |  | TNFSF15 | 3.73 | PPARD |
| CUL4A | 3.84 | PML |  | MX1 | 3.6 | STAT1 |  | TNFSF15 | 5 | IL1B |
| CUL4A | 3.71 | TCF7L2 |  | MYO18A | 3 | TP73 |  | TNFSF8 | 4.05 | PAX3 |
| CX3CL1 | 2 | ELF3 |  | NAE1 | 4.36 | NPM1 |  | TNFSF8 | 3.64 | TBX5 |
| CX3CL1 | 3 | FOS |  | NAIF1 | 4 | STAT1 |  | TOLLIP | 4.01 | SBNO2 |
| CX3CL1 | 2 | HMOX1 |  | NCR1 | 3.49 | TBX3 |  | TOLLIP | 3.42 | NFX1 |
| CX3CL1 | 2 | CEBPB |  | NCR1 | 3.72 | PAX7 |  | TOLLIP | 4 | RELA |
| CX3CL1 | 3 | HIF1A |  | NCR1 | 4.27 | JMY |  | TOLLIP | 3.46 | NFRKB |
| CXCL1 | 3.14 | HIF1A |  | NCR1 | 4.1 | PAX3 |  | TOLLIP | 3.57 | TGFB1 |
| CXCL1 | 3 | ITCH |  | NCR3 | 3 | TGFB1 |  | TOP2A | 3.86 | TP53 |
| CXCL1 | 3.51 | NFX1 |  | NF1 | 4.21 | NME1 |  | TOP2A | 3 | NME2 |
| CXCL1 | 4 | ELF3 |  | NF1 | 3.58 | HMOX1 |  | TOP2A | 4 | NME1 |
| CXCL1 | 3.26 | ABCF1 |  | NF1 | 4.67 | TCF7L2 |  | TOP2A | 4 | NPM1 |
| CXCL2 | 3.58 | FOS |  | NF1 | 4.23 | FOXL2 |  | TP53BP2 | 3.87 | ERC1 |
| CXCL6 | 3.31 | TGFB1 |  | NFAM1 | 3 | NFAM1 |  | TP53I3 | 4.06 | HMOX1 |
| CXCL6 | 3.62 | ABCF1 |  | NFATC3 | 3 | RIPK2 |  | TP53I3 | 3.8 | AATF |
| CXCL6 | 3.19 | ITCH |  | NFE2L1 | 3 | RELA |  | TP53I3 | 3.54 | NME1 |
| CXCL6 | 4.5 | HIF1A |  | NFKBIB | 4 | NFKBIB |  | TP63 | 4 | CDKN2A |
| CXCL6 | 3 | NFX1 |  | NFKBIB | 3 | CREB1 |  | TPST1 | 2 | ELF3 |
| CXCR4 | 3.41 | HIF1A |  | NFKBIB | 2 | HSF1 |  | TPT1 | 3.57 | PPARD |
| CXCR4 | 2 | IL1B |  | NFRKB | 3 | NFATC3 |  | TPT1 | 4 | NPM1 |
| CYBA | 3.36 | NFAM1 |  | NFX1 | 2 | SIGIRR |  | TPT1 | 3.93 | EP300 |
| CYBB | 3 | ELF3 |  | NFX1 | 4 | ITCH |  | TRADD | 3.7 | ETS1 |
| CYBB | 2.82 | CEBPB |  | NFX1 | 3 | HIF1A |  | TRADD | 3.93 | PML |
| CYBB | 2 | SIGIRR |  | NLRP1 | 3.73 | TP53 |  | TRAF2 | 3.59 | NME1 |
| CYBB | 3 | HMOX1 |  | NLRP1 | 3 | NPM1 |  | TRAF2 | 3.51 | NME2 |
| CYCS | 4.23 | TP63 |  | NLRP1 | 3.73 | NME2 |  | TRAF7 | 3.66 | NOTCH2 |
| CYCS | 4 | PML |  | NLRP1 | 4 | PML |  | TRAF7 | 3.97 | TBX5 |
| CYCS | 3.62 | NPM1 |  | NLRP1 | 4 | CDKN2A |  | TRIAP1 | 3.65 | TBX3 |
| CYFIP2 | 4 | CDKN2A |  | NLRP1 | 4.02 | NME1 |  | TRIAP1 | 3.48 | NPM1 |
| CYFIP2 | 4 | HTATIP2 |  | NLRP1 | 3.57 | TP73 |  | TSPO | 4 | PML |
| CYFIP2 | 3.9 | PYCARD |  | NLRP12 | 4.54 | TBX3 |  | TUBB | 3 | TP53 |
| CYP4F11 | 3.99 | NFRKB |  | NLRP12 | 3.34 | NFAM1 |  | TUBB | 3 | NME2 |
| CYP4F11 | 3.38 | ELF3 |  | NLRP12 | 3.27 | NFATC4 |  | TUBB | 4 | NME1 |
| DAD1 | 5.66 | NME1 |  | NLRP12 | 3.86 | TBX5 |  | TUBB | 4 | NPM1 |
| DAP | 3.83 | PML |  | NLRP2 | 4 | TP73 |  | TUBB | 4 | ARHGDIA |
| DAP | 5.06 | PYCARD |  | NME1 | 4 | TP53 |  | TUBB | 3 | TGFB1 |
| DAP | 4 | HTATIP2 |  | NME1 | 3 | TBX3 |  | TXNIP | 3 | NFKBIB |
| DAP | 3.73 | E2F1 |  | NME2 | 4 | TP53 |  | TXNRD2 | 3 | RELA |
| DAPK1 | 3.59 | ERC1 |  | NME2 | 4 | NME1 |  | TXNRD2 | 4 | HSF1 |
| DAPK1 | 3 | TP63 |  | NME2 | 3 | TBX3 |  | TXNRD2 | 3 | NFKBIB |
| DAPK2 | 3.49 | TCF7L2 |  | NME3 | 4.96 | TP63 |  | UCP3 | 3 | CREBBP |
| DAPK2 | 3.97 | CDKN2A |  | NME3 | 3.7 | BCL10 |  | UCP3 | 3 | RELA |
| DAPK2 | 4.36 | TBX3 |  | NME5 | 3.95 | FOS |  | UCP3 | 3 | AP1GBP1 |
| DAPK2 | 3.49 | TGFB1 |  | NME5 | 3.82 | TGFB1 |  | UCP3 | 4.29 | ATF1 |
| DAPK3 | 3.71 | ETS1 |  | NME5 | 4 | NPM1 |  | VCP | 3 | ARHGDIA |
| DAPK3 | 4 | TBX3 |  | NME5 | 4.24 | TP53 |  | VDAC1 | 3.57 | E2F1 |
| DAPK3 | 4.05 | SMAD3 |  | NME5 | 4.13 | NME2 |  | VEGFA | 4 | NOTCH2 |
| DAPK3 | 5.08 | RELA |  | NME5 | 4.4 | NME1 |  | VEGFA | 4 | CDKN2A |
| DAPK3 | 5 | ARHGDIA |  | NME5 | 4 | TBX3 |  | VEGFA | 4.45 | TBX3 |
| DAPK3 | 3.6 | PML |  | NME5 | 3.64 | NFE2L2 |  | VNN1 | 3.31 | HIF1A |
| DAXX | 5 | CDKN2A |  | NME5 | 3 | NFKBIB |  | VNN1 | 3.39 | TGFB1 |
| DAXX | 3.75 | TP63 |  | NOL3 | 4 | TBX3 |  | XPA | 3 | CREB1 |
| DAXX | 4 | PML |  | NOL3 | 4 | SMAD3 |  | XPA | 2.97 | RELA |
| DCC | 3.58 | PML |  | NOL3 | 3.84 | FOXL2 |  | XPA | 4.11 | ATF1 |
| DDAH2 | 5 | TP63 |  | NOL3 | 3.5 | HMOX1 |  | YARS | 4 | NOTCH2 |
| DDAH2 | 4 | ERC1 |  | NOL3 | 4 | PML |  | YWHAB | 3.51 | NME1 |
| DDAH2 | 4 | HTATIP2 |  | NOTCH2 | 5 | CDKN2A |  | YWHAB | 3.64 | TP63 |
| DDAH2 | 3.48 | PPARD |  | NOTCH2 | 4.03 | NOTCH2 |  | YWHAZ | 4 | SMAD3 |
| DDAH2 | 4 | CDKN2A |  | NOX1 | 3 | NLRP3 |  | YWHAZ | 3.61 | NOTCH2 |
| DDX41 | 3.62 | CDKN2A |  | NOX1 | 3.46 | RELA |  | YWHAZ | 4.3 | NPM1 |
| DEDD | 5 | ARHGDIA |  | NOX4 | 3.93 | NFATC3 |  | YWHAZ | 6 | STAT1 |
| DEDD | 3.96 | TP53 |  | NOX5 | 4.2 | PML |  | YWHAZ | 3.97 | PML |
| DEDD | 3.94 | SMAD3 |  | NOX5 | 4 | STAT1 |  | ZNF346 | 3.63 | E2F1 |
| DEDD | 3.74 | STAT1 |  | NOX5 | 3.59 | TGFB1 |  | ZNF346 | 3.61 | AHR |
| DEDD | 4.68 | RELA |  | NOX5 | 4.02 | CDKN2A |  | ZNF346 | 3.6 | PPARD |
| DEDD2 | 5 | CDKN2A |  | NPM1 | 3 | NME2 |  | ZNF346 | 3.5 | NOTCH2 |
| DEDD2 | 4 | NOTCH2 |  | NPM1 | 3 | TBX3 |  |  |  |  |
| DFFA | 4 | CDKN2A |  | NPM1 | 4 | NME1 |  |  |  |  |
