# Supplementary material for: Suppressed Expression of T-Box Transcription Factors Is Involved in Senescence in Chronic Obstructive Pulmonary Disease
Source: PLoS Comput Biol. 2012 Jul 19;8(7):e1002597. doi: 10.1371/journal.pcbi.1002597 (PMC3400575; doi:10.1371/journal.pcbi.1002597)
Supplement: Table S2 — Direct Connections to TBX2 in the CLR-Generated Network. The data in this table correspond to Figure S2A. (DOC) [file pcbi.1002597.s006.doc]

**Table S2. Direct Connections to TBX2 in the CLR-Generated Network (See Figure S2A).**

Note: Certain genes are represented by multiple probe sets and so occur more than once in the network.

| **GENE** | **CLR_LIKELIHOOD_ESTIMATE_(ABSOLUTE)** |  | **GENE** | **CLR_LIKELIHOOD_ESTIMATE_(ABSOLUTE)** |  | **GENE** | **CLR_LIKELIHOOD_ESTIMATE_(ABSOLUTE)** |  | **GENE** | **CLR_LIKELIHOOD_ESTIMATE_(ABSOLUTE)** |
| --- | --- | --- | --- | --- | --- | --- | --- | --- | --- | --- |
| BCL10 | 2.96 |  | DPT | 2.83 |  | LMNB1 | 2.66 |  | RFK | 2.76 |
| BCL10 | 2.78 |  | DPY19L1 | 3.2 |  | LMX1B | 2.85 |  | RFK | 2.69 |
| BCL10 | 2.8 |  | DPYD | 2.74 |  | LOC100127918 | 3 |  | RFNG | 2.52 |
| CREBBP | 3.02 |  | DPYSL4 | 2.81 |  | LOC100129064 | 2.57 |  | RFPL1 | 2.62 |
| HIF1A | 3.35 |  | DPYSL4 | 2.72 |  | LOC100129448 | 2.55 |  | RFPL1 | 2.57 |
| HIF1A | 3.1 |  | DPYSL4 | 2.73 |  | LOC100130134 | 3.84 |  | RFPL1S | 2.58 |
| LOC652346 | 3.19 |  | DRD2 | 3.63 |  | LOC100130134 | 2.5 |  | RFPL3S | 2.57 |
| NFE2L2 | 2.61 |  | DRD2 | 3.5 |  | LOC100130741 | 2.82 |  | RFX1 | 3.6 |
| NFKBIB | 2.5 |  | DRD2 | 3.65 |  | LOC100130741 | 3.5 |  | RGPD4 | 2.82 |
| NKX2-8 | 2.64 |  | DRD2 | 2.7 |  | LOC100130955 | 2.81 |  | RGS11 | 3.42 |
| NOTCH2 | 2.8 |  | DRD3 | 4.02 |  | LOC100131509 | 3.32 |  | RGS12 | 3.24 |
| NOTCH2 | 2.64 |  | DSG1 | 3.34 |  | LOC100131509 | 2.73 |  | RGS12 | 2.71 |
| SMAD3 | 2.64 |  | DSN1 | 2.82 |  | LOC100132214 | 2.74 |  | RGS12 | 3.14 |
| STAT1 | 2.58 |  | DSP | 2.93 |  | LOC100132832 | 2.66 |  | RGS12 | 2.68 |
| STAT3 | 2.76 |  | DTNB | 2.52 |  | LOC100132923 | 3.48 |  | RGS14 | 2.79 |
| TCF7L2 | 2.86 |  | DTX2 | 2.72 |  | LOC100132941 | 2.7 |  | RGS14 | 2.66 |
| TP73 | 3.07 |  | DTX2 | 2.7 |  | LOC100133432 | 2.96 |  | RGS14 | 4.3 |
| A2BP1 | 2.77 |  | DTX3 | 2.91 |  | LOC100133432 | 3.45 |  | RGS14 | 2.78 |
| AAMP | 2.97 |  | DTX3 | 2.51 |  | LOC100133432 | 3.56 |  | RGS14 | 2.95 |
| AANAT | 3.63 |  | DTX3 | 2.52 |  | LOC100133572 | 4.04 |  | RGS14 | 3.25 |
| AARSD1 | 2.8 |  | DTX3 | 2.58 |  | LOC100133946 | 3.17 |  | RHBDD3 | 2.67 |
| AARSD1 | 2.81 |  | DTX3 | 3.59 |  | LOC100134089 | 3.02 |  | RHBDD3 | 3.16 |
| AARSD1 | 3.04 |  | DTX3 | 2.59 |  | LOC100134089 | 2.72 |  | RHBDD3 | 2.59 |
| ABCA2 | 3.09 |  | DUSP21 | 2.98 |  | LOC100134363 | 3.18 |  | RHBDL1 | 2.89 |
| ABCA2 | 2.94 |  | DUSP26 | 3.17 |  | LOC100134410 | 2.99 |  | RHBG | 3.36 |
| ABCA2 | 2.71 |  | DUSP3 | 2.58 |  | LOC100134427 | 4.38 |  | RHBG | 2.78 |
| ABCA2 | 2.72 |  | DUSP7 | 2.89 |  | LOC100134427 | 3.5 |  | RHBG | 2.93 |
| ABCA4 | 2.8 |  | DUSP7 | 3.4 |  | LOC100134427 | 3.31 |  | RHCE | 2.85 |
| ABCA4 | 3.2 |  | DVL3 | 2.96 |  | LOC100134498 | 3.36 |  | RHCG | 3.17 |
| ABCA4 | 2.79 |  | DYNC1I2 | 2.55 |  | LOC128192 | 2.92 |  | RHCG | 2.88 |
| ABCA5 | 2.74 |  | DYNC1I2 | 2.71 |  | LOC131185 | 3.3 |  | RHCG | 4.29 |
| ABCB8 | 2.98 |  | DYRK2 | 2.52 |  | LOC147343 | 2.56 |  | RHOD | 2.98 |
| ABCB8 | 2.86 |  | DZIP3 | 2.73 |  | LOC220077 | 3.09 |  | RHOG | 2.96 |
| ABCB8 | 3.47 |  | E2F2 | 3.65 |  | LOC220077 | 2.77 |  | RIMS2 | 3.14 |
| ABCC3 | 2.91 |  | E2F2 | 3.54 |  | LOC388076 | 2.56 |  | RLBP1 | 2.58 |
| ABCC8 | 3.09 |  | E2F2 | 2.89 |  | LOC388907 | 2.65 |  | RLN1 | 2.73 |
| ABCC9 | 3.29 |  | E2F4 | 3.02 |  | LOC400642 | 3.15 |  | RNASET2 | 2.94 |
| ABCF3 | 3.5 |  | E2F5 | 2.6 |  | LOC440792 | 3.16 |  | RNASET2 | 2.97 |
| ABHD4 | 3.42 |  | E4F1 | 2.55 |  | LOC440792 | 4.2 |  | RND2 | 2.95 |
| ABHD4 | 3.6 |  | E4F1 | 3.94 |  | LOC440792 | 2.83 |  | RND2 | 2.86 |
| ABHD8 | 3.03 |  | E4F1 | 2.53 |  | LOC442240 | 3.16 |  | RNF11 | 2.82 |
| ABI1 | 2.56 |  | ECEL1 | 2.93 |  | LOC442240 | 2.91 |  | RNF11 | 2.55 |
| ABO | 3.22 |  | ECEL1 | 2.92 |  | LOC442240 | 2.54 |  | RNF114 | 3.19 |
| ABO | 4.77 |  | ECEL1 | 3.78 |  | LOC51190 | 2.6 |  | RNF126 | 3.05 |
| ABO | 3.13 |  | EDN3 | 2.75 |  | LOC51190 | 3.82 |  | RNF40 | 2.65 |
| ABTB2 | 2.5 |  | EDNRA | 3.7 |  | LOC642131 | 3.83 |  | RNF44 | 2.77 |
| ACADS | 2.65 |  | EDNRA | 2.59 |  | LOC642131 | 3.28 |  | RNF5 | 2.96 |
| ACADS | 2.73 |  | EDNRA | 3.09 |  | LOC643503 | 3.14 |  | RNF5 | 4.19 |
| ACHE | 2.65 |  | EEF1A2 | 2.66 |  | LOC645468 | 3.17 |  | RNF5 | 2.79 |
| ACLY | 2.62 |  | EEF1A2 | 2.75 |  | LOC645961 | 2.72 |  | ROCK1 | 2.56 |
| ACOT9 | 2.52 |  | EFHA1 | 2.65 |  | LOC648390 | 2.85 |  | RP1-21O18.1 | 2.88 |
| ACOX1 | 2.7 |  | EFHA1 | 2.81 |  | LOC652147 | 3.21 |  | RP11-345P4.4 | 2.58 |
| ACOX1 | 3.46 |  | EFHA1 | 2.72 |  | LOC652346 | 3.19 |  | RP6-213H19.1 | 2.59 |
| ACOX1 | 3.12 |  | EFNA4 | 2.76 |  | LOC653166 | 3.33 |  | RPA2 | 2.53 |
| ACP6 | 2.53 |  | EFNA4 | 3.04 |  | LOC653166 | 2.5 |  | RPA2 | 2.99 |
| ACSL3 | 3.64 |  | EFR3A | 3.71 |  | LOC653562 | 2.58 |  | RPAIN | 2.86 |
| ACSL3 | 3.2 |  | EFR3A | 3.18 |  | LOC653562 | 2.68 |  | RPE65 | 2.79 |
| ACSL5 | 2.99 |  | EGR4 | 2.71 |  | LOC653566 | 2.5 |  | RPE65 | 2.55 |
| ACSL5 | 3.1 |  | EHHADH | 2.52 |  | LOC654342 | 2.84 |  | RPL10L | 2.93 |
| ACSL6 | 3.18 |  | EIF1 | 2.51 |  | LOC727842 | 3.28 |  | RPL10L | 3.57 |
| ACSL6 | 2.81 |  | EIF1 | 2.61 |  | LOC728137 | 3.16 |  | RPL23 | 2.82 |
| ACSL6 | 3.38 |  | EIF1 | 2.97 |  | LOC728137 | 3.24 |  | RPL23 | 2.66 |
| ACTB | 2.89 |  | EIF1AP1 | 2.8 |  | LOC728361 | 2.72 |  | RPL28 | 2.54 |
| ACTG1 | 2.65 |  | EIF1AP1 | 2.69 |  | LOC728866 | 2.57 |  | RPL39L | 2.94 |
| ACTG1 | 2.56 |  | EIF1AP1 | 3.87 |  | LOC728944 | 3.31 |  | RPN1 | 2.56 |
| ACTL6B | 3.7 |  | EIF1B | 2.55 |  | LOC730272 | 2.59 |  | RPRD2 | 2.8 |
| ACTL7A | 3.31 |  | EIF1B | 2.87 |  | LOC731884 | 2.68 |  | RPRD2 | 2.67 |
| ACTL7A | 3.65 |  | EIF1B | 2.67 |  | LOC732160 | 2.78 |  | RPS20 | 2.59 |
| ACTL7A | 4.63 |  | EIF2C3 | 2.84 |  | LOC90379 | 2.55 |  | RPS6KA1 | 3.09 |
| ACTL7B | 4.06 |  | EIF3A | 3.64 |  | LOC90379 | 2.64 |  | RPS6KA1 | 2.51 |
| ACTN1 | 3.47 |  | EIF3A | 4.14 |  | LOC90925 | 2.63 |  | RPS6KA5 | 2.77 |
| ACTR10 | 2.97 |  | EIF3A | 3.18 |  | LOC90925 | 2.64 |  | RQCD1 | 2.82 |
| ACTR10 | 2.91 |  | EIF3B | 2.57 |  | LPA | 3.07 |  | RRBP1 | 2.93 |
| ACTR3 | 2.81 |  | EIF3M | 3.16 |  | LPA | 3.41 |  | RREB1 | 2.88 |
| ACTR3 | 3.13 |  | EIF3M | 2.62 |  | LPHN3 | 2.53 |  | RRP12 | 3.03 |
| ACTR3 | 3.68 |  | EIF4E | 2.99 |  | LPIN1 | 2.68 |  | RRP12 | 3.08 |
| ACTR3 | 3.32 |  | EIF4G2 | 3.85 |  | LPP | 2.99 |  | RRP12 | 3.53 |
| ACTR3 | 2.63 |  | EIF4G2 | 2.82 |  | LRCH4 | 3.74 |  | RRP12 | 3.43 |
| ACTR3 | 2.68 |  | EIF5 | 2.6 |  | LRRC14 | 3.92 |  | RRP1B | 2.68 |
| ACTR3 | 3.05 |  | ELA3A | 3.87 |  | LRRC14 | 2.91 |  | RRP1B | 3.6 |
| ACTR5 | 2.73 |  | ELA3A | 3.3 |  | LRRC14 | 3.36 |  | RSHL1 | 2.66 |
| ADAM12 | 2.51 |  | ELA3A | 3.07 |  | LRRC19 | 2.56 |  | RTEL1 | 2.57 |
| ADAM15 | 3.16 |  | ELA3A | 3.1 |  | LRRC42 | 2.64 |  | RTN4 | 2.57 |
| ADAM19 | 2.5 |  | ELAC1 | 2.77 |  | LRRC42 | 2.54 |  | RTN4 | 3.15 |
| ADAM3A | 3.27 |  | ELAC2 | 2.58 |  | LRRC68 | 2.74 |  | RTN4 | 2.55 |
| ADAM5P | 2.6 |  | ELF1 | 2.74 |  | LRRC68 | 2.61 |  | RTN4 | 3.16 |
| ADAM9 | 2.73 |  | ELF1 | 2.92 |  | LRRFIP1 | 2.97 |  | RUFY2 | 2.91 |
| ADAMTS13 | 2.66 |  | ELK1 | 2.55 |  | LRRFIP1 | 3.18 |  | RUFY2 | 2.5 |
| ADAMTS13 | 2.69 |  | ELL | 2.72 |  | LRRFIP1 | 3.62 |  | RUFY3 | 3.23 |
| ADAMTS2 | 2.63 |  | ELN | 2.79 |  | LRRN2 | 2.79 |  | RUNDC2B | 2.78 |
| ADAMTS7 | 3.39 |  | ELN | 2.63 |  | LRTM1 | 2.55 |  | RUNX1 | 2.72 |
| ADAMTS8 | 3.4 |  | ELN | 3.4 |  | LRTM1 | 2.75 |  | RUNX1 | 2.52 |
| ADAMTS8 | 2.6 |  | ELN | 2.61 |  | LRTM1 | 3.18 |  | RUNX1 | 2.82 |
| ADCK2 | 4.08 |  | ELOVL4 | 2.8 |  | LSS | 2.67 |  | RUNX1T1 | 4.56 |
| ADCY1 | 2.64 |  | EMID1 | 2.68 |  | LTBP1 | 2.72 |  | RUNX1T1 | 2.51 |
| ADCY1 | 2.61 |  | EML2 | 2.8 |  | LTBP1 | 2.59 |  | RUNX1T1 | 3.22 |
| ADCY1 | 2.54 |  | EML2 | 2.53 |  | LTBP4 | 2.66 |  | RUNX2 | 2.72 |
| ADD3 | 3.63 |  | EML2 | 2.64 |  | LTBP4 | 2.98 |  | RUNX2 | 2.61 |
| ADM2 | 3.12 |  | EML3 | 2.81 |  | LTBP4 | 3.6 |  | RUSC2 | 3.02 |
| ADM2 | 3.46 |  | EN1 | 2.61 |  | LTBP4 | 2.53 |  | RYBP | 3.41 |
| ADM2 | 3.23 |  | EN1 | 3.7 |  | LTBP4 | 3.35 |  | RYBP | 2.84 |
| ADORA2A | 2.63 |  | ENO1 | 3.14 |  | LTBP4 | 3.15 |  | RYR2 | 3.23 |
| ADRA1A | 2.82 |  | ENPEP | 2.69 |  | LTC4S | 2.73 |  | S100A13 | 2.71 |
| ADRA1A | 3.2 |  | ENPP1 | 2.83 |  | LTC4S | 3.03 |  | S100A7 | 2.64 |
| ADRA1B | 5.16 |  | ENTPD4 | 2.89 |  | LTC4S | 3.47 |  | S100PBP | 2.62 |
| ADRA1B | 3.29 |  | EP400 | 2.52 |  | LY6G6C | 2.57 |  | S100PBP | 3.15 |
| ADRA1B | 3.92 |  | EPAG | 2.93 |  | LY6G6D | 3.07 |  | S1PR5 | 2.93 |
| ADRA2B | 3.56 |  | EPHA1 | 3.88 |  | LYL1 | 3.52 |  | S1PR5 | 3.23 |
| ADRA2C | 2.66 |  | EPHA1 | 2.62 |  | LYN | 2.81 |  | S1PR5 | 3.19 |
| ADRB2 | 2.72 |  | EPHA1 | 2.77 |  | LYN | 2.57 |  | SAE1 | 2.53 |
| ADRB3 | 2.7 |  | EPHA3 | 3.54 |  | LYN | 2.78 |  | SAFB | 2.7 |
| ADRB3 | 2.93 |  | EPHA3 | 2.65 |  | LYPLA1 | 3.32 |  | SAG | 2.69 |
| ADRBK1 | 3.06 |  | EPHA3 | 2.82 |  | LYPLA1 | 3.19 |  | SAP30 | 2.82 |
| ADRBK1 | 2.74 |  | EPHB1 | 2.82 |  | LYPLA1 | 2.89 |  | SAP30 | 2.53 |
| AEN | 3.31 |  | EPHB1 | 2.64 |  | LZTR1 | 3 |  | SAP30 | 3.11 |
| AEN | 2.7 |  | EPHB1 | 3 |  | LZTS1 | 3.15 |  | SAP30 | 3.38 |
| AFAP1 | 2.75 |  | EPN2 | 3.03 |  | LZTS1 | 3.2 |  | SAR1B | 3.03 |
| AFAP1 | 2.6 |  | EPN2 | 3.34 |  | LZTS1 | 2.95 |  | SARDH | 2.5 |
| AFF3 | 2.91 |  | EPN2 | 3.18 |  | LZTS1 | 3.43 |  | SBF1 | 2.83 |
| AFF3 | 2.94 |  | EPOR | 2.57 |  | MAEA | 2.58 |  | SBF1 | 3.68 |
| AGBL5 | 2.71 |  | EPS15L2 | 4.28 |  | MAEA | 2.74 |  | SCAPER | 2.64 |
| AGGF1 | 2.96 |  | EPS15L2 | 2.62 |  | MAF | 3.41 |  | SCAPER | 2.63 |
| AGPAT1 | 2.73 |  | EPS8L1 | 2.7 |  | MAFK | 3.55 |  | SCGB2A2 | 4.09 |
| AGRN | 3.23 |  | EPS8L1 | 2.54 |  | MAGEB1 | 2.77 |  | SCGB2A2 | 2.66 |
| AGXT | 4.05 |  | EPS8L1 | 2.57 |  | MAML3 | 2.61 |  | SCN1B | 3.2 |
| AGXT | 3.05 |  | ERAF | 2.75 |  | MAN2A1 | 2.99 |  | SCNM1 | 2.5 |
| AHCYL1 | 2.63 |  | ERAP1 | 2.77 |  | MAN2A1 | 3.03 |  | SCNM1 | 3.07 |
| AHDC1 | 2.58 |  | ERAP1 | 2.69 |  | MAP2K7 | 2.79 |  | SCNM1 | 3.24 |
| AHDC1 | 2.69 |  | ERBB2IP | 2.91 |  | MAP2K7 | 3.2 |  | SCNN1A | 2.56 |
| AICDA | 2.98 |  | ERBB2IP | 3.05 |  | MAP3K13 | 2.92 |  | SCNN1A | 3.42 |
| AIM1L | 2.76 |  | ERLIN1 | 3.63 |  | MAP3K5 | 2.81 |  | SCNN1D | 2.53 |
| AIM1L | 2.75 |  | ERN1 | 2.63 |  | MAP4 | 3.04 |  | SCNN1D | 3.45 |
| AIM1L | 3.48 |  | ESD | 2.69 |  | MAP7 | 2.69 |  | SCNN1D | 3.21 |
| AIM1L | 2.66 |  | ESR2 | 2.5 |  | MAP7 | 2.61 |  | SCP2 | 2.68 |
| AIM1L | 3.59 |  | ESRRA | 2.72 |  | MAPK11 | 2.68 |  | SCRIB | 2.57 |
| AIM2 | 2.61 |  | ESRRB | 2.81 |  | MAPK8IP1 | 2.52 |  | SCTR | 2.69 |
| AK1 | 2.54 |  | ETF1 | 3.29 |  | MAPK8IP3 | 2.94 |  | SCTR | 2.78 |
| AK2 | 2.55 |  | ETF1 | 2.58 |  | MAPK8IP3 | 2.75 |  | SCTR | 2.81 |
| AK2 | 2.54 |  | ETF1 | 2.95 |  | MARK4 | 2.72 |  | SCYL3 | 2.52 |
| AK2 | 2.93 |  | ETF1 | 2.88 |  | MAST1 | 2.57 |  | SDCBP | 2.75 |
| AKAP8L | 4.47 |  | ETF1 | 2.6 |  | MAST2 | 3.26 |  | SDCBP | 2.51 |
| AKAP8L | 2.73 |  | ETS1 | 2.94 |  | MAST2 | 3.7 |  | SDCBP | 3.42 |
| AKAP8L | 2.99 |  | EVI5 | 2.57 |  | MAT2A | 3.19 |  | SDCCAG1 | 2.58 |
| AKAP9 | 2.54 |  | EVI5 | 3.11 |  | MATN3 | 3.01 |  | SEC11A | 2.5 |
| ALDH1B1 | 2.76 |  | EVI5 | 2.61 |  | MATN3 | 3.97 |  | SEC16A | 2.54 |
| ALDH1L1 | 2.77 |  | EWSR1 | 2.55 |  | MAX | 2.54 |  | SEC22B | 3.59 |
| ALDH1L1 | 2.98 |  | EXOC7 | 2.9 |  | MAX | 3.41 |  | SEC22B | 2.68 |
| ALDH1L1 | 2.51 |  | EXOC7 | 2.61 |  | MCAM | 2.69 |  | SEC23B | 2.59 |
| ALDH3B2 | 2.79 |  | EXOC7 | 2.63 |  | MCAT | 2.55 |  | SEC23B | 3.61 |
| ALDH3B2 | 3.1 |  | EXOSC4 | 4.08 |  | MCF2L2 | 3.29 |  | SEC23IP | 3.41 |
| ALDH9A1 | 2.57 |  | EXOSC4 | 3.6 |  | MCM10 | 3.05 |  | SEC63 | 3.23 |
| ALDOB | 3.12 |  | EXOSC4 | 2.74 |  | MCRS1 | 2.69 |  | SEMA3A | 2.86 |
| ALG6 | 3.74 |  | EXTL1 | 2.66 |  | MECP2 | 3.15 |  | SEMA3B | 2.83 |
| ALKBH1 | 2.51 |  | EYA3 | 2.69 |  | MECP2 | 3.44 |  | SEMA3B | 3.22 |
| ALPI | 2.94 |  | F8A1 | 3.4 |  | MECR | 2.62 |  | SEMA3B | 2.58 |
| ALS2CL | 2.83 |  | F8A1 | 2.53 |  | MED16 | 2.53 |  | SEMA3F | 2.79 |
| ALS2CL | 2.8 |  | FADS3 | 2.56 |  | MED16 | 2.74 |  | SEMA4C | 2.69 |
| ALS2CL | 3.13 |  | FAF2 | 2.53 |  | MED25 | 3 |  | SEMA4C | 2.82 |
| ALX3 | 2.57 |  | FAIM2 | 3.48 |  | MEF2B | 2.7 |  | SEMG1 | 2.92 |
| ALX4 | 2.89 |  | FAIM2 | 2.75 |  | MEFV | 2.79 |  | SENP3 | 2.83 |
| ALX4 | 3.04 |  | FAM120A | 3.68 |  | MEFV | 2.9 |  | SENP5 | 3.37 |
| ALX4 | 4.28 |  | FAM120A | 2.84 |  | MEGF6 | 2.69 |  | SENP7 | 2.74 |
| AMACR | 2.56 |  | FAM120C | 2.61 |  | MEGF6 | 2.83 |  | SEPHS2 | 2.67 |
| AMACR | 3.41 |  | FAM120C | 3.08 |  | MEGF8 | 2.81 |  | 7-Sep | 3.1 |
| AMACR | 2.7 |  | FAM125B | 3.22 |  | MEGF8 | 3.16 |  | SEPX1 | 2.52 |
| AMBP | 2.52 |  | FAM12A | 2.76 |  | MEGF8 | 2.95 |  | SERBP1 | 2.94 |
| AMD1 | 2.51 |  | FAM131B | 2.83 |  | MEOX2 | 2.63 |  | SERINC3 | 2.68 |
| ANAPC2 | 2.66 |  | FAM131B | 2.57 |  | MEOX2 | 3.26 |  | SERINC3 | 2.56 |
| ANGPT2 | 2.62 |  | FAM135A | 2.75 |  | MERTK | 2.75 |  | SERP1 | 3.3 |
| ANGPT2 | 3.43 |  | FAM136A | 3.61 |  | MET | 2.57 |  | SERPINA4 | 2.8 |
| ANGPTL2 | 3.2 |  | FAM152A | 2.76 |  | METTL1 | 2.7 |  | SERPINA4 | 3.73 |
| ANGPTL7 | 2.91 |  | FAM158A | 3.07 |  | MFAP4 | 2.53 |  | SERPINA6 | 2.5 |
| ANGPTL7 | 2.64 |  | FAM158A | 2.55 |  | MFAP5 | 2.67 |  | SERPINA6 | 2.68 |
| ANK1 | 3.66 |  | FAM176B | 2.67 |  | MFAP5 | 2.61 |  | SERPINB1 | 2.75 |
| ANK3 | 2.63 |  | FAM176B | 2.91 |  | MFAP5 | 2.52 |  | SERPINB1 | 2.87 |
| ANKRA2 | 2.51 |  | FAM182B | 2.85 |  | MFI2 | 2.96 |  | SERPINB1 | 3.92 |
| ANKRA2 | 3.77 |  | FAM38B | 2.73 |  | MFSD1 | 2.95 |  | SERPINC1 | 2.62 |
| ANKZF1 | 2.79 |  | FAM3C | 3.04 |  | MFSD1 | 2.8 |  | SERPINC1 | 2.75 |
| ANO10 | 2.57 |  | FAM45A | 3.13 |  | MGAT2 | 2.83 |  | SETDB1 | 2.62 |
| ANO3 | 3.16 |  | FAM45A | 2.81 |  | MGC3771 | 2.81 |  | SETDB1 | 2.68 |
| ANP32B | 2.51 |  | FAM45A | 2.64 |  | MGC4771 | 3.03 |  | SEZ6L | 2.82 |
| ANP32E | 4.16 |  | FAM75A1 | 2.74 |  | MGC4771 | 2.89 |  | SEZ6L | 3.01 |
| ANP32E | 2.77 |  | FAM76A | 3.8 |  | MGC5370 | 3.23 |  | SF3B1 | 3 |
| ANP32E | 2.67 |  | FAM76A | 2.54 |  | MIS12 | 2.53 |  | SF3B1 | 2.64 |
| ANXA7 | 2.58 |  | FAM76A | 3.27 |  | MKL1 | 2.61 |  | SF3B1 | 2.84 |
| AOX1 | 2.69 |  | FAM86C | 2.67 |  | MKRN1 | 2.79 |  | SF3B4 | 3.05 |
| AP1S1 | 3.44 |  | FAM86C | 3.56 |  | MLANA | 3.04 |  | SF3B4 | 2.8 |
| AP1S1 | 3.42 |  | FAM86C | 3.16 |  | MLL2 | 3.98 |  | SFI1 | 2.77 |
| AP2A2 | 2.78 |  | FANCL | 2.57 |  | MLL2 | 2.79 |  | SFI1 | 2.53 |
| AP2B1 | 3.5 |  | FARP2 | 2.54 |  | MLL2 | 2.87 |  | SFI1 | 3.39 |
| AP3D1 | 2.96 |  | FASN | 3.41 |  | MLL2 | 3.36 |  | SFI1 | 2.86 |
| AP3S2 | 2.53 |  | FASN | 3.56 |  | MLL4 | 3.6 |  | SFI1 | 3.26 |
| AP4M1 | 3.33 |  | FASTKD2 | 3.22 |  | MLLT10 | 2.91 |  | SFRP1 | 2.71 |
| AP4M1 | 2.73 |  | FBLN1 | 2.8 |  | MLN | 3.28 |  | SFRP1 | 3.51 |
| AP4M1 | 4.07 |  | FBLN1 | 2.66 |  | MMP11 | 3.52 |  | SFRS10 | 2.93 |
| APBA3 | 3.05 |  | FBLN1 | 2.54 |  | MMP11 | 2.55 |  | SFRS10 | 3.04 |
| APC2 | 3.1 |  | FBP2 | 2.86 |  | MMP12 | 2.58 |  | SFRS10 | 3.8 |
| APC2 | 3.15 |  | FBP2 | 4.22 |  | MMP15 | 2.5 |  | SFRS11 | 3.01 |
| APC2 | 3.27 |  | FBXL18 | 2.68 |  | MMP17 | 4.38 |  | SFRS14 | 2.68 |
| APC2 | 2.56 |  | FBXO11 | 2.78 |  | MMP17 | 3.42 |  | SFRS14 | 2.57 |
| APC2 | 3.42 |  | FBXO17 | 2.77 |  | MMP17 | 4.2 |  | SFRS16 | 2.79 |
| APCS | 3.88 |  | FBXO2 | 2.63 |  | MMP24 | 2.53 |  | SFRS9 | 2.8 |
| API5 | 3.09 |  | FBXO2 | 2.83 |  | MMP24 | 2.76 |  | SGCA | 2.73 |
| API5 | 2.52 |  | FBXO21 | 2.52 |  | MMP24 | 2.58 |  | SGCB | 2.95 |
| APOA1 | 2.58 |  | FBXO42 | 2.56 |  | MNX1 | 2.56 |  | SGCB | 2.68 |
| APOA1 | 2.9 |  | FBXO5 | 2.63 |  | MOBKL1B | 2.51 |  | SGCD | 2.6 |
| APOA1 | 3.47 |  | FBXO9 | 2.73 |  | MOGAT2 | 2.71 |  | SGCD | 2.81 |
| APOA1 | 3.06 |  | FBXO9 | 3.26 |  | MORF4L1 | 2.64 |  | SH3BGRL | 2.53 |
| APOA1 | 3.8 |  | FBXW4P1 | 3.9 |  | MORN1 | 2.66 |  | SH3BGRL | 2.85 |
| APOA2 | 3.93 |  | FBXW4P1 | 3.2 |  | MORN1 | 2.65 |  | SH3BP4 | 2.52 |
| APOA2 | 2.61 |  | FBXW4P1 | 3.43 |  | MORN1 | 2.99 |  | SH3GL1 | 2.79 |
| APOB | 2.7 |  | FCAR | 3.39 |  | MOSC2 | 2.82 |  | SH3GL1 | 2.76 |
| AQP4 | 2.63 |  | FCAR | 2.52 |  | MPHOSPH9 | 2.62 |  | SH3GL3 | 2.58 |
| AQP4 | 3.23 |  | FCAR | 2.5 |  | MPP6 | 3.13 |  | SH3GLB2 | 2.64 |
| AQP5 | 2.74 |  | FCGR2C | 2.77 |  | MR1 | 2.51 |  | SH3PXD2A | 2.67 |
| AQP6 | 2.56 |  | FCGR2C | 2.64 |  | MRLC2 | 3.48 |  | SHANK1 | 3.67 |
| ARD1A | 4.57 |  | FER1L4 | 2.59 |  | MRM1 | 2.7 |  | SHB | 3.31 |
| ARD1A | 2.94 |  | FER1L4 | 2.78 |  | MRM1 | 3.01 |  | SHH | 2.99 |
| ARD1A | 2.56 |  | FERMT2 | 2.62 |  | MRM1 | 3.28 |  | SHH | 2.9 |
| AREG | 3.2 |  | FETUB | 2.57 |  | MRPL4 | 3.14 |  | SHH | 2.61 |
| AREG | 4.18 |  | FFAR2 | 2.7 |  | MRPL9 | 2.6 |  | SHMT2 | 2.6 |
| ARF3 | 3.06 |  | FGF12 | 2.69 |  | MRPS11 | 2.69 |  | SHMT2 | 3.11 |
| ARF3 | 2.94 |  | FGF12 | 2.6 |  | MRPS12 | 3.07 |  | SHOC2 | 2.67 |
| ARF5 | 2.72 |  | FGF12 | 2.71 |  | MRPS12 | 3.64 |  | SHOC2 | 2.6 |
| ARF6 | 2.52 |  | FGF12 | 2.81 |  | MRPS12 | 2.79 |  | SHOX2 | 2.51 |
| ARHGDIG | 3.12 |  | FGF2 | 3.22 |  | MRPS14 | 2.61 |  | SHOX2 | 2.82 |
| ARHGDIG | 2.52 |  | FGF20 | 3.12 |  | MRPS7 | 3.17 |  | SIAH1 | 2.91 |
| ARHGEF16 | 2.57 |  | FGF3 | 3.57 |  | MRTO4 | 2.51 |  | SIGLEC7 | 4.76 |
| ARHGEF16 | 2.78 |  | FGFR2 | 2.88 |  | MSC | 2.91 |  | SIGLEC7 | 2.59 |
| ARHGEF4 | 2.69 |  | FGFR4 | 3.2 |  | MSN | 2.76 |  | SIL1 | 2.51 |
| ARID4B | 2.52 |  | FGFR4 | 3.37 |  | MST1 | 2.82 |  | SIRT4 | 2.54 |
| ARL14 | 2.91 |  | FGFR4 | 2.89 |  | MST1 | 2.61 |  | SIRT4 | 2.7 |
| ARL4C | 2.75 |  | FH | 4.56 |  | MST1 | 2.57 |  | SIRT5 | 2.73 |
| ARL5A | 4.22 |  | FH | 2.83 |  | MST1 | 2.83 |  | SKI | 2.7 |
| ARL6IP5 | 2.8 |  | FICD | 3.18 |  | MST1R | 2.68 |  | SLC12A1 | 3.82 |
| ARL6IP5 | 3.25 |  | FIP1L1 | 2.97 |  | MSTN | 2.62 |  | SLC12A3 | 3.43 |
| ARL6IP5 | 2.71 |  | FKBP1B | 2.68 |  | MSTO1 | 3 |  | SLC12A3 | 2.55 |
| ARL8B | 2.86 |  | FKBP1B | 2.58 |  | MSTO1 | 3.04 |  | SLC12A4 | 3.76 |
| ARL8B | 2.6 |  | FKBP1B | 3.09 |  | MSTO1 | 2.6 |  | SLC12A4 | 3.4 |
| ARPC2 | 3.84 |  | FKBP8 | 2.74 |  | MT4 | 2.96 |  | SLC12A4 | 3.72 |
| ARPC2 | 3.36 |  | FKBP8 | 2.82 |  | MTERFD2 | 2.71 |  | SLC12A4 | 3.18 |
| ARPC2 | 3.7 |  | FKBP8 | 2.99 |  | MTF1 | 2.52 |  | SLC12A4 | 2.75 |
| ARPC3 | 3.46 |  | FKBPL | 2.73 |  | MTHFD2L | 2.78 |  | SLC12A4 | 3.54 |
| ARPC3 | 3 |  | FLCN | 2.56 |  | MTHFR | 3.27 |  | SLC13A4 | 2.68 |
| ARTN | 2.52 |  | FLJ10232 | 2.72 |  | MTHFR | 2.7 |  | SLC13A4 | 2.88 |
| ARTN | 2.54 |  | FLJ10232 | 3.12 |  | MTHFR | 2.72 |  | SLC16A8 | 3.08 |
| ARVCF | 3.38 |  | FLJ10232 | 2.75 |  | MTHFR | 3.38 |  | SLC16A8 | 3.67 |
| ASAH1 | 3.03 |  | FLJ10404 | 2.6 |  | MTMR3 | 2.89 |  | SLC16A8 | 2.87 |
| ASAH1 | 2.88 |  | FLJ11710 | 3.11 |  | MTNR1B | 2.79 |  | SLC19A1 | 2.9 |
| ASAH1 | 3.02 |  | FLJ11710 | 2.82 |  | MTNR1B | 2.59 |  | SLC1A2 | 2.61 |
| ASAH1 | 2.68 |  | FLJ14100 | 3.02 |  | MTRF1L | 3.27 |  | SLC22A14 | 2.78 |
| ASAH1 | 3.38 |  | FLJ14154 | 3.18 |  | MTSS1 | 2.95 |  | SLC22A14 | 3.17 |
| ASAH1 | 3.19 |  | FLJ20712 | 2.57 |  | MTSS1 | 3.02 |  | SLC22A18AS | 3.25 |
| ASCC1 | 3.04 |  | FLJ21767 | 3.01 |  | MUC4 | 2.78 |  | SLC22A6 | 2.92 |
| ASCL1 | 2.59 |  | FLJ21767 | 2.79 |  | MUC5AC | 2.78 |  | SLC22A6 | 3.07 |
| ASCL1 | 2.54 |  | FLJ22167 | 3 |  | MUC8 | 4.08 |  | SLC22A7 | 2.53 |
| ASMTL | 2.6 |  | FLJ22184 | 3.69 |  | MUC8 | 3 |  | SLC22A7 | 3.72 |
| ATF5 | 3.42 |  | FLJ22596 | 4.04 |  | MUC8 | 2.92 |  | SLC22A7 | 2.87 |
| ATF5 | 4.04 |  | FLJ22596 | 3.92 |  | MUPCDH | 3.53 |  | SLC22A7 | 5.11 |
| ATF5 | 3.3 |  | FLJ22596 | 3.79 |  | MUPCDH | 2.53 |  | SLC22A7 | 2.8 |
| ATF5 | 3.82 |  | FLJ42627 | 2.86 |  | MUT | 3.2 |  | SLC22A8 | 2.53 |
| ATN1 | 3.04 |  | FLT3LG | 2.85 |  | MVK | 2.59 |  | SLC24A1 | 2.64 |
| ATP11A | 2.74 |  | FLT3LG | 2.55 |  | MXD3 | 2.7 |  | SLC25A14 | 2.75 |
| ATP11A | 2.96 |  | FMR1 | 2.59 |  | MXD3 | 2.51 |  | SLC25A15 | 2.51 |
| ATP13A3 | 3.31 |  | FMR1 | 2.56 |  | MXD4 | 4.23 |  | SLC25A17 | 3.17 |
| ATP13A3 | 2.59 |  | FMR1 | 2.88 |  | MXD4 | 2.93 |  | SLC25A21 | 2.54 |
| ATP1B3 | 2.91 |  | FMR1 | 2.67 |  | MYH10 | 2.67 |  | SLC25A42 | 2.87 |
| ATP1B3 | 2.98 |  | FNDC4 | 2.93 |  | MYH10 | 2.66 |  | SLC26A1 | 2.75 |
| ATP1B4 | 3.22 |  | FOSL2 | 2.51 |  | MYH10 | 3.38 |  | SLC26A1 | 3.26 |
| ATP2B3 | 3.73 |  | FOXA2 | 3.15 |  | MYH11 | 2.65 |  | SLC26A1 | 2.88 |
| ATP2B3 | 2.71 |  | FOXA2 | 2.63 |  | MYH11 | 2.6 |  | SLC26A10 | 3.47 |
| ATP5F1 | 2.75 |  | FOXB1 | 2.6 |  | MYH13 | 3.41 |  | SLC26A6 | 2.73 |
| ATP6AP2 | 2.99 |  | FOXD3 | 2.86 |  | MYH13 | 2.68 |  | SLC27A5 | 3.07 |
| ATP6AP2 | 3.2 |  | FOXH1 | 2.61 |  | MYH13 | 2.51 |  | SLC27A5 | 3.13 |
| ATP6AP2 | 3.07 |  | FOXO1 | 2.54 |  | MYH14 | 3.73 |  | SLC27A5 | 3.16 |
| ATP6V0E1 | 3.49 |  | FOXO3 | 2.97 |  | MYH4 | 2.72 |  | SLC29A2 | 2.85 |
| ATP6V0E1 | 3.36 |  | FOXO3 | 4.33 |  | MYH6 | 2.67 |  | SLC2A8 | 3.4 |
| ATP6V0E1 | 2.97 |  | FREQ | 2.63 |  | MYH6 | 3.08 |  | SLC35D1 | 2.97 |
| ATP6V0E1 | 3.37 |  | FRMPD4 | 2.58 |  | MYH7 | 2.54 |  | SLC39A8 | 2.62 |
| ATP6V0E1 | 3.24 |  | FRYL | 2.64 |  | MYH7 | 2.62 |  | SLC4A10 | 3.37 |
| ATP6V0E1 | 2.57 |  | FRYL | 2.89 |  | MYH8 | 2.56 |  | SLC4A3 | 2.66 |
| ATP6V1A | 3.39 |  | FRYL | 3.02 |  | MYH8 | 2.55 |  | SLC4A3 | 2.81 |
| ATP6V1A | 3.07 |  | FSCN2 | 3.51 |  | MYH8 | 3.04 |  | SLC5A12 | 2.72 |
| ATP6V1A | 2.76 |  | FSD1 | 2.61 |  | MYH8 | 2.96 |  | SLC5A3 | 2.54 |
| ATP6V1B1 | 2.8 |  | FSD1 | 2.66 |  | MYL2 | 2.87 |  | SLC5A3 | 2.63 |
| ATP6V1B1 | 2.89 |  | FSD1 | 3.91 |  | MYL3 | 3.03 |  | SLC5A3 | 2.51 |
| ATP6V1B1 | 2.68 |  | FSTL3 | 2.93 |  | MYL3 | 2.95 |  | SLC7A1 | 3.33 |
| ATP6V1E1 | 2.6 |  | FTSJ1 | 2.77 |  | MYL3 | 3.89 |  | SLC7A1 | 2.92 |
| ATP8B4 | 2.86 |  | FTSJ2 | 2.89 |  | MYL4 | 2.84 |  | SLC7A4 | 3 |
| ATRX | 3.13 |  | FTSJ2 | 2.76 |  | MYL4 | 3.36 |  | SLC7A4 | 2.92 |
| ATRX | 4.68 |  | FTSJ2 | 3.57 |  | MYL4 | 3.85 |  | SLC7A4 | 2.85 |
| ATXN3 | 2.9 |  | FUT5 | 3.04 |  | MYL9 | 3.65 |  | SLC7A4 | 2.77 |
| ATXN7 | 3 |  | FUT5 | 2.9 |  | MYL9 | 3 |  | SLC7A5 | 2.84 |
| AVPR1A | 2.97 |  | FUT5 | 3.66 |  | MYL9 | 3.21 |  | SLIT1 | 2.65 |
| AVPR1A | 2.99 |  | FUT7 | 2.91 |  | MYLK3 | 2.55 |  | SLIT3 | 2.51 |
| AVPR1B | 2.83 |  | FUT9 | 3.49 |  | MYO10 | 2.85 |  | SLTM | 2.84 |
| AVPR1B | 2.92 |  | FYB | 2.75 |  | MYO19 | 3.22 |  | SLURP1 | 3.69 |
| AVPR1B | 2.78 |  | FYCO1 | 2.54 |  | MYO19 | 2.83 |  | SLURP1 | 2.75 |
| AVPR2 | 2.59 |  | FZD2 | 2.61 |  | MYO19 | 3.18 |  | SMAD2 | 2.85 |
| AZGP1P1 | 3.86 |  | FZD2 | 2.5 |  | MYO1A | 3.18 |  | SMAD3 | 2.64 |
| AZGP1P1 | 2.89 |  | FZD8 | 3 |  | MYO7B | 3.28 |  | SMAD5OS | 2.7 |
| AZI1 | 2.57 |  | FZD8 | 3.89 |  | MYO7B | 4.55 |  | SMAD5OS | 2.58 |
| AZI1 | 2.78 |  | FZD8 | 3.24 |  | MYO7B | 3.52 |  | SMAP1 | 2.7 |
| AZI1 | 3.2 |  | FZR1 | 2.75 |  | MYOZ2 | 2.51 |  | SMARCA2 | 3.46 |
| B2M | 2.74 |  | FZR1 | 3.16 |  | MYST2 | 3.34 |  | SMARCA4 | 3.28 |
| B3GALT2 | 2.96 |  | FZR1 | 3.28 |  | MYST4 | 3.35 |  | SMARCA4 | 3.13 |
| B3GALT2 | 2.62 |  | FZR1 | 2.79 |  | MYST4 | 2.62 |  | SMARCA4 | 2.72 |
| B3GNT4 | 2.92 |  | G3BP1 | 3 |  | MYT1 | 2.73 |  | SMARCC1 | 2.84 |
| B3GNT4 | 2.73 |  | G3BP1 | 2.72 |  | MZF1 | 2.65 |  | SMC5 | 2.7 |
| B3GNT4 | 3.89 |  | G3BP2 | 3.02 |  | MZF1 | 2.56 |  | SMG6 | 2.97 |
| B4GALT1 | 2.51 |  | G3BP2 | 2.59 |  | MZF1 | 2.55 |  | SMG6 | 2.95 |
| B4GALT6 | 2.78 |  | G3BP2 | 3.71 |  | N4BP2L2 | 3.69 |  | SMTN | 2.71 |
| BACH2 | 2.77 |  | G6PC | 2.59 |  | N4BP2L2 | 3 |  | SNAP23 | 3.67 |
| BAD | 2.6 |  | GABARAP | 2.52 |  | N4BP2L2 | 3.03 |  | SNAP23 | 3.23 |
| BAD | 2.92 |  | GABBR2 | 2.88 |  | NAALAD2 | 2.63 |  | SNAP23 | 3.52 |
| BAI1 | 2.96 |  | GABRA5 | 2.54 |  | NAB1 | 2.53 |  | SNAP25 | 2.53 |
| BAI1 | 3.09 |  | GAD2 | 2.58 |  | NACA2 | 3.77 |  | SNAP25 | 2.51 |
| BAI2 | 2.6 |  | GADD45GIP1 | 3.38 |  | NACA2 | 2.76 |  | SNAPC4 | 3.05 |
| BAIAP2 | 2.64 |  | GADD45GIP1 | 3.4 |  | NACAD | 3.38 |  | SNAPC4 | 3.11 |
| BAIAP3 | 2.71 |  | GADD45GIP1 | 2.96 |  | NACAD | 3.62 |  | SNAPC4 | 3.07 |
| BAIAP3 | 2.87 |  | GADD45GIP1 | 2.94 |  | NACAD | 4.07 |  | SNCA | 3.36 |
| BARX1 | 2.88 |  | GAL | 3.28 |  | NADK | 3.56 |  | SNCA | 2.86 |
| BAT1 | 2.66 |  | GAL | 3.85 |  | NADK | 2.71 |  | SNCA | 4.11 |
| BAT2D1 | 3.08 |  | GAL | 2.71 |  | NADK | 3.5 |  | SNCG | 4.56 |
| BAT2D1 | 2.74 |  | GALK2 | 4.24 |  | NADSYN1 | 2.54 |  | SNCG | 3.59 |
| BAT2D1 | 2.53 |  | GALK2 | 2.77 |  | NAE1 | 2.56 |  | SNCG | 3.19 |
| BAT3 | 2.8 |  | GALNT1 | 2.54 |  | NAG18 | 3.64 |  | SNPH | 3.27 |
| BBS7 | 2.59 |  | GALNT1 | 2.84 |  | NAP1L1 | 2.54 |  | SNRPB2 | 2.82 |
| BCAT2 | 3.4 |  | GALNT1 | 3.23 |  | NAP1L1 | 3.23 |  | SNRPB2 | 2.51 |
| BCAT2 | 3.06 |  | GALNT14 | 2.72 |  | NARS | 3.35 |  | SNTB2 | 2.53 |
| BCL10 | 2.96 |  | GALNT14 | 2.68 |  | NAT13 | 2.97 |  | SNTB2 | 2.56 |
| BCL10 | 2.78 |  | GALR2 | 3.67 |  | NAT13 | 3.21 |  | SNTB2 | 4.05 |
| BCL10 | 2.8 |  | GAMT | 3.26 |  | NAT13 | 3.1 |  | SNX13 | 2.88 |
| BCL2 | 2.65 |  | GAMT | 4.23 |  | NAT8 | 2.53 |  | SNX13 | 2.65 |
| BCL2 | 2.81 |  | GAMT | 3.38 |  | NAT8 | 2.83 |  | SNX3 | 2.54 |
| BCL2 | 2.54 |  | GAP43 | 2.8 |  | NAT8 | 2.6 |  | SNX3 | 2.74 |
| BCL2L10 | 5.05 |  | GAP43 | 3.56 |  | NAT8 | 2.93 |  | SNX3 | 2.84 |
| BCL2L10 | 3.9 |  | GAP43 | 3.3 |  | NAT8 | 3.3 |  | SOCS1 | 2.97 |
| BCL2L10 | 3.82 |  | GART | 2.54 |  | NAT8B | 3.18 |  | SOD2 | 2.58 |
| BCLAF1 | 3.1 |  | GART | 2.52 |  | NAT8B | 2.67 |  | SON | 3.03 |
| BECN1 | 2.84 |  | GART | 2.87 |  | NAV3 | 2.51 |  | SORBS1 | 2.71 |
| BFAR | 2.52 |  | GART | 3.29 |  | NBEA | 2.72 |  | SORL1 | 3.02 |
| BGLAP | 4.62 |  | GAS1 | 4.02 |  | NBEAL2 | 2.71 |  | SOS1 | 2.58 |
| BGLAP | 2.79 |  | GAS1 | 2.84 |  | NBLA00301 | 3.36 |  | SOX15 | 2.5 |
| BLK | 2.88 |  | GAS1 | 2.7 |  | NBPF10 | 2.65 |  | SOX17 | 2.61 |
| BLK | 3.13 |  | GAST | 2.65 |  | NCAM1 | 2.69 |  | SOX18 | 3.58 |
| BLK | 3.23 |  | GAST | 2.59 |  | NCAM1 | 3.73 |  | SOX18 | 2.57 |
| BLVRA | 2.67 |  | GAST | 2.64 |  | NCAPH2 | 3.13 |  | SOX4 | 3.5 |
| BLVRB | 2.59 |  | GATA2 | 2.64 |  | NCAPH2 | 3.07 |  | SOX4 | 3.04 |
| BMI1 | 3.37 |  | GBL | 2.77 |  | NCAPH2 | 3.04 |  | SOX4 | 3.69 |
| BMP1 | 3.88 |  | GBL | 3.82 |  | NCAPH2 | 3.42 |  | SP100 | 2.53 |
| BMP1 | 2.68 |  | GBX1 | 3.51 |  | NCAPH2 | 3.48 |  | SP100 | 2.71 |
| BMP4 | 3.16 |  | GCAT | 3.03 |  | NCDN | 2.78 |  | SPAG11A | 2.55 |
| BMP4 | 2.71 |  | GCDH | 2.7 |  | NCK1 | 2.55 |  | SPAG11A | 3.19 |
| BMP4 | 2.57 |  | GCGR | 3.58 |  | NCK1 | 2.51 |  | SPAG16 | 2.69 |
| BMP7 | 2.59 |  | GCGR | 4.38 |  | NCKIPSD | 2.98 |  | SPANXA1 | 3.33 |
| BMP8A | 3.56 |  | GCGR | 3.44 |  | NCKIPSD | 2.75 |  | SPANXC | 2.83 |
| BOP1 | 3.23 |  | GCK | 2.51 |  | NCKIPSD | 3.56 |  | SPAST | 2.56 |
| BPNT1 | 3.7 |  | GCK | 3.21 |  | NCOR2 | 3.82 |  | SPATA1 | 2.67 |
| BPNT1 | 2.72 |  | GCNT2 | 2.7 |  | NCOR2 | 3.02 |  | SPATA5L1 | 2.6 |
| BPNT1 | 2.54 |  | GDAP2 | 2.56 |  | NCOR2 | 4.16 |  | SPDEF | 2.51 |
| BRCA1 | 3.37 |  | GDF1 | 2.54 |  | NDFIP1 | 4.66 |  | SPDEF | 2.65 |
| BRF2 | 2.66 |  | GDF10 | 3.11 |  | NDFIP1 | 3.01 |  | SPHK2 | 2.8 |
| BTG1 | 3.03 |  | GDF3 | 2.51 |  | NDST1 | 2.6 |  | SPHK2 | 2.96 |
| BTG1 | 3.17 |  | GDI2 | 2.83 |  | NDUFA4L2 | 2.62 |  | SPHK2 | 2.77 |
| BTG1 | 3.09 |  | GDI2 | 2.63 |  | NDUFA4L2 | 2.56 |  | SPPL2B | 2.91 |
| BTN3A2 | 2.92 |  | GDNF | 2.85 |  | NDUFA5 | 2.57 |  | SPPL2B | 2.62 |
| BTN3A2 | 3.53 |  | GDNF | 2.69 |  | NDUFA6 | 2.92 |  | SPR | 2.67 |
| BTRC | 3.31 |  | GDPD5 | 2.63 |  | NDUFS5 | 2.55 |  | SPRR1A | 2.75 |
| BTRC | 2.9 |  | GEMIN7 | 3.31 |  | NDUFS8 | 2.58 |  | SPRR1B | 2.58 |
| BZRPL1 | 3.28 |  | GEMIN7 | 3.23 |  | NEBL | 2.61 |  | SPTB | 2.68 |
| BZRPL1 | 2.65 |  | GFI1B | 2.81 |  | NEU3 | 3.32 |  | SPTB | 3.26 |
| BZW1 | 2.56 |  | GFI1B | 3.84 |  | NEU3 | 3.05 |  | SPTBN1 | 2.78 |
| BZW1 | 2.78 |  | GFI1B | 2.88 |  | NEU3 | 3.56 |  | SPTBN1 | 3 |
| C10orf10 | 2.83 |  | GGA1 | 2.7 |  | NEU3 | 3.66 |  | SPTBN1 | 3.18 |
| C10orf10 | 3.4 |  | GGA3 | 3.39 |  | NEURL | 3.27 |  | SPTLC1 | 2.97 |
| C10orf10 | 3.21 |  | GGCX | 2.73 |  | NEUROG2 | 2.52 |  | SPTLC1 | 2.84 |
| C10orf12 | 2.54 |  | GGT1 | 2.95 |  | NEUROG3 | 2.94 |  | SPTLC1 | 2.95 |
| C10orf137 | 2.56 |  | GGT1 | 2.51 |  | NF1 | 2.55 |  | SPTLC2 | 2.81 |
| C10orf137 | 3.19 |  | GGT1 | 3.92 |  | NF1 | 2.84 |  | SREBF2 | 2.5 |
| C10orf81 | 3.05 |  | GGT1 | 3.93 |  | NFATC2IP | 2.68 |  | SRGAP2 | 2.66 |
| C11orf10 | 2.63 |  | GGT1 | 2.73 |  | NFATC2IP | 2.89 |  | SRP19 | 2.52 |
| C11orf57 | 2.94 |  | GGT1 | 2.65 |  | NFE2L2 | 2.61 |  | SRPK1 | 2.57 |
| C11orf9 | 3.43 |  | GGT1 | 2.92 |  | NFIB | 2.65 |  | SSH3 | 2.97 |
| C11orf9 | 3.25 |  | GGT1 | 2.58 |  | NFIX | 3.1 |  | SSR1 | 2.57 |
| C11orf9 | 3.18 |  | GH2 | 2.64 |  | NFKBIB | 2.5 |  | SSTR1 | 2.97 |
| C12orf32 | 2.81 |  | GIF | 3.31 |  | NFKBIB | 3.12 |  | SSX1 | 2.79 |
| C12orf32 | 3.36 |  | GIMAP5 | 2.53 |  | NFKBIL1 | 2.63 |  | SSX4 | 2.51 |
| C12orf32 | 3.67 |  | GJA3 | 2.79 |  | NFKBIL1 | 2.84 |  | SSX5 | 3.28 |
| C14orf1 | 2.85 |  | GJA3 | 3.94 |  | NKX2-1 | 3.1 |  | SSX5 | 3.07 |
| C14orf1 | 3.09 |  | GJA3 | 2.68 |  | NKX2-5 | 4.08 |  | SSX5 | 3.87 |
| C14orf108 | 2.63 |  | GJC2 | 2.8 |  | NKX2-5 | 2.77 |  | ST13 | 2.65 |
| C14orf108 | 2.6 |  | GKN1 | 3.91 |  | NKX2-8 | 2.64 |  | ST13 | 2.79 |
| C14orf169 | 2.52 |  | GLI3 | 2.59 |  | NKX2-8 | 3.31 |  | ST14 | 2.73 |
| C14orf172 | 2.58 |  | GLRA3 | 3.36 |  | NKX3-2 | 2.68 |  | ST14 | 3.64 |
| C14orf172 | 2.71 |  | GLRA3 | 2.51 |  | NKX3-2 | 2.81 |  | ST3GAL6 | 4.23 |
| C14orf65 | 2.88 |  | GLS | 2.53 |  | NKX3-2 | 2.97 |  | STAG2 | 3.02 |
| C15orf24 | 2.6 |  | GLS | 2.57 |  | NLGN1 | 2.68 |  | STAG2 | 2.91 |
| C15orf24 | 2.91 |  | GLTSCR1 | 3.26 |  | NLGN3 | 2.53 |  | STAG2 | 3.11 |
| C15orf34 | 3.59 |  | GLUL | 3.9 |  | NMT2 | 2.76 |  | STAM | 2.65 |
| C15orf39 | 2.72 |  | GNAI3 | 2.72 |  | NMUR1 | 3.01 |  | STARD3 | 2.72 |
| C16orf42 | 3.71 |  | GNAI3 | 3.18 |  | NMUR1 | 4.25 |  | STARD5 | 2.68 |
| C16orf42 | 3.65 |  | GNAZ | 2.87 |  | NMUR1 | 2.68 |  | STARD8 | 2.58 |
| C16orf45 | 2.58 |  | GNAZ | 2.84 |  | NOL1 | 2.91 |  | STAT1 | 2.58 |
| C16orf53 | 3.69 |  | GNAZ | 2.64 |  | NOL12 | 3.11 |  | STAT1 | 2.56 |
| C16orf58 | 2.51 |  | GNB1 | 2.54 |  | NOL3 | 3.92 |  | STAT1 | 2.66 |
| C16orf71 | 2.62 |  | GNB1L | 2.88 |  | NOL3 | 4.06 |  | STAT1 | 2.94 |
| C17orf70 | 2.58 |  | GNB3 | 3.85 |  | NOL3 | 2.56 |  | STAT3 | 2.76 |
| C17orf73 | 2.58 |  | GNB3 | 3.01 |  | NOL3 | 3 |  | STAT3 | 2.83 |
| C17orf88 | 3.26 |  | GNB3 | 2.59 |  | NOL3 | 2.78 |  | STK17A | 2.64 |
| C17orf88 | 2.77 |  | GNG10 | 3.01 |  | NOL3 | 4.63 |  | STK24 | 4.63 |
| C17orf90 | 2.61 |  | GNG10 | 2.76 |  | NOL6 | 2.53 |  | STK24 | 3.18 |
| C18orf1 | 3.54 |  | GNG7 | 2.72 |  | NOL6 | 2.75 |  | STK3 | 2.8 |
| C18orf22 | 2.68 |  | GNL3L | 2.65 |  | NOLA2 | 2.61 |  | STOML1 | 2.51 |
| C19orf26 | 2.6 |  | GNPDA1 | 2.55 |  | NONO | 2.63 |  | STRN4 | 2.9 |
| C19orf28 | 3.31 |  | GNPDA1 | 2.67 |  | NONO | 3.73 |  | STS | 3.08 |
| C19orf36 | 3.03 |  | GNRH1 | 3.51 |  | NOTCH2 | 2.8 |  | STX12 | 2.6 |
| C19orf36 | 2.61 |  | GNRH1 | 2.6 |  | NOTCH2 | 2.88 |  | STX17 | 2.59 |
| C19orf36 | 2.96 |  | GNRH1 | 2.55 |  | NOTCH2 | 2.64 |  | STX18 | 2.53 |
| C19orf40 | 2.69 |  | GNRH2 | 2.74 |  | NOTCH3 | 2.81 |  | STX2 | 2.84 |
| C19orf40 | 2.84 |  | GNRH2 | 3.42 |  | NOVA2 | 2.69 |  | STX6 | 2.68 |
| C1orf106 | 2.54 |  | GNRH2 | 3.07 |  | NOX1 | 2.54 |  | SUB1 | 4.62 |
| C1orf106 | 3.03 |  | GNS | 2.95 |  | NOX1 | 3.03 |  | SUB1 | 2.92 |
| C1orf165 | 2.58 |  | GNS | 3.42 |  | NOX3 | 2.83 |  | SUB1 | 3.48 |
| C1orf165 | 2.57 |  | GNS | 3.46 |  | NPAS1 | 3.34 |  | SUCLA2 | 2.79 |
| C1orf35 | 2.87 |  | GOLGA3 | 2.61 |  | NPAS2 | 2.9 |  | SUCLA2 | 3.13 |
| C1orf77 | 2.51 |  | GOLGA4 | 2.64 |  | NPAS2 | 3.02 |  | SUCLG2 | 2.86 |
| C1orf77 | 4.19 |  | GOLGA7 | 3.05 |  | NPAS2 | 3.13 |  | SUCLG2 | 3.19 |
| C1orf77 | 3.06 |  | GOLGA7 | 3.38 |  | NPAS2 | 3.92 |  | SUCLG2 | 3.79 |
| C1orf77 | 3.51 |  | GOLGA7 | 3.42 |  | NPC2 | 2.64 |  | SUMO1 | 2.67 |
| C1orf89 | 2.72 |  | GOLPH3 | 3.19 |  | NPFF | 2.95 |  | SUMO2 | 2.57 |
| C1orf89 | 3.09 |  | GORASP2 | 2.53 |  | NPM1 | 2.87 |  | SUPT16H | 2.84 |
| C1orf89 | 2.87 |  | GOSR2 | 2.72 |  | NPPC | 3.93 |  | SUPT16H | 3.11 |
| C1orf9 | 2.68 |  | GOT2 | 2.76 |  | NPPC | 3.18 |  | SUPT6H | 2.58 |
| C1QL1 | 3.12 |  | GP1BB | 2.55 |  | NPPC | 3.51 |  | SUPV3L1 | 2.74 |
| C1QL1 | 2.6 |  | GP9 | 3.08 |  | NPTN | 2.63 |  | SURF1 | 3.08 |
| C20orf117 | 2.72 |  | GP9 | 3.3 |  | NPTXR | 2.8 |  | SUV39H1 | 3.18 |
| C20orf24 | 2.76 |  | GP9 | 3.17 |  | NPTXR | 2.85 |  | SUV39H1 | 2.54 |
| C20orf3 | 2.57 |  | GPATCH1 | 2.53 |  | NPTXR | 3.03 |  | SUV39H1 | 2.83 |
| C20orf39 | 2.63 |  | GPATCH3 | 4.03 |  | NPY2R | 2.56 |  | SUZ12 | 2.71 |
| C20orf4 | 2.5 |  | GPC1 | 3.36 |  | NR1D1 | 3.16 |  | SUZ12 | 3.55 |
| C21orf2 | 2.7 |  | GPC1 | 3.54 |  | NR1D1 | 3.55 |  | SUZ12 | 3.88 |
| C21orf2 | 2.74 |  | GPC1 | 3.38 |  | NR2E3 | 2.74 |  | SYCP2 | 2.66 |
| C21orf2 | 3.4 |  | GPLD1 | 2.76 |  | NR2F2 | 3 |  | SYDE1 | 2.74 |
| C21orf2 | 2.87 |  | GPLD1 | 2.64 |  | NR2F2 | 2.57 |  | SYMPK | 3.01 |
| C21orf2 | 3.13 |  | GPR135 | 3.02 |  | NR2F6 | 2.85 |  | SYNJ2 | 3.59 |
| C2orf34 | 2.65 |  | GPR135 | 2.85 |  | NR4A1 | 3.11 |  | SYNJ2 | 2.98 |
| C3orf63 | 2.65 |  | GPR135 | 4.05 |  | NR6A1 | 3.27 |  | SYNJ2 | 4.43 |
| C5AR1 | 2.58 |  | GPR172B | 2.93 |  | NR6A1 | 2.84 |  | SYT12 | 3.07 |
| C5orf22 | 2.93 |  | GPR18 | 2.66 |  | NR6A1 | 2.67 |  | SYT12 | 3.91 |
| C5orf22 | 2.63 |  | GPR182 | 2.67 |  | NRXN3 | 2.55 |  | SYT12 | 2.96 |
| C5orf4 | 2.71 |  | GPR27 | 3.58 |  | NSBP1 | 2.56 |  | TACSTD1 | 2.61 |
| C5orf45 | 2.78 |  | GPR37 | 2.78 |  | NSF | 2.58 |  | TADA3L | 3.83 |
| C6orf108 | 3.17 |  | GPR37L1 | 3.17 |  | NSL1 | 3.21 |  | TADA3L | 2.81 |
| C6orf25 | 2.65 |  | GPR4 | 3.03 |  | NSL1 | 2.74 |  | TAF1C | 2.71 |
| C6orf26 | 2.65 |  | GPR4 | 3.18 |  | NSMAF | 2.79 |  | TAF1C | 3.06 |
| C6orf27 | 2.87 |  | GPR4 | 2.81 |  | NSMAF | 2.7 |  | TAF7 | 3.98 |
| C6orf27 | 2.53 |  | GPR44 | 2.72 |  | NSUN5 | 2.63 |  | TAF7L | 3.01 |
| C6orf27 | 3.38 |  | GPR44 | 2.68 |  | NSUN5 | 3.08 |  | TAF7L | 2.86 |
| C6orf54 | 3.62 |  | GPR44 | 4.6 |  | NT5C2 | 3.07 |  | TAF7L | 2.75 |
| C6orf54 | 4.2 |  | GPR6 | 2.6 |  | NTF3 | 2.85 |  | TAL1 | 2.72 |
| C7orf44 | 2.7 |  | GPR68 | 3.36 |  | NTN1 | 2.68 |  | TAL1 | 2.65 |
| C8G | 4.13 |  | GPR68 | 2.98 |  | NTN2L | 2.87 |  | TARBP1 | 2.6 |
| C8G | 2.81 |  | GPRC5C | 2.57 |  | NTRK1 | 2.88 |  | TAS2R4 | 2.71 |
| C9orf16 | 2.55 |  | GPS1 | 3.31 |  | NUDC | 3.11 |  | TAT | 2.7 |
| C9orf3 | 2.58 |  | GPT | 3.76 |  | NUDC | 2.58 |  | TAT | 2.92 |
| CA1 | 2.67 |  | GPT | 4.76 |  | NUP210 | 3.5 |  | TAX1BP3 | 3.24 |
| CA1 | 2.56 |  | GPT | 3.43 |  | NUP214 | 3.66 |  | TAZ | 2.98 |
| CA5BL | 2.51 |  | GRB10 | 2.5 |  | NUP214 | 3.35 |  | TBC1D1 | 2.55 |
| CACNA1A | 2.63 |  | GRB14 | 2.58 |  | NUP214 | 3.1 |  | TBC1D15 | 2.65 |
| CACNA1A | 3.75 |  | GRIA3 | 2.78 |  | OAZ1 | 2.89 |  | TBC1D2B | 2.63 |
| CACNA1A | 3.07 |  | GRID2 | 2.69 |  | OAZ1 | 2.92 |  | TBC1D2B | 2.88 |
| CACNA1A | 2.98 |  | GRIK1 | 3.21 |  | OAZ2 | 2.53 |  | TBC1D2B | 3.08 |
| CACNA1A | 2.94 |  | GRIK5 | 2.68 |  | OBP2A | 3.28 |  | TBC1D30 | 2.54 |
| CACNA1B | 2.51 |  | GRIK5 | 2.84 |  | OBP2A | 4.58 |  | TBC1D9B | 2.8 |
| CACNA1D | 3.41 |  | GRIK5 | 3.56 |  | OBP2A | 3.54 |  | TBC1D9B | 2.61 |
| CACNA1D | 3.92 |  | GRIN1 | 2.67 |  | OBSCN | 3.22 |  | TBL3 | 2.62 |
| CACNA1D | 3.61 |  | GRIN1 | 2.73 |  | OBSCN | 4.21 |  | NFIB | 2.65 |
| CACNA1G | 3.27 |  | GRIN1 | 3.07 |  | OBSCN | 3.88 |  | TBX2 | 3.17 |
| CACNA1G | 3.15 |  | GRIN1 | 4.18 |  | OBSL1 | 2.62 |  | FOXA2 | 3.15 |
| CACNA1G | 3.35 |  | GRIN1 | 3.35 |  | OBSL1 | 2.61 |  | NFKBIB | 3.12 |
| CACNA1G | 3.32 |  | GRIN1 | 3.95 |  | OBSL1 | 2.84 |  | PAX3 | 3.5 |
| CACNA1G | 2.85 |  | GRIN1 | 3.38 |  | ODF2 | 3.3 |  | PML | 3.32 |
| CACNA1G | 3.45 |  | GRIN1 | 2.75 |  | ODZ3 | 2.52 |  | STAT1 | 2.66 |
| CACNA1G | 2.61 |  | GRIN2D | 3.04 |  | OGFOD1 | 3.4 |  | STAT3 | 2.83 |
| CACNA1G | 2.9 |  | GRK4 | 2.58 |  | OGFOD1 | 2.97 |  | TBX2 | 3.8 |
| CACNA1H | 2.5 |  | GRK5 | 2.66 |  | OLA1 | 2.69 |  | HIF1A | 3.26 |
| CACNA1S | 3.22 |  | GRLF1 | 2.88 |  | OMP | 2.56 |  | NKX2-8 | 3.31 |
| CACNA1S | 2.52 |  | GRLF1 | 3.08 |  | OPRL1 | 2.55 |  | HSF1 | 3.3 |
| CACNA2D3 | 2.8 |  | GRLF1 | 2.75 |  | OPRL1 | 3.91 |  | TBX2 | 2.86 |
| CACNA2D3 | 2.62 |  | GRM1 | 3.57 |  | OPRS1 | 2.62 |  | PAX3 | 2.76 |
| CACNB1 | 2.5 |  | GRM2 | 3.64 |  | OR2F2 | 2.93 |  | FOXA2 | 2.63 |
| CACNB1 | 2.81 |  | GRM2 | 3.13 |  | OR2H1 | 3.56 |  | PAX3 | 2.98 |
| CACNB1 | 2.95 |  | GRM4 | 3.2 |  | ORC5L | 2.78 |  | STAT1 | 2.56 |
| CACNB2 | 2.62 |  | GRM8 | 2.5 |  | ORC5L | 3.77 |  | ETS1 | 2.94 |
| CACNG4 | 2.66 |  | GRSF1 | 3 |  | ORC5L | 2.51 |  | NOTCH2 | 2.88 |
| CACNG4 | 3.33 |  | GRTP1 | 2.66 |  | OSBP2 | 2.82 |  | GLI3 | 2.59 |
| CACNG4 | 3.31 |  | GSTT2 | 2.67 |  | OSBPL7 | 3.85 |  | TCF7L2 | 2.54 |
| CADM3 | 2.82 |  | GSTT2 | 3.49 |  | OVGP1 | 2.75 |  | PML | 3.63 |
| CADM3 | 3.01 |  | GTF2A1 | 2.56 |  | OVOL1 | 2.8 |  | MAF | 3.41 |
| CADM4 | 2.54 |  | GTF2E1 | 2.51 |  | OVOL1 | 3.67 |  | TP73 | 2.64 |
| CALCA | 2.58 |  | GTF2H1 | 2.8 |  | OVOL1 | 2.81 |  | CREBBP | 2.62 |
| CALCOCO2 | 2.73 |  | GTPBP1 | 2.98 |  | OXT | 3.34 |  | NPM1 | 2.87 |
| CALD1 | 2.8 |  | GTPBP1 | 4.44 |  | OXT | 2.53 |  | STAT1 | 2.94 |
| CALD1 | 3.78 |  | GTSE1 | 3.12 |  | OXT | 3.19 |  | TP73 | 2.79 |
| CALR | 3.11 |  | GUCA1A | 2.62 |  | P2RX2 | 3.61 |  | CREBBP | 2.54 |
| CALY | 2.74 |  | GUCA1A | 2.69 |  | P2RX2 | 2.95 |  | TBXA2R | 3.95 |
| CAMK2B | 4.11 |  | GUCA1B | 2.8 |  | P2RX2 | 2.81 |  | TBXA2R | 2.58 |
| CAMK2B | 3.28 |  | GUCA1B | 2.97 |  | P2RX6 | 3.2 |  | TCEB1P3 | 2.71 |
| CAMSAP1 | 3.1 |  | GUCA1B | 2.61 |  | P2RY11 | 2.83 |  | TCEB1P3 | 3.07 |
| CANX | 3.1 |  | GUCY2F | 2.68 |  | P2RY11 | 3.76 |  | TCEB3 | 2.52 |
| CAPN10 | 3.09 |  | GUK1 | 2.69 |  | P2RY6 | 4.38 |  | TCF21 | 2.93 |
| CAPN5 | 2.94 |  | GUK1 | 3.63 |  | P2RY6 | 2.77 |  | TCF21 | 2.63 |
| CAPN5 | 2.97 |  | GUK1 | 2.81 |  | PA2G4 | 2.57 |  | TCF3 | 2.54 |
| CAPN5 | 2.57 |  | GYPA | 2.52 |  | PAEP | 2.77 |  | TCF3 | 3.69 |
| CAPZA1 | 2.58 |  | GYPA | 2.81 |  | PAFAH1B3 | 4.39 |  | TCF3 | 3.26 |
| CAPZA1 | 2.79 |  | H2AFV | 3.06 |  | PAFAH1B3 | 3.04 |  | TCF3 | 2.94 |
| CAPZA1 | 2.83 |  | H2AFX | 2.55 |  | PAICS | 2.88 |  | TCF7L2 | 2.54 |
| CAPZB | 3.22 |  | H2AFY | 2.5 |  | PAICS | 2.64 |  | TCF7L2 | 2.86 |
| CAPZB | 4.22 |  | H2AFY | 2.54 |  | PALLD | 3.33 |  | TEF | 2.61 |
| CAPZB | 3.49 |  | H2AFZ | 2.74 |  | PALLD | 3.27 |  | TEF | 3.36 |
| CAPZB | 3.42 |  | H2AFZ | 2.67 |  | PALLD | 2.99 |  | TELO2 | 2.83 |
| CARD10 | 2.93 |  | H2AFZ | 2.94 |  | PALM | 3.02 |  | TELO2 | 4.04 |
| CARD10 | 3.08 |  | H3F3A | 3.02 |  | PALM | 3.38 |  | TELO2 | 3.06 |
| CASKIN2 | 3.07 |  | HADH | 3.06 |  | PALM | 2.95 |  | TELO2 | 3.39 |
| CASKIN2 | 2.63 |  | HAPLN1 | 2.83 |  | PAN2 | 3.6 |  | TELO2 | 3.21 |
| CASP2 | 3.57 |  | HAPLN2 | 3.16 |  | PAPOLA | 2.6 |  | TENC1 | 2.93 |
| CASP2 | 2.68 |  | HARS | 2.6 |  | PARD6A | 3.05 |  | TENC1 | 2.89 |
| CASP6 | 2.53 |  | HBE1 | 2.63 |  | PARD6A | 2.76 |  | TERF2 | 2.85 |
| CBFA2T2 | 3.5 |  | HBQ1 | 3.03 |  | PARD6A | 3.3 |  | TESK1 | 3.83 |
| CBFA2T2 | 2.73 |  | HBQ1 | 3.07 |  | PARVA | 2.51 |  | TEX10 | 3.04 |
| CBR4 | 2.61 |  | hCG_1757335 | 2.65 |  | PATZ1 | 2.9 |  | TEX12 | 2.55 |
| CBX7 | 2.7 |  | hCG_1757335 | 3.13 |  | PATZ1 | 2.97 |  | TEX261 | 2.77 |
| CBX8 | 3.31 |  | hCG_1757335 | 2.89 |  | PAWR | 2.91 |  | TEX28 | 3.09 |
| CBX8 | 4.3 |  | hCG_1774568 | 3.21 |  | PAX1 | 2.84 |  | TEX28 | 3.13 |
| CC2D1A | 2.74 |  | HCLS1 | 2.79 |  | PAX1 | 2.62 |  | TFAM | 3.23 |
| CC2D1A | 2.64 |  | HCLS1 | 2.93 |  | PAX3 | 2.98 |  | TFAM | 2.54 |
| CCBL2 | 2.66 |  | HCN2 | 3.44 |  | PAX3 | 3.5 |  | TFAP2C | 2.62 |
| CCDC102B | 2.8 |  | HCN2 | 3.49 |  | PAX3 | 2.76 |  | TFE3 | 3.03 |
| CCDC40 | 2.52 |  | HDAC11 | 2.68 |  | PAX8 | 3.48 |  | TFIP11 | 3.05 |
| CCDC40 | 2.9 |  | HDAC5 | 2.7 |  | PAX8 | 2.57 |  | TFPI | 2.64 |
| CCDC40 | 2.57 |  | HDAC6 | 3.54 |  | PAX8 | 2.58 |  | TFRC | 2.61 |
| CCDC47 | 3 |  | HDAC6 | 2.85 |  | PAX8 | 3.06 |  | TGFA | 3.67 |
| CCDC47 | 2.76 |  | HEATR6 | 2.55 |  | PAX8 | 3.45 |  | TGFB2 | 2.8 |
| CCDC64 | 2.61 |  | HECA | 2.64 |  | PAX8 | 2.89 |  | TGFBRAP1 | 3.97 |
| CCDC64 | 2.95 |  | HEMK1 | 3.03 |  | PBX1 | 2.83 |  | TGFBRAP1 | 2.6 |
| CCDC90B | 2.56 |  | HEMK1 | 2.71 |  | PCCA | 3.44 |  | TGM1 | 3.6 |
| CCDC90B | 2.77 |  | HFE | 3.59 |  | PCDH12 | 2.76 |  | TGM1 | 2.68 |
| CCDC94 | 2.67 |  | HFE | 3.29 |  | PCDH21 | 2.76 |  | TGM1 | 3.49 |
| CCDC94 | 2.73 |  | HFE | 3.12 |  | PCDH24 | 2.55 |  | TGOLN2 | 2.67 |
| CCK | 2.69 |  | HFE | 2.68 |  | PCDH24 | 3.08 |  | TGOLN2 | 3.2 |
| CCK | 3.4 |  | HGF | 2.57 |  | PCDH7 | 2.53 |  | TGOLN2 | 2.75 |
| CCL19 | 2.78 |  | HGF | 3.07 |  | PCDHA2 | 3.08 |  | TGS1 | 3.38 |
| CCL24 | 3.01 |  | HGFAC | 3.36 |  | PCDHB8 | 2.72 |  | THAP3 | 2.84 |
| CCNA2 | 2.61 |  | HGSNAT | 2.73 |  | PCDHGA10 | 3.94 |  | THAP3 | 2.93 |
| CCNE1 | 2.55 |  | HIC2 | 2.71 |  | PCDHGA11 | 2.52 |  | THAP3 | 2.91 |
| CCNE2 | 2.92 |  | HIF1A | 3.1 |  | PCDHGA11 | 2.66 |  | THAP7 | 2.64 |
| CCNI | 3.32 |  | HIF1A | 3.26 |  | PCDHGA3 | 3.61 |  | THAP9 | 2.79 |
| CCNI | 2.99 |  | HIF1A | 3.35 |  | PCDHGA3 | 2.7 |  | THBS3 | 3.63 |
| CCNI | 3.91 |  | HIF3A | 3.08 |  | PCDHGA3 | 3.42 |  | THBS3 | 3.84 |
| CCNT2 | 2.63 |  | HIF3A | 2.53 |  | PCDHGA3 | 2.86 |  | THBS3 | 2.96 |
| CCPG1 | 2.64 |  | HIF3A | 2.73 |  | PCDHGA3 | 4.47 |  | THEG | 2.6 |
| CCR7 | 2.57 |  | HIPK1 | 2.79 |  | PCDHGA3 | 2.83 |  | THOC6 | 3.02 |
| CCS | 2.51 |  | HIST1H1B | 2.87 |  | PCDHGA9 | 2.76 |  | THOC7 | 2.85 |
| CCT8 | 2.66 |  | HIST1H1B | 4.14 |  | PCGF2 | 2.65 |  | THOP1 | 2.86 |
| CCT8 | 2.75 |  | HIST1H1B | 2.78 |  | PCGF2 | 2.85 |  | THOP1 | 3.25 |
| CCT8 | 2.68 |  | HIST1H1E | 2.58 |  | PCGF2 | 2.69 |  | THRA | 2.69 |
| CD24 | 2.75 |  | HIST1H1E | 2.51 |  | PCIF1 | 3 |  | THUMPD1 | 2.82 |
| CD24 | 2.5 |  | HIST1H2AD | 4.01 |  | PCMT1 | 2.59 |  | TIA1 | 3.05 |
| CD4 | 3.67 |  | HIST1H2AD | 3.18 |  | PCNA | 2.69 |  | TIA1 | 2.69 |
| CD4 | 2.54 |  | HIST1H2AD | 2.59 |  | PCNA | 2.54 |  | TIMM13 | 2.58 |
| CD44 | 2.69 |  | HIST1H2AG | 3.08 |  | PCSK1N | 2.68 |  | TIMM44 | 2.55 |
| CD44 | 2.53 |  | HIST1H2AG | 3.51 |  | PCYT1B | 3.77 |  | TIMM44 | 3.22 |
| CD47 | 3.69 |  | HIST1H2AG | 2.88 |  | PCYT1B | 2.59 |  | TIMM8B | 2.53 |
| CD47 | 2.9 |  | HIST1H2BB | 2.56 |  | PCYT1B | 2.61 |  | TIMP1 | 2.56 |
| CD6 | 2.67 |  | HIST1H2BB | 2.75 |  | PDAP1 | 3.94 |  | TINAGL1 | 3.04 |
| CD63 | 2.5 |  | HIST1H2BK | 2.51 |  | PDCD1 | 2.54 |  | TINAGL1 | 2.89 |
| CD63 | 3.19 |  | HIST1H2BN | 2.51 |  | PDCD1 | 3.14 |  | TINF2 | 2.63 |
| CD70 | 2.71 |  | HIST1H2BO | 2.87 |  | PDCD10 | 2.65 |  | TIPRL | 2.55 |
| CD93 | 2.57 |  | HIST1H3F | 2.71 |  | PDCD6 | 2.7 |  | TLE4 | 2.56 |
| CDC25C | 3.93 |  | HIST1H3F | 2.8 |  | PDE4C | 3.04 |  | TLK1 | 2.59 |
| CDC2L6 | 2.61 |  | HIST1H3I | 2.88 |  | PDE6C | 2.7 |  | TLL1 | 3.18 |
| CDC2L6 | 2.5 |  | HIST1H4A | 2.51 |  | PDGFA | 3.2 |  | TLX2 | 3.57 |
| CDC2L6 | 3.08 |  | HIST1H4G | 3.05 |  | PDGFA | 2.65 |  | TLX2 | 2.87 |
| CDC2L6 | 4.34 |  | HIST1H4L | 3.05 |  | PDGFA | 2.86 |  | TM2D1 | 2.61 |
| CDC42 | 3.04 |  | HIST2H2AA3 | 2.78 |  | PDGFA | 2.82 |  | TM2D1 | 2.84 |
| CDC42SE1 | 3.05 |  | HJURP | 2.54 |  | PDHX | 2.57 |  | TM9SF3 | 3.43 |
| CDH15 | 2.57 |  | HKDC1 | 2.72 |  | PDIA6 | 2.56 |  | TM9SF4 | 2.57 |
| CDH2 | 2.81 |  | HLA-DMB | 2.53 |  | PDLIM1 | 2.54 |  | TMC5 | 2.93 |
| CDH22 | 2.87 |  | HLA-DOA | 3.25 |  | PDLIM7 | 3 |  | TMCO1 | 3.07 |
| CDH22 | 3.22 |  | HLA-DPA1 | 2.51 |  | PDS5A | 2.91 |  | TMED1 | 2.94 |
| CDH22 | 2.79 |  | HMG20B | 2.55 |  | PDS5B | 3.47 |  | TMED1 | 2.84 |
| CDIPT | 2.84 |  | HMG2L1 | 2.92 |  | PDS5B | 2.91 |  | TMED1 | 3.36 |
| CDIPT | 3.03 |  | HMGA1 | 2.52 |  | PDS5B | 2.6 |  | TMED10 | 2.82 |
| CDK2 | 3.87 |  | HMGN4 | 2.53 |  | PDZRN3 | 2.85 |  | TMED2 | 2.97 |
| CDK3 | 2.53 |  | HN1L | 2.59 |  | PEA15 | 2.99 |  | TMED7 | 2.61 |
| CDK3 | 4.12 |  | HN1L | 2.85 |  | PEA15 | 2.57 |  | TMEM123 | 2.8 |
| CDK5R2 | 2.53 |  | HNF1A | 2.61 |  | PELO | 3.43 |  | TMEM132A | 2.52 |
| CDK5R2 | 3.87 |  | HNF1A | 2.57 |  | PELO | 4.05 |  | TMEM143 | 2.95 |
| CDKL5 | 3.1 |  | HNF4A | 3.13 |  | PET112L | 3.98 |  | TMEM143 | 3.37 |
| CDKL5 | 2.73 |  | HNF4A | 3.85 |  | PEX16 | 3.41 |  | TMEM161A | 2.63 |
| CDKN1B | 3.19 |  | HNF4A | 4.92 |  | PEX19 | 2.52 |  | TMEM161A | 2.78 |
| CDKN1B | 2.92 |  | HNF4A | 2.96 |  | PEX19 | 2.56 |  | TMEM208 | 2.58 |
| CDKN1B | 2.58 |  | HNF4A | 2.75 |  | PEX6 | 3.59 |  | TMEM30A | 2.5 |
| CDKN1C | 2.74 |  | HNF4A | 3.32 |  | PEX6 | 3.16 |  | TMEM30A | 2.64 |
| CDKN1C | 3.22 |  | HNRNPA1 | 2.71 |  | PF4V1 | 3.36 |  | TMEM41B | 2.51 |
| CDKN2D | 2.59 |  | HNRNPA1 | 3.15 |  | PF4V1 | 3.09 |  | TMEM57 | 2.72 |
| CDR1 | 3.26 |  | HNRNPA3 | 2.83 |  | PFTK1 | 2.6 |  | TMEM66 | 3.35 |
| CDRT1 | 2.82 |  | HNRNPA3 | 2.62 |  | PGAM1 | 2.62 |  | TMPRSS5 | 2.59 |
| CDS2 | 2.51 |  | HNRNPA3 | 3.43 |  | PGAM1 | 2.94 |  | TMPRSS6 | 2.59 |
| CDS2 | 3.01 |  | HNRNPC | 2.77 |  | PGK1 | 3.18 |  | TMPRSS6 | 2.82 |
| CDX1 | 3.67 |  | HNRNPC | 2.52 |  | PGK1 | 2.92 |  | TNFAIP8 | 2.58 |
| CDX1 | 3.23 |  | HNRNPC | 3.61 |  | PGLYRP1 | 2.51 |  | TNFRSF25 | 2.83 |
| CDX1 | 3.12 |  | HNRNPH1 | 2.77 |  | PGLYRP1 | 2.83 |  | TNFRSF25 | 2.61 |
| CDX4 | 3.72 |  | HNRNPH1 | 2.64 |  | PGLYRP1 | 3.8 |  | TNFRSF25 | 2.76 |
| CDX4 | 3.15 |  | HNRNPH1 | 3.81 |  | PGP | 2.79 |  | TNFRSF9 | 2.55 |
| CDY1 | 2.77 |  | HNRNPH2 | 2.59 |  | PGP | 3.02 |  | TNK2 | 2.54 |
| CDYL | 3.08 |  | HNRNPR | 3.31 |  | PHEX | 2.74 |  | TNNC1 | 3.27 |
| CEACAM3 | 2.57 |  | HNRNPR | 4.19 |  | PHEX | 2.78 |  | TNNT1 | 3.28 |
| CEACAM4 | 2.99 |  | HNRNPR | 3.18 |  | PHKA2 | 3.48 |  | TNNT3 | 2.73 |
| CEACAM5 | 2.9 |  | HNRPDL | 2.71 |  | PHKB | 2.62 |  | TNP1 | 2.7 |
| CEACAM5 | 2.71 |  | HNT | 2.85 |  | PHKG1 | 2.92 |  | TNXB | 3.01 |
| CEACAM5 | 4.31 |  | HOMER3 | 3.53 |  | PHKG1 | 2.69 |  | TOM1 | 2.83 |
| CEACAM7 | 2.54 |  | HOMER3 | 4.82 |  | PHKG2 | 2.52 |  | TOM1L2 | 2.79 |
| CEBPE | 3.86 |  | HOMER3 | 3.11 |  | PHLPP | 3.61 |  | TOM1L2 | 3.66 |
| CELSR1 | 2.52 |  | HOXA11 | 3.39 |  | PHLPP | 2.91 |  | TOM1L2 | 2.97 |
| CEND1 | 3.39 |  | HOXB1 | 2.89 |  | PHOX2A | 3.35 |  | TOMM70A | 2.66 |
| CEND1 | 3.03 |  | HOXB1 | 3.28 |  | PHOX2A | 3.05 |  | TOP3B | 2.62 |
| CEND1 | 3.06 |  | HOXB1 | 3.27 |  | PHOX2A | 2.96 |  | TOP3B | 4.24 |
| CENPT | 2.5 |  | HOXB7 | 2.55 |  | PIB5PA | 3.62 |  | TOP3B | 3 |
| CENTB2 | 2.6 |  | HOXB7 | 4.13 |  | PIB5PA | 3.22 |  | TOP3B | 2.77 |
| CENTD1 | 2.71 |  | HOXC5 | 2.57 |  | PICALM | 3.48 |  | TOP3B | 3.68 |
| CENTD1 | 2.75 |  | HOXD12 | 2.67 |  | PICALM | 2.97 |  | TOX | 2.7 |
| CENTG1 | 2.67 |  | HOXD3 | 3.94 |  | PICK1 | 2.96 |  | TP73 | 2.64 |
| CEP152 | 2.91 |  | HOXD3 | 2.69 |  | PICK1 | 2.77 |  | TP73 | 3.07 |
| CEP164 | 2.75 |  | HPCAL4 | 2.62 |  | PICK1 | 2.78 |  | TP73 | 2.79 |
| CEP164 | 3.48 |  | HPR | 2.53 |  | PIGG | 2.64 |  | TPH1 | 2.5 |
| CEP170 | 2.54 |  | HPRT1 | 2.89 |  | PIK3C3 | 2.79 |  | TPM1 | 3.21 |
| CEP350 | 2.6 |  | HR44 | 2.61 |  | PIP4K2A | 2.63 |  | TPO | 3.31 |
| CFDP1 | 2.55 |  | HRBL | 3.1 |  | PIP4K2B | 2.68 |  | TPO | 3.52 |
| CFH | 2.58 |  | HRC | 3.05 |  | PITPNM3 | 3.05 |  | TPSG1 | 3.22 |
| CHAC1 | 2.61 |  | HRC | 2.69 |  | PITPNM3 | 3.26 |  | TPSG1 | 3.82 |
| CHAF1A | 2.61 |  | HRH1 | 2.66 |  | PITPNM3 | 4.21 |  | TPSG1 | 3.3 |
| CHAF1B | 2.82 |  | HRH2 | 3.3 |  | PITX1 | 2.68 |  | TRADD | 2.82 |
| CHAF1B | 3.01 |  | HRH4 | 2.87 |  | PITX3 | 2.78 |  | TRADD | 2.54 |
| CHAF1B | 2.86 |  | HS3ST1 | 2.77 |  | PIWIL2 | 3.51 |  | TRAF3IP1 | 3.32 |
| CHAT | 2.91 |  | HS3ST1 | 3.34 |  | PKD1 | 3.5 |  | TRAF3IP1 | 3.28 |
| CHAT | 2.65 |  | HS3ST1 | 3.75 |  | PKD1 | 2.97 |  | TRAF3IP1 | 2.63 |
| CHCHD3 | 3.14 |  | HS6ST1 | 2.64 |  | PKD1 | 4.37 |  | TRAF3IP3 | 2.65 |
| CHCHD3 | 2.53 |  | HSD11B2 | 2.52 |  | PKNOX1 | 2.56 |  | TRAPPC3 | 2.63 |
| CHCHD3 | 2.66 |  | HSD17B6 | 3.2 |  | PKP3 | 2.86 |  | TRAPPC3 | 2.51 |
| CHD9 | 4.48 |  | HSD17B6 | 2.71 |  | PKP3 | 2.51 |  | TRAPPC9 | 2.53 |
| CHD9 | 2.77 |  | HSF1 | 3.3 |  | PKP3 | 2.53 |  | TREH | 3.53 |
| CHERP | 3.09 |  | HSF2 | 2.62 |  | PKP3 | 2.55 |  | TREML2 | 2.52 |
| CHERP | 3.5 |  | HSP90AA1 | 2.65 |  | PLA2G2A | 2.5 |  | TRIM2 | 2.68 |
| CHGA | 3.13 |  | HSP90B1 | 2.54 |  | PLA2G2E | 3.41 |  | TRIM2 | 2.56 |
| CHGA | 2.96 |  | HSPA1A | 2.7 |  | PLA2G2E | 3.13 |  | TRIM26 | 3.09 |
| CHGA | 2.74 |  | HSPA8 | 2.65 |  | PLA2G6 | 2.62 |  | TRIM27 | 2.7 |
| CHL1 | 2.66 |  | HSPA9 | 2.86 |  | PLA2G6 | 2.64 |  | TRIM3 | 3.01 |
| CHL1 | 2.59 |  | HSPC111 | 2.76 |  | PLA2R1 | 2.98 |  | TRIM33 | 2.51 |
| CHMP2B | 2.7 |  | HSPC111 | 2.85 |  | PLCB1 | 2.63 |  | TRIM33 | 2.96 |
| CHMP2B | 2.99 |  | HSPC111 | 2.58 |  | PLCH1 | 3.22 |  | TRIM33 | 2.56 |
| CHMP5 | 3.61 |  | HSPC111 | 2.61 |  | PLD1 | 2.64 |  | TRIM34 | 2.65 |
| CHMP5 | 2.57 |  | HSPC111 | 2.72 |  | PLD1 | 2.85 |  | TRIM58 | 2.69 |
| CHMP6 | 2.73 |  | HTATSF1 | 3.5 |  | PLEKHA5 | 2.97 |  | TRIM58 | 2.71 |
| CHN2 | 3.04 |  | HTR3A | 2.75 |  | PLEKHA6 | 2.63 |  | TRIO | 2.59 |
| CHRM4 | 2.86 |  | HTR3A | 2.59 |  | PLEKHA6 | 2.51 |  | TRMT1 | 3.34 |
| CHRM4 | 3.82 |  | HTR4 | 3.92 |  | PLEKHB2 | 3.66 |  | TRMT1 | 3.42 |
| CHRNA4 | 2.59 |  | HTR4 | 3.29 |  | PLEKHB2 | 3.35 |  | TRMT1 | 2.69 |
| CHRNA4 | 2.96 |  | HTR4 | 4.37 |  | PLEKHB2 | 3.1 |  | TRMU | 2.69 |
| CHRNA6 | 2.79 |  | HTR7 | 3.68 |  | PLEKHF1 | 2.52 |  | TRMU | 3.11 |
| CHRNB1 | 2.71 |  | HTR7 | 3.1 |  | PLEKHF2 | 3.34 |  | TRMU | 2.66 |
| CHRNB2 | 3.27 |  | HTR7 | 2.99 |  | PLEKHJ1 | 2.5 |  | TRO | 3.07 |
| CHRNB2 | 3.92 |  | HTR7 | 2.65 |  | PLEKHM1 | 2.65 |  | TRO | 3.26 |
| CHRNB2 | 3.2 |  | HUWE1 | 2.63 |  | PLK4 | 2.74 |  | TRO | 2.9 |
| CHRND | 2.87 |  | HUWE1 | 2.65 |  | PLLP | 2.87 |  | TROVE2 | 2.79 |
| CHST10 | 2.64 |  | HUWE1 | 2.79 |  | PLLP | 3.43 |  | TRPC2 | 3.19 |
| CHST10 | 2.78 |  | HYAL3 | 2.59 |  | PLLP | 2.92 |  | TRPC3 | 2.58 |
| CHST2 | 2.58 |  | HYAL3 | 3.28 |  | PLXNC1 | 2.52 |  | TRPM8 | 2.92 |
| CHST3 | 2.61 |  | HYI | 2.98 |  | PLXND1 | 2.51 |  | TSC2 | 2.61 |
| CIB2 | 2.55 |  | HYI | 3.57 |  | PMFBP1 | 3.04 |  | TSC22D2 | 2.52 |
| CIITA | 2.72 |  | HYI | 3.43 |  | PMFBP1 | 3.21 |  | TSFM | 2.6 |
| CIR | 2.6 |  | IBSP | 2.5 |  | PML | 3.63 |  | TSHR | 3.19 |
| CIZ1 | 2.63 |  | ICA1 | 2.81 |  | PML | 3.32 |  | TSKS | 2.63 |
| CLCA3 | 2.82 |  | ICA1 | 3.04 |  | PMPCA | 2.95 |  | TSKS | 3.37 |
| CLCN5 | 2.97 |  | ICOSLG | 2.54 |  | PMS2L11 | 2.81 |  | TSNAX | 3.91 |
| CLCN6 | 2.67 |  | ICOSLG | 2.62 |  | PNKP | 2.54 |  | TSNAX | 2.83 |
| CLDN11 | 2.59 |  | ID2 | 2.93 |  | PNLIPRP2 | 3.03 |  | TSPAN9 | 2.91 |
| CLDN11 | 3.53 |  | ID4 | 2.52 |  | PNMA3 | 2.59 |  | TSPAN9 | 2.7 |
| CLDN11 | 2.88 |  | IDS | 2.76 |  | PNPLA2 | 2.72 |  | TSPYL1 | 3.35 |
| CLDN18 | 2.52 |  | IDS | 3.61 |  | PNPLA2 | 2.71 |  | TSSC4 | 3.44 |
| CLEC11A | 2.65 |  | IDS | 3.2 |  | PNPLA2 | 2.84 |  | TSSC4 | 2.8 |
| CLEC2D | 2.51 |  | IER2 | 2.57 |  | POFUT1 | 3.89 |  | TSSK1A | 2.94 |
| CLIC3 | 2.95 |  | IFI16 | 3.41 |  | POLA2 | 2.63 |  | TSSK2 | 3.63 |
| CLIC3 | 2.62 |  | IFI16 | 2.58 |  | POLD1 | 2.76 |  | TSTA3 | 2.56 |
| CLIP3 | 2.74 |  | IFI16 | 3.1 |  | POLE3 | 2.67 |  | TTC17 | 3.11 |
| CLNS1A | 2.57 |  | IFI16 | 3.27 |  | POLL | 2.71 |  | TTC17 | 2.72 |
| CLOCK | 3.04 |  | IFI16 | 2.6 |  | POLR2A | 3.44 |  | TTC22 | 2.61 |
| CLOCK | 2.58 |  | IFI16 | 3.21 |  | POLR2B | 2.85 |  | TTC22 | 2.76 |
| CLOCK | 2.55 |  | IFIH1 | 2.52 |  | POLR2F | 2.6 |  | TTC22 | 3.55 |
| CLPS | 2.53 |  | IFIH1 | 2.82 |  | POLR2L | 3.04 |  | TTC3 | 2.68 |
| CLTA | 2.52 |  | IFIT2 | 2.53 |  | POLR2L | 3.19 |  | TTC38 | 3.12 |
| CLTA | 2.61 |  | IFNA6 | 2.59 |  | POLR2L | 3.18 |  | TTK | 2.5 |
| CLUL1 | 2.62 |  | IFRD2 | 2.59 |  | POLR3G | 3.36 |  | TTN | 2.69 |
| CLUL1 | 2.87 |  | IGBP1 | 2.62 |  | POLRMT | 4.44 |  | TTPAL | 3.03 |
| CMTM6 | 3.62 |  | IGF2 | 2.56 |  | POLRMT | 3.31 |  | TTR | 2.54 |
| CMTM6 | 3.26 |  | IGF2BP3 | 2.82 |  | POLRMT | 3.03 |  | TTRAP | 2.65 |
| CMTM6 | 3.34 |  | IGFBP5 | 3.07 |  | POMP | 2.59 |  | TTRAP | 2.81 |
| CNDP2 | 3.47 |  | IGFBP5 | 2.66 |  | POSTN | 2.5 |  | TTYH1 | 2.63 |
| CNGB3 | 2.72 |  | IGFBP7 | 2.71 |  | POSTN | 2.62 |  | TTYH1 | 2.64 |
| CNIH | 3.04 |  | IGH@ | 2.81 |  | POU2F1 | 2.72 |  | TTYH1 | 3.91 |
| CNIH | 2.56 |  | IGH@ | 3.46 |  | POU3F2 | 2.8 |  | TUB | 2.61 |
| CNIH | 3.46 |  | IGH@ | 3.07 |  | POU5F1 | 2.85 |  | TUB | 2.87 |
| CNN1 | 3.43 |  | IGH@ | 3.23 |  | POU5F1P3 | 2.63 |  | TUBB | 2.87 |
| CNN1 | 2.96 |  | IGH@ | 2.85 |  | POU6F1 | 2.56 |  | TUBB | 3.16 |
| CNOT1 | 2.87 |  | IGH@ | 3.43 |  | PPARA | 2.9 |  | TUBB4Q | 2.51 |
| CNOT1 | 3.36 |  | IGH@ | 3.48 |  | PPEF1 | 3.06 |  | TULP1 | 2.61 |
| CNOT7 | 2.72 |  | IGH@ | 3.16 |  | PPEF1 | 2.61 |  | TULP3 | 2.73 |
| CNOT7 | 2.79 |  | IGH@ | 2.91 |  | PPEF2 | 3.69 |  | TUT1 | 3.06 |
| CNTD2 | 3.94 |  | IGH@ | 2.53 |  | PPEF2 | 3.05 |  | TXNDC1 | 2.72 |
| CNTD2 | 3.24 |  | IGHA1 | 3.42 |  | PPFIBP1 | 3.07 |  | TXNDC1 | 2.55 |
| CNTD2 | 3.27 |  | IGHA1 | 2.87 |  | PPFIBP1 | 2.62 |  | TXNDC14 | 2.81 |
| CNTFR | 3.96 |  | IGHA1 | 3 |  | PPIH | 2.73 |  | U2AF2 | 2.85 |
| CNTN5 | 2.79 |  | IGHA1 | 2.67 |  | PPIL2 | 2.61 |  | U2AF2 | 2.54 |
| COBLL1 | 2.73 |  | IGHA1 | 3.02 |  | PPIL2 | 3.61 |  | U2AF2 | 3.47 |
| COG2 | 3.31 |  | IGHA1 | 2.79 |  | PPIL2 | 2.69 |  | UBA3 | 3.26 |
| COL11A2 | 2.55 |  | IGHA1 | 2.8 |  | PPIL2 | 2.62 |  | UBA3 | 2.84 |
| COL14A1 | 3.11 |  | IGHA1 | 2.7 |  | PPM1A | 3.06 |  | UBA3 | 2.69 |
| COL17A1 | 2.6 |  | IGHG1 | 2.83 |  | PPM1A | 2.64 |  | UBAP1 | 2.57 |
| COL1A1 | 2.56 |  | IGHG1 | 2.67 |  | PPM1B | 2.53 |  | UBAP2L | 2.74 |
| COL1A1 | 3.3 |  | IGHG1 | 2.61 |  | PPM1F | 2.92 |  | UBE2A | 2.57 |
| COL1A1 | 3.24 |  | IGHG1 | 2.87 |  | PPP1CB | 3.14 |  | UBE2B | 3.32 |
| COL1A2 | 2.53 |  | IGHG1 | 2.99 |  | PPP1CC | 3.52 |  | UBE2D2 | 2.81 |
| COL4A3 | 2.83 |  | IGHG1 | 2.84 |  | PPP1CC | 3.01 |  | UBE2D2 | 2.87 |
| COL5A1 | 3.75 |  | IGHM | 2.7 |  | PPP1R11 | 2.85 |  | UBE2D3 | 2.85 |
| COL5A1 | 2.89 |  | IGHM | 2.51 |  | PPP1R15A | 2.84 |  | UBE2D3 | 2.69 |
| COL5A1 | 2.72 |  | IGHM | 3.73 |  | PPP2R1B | 3.14 |  | UBE2D4 | 3.42 |
| COL6A1 | 2.57 |  | IGK@ | 3.72 |  | PPP2R2D | 2.61 |  | UBE2I | 2.8 |
| COL6A1 | 3.25 |  | IGL@ | 3.2 |  | PPP2R5A | 3.2 |  | UBE2I | 3.57 |
| COL6A1 | 2.8 |  | IGL@ | 2.72 |  | PPP2R5C | 2.91 |  | UBE2I | 3.41 |
| COL6A2 | 3.72 |  | IGL@ | 2.73 |  | PPP2R5C | 2.83 |  | UBE2I | 3.03 |
| COL9A1 | 2.51 |  | IGL@ | 3.55 |  | PPP3CA | 3.51 |  | UBE2O | 2.94 |
| COMMD4 | 3.18 |  | IGL@ | 3.19 |  | PPP3CC | 2.54 |  | UBE2O | 4.03 |
| COMMD4 | 3.1 |  | IGL@ | 2.52 |  | PPP6C | 3.43 |  | UBE2O | 2.63 |
| COMMD8 | 2.75 |  | IGLV6-57 | 2.93 |  | PPP6C | 3.08 |  | UBE3A | 2.74 |
| COMMD8 | 3.39 |  | IGLV6-57 | 2.88 |  | PPP6C | 2.53 |  | UBE4A | 2.7 |
| COMMD8 | 2.54 |  | IGLV6-57 | 3.43 |  | PPT1 | 2.94 |  | UBE4A | 2.92 |
| COPA | 2.55 |  | IKBKAP | 2.74 |  | PPT1 | 2.77 |  | UBE4A | 3.15 |
| COPB1 | 2.77 |  | IKBKE | 2.84 |  | PPY | 3.4 |  | UBL3 | 2.52 |
| COPS4 | 2.84 |  | IL10RB | 2.56 |  | PPY | 3.65 |  | UBP1 | 3.37 |
| COPS5 | 2.81 |  | IL10RB | 2.67 |  | PPY | 4.33 |  | UBP1 | 3.15 |
| COPS7B | 2.77 |  | IL12RB1 | 3.73 |  | PPY2 | 2.57 |  | UBP1 | 2.91 |
| COPZ1 | 3.52 |  | IL12RB1 | 3.54 |  | PPY2 | 2.51 |  | UBQLN2 | 2.7 |
| COQ3 | 2.95 |  | IL12RB1 | 2.62 |  | PRDM10 | 2.68 |  | UBQLN2 | 3.06 |
| COQ7 | 2.72 |  | IL13 | 2.85 |  | PRDM11 | 2.94 |  | UBR4 | 2.73 |
| COQ7 | 2.62 |  | IL13 | 3.22 |  | PRDM11 | 2.8 |  | UBTD1 | 2.9 |
| COQ7 | 3.47 |  | IL13RA1 | 2.67 |  | PRDM11 | 2.58 |  | UBTD1 | 2.71 |
| CORIN | 2.81 |  | IL17RC | 3.31 |  | PRDM12 | 3.58 |  | UBTD1 | 3.14 |
| COX11 | 2.53 |  | IL1RL1 | 2.81 |  | PRDM12 | 3.98 |  | UCK2 | 3.05 |
| COX15 | 2.59 |  | IL27RA | 3.05 |  | PRDM12 | 3.69 |  | UCP1 | 2.63 |
| COX6A2 | 2.7 |  | IL27RA | 4.32 |  | PRDM16 | 3.49 |  | UCP3 | 2.55 |
| CPA1 | 3.23 |  | IL27RA | 3.98 |  | PRDM16 | 2.64 |  | UGT2A3 | 2.8 |
| CPD | 2.66 |  | IL2RA | 3.03 |  | PRDM16 | 3.02 |  | UGT2B4 | 2.76 |
| CPN1 | 2.51 |  | IL2RA | 3.48 |  | PRDM8 | 3.54 |  | UGT2B4 | 3.21 |
| CPN1 | 2.54 |  | IL4 | 2.91 |  | PRDX2 | 2.87 |  | UNC50 | 2.81 |
| CPNE1 | 2.99 |  | IL9R | 2.58 |  | PRDX2 | 3.24 |  | UNC50 | 3.01 |
| CPNE1 | 3.53 |  | IL9R | 2.71 |  | PRG2 | 2.54 |  | UNC50 | 2.61 |
| CPSF4 | 2.73 |  | ILF3 | 2.67 |  | PRICKLE3 | 2.61 |  | UNC93A | 2.75 |
| CPSF4 | 2.64 |  | INF2 | 3 |  | PRICKLE3 | 2.52 |  | UPF1 | 2.72 |
| CPZ | 3.1 |  | INF2 | 2.64 |  | PRICKLE4 | 2.92 |  | UPF1 | 2.99 |
| CRABP1 | 3.46 |  | INO80D | 2.68 |  | PRKACB | 2.64 |  | UPF1 | 2.8 |
| CRABP1 | 3.37 |  | INSL3 | 2.8 |  | PRKAG2 | 2.54 |  | UQCRC2 | 2.63 |
| CRABP1 | 3.9 |  | INVS | 3.21 |  | PRKAG2 | 3.73 |  | UQCRFS1 | 2.92 |
| CRAT | 3.22 |  | INVS | 2.76 |  | PRKAR1A | 3.86 |  | UQCRFS1 | 3.36 |
| CRAT | 3.44 |  | IQGAP1 | 2.6 |  | PRKAR1A | 2.54 |  | URG4 | 2.68 |
| CRAT | 3.69 |  | IRF4 | 2.92 |  | PRKAR2A | 2.84 |  | USE1 | 2.85 |
| CREB3 | 2.55 |  | IRF4 | 3.72 |  | PRKAR2A | 3.2 |  | USP1 | 2.61 |
| CREBBP | 2.62 |  | IRGC | 2.88 |  | PRKDC | 3.06 |  | USP12 | 3.19 |
| CREBBP | 2.54 |  | IRX5 | 2.65 |  | PRKDC | 3.09 |  | USP12 | 2.52 |
| CREBBP | 3.02 |  | ISCA1 | 2.94 |  | PRKG1 | 3.08 |  | USP14 | 2.55 |
| CREBL2 | 2.5 |  | ISG20L2 | 2.69 |  | PRMT2 | 2.62 |  | USP14 | 2.51 |
| CREBZF | 2.58 |  | ISYNA1 | 2.5 |  | PRMT7 | 2.65 |  | USP22 | 2.99 |
| CRELD2 | 2.53 |  | ITFG2 | 2.6 |  | PRMT7 | 2.66 |  | USP22 | 2.66 |
| CRIP2 | 2.51 |  | ITGAM | 3.61 |  | PRND | 2.51 |  | USP34 | 3.7 |
| CRISP1 | 2.75 |  | ITGAM | 3.35 |  | PRNP | 2.58 |  | USP9X | 3.52 |
| CRKRS | 2.83 |  | ITGB1 | 2.64 |  | PRO0132 | 2.65 |  | UTF1 | 3.33 |
| CROCCL2 | 3.69 |  | ITGB1 | 3.26 |  | PRO0132 | 3 |  | UTF1 | 3.47 |
| CROCCL2 | 3.26 |  | ITGB1 | 3.11 |  | PRO1880 | 3.76 |  | UTF1 | 3.05 |
| CROP | 3.02 |  | ITGB1BP1 | 2.74 |  | PRO1880 | 3.3 |  | UTP14A | 3.25 |
| CRTC1 | 3.29 |  | ITGB3 | 3.31 |  | PRO2012 | 2.51 |  | VAC14 | 3.19 |
| CRTC1 | 2.78 |  | ITGB3 | 2.53 |  | PROM1 | 2.75 |  | VAMP3 | 3.24 |
| CRTC1 | 2.54 |  | ITGB3 | 2.64 |  | PROSC | 3.02 |  | VAMP3 | 2.63 |
| CRYAA | 3.61 |  | ITGB4 | 2.62 |  | PROSC | 2.71 |  | VAMP5 | 2.89 |
| CRYAA | 2.62 |  | ITGB4 | 2.52 |  | PRPF31 | 2.52 |  | VAMP5 | 2.82 |
| CRYBB1 | 2.5 |  | ITGB4 | 3.17 |  | PRPF40A | 2.69 |  | VCAM1 | 2.94 |
| CRYGD | 2.66 |  | ITGBL1 | 2.82 |  | PRPF40A | 2.8 |  | VCPIP1 | 2.5 |
| CS | 3.24 |  | ITIH1 | 2.68 |  | PRPS2 | 2.55 |  | VCX2 | 3.99 |
| CS | 2.54 |  | ITM2B | 2.9 |  | PRPSAP1 | 2.55 |  | VCX2 | 3.05 |
| CSDC2 | 2.68 |  | ITM2B | 4.17 |  | PRR13 | 3.21 |  | VDAC2 | 2.9 |
| CSF1 | 3.44 |  | ITM2B | 2.73 |  | PRR13 | 2.67 |  | VDAC2 | 3.01 |
| CSGLCA-T | 2.58 |  | ITPKA | 2.88 |  | PRR14 | 2.77 |  | VDAC3 | 2.55 |
| CSGLCA-T | 3.67 |  | ITSN2 | 2.94 |  | PRR14 | 2.83 |  | VGLL1 | 2.89 |
| CSGLCA-T | 3.31 |  | IVD | 2.52 |  | PRR14 | 2.74 |  | VGLL1 | 2.51 |
| CSH1 | 2.5 |  | IVNS1ABP | 2.59 |  | PRR16 | 3.43 |  | VIP | 2.56 |
| CSH1 | 2.6 |  | IVNS1ABP | 2.92 |  | PRRG3 | 3.04 |  | VIPR1 | 2.75 |
| CSH1 | 2.57 |  | IVNS1ABP | 2.5 |  | PRSS1 | 2.65 |  | VIPR2 | 2.76 |
| CSH1 | 3.24 |  | IVNS1ABP | 3.09 |  | PRSS3 | 3.86 |  | VIPR2 | 2.99 |
| CSH1 | 2.61 |  | IVNS1ABP | 2.69 |  | PRSS3 | 3.06 |  | VPS11 | 2.63 |
| CSH2 | 2.54 |  | JAG1 | 3.23 |  | PRSS3 | 3.09 |  | VPS13B | 2.73 |
| CSH2 | 3.18 |  | JAG1 | 3.22 |  | PRSS3 | 4 |  | VPS16 | 2.55 |
| CSH2 | 3.55 |  | JAK1 | 2.76 |  | PSEN1 | 2.57 |  | VPS24 | 3.27 |
| CSHL1 | 2.73 |  | JAK3 | 2.9 |  | PSEN1 | 3.1 |  | VPS24 | 3.19 |
| CSK | 2.52 |  | JMJD1B | 2.5 |  | PSG1 | 2.56 |  | VPS24 | 3.07 |
| CSK | 2.7 |  | JMJD2B | 2.96 |  | PSKH1 | 2.6 |  | VPS28 | 2.5 |
| CSNK1E | 2.61 |  | JMJD3 | 2.75 |  | PSKH1 | 2.54 |  | VPS39 | 4.27 |
| CSPG4 | 2.62 |  | JMJD4 | 2.63 |  | PSMA2 | 2.62 |  | VPS39 | 2.74 |
| CSPG4 | 3.2 |  | JMJD7 | 3.21 |  | PSMA4 | 3.04 |  | VPS45 | 2.52 |
| CSPG4 | 2.83 |  | JMJD7 | 3.5 |  | PSMA4 | 2.74 |  | VPS4B | 3.37 |
| CSPG4 | 4.13 |  | JMJD7 | 3.13 |  | PSMB1 | 2.64 |  | VPS4B | 2.97 |
| CSPG4LYP1 | 2.66 |  | JRK | 3.34 |  | PSMB1 | 2.59 |  | VPS4B | 3.84 |
| CSPG5 | 2.54 |  | JRK | 3.26 |  | PSMC2 | 3.18 |  | WAS | 2.61 |
| CST4 | 2.51 |  | JUND | 2.96 |  | PSMC2 | 2.61 |  | WBP11 | 2.57 |
| CTBP2 | 2.75 |  | KANK2 | 2.69 |  | PSMD4 | 3.1 |  | WDHD1 | 2.78 |
| CTBS | 2.65 |  | KANK2 | 3.32 |  | PSMD4 | 2.72 |  | WDR26 | 2.72 |
| CTBS | 2.95 |  | KANK2 | 2.53 |  | PSMD7 | 2.79 |  | WDR33 | 2.59 |
| CTGLF1 | 2.98 |  | KARS | 2.7 |  | PSMD7 | 4.41 |  | WDR59 | 2.88 |
| CTGLF1 | 2.54 |  | KCNA4 | 2.61 |  | PSMD7 | 2.86 |  | WDR59 | 2.63 |
| CTNNA1 | 3.38 |  | KCNC4 | 2.7 |  | PSME1 | 3.08 |  | WDTC1 | 3.24 |
| CTNNA1 | 2.7 |  | KCNF1 | 2.99 |  | PSME3 | 3.17 |  | WDTC1 | 3.19 |
| CTNND1 | 3.1 |  | KCNH1 | 2.86 |  | PSMG1 | 2.71 |  | WDTC1 | 4.3 |
| CTSG | 2.78 |  | KCNIP1 | 2.75 |  | PSMG2 | 2.87 |  | WIZ | 2.77 |
| CTSS | 3.35 |  | KCNJ4 | 3.96 |  | PSMG2 | 2.68 |  | WIZ | 3.15 |
| CTSS | 3.59 |  | KCNJ4 | 2.71 |  | PSPN | 3.19 |  | WIZ | 2.96 |
| CTSS | 3.18 |  | KCNJ4 | 4.02 |  | PSPN | 3.5 |  | WNT11 | 2.63 |
| CUGBP1 | 3.06 |  | KCNJ5 | 2.91 |  | PTAFR | 3.6 |  | WNT3 | 3.39 |
| CUGBP1 | 2.78 |  | KCNK13 | 4.31 |  | PTBP1 | 2.65 |  | WNT3 | 2.87 |
| CUGBP2 | 2.63 |  | KCNK13 | 2.7 |  | PTBP1 | 2.57 |  | WNT4 | 2.52 |
| CUX1 | 2.66 |  | KCNN3 | 2.77 |  | PTCD1 | 2.76 |  | WNT4 | 2.66 |
| CUX1 | 2.62 |  | KCNN3 | 2.83 |  | PTCD1 | 3.3 |  | WNT6 | 2.54 |
| CUX1 | 3.43 |  | KCNN3 | 2.79 |  | PTDSS2 | 3.72 |  | WNT6 | 4.14 |
| CUZD1 | 2.57 |  | KCNN4 | 2.53 |  | PTDSS2 | 2.99 |  | WNT7B | 2.56 |
| CUZD1 | 3.56 |  | KCNQ1 | 2.9 |  | PTDSS2 | 3.11 |  | WNT7B | 2.59 |
| CUZD1 | 2.58 |  | KCNQ1 | 2.79 |  | PTGDS | 2.65 |  | WNT7B | 2.66 |
| CXorf36 | 2.8 |  | KCNQ2 | 2.72 |  | PTGER1 | 3.11 |  | WSB1 | 3.24 |
| CXorf36 | 3.23 |  | KCNQ2 | 3.37 |  | PTGER1 | 2.89 |  | WSB1 | 3.71 |
| CXorf57 | 3.24 |  | KCNQ2 | 2.67 |  | PTGER1 | 2.92 |  | WSB1 | 2.53 |
| CXYorf2 | 2.55 |  | KCNQ2 | 2.61 |  | PTGER1 | 3.01 |  | WSB2 | 2.56 |
| CXYorf2 | 4.38 |  | KDELC1 | 2.69 |  | PTGER1 | 3.3 |  | WSCD1 | 3.81 |
| CYCS | 3.35 |  | KDELR3 | 2.62 |  | PTGER1 | 3.15 |  | WSCD1 | 3.06 |
| CYCS | 3.25 |  | KDELR3 | 2.7 |  | PTGER3 | 3.21 |  | WSCD1 | 3.1 |
| CYCS | 3.32 |  | KHDRBS1 | 2.68 |  | PTGES2 | 4.27 |  | WT1 | 3.31 |
| CYCS | 2.99 |  | KHDRBS1 | 3.61 |  | PTGIR | 2.96 |  | WT1 | 2.65 |
| CYHR1 | 2.54 |  | KHDRBS1 | 2.59 |  | PTGS1 | 2.53 |  | WWC1 | 3.31 |
| CYHR1 | 3.9 |  | KHDRBS3 | 3.15 |  | PTHR1 | 3.12 |  | WWC1 | 2.77 |
| CYLD | 2.72 |  | KHDRBS3 | 2.79 |  | PTHR1 | 2.77 |  | WWC1 | 3.28 |
| CYLD | 2.7 |  | KHSRP | 2.99 |  | PTK7 | 2.52 |  | WWP1 | 3.15 |
| CYLD | 2.56 |  | KIAA0100 | 2.85 |  | PTMS | 3.16 |  | WWP1 | 2.85 |
| CYP1A2 | 2.95 |  | KIAA0174 | 2.76 |  | PTMS | 3.25 |  | XPA | 2.71 |
| CYP1A2 | 2.78 |  | KIAA0194 | 3.66 |  | PTMS | 4.65 |  | XPNPEP2 | 2.67 |
| CYP1A2 | 3.1 |  | KIAA0194 | 3.32 |  | PTP4A2 | 3.13 |  | XPNPEP2 | 2.51 |
| CYP1B1 | 2.89 |  | KIAA0194 | 3.2 |  | PTP4A2 | 2.94 |  | XRCC6 | 2.55 |
| CYP1B1 | 3.07 |  | KIAA0195 | 3.23 |  | PTP4A2 | 3.91 |  | XYLB | 2.54 |
| CYP27B1 | 2.56 |  | KIAA0195 | 3.8 |  | PTP4A2 | 3.11 |  | YBX1 | 2.86 |
| CYP2B6 | 3.07 |  | KIAA0195 | 3.99 |  | PTP4A2 | 2.57 |  | YBX1 | 3.14 |
| CYP2B6 | 3.21 |  | KIAA0241 | 2.51 |  | PTPN1 | 3.24 |  | YIPF2 | 2.52 |
| CYP2B6 | 2.83 |  | KIAA0319 | 2.59 |  | PTPN1 | 2.75 |  | YIPF2 | 3.1 |
| CYP2D6 | 2.5 |  | KIAA0319 | 2.71 |  | PTPN1 | 2.53 |  | YIPF2 | 2.73 |
| CYP2D6 | 2.79 |  | KIAA0319 | 2.95 |  | PTPN21 | 2.53 |  | YIPF2 | 3.22 |
| CYP2E1 | 2.55 |  | KIAA0391 | 2.74 |  | PTPN21 | 3.03 |  | YIPF2 | 2.64 |
| CYP3A4 | 2.7 |  | KIAA0391 | 3.03 |  | PTPN21 | 2.94 |  | YLPM1 | 2.75 |
| CYP3A4 | 2.5 |  | KIAA0406 | 2.85 |  | PTPRK | 2.65 |  | YME1L1 | 2.52 |
| CYP4F8 | 2.61 |  | KIAA0408 | 2.66 |  | PTPRU | 2.83 |  | YME1L1 | 2.7 |
| CYP4F8 | 3.05 |  | KIAA0427 | 3.74 |  | PTTG1IP | 3.16 |  | YME1L1 | 2.9 |
| CYTH1 | 2.62 |  | KIAA0427 | 2.94 |  | PUM1 | 3.79 |  | YME1L1 | 2.88 |
| D4S234E | 2.99 |  | KIAA0427 | 3.6 |  | PVR | 2.5 |  | YME1L1 | 2.99 |
| D4S234E | 2.92 |  | KIAA0494 | 2.96 |  | PVRL1 | 4.23 |  | YME1L1 | 2.85 |
| D4S234E | 3.01 |  | KIAA0506 | 2.81 |  | PVRL1 | 3.72 |  | YME1L1 | 2.65 |
| DAD1 | 2.51 |  | KIAA0652 | 2.89 |  | PVRL1 | 3.53 |  | YME1L1 | 2.77 |
| DAPK3 | 2.64 |  | KIAA0774 | 2.57 |  | PXDN | 2.97 |  | YOD1 | 2.54 |
| DAPK3 | 2.76 |  | KIAA0892 | 4.15 |  | PXDN | 3.69 |  | YTHDF2 | 3.12 |
| DARS2 | 2.79 |  | KIAA0892 | 3.22 |  | PYCRL | 2.86 |  | YTHDF2 | 2.91 |
| DAZAP2 | 3 |  | KIAA0892 | 3.73 |  | PYCRL | 2.71 |  | YTHDF2 | 3.07 |
| DBH | 3.43 |  | KIAA0922 | 2.77 |  | PYCRL | 2.91 |  | YTHDF3 | 3.55 |
| DBN1 | 3.03 |  | KIAA0999 | 2.75 |  | PYGM | 2.66 |  | YTHDF3 | 3.47 |
| DBN1 | 3.36 |  | KIAA1024 | 2.85 |  | PYROXD1 | 2.66 |  | YWHAB | 3.74 |
| DBN1 | 3.29 |  | KIAA1109 | 3.61 |  | QKI | 2.88 |  | YWHAB | 2.67 |
| DBNDD1 | 2.59 |  | KIAA1128 | 2.56 |  | QKI | 2.57 |  | YWHAB | 3.17 |
| DBNDD1 | 3.04 |  | KIAA1245 | 2.67 |  | QSER1 | 2.58 |  | YWHAQ | 3.32 |
| DBNDD1 | 3.79 |  | KIAA1245 | 3.18 |  | QTRT1 | 2.8 |  | YWHAQ | 3.17 |
| DBP | 2.58 |  | KIAA1245 | 2.79 |  | QTRT1 | 2.73 |  | YWHAZ | 3.33 |
| DBP | 2.78 |  | KIAA1310 | 3.44 |  | QTRT1 | 3.44 |  | YY1 | 2.87 |
| DCAKD | 2.76 |  | KIAA1539 | 2.62 |  | RAB11A | 2.52 |  | YY1 | 2.91 |
| DCAKD | 2.68 |  | KIAA1622 | 2.55 |  | RAB13 | 2.57 |  | YY1 | 3.66 |
| DCHS2 | 2.77 |  | KIAA1655 | 2.81 |  | RAB14 | 2.59 |  | ZBED4 | 2.84 |
| DCPS | 2.65 |  | KIAA1655 | 3.1 |  | RAB1A | 2.9 |  | ZBTB17 | 3.17 |
| DCTN1 | 3.26 |  | KIAA1967 | 3.01 |  | RAB26 | 2.91 |  | ZBTB25 | 2.51 |
| DCTN1 | 2.54 |  | KIAA1967 | 3.67 |  | RAB27B | 2.7 |  | ZBTB32 | 3.07 |
| DCX | 2.94 |  | KIF14 | 2.61 |  | RAB2A | 2.92 |  | ZBTB32 | 2.92 |
| DDAH1 | 2.65 |  | KIF18A | 2.62 |  | RAB2A | 2.68 |  | ZBTB43 | 2.62 |
| DDC | 2.71 |  | KIF1A | 2.84 |  | RAB35 | 3.01 |  | ZBTB7A | 2.57 |
| DDOST | 3.74 |  | KIF22 | 2.61 |  | RAB3A | 2.65 |  | ZBTB7A | 2.92 |
| DDT | 3.18 |  | KIF23 | 2.9 |  | RAB3B | 2.66 |  | ZBTB7A | 2.62 |
| DDX28 | 2.65 |  | KIF25 | 2.9 |  | RAB40AL | 2.83 |  | ZBTB7B | 3.45 |
| DDX28 | 2.66 |  | KIF26B | 2.93 |  | RAB40C | 3.54 |  | ZBTB7B | 2.97 |
| DDX3X | 2.8 |  | KIF26B | 3.43 |  | RAB40C | 4.06 |  | ZC3H11A | 3 |
| DDX49 | 2.57 |  | KIF26B | 2.77 |  | RAB40C | 3.44 |  | ZC3H7B | 2.7 |
| DDX60 | 2.52 |  | KIF4A | 2.72 |  | RAB4A | 3.11 |  | ZC3H7B | 2.8 |
| DEDD | 3.07 |  | KIFC1 | 2.99 |  | RAB5B | 2.97 |  | ZCCHC8 | 2.87 |
| DEDD | 2.72 |  | KIFC1 | 3.06 |  | RAB5B | 3.53 |  | ZCCHC8 | 3.12 |
| DEF6 | 3.49 |  | KIFC1 | 2.53 |  | RAB6A | 3.15 |  | ZDHHC3 | 2.7 |
| DEF8 | 2.82 |  | KIR2DL4 | 2.67 |  | RAB7A | 3.2 |  | ZER1 | 3.4 |
| DENND1B | 3.19 |  | KIR2DL4 | 3.07 |  | RAB7A | 3.33 |  | ZER1 | 3.03 |
| DENND1B | 3.65 |  | KITLG | 2.87 |  | RAB7A | 2.56 |  | ZER1 | 2.53 |
| DENND1B | 3.1 |  | KITLG | 2.62 |  | RAB9P1 | 3.64 |  | ZFAND6 | 3 |
| DENND2A | 3.08 |  | KLF13 | 2.8 |  | RABIF | 3.08 |  | ZFAND6 | 2.98 |
| DENND2A | 2.6 |  | KLF5 | 2.59 |  | RAC1 | 2.71 |  | ZFAND6 | 2.52 |
| DEPDC1 | 2.58 |  | KLF6 | 2.98 |  | RAC1 | 2.56 |  | ZFP2 | 2.62 |
| DERA | 2.79 |  | KLHL1 | 4.52 |  | RAC3 | 2.98 |  | ZFP64 | 3.4 |
| DES | 3.59 |  | KLHL1 | 3.04 |  | RAD21 | 4.07 |  | ZFPL1 | 3.73 |
| DGCR2 | 2.82 |  | KLHL1 | 2.68 |  | RAD21 | 3.13 |  | ZFPL1 | 2.58 |
| DGCR2 | 3.4 |  | KLHL22 | 2.9 |  | RAD21 | 3.16 |  | ZG16 | 3 |
| DGCR2 | 2.96 |  | KLHL23 | 3.22 |  | RAD52 | 2.86 |  | ZG16 | 4.02 |
| DGKI | 2.57 |  | KLHL35 | 2.59 |  | RAD54L2 | 3.22 |  | ZG16 | 3.72 |
| DGKQ | 3.37 |  | KLHL35 | 2.96 |  | RALA | 2.77 |  | ZGPAT | 2.53 |
| DGKQ | 4.7 |  | KLHL9 | 4.4 |  | RALGPS1 | 3.03 |  | ZHX3 | 2.99 |
| DGKQ | 3.03 |  | KLHL9 | 2.6 |  | RAMP2 | 2.77 |  | ZIC3 | 2.61 |
| DHODH | 2.53 |  | KLK13 | 2.74 |  | RAMP2 | 3.26 |  | ZKSCAN1 | 2.95 |
| DHPS | 2.66 |  | KLK13 | 2.63 |  | RAMP2 | 2.94 |  | ZKSCAN5 | 2.84 |
| DHX30 | 2.57 |  | KLK13 | 3.15 |  | RAN | 2.64 |  | ZMAT5 | 2.9 |
| DHX34 | 2.53 |  | KLK13 | 2.79 |  | RANBP2 | 3.09 |  | ZNF137 | 2.97 |
| DHX34 | 2.86 |  | KLK14 | 3.15 |  | RANBP2 | 2.54 |  | ZNF142 | 2.52 |
| DHX34 | 3.07 |  | KLK15 | 2.63 |  | RANBP9 | 3.03 |  | ZNF142 | 3.38 |
| DHX34 | 3.01 |  | KLK2 | 2.84 |  | RANBP9 | 2.96 |  | ZNF174 | 3.03 |
| DHX38 | 3.18 |  | KLK7 | 2.8 |  | RAP1GAP | 3.24 |  | ZNF174 | 3.64 |
| DIAPH1 | 2.96 |  | KLRC3 | 2.74 |  | RAP1GAP | 2.76 |  | ZNF224 | 2.98 |
| DIAPH1 | 2.57 |  | KLRC4 | 2.72 |  | RAP1GAP | 2.77 |  | ZNF236 | 2.6 |
| DICER1 | 2.84 |  | KPNA2 | 2.76 |  | RAPGEF2 | 2.69 |  | ZNF24 | 2.65 |
| DICER1 | 2.71 |  | KPNA2 | 3.35 |  | RAPGEF2 | 3.04 |  | ZNF268 | 3.07 |
| DIO2 | 2.99 |  | KPNA3 | 3.27 |  | RAPGEF3 | 2.74 |  | ZNF282 | 4.11 |
| DIO2 | 2.85 |  | KPNA3 | 2.62 |  | RAPGEF3 | 2.8 |  | ZNF32 | 2.5 |
| DIO2 | 3.06 |  | KPNA3 | 2.54 |  | RAPGEF3 | 4.33 |  | ZNF330 | 2.69 |
| DIO2 | 2.9 |  | KPNA5 | 2.62 |  | RAPGEF4 | 2.85 |  | ZNF334 | 2.95 |
| DIXDC1 | 2.62 |  | KRAS | 2.84 |  | RAPSN | 3.45 |  | ZNF335 | 3.52 |
| DKC1 | 3.53 |  | KRAS | 2.92 |  | RARA | 3.1 |  | ZNF335 | 3.12 |
| DKC1 | 2.77 |  | KRCC1 | 3.18 |  | RARA | 2.55 |  | ZNF335 | 2.58 |
| DKFZP434A062 | 2.62 |  | KRT16 | 3.78 |  | RARG | 2.68 |  | ZNF337 | 2.68 |
| DKFZp434K191 | 3.22 |  | KRT16 | 2.86 |  | RASAL2 | 3.83 |  | ZNF408 | 2.56 |
| DKFZp434K191 | 3.25 |  | KRT19P2 | 2.53 |  | RASAL2 | 3.38 |  | ZNF467 | 3.9 |
| DKFZp434K191 | 3.96 |  | KRT19P2 | 3.86 |  | RASAL2 | 2.92 |  | ZNF467 | 3.41 |
| DKFZp566H0824 | 2.53 |  | KRT19P2 | 3.37 |  | RASL10A | 3.21 |  | ZNF467 | 2.73 |
| DKFZp686O1327 | 2.6 |  | KRT6A | 2.62 |  | RASL10A | 2.97 |  | ZNF528 | 3.45 |
| DLAT | 2.7 |  | KRT81 | 2.71 |  | RASL10A | 2.74 |  | ZNF528 | 3.83 |
| DLG1 | 2.72 |  | KRT81 | 2.65 |  | RASL12 | 3.11 |  | ZNF609 | 4.06 |
| DLG5 | 2.75 |  | KRT83 | 2.54 |  | RASL12 | 2.62 |  | ZNF609 | 2.52 |
| DLGAP1 | 2.58 |  | KRT86 | 2.54 |  | RAX | 2.92 |  | ZNF609 | 2.81 |
| DLGAP2 | 2.64 |  | KSR1 | 3.3 |  | RBBP8 | 2.59 |  | ZNF674 | 2.81 |
| DLGAP2 | 3.52 |  | L1CAM | 3.42 |  | RBBP8 | 3.04 |  | ZNF674 | 3.36 |
| DLGAP2 | 2.6 |  | LAG3 | 2.69 |  | RBM19 | 2.79 |  | ZNF688 | 2.59 |
| DLGAP4 | 4.21 |  | LAGE3 | 3.45 |  | RBM19 | 2.72 |  | ZNF711 | 2.99 |
| DLX2 | 2.73 |  | LAGE3 | 2.98 |  | RBM19 | 2.65 |  | ZNF75D | 3.46 |
| DLX2 | 3.19 |  | LAMA4 | 2.62 |  | RBM25 | 2.57 |  | ZNF768 | 2.58 |
| DLX4 | 2.83 |  | LAMA4 | 2.54 |  | RBM25 | 2.67 |  | ZNF768 | 2.74 |
| DLX5 | 3.06 |  | LAMC3 | 4.07 |  | RBM25 | 2.55 |  | ZNF771 | 3.13 |
| DMPK | 2.63 |  | LAMC3 | 2.83 |  | RBM26 | 2.92 |  | ZNF771 | 2.73 |
| DMPK | 3.1 |  | LAMC3 | 2.87 |  | RBM39 | 2.54 |  | ZNF771 | 3.33 |
| DMPK | 3.19 |  | LAP3 | 2.67 |  | RBM39 | 2.88 |  | ZNF771 | 3.12 |
| DMTF1 | 3.27 |  | LAPTM4A | 2.53 |  | RBM47 | 3.35 |  | ZNF780B | 2.92 |
| DNAH17 | 2.64 |  | LARP4 | 2.82 |  | RBM4B | 3.34 |  | ZNF80 | 3.21 |
| DNAJA1 | 2.77 |  | LARP4 | 2.96 |  | RBM8A | 3.38 |  | ZNF821 | 3.42 |
| DNAJA1 | 2.6 |  | LARP4 | 2.87 |  | RBM9 | 2.61 |  | ZNF821 | 4.28 |
| DNAJB12 | 2.6 |  | LARS2 | 2.7 |  | RBP3 | 2.75 |  | ZNF821 | 3.1 |
| DNAJC10 | 2.59 |  | LASP1 | 3.07 |  | RCAN3 | 2.63 |  | ZNF821 | 3.71 |
| DNAJC2 | 2.67 |  | LASP1 | 3.26 |  | RCBTB2 | 2.58 |  | ZNF839 | 2.93 |
| DNMT3L | 2.69 |  | LASP1 | 3.24 |  | RCBTB2 | 3.13 |  | ZNF839 | 3.15 |
| DOC2B | 2.95 |  | LASS4 | 2.87 |  | RCE1 | 2.66 |  | ZNF839 | 3.46 |
| DOC2B | 2.96 |  | LBX1 | 2.59 |  | RCN3 | 2.8 |  | ZNF84 | 3.68 |
| DOC2B | 3.19 |  | LBX1 | 3.07 |  | RCN3 | 2.94 |  | ZNF85 | 2.73 |
| DOCK3 | 2.87 |  | LBX1 | 2.63 |  | RCP9 | 2.53 |  | ZNHIT2 | 2.75 |
| DOCK4 | 2.7 |  | LDB3 | 2.97 |  | RDH11 | 2.9 |  | ZNRF4 | 3.22 |
| DOCK6 | 3.19 |  | LDHA | 2.73 |  | RDH5 | 2.87 |  | ZNRF4 | 3.94 |
| DOK3 | 3.33 |  | LDLR | 2.53 |  | RDH5 | 2.58 |  | ZNRF4 | 4.51 |
| DOK4 | 2.55 |  | LDLR | 2.87 |  | RECQL4 | 3.13 |  | ZP2 | 2.72 |
| DOM3Z | 2.9 |  | LHX2 | 2.94 |  | RECQL4 | 2.59 |  | ZP2 | 3.79 |
| DOT1L | 4.35 |  | LHX2 | 2.56 |  | RECQL4 | 3.04 |  | ZSCAN18 | 2.57 |
| DOT1L | 3.37 |  | LILRA5 | 2.68 |  | REEP2 | 3.03 |  | ZSCAN2 | 2.75 |
| DOT1L | 2.87 |  | LIMA1 | 2.5 |  | REG1A | 2.84 |  | ZSCAN2 | 2.83 |
| DPEP1 | 2.5 |  | LIMK1 | 3.08 |  | REN | 2.68 |  | ZSCAN5A | 3.22 |
| DPEP3 | 2.67 |  | LIMS1 | 3.29 |  | RERE | 2.9 |  | ZZEF1 | 2.87 |
| DPP8 | 2.86 |  | LIN7B | 2.65 |  | RERE | 2.91 |  | ZZEF1 | 3.61 |
| DPT | 2.73 |  | LITAF | 2.52 |  | RERGL | 2.96 |  | ZZEF1 | 3.51 |
| DPT | 2.68 |  | LMCD1 | 2.5 |  | RERGL | 3.62 |  | ZZZ3 | 2.57 |
| DPT | 2.82 |  | LMF2 | 2.68 |  | RFK | 2.58 |  |  |  |
