# Supplementary material for: Suppressed Expression of T-Box Transcription Factors Is Involved in Senescence in Chronic Obstructive Pulmonary Disease
Source: PLoS Comput Biol. 2012 Jul 19;8(7):e1002597. doi: 10.1371/journal.pcbi.1002597 (PMC3400575; doi:10.1371/journal.pcbi.1002597)
Supplement: Table S3 — Direct Connections to CDKN2A in the CLR-Generated Network. The data in this table correspond to Figure S2B. (DOC) [file pcbi.1002597.s007.doc]

**Table S3. Direct Connections to CDKN2A in the CLR-Generated Network (See Figure S2B)**

Note: Certain genes are represented by multiple probe sets and so occur more than once in the network.

| GENE | CLR_LIKELIHOOD_ESTIMATE_(ABSOLUTE) |  | GENE | CLR_LIKELIHOOD_ESTIMATE_(ABSOLUTE) |  | GENE | CLR_LIKELIHOOD_ESTIMATE_(ABSOLUTE) |  | GENE | CLR_LIKELIHOOD_ESTIMATE_(ABSOLUTE) |
| --- | --- | --- | --- | --- | --- | --- | --- | --- | --- | --- |
| ABCB4 | 3.78 |  | EPAG | 2.53 |  | MAGEA9 | 2.7 |  | RNASEN | 2.57 |
| ABCF2 | 2.53 |  | EPO | 2.54 |  | MAGEC2 | 2.62 |  | RNF8 | 2.62 |
| ACCN3 | 2.8 |  | ETV4 | 2.78 |  | MAGOHB | 2.52 |  | RPAP3 | 2.63 |
| ACOX2 | 2.5 |  | FAIM | 2.61 |  | MAN1A2 | 2.55 |  | RPL22 | 2.56 |
| ACRV1 | 2.6 |  | FAM13A1 | 2.67 |  | MAN1A2 | 2.98 |  | SCAMP3 | 2.54 |
| ACSL6 | 2.9 |  | FASTKD2 | 2.52 |  | MAP2K2 | 3.58 |  | SCARB1 | 3.26 |
| ACSM3 | 2.81 |  | FLJ20433 | 2.71 |  | MAP3K4 | 2.67 |  | SCD5 | 2.65 |
| AFF3 | 3.04 |  | FLJ35348 | 2.64 |  | MAP6D1 | 3.29 |  | SDHC | 2.65 |
| ALAD | 2.54 |  | FPR1 | 2.69 |  | MAPK8IP3 | 2.84 |  | SEMA6C | 2.82 |
| ALPI | 2.7 |  | FRMPD1 | 2.63 |  | MAPT | 2.64 |  | SGSH | 2.57 |
| ANKRD2 | 2.9 |  | FURIN | 3.02 |  | MATN4 | 2.68 |  | SH2B2 | 2.63 |
| ANKRD53 | 3.52 |  | FUT9 | 2.73 |  | MCM3 | 2.61 |  | SIM1 | 2.53 |
| ANP32C | 3.5 |  | GARNL1 | 3.23 |  | MED1 | 2.68 |  | SIRT5 | 3.33 |
| APOBEC3G | 2.67 |  | GDF2 | 2.77 |  | METAP2 | 2.58 |  | SLC12A4 | 3.99 |
| ARID5A | 2.69 |  | GFER | 2.63 |  | MFGE8 | 2.87 |  | SLC20A1 | 2.85 |
| ASPH | 2.8 |  | GGTLC1 | 2.77 |  | MFN2 | 2.68 |  | SLC22A11 | 3.83 |
| ATF7IP | 2.61 |  | GIYD1 | 2.53 |  | MINK1 | 2.85 |  | SLC28A2 | 2.59 |
| ATP1A3 | 2.69 |  | GNAT1 | 4.55 |  | MLH3 | 2.63 |  | SLC35E1 | 2.89 |
| ATP4A | 3.49 |  | GNL3L | 2.81 |  | MLX | 2.64 |  | SLCO1C1 | 2.52 |
| ATXN2L | 2.53 |  | GP1BB | 2.68 |  | MLXIPL | 2.88 |  | SLITRK3 | 2.93 |
| BAG2 | 2.77 |  | GPR172A | 2.9 |  | MORC4 | 2.63 |  | SMARCA4 | 2.71 |
| BANF1 | 2.54 |  | GPR77 | 2.59 |  | MOSPD3 | 2.55 |  | SOS1 | 2.8 |
| BAP1 | 2.62 |  | GRAP | 2.52 |  | MPHOSPH9 | 2.56 |  | SPP2 | 2.52 |
| BCL6 | 2.5 |  | GRIA1 | 2.85 |  | MPZL1 | 2.8 |  | SQSTM1 | 3.01 |
| BMP1 | 2.8 |  | GRIN1 | 2.76 |  | MRPS22 | 2.54 |  | SSTR2 | 2.73 |
| BPY2 | 2.89 |  | GRRP1 | 2.65 |  | MS4A1 | 2.53 |  | SSTR3 | 2.6 |
| BRAF | 2.93 |  | GSG1 | 3.08 |  | MSRB2 | 2.68 |  | SSX3 | 2.56 |
| BRPF1 | 2.56 |  | GSTM4 | 2.63 |  | MST1 | 2.72 |  | STARD13 | 2.9 |
| BTBD2 | 2.55 |  | GUCA2A | 2.79 |  | MTM1 | 2.6 |  | STK11 | 2.98 |
| BTNL2 | 2.94 |  | HABP4 | 2.65 |  | MYH3 | 2.56 |  | STK24 | 2.72 |
| C19orf54 | 2.92 |  | HADHA | 2.55 |  | MYL7 | 2.54 |  | SUPT6H | 2.62 |
| C1orf113 | 3.3 |  | HCFC1 | 2.8 |  | MYO15A | 2.87 |  | SWAP70 | 2.66 |
| C3orf36 | 2.76 |  | HERC6 | 2.7 |  | MYOM1 | 2.88 |  | SYN2 | 2.6 |
| CAMK1D | 2.5 |  | HIBCH | 2.59 |  | N4BP2L1 | 3.65 |  | TACR1 | 3.25 |
| CARD10 | 2.86 |  | HIC1 | 3.57 |  | NADK | 2.78 |  | TACR2 | 3.54 |
| CARD14 | 2.93 |  | HIST1H3J | 2.68 |  | NADSYN1 | 2.57 |  | TAF5L | 2.54 |
| CBX1 | 2.72 |  | HMG20B | 2.68 |  | NAT11 | 2.62 |  | TAF9B | 2.55 |
| CCDC21 | 2.66 |  | HNRNPA1 | 2.57 |  | NAT11 | 2.52 |  | TAOK2 | 2.82 |
| CCDC48 | 3.62 |  | HOXA3 | 2.9 |  | NDUFS8 | 2.52 |  | TAS2R4 | 2.8 |
| CCPG1 | 2.58 |  | HOXD11 | 2.54 |  | NEK2 | 2.61 |  | TCP11L1 | 2.78 |
| CCPG1 | 2.51 |  | HRSP12 | 2.59 |  | NF2 | 2.7 |  | TESC | 3.28 |
| CCR10 | 3.33 |  | HSD11B1 | 2.59 |  | NFASC | 3.12 |  | TEX11 | 2.5 |
| CDC25A | 3.59 |  | HTR7P | 3.34 |  | NFKBIL2 | 2.81 |  | TGFB2 | 2.63 |
| CDRT1 | 2.79 |  | HUWE1 | 2.62 |  | NOLA1 | 2.6 |  | TGM4 | 2.68 |
| CDYL | 2.58 |  | HYMAI | 2.56 |  | NPAT | 2.67 |  | TLK1 | 2.66 |
| CENTD1 | 2.56 |  | IFNA21 | 2.95 |  | NPHS1 | 2.55 |  | TLN2 | 3.39 |
| CHMP1B | 2.58 |  | IGFALS | 3.05 |  | NPTXR | 3.04 |  | TMEM144 | 2.68 |
| CHRNE | 3.26 |  | IKBKG | 2.6 |  | NR1D1 | 3.22 |  | TMEM57 | 2.52 |
| CIAPIN1 | 2.55 |  | IL1RAPL1 | 2.82 |  | OR12D3 | 2.52 |  | TMOD2 | 2.71 |
| CLCN7 | 2.66 |  | IL1RN | 2.79 |  | ORC4L | 2.65 |  | TNRC4 | 2.54 |
| CNPY4 | 2.65 |  | IL6ST | 2.55 |  | OXCT2 | 2.52 |  | TNS1 | 2.8 |
| CNTLN | 2.59 |  | IL7 | 3.34 |  | PART1 | 2.53 |  | TOX | 3.01 |
| COL15A1 | 2.94 |  | INCENP | 3.71 |  | PBX2 | 2.62 |  | TRIM10 | 2.97 |
| COL4A3 | 2.61 |  | KCNA6 | 3.22 |  | PCDHGC3 | 2.87 |  | TRIM23 | 2.56 |
| CPLX3 | 2.5 |  | KIAA0515 | 2.75 |  | PDE4A | 2.54 |  | TRIP11 | 2.57 |
| CREBL1 | 2.77 |  | KIAA0892 | 2.53 |  | PDIA2 | 4.52 |  | TSC1 | 2.56 |
| CRIP1 | 2.69 |  | KIF4A | 2.82 |  | PHF20 | 2.9 |  | TSR1 | 2.66 |
| CTSL2 | 2.51 |  | KLF8 | 2.83 |  | PHF8 | 2.57 |  | TTC38 | 2.69 |
| CUGBP1 | 2.69 |  | LAMA1 | 2.71 |  | PIGH | 2.5 |  | TXNDC3 | 2.79 |
| DES | 2.92 |  | LARGE | 2.89 |  | PIGL | 2.65 |  | TYMS | 2.71 |
| DHPS | 2.55 |  | LECT1 | 3.86 |  | PIK3CD | 2.56 |  | UBA6 | 2.54 |
| DIS3 | 2.68 |  | LEF1 | 2.63 |  | PLK3 | 2.83 |  | UBE2H | 2.67 |
| DKKL1 | 2.73 |  | LOC100129015 | 3.74 |  | POLE2 | 3.03 |  | UNC119 | 2.97 |
| DNAJB12 | 2.51 |  | LOC100129141 | 3.03 |  | POU6F1 | 3 |  | UTP14A | 2.55 |
| DNAJC2 | 2.64 |  | LOC100130829 | 2.63 |  | PPAP2B | 2.9 |  | VPS13A | 2.63 |
| DNASE1L2 | 2.63 |  | LOC100131795 | 2.99 |  | PPIL6 | 3.11 |  | WNK1 | 2.57 |
| DOCK9 | 2.81 |  | LOC100134363 | 2.55 |  | PPP4C | 2.5 |  | WNT10B | 3.08 |
| DTNB | 2.8 |  | LOC196993 | 3.32 |  | PRO1880 | 2.54 |  | XAGE1A | 2.55 |
| DUSP26 | 2.66 |  | LOC222070 | 2.5 |  | PTCRA | 2.65 |  | YOD1 | 3.06 |
| DUSP9 | 2.89 |  | LOC388796 | 2.54 |  | PTGS1 | 2.99 |  | ZFR | 2.94 |
| E2F3 | 2.76 |  | LOC653188 | 2.64 |  | PTPRB | 3.46 |  | ZMAT5 | 2.53 |
| EBI3 | 3.19 |  | LOH3CR2A | 2.54 |  | PXMP4 | 2.71 |  | ZMYM2 | 2.59 |
| EDA | 3.52 |  | LPPR2 | 2.53 |  | RBM14 | 2.52 |  | ZNF117 | 2.8 |
| EGFR | 2.74 |  | LRRC31 | 2.69 |  | RBM19 | 3.31 |  | ZNF187 | 2.56 |
| EGO | 2.66 |  | LRRC41 | 2.79 |  | RBMS2 | 2.58 |  | ZNF44 | 2.62 |
| EHMT1 | 3.64 |  | LY6E | 2.51 |  | RGS10 | 2.57 |  | ZNF646 | 2.82 |
| EIF3B | 2.54 |  | LYK5 | 4.11 |  | RMND5A | 2.78 |  | ZNF770 | 2.51 |
| ENDOD1 | 2.86 |  | MAFF | 2.54 |  | RNASE4 | 2.56 |  | ZNF93 | 2.72 |
