# Supplementary material for: Suppressed Expression of T-Box Transcription Factors Is Involved in Senescence in Chronic Obstructive Pulmonary Disease
Source: PLoS Comput Biol. 2012 Jul 19;8(7):e1002597. doi: 10.1371/journal.pcbi.1002597 (PMC3400575; doi:10.1371/journal.pcbi.1002597)
Supplement: Table S4 — A: Membership of Five Biclusters Learned Using Factor Analysis for Bicluster Acquisition (FABIA). This table identifies the genes and phenotypes that clustered together when the algorithm was applied to learn five biclusters from the experiments in the compendium. B: Membership of Ten Biclusters Learned Using Factor Analysis for Bicluster Acquisition (FABIA). This table identifies the genes and phenotypes that clustered together when the algorithm was applied to learn ten biclusters from the experiments in the compendium. C: Membership of Twenty Biclusters Learned Using Factor Analysis for Bicluster Acquisition (FABIA). This table identifies the genes and phenotypes that clustered together when the algorithm was applied to learn twenty biclusters from the experiments in the compendium. (DOC) [file pcbi.1002597.s008.doc]

**Table S4A. Membership of *Five* Biclusters Learned Using Factor Analysis for Bicluster Acquisition (FABIA)**

| **BICLUSTER NUMBER** | **BICLUSTER GENES** | **BICLUSTER PHENOTYPES**  *BAL or **Smoking Status |
| --- | --- | --- |
| **1** | AAAS AASDHPPT ABAT ABCB7 ABCB9 ABCE1 ABCG1 ABHD5 ABI1 ACAA1 ACACB ACAP1 ACAT2 ACBD3 ACO2 ACOT7 ACOX1 ACSL4 ACSM5 ACTA1 ACTR6 ACTR8 ADAM11 ADAM28 ADARB1 ADCK3 ADCY3 ADCY7 ADCY8 ADD2 ADH5 ADNP2 ADRBK1 ADSL AGA AGFG2 AGGF1 AGK AGPS AGRN AHCTF1 AIDA AIMP1 AKAP1 AKAP7 AKAP8L AKT3 ALDH1A2 ALDH5A1 ALG12 ALG13 ALKBH4 ALLC ALOX15B ALOX5 AMD1 AMIGO2 AMN ANAPC10 ANGEL2 ANK1 ANKLE2 ANKRD10 ANKRD12 ANKRD2 ANKRD27 AP1AR AP1S1 APBB1 APC APIP APOA4 APOBEC2 APOBEC3G APOC4 APOL2 APOOL APPL1 APTX AQP7 AREG ARFGEF1 ARFGEF2 ARFRP1 ARHGAP33 ARHGAP5 ARHGAP6 ARHGEF12 ARID1A ARID4B ARID4B///RBM34 ARIH1 ARIH2 ARMC1 ARMC6 ARMC8 ARMCX1 ARMCX3 ARPP19 ARSA ARSF ARVCF ASB6 ASF1A ASGR2 ASPM ATAD2B ATF1 ATF2 ATF6B ATG12 ATG14 ATIC ATMIN ATP2A3 ATP2B1 ATP2C1 ATP4A ATP5S ATP7A ATP9A ATP9B ATR ATRX ATXN2L ATXN3 AVL9 AVP AZI2 B3GALNT1 B3GNT3 BACH1 BAZ1A BAZ1B BAZ2B BBC3 BBS7 BCAR3 BCAS2 BCAT2 BCL2 BCL2L1 BCOR BEST1 BET1 BLNK BLZF1 BMP1 BMP2K BNIP1 BNIP2 BOLA2///LOC440354///LOC595101 BPY2 BRD7P3 BTG2 BTNL3 BUB3 BUD31 C10orf137 C10orf18 C12orf11 C12orf47 C12orf5 C14orf106 C14orf138 C14orf139 C14orf147 C15orf2 C17orf53 C17orf91 C18orf1 C19orf2 C19orf42 C1D C1orf103 C1orf107 C1orf109 C1orf144 C1orf156 C1orf174 C1orf25 C1orf27 C20orf117 C21orf2 C21orf7 C22orf29 C2orf3 C2orf83 C3 C3orf36 C3orf64 C4orf41 C5orf13 C5orf44 C6orf120 C6orf15 C6orf211 C7orf64 C8orf44///SGK3 C9orf95 CA2 CACNA1I CACNG4 CACYBP CALD1 CAMK2B CAMKV CAMSAP1 CAMSAP1L1 CAPZA2 CARD10 CARD14 CARD8 CASP6 CASP8AP2 CAV2 CBL CBWD1///CBWD2///CBWD3///CBWD5///CBWD6///CBWD7///LOC100507355 CBX1 CCDC134 CCDC22 CCDC28B CCDC48 CCDC59 CCDC76 CCDC85B CCDC90A CCDC91 CCDC93 CCKAR CCL1 CCL21 CCNA2 CCNF CCNG2 CCNT2 CCR10 CD164 CD22 CD24 CD2AP CD320 CD40 CD44 CD6 CD68 CD69 CDA CDC23 CDC25A CDC25C CDC27 CDC42BPA CDC42BPB CDC42EP2 CDC42EP3 CDC42EP4 CDC73 CDCP1 CDH15 CDH4 CDK13 CDK14 CDK17 CDK7 CDK8 CDKN2C CDKN2D CEACAM3 CEACAM8 CEBPG CEBPZ CECR5 CELF2 CELF3 CELP CENPN CENPO CEP135 CEP170 CEP57 CEP76 CES1P1 CETN3 CETP CFB CHCHD7 CHD9 CHERP CHMP2B CHRD CHRM5 CHRNE CHST8 CHUK CIDEA CKLF CLASP2 CLCA1 CLCC1 CLCF1 CLCN1 CLCN3 CLCN4 CLCN7 CLDN6 CLIC4 CLIP4 CLK1///PPIL3 CLK4 CLN5 CLPX CNIH4 CNPY4 CNR1 COIL COL11A2 COL13A1 COL15A1 COLEC12 COPE COPS2 COPS8 COQ10B COX11 COX15 CP CPE CPEB3 CPLX3///LMAN1L CPNE6 CPNE7 CPOX CPSF1 CPT2 CR1 CRABP2 CREBL2 CREBZF CREM CRHR1 CRIM1 CRIPT CRLF3 CRMP1 CRNKL1 CRTAM CRX CRYBB3 CRYGA CRYZ CSAD CSE1L CSGALNACT2 CSH2 CSNK1G1 CSNK1G2 CSTF1 CSTF2T CTAGE9 CTF1 CTRB1///CTRB2 CUL2 CUL3 CUL4A CUL5 CXCL12 CXCL5 CXCR3 CXCR5 CXCR7 CYB561 CYP11B2 CYP17A1 CYP20A1 CYP2C9 CYP4F12 DAG1 DAPK1 DAPK2 DARS DBR1 DCAF15 DCAF6 DCK DCN DCP2 DCUN1D4 DDIT3 DDN DDR1 DDR2 DDX17 DDX18 DDX24 DDX46 DEDD DEGS1 DENND4A DERL1 DES///FAM48A DHRS12 DHX29 DKKL1 DLG4 DMXL1 DMXL2 DNAJB14 DNAJB9 DNAJC13 DNAJC24 DNAJC6 DNAJC9 DNASE1L2 DNM1 DNM2 DOCK10 DOHH DOPEY1 DPF1 DPY30///MEMO1 DPYD DPYSL2 DR1 DRD2 DSC2 DST DUT DUX1 DVL2 DYNC1LI1 DYRK1B E2F3 EBAG9 EBI3 ECE1 ECM1 EDA EDEM3 EDNRB EEA1 EED EFEMP2 EGLN1 EGOT EHMT1 EHMT2 EIF1AX EIF2C4 EIF3A EIF3J EIF4E EIF4ENIF1 ELAVL3 ELF2 ELF3 ELK1 ELMO2 EMILIN1 ENOSF1 ENOX2 ENPP4 EP300 EPAS1 EPB41L3 EPB41L4A EPB49 EPHB4 EPN1 EPO EPOR EPS15 EPS8L3 EPX ERAP1 ERAP2 EREG ERI2 ESF1 ESR1 ESRP2 ETNK1 ETV4 ETV7 EVI5 EVX1 EXOSC10 EXOSC8 EZR F12 FABP2 FAIM3 FAM115A FAM116B FAM134A FAM13B FAM153A FAM178A FAM49A FAM55C FAM63B FAM70A FASLG FASTKD1 FASTKD3 FBLN1 FBN1 FBXO11 FBXO24 FBXO28 FBXO38 FBXO5 FCN2 FDX1 FECH FEM1B FEM1C FEZ2 FGB FGF21 FGF6 FGL2 FH FKBP15 FKBP3 FKBP8 FKSG49 FLI1 FLVCR2 FMOD FMR1 FNDC3A FOXC2 FOXE1 FOXN1 FOXO4 FOXRED2 FPGT FRY FRYL FSCN3 FSTL3 FUBP1 FUBP3 FURIN FUS FUT1 FUT7 FXR1 FZD5 FZD9 GABARAPL1///GABARAPL3 GADD45B GALNT1 GALNT11 GAPVD1 GART GAS6 GATAD1 GBE1 GCAT GCFC1 GCN1L1 GCSH///LOC100329108 GDF2 GDPD2 GFER GGTLC2 GIMAP6 GJA8 GJC2 GJD2 GLI1 GLP1R GLRX2 GLS GLT8D2 GLYR1///SEPT6 GMFB GNAT1 GNL3 GNL3L GNPTAB GOLGA8H GOLM1 GOLT1B GP1BB GPN3 GPR135 GPR137 GPR144 GPR162 GPR25 GPR31 GPR89A///GPR89B///GPR89C GPRC5B GRAP2 GRIK5 GRIN1 GRK1 GRK5 GRM7 GRSF1 GSK3A GSPT1 GSPT2 GTF2H2///GTF2H2B///GTF2H2C///GTF2H2D GTF3A GTPBP4 GUCA2A GYG1 H2AFX H3F3A///LOC440926 H6PD HAB1 HABP4 HAND2 HAUS3 HAUS5 HAX1 HBEGF HBP1 HCCS HCFC1 HDAC10///LOC100509694///MAPK12 HDAC2 HDAC4 HDAC7 HDHD3 HDLBP HEATR1 HEATR3 HERC2 HERC2P2///HERC2P9 HEYL HIBCH HIC1 HIC2 HIF3A HIST1H1D HIST1H2BI HLA-G HMG20A HMGA1 HMGCR HMGN4 HMX1 HNF1B HNRNPA0 HNRNPD HNRNPH3 HNRNPUL2 HPCA HPGD HPN HRASLS2 HRK HS2ST1 HSDL2 HSF4 HSP90B1 HSPA13 HSPA4 HTR2C HTR4 HTR6 HUS1 IBTK ICAM1 ID2///ID2B IFI16 IFIH1 IFIT5 IFITM1 IFNA17 IFNA21 IFRD1 IGFALS IGFBP3 IGHA1///IGHA2///IGHD///IGHG1///IGHG3///IGHM///IGHV4-31///IGHV4-59///LOC100126583 IGLV2-18///IGLV3-19///LOC100290481 IGLV2-23 IGSF6 IHH IKBKG IL13RA1 IL15 IL18BP IL1F6 IL1RL2 IL1RN IL7 IMMT IMPACT INO80B INPP5F INS INSIG1 INSIG2 INTS6 IPO5 IPO7 IRAK3 IREB2 ISCA1 ISOC1 ITGA4 ITGA7 ITGB1BP1 ITGB3 ITGB4 ITPR1 ITPR2 ITPR3 ITSN1 ITSN2 IVD JAK2 KAT2B KCNA6 KCNAB1 KCNC1 KCNC2 KCNJ5 KCNN3 KCTD17 KCTD9 KDM2A KDM3A KDM4A KDM6B KDSR KEL KHDRBS2 KHSRP KIAA0020 KIAA0240 KIAA0317 KIAA0368 KIAA0562 KIAA0776 KIAA0913 KIAA0947 KIAA1033 KIAA1109 KIAA1598 KIF16B KIR2DL2 KIR2DS5 KL KLC1 KLF10 KLF9 KLHDC10 KLHL2 KLHL20 KLK1 KMO KPNA2 KPNA4 KRAS KREMEN2 KRIT1 KRR1 KRT19 KRTAP5-9 L3MBTL1 LACTB2 LAMA1 LAMA2 LAMB1 LARP4 LASS6 LBR LCN2 LDB1 LECT1 LEF1 LEPR LETM1 LFNG LGALS2 LGI2 LGMN LILRA5 LIMD2 LIMK1 LIN7C LIPE LMNA LOC100129503 LOC100133321 LOC100288679 LOC100288939 LOC100499177 LOC100505650 LOC100506002 LOC100506168 LOC100506168///SFPQ LOC100506517 LOC100506935 LOC100507328///LOC100508591 LOC100508624 LOC100510692///NAIP LOC150776///SMPD4 LOC220594 LOC254896///TNFRSF10C LOC642869///SET LOC80054 LOC91316 LOX LPGAT1 LPHN1 LPL LPO LRCH3 LRCH4 LRCH4///SAP25 LRP12 LRP3 LRP6 LRRC23 LRRC40 LSM14A LSM5 LTA LTK LTN1 LUC7L3 LY75 LYST LZTS1 MAF MAK16 MAN1A1 MAN1A2 MANEA MAP2K2 MAP2K3 MAP2K5 MAP2K7 MAP3K10 MAP3K5 MAP3K7 MAP3K8 MAP3K9 MAP4K3 MAP4K4 MAP4K5 MAP6D1 MAP7 MAPK8 MAPK8IP1 MAPK8IP2 MAPK8IP3 MAPK9 MAPRE3 MAPT MARS MASP2 MAST2 MATK MBD2 MBD4 MBIP MBNL1 MBTPS1 MC1R MCFD2 MDC1 MDFIC ME1 MED13L MED14 MED17 MED23 MED4 MED6 MED7 MEF2A MEF2D MEPE METTL13 METTL2A///METTL2B METTL3 MEX3D MFF MFHAS1 MFN1 MFNG MGAT2 MGAT4A MICALL1 MINK1 MINPP1 MKI67 MLL4 MLXIPL MMD MME MMP14 MMP25 MOAP1 MOBKL3 MOCS2 MOCS3 MORC3 MOSPD1 MPHOSPH8 MPHOSPH9 MPZ MRC1///MRC1L1 MRC2 MRPL13 MRPL41 MRPS30 MRPS31 MRS2 MS4A4A MSMB MSR1 MSRB2 MT1G MT1P2 MT2A MTA1 MTA2 MTAP MTDH MTF2 MTHFD2 MTHFS MTIF2 MTMR6 MTO1 MTR MTRF1 MTX2 MUC16 MUC2 MUC3A MUC3B MUC4 MUC5B MXD4 MYCBP2 MYL10 MYL7 MYO6 MYO7A MYO9A MYOG MYST4 N4BP1 N4BP2L1 N4BP2L2 NAA16 NAA40 NACC2 NAP1L1 NARG2 NCOA2 NCOR2 NCR3 NDRG2 NDUFAF1 NEK7 NF2 NFATC2IP NFKB2 NGB NGLY1 NHLRC2 NID1 NINJ1 NIPAL3 NIPBL NKX3-1 NMD3 NNT NOC3L NOL11 NOP2 NOP56 NPAT NPC1 NPEPL1 NPEPPS NPHP4 NPHS1 NR2C1 NR2F6 NR3C1 NR4A2 NR4A3 NRIP1 NRP1 NSUN3 NUBPL NUDCD3 NUDT7 NUMB NUP133 NUP160 NUP205 NUP43 NUP54 NUPL1 NUSAP1 NXT2 NYX OBFC2A OGFR OGT OLR1 OPA1 OPN3 OR1A1 OR2A20P///OR2A9P OSBPL10 OSBPL1A OSBPL8 OSTM1 OTOR OXCT2 OXR1 PAFAH1B1 PAK1IP1 PAK2 PAN2 PANK3 PAPOLA PAPOLG PAPSS2 PAQR3 PARG PARVA PATZ1 PAX8 PAXIP1 PBX2 PCDH1 PCDHA10 PCF11 PCGF2 PCMT1 PCNA PCOLCE PCOLCE2 PCTP PDCD11 PDCD2 PDE1B PDE3B PDE4A PDE4B PDE4DIP PDE6G PDHA1 PDHX PDIA2 PDK2 PDK4 PDLIM7 PDSS2 PDZD7 PEX3 PEX5 PFDN4 PFKFB3 PGF PGRMC2 PHF1 PHF15 PHF20 PHKA2 PHLDA1 PHTF2 PHYH PI3 PIAS1 PIAS2 PICALM PIGO PIGR PIK3C2A PIK3CB PIK3CD PIK3R2 PIKFYVE PION PIP5K1A PITRM1 PKD1 PKD2 PKIA PKLR PKN2 PLAA PLAGL1 PLCD1 PLEKHM1 PLSCR3 PMPCB PNLIP PNMA3 PNN PNPLA2 POFUT2 POLD3 POLDIP3 POLE POLG POLI POLR3G PORCN POT1 POU3F1 PPAN PPAP2A PPCS PPEF2 PPFIA1 PPIG PPIL6 PPIP5K2 PPM1B PPM1D PPP1R12A PPP1R2P9 PPP2CB PPP2R1B PPP2R3C PPP3R1 PPWD1 PRDM10 PRDM14 PREPL PRKACB PRKACG PRKAR2B PRKD3 PRKRIR PRLH PRNP PROC PROCR PROM1 PRPF3 PRPF38B PRPF39 PRRG1 PRSS53 PSD PSD3 PSENEN PSMC6 PSME4 PSMF1 PSPC1 PTAFR PTBP2 PTCRA PTEN PTGDS PTK2 PTP4A1 PTPLA PTPLB PTPN12 PTPN2 PTPN22 PTPRA PTPRC PTPRN2 PTPRO PURA PWP1 PYROXD1 PYY2 QKI R3HDM1 RAB11B RAB11FIP2 RAB22A RAB4A///SPHAR RAB5A RAB7A RAB7L1 RABEP2 RABGAP1 RABGAP1L RABGGTB RAD17 RAD51AP1 RAI1 RALA RALGAPA1 RANBP6 RAP2C RARA RARB RASGRF1 RASGRP2 RB1 RB1CC1 RBBP6 RBCK1 RBM16 RBM22 RBM25 RBM8A RBMS1 RBP4 RC3H2 RCAN1 RCC1 RCE1 RCHY1 RCOR1 RDH14 RDX RECK RECQL RECQL5 REST REV1 REV3L RFC4 RFPL3S RFTN1 RGS1 RGS16 RGS6 RHEB RHOQ RHOT1 RIF1 RIOK3 RND2 RNF139 RNF14 RNF170 RNF216 RNF219 RNF38 RNMT ROBO4 ROCK2 ROM1 RP2 RPAP3 RPLP0 RPRD1A RPS6KB1 RRAGD RREB1 RRM1 RRM2 RRN3 RRN3///RRN3P1///RRN3P2 RRP15 RRP1B RRP7A///RRP7B RSF1 RTCD1 RTF1 RTN1 RWDD2A SAA1///SAA2 SAC3D1 SACM1L SACS SAMM50 SAMSN1 SAP30 SAR1A SART3 SCAMP1 SCML1 SCN2B SCT SCYL3 SDCCAG1 SDCCAG3 SDK2 SEC14L2 SEC23A SEC24B SEC24D SEC62 SEH1L SEL1L SEMA5A SEMA6A SEMA6C SENP6 SEPT10 SEPT11 SERBP1 SERP1 SERPINB3 SERPINB4 SERPINI1 SETD1A SETD2 SF3A3 SFRS18 SFTPC SGMS1 SGPP1 SH3BP1 SIGLEC6 SIGLEC8 SIGMAR1 SIK3 SILV SIRT1 SLC12A4 SLC12A9 SLC13A2 SLC19A2 SLC1A3 SLC1A6 SLC22A11 SLC22A6 SLC25A11 SLC25A17 SLC25A32 SLC25A37 SLC28A1 SLC31A1 SLC33A1 SLC38A6 SLC39A2 SLC39A6 SLC39A8 SLC44A4 SLC4A1AP SLC4A7 SLC5A3 SLC6A2 SLCO4A1 SLPI SMAD2 SMAD3 SMAD4 SMAD6 SMARCA4 SMARCA5 SMARCD1 SMC1A SMC2 SMC3 SMC4 SMCHD1 SMNDC1 SMOX SMPD3 SMURF2 SNRNP70 SNTA1 SNX16 SOAT1 SOCS3 SOCS5 SOCS6 SOS1 SOX10 SOX15 SP2 SP3 SPARC SPAST SPATA5L1 SPCS3 SPG20 SPOCK2 SPOP SPTAN1 SPTBN5 SQSTM1 SR140 SRBD1 SRCAP SRP19 SRP19///ZRSR1 SRP72 SRPK2 SRSF1 SRSF10 SRSF11 SRSF3 SRSF7 SSB SSTR2 SSTR3 ST3GAL4 ST3GAL6 ST6GALNAC4 ST7 ST8SIA2 STAG1 STAG3L4 STAM STAM2 STAMBP STC1 STK3 STK38 STRN3 STS STX4 STX7 STXBP3 SUB1 SUPT7L SVIL SWAP70 SYNC SYNCRIP SYNJ1 SYNJ2 SYNRG TAB2 TACC1 TACR1 TACR2 TACSTD2 TAF1 TAF11 TAF1D TAF2 TANK TAOK2 TARDBP TAX1BP1 TAZ TBC1D30 TBC1D4 TBCCD1 TCEB1 TCEB2 TCERG1 TCF12 TCF15 TCF3 TCL6 TDG TEK TET3 TEX10 TEX11 TEX13A TEX14 TFAM TFEC TFR2 TG TGM4 THBD THBS1 THUMPD1 TIA1 TIAL1 TIAM1 TIGD1L TIMM17A TK2 TLK1 TLK2 TLN2 TLR7 TM2D1 TMBIM1 TMED5 TMED7 TMEFF1 TMEM106B TMEM38B TMEM59L TMEM63A TMF1 TMOD1///TSTD2 TMOD3 TMPRSS15 TMPRSS2 TMX4 TNFRSF10B TNFRSF12A TNFRSF13B TNFRSF21 TNK1 TNK2 TNPO1 TNPO2 TNS1 TNS3 TNXA///TNXB TOP3A TOPBP1 TOPORS TOR1AIP1 TP53 TPK1 TPM1 TPMT TPP2 TPPP TPPP3 TPSAB1 TRAF2 TRAPPC10 TRAPPC2 TRBC1///TRBC2 TREX1 TRHDE TRIM10 TRIM15 TRIM29 TRIM36 TRIM38 TRIM5 TRIM52 TRIM62 TRMT11 TRMT61A TROAP TROVE2 TSC1 TSC22D4 TSEN2 TSFM TSN TSPAN1 TSPAN6 TSPY1 TTC3 TTC33 TTC37 TTC38 TTF2 TTLL3 TUBGCP2 TUBGCP3 TULP4 TXNDC15 TXNDC9 UBE2B UBE2D1 UBE2K UBE2V2 UBE2W UBE3B UBR5 UBXN2B UBXN4 UCHL5 UCP3 UEVLD UFD1L UGCG UHRF1BP1L ULK2 UNC119 UNG UPF3A UPF3B USO1 USP1 USP33 USP34 USP8 UTP3 VAMP4 VCAN VCY///VCY1B VIPR2 VNN3 VPS13A VRK1 WAPAL WBP4 WDR11 WDR33 WDR37 WDR44 WDR62 WDR67 WFDC2 WIPF1 WIPI1 WIZ WNT10B WNT6 WSB1 WSB2 WWP1 XAGE1A///XAGE1B///XAGE1C///XAGE1D///XAGE1E XDH XK XPOT YBX1///YBX1P2 YTHDC2 ZBP1 ZBTB1 ZBTB10 ZBTB11 ZBTB17 ZC3H13 ZC3H15 ZC3H7A ZCCHC10 ZDHHC11 ZDHHC17 ZEB2 ZFAND1 ZFP112 ZFP30 ZFP36L1 ZFR ZFYVE16 ZMYM4 ZMYND11 ZMYND8 ZNF124 ZNF131 ZNF140 ZNF143 ZNF157 ZNF184 ZNF217 ZNF226 ZNF235 ZNF292 ZNF302 ZNF318 ZNF330 ZNF335 ZNF419 ZNF45 ZNF451 ZNF468 ZNF493 ZNF518A ZNF571 ZNF623 ZNF646 ZNF673 ZNF771 ZNF83 ZNF84 ZNHIT3 ZNHIT6 ZZZ3 | BAL_26_A2B0 BAL_17_A0B0 BAL_39_A0B1 BAL_40_A0B0 BAL_13_A0B0 BAL_3_A1B0 BAL_27b_A2B1 BAL_44b_A0B0 BAL_4_A0B0 BAL_45a_A1B0 BAL_25_A0B0 BAL_43_A0B0 BAL_18_A1B0 BAL_33_A0B1 BAL_15a_A0B1 BAL_47b_A0B1 BAL_45b_A2B0 BAL_37_A1B1 BAL_12_A1B0 BAL_1_A0B0 BAL_27a_A0B0 BAL_20b_A1B1 BAL_42_A0B0 BAL_47a_A0B0 BAL_21_A0B0 BAL_46_A2B0 BAL_47c_A0B1 BAL_29_A1B0 BAL_31_A0B1 BAL_15b_A0B0 BAL_41_A1B0 BAL_19_A0B0 BAL_44a_A0B0 |
| **2** | AACS ABCB6 ABCC10 ABCF2 ABI1 ABL1 ABLIM1 ABR ACACB ACE ACLY ACOX3 ACP1 ACSL1 ACSL3 ACTB ACTG1 ACTN4 ACTR2 ADAM10 ADCY9 ADD1 ADD3 ADRA2A ADRBK1 AGL AGR2 AGRN AHNAK2 AIDA AK1 AKAP1 AKAP17A AKR1B10 AKR1C1 AKR1C2 AKR7A2 ALCAM ALDH3A1 ALDH3A2 ALDH9A1 ALG13 ALMS1 ALOX5 ALOX5AP AMMECR1 AMY1A///AMY1B///AMY1C///AMY2A///AMY2B ANAPC5 ANK3 ANKHD1///ANKHD1-EIF4EBP3 ANKRD28 ANP32A ANXA1 ANXA4 ANXA5 ANXA7 AP3M2 APLP2 APOB48R APOC2 APOE APP APRT APTX AQP3 ARF1 ARF3 ARF4 ARF6 ARFGEF1 ARFIP1 ARFRP1 ARGLU1 ARHGDIA ARL1 ARL3 ARL6IP4 ARL6IP5 ARPC2 ARPC4 ARPC5L ASMTL ASPH ATP13A3 ATP2B1 ATP5D ATP5E ATP5I ATP5O ATP6AP2 ATP6V0E1 ATP6V1A ATP6V1B2 ATP6V1C1 ATP6V1G1 ATP8B1 ATP9A ATP9B ATPIF1 AZGP1 B2M B3GALT4 B3GNT2 B4GALT7 BAG1 BAIAP2 BCAS1 BCL2A1 BCL2L1 BCL3 BCL6 BHLHE40 BLCAP BNIP3 BNIP3L BRAP BRD3 BRD8 BSCL2 BSG BTBD1 BTBD3 BTF3 BTG2 BUB3 BUD31 BZW2 C10orf116 C10orf26 C10orf81 C11orf10 C11orf2 C11orf48 C11orf67 C14orf1 C14orf147 C14orf2 C15orf63///SERF2 C17orf101 C18orf1 C18orf10 C19orf2 C19orf22 C19orf42 C19orf6 C19orf60 C1orf106 C1orf116 C1orf144 C1orf25 C1orf38 C1orf63 C1QA C1QB C20orf12 C20orf30 C21orf33 C22orf28 C4orf46///TOMM7 C6orf103 C6orf106 C6orf130 C6orf62 C7orf25///PSMA2 C9orf91 CALM1 CALR CALU CANT1 CANX CAPN3 CAPN7 CAPN9 CAPRIN1 CAPZB CARS CASC1 CASP6 CAT CCDC19 CCDC69 CCDC72 CCL18 CCL20 CCL4 CCND1 CCNG2 CCNL2 CCPG1 CCR1 CCT2 CCT3 CCT6A CD14 CD163 CD164 CD24 CD37 CD46 CD47 CD52 CD53 CD55 CD59 CD74 CD9 CD99 CDC16 CDC42EP3 CDC5L CDH1 CDK11A///CDK11B CDK5RAP3 CDKN2C CDS1 CDV3 CEACAM1 CEACAM6 CEL///LOC100508206 CEP290 CEP57 CEP68 CES2 CETN3 CFDP1 CFH CFLAR CHD8 CHERP CHL1 CHMP1A CHMP1B CHMP4A CHST11 CHST12 CIRBP CISD1 CITED2 CKAP4 CLCA2 CLDN8 CLINT1 CLK3 CLMN CLPTM1 CLU CMAH CNOT2 CNOT8 COBRA1 COL16A1 COL21A1 COMMD10 COMT COPB1 COPS8 COX16 COX4I1 COX5A COX5B COX6B1 COX7A2 CPD CREB1 CREM CRK CROCCP2 CROT CSDA CSDE1 CSE1L CSF2RB CSF3R CSNK1A1 CSNK1G2 CSPP1 CSTF2T CTBP2 CTDSPL CTSB CTSC CTSS CTSZ CTTN CUL1 CUL5 CUTA CWF19L1 CXCL2 CXCL5 CXCR4 CYBA CYBB CYP2B6///CYP2B7P1 CYP2F1 CYP4B1 CYP51A1 CYTH1 DAAM1 DAB2 DAPK3 DAPP1 DAZAP2 DBT DCAF11 DCAF15 DCI DCLRE1C DCTD DCXR DDA1 DDAH1 DDX18 DDX19A///DDX19B DDX27 DDX27///SS18 DDX3X DENR DGCR2 DGCR6///DGCR6L DGCR8 DHCR24 DHRS3 DHRS9 DHX15 DHX30 DIAPH2 DICER1 DKFZP586I1420 DLG5 DNAH9 DNAI2 DNAJB1 DNAJB2 DNAJB6///TMEM135 DNAJC10 DPM3 DRG2 DROSHA DSP DSTYK DUSP1 DUSP22 DUSP6 DUT DVL1 DYNC2LI1 DYRK2 DZIP3 EBNA1BP2 ECHS1 EDF1 EEF1D EFCAB2 EFEMP1 EFHC1 EFS EGLN2 EID1 EIF1 EIF2B4 EIF3B EIF3C///EIF3CL EIF3K EIF3L EIF5A EIF5B ELF3 ELK3 EML4 EMR2 ENDOG ENO1 ENPP4 ENTPD6 EPAS1 EPCAM EPHX1 EPS8L2 ERCC1 ERLIN1 ERLIN2 ETFB ETS2 EXOC3 EXOC7 EXOSC2 EZH1 EZR FABP4 FABP6 FAHD2A FAM134A FAM134C FAM136A FAM149A FAM162A FAM21A///FAM21B///FAM21C///FAM21D FAM35A FARS2 FASTK FBL FBXO11 FBXO9 FBXW12 FCER1G FCGBP FCGR2A FCGR3A///FCGR3B FCGR3B FDFT1 FDXR FFAR2 FGFR3 FHL1 FKBP11 FKBP1A FKBP2 FLII FLJ10038 FLNA FLOT2 FN1 FNBP1L FNDC3B FOS FOSL2 FOXO3 FOXO3///FOXO3B FPR1 FPR2 FUS FXYD3 G0S2 G3BP2 GADD45B GAK GALK2 GAPDH GAPVD1 GAR1 GBP1 GCN1L1 GCSH///LOC100329108 GDE1 GDI1 GDI2 GGA2 GIPC2 GK GLIPR1 GLRX GLT8D1 GLTSCR2 GLUD1 GLUD2 GLUL GM2A GNA11 GNAI2 GNAI3 GNAQ GNAS GNB1 GNB2 GNS GOLGA1 GOLGA6L5///GOLGA6L9 GOLGA8A///GOLGA8B GOLGA8H GOLGB1 GORASP2 GOSR1 GPM6B GPN1 GPNMB GPR56 GPX3 GRSF1 GSN GSS GSTA1 GTF2H1 GTF2H3 GTF2I///GTF2IP1///LOC100093631 GTF3C1 GTF3C2 GTPBP8 GULP1 H2AFX H2AFY H2BFS H3F3B HADHA HAUS2 HCK HDLBP HERC6 HEY1 HFE HIBCH HIF1AN HK3 HLA-B HLA-C HLA-DPA1 HLA-DQA1///HLA-DQA2 HLA-DQB1 HLA-DQB1///LOC100133583 HLA-DRA HLA-DRB1///HLA-DRB3///HLA-DRB4 HLA-DRB1///HLA-DRB4///HLA-DRB5 HLA-DRB4///LOC100509582 HLA-G HMGN3 HN1L HNRNPC HNRNPF HNRNPH1 HNRNPU HOMER1 HPS1 HRAS HSBP1 HSD17B7 HSP90AA1 HSP90AB1 HSP90B1 HSPA1A HSPA8 HSPB11 HSPD1 HSPH1 ICAM1 IDE IFI30 IFITM2 IFITM3 IFNGR1 IFT20 IGF1R IGF2R IGFBP2 IGFBP5 IGFBP7 IL1B IL1R2 IL1RN IL2RG IL7R IL8 ILKAP IMPDH2 INADL INHBA INSR INTS3 INTS5 IPO5 IPP IQCE IQGAP1 ISL1 ITGAM ITGB2 ITGB4 ITM2B IVNS1ABP JAG1 JUND KANK1 KARS KAZ KBTBD4///PTPMT1 KDELR1 KDELR2 KDM2A KDM4B KDM6B KHSRP KIAA0101 KIAA0232 KIAA0240 KIAA0485 KIAA0494 KIAA0894 KIAA1797 KIF3B KLF4 KLF5 KLF6 KLHDC10 KLHDC4 KLHL12 KLHL22 KPNA1 KRIT1 KRT10 KRT13 KRT15 KRT18 KRT19 KRT4 KRT5 KRT7 KTN1 LAGE3 LAMP1 LAPTM4B LAPTM5 LARS LCMT1 LCMT2 LCP1 LCP2 LDLRAP1 LGALS1 LILRA2 LILRA6 LILRA6///LILRB3 LILRB2 LIMK2 LIPA LITAF LMBRD1 LOC100272216 LOC100288142///NBPF1///NBPF10 LOC100506168///SFPQ LOC100506517 LOC100509558///LOC100510047 LOC100510712///MTX1 LOC728825///SUMO2 LONP1 LPL LRBA LRIG1 LRP1 LRRC47 LRRFIP1 LSM4 LUC7L3 LY75 LY96 LYN LZTFL1 M6PR MACF1 MAN1C1 MANSC1 MAP2K2 MAP2K3 MAP3K4 MAP3K7 MAP4 MAP7 MAPK14 MAPK9 MAPKAPK2 MAPRE1 MAST2 MAST4 MAT2A MATR3 MAX MAZ MBD3 MBTPS1 MCCC1 MCL1 MCM7 MDH2 ME2 MEA1 MECOM MED16 MED6 MEIS3P1 MEPCE METTL7A MFNG MGAT4B MGEA5 MIA3 MID1 MIER2 MKL2 MKNK2 MLF1 MLLT4 MLPH MLXIP MMP14 MNDA MORC2 MPDU1 MRC1///MRC1L1 MRP63 MRPL20 MRPL23 MRPL40 MRPL46 MRPL52 MRPS18A MRPS18B MRPS31 MRPS35 MS4A4A MSLN MSMB MTUS1 MUC1 MUC16 MUC4 MUC5AC MUT MXRA7 MYB MYCBP MYCBP2 MYH9 MYL6 MYL6B MYO1C MYO6 MYO9B MYOF MZF1 MZT2A MZT2B NAA40 NAB1 NACC2 NADK NAMPT NANS NAP1L1 NAV2 NBAS NBL1 NBPF1///NBPF10///NBPF11///NBPF12///NBPF14///NBPF15///NBPF16///NBPF24///NBPF8 NBR1 NCF1///NCF1B///NCF1C NCF1C NCF2 NCF4 NCK1 NCOA2 NCOR1 NDUFA1 NDUFA13 NDUFA2 NDUFA3 NDUFA8 NDUFB1 NDUFB11 NDUFB2 NDUFB7 NDUFB8 NDUFS5///RPL10 NDUFS6 NDUFS8 NDUFV1 NEDD9 NET1 NF1 NFATC2IP NFE2 NFIB NFKB1 NFYC NGFRAP1 NGRN NISCH NLRP1 NOLC1 NONO NOP56 NOSIP NOTCH2 NPEPL1 NPIPL3 NPM1 NQO1 NR2F2 NSDHL NTHL1 NUCB1 NUCB2 NUCKS1 NUDT4///NUDT4P1 NUMB NUP133 NUP43 NXN OAS1 OAZ1 OFD1 OLA1 OLR1 OR7E47P ORAI2 ORM1///ORM2 P4HA2 P4HB PABPC1 PAF1 PAIP1 PAM PANK2 PARK7 PATZ1 PBXIP1 PCBP1 PCGF2 PCM1 PCSK5 PDAP1 PDCD4 PDCD6 PDE4B PDE4C PDE6D PDE8A PDIA4 PDLIM4 PDLIM5 PDSS2 PDXK PEBP1 PECAM1 PGD PGF PGK1 PGLS PGRMC1 PHF1 PHF17 PHF20 PHF21A PHIP PHKB PI3 PICALM PIGF PIGV PIK3R3 PILRA PIN1 PIN4 PIR PKIG PKP4 PLAU PLAUR PLD3 PLEC PLEK PLEKHA5 PLLP PLS3 PLXNB2 PLXNC1 PNN POFUT2 POLDIP3 POLR1B POLR1C POLR1D POLR2E POLR2H POLR2I POLR2L POM121///POM121C POSTN POU2AF1 PPBP PPIB PPIF PPIG PPL PPP1R7 PPP3CA PQLC1 PRDM2 PRDX2 PRDX3 PRDX6 PRKAB1 PRKAR1A PRKCD PRKCI PRKCZ PRKD2 PRKDC PRNP PROM1 PRPF19 PRPF31 PRPF4 PRPF40A PRPF4B PRPF6 PRPS1 PRR11 PRR15L PRUNE PSAP PSCA PSD3 PSD4 PSEN1 PSMA1 PSMB1 PSMB7 PSMC5 PSMD13 PSMD2 PSMD4 PSME4 PTBP1 PTGER4 PTGS2 PTK2B PTMA PTP4A2 PTPLAD1 PTPN11 PTPN12 PTPRC PTPRF PTPRK PTPRO PTPRZ1 PUM1 PXN PYROXD1 QARS QPCT RAB11B RAB13 RAB14 RAB1A RAB2A RAB3GAP1 RAB4A RAB5A RAB5C RABEP2 RABGAP1 RABGAP1L RABL2A///RABL2B RAC2 RAD21 RAD23A RAE1 RALGAPB RANBP2 RANBP9 RARRES3 RASSF2 RB1CC1 RBBP6 RBM10 RBM22 RBM26 RBM5 RBM6 RBMS1 RC3H2 RCN1 RDBP RDH14 RELA RENBP RERE RFXANK RGL1 RGS2 RHEB RHOA RIN2 RIOK3 RNASE4 RNF114 RNF13 RNF146 RNF187 RPA1 RPL13 RPL14 RPL22 RPL24 RPL27 RPL3 RPL31 RPL36 RPL37A RPL38 RPL39 RPL4 RPL5 RPN2 RPS10 RPS17 RPS21 RPS28 RPS29 RPS4X RPS6KA2 RPS7 RPS9 RPSA RRAD RRAS2 RRBP1 RRM1 RUFY3 RUVBL2 S100A11 S100A13 S100A2 S100A8 S100P SAFB2 SAMSN1 SAP18 SATB1 SBNO2 SCAND1 SCARB2 SCD SCGB1A1 SCGB2A1 SCNN1A SCNN1B SCO2 SCP2 SDC1 SDC4 SDHC SDR39U1 SEC11A SEC23B SEC62 SECISBP2 SEL1L3 SELENBP1 SELL SELPLG SELT SEMA3F SEPP1 SEPT2 SERINC3 SERP1 SERPINA1 SERPINB3 SERPINB6 SERPING1 SET SETD5 SF3A2 SF3B1 SF3B5 SFN SFTPC SFXN3 SGSM3 SH3BGRL SH3BGRL3 SH3GLB1 SIPA1L3 SIRPA SKAP2 SKP1 SLC11A1 SLC11A2 SLC16A3 SLC16A5 SLC1A4 SLC24A1 SLC25A11 SLC25A38 SLC25A44 SLC2A14///SLC2A3 SLC2A3 SLC35A2 SLC35A3 SLC35D2 SLC35E1 SLC38A2 SLC39A6 SLC6A16 SLC6A6 SLC6A8 SLCO2B1 SLK SLPI SMARCA2 SMARCA4 SMARCC1 SMARCD2 SMYD2 SNN SNRNP70 SNRPA1 SNRPD2 SNRPE SNRPN///SNURF SNW1 SNX10 SNX2 SNX3 SOBP SOD2 SORBS3 SOX4 SP110 SPA17 SPAG16 SPAG6 SPARCL1 SPI1 SPINLW1 SPOP SPSB3 SPTAN1 SPTLC1 SPTLC2 SRD5A1 SRGN SRI SRPR SRRM2 SRRT SRSF1 SRSF10 SRSF2 SRSF3 SRSF7 SS18 SSRP1 ST5 ST6GALNAC4 STAG2 STAG3L1 STAT1 STAT3 STAT6 STIP1 STK24 STK38 STOM STOML1 STX11 STYK1 SUB1 SUGP1 SULT1A1 SULT1A3///SULT1A4 SUMO1 SUMO2 SURF1 SYF2 SYPL1 TAB2 TACC2 TACSTD2 TAF11 TAGLN2 TALDO1 TANK TAOK1 TARDBP TAX1BP3 TBC1D22A TBCA TBL1X TBXAS1 TCEB2 TCF12 TCF25 TCF3 TCF4 TCF7L2 TCP1 TEKT2 TEX264 TFB2M TFDP1 TFEB TFF3 TFRC TGFB1 TGM2 THADA THYN1 TIMM13 TIMM17A TIMM23///TIMM23B TKT TLN1 TLR5 TM2D1 TM9SF1 TM9SF2 TM9SF3 TMBIM6 TMC5 TMC6 TMCC1 TMCO1 TMCO3 TMCO6 TMED2 TMED3 TMED9 TMEM189-UBE2V1///UBE2V1 TMEM231 TMEM41B TMEM43 TMEM87A TMF1 TMPRSS3 TMX1 TNFAIP2 TNFAIP3 TNFAIP6 TNFSF10 TNFSF12-TNFSF13///TNFSF13 TNPO1 TNPO2 TNPO3 TNS1 TOB2 TOX3 TP53TG1 TPD52 TPM1 TPM4 TPP1 TPT1 TRA2B TRAF4 TRAK1 TRAM1 TRAPPC2L TRAPPC4 TRIM22 TRIM27 TRIM8 TROVE2 TRRAP TSC1 TSC22D1 TSC22D3 TSPAN13 TSPAN3 TSPAN31 TSPAN8 TSR1 TST TTC12 TTC15 TTC3 TTF1 TUBA1A TUBA4A TUBA4B TUBB2A TUBB2A///TUBB2B TUBBP5 TUBGCP2 TUFT1 TUG1 TWF1 TXN2 TXNIP TXNL4A TYROBP UAP1 UBAP2 UBAP2L UBE2D3 UBE2H UBE2I UBE2J1 UBE2M UBE2N UBE2Q1 UBE3A UBE4B UBQLN4 UBR5 UBXN4 UBXN7 UCP2 UGDH UPF3A UPK1B UQCR11 UQCRQ UROD USP10 USP34 USP4 USP47 USP48 USP7 UTRN VAMP1 VAMP3 VARS VAV3 VCAN VDAC1 VDAC3 VEGFA VEZF1 VGLL4 VILL VIM VPS13B VPS16 VSIG4 WAC WARS WBP11 WBSCR22 WDR1 WDR13 WDR43 WDR45 WDR48 WFS1 WIPI1 WIPI2 WLS WTAP WWOX WWTR1 XPA XPNPEP1 XPO7 XRCC6 XYLT2 YBX1 YIPF5 YIPF6 YWHAE YWHAH YWHAQ YWHAZ ZBED5 ZBTB20 ZBTB7A ZC3H14 ZC3H7B ZFAND5 ZFP36 ZFP36L2 ZMIZ2 ZMYM2 ZMYND10 ZNF148 ZNF160 ZNF207 ZNF3 ZNF329 ZNF395 ZNF432 ZNF44 ZNF451 ZNF544 ZNF573 ZNF580 ZNF593 ZNF611 ZNF688 ZNF721 ZNHIT1 ZYX | BAL_44a_A0B0 BAL_42_A0B0 BAL_37_A1B1 BAL_43_A0B0 BAL_25_A0B0 BAL_45a_A1B0 BAL_33_A0B1 BAL_12_A1B0 BAL_26_A2B0 BAL_13_A0B0 BAL_17_A0B0 BAL_1_A0B0 BAL_15b_A0B0 BAL_19_A0B0 BAL_4_A0B0 BAL_31_A0B1 BAL_3_A1B0 BAL_44b_A0B0 BAL_18_A1B0 BAL_21_A0B0 BAL_27a_A0B0 BAL_45b_A2B0 BAL_47c_A0B1 BAL_47b_A0B1 BAL_46_A2B0 BAL_40_A0B0 BAL_20b_A1B1 BAL_47a_A0B0 BAL_27b_A2B1 BAL_39_A0B1 BAL_29_A1B0 BAL_41_A1B0 BAL_15a_A0B1 BAL_20a_A1B1 NeverSmoker_3 |
| **3** | A2M AAK1 ABCC3 ABCG1 ABCG2 ABHD5 ABL1 ABLIM1 ACAA1 ACAA2 ACACB ACO1 ACOT1///ACOT2 ACOT7 ACOX1 ACP5 ACSL1 ACTN1 ACTR2 ADAM10 ADAM17 ADAM28 ADAMTSL4 ADAP2 ADCY3 ADCY7 ADD3 ADH7 ADIPOR2 ADO ADORA1 ADORA2B ADRBK1 ADSS AGBL2 AGFG1 AGPS AGR2 AIF1 AK2 AKAP13 AKR1B1 ALAS1 ALCAM ALDH1A1 ALDH1A2 ALDH1A3 ALDH2 ALDH3A1 ALG9///FDXACB1 ALOX5 ALOX5AP ALPL AMD1 AMIGO2 AMPD3 ANK3 ANPEP ANXA1 ANXA3 ANXA7 AOC3 AP1M2 AP1S2 AP2S1 APOB48R APOC1 APOC2 APOE APPL1 APRT AQP3 AQP5 AQP9 ARAP1 AREG ARHGAP25 ARHGAP32 ARHGAP5 ARHGAP6 ARHGDIA ARHGDIB ARHGEF10L ARHGEF12 ARID5B ARL3 ARL6IP4 ARMCX6 ARPC1B ARPC2 ARRB1 ARRB2 ASAP2 ASPH ASS1 ATG4B ATOX1 ATP10A ATP12A ATP1B1 ATP2A2 ATP2B1 ATP2B4 ATP2C1 ATP2C2 ATP5D ATP6AP1 ATP6V0B ATP6V0D1 ATP6V1A ATP6V1B2 ATP6V1E1 ATP6V1F ATP6V1G1 ATP7A ATP7B ATP9A ATP9B AUTS2 AVPI1 AXL AZIN1 B2M B4GALT4 B4GALT7 B9D1 BACE1 BACH1 BAG4 BASP1 BATF3 BAX BCAP31 BCL2A1 BCL6 BEX4 BHLHE41 BICD2 BID BIK BIN2 BLVRA BNIP2 BRD9 BST1 BST2 BTK C10orf18 C10orf76 C11orf75 C12orf5 C13orf15 C13orf18 C14orf109 C14orf45 C17orf101 C17orf62 C19orf10 C19orf24 C19orf56 C19orf60 C1orf38 C1QA C1QB C2 C20orf46 C21orf59 C22orf9 C3AR1 C3orf63 C4orf19 C5AR1 C6orf105 C6orf211 C6orf97 C7orf63 C8B C8orf44///SGK3 C9orf116 C9orf16 C9orf3 CA2 CALHM2 CALM1 CALML4 CAMKK2 CAMP CAMSAP1L1 CAMTA1 CAP1 CAP2 CAPG CAPN3 CARD9 CASC1 CASP1 CAV2 CCDC101 CCDC19 CCDC85B CCDC88A CCDC90B CCL18 CCL3///CCL3L1///CCL3L3 CCND3 CCNO CCR1 CCRL2 CCT2 CD101 CD14 CD163 CD164 CD24 CD302 CD33 CD36 CD37 CD44 CD46 CD52 CD53 CD58 CD68 CD69 CD81 CD83 CD86 CD97 CDA CDC14B CDC42EP3 CDH1 CDH3 CDK18 CDK7 CEACAM21 CEACAM5 CEACAM6 CEACAM8 CEBPA CEBPB CELF2 CELSR1 CEP170 CETN2 CFD CFH///CFHR1 CHI3L1 CHIT1 CHMP2B CITED2 CKLF CLCN3 CLDN10 CLDN3 CLDN4 CLDN7 CLEC4A CLEC5A CLEC7A CLIC2 CLIC4 CLINT1 CLMN CLU CMAH CNN3 CNPY3 COL8A2 COLEC12 COMMD9 COPA COPB1 COQ2 CORO1C COTL1 COX6B1 CP CPE CPM CPVL CREB1 CREG1 CRIP1 CRTAM CRTAP CRTC3 CSDA CSF1R CSF2RA CSF2RB CSGALNACT2 CSNK1A1 CTBP2 CTGF CTNNA1 CTSA CTSD CTSH CTSL1 CTSZ CXADR CXCL1 CXCL2 CXCL3 CXCL5 CXCR4 CXCR7 CYB561 CYBB CYP27A1 CYP2B6///CYP2B7P1 CYP2B7P1 CYP2J2 CYP4B1 CYTH1 CYTIP DAB2 DAPK1 DAZAP2 DCN DCTN2 DDB2 DDR1 DDX58 DECR1 DEGS1 DENND2D DENND4B DENND4C DENND5A DERL1 DGAT1 DHRS9 DIAPH1 DLC1 DLEC1 DLG3 DMXL2 DNAH3 DNAH7 DNAI1 DNAJC10 DNAJC8 DNALI1 DNASE2 DNASE2B DNM2 DOCK10 DOCK2 DOCK9 DOK2 DPEP2 DPYSL2 DR1 DRAM1 DRAP1 DSC2 DSE DSG2 DSP DST DSTN DTX4 DUOX1 DUSP14 DUSP3 DZIP3 ECE1 ECH1 EDEM2 EDF1 EEF1D EFCAB1 EFEMP1 EFHC1 EFHC2 EGFR EGR2 EHBP1L1 EHF EID1 EIF1AX EIF3K ELF3 ELF4 ELMO1 ELMO2 EML4 EMP2 EMP3 EMR1 ENC1 ENG ENSA ENTPD3 EPB41L3 EPB41L4B EPCAM EPHA2 EPS8 ERAP1 ERBB2 ERBB3 EREG ESRP1 ESYT1 EVI2A EVI2B EVL EXOC5 EXOC7 EXPH5 EYA2 EZR F3 FABP3 FABP4 FABP5 FADS1 FAH FAM102A FAM105A FAM114A1 FAM120A FAM134A FAM134C FAM171A1 FAM178A FAM179B FAM38A FAM49A FAM49B FAM59A FAM60A FAM82A2 FAR2 FAT1 FBP1 FBXO11 FCER1G FCGR1A///FCGR1C FCGR1B FCGR2A FCGR2C FCGR3A///FCGR3B FCGR3B FCGRT FEZ2 FGD2 FGFR2 FGFR3 FGR FHL1 FIG4 FILIP1L FKBP15 FLI1 FLNA FLOT2 FLVCR2 FMNL1 FMO3 FN1 FOLR1 FOXA1 FOXJ1 FPR1 FPR2 FPR3 FRMD4A FUT3 FUT6 FXYD3 FXYD5 FZD6 G0S2 GAA GABARAPL1 GABARAPL1///GABARAPL3 GABRP GALNT3 GALNT7 GALT GAPDH GAS2L1 GBA///GBAP1 GBAP1 GCA GCC2 GCDH GCHFR GCLM GGA2 GK3P GLA GLB1 GLIPR1 GLRX GLRX2 GLS GLT25D1 GLTSCR2 GLYR1///SEPT6 GM2A GMFB GMFG GMIP GNA14 GNAI2 GNAS GNPDA1 GNS GOLGA8B GOLM1 GOLT1B GPA33 GPD1 GPNMB GPR109B GPR137B GPR56 GPR65 GPRC5A GPSM3 GPX1 GPX3 GRAMD3 GRB2 GRHL2 GRINA GRN GSTA1 GSTA3 GSTA4 GSTO1 GTF2I///GTF2IP1///LOC100093631 GTPBP2 GULP1 GUSB GYPC H2AFY HBEGF HCFC1R1 HCK HCLS1 HDDC2 HEBP1 HECTD3 HERC5 HEXA HEXB HHEX HIST2H2AA3///HIST2H2AA4 HIST2H2BE HK2 HK3 HLA-DMB HLA-DPA1 HLA-DQA1 HLA-DQB1 HLA-DQB1///LOC100133583 HLA-DQB2 HLA-DRB1///HLA-DRB3///HLA-DRB4///HLA-DRB5///LOC100133661///LOC100294036///LOC100509582///LOC100510495///LOC100510519 HMOX1 HMOX2 HN1L HNMT HNRNPD HOXB6 HP HP///HPR HPCAL1 HPGD HPS5 HRASLS2 HS3ST1 HS3ST2 HSD17B10 HSD17B14 HSP90AA1 HSP90AB1 HSPA13 HSPA4 HSPA6 HSPA8 HSPD1 HSPH1 ICAM2 ICOS ID1 ID2 ID3 IER5 IFFO1 IFI30 IFIH1 IFRD1 IFT52 IFT57 IFT88 IGF2BP2 IGF2R IGFBP2 IGFBP3 IGFBP5 IGFBP7 IGSF6 IL10RA IL13RA1 IL17RB IL1B IL27RA IL2RG IL7R INHBA INPP4A IPO7 IQCG IQCK IQGAP2 IQSEC1 IRAK1 IRF6 IRF7 IRF8 IRX5 ISG15 ITGA2 ITGA5 ITGAL ITGAM ITGB2 ITGB4 ITIH5 ITPK1 ITPR2 ITPR3 JAKMIP2 JMJD6 JUP KCNA3 KCNAB1 KCNAB2 KCNK1 KCTD5 KHSRP KIAA0226 KIAA0922 KIAA1033 KIAA1324 KIAA1598 KIF16B KIF2A KLF10 KLF11 KLF5 KLF6 KMO KRT15 KRT17 KRT18 KRT19 KRT5 KRT7 KRT8 KYNU LAIR1 LAMB1 LAMP1 LAMP2 LAMP3 LAPTM4B LAPTM5 LAT2 LCN2 LCP1 LCP2 LDLR LEPROTL1 LGALS1 LGALS3BP LGALS9 LGMN LILRA2 LILRA6 LILRB1 LIMCH1 LMNA LOC100510712///MTX1 LOC220594 LONP1 LPL LPXN LRIG1 LRP1 LRPAP1 LRRC23 LRRC48 LRRC50 LRRC6 LSP1 LSR LST1 LTA4H LTB4R LXN LY86 LY96 LZTFL1 M6PR MAD1L1 MAF MAFB MAK MAN1A1 MAN2B1 MANBA MAOA MAP1B MAP2K2 MAP3K11 MAP3K8 MAP7D1 MAPK10 MAPKAPK2 MAPKAPK3 MARCKS MARCKSL1 MARCO MAST3 MBD3 MBNL1 MBOAT2 MCL1 MCM5 MCOLN1 MDFIC MDK MEAF6 MECOM MED6 MED8 MEF2A MEGF9 MEIS1 MEIS2 MET MFNG MGAT1 MICAL1 MICB MIIP MITF MLF1 MLX MMD MME MMP19 MNDA MNS1 MOBKL1B MPP1 MR1 MRC1///MRC1L1 MRPL18 MS4A4A MS4A6A MSLN MSMB MSN MSR1 MSRB2 MTR MTSS1 MTX2 MUC1 MUC16 MUC4 MUC5AC MXRA7 MYD88 MYH10 MYO1B MYO1F MYO5A MYO5C MYO9B MYST1 N4BP2L1 NAGA NAGK NAGLU NAGPA NAMPT NAT1 NBPF1///NBPF10///NBPF11///NBPF12///NBPF14///NBPF15///NBPF16///NBPF24///NBPF8 NCALD NCF2 NCF4 NCK2 NCKAP1 NCKAP1L NDRG2 NDUFB2 NDUFB7 NDUFS8 NEBL NECAP2 NEK1 NEK11 NENF NET1 NFE2L2 NFIB NFIL3 NLRP1 NME5 NME7 NNMT NOP10 NOP2 NPC1 NPC2 NPL NQO1 NQO2 NR1H3 NR2F2 NR3C1 NRBF2 NRIP1 NRP1 NTAN1 NUCB1 NUCB2 NUDT1 NUP210 NUS1///NUS1P3 OAS2 OAS3 OASL OAT OLR1 OPN3 ORAI3 OSBPL11 OSBPL1A OSTM1 OVGP1 P2RX4 P2RY13 PACRG PAK2 PALLD PAPOLA PARD3 PARP12 PARVB PAWR PBX3 PCDHGA1///PCDHGA10///PCDHGA11///PCDHGA12///PCDHGA2///PCDHGA3///PCDHGA4///PCDHGA5///PCDHGA6///PCDHGA7///PCDHGA8///PCDHGA9///PCDHGB1///PCDHGB2///PCDHGB3///PCDHGB4///PCDHGB5///PCDHGB6///PCDHGB7///PCDHGC3///PCDHGC4///PCDHGC5 PCK2 PCNX PCOLCE2 PDE4A PDE4B PDE4DIP PDGFC PDGFD PDLIM2 PDXK PEA15 PECAM1 PEPD PERP PFDN5 PFN1 PFN2 PGLS PHACTR1 PHKA2 PHTF1 PI4KA///PI4KAP1///PI4KAP2 PIBF1 PICALM PIK3CA PIK3CB PIK3R3 PILRA PION PITX1 PKP4 PLA2G15 PLA2G16 PLA2G7 PLAU PLAUR PLBD1 PLD3 PLEK PLEKHA5 PLEKHB2 PLEKHM2 PLIN2 PLOD1 PLS1 PLS3 PLXNB1 PLXND1 PMP22 PMS2P4 PNP PNPLA6 PODXL POGK POLD4 POLG POMZP3///ZP3 PON2 PPAP2C PPARD PPARG PPBP PPIF PPL PPP1R12A PPP2CB PPP2R5B PPP3CB PPT1 PQLC1 PRKACB PRKAR1A PRKAR2B PRKCI PRNP PROCR PROM1 PRSS21 PRSS22 PRSS23 PSAP PSENEN PSMA7 PSMB1 PSMD2 PTGER4 PTK2B PTPN12 PTPN22 PTPN3 PTPN6 PTPRC PTPRF PTPRK PTPRN2 PTPRO PTPRZ1 PURA PYCARD PYGL QDPR QKI QPCT RAB20 RAB25 RAB31 RAB36 RAB7L1 RAB8A RAB8B RAC2 RAI14 RALGAPA1 RANBP9 RANGRF RAP2B RAP2C RARA RASA1 RASSF1 RBP4 RBPJ RBPMS RCN2 RECQL RENBP RETN RFXANK RGS1 RGS12 RGS19 RGS2 RHOG RHOQ RHOT1 RIN3 RIPK2 RNASE4 RNASE6 RNASET2 RNF111 RNF13 RNFT1 RNH1 RNPEPL1 ROBLD3 RP2 RPL14 RPS11 RPS15 RPS4Y1 RPS6KA1 RRAD RRAGC RRAGD RRAS2 RRP7A RSU1 RTF1 RTN1 RUFY2 RXRA S100A14 S100A2 S100A4 S100P SAC3D1 SAMSN1 SART1 SASH3 SCARB2 SCD SCD5 SCGB2A1 SCNN1A SCO2 SDC1 SDC2 SEC23A SEH1L SEL1L SELENBP1 SELPLG SEPT11 SERPINA1 SERPINB13 SERPINB3 SERPINB3///SERPINB4 SERPINB5 SERPINB6 SERPINF1 SERPING1 SFXN3 SGPP1 SH2B3 SH3BGRL3 SH3BP5 SH3GL3 SH3TC1 SIAH2 SIDT2 SIGLEC1 SIGLEC7 SIRPA SIRPB1 SIX2 SKAP2 SLA SLAMF8 SLC11A1 SLC15A2 SLC15A3 SLC16A6 SLC19A2 SLC19A3 SLC22A4 SLC25A4 SLC27A2 SLC27A3 SLC29A1 SLC2A10 SLC2A14///SLC2A3 SLC2A3 SLC31A2 SLC34A2 SLC38A1 SLC3A2 SLC43A3 SLC44A4 SLC47A1 SLC4A4 SLC6A14 SLC7A7 SLC7A8 SLCO2B1 SLPI SMAD3 SMAD7 SMAGP SMARCA2 SMCR7L SNTB1 SNX10 SNX2 SNX7 SORT1 SOX2 SOX4 SP110 SPA17 SPAG6 SPARC SPATS2L SPI1 SPP1 SPTBN1 SQLE SRGN SRP9 SSBP2 SSR3 ST3GAL6 ST6GAL1 STAC STEAP1 STK10 STMN1 STON1 STX11 STX12 STX6 STX7 STXBP2 SUPT6H SURF1 SVIL SYBU SYK SYNCRIP SYNE1 SYNE2 TACC2 TACSTD2 TAGLN TAOK3 TARP TARP///TRGC2 TBC1D2 TBC1D4 TBC1D8 TBXAS1 TCEB2 TCF25 TCF4 TCF7L2 TCIRG1 TEX14 TEX264 TFDP1 TFEC TFRC TGFB1 TGFBI TH1L THBD THBS1 TIAM1 TIMM13 TIMP2 TJP1 TJP3 TLN1 TLR1 TLR4 TLR7 TLR8 TM6SF1 TM7SF2 TM7SF4 TM9SF2 TMC5 TMC6 TMCO1 TMED5 TMEFF1 TMEM127 TMEM14A TMEM14B TMEM160 TMEM176B TMEM231 TMEM30B TMEM38B TMEM50B TMPRSS4 TMSB10 TMX4 TNFRSF1B TNFRSF21 TNFSF10 TNFSF12 TNFSF12-TNFSF13///TNFSF13 TNFSF13 TNIK TNNI2 TNS3 TOB1 TOM1L1 TPBG TPK1 TPP1 TPPP3 TPT1 TRAF4 TRAK1 TREM1 TREM2 TRHDE TRIM14 TRIM2 TRIM29 TRIP13 TRPV2 TSC22D2 TSGA10 TSPAN1 TSPAN13 TSPAN3 TSPAN4 TSPAN6 TSPAN8 TSPO TTC9 TUBB TUBB2C TUBB6 TUG1 TUSC3 TXNRD2 TYROBP UAP1L1 UBE2K UBXN2B UBXN4 UGT1A1///UGT1A10///UGT1A3///UGT1A4///UGT1A5///UGT1A6///UGT1A7///UGT1A8///UGT1A9 UPK1B UPP1 UROD USP10 USP15 USP25 VAC14 VAMP3 VAMP4 VASH1 VASP VAT1 VAV1 VCAN VDAC3 VDR VIM VKORC1 VPS16 VPS4A VSIG4 VTI1B WAPAL WAS WDR19 WDR78 WFDC2 WIPF1 WWTR1 XIST XRCC5 YWHAE ZBBX ZCCHC2 ZDHHC13 ZEB2 ZFP36L2 ZFYVE16 ZFYVE26 ZMIZ1 ZMYND10 ZMYND8 ZNF238 ZNF331 ZNF589 ZYX | BAL_17_A0B0 BAL_15a_A0B1 BAL_1_A0B0 BAL_26_A2B0 BAL_3_A1B0 BAL_45a_A1B0 BAL_44b_A0B0 BAL_33_A0B1 BAL_43_A0B0 BAL_4_A0B0 BAL_40_A0B0 BAL_18_A1B0 BAL_39_A0B1 BAL_25_A0B0 BAL_13_A0B0 BAL_27a_A0B0 BAL_37_A1B1 BAL_12_A1B0 BAL_47c_A0B1 BAL_31_A0B1 BAL_47a_A0B0 BAL_47b_A0B1 BAL_15b_A0B0 BAL_41_A1B0 BAL_44a_A0B0 BAL_20b_A1B1 BAL_42_A0B0 BAL_29_A1B0 BAL_45b_A2B0 BAL_27b_A2B1 BAL_19_A0B0 BAL_21_A0B0 BAL_46_A2B0 BAL_20a_A1B1 |
| **4** | AAK1 AAMP AARS ABCA1 ABCA2 ABCA7 ABCB8 ABCF3 ABHD8 ABO ABT1 ACO2 ACOX1 ACSBG2 ACSL3 ACTL7A ACTR3 ADAM21///ADAM21P1 ADCK2 ADD1 ADD2 ADM ADRA1B ADRM1 AES AGPAT2 AHSA1 AK1 AKAP1 AKAP8L AKAP9 AKR7A2 AKT3 ALDH1A1 ALDH3A2 ALDH3B1 ALDOA ALG6 ALK ANAPC2 ANKLE2 ANKRD12 ANKRD5 ANP32E ANXA11 AOX1 AP1S1 AP2A2 AP3D1 AP4M1 APBA3 APC2 API5 APLP2 APRT AQP3 AQP9 ARAP2 ARF5 ARL2 ARL5A ARL6IP4 ARPC2 ASAH1 ASB8 ASCC2 ATF5 ATP13A1 ATP13A2 ATP2A2 ATP5G2 ATPIF1 ATXN2 AURKAIP1 AZI1 B2M BAD BAG1 BAIAP2 BANF1 BAT2L2 BAT3 BCL10 BGLAP///PMF1 BIRC2 BLM BLVRB BSCL2 BTBD2 BTN3A2///BTN3A3 BUD31 C11orf2 C14orf1 C15orf34 C15orf39 C16orf53 C18orf25 C19orf28 C1orf105 C1orf135 C1orf77 C21orf33 C21orf59 C21orf91 C4orf29 C6orf108 C6orf54 C7orf26 C8orf33 C9orf16 CACNA1G CALM3 CAPN1 CAPN3 CASP2 CBFA2T2 CBL CC2D1A CCDC101 CCDC56 CCL3///CCL3L1///CCL3L3 CCL4 CCND2 CCNL2 CCR3 CCR5 CCS CCT2 CCT3 CCT7 CD151 CD177 CD1A CD2BP2 CD59 CD93 CD99 CDC42BPA CDC42SE1 CDK10 CDK11A///CDK11B CDK16 CDK19 CDK2AP2 CDK3 CDK5 CDYL CENPE CENPT CEP164 CES1 CHCHD2 CHMP6 CHPF2 CHRM2 CHST15 CHSY1 CLC CLDN3 CLEC1A CLN6 CLPP CLTB CLU CMTM6 CNOT3 CNPY2 COL6A1 COMMD4 COPE COQ4 COQ7 COQ9 CORO1B COX16 COX5B COX6B1 COX8A CPNE1 CPSF1 CPSF3L CREB3 CRIP1 CRIP2 CROCCP3 CSDA CSF2RB CSNK1D CTNNA1 CTSH CTSS CUL7 CXCL9 CXCR4 CXXC1 CYC1 CYHR1 CYP1A2 CYP2C9 DAK DAPK3 DAXX DAZ1///DAZ2///DAZ3///DAZ4 DCAF11 DCPS DCTN1 DDIT3 DDRGK1 DDT DDX1 DDX17 DDX27 DDX41 DDX49 DEF6 DGCR6///DGCR6L DGKQ DHPS DHRS7B DHX16 DHX30 DHX38 DICER1 DKFZp686O1327 DLEC1 DMD DNAJC10 DNAJC4 DND1 DNPEP DOK3 DOK5 DOT1L DTX2 DTX3 DYNC1H1 DYNLRB1 E4F1 ECHDC2 ECSIT EDC3 EDDM3A EDF1 EDNRB EEF1D EEF1G EFNA4 EID1 EIF3G EIF4B EIF4G1 EIF6 ELAC2 ELAVL2 ELN ELOVL1 EMR2 ENSA EPAG EPHX1 ERGIC3 ETFB EWSR1 EXOC7 EXOSC10 EXOSC4 EZR FABP6 FAHD2A FAM108A1 FAM125B FAM160B2 FAM176B FAM190B FAM96B FARP1 FARP2 FAS FAU FBXL15 FCGR3B FDPS FER1L4 FFAR2 FGFR3 FH FIP1L1 FIS1 FKBP1B///MFSD2B FKBP4 FKBP8 FKTN FLT3LG FOLR1 FOXO3///FOXO3B FUS FZD8 GADD45GIP1 GALK2 GAMT GAS1 GAS6 GBP1 GBP2 GCGR GCH1 GDAP2 GFI1B GJA3 GML GMPPB GOLGA2 GPAA1 GPKOW GPR44 GPS1 GPT GRHPR GRIK5 GRIN2C GRLF1 GRSF1 GSTA1 GSTK1 GSTP1 GTF2F1 GTF2H1 GUK1 H1FX HAGH HARS HAUS5 HDAC5 HES2 HFE HGS HIF1A HIF3A HIST1H2AJ HIST1H3G HLA-A///HLA-F///HLA-J HMG20B HNF4A HNRNPH1 HNRNPM HNRNPR HOMER3 HSBP1 HSF1 HSP90AB1 HSPA1A///HSPA1B HSPA8 HSPB1 HYAL2 HYMAI ICT1 IDH2 IDH3G IDS IDUA IFT122 IFT27 IGFBP2 IGFBP5 IGHG1 IGHM///LOC100133862 IK IL12RB1 IL1B IL1RL1 IL1RN ILF3 ILVBL ING2 INHBC INO80B INTS1 INTS6 IPW IRF3 ITM2B IVNS1ABP JUND KANK2 KAT5 KATNB1 KCNA5 KCNJ15 KCNK13 KCNMB1 KDM6B KIAA0101 KIAA0125 KIF5A KIR3DL1///KIR3DL2///LOC727787 KLF13 KLHL1 KRT19 KYNU LAIR2 LARP1 LASS4 LIN7B LMAN2 LMNA LMNB1 LOC100132247///LOC348162///LOC613037///LOC728888///NPIPL3 LOC100507328 LOH3CR2A LPCAT1 LPCAT4 LPPR3 LRDD LRRC14 LRRC41 LRRC42 LSM4 LSM7 LTBP3 LTBP4 LTC4S LYN LYZ MACROD1 MAFF MAGEA9///MAGEA9B MAGED2 MAN1B1 MAP2K2 MAST2 MBD5 MBTPS1 MCAT MCRS1 MED16 MED25 METTL1 MFSD10 MFSD9 MIF MKI67 MLST8 MMP12 MMP17 MPST MRPL12 MRPL17 MRPL18 MRPL23 MRPL4 MRPL40 MRPS12 MRPS2 MRPS7 MS4A1 MSMB MUC5B MUC6 MXD4 MYH10 MYH14 MYL9 MYO1C MYO7A MYST2 MZF1 MZT2A N4BP2L2 NAA10 NADSYN1 NAMPT NANS NASP NBEAL2 NCOR2 NDUFA13 NDUFAB1 NDUFS8 NDUFV1 NFYC NGRN NHEJ1 NIPSNAP3B NLRP3 NMBR NME3 NME4 NOL12 NOL12///TRIOBP NOL3 NOMO1///NOMO2///NOMO3 NOP56 NPEPL1 NPIPL2///NPIPL3///PDXDC2P NPM1 NPPC NR1H2 NSDHL NSUN5 NT5C NTRK3 NUDC NUMA1 NUP62 NUPR1 OAZ2 ODF2 OGDH OGFOD1 OPA1 OR12D3///OR5V1 OR1A1 OR7A5 OSGEP OTUB1 P4HB PAFAH1B3 PALLD PAN2 PCBD1 PCDH7 PCNXL2 PCSK1N PCYT2 PDAP1 PDE4B PDHB PDLIM4 PEBP1 PEX16 PEX6 PFDN5 PFKFB3 PGLS PGLYRP1 PHF8 PHLDA1 PIAS4 PIGO PILRB PITPNM3 PKD1 PKN1 PLA2G6 PLEK PLEKHB2 PLXNA2 PLXNB1 PLXNC1 PMM1 PMPCA PMS2L2 PMS2P3 PMVK PNKP POFUT1 POLQ POLR2E POLR2F POLR2L POLRMT POR PPDPF PPIB PPIL2 PPM1F PPP1R7 PPP2R2D PPP3R1 PPP5C PPY PQBP1 PRCC PRDM12 PRKAR1A PRKCSH PRKD2 PRMT1 PRMT2 PRMT7 PRPF31 PRPF6 PRPF8 PSMD4 PTGS1 PTGS2 PTH2R PTMS PTOV1 PTPRF PUF60 PUM1 PURA PYROXD1 QTRT1 RABAC1 RABL2A///RABL2B RABL3 RAD51L3 RALY RANBP1 RARA RASSF2 RBM42 RBM4B RBM5 RCN3 REL REST RFX1 RGS12 RGS14 RGS2 RHBDD3 RHOC RHOH RNASEH2B RNF126 RNF40 RNF5 RNF8 ROGDI RPL13 RPL13A///RPL13AP5///RPL13AP6 RPL18 RPL18A///RPL18AP3 RPL22 RPL29 RPL3 RPL4 RPL8 RPN1 RPS3 RPS6 RPS6KA6 RPS9 RRAD RRBP1 RRH RRP7A RUNX1T1 S100A13 S100A6 S100PBP SAFB2 SAMSN1 SBF1 SCAMP4 SCD5 SCGB1A1 SDCBP SDF2L1 SEC14L3 SEC23B SEC23IP SEC24C SEC61A1 SEL1L SELL SEPW1 SERPINB1 SERPINB6 SERPINB9 SF3B2 SFI1 SGSM3 SH3GLB2 SIGIRR SIGLEC6 SIL1 SIRPB1 SIVA1 SLC10A2 SLC16A1 SLC17A1 SLC22A7 SLC25A14 SLC2A3 SLC30A10 SLC35A2 SLC7A5 SLCO1A2 SLPI SMARCA2 SMARCA4 SMARCB1 SNCA SNCG SNRPA1 SNRPD3 SNRPN///SNURF SNX1 SNX27 SOD2 SPAG8 SPHK2 SPINT1 SPSB3 SPTAN1 SRC SRF SRGAP2 SRGN SSRP1 SSSCA1 SSX1 SSX3 SSX4///SSX4B ST7 STAG2 STC1 STOML2 STRN4 SUB1 SUGP1 SULT1A1 SULT1A3///SULT1A4 SUPT5H SUPV3L1 SURF2 SUZ12 SYMPK TAAR2 TAF6L TAS2R4 TAZ TBCD TBL3 TBX2 TBXA2R TCEB2 TCF20 TCF3 TCF7L2 TEKT2 TELO2 TEX264 TFAM TFDP1 TFIP11 TFPT TH1L THAP4 THAP7 THOC6 THOP1 TIMM44 TLR2 TM9SF4 TMED1 TMEM115 TMEM160 TMEM222 TMEM57 TNFAIP6 TNFRSF25 TOP3B TPD52L1 TPM1 TPM2 TPRA1 TRAF3IP1 TRAPPC6A TRIM28 TRNAU1AP TRO TSC2 TSPAN3 TSSK2 TSTA3 TTC17 TUBA1B TUBA3D TUBB2A///TUBB2B TUBB2C TUBB3 TUBGCP2 TUSC2 TUT1 TXLNA U2AF1 U2AF2 UBAC1 UBC UBE2NL UBTF UBXN6 UCKL1 UPF1 UPF3A USE1 USP11 USP34 UTP18 VAMP1 VCP VCX2 VDAC3 VNN2 VPS28 VPS39 VPS4B WAS WDR18 WDR46 WDR59 WDTC1 WNT6 WSCD1 WWC1 XAB2 XPO6 YIPF2 YIPF3 YY1 ZBTB17 ZBTB44 ZBTB48 ZC3H13 ZCCHC8 ZDHHC11 ZFP64 ZGPAT ZIC3 ZMAT5 ZNF167 ZNF267 ZNF282 ZNF32 ZNF528 ZNF768 ZNF839 ZNRF4 ZWINT | BAL_46_A2B0 BAL_39_A0B1 BAL_20a_A1B1 BAL_27b_A2B1 BAL_18_A1B0 BAL_42_A0B0 BAL_45a_A1B0 BAL_26_A2B0 BAL_47b_A0B1 BAL_21_A0B0 BAL_40_A0B0 BAL_43_A0B0 BAL_4_A0B0 BAL_20b_A1B1 BAL_33_A0B1 BAL_37_A1B1 BAL_13_A0B0 BAL_47a_A0B0 BAL_47c_A0B1 BAL_17_A0B0 BAL_19_A0B0 BAL_25_A0B0 BAL_45b_A2B0 BAL_12_A1B0 BAL_3_A1B0 BAL_29_A1B0 BAL_44b_A0B0 BAL_15a_A0B1 BAL_27a_A0B0 BAL_1_A0B0 BAL_15b_A0B0 BAL_44a_A0B0 BAL_41_A1B0 BAL_31_A0B1 FormerSmoker_69 |
| **5** | ALDH1A1 ANKRD12 ANXA1 ANXA2 ATP2A2 ATP5A1 ATXN3 AZIN1 BANF1 BCLAF1 BTF3 CALM1 CAPNS1 CD164 CD59 CES1 COX6A1 CTNNA1 DAD1 DDX1 DSTN DYNLRB1 EEF2 EIF4A2 ELAVL2 EPHA3 ERP29 FAM120A GAPDH GPX4 GTF2I///GTF2IP1///LOC100093631 HAX1 HIF3A HNRNPA2B1 HSP90AA1 HSP90AB1 HSPA8 KRT6A LATS1 MAP2K5 MBD5 MBTPS1 MMADHC MS4A1 NRG2 P4HB PCBP2 PDIA3 PDLIM1 PHLDA1 PPP1R7 PPP2CB PRKAR1A PRPF8 PTGES3 RFPL3S RPL15 RPL8 RPS15 RPS9 SCN3A SET SKP1 SLC30A10 SNRNP200 SRP14 SRSF9 TKTL1 TM9SF1 TNFAIP6 TSPAN3 UTP18 VDAC3 ZNF157 | BAL_20a_A1B1 CurrentSmoker_54 BAL_46_A2B0 CurrentSmoker_37 NeverSmoker_52 CurrentSmoker_29 Sample_43_never_smoker CurrentSmoker_1 FormerSmoker_69 BAL_19_A0B0 BAL_21_A0B0 FormerSmoker_27 FormerSmoker_18 NeverSmoker_93 NeverSmoker_3 NeverSmoker_103 Sample_44_never_smoker NeverSmoker_38 CurrentSmoker_39 Sample_48_never_smoker Sample_40_former_smoker_without_cancer CurrentSmoker_2 CurrentSmoker_12 FormerSmoker_76 NeverSmoker_45 FormerSmoker_28 BAL_27b_A2B1 CurrentSmoker_115 CurrentSmoker_36 FormerSmoker_41 NeverSmoker_42 Sample_46_never_smoker FormerSmoker_84 BAL_44a_A0B0 FormerSmoker_87 CurrentSmoker_10 NeverSmoker_105 Sample_47_never_smoker FormerSmoker_104 CurrentSmoker_98 NeverSmoker_78 FormerSmoker_34 NeverSmoker_100 BAL_29_A1B0 |

*BAL- Human Lung transplant. The numeric part of the sample name is an arbitrary identifier for individual patients. Bronchoalveolar lavage samples obtained from lung transplant recipients whose biopsies had a perivascular score (A) of between 0 and 2, and a bronchiolar score (B) of between 0 and 1. A combined A and B score of 2 or more represents an acute rejection [46].

**Lung epithelial cell transcriptome study of 34 current smokers, 18 former smokers, and 23 subjects who had never smoked [47].

**TableS4B: Membership of *Ten* Biclusters Learned Using Factor Analysis for Bicluster Acquisition (FABIA)**

| **BICLUSTER NUMBER** | **BICLUSTER GENES** | **BICLUSTER PHENOTYPES**  *BAL or **Smoking Status |
| --- | --- | --- |
| **1** | A2M AAMP ABCA1 ABCA6 ABCB9 ABCG1 ABCG2 ABHD11 ABHD5 ABHD8 ABL1 ABLIM1 ABT1 ACADVL ACSL1 ACSL5 ACSL6 ACSM3 ACTN1 ACTN4 ACTR2 ADAM20 ADAM21///ADAM21P1 ADAM28 ADAM8 ADAMDEC1 ADAMTS1 ADAMTS6 ADAMTSL4 ADCY9 ADD2 ADM AGBL2 AGR2 AGRN AHDC1 AIF1 AIFM1 AK1 AK4 AKAP1 AKAP13 AKAP8L AKR1C1 AKR1C3 AKR7A2 AKR7A3 ALDH1A2 ALDH1A3 ALDH3A1 ALDOA ALG12 ALG5 ALK ALOX5 ALOX5AP ALS2CL AMD1 AMZ2 ANAPC2 ANKHD1-EIF4EBP3///EIF4EBP3 ANKMY1 ANKMY2 ANKRD12 ANKRD36BP2 ANP32A///ANP32D ANXA10 ANXA11 ANXA7 ANXA9 AOC2 AOX1 AP1G2 AP1S1 AP1S2 APC APEH API5 APOBEC3A APOC2 APOL2 APP APRT AQP9 ARAP1 ARFIP2 ARHGAP15 ARHGAP25 ARHGAP33 ARHGAP5 ARHGAP8///PRR5-ARHGAP8 ARHGDIB ARL4C ARMC9 ARPC4 ARPC5 ART4 ARTN ARVCF ASL ASS1 ATF6 ATP13A1 ATP13A2 ATP1B1 ATP2A2 ATP2B1 ATP2C2 ATP6V0A2 ATP6V1A ATP6V1B2 ATP6V1C1 ATP6V1D ATP6V1G2///BAT1 ATR ATXN1 ATXN2 ATXN7 B9D1 BACE2 BACH1 BAIAP2 BAIAP2L2 BAIAP3 BAK1 BAT2L1 BAT2L2 BAT3 BAX BAZ2A BBS1 BBX BCAT1 BCL2 BCL2A1 BCL3 BCLAF1 BDH1 BEST1 BID BIK BIRC3 BIRC5 BLVRA BLVRB BMP6 BMS1 BNIP1 BPHL BPY2 BRD4 BTBD1 BTBD2 BTF3 BTG1 BTG2 BTK BTN3A1 BUB1 BUD31 C10orf118 C10orf18 C10orf92 C11orf24 C12orf35 C12orf5 C13orf18 C15orf29 C15orf39 C16orf3 C16orf58 C17orf91 C19orf42 C1orf38 C1orf54 C1QB C1QBP C22orf29 C2CD2 C3AR1 C6orf15 C6orf211 C6orf62 C7orf58 C9orf116 C9orf95 CA5BP CABIN1 CACNB3 CALM1 CANT1 CAPNS1 CARKD CASP1 CAV2 CBL CCDC130 CCDC19 CCDC41 CCDC48 CCDC56 CCDC88A CCDC90B CCL18 CCL2 CCL3///CCL3L1///CCL3L3 CCL4 CCL8 CCNA2 CCND2 CCNE2 CCNF CCNG2 CCNL2 CCNO CCR1 CCR2 CCR3 CCR5 CCR7 CCRL2 CCT7 CD101 CD14 CD151 CD163 CD164 CD177 CD2 CD24 CD28 CD300A CD36 CD37 CD3D CD40 CD44 CD46 CD47 CD48 CD52 CD53 CD6 CD69 CD83 CD84 CD86 CD8A CD8B CD9 CDC25A CDC34 CDC42BPB CDC42EP3 CDC42SE1 CDC5L CDC6 CDCP1 CDH1 CDK1 CDK11A///CDK11B CDK19 CDK2 CDK2AP2 CDK9 CEACAM3 CEACAM6 CEACAM8 CELF2 CENPE CENPT CEP63 CEP68 CEPT1 CETN2 CFB CFLAR CHD1 CHI3L1 CHIT1 CHKB-CPT1B///CPT1B CHPF CHRD CHRM2 CHRNE CHST11 CHST15 CHST3 CHST7 CHSY1 CIDEC CIZ1 CKB CKLF CLC CLCN3 CLDN3 CLDN4 CLDN7 CLEC16A CLEC2B CLEC4A CLEC4M CLEC5A CLEC7A CLIC2 CLIC4 CLMN CLN3 CLPTM1 CLTB CLU CNPY4 COBL COG7 COL1A2 COL4A6 COL6A1 COLEC12 COMMD4 COMT COPE COPG COQ4 COQ9 CORO1A CORO1B COX4I1 COX5B CPB1 CPM CPNE1 CPSF1 CPVL CRAT CREB1 CREB3L1 CREB5 CREBL2 CRELD2 CREM CRH CRIP1 CRIP2 CRKL CROCC CRTAP CSDA CSF2RB CSF3R CSGALNACT1 CSGALNACT2 CSHL1 CSNK1D CST3 CST6 CTBP1 CTBP2 CTBS CTNNA1 CTPS2 CTSK CUZD1 CWC25 CWH43 CXCL10 CXCL11 CXCL13 CXCL5 CXCL9 CXCR2 CXCR4 CXCR6 CYBB CYC1 CYFIP2 CYLD CYP19A1 CYP1A2 CYP27A1 CYP2A7 CYP2B6///CYP2B7P1 CYP2C9 CYTH4 CYTIP DAB2 DACH1 DAPK1 DAZ1///DAZ2///DAZ3///DAZ4 DCAF15 DCAF16 DCPS DCTN1 DDIT3 DDR1 DDT DDX17 DDX21 DDX41 DDX58 DDX60 DEFA1///DEFA1B///DEFA3 DEFB1 DENND3 DEPDC5 DES DFNB31 DGCR11 DGKQ DGKZ DHPS DHRS1 DHRS4///DHRS4L2 DHRS7 DHRS7B DHX30 DHX9 DICER1 DKFZP434C153 DLAT DLC1 DLEC1 DLG3 DLG4 DNAH3 DNAI1 DNAI2 DNAJB14 DNAJC10 DNAJC16 DNALI1 DNASE2B DOCK2 DOCK4 DOCK9 DOHH DOK2 DOM3Z DOPEY1 DPAGT1 DRAM1 DST DSTN DTX2 DUOX1 DUSP1 DYNC1LI2 E2F3 EBI3 ECM1 EDEM3 EDNRB EEF1B2 EEF1D EFCAB1 EFHC1 EFTUD1 EGR2 EHBP1 EHD1 EIF1AX EIF2AK1 EIF2AK2 EIF2S3 EIF4B EIF4G1 EIF5 EIF5A ELF3 ELK1 ELK4 ELL2 ELL3///SERINC4 ELMO3 EMP1 EMP2 EMR2 EMR3 ENG ENOSF1 ENPP2 ENTPD1 EPB41L1 EPB41L3 EPB41L4B EPB41L5 EPCAM EPHA2 EPHX1 EPOR EPS8L1 EPS8L2 ERAP1 ERBB2 ERC1 EREG ERLIN1 ESR1 ETV1 EVC EVI2A EVI2B EVX1 EXOC3 EXOC7 EXT2 FA2H FABP2 FADS1 FAIM3 FAM102A FAM105A FAM125B FAM127B FAM129A FAM13A FAM149A FAM153A FAM160B2 FAM171A1 FAM49A FAM57A FAM65B FANCA FARP2 FAS FASLG FBXL15 FBXO22 FBXO46 FCAR FCGR1A///FCGR1C FCGR1B FCGR2A FCGR2B FCGR2C FCGR3A///FCGR3B FCGR3B FCN1 FCN2 FDXR FEZ2 FEZF2 FFAR2 FGD2 FGL2 FGR FH FIG4 FKBP11 FKBP8 FKSG49 FLNA FLRT2 FN1 FOLR1 FOSL2 FOXA1 FOXJ1 FOXN2 FPR1 FPR2 FPR3 FURIN FUT3 FUT6 FXYD3 FYB FZD5 G0S2 G6PC3 GAB2 GABARAPL1 GABRD GAD1 GADD45B GAGE12F///GAGE12G///GAGE12I///GAGE5///GAGE7 GAK GALK2 GALNT6 GALNT7 GARNL3 GATA3 GATM GBP1 GC GCA GCFC1 GCH1 GCN1L1 GDAP2 GDF15 GDF2 GGTLC2 GIN1 GK GK///GK3P GK3P GLA GLB1L2 GLI1 GLIPR1 GLRX3 GLYR1 GM2A GMDS GMFB GMFG GMPPB GNA11 GNAI3 GNAS GNAT1 GNAT2 GNG11 GNLY GOLGA8A GOLGA8H GORASP1 GOSR1 GOT1 GP1BB GPAA1 GPC1 GPNMB GPR109B GPR171 GPR172A GPR18 GPR183 GPR22 GPR3 GPR56 GPR65 GPR89A///GPR89B///GPR89C GPRC5C GPS1 GPSM3 GPX5 GRAMD3 GRIN1 GRIN2B GRM6 GRSF1 GSPT1 GSTA1 GTDC1 GTF3C5 GTSE1 GUCY1B3 GZMA GZMB H1FX H3F3A///LOC440926 H3F3B HAB1 HAL HARS HAUS5 HBA1///HBA2 HBEGF HCFC1 HCLS1 HDAC6 HEMK1 HERC2P2///HERC2P9 HERC5 HERPUD1 HES1 HEXIM1 HGS HIBCH HIF3A HIP1 HIPK1 HIST1H1C HIST1H3C HLA-DQA1 HLA-DQB1 HMG20B HMHA1 HN1L HNMT HNRNPA0 HNRNPD HNRNPH1 HOPX HP///HPR HPCAL1 HPSE HRAS HRASLS2 HRG HS3ST2 HSD11B1 HSD17B11 HSD17B6 HSF1 HSP90AB1 HSP90B1 HSPA6 HSPD1 HTR2A HTR5A HTR7P1 HYMAI HYOU1 ICAM1 ICAM2 ICMT ICOS ID2 IDE IDS IER5 IFI44L IFIH1 IFIT1 IFIT3 IFITM1 IFITM2 IFNA14 IFNA17 IFNA4 IFNA7 IFRD1 IFT122 IFT140 IFT27 IFT88 IGF1 IGFBP2 IGHD///IGHG1///IGHM IGK@///IGKC IGK@///IGKC///IGKV1-5 IGSF6 IKBKAP IL10RA IL13RA1 IL18RAP IL1A IL1B IL1R2 IL1RAPL2 IL1RN IL27RA IL2RB IL6R IL6ST IL7R IL8 INADL ING2 INHBA INHBB INO80B INPP5D INPPL1 INTS6 IQCG IRAK4 IRF1 IRF3 IRF8 ITGA4 ITGAM ITGAX ITGB1 ITGB3 ITK ITM2A ITPA ITPR2 ITPR3 ITSN1 JAKMIP2 JMJD6 JUND KATNB1 KCNAB1 KCNAB2 KCNJ15 KCNJ2 KCNJ5 KCNMA1 KCTD12 KCTD13 KDM4C KDM5A KDM6B KIAA0090 KIAA0101 KIAA0182 KIAA0509 KIAA1967 KIF13B KIR2DL1 KIR2DL5A KIR2DS2 KIR2DS5 KIR3DL1///KIR3DL2///LOC727787 KIR3DL3 KLC1 KLHDC3 KLRD1 KLRK1 KMO KRIT1 KRT18 KRT19 KRT2 KRT23 KRT38 KRT7 KRT76 KRT8 LAIR1 LAMA4 LAPTM4B LAPTM5 LAT2 LCMT1 LCN2 LCP1 LCP2 LDLR LEPROT LGALS2 LGALS8 LGI2 LGMN LGR5 LILRA5 LIMCH1 LIMS1 LMNA LMNB1 LMO3 LOC100133321 LOC100288679 LOC100294402///SIGIRR LOC100505503///RPS17 LOC100505650 LOC100507328 LOC100507328///LOC100508591 LOC100509558///LOC100510047 LOC150759 LOC150776///SMPD4 LOC157627 LOC399491 LOC442381///LOC650293///OR7E87P LOC80054 LOC91316 LOXL1 LPCAT1 LPCAT4 LPHN3 LPL LRBA LRRC41 LRRC47 LRRC48 LRRC50 LRRC6 LSM14B LSP1 LSR LST1 LTB LTBP4 LXN LY86 LY9 LY96 LYN MACF1 MACROD1 MAFB MAFF MAGEA9///MAGEA9B MAGEC1 MAN1A1 MAN2C1 MAP1B MAP2K5 MAP3K4 MAPK10 MAPK13 MAPK14 MAPK8 MARCKS MBD3 MBNL1 MBP MCAT MCL1 MCM3AP MCRS1 MDH2 MECOM MED1 MED13L MED15 MED16 MED24 MED8 METT11D1 METTL5 MFAP5 MFNG MFSD10 MGEA5 MGRN1 MICAL3 MID1 MID1IP1 MKI67 MLEC MLL MLPH MMD MMP1 MMP12 MNDA MOBKL1B MORC2 MOSPD3 MPP1 MPP2 MPZ MR1 MRC1///MRC1L1 MREG MRPL12 MRPL24 MRPL52 MRPS12 MRPS2 MRPS7 MS4A1 MS4A4A MSI1 MSLN MSMB MSR1 MST1///MST1P2///MST1P9 MTA1 MTERFD2 MTF2 MTHFD1 MTHFS MTR MTX2 MUC1 MUC16 MUC4 MUC5AC MUTED///TXNDC5 MXD3 MXD4 MYH10 MYH14 MYO1C MYO5C MZF1 N4BP1 N6AMT1 NAA40 NACC2 NADSYN1 NAMPT NBN NCAM1 NCF2 NCF4 NCK2 NCKAP1L NCS1 NDUFS8 NEDD9 NEK11 NET1 NEU3 NEUROG2 NF2 NFAT5 NFKB2 NFKBIA NFKBIB NHLH2 NIPSNAP3B NIT2 NMBR NME5 NMT1 NOL3 NOLC1 NOMO1///NOMO2///NOMO3 NPDC1 NPEPL1 NPHP4 NPIPL3 NPL NR1D2 NR1H3 NR4A3 NRG1 NRG2 NRIP1 NRP1 NRXN1 NSMAF NSMCE4A NTAN1 NTRK3 NUCKS1 NUP188 NUTF2 NYX OAS1 OAS2 OAS3 OASL OBSL1 OGDH OGFOD2 OLFML2B OLIG2 OLR1 OPHN1 OPLAH OR1F1 OR2B2 OR7C1 ORAI2 ORC4 ORM1 ORM1///ORM2 OSBPL3 OSBPL8 OSTM1 OTOR OVGP1 OXR1 P2RY13 P2RY14 P4HB P4HTM PABPC1///RLIM PABPN1 PACRG PADI4 PALLD PAN2 PAX8 PBRM1 PBX2 PCDH1 PCDHGA1///PCDHGA10///PCDHGA11///PCDHGA12///PCDHGA2///PCDHGA3///PCDHGA4///PCDHGA5///PCDHGA6///PCDHGA7///PCDHGA8///PCDHGA9///PCDHGB1///PCDHGB2///PCDHGB3///PCDHGB4///PCDHGB5///PCDHGB6///PCDHGB7///PCDHGC3///PCDHGC4///PCDHGC5 PCDHGA10///PCDHGA11///PCDHGA12///PCDHGA3///PCDHGA5///PCDHGA6 PCGF3 PCIF1 PCNT PCNX PCOLCE2 PCYT2 PDCD11 PDCD6 PDE12 PDE1C PDE4B PDGFC PDGFD PDLIM1 PDLIM4 PDP1 PDPK1 PDZK1IP1 PEA15 PECAM1 PEX14 PEX5 PFDN4 PFDN6 PFKP PFN2 PGLS PGPEP1 PHACTR1 PHACTR2 PHF20L1 PHF3 PHF8 PHKA2 PI3 PI4K2A PIAS1 PICALM PIGO PIH1D1 PIK3CD PIK3R1 PILRB PINK1 PIP4K2C PITRM1 PKN1 PKN2 PKP3 PLA2G10 PLA2G7 PLAC1 PLAU PLAUR PLCB2 PLEC PLEK PLEKHB2 PLEKHM1 PLIN2 PLK3 PLXNB1 PLXNC1 PMAIP1 PMPCA PNKP PNN PNO1 PNPLA3 POLD1 POLG POLR1C POLR1E POLR2C POLR2H POLR3E POLR3K POLRMT POP7 POR PPAP2B PPAP2C PPARG PPBP PPIB PPIF PPL PPM1F PPOX PPP1R12A PPP1R12B PPP1R13L PPP1R3A PPP1R7 PPP2CB PPP3CA PPP3R1 PPP4C PPP4R1 PPT1 PRAME PREB PREPL PRKACB PRKAR2B PRKCSH PRKG1 PRKX///PRKY PRL PRMT7 PRO2012 PRODH PRPF31 PRPF4B PRPSAP1 PRR4 PRSS22 PRUNE PTAFR PTEN PTEN///PTENP1 PTGES2 PTGS1 PTGS2 PTMS PTOV1 PTPN11 PTPN22 PTPRA PTPRC PTPRE PTPRF PTPRO PTPRU PUF60 PUM1 PURA PYGB QKI QPCTL QSOX1 QTRT1 RAB14 RAB1B RAB25 RAB27A RAB31 RAB36 RAB7L1 RABAC1 RABGAP1 RAC2 RAD51L3 RAGE RALA RALGAPA1 RALGAPB RANBP1 RANBP2 RAP2A RAPGEF2 RASA1 RASGRF1 RASGRP1 RASGRP2 RASSF2 RBBP6 RBM14 RBM38 RBM5 RBP4 RBPJ RECK RECQL RECQL5 RELN RETN RFX1 RGS1 RGS2 RGS4 RGS6 RHBDF1 RHEB RHOH RHOQ RIF1 RIMS3 RIOK3 RIPK2 RMND5A RNASE4 RNF19A RNF208 RNF40 ROD1 RPL10 RPL10A RPL13 RPL14 RPL15 RPL19 RPL21 RPL23 RPL23A RPL24 RPL26 RPL27 RPL27A RPL28 RPL29 RPL3 RPL31 RPL35A RPL37 RPL37A RPL38 RPL39 RPL4 RPL5 RPL7 RPL9 RPLP0 RPLP0///RPLP0P6 RPLP2 RPN1 RPP25 RPS10 RPS11 RPS14P3 RPS15 RPS15A RPS17 RPS18 RPS19 RPS2 RPS20 RPS21 RPS25 RPS26 RPS27 RPS27A RPS29 RPS3 RPS6 RPS7 RPS9 RRAD RRBP1 RSAD2 RSL1D1 RSL24D1 RSRC1 RTDR1 RUNX3 RUVBL2 RYBP S100A13 SAFB2 SAMSN1 SBF1 SCAMP4 SCARB2 SCARF1 SCD SCD5 SCG5 SCGB2A1 SCNN1A SCRIB SDC2 SDF4 SEC13 SEC14L1 SEC22B SEC31A SEC61A1 SEL1L SELENBP1 SELL SEMA3B SEMA3C SEMA4C SEMA6D SENP2 SEPW1 SERINC3 SERPINB9 SERPINF1 SERPING1 SERPINI1 SETD4 SF1 SF3A2 SF3B1 SFN SFTPC SGSM3 SH3BP1 SH3BP4 SH3BP5 SH3GL3 SH3GLB2 SHQ1 SIGIRR SIGLEC1 SIGLEC6 SIGMAR1 SIRPA SKAP2 SLA SLAMF8 SLC12A3 SLC12A7 SLC16A1 SLC16A5 SLC16A6 SLC17A1 SLC18A1 SLC19A3 SLC1A6 SLC25A16 SLC25A36 SLC25A37 SLC25A6 SLC2A10 SLC2A14///SLC2A3 SLC2A3 SLC30A3 SLC30A5 SLC31A2 SLC35A2 SLC35E1 SLC38A10 SLC39A6 SLC39A8 SLC39A9 SLC47A1 SLC4A2 SLC4A4 SLC6A10P///SLC6A8 SLC6A2 SLC6A8 SLC7A5 SLC7A6 SLC7A7 SLC7A8 SLCO2B1 SLITRK3 SMAD3 SMAD5 SMARCA4 SMARCB1 SMARCC2 SMARCD1 SMC5 SMCHD1 SMG6 SMG7 SMPD2 SNAPC4 SNRPB SNRPD3 SNRPE SNX10 SNX13 SNX27 SOD2 SOLH SORBS2 SOX13 SOX15 SOX2 SP100 SP110 SPAG6 SPAG8 SPARC SPATA2 SPATS2L SPDEF SPEF1 SPEN SPG7 SPIN1 SPINT1 SPN SPOCK2 SPP1 SPPL2B SPR SPRR1A SPTAN1 SQSTM1 SRC SRGN SRPK1 SRPX2 SRRM2 SRRT SRSF2IP SRSF4 SSH1 SSSCA1 SSX2IP SSX4///SSX4B ST14 ST5 ST6GALNAC2 STAG2 STAT1 STAT5B STAT6 STATH STEAP4 STK11 STK16 STK25 STRA13 STRA6 STRN4 STX16 STX18 SUCLG1 SULT1A1 SUPT5H SUPT7L SYBU SYN1 SYNCRIP TACC2 TACSTD2 TAF1 TAF10 TAF6L TANK TAPBP TARP TARP///TRGC2 TARP///TRGV3///TRGV5 TAS2R13 TAS2R16 TBC1D13 TBC1D2B TBC1D4 TBCB TBCD TCEB3 TCF15 TCF3 TCF7L2 TCTN2 TECR TEKT2 TERT TEX28 TFCP2L1 TFEC TFF3 TFIP11 TFR2 TH1L THBD THUMPD1 TICAM1 TIMM44 TJP3 TLE6 TLR1 TLR2 TLR4 TLR8 TM7SF4 TM9SF1 TMC5 TMED3 TMEFF1 TMEM120B TMEM140 TMEM14A TMEM176A TMEM176B TMEM231 TMEM53 TMEM57 TMEM62 TMEM63A TMEM87A TMEM93 TMPRSS4 TMX1 TNF TNFAIP6 TNFRSF10C TNFRSF13B TNFRSF1A TNFRSF1B TNFSF12 TNIK TNPO1 TOMM40 TPBG TPM2 TPMT TPP1 TPPP3 TPR TPSAB1 TPSAB1///TPSB2 TRA2A TRAC TRAC///TRAJ17///TRAV20 TRAK1 TRAPPC2L TRBC1 TRBC1///TRBC2 TRD@ TREM1 TREX1 TRIM10 TRIM14 TRIM2 TRIM25 TRIM28 TRIM3 TRIM37 TROAP TRPM3 TRPM4 TSC2 TSNAXIP1 TSPAN1 TSPAN13 TSPAN3 TSPAN8 TSPY1 TSSK2 TTC12 TTC21B TTC3 TTC39A TTLL5 TTTY15 TUBA1B TUBA4B TUBG1 TUBGCP2 TULP2 TULP3 TUSC3 TWF1 TXLNA TXN UBA6 UBAC1 UBE2B UBE2D1 UBE2J1 UBQLN4 UCKL1 UFD1L UNC119B UNC13B UQCRC1 UROS USF2 USP12 USP15 USP22 USP33 USP34 USP48 UTP14C VAMP3 VAPA VAPB VAV1 VCAN VDR VGLL1 VNN2 VPS33B VRK3 VSIG4 WAC WDR59 WDR70 WDR74 WFDC2 WFS1 WIPF1 WIPI2 WNT10B WNT6 WRAP53 WSB2 WSCD1 XAB2 XRCC2 YIF1A YIPF3 YY1 ZBBX ZBED4 ZBTB38 ZBTB48 ZC3H7B ZDHHC11 ZDHHC13 ZDHHC24 ZDHHC4 ZEB2 ZFAND5 ZFPL1 ZFR ZFYVE26 ZMYND10 ZMYND8 ZNF142 ZNF148 ZNF160 ZNF192 ZNF200 ZNF24 ZNF266 ZNF267 ZNF282 ZNF286A ZNF331 ZNF580 ZNF593 ZNF646 ZNF673 ZNF747 ZNF749 ZNF768 ZNF816 ZNF91 ZNHIT2 | BAL_19_A0B0 BAL_21_A0B0 BAL_27b_A2B1 BAL_44a_A0B0 BAL_44b_A0B0 BAL_15b_A0B0 BAL_45b_A2B0 BAL_46_A2B0 BAL_15a_A0B1 BAL_41_A1B0 BAL_12_A1B0 BAL_31_A0B1 BAL_27a_A0B0 BAL_25_A0B0 BAL_29_A1B0 BAL_40_A0B0 BAL_47a_A0B0 BAL_13_A0B0 BAL_33_A0B1 BAL_1_A0B0 BAL_26_A2B0 BAL_17_A0B0 BAL_47c_A0B1 BAL_45a_A1B0 BAL_3_A1B0 BAL_37_A1B1 BAL_4_A0B0 BAL_43_A0B0 BAL_39_A0B1 BAL_47b_A0B1 BAL_20b_A1B1 BAL_42_A0B0 BAL_18_A1B0 BAL_20a_A1B1 |
| **2** | ABCB6 ABCD3 ABCG1 ABHD2 ABHD5 ABLIM1 ACAD8 ACLY ACO1 ACOT9 ACP1 ACP5 ACSF2 ACSL1 ACSL3 ACTN1 ACTR2 ACTR3 ADAM10 ADAM17 ADAM28 ADAM9 ADAMDEC1 ADCY7 ADD3 ADH7 ADM ADSL AES AFF1 AGAP1 AGFG1 AGPAT2 AGR2 AGXT AHCYL2 AHNAK2 AIF1 AKAP1 AKAP10 AKAP12 AKAP13 AKAP2///PALM2-AKAP2 AKAP9 AKR1B10 AKR1C1 AKR1C2 ALAS1 ALDH18A1 ALDH3A2 ALDH6A1 ALDOC ALOX5 ALOX5AP ALPK1 AMBRA1 AMD1 AMFR AMIGO2 AMMECR1 ANKRD11 ANKRD12 ANKRD36 ANKRD36B ANP32A///ANP32D ANP32E ANXA3 AP1G2 AP1S2 AP2A2 APOL1 APOO APPL2 APRT AQP9 ARAP1 AREG ARF4 ARFGEF2 ARG2 ARGLU1 ARHGDIA ARHGDIB ARID1A ARL2 ARL3 ARL6IP4 ARMC1 ARMCX3 ARPC2 ARPC5 ARRB2 ASPH ASPHD1 ASPM ATF3 ATF6 ATG3 ATN1 ATOX1 ATP10A ATP10D ATP1B1 ATP2A2 ATP2B2 ATP2B4 ATP5E ATP5G2 ATP6V0E2 ATP6V1B2 ATP6V1G2///BAT1 ATP7B ATPAF2 ATPIF1 ATR ATXN10 AUH AZGP1 B4GALT4 B4GALT5 BACH1 BAG1 BAG3 BAIAP2 BAZ1A BBS7 BCAP29 BCL2A1 BCL2L14 BCL3 BCL6 BEND5 BHLHE40 BICD2 BLOC1S1 BMPR1B BMPR2 BRE BRF2 BTBD3 BTD BUB1 C10orf116 C10orf57 C11orf75 C11orf9 C12orf5 C13orf15 C13orf18 C14orf109 C14orf156 C16orf42 C16orf80 C18orf10 C19orf22 C19orf26 C1orf112 C1orf135 C1orf38 C1QA C1QB C22orf9 C2CD3 C5AR1 C5orf15 C6 C6orf108 C6orf48 C7orf58 C8orf33 C8orf4 C8orf44///SGK3 CA12 CA2 CA5BP CABYR CADM3 CALCA CALCOCO2 CALM2 CALR CAMK1 CAP1 CAPN9 CAPZA2 CASP1 CASP3 CASP8 CBFB CBR1 CBX7 CC2D1A CCDC19 CCDC69 CCL18 CCL20 CCNA1 CCNB1IP1 CCR1 CCR8 CD14 CD163 CD24 CD36 CD37 CD44 CD46 CD47 CD52 CD53 CD55 CD58 CD59 CD69 CD74 CD83 CDC14B CDC27 CDC42 CDC42BPA CDC42EP3 CDK2AP1 CDYL CEACAM1 CEACAM5 CEBPA CEBPB CELF2 CENPF CENPM CEP290 CES1 CFB CFD CFI CFL1 CFLAR CHCHD8 CHL1 CHMP2B CHRNA5 CHST15 CHST7 CINP CIT CKAP4 CLCA2 CLCA4 CLCN3 CLDN10 CLEC2B CLEC4A CLEC7A CLIC4 CLIC5 CLINT1 CLIP1 CLMN CLN6 CLU CMAH CNN3 CNOT8 COL21A1 COLEC12 COQ2 CORO1A CORO1C COX5B COX7C COX8A CPA3 CPD CPM CPVL CRELD2 CREM CROCC CROCCP2 CROCCP3 CSDA CSE1L CSF1R CSF2RB CSGALNACT2 CSN2 CSNK1A1 CST6 CTAGE11P CTAGE5 CTBP2 CTNNA1 CTNND1 CTSB CTSH CXCL1 CXCL10 CXCL11 CXCL12 CXCL14 CXCL2 CXCL3 CXCL5 CXCL6 CXCL9 CXCR4 CYBB CYP1A1 CYP1B1 CYP26B1 CYP2B6 CYP2B6///CYP2B7P1 CYP2C9 CYP3A5 CYP4B1 CYP4F11 CYP4F2 CYP4F3 CYR61 CYTIP DAAM1 DAB2 DAG1 DAPK1 DDAH2 DDR1 DDX17 DDX49 DEFB1 DEFB4A DEGS1 DENND4C DENND5A DEXI DHCR24 DHRS7B DHX15 DHX35 DHX40 DLG1 DLGAP4 DMD DNAH9 DNAI1 DNAJB9 DNAJC10 DNAJC12 DNAJC16 DNALI1 DNASE1L3 DOCK2 DOK1 DOK5 DPY30///MEMO1 DPYSL2 DPYSL3 DRAM1 DROSHA DSC2 DSE DUOX2 DUSP3 DUSP5 DUSP6 DYNLL1 DYNLRB1 DYNLT3 DYRK2 ECHDC3 EDEM1 EEF1D EEF1G EFEMP1 EFHC2 EFHD2 EFR3B EGFL6 EGR1 EGR3 EI24 EIF2AK2 EIF2AK3 EIF3G EIF3H EIF3K EIF3L EIF4A1 EIF4B EIF5B ELF4 ELK1 ELK3 ELMO1 ELMO2 EMD EML4 EMP1 EMP3 EMR2 ENPP4 EPAS1 EPM2A EPS8 ERBB2 ERBB3 EREG ETNK1 EVI2B EXOSC10 F11R F2RL1 FABP6 FADD FAM134B FAM13A FAM172A FAM173A FAM179B FAM3C FAM49B FAM50A FAM55D FAM65B FAM82A2 FAR2 FARSA FAU FBL FBLN1 FBP1 FBXL15 FBXO3 FBXO34 FCER1G FCGR1A///FCGR1C FCGR1B FCGR2A FCGR2C FCGR3A///FCGR3B FCGR3B FEM1B FERMT1 FFAR2 FGFBP1 FGFR1OP FGFR3 FHL1 FKBP11 FKBP1A FKBP1B FLJ23519///RNH1 FLRT2 FLVCR2 FMO3 FN1 FNDC3B FOLR1 FOSB FOSL2 FOXF2 FOXN2 FOXN3 FPR1 FPR2 FSCN1 FUS FUT3 FUT6 FUT8 FXR1 FXYD3 G0S2 G3BP2 GABBR1///UBD GAD1 GADD45A GAGE1///GAGE12F///GAGE12G///GAGE12I///GAGE12J///GAGE2A///GAGE2B///GAGE2C///GAGE2D///GAGE2E///GAGE3///GAGE4///GAGE5///GAGE6///GAGE7///GAGE8 GAGE1///GAGE12F///GAGE12G///GAGE12I///GAGE12J///GAGE4///GAGE5///GAGE6///GAGE7 GAGE12C///GAGE12D///GAGE12E///GAGE12F///GAGE12G///GAGE12H///GAGE12I///GAGE2A///GAGE2C///GAGE4///GAGE5///GAGE6///GAGE7 GAK GALNT3 GALNT6 GALNT7 GBAP1 GBP1 GBP2 GCA GCAT GCH1 GCLC GCLM GCNT3 GDF15 GDI1 GEMIN4 GFPT1 GGA2 GK GLB1 GLB1L GLB1L2 GLIPR1 GLS GLT8D1 GLTSCR2 GMDS GMFB GMFG GML GNA12 GNAL GNE GNG10 GOLGA2 GOLGA6L5///GOLGA6L9 GPN2 GPNMB GPR109B GPR172A GPR63 GPR65 GPX2 GPX4 GRAMD3 GRM1 GRN GSDMB GSPT1 GSTA1 GTSE1 GUCY1B3 GULP1 GUSBP3 GZMA GZMB H2BFS HBA1///HBA2 HBB HBE1 HBEGF HBG1///HBG2 HCFC1R1 HCK HCLS1 HCP5 HDAC9 HEBP2 HECA HERC2P2///HERC2P9 HERPUD1 HEXB HIPK1 HIST1H2AC HIST1H2BE HIST1H2BK HIST2H2BE HK2 HLA-DMA HLA-DPA1 HLA-DQA1 HLA-DQA1///HLA-DQA2 HLA-DQB2 HLA-DRB4///LOC100509582 HMGA1 HMGCS2 HMGN3 HMGN4 HMGXB4 HMOX1 HNMT HNRNPD HNRNPH2 HP1BP3 HPRT1 HPS5 HS3ST1 HSD17B14 HSP90AA1 HSPA1A///HSPA1B HSPA5 HSPB1 HSPB11 HSPC072 HTR2C HUWE1 HYOU1 ICAM1 ID2 IDO1 IDS IER2 IER3 IER5 IFI30 IFIH1 IFITM1 IFITM2 IFITM3 IFNB1 IFNGR1 IFRD1 IGBP1 IGF2BP3 IGF2R IGFBP3 IGFBP7 IGHA1///IGHA2///LOC100126583 IGHA1///IGHG1///IGHG2///IGHG3///IGHM///LOC100126583///LOC100290036 IGHG1///IGHG2///IGHM///IGHV4-31 IGK@///IGKC IGK@///IGKC///IGKV1-5 IGSF6 IL13RA1 IL1A IL1B IL1R2 IL1RN IL2RG IL6ST IL8 IMPA2 INHBA INPP1 INPP4B IPO8 IPW IQGAP1 IQSEC1 IRAK3 IRF1 IRF8 IRS2 ITGA4 ITGA6 ITGAM ITGAV ITGB4 ITGB6 ITIH2 ITSN1 JAG1 JUND KAL1 KAT5 KBTBD4///PTPMT1 KCNJ15 KCNJ2 KCNN3 KCTD7 KDELR2 KDELR3 KDM4C KDM5B KDR KIAA0101 KIAA0226 KIAA0485 KIAA0907 KIAA1598 KIF16B KLF10 KLF11 KLF4 KLHDC10 KLK11 KMO KPNA2 KPNB1 KRAS KRT13 KRT15 KRT18 KRT19 KRT23 KRT24 KRT5 KRT6A KRT6A///KRT6B///KRT6C KRT6B KRT9 KTN1 KYNU L3MBTL1 LAIR1 LAMC1 LAMP1 LAMP2 LAPTM4B LAPTM5 LAT2 LBH LCP1 LCP2 LDHA LDLR LEPROT LGALS13 LIPA LITAF LMO3 LOC100505503///RPS17 LOC100506076///LOC100506123 LOC100507804///TPSAB1 LOC100509749 LOC100510735///RPL29 LOC150759 LOC220594 LPAR1 LPCAT4 LPL LPXN LRBA LRCH4 LRIT1 LRP10 LRRC23 LRRC37A3 LRRC48 LRRC50 LRRC59 LTA4H LTB4R LUC7L3 LY6D LY96 LYN MAB21L1 MACROD1 MAFB MAFF MAGEA12 MAL MALT1 MAN1A1 MAN2A1 MANSC1 MAOA MAP2K1 MAP2K5 MAP2K6 MAP7D1 MAP9 MAPK1 MAPRE3 MAPT MARCKS MARCKSL1 MARCO MB MBD5 MBOAT7 MBP MCL1 MCOLN1 MCTP2 MDM1 MECP2 MED24 MEGF9 METRN METTL3 MEX3D MFSD7 MICA///MICB MICAL2 MICB MID1 MID1IP1 MITF MKNK1 MLX MMD MME MMP7 MNDA MOBKL1B MOBKL3 MORF4L2 MPZL1 MPZL2 MRC1///MRC1L1 MRPL18 MRPL23 MRPL40 MRPS22 MRPS33 MS4A1 MS4A4A MS4A6A MSH3 MSL1 MSMB MSN MSR1 MT1E MT1X MTA1 MTCH2 MTCP1NB MTDH MTHFD2 MTMR2 MTUS1 MUC1 MUC13 MUC16 MUC5AC MUTED///TXNDC5 MYCBP MYD88 MYL10 MYO1D MYO6 MYO7A MYOZ1 MYOZ2 N4BP1 NAB2 NACA NAGA NAMPT NAT1 NCF2 NCF4 NCK1 NCR2 NDN NDST1 NDUFA3 NDUFA6 NDUFB2 NEAT1 NEDD9 NEK1 NEK2 NENF NET1 NFE2L2 NFIL3 NFYC NIPSNAP1 NOP10 NPC1 NPIPL3 NPL NQO1 NR2F2 NR2F6 NR3C1 NR4A1 NR4A2 NR4A3 NRG2 NRIP1 NRP1 NUCB1 NUCB2 NUDC NUP50 NUPR1 OAT OCM2 OLR1 OPN3 OR1A1 OR2J2 OR7E14P OSBPL11 OSBPL3 OSBPL8 OSTM1 P2RX4 P2RX7 P4HA2 PABPN1 PAICS PAIP1 PAK2 PALLD PAM PARP12 PAWR PCDHGA10///PCDHGA11///PCDHGA12///PCDHGA3///PCDHGA5///PCDHGA6 PCM1 PCOLCE2 PDCD2 PDE12 PDE4B PDE4DIP PDIA4 PDLIM5 PDXDC1 PDZK1IP1 PECAM1 PECI PELI1 PER2 PERP PFDN5 PFDN6 PFN1 PGK1 PGRMC1 PHF11 PHGDH PHLDA2 PHLDA3 PI3 PI4K2A PICALM PIK3C2B PIK3CG PILRB PION PIR PKIG PKP4 PLAT PLAUR PLBD1 PLCB1 PLCB4 PLD1 PLEK PLIN2 PLOD2 PLSCR1 PLUNC PLXNC1 PMAIP1 PMP22 PMS2P1 PNP PNPLA6 POGK POLD2 POLDIP3///RRP7B POLG POLQ POLR1E POLR2K POLR2L POLRMT POMGNT1 PON1 PON2 PPAP2C PPARG PPBP PPIC PPP2CA PPP3CA PPP3CB PPP5C PPPDE1 PQBP1 PRICKLE4///TOMM6 PRKACB PRKCI PRKRIP1 PROS1 PRPF4B PRPF6 PRPF8 PRR4 PRRG4 PSD3 PSMD4 PSMD5 PTEN PTGES PTGS2 PTHLH PTK2 PTK6 PTN PTP4A1 PTPN1 PTPN11 PTPN12 PTPRC PTPRE PTPRF PTPRH PTPRO QKI QPCT QPRT RAB11A RAB11FIP1 RAB11FIP3 RAB21 RAB27A RAB2A RAB5A RAB5C RAB8B RABEPK RABGAP1L RABGGTB RAC1 RAC2 RANBP3 RAP1A RAP1B RAP1GDS1 RAP2B RAP2C RARRES1 RASA1 RASSF2 RBM23 RBM3 RBMS3 RBMX2 RBP4 RCN2 RDH11 RFC5 RFTN1 RFX5 RGS1 RGS10 RGS19 RGS2 RHEB RHOB RHOBTB3 RHOD RHOQ RINT1 RIT1 RNASE4 RND3 RNF103 RNF13 RNF19B RNF39 RNGTT RPA1 RPL10 RPL10A RPL11 RPL12 RPL13 RPL13A RPL13A///RPL13AP5///RPL13AP6 RPL14 RPL15 RPL17 RPL18 RPL18A///RPL18AP3 RPL19 RPL21 RPL22 RPL23 RPL23A RPL24 RPL26L1 RPL27 RPL27A RPL28 RPL29 RPL3 RPL30 RPL31 RPL32 RPL34 RPL35 RPL36A RPL37A RPL38 RPL4 RPL5 RPL7A RPL8 RPL9 RPLP0 RPLP1 RPLP2 RPN2 RPS10 RPS11 RPS12 RPS13 RPS14 RPS14P3 RPS15 RPS15A RPS18 RPS19 RPS2 RPS20 RPS21 RPS25 RPS26 RPS3 RPS3A RPS4X RPS4Y1 RPS5 RPS6 RPS6KA3 RPS7 RPS9 RRAD RRH RRM2 RRP7A RSU1 RTEL1 RTEL1///TNFRSF6B RTN1 RTN4 RUFY3 RUSC1 RUVBL2 RWDD1 S100A12 S100A13 S100A2 S100A8 S100A9 SAA1///SAA2 SAA4 SAMD4A SAMSN1 SARS SART1 SASH3 SCD SCEL SCGB1A1 SCN4A SDC2 SEC23A SECISBP2L SECTM1 SELENBP1 SELL SEMA4D SEMA4G SEMA5A SERBP1 SERINC5 SERPINA1 SERPINB1 SERPINB13 SERPINB2 SERPINB3 SERPINB4 SERPINB9 SERPING1 SF1 SF3A3 SGCG SGPP1 SH2B3 SH3BGRL3 SH3BP4 SH3GLB2 SHOX2 SIGLEC9 SIK1 SIRPA SKAP2 SLA SLC11A1 SLC12A5 SLC15A3 SLC16A3 SLC16A7 SLC19A1 SLC19A3 SLC20A1 SLC25A24 SLC25A36 SLC25A37 SLC25A46 SLC25A6 SLC26A2 SLC26A4 SLC2A14///SLC2A3 SLC2A3 SLC30A1 SLC30A10 SLC31A2 SLC33A1 SLC34A2 SLC35A3 SLC35D1 SLC38A6 SLC39A14 SLC4A1AP SLC4A4 SLC5A3 SLC7A1 SLC7A11 SLC7A7 SLC9A6 SLC9A8 SLCO2B1 SLPI SMAD1 SMAD6 SMARCA4 SMARCAL1 SMARCE1 SMC1A SNAP23 SNRPA1 SNRPN///SNURF SNUPN SNX1 SNX10 SNX2 SOBP SOD2 SORBS2 SORD SORL1 SOX2 SOX4 SPAG1 SPAG6 SPANXB1///SPANXB2///SPANXF1 SPATA7 SPG21 SPI1 SPOCK2 SPP1 SPRR1A SPRR1B SPRR3 SPTBN1 SPTLC1 SPTLC2 SQRDL SRD5A1 SREK1 SRGN SRP72 SRPK1 SRPR SRPRB SRPX2 SRSF2 SRSF2IP SSR1 SSX7 ST14 ST3GAL2 ST3GAL6 ST5 ST6GAL1 ST6GALNAC2 STAT1 STATH STAU1 STC1 STEAP1 STIL STK17A STK17B STK38 STS SULT2A1 SYNCRIP SYNGR2 SYNJ2 SYTL2 TAAR2 TAAR3 TAP1 TBC1D4 TBR1 TBXAS1 TCEB2 TCF4 TCF7L2 TCN1 TEKT2 TES TEX12 TF TFEC TFF1 TFPI TFPT TFRC TGFBI TGIF1 THBD THBS1 THYN1 TIAM1 TIMP1 TIMP3 TJP2 TLE1 TLN1 TLR2 TLR4 TM4SF1 TM6SF1 TM9SF1 TMBIM4 TMED5 TMEM110 TMEM177 TMEM222 TMEM30A TMEM33 TMEM38B TMEM45A TMSB4X///TMSL3 TNFAIP2 TNFAIP3 TNFAIP6 TNFRSF10C TNFRSF21 TNFSF10 TNFSF12-TNFSF13///TNFSF13 TNIP1 TOB1 TOR1B TOX3 TPD52 TPK1 TPM1 TPP1 TPPP3 TPSAB1 TPSAB1///TPSB2 TPSB2 TRAC///TRAJ17///TRAV20 TRAK1 TRAK2 TREM1 TRIB1 TRIB2 TRIM16 TRIM22 TRIM31 TRIM37 TRIM38 TRMT1 TSEN34 TSPAN1 TSPYL4 TTC19 TTC3 TTTY2 TUBB2A TUBB2C TUBB3 TUBB6 TUG1 TWF1 TXN TYROBP UBA52 UBE2J1 UBE4B UBXN1 UCHL1 UCP2 UGCG UGDH UGT1A1///UGT1A10///UGT1A3///UGT1A4///UGT1A5///UGT1A6///UGT1A7///UGT1A8///UGT1A9 ULK1 UNC93B1 UPK1B UQCRH USE1 USP14 USP15 UTP14C UTP18 UTRN VAMP3 VCAN VCP VDAC1 VDAC3 VEGFA VEGFC VILL VNN1 VPS16 VPS28 VPS37B VSIG4 VWA5A WASL WBP11 WBP5 WDR45L WDR52 WDR74 WEE1 WFDC2 WIPF1 WRB WSB1 WSB2 XBP1 XIST XRCC4 YES1 YIPF6 YME1L1 YWHAZ ZBTB44 ZC3H12A ZDHHC11 ZEB2 ZFAND5 ZFP36L1 ZKSCAN1 ZMAT5 ZMIZ1 ZMYM2 ZMYM5 ZMYND10 ZNF236 ZNF238 ZNF273 ZNF32 ZNF34 ZNF365 ZNF442 ZNF451 ZNF467 ZNF592 ZNF652 ZNF667 ZSCAN18 | Sample_58_current_smoker_without_cancer BAL_41_A1B0 BAL_20a_A1B1 BAL_31_A0B1 BAL_15b_A0B0 BAL_29_A1B0 BAL_1_A0B0 BAL_20b_A1B1 BAL_15a_A0B1 BAL_47a_A0B0 BAL_27a_A0B0 BAL_47c_A0B1 BAL_45b_A2B0 BAL_19_A0B0 BAL_21_A0B0 BAL_3_A1B0 BAL_47b_A0B1 BAL_44a_A0B0 BAL_12_A1B0 BAL_18_A1B0 BAL_13_A0B0 BAL_37_A1B1 BAL_44b_A0B0 BAL_42_A0B0 BAL_25_A0B0 BAL_17_A0B0 BAL_46_A2B0 BAL_4_A0B0 BAL_40_A0B0 BAL_43_A0B0 BAL_27b_A2B1 BAL_26_A2B0 BAL_33_A0B1 BAL_45a_A1B0 BAL_39_A0B1 |
| **3** | AACS AATK ABCA12 ABCA8 ABCB11 ABCB6 ABCD1 ABHD2 ABI2 ACADSB ACAP2 ACIN1 ACN9 ACOXL ACSF2 ACTB ACTG1 ACTN1 ACTR6 ADAM2 ADAM22 ADAM23 ADCY10 ADD1 ADH1B ADH7 ADIPOQ ADK ADRA1B AFF3 AFP AGXT AHCY AHCYL2 AHSG AKAP12 AKAP8 AKR1B10 AKR1C1 AKR1C2 AKR1C3 AKR1C4 AKR1D1 ALDH1A3 ALDH3A1 ALDH3B2 ALDOB AMACR AMMECR1 ANAPC13 ANG ANK2 ANKRD36 ANKRD36B ANKRD36BP2 ANKRD5 ANP32A ANP32B ANP32E ANPEP ANXA3 ANXA4 ANXA6 AP2A2 AP3D1 APBB2 APOBEC3F APOC1 APOE APOF AQP4 AREG ARHGAP4 ARHGDIA ARHGEF12 ARID1A ARIH2 ARL17A///ARL17B ARL4C ARPC2 ART3 ASB4 ASCL2 ASMTL ASPH ASRGL1 ATF3 ATG4B ATP11A ATP1A2 ATP1B4 ATP2A2 ATP5A1 ATP6AP2 ATP6V0A4 ATP6V0E1 ATP6V0E2 ATP6V1G2///BAT1 ATP8A2 ATXN3 AZGP1 B3GALT2 B4GALT1 B4GALT4 B4GALT5 BACE2 BARD1 BBX BCL2L11 BCL2L14 BCL6 BCLAF1 BEST1 BLMH BLZF1 BMP5 BNIP3L BOP1 BPTF BRCA1 BRD4 BRSK2 BTBD2 BTBD7 BTG1 BTN2A3 BUB1 C10orf12 C10orf137 C10orf84 C11orf30 C12orf47 C14orf1 C14orf105 C14orf139 C15orf63///SERF2 C17orf86 C19orf40 C1orf107 C1orf56 C21orf2 C22orf9 C2orf27A C2orf3 C3orf14 C4orf10 C6orf26///MSH5 C6orf48 C6orf54 C7orf64 C7orf69 C8orf4 C8orf79 C9 C9orf38 CA12 CABP2 CABYR CACNA1C CACNA1G CACNG4 CALCOCO1 CALCRL CALD1 CALM1 CALML3 CALR CAPN5 CAPRIN2 CARD10 CASP3 CAST CATSPERB CBR1 CBR3 CBX7 CCDC102B CCDC144A CCDC85C CCDC92 CCNA1 CCND2 CCNI CCPG1 CD164 CD2AP CD4 CD40 CD47 CD74 CD81 CD99 CDC14B CDC20 CDC42BPB CDC5L CDH1 CDH5 CDH9 CDIPT CDK1 CDK19 CDK5RAP2 CDKAL1 CDKL2 CDKN2A CDKN2B CDR1 CDYL CEACAM5 CEACAM6 CEACAM7 CEBPB CECR1 CELSR1 CENPF CENPT CEP135 CEP152 CEP350 CFD CFLAR CHD1L CHD7 CHD9 CHERP CKAP4 CLC CLCA3P CLCA4 CLDN10 CLDN14 CLDN7 CLINT1 CLPTM1 CLUL1 CMTM6 CNGB1 CNOT7 CNTD2 CNTF///ZFP91///ZFP91-CNTF CNTN6 COL4A3 COL4A3BP COPA COPB2 COPG COPS8 COX5B COX7C CPA3 CPEB3 CPLX2 CR2 CRABP1 CRBN CREB3L1 CRHR1 CRIP1 CRIPT CRISP3 CRP CRYBB2///CRYBB2P1 CSE1L CSNK1D CSNK2A2 CST3 CSTA CSTF1 CSTF2 CTBP1 CTDSP2 CTDSPL CTGF CTNNA1 CTSH CTSZ CXCL11 CXCL14 CXCL6 CXorf27 CXXC1 CYFIP2 CYLC1 CYLD CYP1A1 CYP1B1 CYP24A1 CYP39A1 CYP3A5 CYP4F11 CYP4F2///CYP4F3 CYP4F3 CYP7A1 DAZ1///DAZ2///DAZ3///DAZ4 DBNDD1 DCAF15 DCAF8 DCLK1 DCT DCTN1 DCTN2 DDAH2 DDX17 DDX49 DDX5 DDX50 DEFB1 DEFB4A DENND1C DENND5B DGKE DGKG DHPS DHRS12 DHRS3 DHRS7 DHX35 DIAPH2 DICER1 DIMT1L DIRAS2 DKC1 DLG1 DLGAP1 DLX2 DLX5 DNAJC10 DNAJC12 DNMT3B DOCK9 DOT1L DPP3 DPP8 DPPA4 DPY19L1P1 DPYSL3 DRAP1 DRD4 DSG1 DSG3 DST DSTNP2 DSTYK DUOX2 DUT DYRK2 ECE1 ECT2 EDA EDDM3A EDNRA EDNRB EEF1A1 EEF1D EEF2 EFNA4 EHBP1L1 EHD1 EHMT2 EIF1 EIF2AK3 EIF3D EIF3F EIF3K EIF4G2 EIF4G3 EIF5A EIF5B EIF6 ELAVL1 ELF1 ELF2 EML3 EMP3 EN1 ENOSF1 ENPEP EPB41 EPB41L1 EPHA3 EPHB6 EPM2AIP1 EPN2 EPOR EPS8L1 ERAP1 ERBB4 ERCC6L ESD ESM1 ESR1 ESR2 ETV1 EXOC7 EXT2 EZH1 F2RL1 FA2H FABP4 FABP5 FAM107A FAM111A FAM114A1 FAM120A FAM135A FAM193A FAM198B FAM21A///FAM21B///FAM21C FAM36A///HOXA7 FAM45A///FAM45B FAM50A FAM53C FAM5C FAM76A FAT4 FBN1 FBN2 FBXO11 FBXO22 FCGR2C FETUB FGF12 FGF18 FGF2 FGFBP1 FGFR2 FHOD3 FKBP11 FLJ11292 FLJ13224 FLOT2 FLRT3 FLT1 FMO2 FOLH1 FOLH1B FOLR1 FOXJ2 FOXK2 FOXL1 FOXN3 FRZB FUT2 FUT3 FUT6 FXYD6 G0S2 G6PC GABARAP GABBR1///UBD GABRA5///LOC100509612 GABRA6 GABRG2 GAD1 GAD2 GADD45B GAL GALE GALK2 GALNT3 GALNT6 GALNT7 GAR1 GAS1 GATA6 GC GCHFR GCLC GCLM GCM2 GCNT3 GDF15 GDI1 GFPT1 GGA1 GIF GINS2 GIP GLP1R GLRA3 GLRX3 GLUL GMDS GMNN GMPR2 GNAL GNAS GNE GNG13 GNG7 GNL3L GNLY GOLGA7 GOLGA8A GOLGA8B GOLPH3L GON4L GPM6A GPR153 GPR3 GPR37 GPX2 GPX4 GREB1 GREM1 GRID2 GRIK2 GRINA GRM8 GSTM3 GSTT2 GTF2A1L///STON1-GTF2A1L GTF2H2///GTF2H2B///GTF2H2C///GTF2H2D GTF2I///GTF2IP1///LOC100093631 GTF3C2 GUCY1B2 GULP1 GYPA GYPE H2AFV H2AFX H2BFS H3F3B HAPLN1 HAUS6 HBG1///HBG2 HBP1 HDAC6 HDAC9 HDGFRP3 HEATR6 HERC6 HERPUD1 HGD HGF HINT1 HIPK1 HIST1H2AC HIST1H2AI HIST1H2BB HIST1H2BD HIST1H2BE HIST1H2BF HIST1H2BG HIST1H2BH HIST1H2BI HIST1H2BK HIST1H4F HIST1H4H HIST1H4J///HIST1H4K HIST2H4A///HIST2H4B HLA-A HLA-DOA HLA-DPA1 HLA-DPB1 HLA-DQB2 HLA-DRA HLA-DRB1///HLA-DRB4///HLA-DRB5 HMBOX1 HMGB1 HMGCS1 HMP19 HN1L HNRNPA1 HNRNPA3 HNRNPD HNRNPK HNRNPR HNRNPU HOXA10 HOXB1 HOXB9 HOXD3 HPR HRC HRG HS3ST1 HS3ST3A1 HSBP1 HSF2 HSP90AA1 HSP90B1 HSPA1A///HSPA1B HSPA4L HSPB1 HSPC072 HTATIP2 HTR7 HUS1 HUWE1 ICA1 ICK IDE IDH2 IDH3B IDO1 IFI6 IFNAR2 IGF1 IGF2///INS-IGF2 IGF2BP3 IGHA1///IGHA2///IGHD///IGHG1///IGHG3///IGHG4///IGHM///IGHV4-31///IGHV4-59///LOC100126583 IGHA1///IGHA2///IGHD///IGHG1///IGHG3///IGHM///IGHV3-48///IGHV4-31///LOC100291917 IGHA1///IGHA2///IGHG1///IGHG4///IGHM///IGHV4-31 IGHA1///IGHG1///IGHG3///IGHM///IGHV4-31///LOC100510678 IGHG1 IGHM///LOC100133862 IGHV5-78 IGJ IGLL5///IGLV2-11 IGLV6-57 IK///TMCO6 IL12B IL19 IL25 IL4R IL6R IL7R IMPA2 ING3 IPO9 IPPK IQCB1 IQSEC1 IREB2 IRF4 IRS4 ITGA1 ITGA6 ITGB1 ITGB3 ITGB8 ITM2A ITM2B ITSN1 IVD JAG1 JAG2 JUND KAL1 KCNAB1 KCND3 KCNJ2 KCTD12 KCTD13 KCTD7///RABGEF1 KDELR3 KDM5B KDM6B KDSR KERA KHDC1L KHDRBS1 KHSRP KIAA1024 KIAA1467 KIAA1659 KIF20A KIF23 KIF2A KLC1 KLF12 KLHDC3 KLHL20 KLRC3 KPNA5 KPNB1 KRAS KRT13 KRT14 KRT19P2 KRT23 KRT24 KRT35 KRT4 KRT6A KRT6A///KRT6B///KRT6C KRT6B KRTAP1-1 KYNU L3MBTL1 LAGE3 LAMA2 LAMB1 LARP7 LARS2 LCE2B LCN1 LDLR LEPREL4 LEPROT LETM1 LGALS7///LGALS7B LGALS8 LGSN LHX6 LIMD1 LMAN2 LMO2 LMO7 LOC100129648 LOC100287076 LOC100287927 LOC100288142///NBPF1///NBPF10 LOC100290070 LOC100505960 LOC100506076///LOC100506123 LOC100506168 LOC100506935 LOC100507328 LOC100507328///LOC100508591 LOC150759 LOC150776///SMPD4 LOC283079 LOC389906 LOC441601 LOC442421///LOC728297 LOC51152 LONP2 LPA LPAR1 LPCAT4 LRP12 LRP1B LRRC16A LRRC19 LRRC31 LRRFIP1 LTBP3 LUC7L3 LY6D LY75 LYPD3 LZTS1 MAB21L2 MAGEA4 MAGEB1 MAGEB4 MAGEC1 MAGED4///MAGED4B MAGT1 MAML1 MAML3 MANEA MAP2K3 MAP2K5 MAP3K13 MAP4 MAPK11 MAST3 MATN3 MATR3 MAX MBD4 MBP MCF2L2 MCFD2 MCL1 MCM3AP MCM4 MDC1 MDM2 ME1 MECP2 MED24 MED6 MEFV MEOX2 MEP1A MET METT11D1 METTL2A///METTL2B METTL5 METTL9 MFAP3L MGC13053 MGC3771 MGLL MGRN1 MIA MID1IP1 MINK1 MKRN1 MKRN3 MLL MLLT3 MLLT4 MMP13 MMP16 MMP27 MMS19 MOCS1 MORC4 MPO MPPED2 MPZL2 MRPL22 MRPL52 MRPS18A MS4A2 MSL1 MSL3 MSLN MSMB MSRB2 MSTN MT1F MT1G MTAP MTCH1 MTDH MTFR1 MTM1 MTMR2 MTTP MUC1 MUC13 MUC2 MUC5AC MUC5B MUTED///TXNDC5 MYCBP MYH1 MYL10 MYLK3 MYO1B MYO3A MYOT MYST3 N4BP2L1 N4BP2L2 N6AMT1 NAALAD2 NACA NAP1L1 NAPA NAT8B NAV3 NBPF10 NBPF10///NBPF11///NBPF12///NBPF15///NBPF16///NBPF24///NBPF8///NBPF9 NBPF10///NBPF12///NBPF15///NBPF16///NBPF8///NBPF9 NCOA1 NCOR2 NDUFA8 NEK2 NEK9 NET1 NEUROD6 NF1 NF2 NFASC NFATC3 NFKB1 NFKBIA NID1 NKTR NKX2-1 NKX3-1 NLGN1 NLRP1 NMU NOP16 NOTCH1 NOVA1 NOX1 NPDC1 NPY2R NQO1 NR0B1 NR2C1 NR3C1 NRAP NRCAM NRG1 NRXN2 NSL1 NTRK2 NUCB1 NUP98 NXF1 OAT OGG1 OLFML1 OLIG2 OPCML OR2B6 OR2J2 ORC5 OS9 OSBPL10 OSBPL3 OVOL1 P2RY10 P2RY6 PABPC1 PACS2 PAICS PAIP1 PAM PAPSS1 PARG PARVB PAX4 PAX8 PBRM1 PCBP2 PCCA PCDHA2 PCDHGA10 PCLO PDAP1 PDE10A PDE3A PDE4A PDE4DIP PDE5A PDGFRL PDIA4 PDK3 PDLIM5 PDX1 PDXK PDZK1 PEBP1 PECI PELI1 PEX14 PF4V1 PFDN5 PFKL PGLS PHF14 PHF16 PHF7 PHLDA2 PI4KB PIBF1 PIGO PIGR PIK3CD PIK3R1 PIK3R4 PIP5K1A PIR PIWIL2 PKM2 PKNOX1 PKP2 PLA2G10 PLA2G16 PLA2G4A PLA2G5 PLA2R1 PLAT PLD1 PLEKHF1 PLK3 PLK4 PLTP PLXNA1 PLXNC1 PMAIP1 PML PMS2P3 PNLIPRP2 PNO1 POLDIP3 POLH POLR3G POR POU2F3 PP14571 PPAP2B PPEF2 PPFIBP1 PPIA PPIB PPIE PPIF PPP1R7 PPPDE1 PQLC1 PRB1 PRB4 PRDX1 PRIM2 PRKAA1 PRKAB1 PRKAR1A PRKCI PROS1 PROSC PRPF40A PRPF8 PRR4 PSCA PSD3 PSENEN PSG1 PSG4 PSMB1 PSMC2 PSMD5 PSME3 PSORS1C2 PSPH PTEN PTGER3 PTHLH PTK6 PTN PTP4A1 PTP4A2 PTPN11 PTPN20A///PTPN20B PTPRH PUM2 PVRL1 PYCR1 PYGO1 PYY QSER1 RAB11A RAB3GAP1 RAB3GAP2 RAB5B RAD23B RAG2 RALGDS RAP1GAP RAPGEF3 RAPGEF4 RBBP4 RBM23 RBM34 RBM39 RBM42 RBM5 RBP3 RBPJ RCAN1 RCHY1 RCN2 RDX REEP5 RELA REPS1 REXO2 RFNG RFX7 RGS12 RGS13 RHOB RHOBTB3 RIMBP2 RIMS2 RIN2 RLN1 RNASEH1 RND3 RNF113A RNFT2 RNMT RNPS1 ROR1 RPAIN RPE65 RPL11 RPL14 RPL15 RPL22 RPL26L1 RPL27A RPL37A RPL38 RPL5 RPL6 RPLP2 RPS11 RPS15 RPS2 RPS3A RPS6 RPS6KA1 RPS6KA5 RRP15 RSAD1 RTCD1 RTEL1///TNFRSF6B RTN4 RUNX1 RXRA RYBP RYR2 S100A11 S100A14 S100A8 S100P SALL1 SAMD14 SCAND2 SCARB1 SCGB1A1 SCML2 SCN11A SCN1A SCPEP1 SEC14L1 SEC14L3 SEC24D SELPLG SERINC3 SERP1 SERPINB13 SERPINB2 SERPINB4 SERPINB5 SESN1 SET SF1 SF3B4 SFN SFRS18 SFTPC SFXN3 SGCB SGCD SH3BGRL SH3YL1 SHANK2 SHC3 SHFM1 SHMT2 SHOX2 SIK2 SIM1 SIRT5 SLC12A1 SLC12A2 SLC13A1 SLC14A1 SLC15A1 SLC16A3 SLC22A3 SLC24A1 SLC25A1 SLC25A17 SLC25A21 SLC25A36 SLC25A37 SLC25A6 SLC26A4 SLC29A1 SLC30A1 SLC30A5 SLC35A3 SLC35E2B SLC35F5 SLC37A1 SLC38A10 SLC4A10 SLC4A7 SLC5A12 SLC6A2 SLC6A6 SLC7A11 SLC9A3 SLIT2 SLIT3 SMARCA2 SMC4 SMEK2 SMPDL3B SMPX SNCA SNN SNRNP200 SNRPE SNRPG SON SOX2 SOX3 SPAG1 SPANXB1///SPANXB2///SPANXF1 SPATS2L SPDEF SPOCK3 SPON1 SPOP SPRR1A SPRR1B SPRR3 SPTBN1 SPTBN4 SRD5A3 SRPX2 SRRM1 SRRM2 SRSF11 SRSF5 SRSF6 SRSF7 SSRP1 SSX2IP SSX5 ST8SIA4 STAG2 STAP1 STAT1 STAT5A STAT5B STAT6 STATH STEAP3 STK25 STK3 STRA13 STS STX2 STXBP2 SUMO2 SUPT16H SUPT6H SUSD5 SUZ12P SYCE1L SYCP1 SYDE1 SYNJ2 SYNPO2L T TADA3 TAF10 TAF13 TAF1B TALDO1 TAS2R14 TAT TBC1D10B TBC1D2B TBC1D5 TBCB TBL1X TBX1 TBX2 TBX3 TCN1 TEF TELO2 TERF2 TERF2IP TEX12 TF TFE3 TFF1 TFF3 TFPI TFR2 THOC5 THOP1 THUMPD1 TIMELESS TIMP3 TINAGL1 TJP2 TLE1 TLE3 TLL1 TLR3 TLX2 TM4SF1 TM4SF20 TM9SF4 TMC6 TMEFF1 TMEM183A///TMEM183B TMEM19 TMEM47 TMEM5 TMEM80 TMPRSS2 TMPRSS4 TMSB4Y TNFAIP3 TNFRSF14 TNFSF10 TNIP1 TNIP3 TNK2 TNKS TNNI3 TNNT3 TNPO1 TNS1 TOM1L1 TOP3A TP53 TP53AIP1 TPBG TPD52 TPD52L1 TPH1 TPMT TPR TPT1 TPTE TRBC2 TRD@ TRDN TRIM10 TRIM16 TRIM28 TRIM31 TRIM49///TRIM49L2 TRIO TRMT112 TSC22D2 TSC22D3 TSPAN9 TSPY1///TSPY3///TSPY4///TSPY8 TSSK2 TSTA3 TTC18 TTC22 TTF1 TTF2 TTLL12 TUG1 TWF1 TWISTNB TXN TXNRD1 UBE2L3 UBL4A UCHL1 UCKL1 UGT1A1///UGT1A10///UGT1A3///UGT1A4///UGT1A5///UGT1A6///UGT1A7///UGT1A8///UGT1A9 UGT1A1///UGT1A10///UGT1A4///UGT1A6///UGT1A8///UGT1A9 UGT2A3 UGT2B15 UGT2B17 UMPS UPF3A UPK1B UQCRC1 UQCRQ URB1 USP4 USP46 USP6 UTP14A VAMP2 VCAM1 VEGFB VEZT VGLL1 VPS13C VPS13D VPS33A VPS37B VPS53 VPS72 WARS WBP2 WBP5 WDHD1 WNK1 WSB1 WSB2 WWP1 XBP1 XPNPEP1 XPO6 XYLB YARS2 YES1 YTHDC1 YWHAB ZC3H15 ZCCHC11 ZCCHC24 ZFAND5 ZFP2 ZFP30 ZFP36L1 ZFP36L2 ZFPL1 ZHX2 ZHX3 ZNF12 ZNF141 ZNF148 ZNF157 ZNF22 ZNF234 ZNF263 ZNF287 ZNF292 ZNF323 ZNF407 ZNF43 ZNF467 ZNF528 ZNF552 ZNF623 ZNF643 ZNF674 ZNF771 ZNF816 ZP2 ZXDA///ZXDB | BAL_27b_A2B1 BAL_44a_A0B0 BAL_29_A1B0 BAL_31_A0B1 BAL_47b_A0B1 BAL_47c_A0B1 BAL_41_A1B0 BAL_47a_A0B0 BAL_39_A0B1 BAL_15b_A0B0 BAL_20b_A1B1 BAL_42_A0B0 BAL_12_A1B0 BAL_25_A0B0 BAL_40_A0B0 BAL_43_A0B0 BAL_27a_A0B0 BAL_18_A1B0 BAL_33_A0B1 BAL_13_A0B0 BAL_37_A1B1 BAL_45b_A2B0 BAL_1_A0B0 BAL_45a_A1B0 BAL_15a_A0B1 BAL_4_A0B0 BAL_44b_A0B0 BAL_3_A1B0 BAL_26_A2B0 BAL_17_A0B0 |
| **4** | ABAT ABCA5 ABCC1 ABCC4 ABCD1 ABCD3 ABHD2 ABR ACACB ACADM ACADVL ACE ACP2 ACSBG1 ACTB ACTR3 ACTR6 ACVR1B ACYP1 ADAM19 ADAM28 ADAM8 ADCY2 ADCY9 ADD3 ADH1C ADH6 ADH7 ADM ADRBK1 AGAP1 AGL AGRN AHCYL1 AHI1 AHNAK AHNAK2 AHSA1 AK1 AKAP9 AKR1C2 AKR7A2 ALCAM ALDH1A1 ALDH3A2 ALDH3B1 ALDH5A1 ALDH6A1 ALDH7A1 ALG13 ALMS1 ALOX15 AMMECR1 AMY1A///AMY1B///AMY1C///AMY2A///AMY2B ANAPC5 ANGEL2 ANK3 ANKHD1///ANKHD1-EIF4EBP3 ANKRA2 ANKRD11 ANKRD12 ANKRD36B ANKRD46 ANKRD6 ANXA4 AP1S2 AP2B1 AP3M2 APBB1IP APEX2 APOBEC3A APOD APOE APOL6 APP APPL2 APRT AQP3 ARF1 ARF3 ARGLU1 ARHGAP26 ARHGDIA ARHGEF12 ARHGEF2 ARIH1 ARL1 ARL4A ARL6IP4 ARNT ASCC2 ASCL1 ASMTL ASTN2 ATF7IP2 ATG5 ATP1A1 ATP2A2 ATP5A1 ATP5I ATP5L ATP5O ATP6V0A1 ATP6V0E1 ATP8B1 ATP9A ATPIF1 ATRN ATRX ATXN10 ATXN7L3B AUH AUTS2 AVIL AXL AZIN1 B3GALT4 BAG1 BAG3 BAK1 BAT2L1 BAT2L2 BBOX1 BBS1 BBS4 BBS9 BCAM BCCIP BCKDK BCL2L1 BCL3 BCL6 BDH2 BIN3 BLCAP BMI1 BMPR1B BMS1 BNIP3 BNIP3L BPGM BPTF BRD2 BRD8 BSCL2 BTBD3 BTF3 BUD31 C10orf26 C10orf57 C10orf81 C11orf2 C11orf63 C11orf67 C11orf95 C12orf10 C12orf29 C14orf104 C14orf132 C14orf156 C14orf2 C15orf39 C15orf5 C15orf63///SERF2 C16orf7 C16orf80 C17orf91 C18orf10 C19orf2 C19orf22 C19orf42 C19orf6 C1orf114 C1orf135 C1orf25 C1orf66 C1QBP C20orf12 C21orf33 C21orf59 C21orf91 C2orf28 C2orf67 C2orf68 C3 C3orf64 C4orf46///TOMM7 C5orf15 C6 C6orf103 C6orf106 C6orf108 C6orf130 C6orf26///MSH5 C6orf97 C8orf33 C9orf95 CALHM2 CALM1 CALM2 CALML4 CAMLG CAPN2 CARS CASC1 CASC3 CBX5 CBX7 CCDC21 CCDC41 CCDC56 CCDC69 CCDC72 CCDC81 CCL14-CCL15///CCL15 CCL20 CCL3///CCL3L1///CCL3L3 CCL4 CCNB1IP1 CCNB2 CCND1 CCR2 CCR5 CCRL1 CCT2 CCT3 CCT6A CCT6B CD14 CD2AP CD300A CD55 CD59 CD6 CD84 CD93 CD97 CDC14B CDC16 CDC25B CDC42BPA CDKN1C CDKN2C CDS1 CEACAM1 CEBPZ CELSR1 CENPE CEP290 CEP57 CEP68 CES1 CES1P1 CES2 CETN2 CETN3 CFDP1 CH25H CHD4 CHERP CHL1 CHMP1B CHMP2A CHN2 CHP2 CHRNA5 CIB1 CIRBP CKB CKS1B CLCA2 CLDN8 CLMN CLSTN1 CLU CLUAP1 CMAH CNOT2 COIL COL21A1 COL4A5 COL4A6 COL7A1 COPS6 COPS8 CORO1A CORO2B COX5B COX6A1 COX6B1 COX6C COX7A1 COX7C CPD CRBN CREB5 CRIM1 CRISP2 CRK CROCCP2 CROT CSDA CSDE1 CSE1L CSF3R CSK CSNK2A2 CSPP1 CST7 CSTA CTDSPL CTNNAL1 CTNND1 CTSC CUL5 CUTA CX3CL1 CXADR CXCL12 CXCL13 CXCL2 CXCR2 CXCR4 CXorf57 CYB5A CYB5R1 CYBA CYFIP2 CYP2A6 CYP2B6 CYP2F1 CYP4B1 CYP51A1 CYTH1 CYTH4 DAAM1 DALRD3 DAZAP2 DCI DCLRE1C DCTN5 DDAH1 DDC DDIT4 DDX1 DDX17 DDX18 DDX24 DDX3X DDX42 DDX50 DENND1C DENR DGCR2 DGCR6///DGCR6L DHCR24 DHRS7B DHX40 DIAPH1 DIAPH2 DKK3 DLG5 DMBT1 DMD DNAH7 DNAH9 DNAJB1 DNAJC12 DNAJC16 DPH5 DPM1 DPM3 DROSHA DSP DST DSTN DSTYK DUOX2 DUSP1 DUSP5 DUSP6 DVL1 DYNC2H1 DYNC2LI1 DYNLL1 DYNLRB1 DYNLT1 DYRK2 DYSF DZIP3 E2F3 EAPP EBAG9 EBNA1BP2 EBP ECHDC2 ECHS1 ECSIT EDF1 EDNRA EEF1A1 EEF1A1///EEF1A1P9 EEF1B2 EEF1D EEF1G EEF2 EFCAB1 EFCAB2 EFCAB6 EFEMP1 EFHC1 EFHC2 EFHD2 EFNB2 EFNB3 EFS EGFR EGR3 EHBP1 EHBP1L1 EHD1 EHF EID1 EIF2B5 EIF3C///EIF3CL EIF3D EIF3E EIF3F EIF3K EIF3L EIF5B ELF5 ELK1 ELK3 ELN EMP1 EMR2 ENG ENOSF1 ENSA ENTPD1 EP400 EPAS1 EPHX1 EPM2AIP1 EPS15L1 EPS8 ERBB4 ERCC3 ERGIC3 ERLIN2 ESF1 ETFB EWSR1///FLI1 EXOSC8 EXPH5 EZR F13A1 FABP6 FAHD2A FAIM FAM107A FAM117A FAM134B FAM13A FAM149A FAM164A FAM168B FAM172A FAM179B FAM18B1 FAM38B FAM3C FAM50B FAM65B FAM86B1 FAM8A1 FAN1 FARP1 FARS2 FAU FBL FBN1 FBXO11 FBXO21 FBXO3 FBXO9 FBXW12 FCER1A FCER1G FCF1///LOC100507758///MAPK1IP1L FCGBP FCGR2B FCGR3B FDPS FDXR FERMT2 FFAR2 FGF14 FGFR1OP FGFR2 FGFR3 FHOD3 FIP1L1 FIS1 FKBP3 FKBP9 FLJ13197 FLOT1 FLRT3 FMNL1 FMO2 FMO3 FMO5 FN1 FNTA FOLR1 FOS FOSL2 FOXN3 FOXO3 FPR1 FSTL1 FTL FTO FXR1 FXYD1 FYB FYCO1 FYN FZD1 G0S2 G6PD GADD45B GAGE12F///GAGE12G///GAGE12I///GAGE5///GAGE7 GALNS GALNT3 GAPDH GAPVD1 GAR1 GAS6 GAS7 GATM GBAS GBP2 GCH1 GCLC GCLM GCSH///LOC100329108 GEMIN4 GGPS1 GIMAP5 GIPC2 GK GLE1 GLT25D1 GLT8D1 GLTPD1 GLTSCR2 GLUL GMNN GNA11 GNA15 GNAI1 GNAL GNAQ GNAS GNB2L1 GNG11 GNG12 GOLGA2 GOLGA6L5///GOLGA6L9 GOLGA8A GOLGB1 GOSR1 GOSR2 GP1BB///SEPT5 GPATCH1 GPD1L GPR172A GPR183 GPR97 GPRC5B GRAMD1C GRAMD3 GRAMD4 GRINA GRK6 GRP GSTA4 GSTM2 GTF2H3 GTF2H5 GTF2I///GTF2IP1///LOC100093631 GTSE1 GULP1 GVINP1 H1F0 H1FX H3F3B HCK HDAC1 HDAC5 HDGFRP3 HEATR2 HEBP2 HEY1 HEY2 HHAT HIBCH HINT1 HIP1 HIPK1 HIST1H2BK HIST1H4C HK3 HLA-B HLA-C HLA-DRB4///LOC100509582 HLA-G HLF HMGB1 HMGCR HMGN1 HMGN3 HMGXB4 HN1 HN1L HNRNPA0 HNRNPA3///HNRNPA3P1 HOOK1 HOXA5 HPSE HSBP1 HSD17B7 HSD17B8 HSF2 HSP90AA1 HSPA2 HSPA4 HSPA6 HSPA8 HSPB11 HSPD1 HSPH1 IARS ICAM1 ICAM3 ICT1 ID2///ID2B IDO1 IER3 IFI30 IFITM2 IFITM3 IFT140 IFT20 IFT57 IFT74 IFT81 IFT88 IGBP1 IGF1R IGFBP2 IGFBP5 IGFBP7 IGHA1///IGHA2///LOC100126583 IGHG1///IGHG2///IGHM///IGHV4-31 IGLV1-44///LOC100290481 IK IKBKAP IKBKG IKZF1 IL16 IL17RA IL18R1 IL18RAP IL1B IL1R2 IL1RN IL2RG IL33 IL6ST IL7 IL8 IMPDH1 IMPDH2 INHBB INSR INTS5 IPO5 IPP IQCE IQCG IQCH IQCK IRAK3 IRF7 ISCA1 ISG15 ISL1 ISLR ITGAX ITGB3BP ITGB5 ITK ITM2A JAG1 JAG2 JMJD7 JUND KAL1 KANK1 KAT5 KAZ KBTBD4///PTPMT1 KCNJ15 KCNJ16 KCNMB1 KCTD12 KDELR2 KDM3B KDM5B KDM6B KIAA0232 KIAA0485 KIAA0494 KIAA0754 KIAA0907 KIAA1539 KIF3A KIF3B KIF5B KLF2 KLF4 KLF5 KLF7 KLHDC10 KLHDC2 KRIT1 KRR1 KRT10 KRT15 KRT4 KRT5 KRT6A KTN1 LAIR2 LAMP1 LANCL1 LAPTM5 LARP6 LARS LAT///SPNS1 LCMT2 LCP2 LDHB LDLRAP1 LGALS8 LGTN LILRA2 LILRA6 LILRA6///LILRB3 LILRB1 LILRB2 LILRB3 LIMK2 LITAF LMO2 LMO4 LOC100127972 LOC100129250 LOC100505503///RPS17 LOC100506168///SFPQ LOC100506517 LOC100507666///NPIPL2 LOC100509558///LOC100510047 LOC100509749 LOC100510712///MTX1 LOC100510735///RPL29 LOC285359///PDCL3 LOC285830 LOC390940 LOC441259///PMS2L2///PMS2P1///PMS2P6 LOC441454///LOC728026///PTMA///PTMAP5 LOC728392///NLRP1 LOC730101 LONP1 LONP2 LONRF3 LPCAT1 LPPR3 LRBA LRIG1 LRRC42 LRRC47 LRRC49 LSM1 LSM14A LSM5 LSP1 LST1 LTB LTB4R LTBP3 LTF LUC7L3 LXN LY75 LYN LZTFL1 MACF1 MAF MAFF MAGEH1 MAGI2 MAN1A2 MAN1C1 MAOB MAP4K1 MAP7 MAP9 MAPK13 MAPKAPK2 MARS MATR3 MB MBNL2 MBTPS1 MCAM MCCC1 MDC1 MDM1 ME1 MEA1 MED6 MEIS3P1 METAP2 METRN METTL7A MEX3D MFAP3L MGMT MGST2 MIA3 MICAL2 MICAL3 MID1 MINA MIOS MIPEP MKI67 MKL2 MKNK2 MLF1 MLL MMP10 MMP14 MMP9 MNS1 MOCOS MORF4L2 MPDU1 MPDZ MPZL2 MRP63 MRPL20 MRPL23 MRPL33 MRPL40 MRPL46 MRPL49 MRPL9 MRPS14 MRPS18B MRPS22 MRPS27 MRPS31 MSH3 MSH6 MT1F MT1G MT1H MT1M MT1P2 MT1X MT2A MT3 MTCP1NB MTIF2 MTMR1 MTMR2 MTSS1 MTUS1 MUC5B MUC7 MUM1 MUT MX2 MXI1 MXRA7 MYB MYCBP MYH10 MYL6B MYO1D MYO1E MYO1F MYO6 MYO7A MYO9B MYST4 MZT2B NAA35 NAA40 NAB1 NACAP1 NACC2 NADK NAE1 NAMPT NANS NAP1L1 NAPA NASP NAT1 NBEA NBPF1///NBPF10///NBPF11///NBPF12///NBPF14///NBPF15///NBPF16///NBPF24///NBPF8 NCDN NCF1///NCF1B///NCF1C NCF1C NCL NCOA2 NCRNA00081 NCRNA00094 NDE1 NDEL1 NDRG3 NDUFA1 NDUFA10 NDUFA13 NDUFA2 NDUFA3 NDUFA4 NDUFA6 NDUFA7 NDUFA8 NDUFAB1 NDUFB1 NDUFB7 NDUFB8 NDUFB8///SEC31B NDUFC1 NDUFC2 NDUFS5///RPL10 NDUFS6 NDUFS8 NDUFV1 NEBL NEK1 NEK4 NELL2 NET1 NFAT5 NFATC2IP NFE2 NFIB NFU1 NFX1 NFYC NGFRAP1 NGRN NIPBL NISCH NKIRAS2 NKTR NLRP3 NME3 NME7 NOL7 NOLC1 NOP56 NOS2 NOSIP NPRL3 NPY2R NR2F2 NR3C2 NRGN NRP1 NSFL1C NSMCE4A NT5E NTS NUAK1 NUBPL NUCB2 NUCKS1 NUDC NUDT15 NUDT18 NUDT4///NUDT4P1 NUP133 NUP50 NXN OAS2 OAT OFD1 OGT OR7E47P ORC3 ORM1///ORM2 OSBPL3 OSM OTUD4 OXR1 OXTR P2RY14 P4HA2 PA2G4 PAFAH1B1 PAIP1 PALLD PAM PAPOLA PARK7 PARP2 PARVB PBLD PBX1 PCDH7 PCM1 PCSK5 PCSK7 PDCD4 PDCD6 PDE4B PDE4DIP PDE8B PDHB PDLIM4 PDLIM7 PEBP1 PER2 PER3 PERP PEX11A PEX3 PEX7 PFDN5 PGAP1 PGD PGRMC1 PHF10 PHF8 PHIP PHLDA1 PI3 PI4KB PIBF1 PIGF PIGH PIGV PIK3R1 PIM1 PIN4 PIP PIP5K1B PIR PITPNC1 PKD1 PKIG PKP4 PLAC8 PLAG1 PLAU PLAUR PLCB4 PLEK PLEKHA1 PLEKHA5 PLEKHB1 PLEKHO1 PLEKHO2 PLK1S1 PLK3 PLP1 PLS3 PLSCR4 PLUNC PLXNA1 PLXNA2 PLXNB2 PLXNC1 PML PMM1 PMS1 PMS2P1 PMS2P3 PNMA1 PNMAL1 POFUT2 POLI POLR1D POLR2I POLR2L PON2 POP1 POSTN POU2AF1 PPIF PPIG PPM1H PPP1R7 PPP2CB PPP3CB PPP4R1 PQLC1 PRB3 PRB4 PRDX2 PRDX6 PRF1 PRKCI PROM1 PROS1 PRPF19 PRPF31 PRPF4B PRPF8 PRPS1 PRPSAP2 PRR11 PRR4 PRSS12 PSCA PSD3 PSIP1 PSMB1 PSMB5 PSMB7 PSMC5 PSMD4 PSMF1 PSPH PSTPIP1 PSTPIP2 PTCH1 PTGFR PTGS2 PTK2 PTMA PTOV1 PTPN12 PTPN18 PTPRZ1 PTS PUM2 PURA QARS QKI RAB27A RAB40B RAB4A RABEPK RABGAP1 RABGAP1L RABL2A///RABL2B RAC2 RAD51L3 RAE1 RALBP1 RALGAPA1 RALGPS1 RARRES1 RASSF2 RB1CC1 RBBP6 RBBP7 RBM25 RCBTB1 RCN2 RDBP RDH14 REEP5 RELB RENBP RERE REV1 RFC1 RGS2 RHBDF2 RHOBTB3 RHOH RIN2 RIOK3 RMI1 RNASE4 RNF187 RNF24 RPA3 RPGR RPL10 RPL10A RPL11 RPL12 RPL13 RPL13A RPL13A///RPL13AP5///RPL13AP6 RPL14 RPL15 RPL17 RPL18 RPL19 RPL21 RPL22 RPL23 RPL23A RPL23AP32 RPL24 RPL26 RPL26L1 RPL27 RPL27A RPL29 RPL3 RPL30 RPL31 RPL32 RPL34 RPL35 RPL35A RPL36 RPL36A RPL37 RPL37A RPL38 RPL39 RPL4 RPL41 RPL5 RPL6 RPL7 RPL7A RPL8 RPL9 RPLP0 RPLP0///RPLP0P6 RPLP1 RPLP2 RPS10 RPS10///RPS10P7 RPS11 RPS12 RPS14 RPS14P3 RPS15 RPS15A RPS16 RPS17 RPS18 RPS20 RPS21 RPS23 RPS24 RPS25 RPS26 RPS27 RPS27A RPS28 RPS29 RPS3 RPS3A RPS4X RPS5 RPS6 RPS6KA6 RPS7 RPS8 RPS9 RPSA RRAD RRAS2 RRM1 RRM2 RUFY3 RXRB RYK RYR3 S100A12 S100A8 S100A9 S1PR4 SAP18 SATB1 SBNO2 SC4MOL SCAMP1 SCGB1A1 SCRN1 SDC4 SDHA SDHC SDR39U1 SEC14L3 SEC62 SECISBP2L SECTM1 SELL SEMA4D SEMA5A SEPP1 SEPT6 SEPX1 SERP1 SERPINA1 SERPINB3 SERPINB3///SERPINB4 SERPINB6 SERPINB8 SERPINB9 SESN1 SET SETD5 SETD6 SF1 SF3B2 SFRS18 SFTPC SFXN3 SGCE SGPL1 SGSH SGSM2 SH3YL1 SHFM1 SHQ1 SIGLEC9 SIRT7 SIVA1 SKP1 SKP2 SLC11A1 SLC12A6 SLC15A2 SLC16A3 SLC17A9 SLC1A4 SLC20A2 SLC22A4 SLC24A1 SLC25A12 SLC25A36 SLC25A37 SLC26A2 SLC27A2 SLC2A10 SLC2A3 SLC30A1 SLC30A9 SLC35A3 SLC35D2 SLC39A6 SLC39A8 SLC4A4 SLC6A16 SLC7A5 SLCO4C1 SLIT1 SLIT2 SLK SLTM SMAD1 SMARCA2 SMARCA4 SMARCE1 SMC3 SMN1///SMN2 SMYD2 SMYD3 SNAI2 SNAPC1 SNRNP200 SNRNP25 SNRPA1 SNRPD2 SNRPD3 SNRPE SNRPG SNRPN///SNURF SNX4 SNX7 SOBP SOCS1 SOCS3 SOD1 SOD2 SORD SOX12 SPA17 SPAG1 SPAG16 SPAG6 SPAG7 SPARCL1 SPATA7 SPEN SPI1 SPINLW1 SPP1 SPRR1B SPTAN1 SPTBN1 SPTLC2 SQLE SRC SRD5A1 SREBF2 SRGAP2 SRGN SRI SRRM2 SRSF3 SRSF6 SRSF7 SSB SSBP2 SSPN SSRP1 SSX4///SSX4B ST6GALNAC2 ST7 STARD8 STAT2 STAT3 STATH STAU1 STEAP1 STEAP3 STIL STK3 STK38 STUB1 STX3 STYXL1 SUCLG1 SUGP2 SULT1A1 SULT1A2 SULT1A3///SULT1A4 SUMO2 SUV420H1 SYF2 SYNE1 SYNE2 SYNJ2BP SYPL1 TACC3 TADA3 TAF15 TAGLN2 TAP1 TAX1BP1 TBC1D22A TBC1D8 TBCA TBL1X TCEA2 TCEAL2 TCEB2 TCEB3 TCF25 TCF4 TCFL5 TCIRG1 TCP1 TEX264 TFAM TFB2M TFCP2 TFDP2 TFEB TFRC THADA THAP10 THAP4 THAP7 THNSL2 THOC2 THUMPD1 THYN1 TIMP1 TIMP3 TJP2 TKT TLE3 TLR2 TLR5 TMBIM6 TMCO3 TMCO6 TMEM121 TMEM123 TMEM131 TMEM134 TMEM14A TMEM158 TMEM212 TMEM231 TMEM30B TMEM41B TMEM45A TMEM5 TMEM59 TMEM87A TMEM97 TMF1 TMSB10 TNC TNF TNFAIP2 TNFAIP3 TNFAIP6 TNFRSF10C TNFRSF1B TNFSF10 TNKS TNPO2 TNRC6B TNS1 TOB1 TOB2 TOMM20 TOP2B TOX3 TP53TG1 TP63 TPD52 TPM1 TPM2 TPP2 TPR TRAC///TRAJ17///TRAV20 TRAK2 TRAPPC2L TRIM13 TRIM2 TRIM68 TRIM8 TRIP13 TRIT1 TRMT61B TROVE2 TSC22D1 TSPAN3 TSPAN6 TSPYL4 TSPYL5 TST TTBK2 TTC12 TTC19 TTC3 TTF1 TTLL1 TUBA1A TUBB2A TUBBP5 TUBGCP2 TUFM TUG1 TULP3 TWF1 TXN TXN2 TYMP TYMS TYROBP UAP1 UBAC1 UBE2I UBE2NL UBE3A UBL3 UBL5 UBXN4 UFC1 UGDH UGT2A1///UGT2A2 ULK2 UNC119B UNC93B1 UPF3A UQCR10 UQCR11 UQCRQ USE1 USP11 USP2 USP34 USP7 UXT VASP VAV3 VDAC3 VEZF1 VGLL4 VIM VPS13B VRK1 WARS WAS WASL WBSCR22 WDR13 WDR19 WDR45L WDR52 WDR61 WDR78 WEE1 WIF1 WIPI1 WIPI2 WLS WNT5A WRB WWTR1 XBP1 YES1 YIPF6 YKT6 YWHAE YWHAZ ZBBX ZBED5 ZBTB16 ZBTB20 ZBTB22 ZBTB7A ZC3H12A ZC3H14 ZC3H7B ZCCHC6 ZDHHC11 ZDHHC18 ZDHHC24 ZFP106 ZFP36 ZFP36L1 ZKSCAN1 ZMYM2 ZMYND8 ZNF12 ZNF146 ZNF148 ZNF160 ZNF177 ZNF20///ZNF625 ZNF204P ZNF207 ZNF23 ZNF232 ZNF238 ZNF24 ZNF268 ZNF273 ZNF3 ZNF302 ZNF32 ZNF329 ZNF395 ZNF432 ZNF44 ZNF440 ZNF544 ZNF638 ZNF91 ZNHIT1 ZSCAN18 ZYX | FormerSmoker_18 FormerSmoker_69 BAL_39_A0B1 BAL_15a_A0B1 BAL_47b_A0B1 BAL_17_A0B0 BAL_3_A1B0 BAL_40_A0B0 BAL_18_A1B0 BAL_41_A1B0 BAL_27a_A0B0 BAL_1_A0B0 BAL_13_A0B0 BAL_20b_A1B1 BAL_25_A0B0 BAL_43_A0B0 BAL_27b_A2B1 BAL_26_A2B0 BAL_44b_A0B0 BAL_37_A1B1 BAL_4_A0B0 BAL_33_A0B1 BAL_29_A1B0 BAL_45a_A1B0 BAL_47a_A0B0 BAL_12_A1B0 BAL_45b_A2B0 BAL_31_A0B1 BAL_47c_A0B1 BAL_15b_A0B0 BAL_21_A0B0 BAL_42_A0B0 BAL_44a_A0B0 BAL_19_A0B0 BAL_46_A2B0 BAL_20a_A1B1 |
| **5** | AASDHPPT ABAT ABCA1 ABCB6 ABCC3 ABHD2 ABHD5 ABHD8 ABLIM1 ACACB ACAT2 ACIN1 ACSL3 ACTB ACTG1 ACTN4 ACTR5 ADAM28 ADAM8 ADAMDEC1 ADCK3 ADCY3 ADD3 ADM ADORA2A///SPECC1L AGFG1 AGR2 AKAP10 AKAP2///PALM2-AKAP2 AKAP7 AKIRIN1 AKT2 AKT3 ALCAM ALDH18A1 ALDH3A2 ALMS1 ALPK1 ALPK3 ALPL ALPP///ALPPL2 AMACR///C1QTNF3 AMBRA1 AMFR AMIGO2 AMPD2 AMPD3 ANAPC1 ANKHD1-EIF4EBP3///EIF4EBP3 ANKRD1 ANKRD36 ANKRD36B ANKRD6 ANP32A///ANP32D ANXA1 ANXA3 AOC3 AP2A2 APC APLP2 APOBEC3A APOBEC3B APOE APOL1 APOL3 APPL1 AQP3 ARAP2 ARF1 ARG2 ARHGAP10 ARHGAP26 ARHGAP32 ARHGAP6 ARHGEF1 ARID5B ARL4A ARL4C ARNTL2 ASAP2 ASB13 ASPH ASS1 ATF1 ATF2 ATF3 ATF4 ATG14 ATP10A ATP12A ATP13A2 ATP1B1 ATP2A2 ATP2A3 ATP2B4 ATP2C1 ATP5L ATP5S ATP6V0E2 ATP6V1G1 ATP6V1G2///BAT1 ATP7A ATP7B AUTS2 AZI2 B2M B3GNT1 B4GALT4 B4GALT5 BACE1 BACE2 BAG3 BASP1 BAT2L2 BATF BAZ1A BAZ2A BCAR3 BCL2L1 BCL3 BCL6 BHLHE41 BIRC3 BMI1 BMP8B BNIP3L BPTF BRD2 BTG1 BTG2 C12orf35 C14orf101 C15orf39 C16orf57 C17orf101 C19orf22 C1orf25 C1R C2orf3 C3 C3orf64 C4orf19 C5 C6orf120 C7orf58 C8B C8orf17///LOC100293561 C8orf4 C9orf16 CA2 CACYBP CAMK2G CAMP CAMSAP1L1 CAMTA1 CAPN1 CAPRIN2 CASP3 CASP4 CASP8AP2 CBLB CBX5 CBX7 CCDC91 CCL13 CCL2 CCL20 CCL3///CCL3L1///CCL3L3 CCL4 CCL5 CCL8 CCND2 CCR2 CCR5 CCR7 CD2 CD24 CD247 CD28 CD300A CD302 CD3D CD3E CD48 CD55 CD5L CD68 CD7 CD72 CD84 CD8A CD8B CD93 CDC16 CDC20 CDC42BPA CDCP1 CDH1 CDK11A///CDK11B CDK14 CDK17 CDKN1A CEACAM1 CEACAM21 CEACAM5 CEACAM6 CEACAM8 CEBPD CEP350 CES1 CFB CFH///CFHR1 CFI CFLAR CHCHD7 CHCHD8 CHI3L1 CHIT1 CHST15 CIT CKAP4 CLC CLCA4 CLCN4 CLDN10 CLDN3 CLEC2D CLEC4E CLIC4 CLN5 CLPB CNOT3 COL3A1 COL6A1 CORO1A COX11 COX15 CP CPA3 CPE CPEB3 CREBL2 CRIM1 CSDA CSE1L CSF3R CSGALNACT1 CSNK1A1 CSNK1G2 CST7 CSTF2T CSTF3 CTBP2 CTGF CTNNA1 CUL2 CX3CR1 CXCL1 CXCL10 CXCL11 CXCL13 CXCL14 CXCL6 CXCL9 CXCR1 CXCR2 CXCR4 CXCR6 CXCR7 CXorf56 CYP1B1 CYP2C18 CYP51A1 CYR61 CYTH4 DAP DAPP1 DBT DCTN1 DDB2 DDIT4 DDO DDX3Y DDX54 DEFA1///DEFA1B///DEFA3 DENND3 DET1 DGKA DICER1 DNAJB1 DNAJC12 DNAJC16 DNAJC6 DNASE1L1 DPYSL3 DSC2 DSP DST DUOX2 DUS4L DUSP1 DUSP2 DUSP4 DUSP5 DUSP6 DYNC1LI2 EBAG9 ECE1 EDDM3A EEA1 EEF1D EGR1 EGR3 EHBP1L1 EHD1 EHF EIF1AX EIF4A1 EIF4G1 ELF3 ELK1 ELP4 EMP1 EMR1 EMR2 ENOPH1 ENOSF1 ENOX2 ENPP2 EPB41L4A EPCAM EPHB6 EPS15 EPS8 ERAP1 ERBB3 ERI2 ERLIN2 ETV1 EVI5 EWSR1///FLI1 EXOSC8 EXPH5 EXTL2 EZR F11R F2RL1 F3 FADS1 FAIM3 FAM108A1 FAM118A FAM129A FAM134B FAM162A FAM35A FAM55C FAM65B FAS FASTKD5 FAT1 FBXL15 FBXL5 FBXL6 FBXO42 FBXO9 FBXW12 FCAR FCF1///LOC100507758///MAPK1IP1L FCGR2B FCGR3B FCN1 FDFT1 FFAR2 FGFBP1 FGFR1 FGFR2 FGL2 FHL1 FHL2 FKBP11 FKSG49 FKTN FLOT1 FNDC3A FOLH1 FOSB FOSL2 FPGT FPR1 FRMD4A FSCN2 FUBP3 FURIN FUS FUT2 FUT3 FUT6 FXYD6 FYB FYN FZD6 G0S2 GABBR1///UBD GABRP GADD45B GALNT3 GAPDH GAPVD1 GATAD1 GBAS GBF1 GBP1 GCC1 GCFC1 GCH1 GCHFR GCLM GCNT1 GCNT3 GCOM1///GRINL1A GCSH///LOC100329108 GDF15 GGA2 GIMAP5 GLG1 GLS GMDS GNB2 GNE GNLY GOLGA2 GOLGA8B GOLIM4 GOLM1 GPD1 GPN3 GPR124 GPR171 GPR173 GPR18 GPR183 GPR97 GPRC5A GTF3A GTSE1 GUK1 GZMA GZMB GZMH GZMK H2AFV H2BFS H3F3A///LOC440926 H3F3B HADH HAUS2 HBA1///HBA2 HBB HBEGF HCP5 HDDC2 HDGFRP3 HEATR3 HERC2P2///HERC2P9 HHEX HIC2 HIF3A HIP1 HIPK1 HIST1H2AC HIST1H2BC HIST2H2AA3///HIST2H2AA4 HIST2H2BE HIVEP2 HLA-A HLA-A///HLA-F///HLA-J HLA-B HLA-C HLA-DQA1 HLA-DQB1 HLA-DQB2 HLA-E HLA-F HLA-G HLTF HMGB1 HMGB3 HMGCR HMGCS1 HMHA1 HNRNPA2B1 HNRNPF HNRNPM HNRPDL HOPX HOXB6 HOXC8 HP HP///HPR HPGD HPGDS HPSE HS2ST1 HS3ST1 HS3ST2 HSD11B1 HSD17B11 HSDL2 HSP90AB1 HSPA1A///HSPA1B HSPA8 HTR1B HUS1 ICAM1 ICAM3 ID1 IDH2 IDO1 IER2 IER3 IFITM1 IFITM2 IFITM3 IFNA14 IFNAR2 IFNG IFRD1 IGF1R IGF2BP3 IGFBP3 IGFBP7 IGHA1///IGHA2///LOC100126583 IGHG1///IGHG2///IGHM///IGHV4-31 IGK@///IGKC IGK@///IGKC///IGKV1-5 IGL@ IGLC7///IGLV1-44 IGLC7///IGLV1-44///LOC100290481 IGLL3P IGLV1-44///LOC100290481 IL11RA IL18R1 IL18RAP IL1A IL1B IL1R1 IL1R2 IL1RN IL2RB IL32 IL6R IL6ST IL7R IL8 IMPACT INHBA INPP1 INPP5A INPP5K IPO5 IPO7 IQSEC3 IRF1 IRF9 ISG20 ITGB1 ITGB1BP1 ITIH5 ITK ITPR2 ITPR3 ITSN1 IVD JHDM1D JUP KAL1 KCNAB1 KCNB2 KCNJ15 KCNJ2 KCNK1 KCTD12 KDM2A KDM5B KDM6B KIAA0913 KIAA0947 KIAA1598 KIAA1704 KIF16B KIR2DL4 KIR3DL3 KL KLF5 KLF6 KLHDC10 KLHL11 KLHL20 KLHL24 KLRB1 KLRC1///KLRC2 KLRD1 KMO KPNA1 KPNA4 KPNB1 KRAS KRT13 KRT18 KRT19 KRT23 KRT6A KRT6A///KRT6B///KRT6C KRT6B KRT7 KRT8 LACTB2 LAMB1 LAMP3 LAPTM4B LBH LCK LCN2 LGALS2 LGMN LILRA3 LILRB4 LIMK2 LLGL1 LMNB1 LOC100132247///LOC348162///LOC613037///LOC728888///NPIPL3 LOC100507192 LOC100507804///TPSAB1 LOC100507851///PRAMEF1///PRAMEF2 LOC150759 LOC441259///PMS2L2///PMS2P1///PMS2P6 LOC91316 LONRF3 LOX LPCAT1 LPCAT4 LPL LRP10 LRP12 LRRC40 LRRC41 LSAMP LSM3 LSM5 LSP1 LTB LY75 LYN MACF1 MAGED1 MAL MAN1A1 MAP2K3 MAP3K4 MAP4K5 MAP9 MAPK9 MARCKS MARCKSL1 MBOAT7 MBP MCTP2 MED13L MED15 MERTK MGAM MGAT4B MICAL2 MINK1 MINPP1 MIR21///TMEM49 MKI67 MLL MME MMP12 MMP14 MMP7 MMP9 MOBKL2B MOSPD1 MPDU1 MPZL1 MREG MRPS18B MS4A1 MSMB MSR1 MSRA MST1///MST1P2///MST1P9 MT2A MTCP1NB MTF1 MTM1 MTO1 MTUS1 MUC1 MUC13 MUC4 MUC5AC MUC5B MVP MXD4 MYLIP MYLPF MYO1B MYO1D MYST1 MYST3 N4BP2L2 NAA40 NAB1 NADK NBEAL2 NBN NBPF10 NCOA3 NCR3 NDUFB7 NEAT1 NEBL NEU3 NF2 NFAT5 NFE2L2 NFKB2 NFKBIA NID1 NINJ1 NIPBL NKG7 NKTR NKX2-1 NOC3L NPAT NPFF NPIPL3 NQO1 NR3C1 NR4A2 NRBP1 NRG2 NRP1 NRXN2 NSUN3 NUDT4 NUP160 NXF3 OAZ2 OAZ3 OGDH OLFML2B OLR1 OPRM1 ORM1 ORM1///ORM2 OSBPL1A OSM P2RY14 P4HB PABPN1 PALLD PARG PAWR PBX1 PBXIP1 PCBP2 PCMT1 PCOLCE2 PDCD4 PDCL PDE3B PDE4C PDGFD PDHA1 PDIA3 PDIA6 PDP1 PDZK1IP1 PEA15 PECI PELI1 PERP PEX11A PEX19 PEX3 PFAS PFKP PGF PHC2 PHF3 PHIP PHLDA3 PHYH PI3 PIAS1 PIGK PIGR PIK3CB PIK3CD PIM1 PIM2 PITPNA PKD2 PKM2 PKN2 PKNOX1 PLA2G16 PLA2G7 PLAT PLCB4 PLCXD1 PLEK PLK3 PLOD2 PLS1 PLTP PLUNC PLXNC1 PMAIP1 PMS2P1 PMS2P3 POLR1B POLR2K POLRMT POP4 POT1 POU3F1 POU4F2 PPA1 PPARG PPIB PPIF PPP1R10 PPP1R14B PPP1R15A PPP2R1A PPP2R1B PPP2R2A PRDX2 PREPL PRF1 PRKCB PRKCI PROS1 PRR11 PRSS21 PRSS8 PSENEN PSMB8 PSMB9 PSMF1 PSTPIP2 PTGES PTGS2 PTMA PTN PTPLA PTPN22 PTPN4 PTPN7 PTPRF PTPRH PTPRM PTTG1 PVRIG PXN PYY2 R3HDM2 RAB11FIP2 RAB22A RAB25 RAB27A RAB4A///SPHAR RABGGTB RAD51L3 RAF1 RALGDS RAN RAP2C RARRES1 RARRES3 RASAL2 RASGRP1 RASSF2 RBM14///RBM4 RBM5 RBMX2 RBP4 RBPMS RCAN1 RDH14 RECK REEP5 RELA RELB RERE RHBDF2 RHOBTB1 RHOBTB3 RHOC RHOH RHOQ RIOK3 RNASE1 RNF103 RNF115 RNF19B RP2 RPL14 RPL3 RPLP0 RPN2 RPRD1A RPS3 RPS6 RPS9 RRAS2 RRP15 RRS1 RTEL1 RTEL1///TNFRSF6B RTN1 RUNX3 RYBP S100A12 S100A8 S100A9 S100P S1PR4 SAA1///SAA2 SAA4 SAMM50 SATB1 SAV1 SBNO2 SC4MOL SCAMP1 SCARF1 SCD5 SCML1 SDC1 SDF4 SDS SEC14L1 SEC23A SEC61A1 SECISBP2L SECTM1 SEL1L SELL SEMA4D SEMA4G SEPP1 SEPT10 SERF2 SERINC3 SERP1 SERPINB1 SERPINB13 SERPINB3 SERPINB4 SERPINB5 SERPINB9 SERPINI1 SET SETD5 SF1 SF3A3 SFRS18 SFTPA2 SFTPB SGK1 SGPP1 SH2D1A SH3GL3 SH3GLB2 SHQ1 SIDT2 SIK2 SIN3B SIX3 SLAMF1 SLC12A5 SLC16A3 SLC19A1 SLC19A2 SLC19A3 SLC20A1 SLC25A16 SLC25A37 SLC26A2 SLC26A4 SLC34A2 SLC35A2 SLC35E1 SLC35E3 SLC36A1 SLC38A1 SLC39A6 SLC39A8 SLC44A4 SLC46A3 SLC47A1 SLC4A4 SLC5A3 SLC6A14 SLC7A11 SLC7A5 SLC9A8 SLPI SMAD6 SMAD7 SMARCA5 SMCHD1 SMG1 SMPDL3A SNAPC1 SNN SNX24 SOBP SOCS3 SOCS5 SOD2 SON SORL1 SORT1 SOX21 SPARC SPDEF SPG20 SPG21 SPOCK2 SPP1 SPRR1A SPRR1B SPRR3 SPRY1 SPTBN1 SQSTM1 SRBD1 SREBF1 SRPK1 SRRM2 SRSF11 SRSF5 SSRP1 ST13 ST14 ST3GAL6 ST6GAL1 ST8SIA4 STAB1 STAC STAG1 STAT1 STAT3 STAT4 STEAP4 STK17A STK3 STK38 STMN1 STON1 STX12 SUB1 SUPT6H SUV420H1 SVIL SYNE1 SYNE2 SYNGR1 SYNJ2 TACC1 TACSTD2 TAF1 TAP1 TARBP1 TBC1D4 TBC1D5 TBX21 TCEAL4 TCERG1 TCF4 TCF7 TCF7L2 TCN1 TES TEX14 TF TFAM TGDS THBS1 TIMP1 TLR2 TLR7 TLX3 TM4SF1 TMC5 TMEFF1 TMEM110 TMEM144 TMEM149 TMEM176B TMEM212 TMEM30B TMEM38B TMEM70 TMEM97 TMEM9B TMPRSS2 TNFAIP3 TNFAIP6 TNFRSF10C TNFRSF1A TNFRSF1B TNFRSF21 TNFSF10 TNFSF14 TNFSF15 TNIP1 TNIP2 TNNI2 TNPO1 TOM1 TOP1 TP63 TPBG TPCN1 TPD52 TPI1 TPM1 TPPP TPR TPSAB1 TPSAB1///TPSB2 TPSB2 TRA2A TRAC TRAC///TRAJ17///TRAV20 TRAF3IP3 TRAF5 TRAFD1 TRAK1 TRBC1 TRBC1///TRBC2 TRD@ TREML2 TRHDE TRIB1 TRIB2 TRIM14 TRIM31 TRIM36 TSEN2 TSFM TSNAX TSPAN1 TSPAN6 TSPAN8 TSR1 TSTA3 TTC3 TTC33 TTF2 TUG1 TXN TYMP U2AF1 UBA1 UBC UBE2H UBE2I UCP2 UGCG UPF1 UPK1B USP10 VAMP4 VAMP5 VCP VEGFA VGLL1 VPS37B WARS WDR44 WDR74 WFDC2 WNK1 XBP1 XCL1 XCL1///XCL2 XPO6 YES1 YWHAZ ZBED2 ZBED5 ZBTB38 ZC3H11A ZC3H12A ZDHHC18 ZEB1 ZFAND1 ZFP30 ZFP36 ZFP36L1 ZFR ZKSCAN5 ZNF143 ZNF160 ZNF235 ZNF271 ZNF292 ZNF330 ZNF442 ZNF443 ZNF468 ZNF518A ZNF589 ZNF611 ZNF721 ZNF83 | CurrentSmoker_1 BAL_42_A0B0 BAL_45b_A2B0 BAL_20b_A1B1 BAL_41_A1B0 BAL_29_A1B0 BAL_15b_A0B0 BAL_47a_A0B0 BAL_31_A0B1 BAL_47c_A0B1 BAL_4_A0B0 BAL_27a_A0B0 BAL_3_A1B0 BAL_37_A1B1 BAL_15a_A0B1 BAL_18_A1B0 BAL_47b_A0B1 BAL_1_A0B0 BAL_12_A1B0 BAL_25_A0B0 BAL_33_A0B1 BAL_44a_A0B0 BAL_13_A0B0 BAL_45a_A1B0 BAL_43_A0B0 BAL_17_A0B0 BAL_44b_A0B0 BAL_26_A2B0 BAL_40_A0B0 BAL_19_A0B0 BAL_21_A0B0 BAL_46_A2B0 BAL_27b_A2B1 BAL_39_A0B1 |
| **6** | AAAS ABCA1 ABCB9 ACBD3 ACCN3 ACSL3 ACSL4 ACSM2A///ACSM2B ACTL6B ACTR6 ACTR8 ACVR1B ADRA1D ADRA2B AGAP2 AKAP1 AKAP8L AKR1B10 ALG13 ALKBH4 ALPP///ALPPL2 ANK1 ANKRD53 ANP32A///ANP32D AP1S1 APOBEC2 APOBEC3A APOD APTX ARAP2 ARFRP1 ARHGAP26 ARHGAP28 ARHGDIA ARID1A ARID4B ARIH2 ASF1A ASPHD1 ATN1 ATP2A3 ATP4A ATP6V1B1 AVP B4GALT1 BARX1 BAZ1B BCL2 BCL3 BFSP1 BIRC2 BIRC3 BMP8A BMPR1B BNIP3 BOLA2///LOC440354///LOC595101 BRD7P3 BTNL3 BUB3 C14orf138 C15orf29 C16orf42 C17orf53 C18orf1 C19orf26 C19orf73 C1orf144 C1orf156 C22orf31 C3orf14 C3orf64 C4orf29 C6 C7orf64 CACNA1A CACNA1E CACNA1F CALD1 CAMK2B CASKIN2 CBLB CCDC101 CCDC134 CCDC70 CCDC91 CCKAR CCL8 CCR10 CCR2 CD22 CD2AP CD3EAP CD93 CDA CDC25C CDC42EP4 CDC73 CDK13 CDK8 CELP CENPJ CES1P1 CHD9 CHRNE CKS2 CLK4 CNOT2 CNOT3 CNPY2 CNR1 CNTNAP2 COL13A1 COQ10B CPEB3 CPNE6 CRIPT CROCC CRYBB3 CSF1 CSNK1G1 CST1 CSTA CSTF1 CSTF2T CTRC CTSB CXCL12 CYP1A1 CYP1B1 CYR61 DAG1 DBR1 DDX28 DENR DLG4 DLGAP4 DLX4 DNAJB5 DNASE1L2 DNM2 DOCK6 DPY19L4 DST DTX3 DUSP1 DYRK1B EFNB1 EIF4E EIF4E2 EMILIN1 ENPP2 ENTPD1 ENTPD4 EPAS1 EPHB6 EPN1 EPOR ERAP2 ERI2 ETNK1 F12 FAM134A FAS FBXL18 FBXO38 FBXO42 FCAR FCGR2B FGF14 FGF6 FGFR3 FGL2 FHOD3 FKBP3 FKBP8 FMR1 FN1 FOXA2 FOXB1 FOXC2 FOXE1 FOXO3///FOXO3B FRMD4B FRS3 FSTL3 FUBP1 FUS FUT7 FYN GALNT1 GALNT7 GATA1 GBX2 GCH1 GCN1L1 GHRHR GLRA1 GLRA2 GNAS GOLIM4 GORASP2 GP1BB GP2 GPR135 GPR144 GPR68 GPR89A///GPR89B///GPR89C GPRC5B GRAP2 GRIN1 GRLF1 GSTTP1 GTF2F1 GTF2H1 GTF2H2///GTF2H2B///GTF2H2C///GTF2H2D GTPBP4 GUCA2A H2AFX HAB1 HBG1///HBG2 HIC1 HIRIP3 HIST1H1D HIST1H2BI HMGA1 HMGCR HNRNPUL2 HOPX HOXA6 HOXD10 IBTK ICAM3 ICOSLG ID2///ID2B IGF2AS IGFALS IGHA1///IGHA2///IGHD///IGHG1///IGHG3///IGHM///IGHV4-31///IGHV4-59///LOC100126583 IGLV2-18///IGLV3-19///LOC100290481 IL17B IL9R INTS1 INTS6 IQSEC2 IRF5 ITGA4 ITGA7 ITGB4 ITK ITPR1 KCNA6 KCNE1 KCNN3 KCNQ1DN KCNQ4 KCTD17 KDM2A KDM5A KDSR KHSRP KIAA0368 KIAA0776 KIAA1751 KIR2DL1///KIR2DL2///KIR2DL3///KIR2DL5A///KIR2DL5B///KIR2DS1///KIR2DS2///KIR2DS3///KIR2DS4///KIR2DS5///KIR3DP1///LOC727787 KIR2DL2 KIR3DX1 KLF9 KLHL20 KLK14 KLK15 KPNA2 KRAS KRIT1 KRT13 KRT6A KRT9 LARGE LBR LCK LECT1 LETM1 LFNG LHX1 LHX5 LILRA5 LIMD2 LIMK1 LOC100287927 LOC100506517 LOC100507804///TPSAB1 LOC100508624 LOC254896 LOC729991-MEF2B///MEF2B LOC92249 LRCH3 LRIT1 LRRC23 LRRC3 LRRC50 LRRC68 LTBP4 MAG MAP2K3 MAP3K7 MAPK8IP1 MAPK8IP3 MAPRE3 MAPT MAST2 MBP MBTPS1 MDC1 ME1 MED13L MED23 MED6 MED7 MEX3C MFAP4 MFNG MINK1 MIR622 MLL MLL4 MLLT1 MLLT4 MLXIPL MOAP1 MOCS3 MPHOSPH8 MPL MREG MRPL41 MST4 MTA1 MTA2 MTAP MTO1 MUC5B MVK MYBL1 MYBPC2 MYH11 NAA16 NCAM1 NCOR2 NDEL1 NELL2 NEUROG3 NF1 NF2 NFIX NFKBIL2 NIPAL3 NIPSNAP3B NNAT NPPC NR2F6 NRP2 NRXN1 NRXN2 NUP133 ODZ3 OGFR OSBPL10 OXTR P2RY14 PAQR3 PARG PAX8 PCDHB11 PCDHGA10///PCDHGA11///PCDHGA12///PCDHGA3///PCDHGA5///PCDHGA6 PCDHGA3 PCGF2 PCIF1 PDE4A PDE6G PDIA2 PDK2 PDLIM7 PDPN PDSS2 PDXK PEX5 PGAM2 PHF15 PHF20 PHKB PHLDA1 PKD1 PKP3 PLA2G7 PLEC PLUNC PLXNC1 PMS2P4 PNN PNPLA2 POLG POMC PORCN POT1 POU3F1 PPBP PPIL6 PPP1R1A PPP1R2P9 PPP2CB PRF1 PRKCB PRKCG PRKDC PRLH PROS1 PRSS50 PSMC6 PSPC1 PSTPIP2 PTBP2 PTEN PTH2R PTMS PTP4A1 PYGO1 PYROXD1 PYY PYY2 RAB11B RAB27A RAB3D RAB4A///SPHAR RABGAP1L RAD17 RAD51L3 RAI1 RALA RANBP6 RARRES1 RASA3 RASA4///RASA4P RASGRP1 RASGRP2 RBM22 RCHY1 RERE RFTN1 RGS16 RHEB RNF126 ROBO4 RRBP1 RRM1 RRP15 RRP1B RUNX1T1 RYBP SAA1///SAA2 SAE1 SAFB SAR1A SAR1B SCN2A SCN2B SCNN1A SCRT1 SCT SEC14L3 SEC23B SEC24D SELPLG SEMA4G SEMA6A SEPHS2 SEPT9 SERPINB1 SERPINB9 SGCE SGCG SLC12A4 SLC12A9 SLC13A2 SLC16A1 SLC16A3 SLC20A1 SLC22A11 SLC22A6 SLC22A8 SLC39A8 SLC4A7 SLC5A3 SLC7A11 SLC9A3R2 SLIT2 SMAD6 SMARCA4 SMARCD1 SMCHD1 SMURF2 SNRNP70 SOCS5 SOD3 SORBS3 SORL1 SOX14 SOX21 SP2 SPANXA1///SPANXA2///SPANXB1///SPANXB2///SPANXC///SPANXF1 SPAST SPRR1B SRCAP SRP19///ZRSR1 SRPK1 SRPK2 ST6GALNAC4 ST8SIA2 STAG2 STAT1 STK17A STK38L STRN3 STS STX2 SUZ12 TAF11 TALDO1 TAP1 TAX1BP3 TBPL1 TCF12 TCF15 TET3 TEX13A TIA1 TIAL1 TIGD1L TIMM17A TLE4 TLX3 TM2D1 TMEM45A TNFSF10 TNK2 TNPO2 TNS1 TP53 TPPP TPSAB1 TRA2A TRAF5 TRIM62 TRPV4 TSN TSSK1B TTC37 TTC38 TTC39A TTLL3 TUBB2A///TUBB2B TUBGCP2 TXN TYRO3 UBAP2 UCP3 USP2 USP4 USP8 VAV2 VCX2 VDAC1 VIL1 VPS13A WBP4 WDR44 WDR55 WIPI1 WNT5B WNT6 WSB2 XDH YARS2 YBX1///YBX1P2 YTHDC2 ZBTB10 ZBTB16 ZBTB17 ZBTB7B ZCCHC10 ZEB1 ZER1 ZFP36L1 ZNF587 ZNF623 ZNF646 ZNF673 ZNF702P | CurrentSmoker_67 CurrentSmoker_81 BAL_46_A2B0 FormerSmoker_72 BAL_47c_A0B1 BAL_29_A1B0 BAL_47a_A0B0 BAL_27a_A0B0 BAL_1_A0B0 BAL_12_A1B0 BAL_42_A0B0 BAL_37_A1B1 BAL_20b_A1B1 BAL_25_A0B0 BAL_33_A0B1 BAL_43_A0B0 BAL_44b_A0B0 BAL_27b_A2B1 BAL_47b_A0B1 BAL_45a_A1B0 BAL_18_A1B0 BAL_15a_A0B1 BAL_13_A0B0 BAL_3_A1B0 BAL_4_A0B0 BAL_40_A0B0 BAL_39_A0B1 BAL_45b_A2B0 BAL_17_A0B0 BAL_26_A2B0 |
| **7** | ABAT ABCA1 ABCG4 ABHD2 ACHE ACP5 ACP6 ACRV1 ADA ADAM19 ADAMTS5 ADM ADORA3 ADRB3 AGR2 AHCYL1 AKT2 ALDH1A1 ALOX5 AMPH ANKRD1 ANKRD11 ANKRD36B ANKRD36BP2 ANO3 ANXA6 ANXA7 AP1S2 APOBEC3A APOBEC3F///APOBEC3G APOBEC3G APOE AQP9 ARHGEF12 ARHGEF40 ARL1 ARL4C ASPA ASXL1 ATP1B1 ATP5G2 ATP6V0E2 ATP8A1 AURKA AVIL B4GALT6 BACE2 BACH2 BANF1 BARX1 BAT2L2 BAZ2B BCAT2 BCL2 BCL2A1 BCL6 BCL7A BEX1 BIRC3 BLMH BLZF1 BRD4 BRE BRSK2 BTF3 BTF3P11 BTN3A1 BTNL8 BTRC C10orf68 C11orf57 C14orf139 C14orf156 C15orf63///SERF2 C17orf101 C19orf40 C1orf38 C1QB C20orf103 C21orf59 C21orf7 C2orf43 C2orf47 C5orf4 C6orf25 C6orf64 CALCRL CALM1 CAMK1 CASK CASP7 CASQ2 CCL18 CCL2 CCL3///CCL3L1///CCL3L3 CCL4 CCL5 CCL8 CCND2 CCR2 CCR5 CCR7 CD14 CD163 CD2 CD247 CD300A CD34 CD36 CD3E CD3EAP CD40 CD6 CD69 CD83 CD8A CD9 CD93 CD96 CDC14A CDC14B CDC42 CDC42BPA CDH5 CDH6 CDK11A///CDK11B CDKN1A CDR1 CEACAM8 CEP350 CEP57 CH25H CHD9 CHIT1 CHN2 CHST15 CKMT2 CLC CLEC4A CLEC4E CLTA CMKLR1 CNOT4 CNR1 CNTLN COBLL1 COMMD3 CORO1A COX11 COX4I1 CPS1 CPSF6 CPT1A CR1 CREM CRHR1 CSF3R CSGALNACT1 CSH1///CSH2///CSHL1///GH1///GH2 CSNK1A1 CTNNA1 CTSE CTSZ CTTN CXCL10 CXCL13 CXCL14 CXCL9 CXCR1 CXCR4 CXCR6 CYBB CYLC1 CYLD CYP1A1 CYP2B6 DCP2 DDX28 DDX3Y DDX51 DENND1C DHODH DHRS2 DIABLO DLAT DNAH3 DNAJC3 DOCK4 DOCK5 DOPEY1///LOC100509911 DPP3 DSE DYNC1LI2 EDDM3A EDNRB EGR3 EHD1 EID1 EIF1AY EIF2AK1 EIF2C1 EIF2C2 EIF3A EIF5 EMP3 EMR2 ENDOU ENPP2 ENTPD1 ENTPD4 EP400 EPB41L5 ERAP2 ERGIC2 ESPL1 EVI2A EVX1 EXOC6B EXOG F13A1 F13B F7 FABP3 FABP4 FABP7 FAIM FAIM3 FAM118A FAM134A FAM155A FAM164A FAM48A FAM49A FAM89B FANCG FAS FBN1 FCER1G FCGR1B FCGR2B FCGR2C FCGR3A///FCGR3B FCN1 FFAR2 FFAR3 FGF7 FGL2 FGR FICD FLJ11292 FN1 FOLR3 FOSL2 FOXM1 FPGS FPR1 FRZB FTO FYB FYCO1 FYN G0S2 GADD45B GALNT6 GALR3 GCH1 GGTLC1 GHRHR GIMAP5 GIMAP6 GLO1 GLP1R GM2A GNAS GNG12 GNL3 GNLY GOLGA8A///GOLGA8B GPR109B GPR171 GPR18 GPR97 GPRASP1 GREM2 GRIA2 GRIA4 GRIK1 GSTM4 GSTT1 GUCA2B GZMA GZMB H2AFX HBA1///HBA2 HBB HCG4P6 HCK HDAC2 HEATR6 HEXIM1 HIST1H2BH HIST1H2BI HIST1H4E HMG20B HNRNPD HNRNPF HOPX HOXC10 HOXD1 HSP90AA1 HSPA2 HSPA6 HSPE1 HSPH1 HTN1 ICAM1 ICAM3 IDH3G IER3 IFITM1 IFNA16 IGHA1 IGHA1///IGHG1///IGHG2///IGHG3///IGHM///LOC100126583///LOC100290036 IGHG1 IGHM IKBKAP IKZF2 IL10RA IL18RAP IL1B IL1R2 IL21 IL21R IL2RA IL2RB IL32 IL6R IL8 INPP1 INPP5D IRF4 IRGC ITGA4 ITK ITM2A ITSN1 IVNS1ABP KCNJ15 KCNJ2 KCNMB3 KDM4B KIAA0368 KIF15 KIF5B KIR2DL4///KIR2DL5A///LOC100287534 KIR2DL5A KLF12 KLHL22 KLRB1 KLRC1///KLRC2 KLRK1 KPNA3 KRT18 KRT19 KYNU L1CAM L1TD1 LAG3 LAMB1 LAPTM5 LARGE LAT///SPNS1 LCK LCN1 LCN2 LCP2 LECT2 LGALS1 LGMN LILRB4 LMAN2 LMNA LMNB1 LOC100127972 LOC100287515///ZNF26 LOC100507328 LOC100507328///LOC100508591 LOC283079 LOC389906 LPL LRRC32 LSM14A LTB LUC7L3 LUZP2 MAGEA4 MAGEA9///MAGEA9B MAP7D3 MARCKS MARCO MBD2 MC3R MCM10 MCM2 MCOLN1 MDC1 MDM4 MECP2 MEF2C METTL1 METTL2B MGAM MGAT2 MGST2 MID1 MIR1244-1///MIR1244-2///MIR1244-3///PTMA///PTMAP5 MMP12 MOS MOXD1 MPHOSPH10 MPL MRC1///MRC1L1 MREG MRPL17 MRPL18 MRPS18B MRPS31 MS4A1 MS4A4A MS4A6A MSI1 MSMB MSN MTX2 MUC16 MUC5AC MUM1 MYCL1 MYH3 MYO1F MYO6 N4BP1 NAA35 NARFL NCAM1 NCAN NCF1C NCF4 NCLN NEIL3 NEK1 NF2 NFYB NINJ1 NIPSNAP3B NKG7 NKTR NLGN1 NLRP3 NMBR NMT2 NOS1 NOX1 NPRL3 NPY1R NR0B1 NR1H3 NR2F6 NR5A2 NRF1 NRG1 NTRK3 NUBPL OAZ2 OBSL1 OGG1 OLFML2B OR12D3 OTOF OTUD7B P2RY13 P2RY14 PAPPA2 PAPSS2 PBRM1 PCBP2 PCSK6 PCYOX1 PDCD6 PDE4A PDE4B PDIA4 PDLIM1 PDX1 PFKFB3 PGLYRP4 PGR PHF15 PHF16 PHF8 PHLDA1 PI3 PIGL PIK3CD PIM1 PIP4K2B PIP5K1B PLA2G7 PLAUR PLCXD1 PLEKHG6 PLIN1 PLIN2 PLK3 PLSCR3 PLTP PLXNC1 PNPLA6 POLR2D POLR3G POM121L9P POMGNT1 PPIB PPIF PRDX4 PRF1 PRKCB PRKCQ PRKDC PRKX///PRKY PRR3 PRUNE PSD3 PSG9 PSTPIP1 PSTPIP2 PTGER2 PTMA PTPN7 PTPRC PTPRE PVRIG RAB11B RABEP2 RANBP1 RAP2C RASA4 RASGRP1 RASSF2 RBBP6 RBM4B RBM5 RC3H2 RCC1 RCN2 RECK REL REXO2 RFC5 RFTN1 RIC3 RNF216 RNF24 RNF43 RNF8 RPS2P45 RPS4Y1 RRAGA RSAD2 RUNX1 RUNX3 RXRB S100A12 S100G SAMSN1 SAP18 SART3 SCAMP1 SCGB1D1 SCN1A SCN3B SCNN1A SDHD SEC14L1 SEC14L3 SEC23B SEC31A SELL SEMA4D SEMA4F SEPHS1 SEPT6 SERINC3 SERPINB9 SFTPA2 SFTPB SFTPC SH2D1A SH2D2A SH3BP4 SIGLEC6 SIK3 SIRT3 SLAMF1 SLC12A3 SLC17A3 SLC17A6 SLC1A1 SLC20A1 SLC22A2 SLC23A2 SLC24A3 SLC25A15 SLC25A36 SLC25A37 SLC30A4 SLC34A1 SLC37A1 SLC38A7 SLC4A4 SLC7A11 SLC7A5 SLC7A6 SLCO1B3 SMG6 SNCG SNN SNRPG SOCS1 SOD2 SOHLH2 SOX2 SOX4 SP100 SPARCL1 SPATS2L SPINLW1 SPOCK2 SPP1 SPRY1 SRSF2IP SRSF6 SSB SSX2///SSX2B SSX3 SSX4///SSX4B ST6GALNAC5 STAT5A STAT5B STYK1 SVEP1 SYT1 TAF6L TAP1 TARP///TRGC2 TAS2R16 TBRG4 TBX1 TCF25 TCTN3 TECPR2 TESK2 TFAP2A TFEB TFF3 TFRC TGFB1I1 TGFB2 TH1L THRAP3 THSD7A TIMM17A TIMM23 TIMP3 TLE4 TLR2 TLX2 TMEM140 TMEM158 TMEM204 TMEM209 TNF TNFAIP6 TNFRSF1B TNFSF4 TNFSF8 TNIP1 TNS1 TP63 TPD52L2 TPP1 TPPP TPPP3 TPR TRAC TRAC///TRAJ17///TRAV20 TRAF1 TRAF3IP3 TRAF5 TRAT1 TRBC1 TRBC1///TRBC2 TRD@ TRHR TRIM14 TRIM16 TRIM2 TRIM29 TRNAU1AP TRPC6 TRPV4 TSPAN3 TSPYL4 TSR1 TTC3 TTR TUBA3D TUBGCP5 TXNL4A UBE2D1 UCHL1 UGCG UGT8 UHRF1BP1L UPP1 USO1 USP20 USP27X USP4 VAMP1 VARS VASH2 VCAN VDAC3 VEGFA VGLL1 VPREB3 VPS72 VSIG10 WBP2 WDHD1 WFDC8 WIPF1 WIPF2 XIAP XIST XYLT1 YIPF6 YWHAE YY1 ZBED2 ZBTB44 ZC3H15 ZC3HAV1 ZDHHC17 ZEB1 ZER1 ZFAND5 ZFHX3 ZMYND11 ZMYND8 ZNF137P ZNF155 ZNF239 ZNF248 ZNF267 ZNF281 ZNF529 ZNF702P ZNF79 | FormerSmoker_69 NeverSmoker_3 BAL_44a_A0B0 BAL_43_A0B0 BAL_39_A0B1 CurrentSmoker_1 BAL_19_A0B0 BAL_27a_A0B0 BAL_1_A0B0 BAL_47a_A0B0 BAL_3_A1B0 BAL_31_A0B1 BAL_33_A0B1 BAL_47c_A0B1 BAL_25_A0B0 BAL_41_A1B0 BAL_15b_A0B0 BAL_47b_A0B1 BAL_18_A1B0 BAL_15a_A0B1 BAL_37_A1B1 BAL_17_A0B0 BAL_29_A1B0 BAL_4_A0B0 BAL_44b_A0B0 BAL_46_A2B0 BAL_20b_A1B1 BAL_40_A0B0 BAL_12_A1B0 BAL_42_A0B0 BAL_21_A0B0 BAL_45a_A1B0 BAL_13_A0B0 BAL_27b_A2B1 BAL_26_A2B0 BAL_45b_A2B0 BAL_20a_A1B1 |
| **8** | AAK1 ABHD2 ADD3 ADORA2A///SPECC1L AKAP1 AKR1B10 AKR1C3 ALCAM ANKRD11 ANKRD12 ANKRD36B ANO3 AP1S1 APP APPL2 ARHGEF12 ARPC2 ATP2A2 ATRX AVIL AZGP1P1 BAT2L2 BCL6 BCLAF1 BMPR1B C11orf10 C11orf24 C14orf106 C17orf101 C19orf60 C1orf56 C20orf117 C22orf28 C2orf24 CALM1 CALR CAPZB CCT2 CDC42BPA CDK7 CEP290 CLCN3 CNDP2 COIL COPB1 COPZ1 COX15 COX5A COX7A2 CSDA CSDE1 CSK CSNK1A1 CSNK1G2 CSTA CTBP2 CTNS CTSB CX3CR1 CYP1A1 CYP1B1 CYP2B6 DAXX DCTN2 DDOST DDX17 DDX24 DDX3Y DEF6 DEFB1 DNAJC16 DNAJC8 DPY19L1 DTX4 DYRK2 EBAG9 EID1 EIF3A ELK1 ELN EPOR ERLIN2 ESF1 EZR FAM134C FBXO9 FDFT1 FGF20 FGFR2 FGFR3 FIP1L1 FLRT3 FLT3LG FTSJ2 FUS GAK GALK2 GDF1///LASS1 GDI1 GOLGA2 GORASP2 GPATCH3 GRIN1 GYG2 H3F3B HIST1H2BK HLF HNF4A HNRNPA3 HNRNPM HOMER3 HSP90AA1 HSPA1A HSPH1 IDS IL1F6 IL7 IL8 ILF3 IQCK IREB2 ITPR1 JAG1 KCNJ5 KCNK13 KCTD12 KDM6B KIAA0562 KIAA1033 KLF6 KLHDC10 KLHL1 KPTN KRT86///LOC100509764 LIMK2 LOC100288142///NBPF1///NBPF10 LONP2 LRRC37A///LRRC37A2///LRRC37A3///LRRC37A4 LRRFIP1 LUC7L3 MANF MAP9 MARCKS MED25 METTL1 MPDZ MUC8 MYH10 MYH14 MYL12B MYO6 MYST4 NAA15 NBPF1///NBPF10///NBPF11///NBPF12///NBPF14///NBPF15///NBPF16///NBPF24///NBPF8 NBPF10 NEK1 NF1 NFAT5 NKTR NOL12///TRIOBP NOP2 NPEPPS NPM1 NUBPL OAZ2 P2RY14 PABPC1 PACSIN2 PALLD PARK7 PDAP1 PDCD11 PDIA4 PGRMC1 PHKA2 PIK3CD PIK3R1 PKD1 PLCB4 PLOD1 PMS2P3 PPP4R2 PRDM12 PRDX4 PRICKLE4///TOMM6 PRKCI PRRC1 PSMD7 PTAFR PTPN18 PTPN2 PUM1 RAB13 RAB2A RAB9BP1 RABEP2 RALGDS RARRES1 RBM25 RBM5 REST RHEB RNASE4 RNF5 RNPEPL1 RPS6 RRBP1 RSRC2 RUNX1T1 RYBP SART3 SEC14L3 SECISBP2L SERINC3 SERPINB1 SF3B1 SF3B5 SFRS15 SFRS18 SFTPB SLC22A7 SLC25A36 SMARCA2 SMARCA4 SMC3 SMG1 SNAPC1 SNCA SNRPN///SNURF SOD2 SON SPCS3 SPEN SPTBN1 SRRM2 SRSF2IP SSRP1 STK24 STK3 SUCLG2 SUMO2 SUPT6H SYCP2 TACR2 TAGLN2 TALDO1 TAX1BP3 TBL1X TCF15 TCF3 TCF7L2 TES TET3 TFAM TFDP1 TFE3 TFIP11 TGFBRAP1 TH1L THAP4 THOC2 TM9SF3 TMED9 TMEM5 TMEM66 TOP1 TPR TRA2A TRAF3IP1 TSPAN31 TTC3 UBAP2 UBXN4 UGCG ULK2 UQCR11 USP1 USP9Y VPS37C WARS WASL WSCD1 YWHAE YY1 ZC3H11A ZDHHC11 ZMYND8 ZNF292 ZNF410 ZNF528 ZNF75D ZSCAN18 | BAL_27b_A2B1 BAL_21_A0B0 BAL_20a_A1B1 BAL_46_A2B0 CurrentSmoker_10 CurrentSmoker_12 CurrentSmoker_2 FormerSmoker_41 CurrentSmoker_36 FormerSmoker_18 CurrentSmoker_29 FormerSmoker_27 Sample_43_never_smoker Sample_46_never_smoker CurrentSmoker_37 CurrentSmoker_54 NeverSmoker_3 FormerSmoker_28 CurrentSmoker_39 FormerSmoker_34 CurrentSmoker_1 Sample_44_never_smoker Sample_48_never_smoker NeverSmoker_42 NeverSmoker_52 Sample_47_never_smoker NeverSmoker_38 Sample_40_former_smoker_without_cancer NeverSmoker_45 |
| **9** | ABCA7 ACADVL ADRM1 ALDOA ANPEP AP2A2 APRT ARHGDIA ARL6IP4 ATP5D C9orf16 CALM1 CCDC85B CCL18 CCT3 CD151 CDK5RAP3 CLPP CLTB CORO1B COX6B1 CPSF1 CRIP1 DDRGK1 EDF1 EEF1D EHBP1L1 EIF3G EIF6 FAM120A FAU FOLR1 FUS GADD45GIP1 GBAP1 GDF15 GMPPA GPR172A GSS GSTK1 GUK1 HGS IRF3 KIAA0415 KRT13 LGALS3BP LMNA LOC100507328 MAP2K2 MBD3 MED16 MFSD10 MRPS2 MVP NADSYN1 NDUFA13 NDUFS8 NDUFV1 NOP56 NSUN5 NUCB1 NUPR1 P4HB PFKL PGLS POLR2L PPIB PPP1R14B PRKAR1A PSMB6 PSMD2 PUF60 RABAC1 RFXANK RPN2 RPS11 SCO2 SDF2L1 SEC24C SEC61A1 SERPINB6 SPP1 SPRR3 SRRT SUGP1 SULT1A3///SULT1A4 TBCD TCEB2 TCF25 TCIRG1 TEX264 TFPT TIMM13 TIMP1 TMEM160 TMEM222 TOM1 TPRA1 TPSAB1 TPSB2 TYMP UBA1 UBA7 VPS28 VPS37B YWHAE ZGPAT | CurrentSmoker_109 CurrentSmoker_111 CurrentSmoker_98 CurrentSmoker_102 BAL_47b_A0B1 FormerSmoker_61 Sample_47_never_smoker NeverSmoker_3 NeverSmoker_45 BAL_18_A1B0 CurrentSmoker_115 BAL_43_A0B0 BAL_42_A0B0 FormerSmoker_104 FormerSmoker_110 BAL_20b_A1B1 CurrentSmoker_2 BAL_40_A0B0 Sample_40_former_smoker_without_cancer BAL_37_A1B1 NeverSmoker_42 BAL_13_A0B0 Sample_46_never_smoker BAL_45a_A1B0 BAL_33_A0B1 BAL_45b_A2B0 BAL_44a_A0B0 NeverSmoker_100 BAL_29_A1B0 BAL_25_A0B0 BAL_26_A2B0 BAL_4_A0B0 CurrentSmoker_10 BAL_12_A1B0 BAL_47a_A0B0 NeverSmoker_105 CurrentSmoker_39 Sample_48_never_smoker FormerSmoker_28 CurrentSmoker_37 BAL_47c_A0B1 NeverSmoker_38 NeverSmoker_103 FormerSmoker_34 BAL_27a_A0B0 FormerSmoker_27 BAL_44b_A0B0 BAL_3_A1B0 BAL_15b_A0B0 CurrentSmoker_36 Sample_44_never_smoker CurrentSmoker_12 FormerSmoker_41 BAL_17_A0B0 BAL_41_A1B0 BAL_1_A0B0 CurrentSmoker_29 BAL_31_A0B1 FormerSmoker_18 BAL_15a_A0B1 NeverSmoker_52 Sample_43_never_smoker CurrentSmoker_54 |
| **10** | ACO2 ACSF2 ADH7 AHSA1 AKR1B10 AKR1C1 AKR1C2 AKR1C3 ALDH1A1 ALDH1A2 ANKRD11 ANKRD12 ANXA1 ANXA2 ANXA7 APOO APP AQP3 ASPH ATP1B1 ATP2A2 AZIN1 BAT2L2 BBX BTF3 BUB1 C14orf1 C16orf7 C18orf10 C20orf117 CALM1 CBR1 CCNA2 CCT2 CD59 CDC16 CDC42BPA CDH1 CEACAM5 CEP350 CLC CLDN10 CLINT1 CMAH COPB1 COPB2 CTGF CTNNA1 CYB5A CYP1A1 CYP1B1 DAD1 DDX24 DMD DNAJC10 DNPEP DSTN DYNLL1 DYNLRB1 EHD1 EID1 EIF5A ERH ESF1 EZR FAM129A FDFT1 GAPDH GCLC GCLM GOLGA8H GRM1 H2BFS HAX1 HBXIP HIST1H2BE HMG20B HNRNPA2B1 HSP90AB1 HSPA1A///HSPA1B HSPA8 IMPA2 KCNJ15 KDELR2 KDM6B KIFAP3 LSM4 LUC7L3 MAB21L1 MBD4 MBD5 MBTPS1 MEAF6 MED13L MMADHC MPDU1 MRPL18 MSMB MTDH MTUS1 MUC5AC MYCBP NDRG2 NDUFB4 NEK1 NET1 NFAT5 NFKBIA NKTR NPM1 NQO1 NR0B1 NUP62 OAT P4HB PCBP2 PCM1 PDIA3 PERP PGRMC1 PHTF1 PIBF1 PIGR POU4F2 PPIG PPP2CB PQLC1 PRDX1 PRKAR1A PRPF8 PRUNE2 PSENEN PSMC3 PSMD4 RAB11A RNASE4 RRBP1 SAP18 SCD5 SDHC SEL1L3 SERF2 SKP1 SLC25A36 SLC25A37 SLC30A10 SMARCA4 SNRPN///SNURF SOD1 SOD2 SPTBN1 SRRM2 SRSF2IP STC1 TAAR3 TBL1X TCN1 TM9SF1 TM9SF2 TMBIM6 TMCO1 TMED10 TMEM14A TMEM183A///TMEM183B TNFAIP6 TNFRSF21 TNPO1 TPD52 TPR TRIM16 TSPAN3 TTF1 TWF1 TXN TXNRD1 UCHL1 UCP2 UPK1B UTP18 VARS VCP VDAC3 WNK1 YWHAE ZNF148 ZNF286A ZNF292 | BAL_31_A0B1 BAL_1_A0B0 FormerSmoker_27 CurrentSmoker_98 BAL_3_A1B0 BAL_45a_A1B0 NeverSmoker_52 BAL_27a_A0B0 NeverSmoker_100 BAL_45b_A2B0 FormerSmoker_87 CurrentSmoker_115 Sample_43_never_smoker BAL_44b_A0B0 BAL_12_A1B0 BAL_13_A0B0 BAL_25_A0B0 FormerSmoker_104 BAL_26_A2B0 BAL_15b_A0B0 BAL_37_A1B1 BAL_17_A0B0 BAL_41_A1B0 BAL_33_A0B1 FormerSmoker_69 FormerSmoker_84 BAL_43_A0B0 BAL_39_A0B1 BAL_15a_A0B1 NeverSmoker_93 BAL_40_A0B0 BAL_4_A0B0 NeverSmoker_105 BAL_47c_A0B1 CurrentSmoker_29 BAL_44a_A0B0 BAL_47b_A0B1 BAL_20b_A1B1 BAL_47a_A0B0 CurrentSmoker_37 BAL_42_A0B0 BAL_18_A1B0 BAL_29_A1B0 CurrentSmoker_54 NeverSmoker_103 BAL_27b_A2B1 BAL_21_A0B0 BAL_19_A0B0 BAL_46_A2B0 BAL_20a_A1B1 |

*BAL- Human Lung transplant. The numeric part of the sample name is an arbitrary identifier for individual patients. Bronchoalveolar lavage samples obtained from lung transplant recipients whose biopsies had a perivascular score (A) of between 0 and 2, and a bronchiolar score (B) of between 0 and 1. A combined A and B score of 2 or more represents an acute rejection [46].

**Lung epithelial cell transcriptome study of 34 current smokers, 18 former smokers, and 23 subjects who had never smoked [47].

**Table S4C: Membership of *Twenty* Biclusters Learned Using Factor Analysis for Bicluster Acquisition (FABIA)**

| **BICLUSTER NUMBER** | **BICLUSTER GENES** | **BICLUSTER PHENOTYPES**  *BAL or **Smoking Status |
| --- | --- | --- |
| **1** | AAGAB AAK1 ABCC1 ABCC3 ABCC6 ABCC9 ABCD1 ABI2 ABI3BP ABL2 ABO ABT1 ACACB ACP2 ACRV1 ACSBG1 ACSF2 ACSL6 ACSM3 ACTR5 ACVR1B ACYP1 ADA ADAM12 ADAM18 ADAM19 ADAM22 ADAM23 ADAM7 ADAMTS5 ADAMTS8 ADAMTS9 ADAMTSL2 ADARB1 ADAT1 ADCY1 ADCY10 ADCY9 ADD3 ADH1B ADH6 ADH7 ADI1 ADIPOQ ADIPOR1 ADRA1A AFF3 AFTPH AGA AGXT AHCYL1 AHNAK AICDA AK2 AK5 AKAP11 AKAP6 AKAP9 AKR1B10 AKR1C1 AKR1C2 AKR1C4 AKR7A3 AKT2 ALDH3A1 ALPP AMACR AMFR AMOT AMPH ANGPT1 ANGPTL3 ANK2 ANKRD12 ANKRD26 ANKRD34C ANKRD36BP2 ANKRD40 ANKRD55 ANKRD6 ANKRD7 ANP32B ANXA2 ANXA2P2 ANXA3 AOX1 AP2A2 AP2S1 AP3D1 AP3S2 AP4E1 APAF1 APBA1 APBB2 API5 APOBEC3G APOM APOO APOOL APP APRT AQP4 ARAF ARAP2 AREG ARGLU1 ARHGAP10 ARHGAP24 ARHGAP32 ARHGEF12 ARHGEF26 ARHGEF9 ARID3A ARID5B ARIH2 ARL1 ARL4C ARPC2 ARPC4 ARPP19 ART3 ASAH1 ASB13 ASB4 ASB6 ASB8 ASCL3 ASMTL ASPH ASTN1 ATF3 ATG3 ATG4B ATG5 ATM ATMIN ATP11B ATP13A2 ATP1A2 ATP1B1 ATP2B3 ATP5D ATP5J ATP5L ATP5S ATP6V0E1 ATP6V0E2 ATP8A2 ATP8B1 ATRNL1 ATXN2L ATXN3 ATXN3L AURKC AVEN AZIN1 B3GALT1 B3GALT2 B4GALT3 BACE2 BAGE BAI3 BAK1 BASP1 BAT2L2 BAZ2A BBS7 BBS9 BBX BCAS2 BCAT1 BCHE BCKDHB BCL6 BEAN1 BEGAIN BICC1 BICD2 BIN1 BMI1 BMP2K BMP5 BMPR1A BNIP3 BPY2 BRCA1 BRCA2 BRD4 BRD8 BRDT BRPF1 BSCL2 BST2 BTAF1 BTBD18 BTBD3 BTD BTF3 BTF3P11 BTG3 BTG4 BTN1A1 BTN3A2///BTN3A3 BUB1 C10orf110 C10orf12 C10orf137 C10orf18 C10orf68 C10orf84 C11orf1 C11orf17 C11orf30 C11orf63 C11orf67 C11orf71 C12orf43 C13orf27 C14orf105 C14orf132 C14orf139 C14orf156 C15orf39 C15orf63///SERF2 C16orf62 C17orf101 C17orf80 C18orf1 C19orf28 C19orf29 C19orf50 C19orf6 C19orf60 C19orf66 C1GALT1 C1orf129 C1orf156 C1orf63 C1QBP C1QL1 C2 C20orf27 C20orf4 C20orf46 C21orf62 C2CD2L C2CD3 C2orf24 C2orf67 C3orf32 C3orf37 C3orf51 C4orf23 C4orf29 C4orf31 C5orf30 C6 C6orf10 C6orf35 C6orf97 C7orf44 C7orf68 C7orf69 C8orf39 C8orf51 C9orf156 C9orf95 CA5B CABYR CACNA1C CACNB3 CADM3 CALCRL CALD1 CALM1 CAMK1D CAMLG CAMSAP1L1 CAMTA2 CAND1 CAPN10 CAPZB CARD9 CARKD CASC1 CASC5 CASP10 CASP5 CASP7 CBR1 CBR3 CBS CBX4 CC2D1A CCDC101 CCDC130 CCDC132 CCDC15 CCDC48 CCDC76 CCDC85B CCKBR CCL3///CCL3L1///CCL3L3 CCL4 CCL5 CCL8 CCNA2 CCND2 CCNT2 CCR2 CD1E CD22 CD27 CD300C CD4 CD40 CD47 CD58 CD59 CD6 CD7 CD72 CD84 CDA CDADC1 CDC14A CDC14B CDC20 CDC23 CDC25C CDC42 CDC42BPB CDC45 CDC5L CDC73 CDCA4 CDH17 CDH6 CDH9 CDHR5 CDK11A CDK17 CDK19 CDK2 CDK20 CDK2AP2 CDKN1A CDKN1C CDO1 CDRT1 CDSN CEACAM7 CEBPZ CELF3 CENPA CENPB CENPE CEP110 CEP135 CEP192 CEP70 CEPT1 CERK CES1 CGGBP1 CGREF1 CHD2 CHD5 CHD9 CHEK1 CHERP CHI3L1 CHIT1 CHKB-CPT1B///CPT1B CHMP6 CHN1 CHRFAM7A///CHRNA7 CHRNA10 CHRNA4 CHST12 CHST7 CIAO1 CIAPIN1 CILP CIR1 CITED1 CKS1B CKS2 CLCA1 CLCA2 CLCN5 CLCNKB CLDN1 CLEC4E CLEC4M CLIP3 CLPB CLPTM1 CLSTN2 CLTA CMAH CNGA1 CNGB1 CNGB3 CNNM1 CNOT2 CNOT7 CNTF///ZFP91///ZFP91-CNTF CNTN1 CNTN6 COBLL1 COG8///PDF COL10A1 COL13A1 COL14A1 COL4A3 COL5A3 COPB1 COPS8 CORO2B COX15 COX5A COX6A2 COX8A CPA4 CPB1 CPB2 CPEB3 CPS1 CPSF1 CPSF6 CPT1A CRAT CRCT1 CREBZF CRHR1 CRKL CROCCP3 CRP CRYBB2 CSDE1 CSE1L CSF3R CSGALNACT1 CSH1 CSHL1 CSK CSN1S1 CSNK1A1 CSNK1G2 CSPP1 CSTA CSTF1 CSTF2T CTAGE1 CTAGE9 CTBP1 CTDNEP1 CTDSPL CTNNA1 CTNNAL1 CTNNB1 CTRL CTSB CTSS CUX1 CUZD1 CXCL13 CXCL9 CXCR1 CXCR2 CXorf27 CXorf57 CYB561D2 CYB5R2 CYBB CYC1 CYFIP2 CYLC2 CYLD CYP1A1 CYP1A2 CYP1B1 CYP24A1 CYP26B1 CYP2A7 CYP2C9 CYP2E1 CYP2U1 CYP3A7 CYP4F2 CYP7A1 CYR61 D4S234E DALRD3 DARS DAZ1///DAZ2///DAZ3///DAZ4 DBR1 DBT DCAF13 DCAF7 DCC DCLK1 DCN DCT DCTN2 DCX DDB2 DDOST DDT DDX19A DDX24 DDX28 DDX3Y DDX58 DEFB1 DENND1C DENR DGAT1 DGCR2 DGKA DGKE DGKI DGKZ DHDDS DHRS4///DHRS4L2 DHRS7B DHX57 DIAPH2 DIDO1 DIRAS2 DIRAS3 DIS3 DKC1 DKFZP434C153 DKFZp686O1327 DKK2 DLC1 DLEU1 DLG1 DLG2 DLGAP2 DLGAP4 DLK1 DMC1 DMD DNAH3 DNAH6 DNAJA3 DNAJC16 DNAJC17 DNAJC28 DNASE1 DNASE2 DND1 DNMT3B DNTTIP2 DOCK3 DOK1 DOM3Z DOPEY1 DPF2 DPH1///OVCA2 DPH2 DPP4 DPP8 DPY19L1 DRD2 DRD3 DRP2 DSC1 DSCC1 DSCR3 DSCR4 DSG1 DSG3 DSTNP2 DTNA DUS1L DUSP10 DUT DYNC1LI2 DYNC2H1 DYNC2LI1 DYRK3 DZIP3 E2F6 EBAG9 EDA EDNRB EED EEF2 EFCAB6 EFHC2 EFNB3 EGLN3 EIF1AX EIF1AY EIF2B2 EIF2S1 EIF2S3 EIF3B EIF3K EIF3M EIF4B EIF4ENIF1 EIF4G1 EIF4G3 EIF5A EIF5B ELAVL2 ELF1 ELOVL2 ELP3 ELSPBP1 EMCN EML3 EMR3 EN1 ENO1 ENOPH1 ENPP1 ENSA EP300 EPAG EPAS1 EPB41 EPB41L1 EPB41L5 EPHA1 EPHA3 EPHB1 EPM2A EPOR EPS15L1 EPYC ERAP1 ERBB2 ERBB4 ERC2 ERCC3 ERCC4 ERLIN2 ESPL1 ESR1 ESR2 ETFA ETV1 ETV2 EWSR1 EXOG EXOSC2 F9 FABP2 FABP5 FAIM3 FAM118A FAM134B FAM13C FAM155A FAM162A FAM164A FAM168A FAM173A FAM190B FAM198B FAM21A///FAM21B///FAM21C FAM48A FAM5C FAM65B FAM69A FAM8A1 FANCA FANCE FARSA FASTKD2 FAT4 FBLN1 FBN1 FBN2 FBXL18 FBXL5 FBXO28 FBXO40 FBXO42 FBXW11 FCAR FEN1 FER1L4 FETUB FFAR2 FFAR3 FGA FGF1 FGF12 FGF13 FGF22 FGF4 FGF5 FGF7 FGFR1 FGFR2 FHOD3 FLJ11292 FLJ42627 FLOT1 FLRT2 FMO3 FNTA FOLR1 FOXK2 FOXN2 FPR1 FRAT2 FRG1 FRMD1 FRMPD1 FRY FSCN1 FSTL1 FTSJ2 FTSJD2 FUBP1 FYB FZD5 FZD6 FZR1 G0S2 G3BP1 G3BP2 G6PC GABARAPL2 GABARAPL3 GABBR2 GABRA2 GABRA4 GABRA5 GABRB1 GABRB3 GABRR1 GAD2 GALNT1 GALNT2 GALNT6 GALNT8 GAN GAS2 GATA1 GBA///GBAP1 GBF1 GCFC1 GCG GCHFR GCLM GCM2 GCNT2 GFM1 GFRA1 GGCX GGH GHRH GIF GIMAP6 GK3P GLDC GLE1 GLG1 GLRA3 GLRX GLRX3 GLS GM2A GNA14 GNAL GNB2L1 GNB5 GNE GNG11 GNG7 GNGT1 GNL2 GNL3L GNPTAB GNRHR GOLGA4 GOLGA6A GOLGA7 GOLIM4 GON4L GORASP2 GOSR1 GP1BB///SEPT5 GPLD1 GPM6A GPM6B GPN2 GPR116 GPR153 GPR17 GPR19 GPR3 GPR37 GPR44 GPR50 GPR88 GPRASP1 GPRC5B GPSM2 GPX1 GPX3 GPX5 GRAMD1B GRB10 GREB1 GREM1 GREM2 GRIA2 GRID2 GRIK2 GRIK5 GRIN2B GRK4 GRM1 GRM6 GRPEL1 GRSF1 GSPT1 GSPT2 GSTT1 GSTT2 GTF2F1 GTF2H1 GTF3A GTF3C5 GTSE1 GUCY1A2 GUCY1A3 GUCY2C GULP1 GYG2 GYPA GYPB GYS2 GZMB H2AFV H2AFX H2BFS H3F3A///LOC440926 HAB1 HAL HAO1 HAPLN1 HAUS5 HAUS7 HAX1 HBBP1 HBG1///HBG2 HCG4 HCN2 HCP5 HDAC1 HDAC4 HDGFRP3 HDLBP HEATR3 HECW1 HEPH HERC2 HERC4 HERC6 HERPUD1 HEXIM1 HFE HGD HGF HHIPL2 HIF3A HIGD2A HINFP HIST1H2BD HIST1H3J HIST1H4G HIST1H4H HLA-C HLA-DOA HLA-DQA1 HLA-DRB1///HLA-DRB3///HLA-DRB4///HLA-DRB5///LOC100133661///LOC100294036///LOC100509582///LOC100510495///LOC100510519 HLA-DRB4 HMG20A HMGA1 HMGB2 HMGXB4 HN1L HNF1B HNF4A HNRNPA3///HNRNPA3P1 HNRNPD HNRNPH1 HNRPDL HOXA10 HOXA5 HOXB7 HOXC10 HOXD1 HOXD3 HOXD3///HOXD4///MIR10B HPCAL4 HPR HR44 HRH4 HS2ST1 HS2ST1///LOC339524 HSD17B2 HSD3B1 HSF2 HSP90AA1 HSPA12A HSPA1A///HSPA1B HSPA4 HSPA4L HSPB3 HSPC072 HTN1 HTR1F HTRA2 HYDIN ICMT ID2///ID2B ID2B IDI1 IDS IER3 IER3IP1 IFI16 IFI27 IFI35 IFI44 IFI44L IFI6 IFITM1 IFITM3 IFNA16 IFNA2 IFNA7 IFRD2 IFT27 IGF1 IGF2///INS-IGF2 IGF2BP3 IGFBP2 IGFBP5 IGFBP7 IGHA1///IGHG1///IGHG2///IGHG3///IGHM///LOC100126583///LOC100290036 IGHA1///IGHG1///IGHM///IGHV3-23///IGHV4-31 IGHG1///IGHM///LOC100133862 IGHMBP2 IGK@///IGKC///LOC652493///LOC652694 IGKC IGKC///IGKV1-5///IGKV1D-8///LOC652493///LOC652694 IGKV1D-8 IGL@ IKZF3 IL17A IL17RA IL18R1 IL19 IL1B IL1F7 IL1R1 IL1R2 IL1RN IL21R IL23A IL25 IL3 IL32 IL6ST IL8 IL9R ILF3 IMP3 IMP4 IMPA1 IMPA2 IMPDH2 IMPG1 INO80D INPP5K INSL5 INSM1 INSR INTS1 IPO9 IQCB1 IQCC IQCK IQSEC1 IRF9 ISG15 ISG20L2 ITGA10 ITGA2B ITGAV ITGB1BP2 ITGB3 ITM2B ITPKB ITPR1 ITSN1 IVD JAG1 JAK3 JAKMIP2 JUND KALRN KATNB1 KCNA5 KCNAB1 KCNH1 KCNIP1 KCNJ1 KCNJ13 KCNJ14 KCNJ16 KCNJ2 KCNJ5 KCNJ8 KCNK2 KCNMA1 KCNMB4 KCNN3 KCNQ1 KDELR3 KDM4A KDM4B KDM5A KDM5B KDM5D KDM6A KDM6B KDSR KERA KHDRBS1 KIAA0040 KIAA0125 KIAA0319 KIAA0391 KIAA0495 KIAA0556 KIAA0562 KIAA1033 KIAA1045 KIAA1659 KIAA1751 KIF14 KIF16B KIF17 KIF18A KIF18B KIF25 KIF2A KIFAP3 KIR2DL3 KIR2DL4 KIR2DS3 KIR3DL1///KIR3DL2///LOC727787 KITLG KLC1 KLF6 KLHDC10 KLHL18 KLHL20 KLHL23 KLK10 KLK3 KLK6 KLRC1///KLRC2 KLRC4 KPNA1 KPNA2 KPNA5 KRT34 KRT35 KRT38 KRT7 KYNU LAG3 LAGE3 LAIR2 LALBA LAMA2 LAMA4 LAMB1 LARP7 LARS2 LAT///SPNS1 LBR LCAT LCK LCT LDB3 LDHAL6B LEFTY1 LEFTY2 LGALS13 LGALS3BP LGSN LHCGR LIMCH1 LIMD1 LIMK1 LIPC LMF1 LMOD1 LMTK2 LOC100127886 LOC100131510 LOC100134822///LOC100288069 LOC100272228 LOC100286895 LOC100287076 LOC100287163///ZNF717///ZNF73 LOC100287927 LOC100288679 LOC100507397 LOC100508797 LOC100510546///STX5 LOC1720 LOC220077 LOC254896 LOC257152 LOC283079 LOC339290 LOC389906 LOC400573 LOC441204 LOC441601 LOC442421///LOC728297 LOC51152 LOC644450 LOC728392///NLRP1 LOC728825///SUMO2 LOC729991 LOH3CR2A LONP1 LPAL2 LPAR1 LPAR3 LPAR6 LPGAT1 LPHN3 LPIN1 LPPR4 LRCH4 LRFN4 LRP1B LRP2 LRP8 LRRC19 LRRC23 LRRC31 LRRC37A4 LRRC41 LRRC47 LRRC50 LRRN2 LSM14B LSM5 LSM7 LSS LST1 LTB4R LTBP2 LTBP3 LUC7L3 LUM LY6D LY6E LY6G6C LY9 LYRM4 LYZ MAB21L1 MAB21L2 MACF1 MAD2L1 MAEA MAF MAGEA4 MAGEA9///MAGEA9B MAGEB4 MAGOH MAL MALL MALT1 MAN1C1 MAOB MAP1B MAP2 MAP2K2 MAP2K5 MAP2K6 MAP3K14 MAP3K4 MAP3K5 MAP4K1 MAPK13 MAPK4 MAPK7 MAPKAPK2 MAPRE3 MARK2 MAT2A MATN1 MAU2 MAX MBD1 MBD5 MBL2 MBNL2 MCF2 MCL1 MCM3AP MCM3AP-AS MCM4 MCTP2 MCTS1 MDFIC MDH2 MDM1 MDM4 ME1 MECOM MED12 MED13 MED14 MED15 MED20 MED22 MED31 MED8 MEF2D MEFV MEGF6 MEIS2 MEN1 MEP1A METT10D METTL13 MFAP3L MFAP5 MFGE8 MFN2 MGC12488 MGC2889 MGC5590 MGEA5 MGP MIA2 MICAL2 MINK1 MKL1 MKL2 MLANA MLF1 MLF1IP MLLT1 MMACHC MMP12 MMP16 MMP3 MMP9 MN1 MNS1 MOCOS MPHOSPH9 MPO MPP2 MPPE1 MPPED2 MPRIP MRPS16 MRPS17///ZNF713 MRPS18B MRPS2 MRPS31 MRPS34 MRTO4 MS4A5 MSH4 MSH6 MSL3 MST1P9 MSTN MTAP MTM1 MTMR2 MTMR3 MTMR4 MTMR7 MTMR8 MTOR MTSS1 MUC2 MUC3B MUC5AC MX1 MX2 MXI1 MXRA8 MYC MYCBP2 MYCN MYCT1 MYEF2 MYH4 MYLK MYLK3 MYO1B MYOT MYOZ2 MYST4 MYT1L N4BP2L2 N6AMT1 NAA11 NAB1 NAB2 NACA NADSYN1 NAP1L3 NAT1 NAT2 NAT8 NAV3 NBN NCAPG2 NCBP1 NCBP2 NCKIPSD NCL NCLN NCOA1 NCOA2 NCOR2 NCRNA00287 NDUFA2 NDUFA3 NDUFA6 NDUFB2 NDUFB7 NDUFB8 NDUFS7 NDUFS8 NEBL NEFM NEIL3 NEK1 NELL2 NEUROD1 NEUROD2 NEUROD6 NF1 NF2 NFAT5 NFATC2IP NFIB NFIC NFIX NFKB2 NFKBIB NFX1 NFYB NGFR NHLH2 NID1 NIPSNAP3B NIT1 NKG7 NKRF NKTR NMBR NMT1 NMT2 NOC3L NOLC1 NONO NOS1 NOS3 NOTCH1 NOVA1 NOX1 NPAS1 NPAS2 NPEPPS NPHS2 NPM1 NPY5R NPY6R NQO1 NR0B1 NR1D1///THRA NR2C1 NRF1 NRG1 NRIP3 NRN1 NRP2 NRXN1 NSD1 NSMCE4A NSUN6 NSUN7 NT5C2 NTRK3 NUDCD3 NUDT3 NUMA1 NUP133 NUP43 NUP50 NUP62CL NUTF2 OAS1 OAZ2 OBFC2B ODZ1 OGFR OGN OGT OLFML1 OMD OPHN1 OPRL1 OR10C1 OR10H3 OR12D3 OR12D3///OR5V1 OR1D2 OR1E1 OR2A20P///OR2A9P OR2B2 OR2F1///OR2F2 OR2J2 OR2W1 OR52A1 OR5I1 OR7A5 OR7E47P ORAI2 ORM1 ORM1///ORM2 OS9 OSBPL10 OSGIN1 OSGIN2 OTUB1 OTUD4 OXTR P2RY14 P2RY6 PABPC4 PABPN1 PACRG PADI4 PAFAH2 PAICS PAK1IP1 PALMD PAMR1 PANK4 PANX1 PAPOLB PAPPA PAPPA2 PAPSS2 PARD6B PARP1 PARP12 PARP8 PAX8 PBRM1 PBXIP1 PCBP2 PCDH8 PCDH9 PCDHA2 PCDHB11 PCGF3 PCLO PCNP PCNXL2 PCSK2 PCSK6 PDAP1 PDCD10 PDCD1LG2 PDCD4 PDCL PDE12 PDE1C PDE3A PDE4B PDE4DIP PDE5A PDHA2 PDIA3 PDIA6 PDK1 PDLIM1 PDLIM3 PDLIM5 PDPK1 PDPR PDZD8 PDZK1 PDZRN4 PER1 PEX11B PEX5L PFAS PFKL PFKM PGK1 PGLS PGRMC1 PHB PHF14 PHF15 PHKA1 PHLDA2 PHLPP2 PI15 PI3 PIAS2 PIAS4 PIGH PIGL PIK3R1 PIK3R4 PIN4 PIP5K1C PIR PITPNB PITRM1 PKNOX2 PLA1A PLAC1 PLCB4 PLD1 PLEC PLEK PLEKHM2 PLK3 PLN PLOD2 PLXNC1 PMPCB PMS2///PMS2CL PMS2P3 PNLIPRP2 PNMA2 PNN PNO1 POLR1E POLR2J4 POLR2L POLR3B POLR3G POM121 POMZP3///ZP3 POP4 POPDC3 POSTN POU2F3 POU3F1 POU4F2 PPAP2B PPARD PPAT PPEF1 PPEF2 PPFIA4 PPFIBP1 PPFIBP2 PPIG PPM1E PPP1R12B PPP1R15A PPP1R3A PPP1R3D PPP2R1B PPP2R2D PPP3CB PPP4C PQLC1 PRAMEF12 PRB4 PRDM1 PRDM12 PRDM2 PRDX1 PREB PRELP PRF1 PRG4 PRKAB2 PRKAR1B PRKAR2A PRKCE PRKG1 PRKRIP1 PRL PRO2012 PROL1 PROS1 PRPF18 PRPF40A PRR16 PRRX1 PRUNE2 PSG1 PSG3 PSG6 PSG7 PSG9 PSMB10 PSMB2 PSME4 PSMF1 PSORS1C1 PSPH PSTPIP2 PTBP1 PTCH1 PTGER3 PTH PTP4A2 PTPN18 PTPRB PTPRJ PTPRR PUM1 PUM2 PUS1 PUS7 PXDN PZP QRSL1 QSER1 RAB11B RAB2A RAB40B RAB6B RAB9BP1 RABEPK RABGAP1 RABGGTA RAC1 RAD1 RAD21 RAD51L1 RALBP1 RANBP3 RAP1GDS1 RAPGEF2 RAPGEF3 RARA RARS2 RASGRP2 RASIP1 RASSF1 RAVER2 RBBP4 RBBP6 RBM10 RBM19 RBM25 RBM28 RBM34 RBMS2 RCAN1 RCBTB2 RCC1 RCHY1 RCN3 RECK REEP1 REEP5 REPS1 RERGL RFC4 RFPL1S RFPL2 RFPL3 RFPL3S RFX3 RFXANK RGS1 RGS5 RGS7 RHBG RIMS2 RLBP1 RMND5B RNASE1 RND2 RNF114 RNF126 RNF17 RNF8 RNFT2 RNMT RNPEPL1 ROS1 RPA3 RPAIN RPE65 RPIA RPL10 RPL13 RPL22 RPL35A RPL36 RPS14 RPS18 RPS4Y1 RPS6KA3 RPS6KA6 RPS6KB2 RPS8 RQCD1 RRAS RRBP1 RREB1 RRN3P1 RRP1B RRP8 RRS1 RS1 RTP4 RUFY3 RUNDC3A RUNX1 RUNX1T1 RWDD1 RYBP RYR2 RYR3 S100B S100G S1PR4 SAC3D1 SAFB2 SALL2 SAMD4A SAMD4B SAP30 SART3 SBF1 SCAND2 SCAP SCAPER SCARB2 SCARF1 SCD5 SCG5 SCGB1D2 SCGB2A1 SCN11A SCN1A SCN3A SCN3B SCNN1A SCO2 SCRG1 SCT SDCCAG3 SDF2L1 SDF4 SDHB SDHC SDHD SEC14L1 SEC14L4 SEC22B SEC24A SEC24B SEC31A SEC31B SEC61G SEC63 SECISBP2L SECTM1 SELL SELP SEMA3B SEMG1 SEPHS1 SERBP1 SERPINA6 SERPINB2 SERPINE1 SERTAD3 SETD1B SEZ6L SF1 SF3B4 SFRP4 SFTPB SFTPD SGK1 SGPL1 SH2B2 SH2D3C SH3GL2 SH3GL3 SHANK2 SHC3 SHPK///TRPV1 SIGLEC15 SIKE1 SIRT3 SIRT4 SLAMF7 SLC10A1 SLC10A3 SLC12A3 SLC12A5 SLC13A1 SLC14A1 SLC15A1 SLC16A1 SLC16A4 SLC18A1 SLC1A4 SLC1A7 SLC20A1 SLC22A6 SLC24A1 SLC24A3 SLC25A13 SLC25A14 SLC25A16 SLC25A21 SLC25A30 SLC25A40 SLC2A2 SLC2A4 SLC2A4RG SLC30A5 SLC33A1 SLC35A2 SLC35D1 SLC38A2 SLC39A6 SLC4A10 SLC4A4 SLC4A7 SLC5A1 SLC5A12 SLC6A15 SLC7A11 SLC7A6 SLC7A9 SLC9A1 SLC9A7 SLCO3A1 SLCO5A1 SLIT3 SLITRK3 SMA4 SMAD4 SMARCA2 SMARCA4 SMARCA5 SMARCC1 SMC1A SMC5 SMR3A SMR3B SNAP23 SNAPC5 SNCA SNRNP70 SNRPA1 SNRPB SNRPD3 SNRPF SNW1 SNX7 SOBP SOD2 SOHLH2 SOLH SORBS1 SOS2 SOX11 SOX2 SOX9 SP2 SP3 SP3P SP4 SPAG6 SPAM1 SPATA2L SPATA6 SPHK1 SPINK1 SPINK4 SPO11 SPON1 SPOP SPP1 SPRR1B SPRR3 SPTBN1 SPTLC2 SPTLC3 SQSTM1 SR140 SRD5A2 SREBF2 SRM SRP72 SRP9 SRPK2 SRPR SRPRB SRRD SRRT SRSF11 SRSF7 SRSF9 SSR1 SSX1 SSX2///SSX2B SSX2///SSX2B///SSX3 SSX7 ST13 ST6GAL1 ST6GALNAC2 ST6GALNAC5 ST8SIA1 ST8SIA4 STAG3 STAG3L1 STAG3L4 STAMBPL1 STAP1 STARD3 STAT1 STC2 STIM1 STK11 STK16 STK24 STK25 STK3 STK38 STK39 STMN1 STMN2 STRN STS STT3A STX16 STYK1 SUCLG2 SUGP2 SULF1 SULT1B1 SULT1C2 SULT2A1 SUMO2 SUPT6H SUPV3L1 SUSD5 SUV39H2 SV2B SVEP1 SYCP1 SYCP2 SYDE1 SYNCRIP SYNE2 SYNPO2L T TAAR3 TAAR5 TAC1 TACC3 TACR3 TAF1C TAF1D TAF6L TAF9B TAGLN3 TALDO1 TAP1 TARDBP TARP///TRGV3///TRGV5 TAS2R1 TAS2R14 TAS2R7 TAS2R8 TAT TAX1BP1 TAX1BP3 TAZ TBC1D19 TBC1D2B TBCE TBL2 TBR1 TBRG4 TBX6 TBXA2R TCEAL4 TCERG1 TCF12 TCF3 TCF4 TCF7 TCF7L2 TCIRG1 TCN2 TCTN2 TDO2 TDRD1 TDRD3 TEAD1 TECR TEX12 TEX13A TEX15 TEX28 TFE3 TFF1 TFIP11 TFPI2 TGFB1 TGFBR3 TGS1 THBS1 THBS2 THPO THSD4 THSD7A THYN1 TIE1 TIMELESS TIMM8A TIMP4 TIPIN TJAP1 TLE4 TLL1 TLL2 TM4SF1 TM4SF4 TM9SF3 TMBIM4 TMBIM6 TMC7 TMCC1 TMED7 TMEM100 TMEM14A TMEM19 TMEM22 TMEM35 TMEM5 TMEM8A TMEM9B TMOD1 TMOD3 TMPRSS15 TMSB15A TMSB15B TMSB4Y TMX4 TNFAIP6 TNFRSF14 TNFSF11 TNK2 TNNI3K TNPO3 TNXB TOM1 TOMM22 TOP3A TP53 TP53AIP1 TP53TG3///TP53TG3B TP63 TPH1 TPM1 TPMT TPP1 TPTE TRA2B TRAC TRAC///TRAJ17///TRAV20 TRAF3IP2 TRAF3IP3 TRAF5 TRAIP TRANK1 TRBC1///TRBC2 TRBC2 TRD@ TRDN TREML2 TRGV5 TRH TRIM10 TRIM14 TRIM2 TRIM27 TRIM28 TRIM33 TRIM49///TRIM49L2 TRIM8 TRIO TRIP11 TRIP13 TRIP6 TRMT112 TRO TRPM1 TRPM3 TRRAP TSFM TSHB TSNAX TSPAN14 TSPAN2 TSPY1///TSPY3///TSPY4///TSPY8 TSPYL4 TSR1 TSSC1 TSSK2 TTC13 TTC18 TTC22 TTC31 TTI1 TTLL1 TTLL7 TUBA4A TUBAL3 TUBG1 TUBGCP2 TUG1 TULP2 TUSC3 TWF1 TXK TXN TXNRD1 TYK2 TYMP TYRP1 U2AF2 UBAP2L UBC UBE2D2 UBE2E1 UBE2H UBE2I UBE3A UBE4B UBFD1 UBIAD1 UBP1 UBQLN2 UBR7 UBTF UCHL1 UCKL1 UGT2B15 UGT2B17 UIMC1 ULK2 UMPS UNC45A UPF3A UPK2 UQCRC1 URB1 USE1 USF2 USP1 USP10 USP24 USP27X USP33 USP47 USP48 USP9Y UTP6 UTY VAMP1 VAMP2 VAPB VARS VBP1 VCL VDAC3 VEGFB VGF VGLL3 VPS13B VPS13D WARS2 WBP11 WBP4 WDFY3 WDR18 WDR3 WDR46 WDR55 WDR59 WDR61 WDR74 WDR78 WDR82 WEE1 WHSC1 WHSC1L1 WHSC2 WIPI1 WISP1 WNK1 WNT2B WSB1 XPNPEP1 XPNPEP2 XRCC2 XYLB YAF2 YLPM1 YME1L1 YPEL5 YTHDC2 YTHDF2 YTHDF3 YWHAE YY1 ZAK ZAP70 ZBED5 ZBTB43 ZC3H15 ZC3H7B ZC3HAV1 ZCCHC4 ZDHHC17 ZDHHC3 ZEB1 ZFAND5 ZFHX4 ZFY ZFYVE9 ZIC1 ZKSCAN1 ZMYM2 ZMYM4 ZNF117 ZNF132 ZNF14 ZNF154 ZNF157 ZNF192 ZNF195 ZNF207 ZNF208 ZNF22 ZNF222 ZNF225 ZNF235 ZNF254 ZNF257 ZNF268 ZNF276 ZNF280D ZNF304 ZNF330 ZNF334 ZNF37A///ZNF37BP ZNF407 ZNF410 ZNF419 ZNF43 ZNF440 ZNF468 ZNF492 ZNF507 ZNF518A ZNF562 ZNF571 ZNF580 ZNF586 ZNF639 ZNF643 ZNF654 ZNF702P ZNF747 ZNF75D ZNF770 ZNF771 ZNF79 ZNF80 ZNF804A ZNF816 ZNF83 ZNF835 ZNF839 ZNF85 ZNHIT1 ZNHIT6 ZSCAN12 ZXDA///ZXDB ZXDB ZXDC ZZEF1 | BAL_26_A2B0 BAL_17_A0B0 BAL_3_A1B0 BAL_15a_A0B1 BAL_44b_A0B0 BAL_45a_A1B0 BAL_45b_A2B0 BAL_4_A0B0 BAL_33_A0B1 BAL_1_A0B0 BAL_43_A0B0 BAL_13_A0B0 BAL_40_A0B0 BAL_18_A1B0 BAL_37_A1B1 BAL_39_A0B1 BAL_12_A1B0 BAL_27b_A2B1 BAL_27a_A0B0 BAL_25_A0B0 BAL_15b_A0B0 BAL_31_A0B1 BAL_47b_A0B1 BAL_20b_A1B1 BAL_41_A1B0 BAL_47c_A0B1 BAL_42_A0B0 BAL_47a_A0B0 BAL_19_A0B0 BAL_44a_A0B0 BAL_21_A0B0 BAL_29_A1B0 |
| **2** | AANAT ABAT ABCA11P ABCA2 ABCB11 ABCC9 ABCD2 ABCD4 ABCE1 ABCF1 ABCF2 ABHD6 ABI1 ABLIM3 ACD ACHE ACLY ACO2 ACOX1 ACP1 ACPP ACRV1 ACSL6 ACTB ACTR2 ACVR1 ACVR2A ADAM22 ADAM28 ADAMTS5 ADAMTS6 ADAMTS8 ADCK2 ADCK4 ADD1 ADD3 ADH5 ADIPOR1 ADNP ADORA3 ADRA2A ADRB3 ADSS AFF1 AGPAT1 AGRN AGXT AHCYL1 AHI1 AKAP1 AKAP9 AKR1B10 AKR1C1 AKT2 ALCAM ALDH1A1 ALDH3B1 ALDH5A1 ALDH7A1 ALOX5 ALPP///ALPPL2 AMACR///C1QTNF3 AMDHD2 ANAPC13 ANGEL2 ANGPT4 ANGPTL2 ANKRA2 ANKRD1 ANKRD12 ANKRD36BP2 ANKRD6 ANP32A///ANP32D ANP32C ANXA4 ANXA7 APAF1 APBA2 APC APH1A API5 APIP APLP2 APOA2 APOD APOH APOOL APP APPBP2 APPL2 ARF1 ARF3 ARF4 ARFGEF1 ARHGAP11A ARHGAP6 ARHGDIA ARHGEF12 ARIH1 ARL17A///ARL17B ARMCX6 ARPC1A ASB13 ASCC2 ASF1A ASF1B ASNA1 ASNS ASPA ASPH ATF6 ATF6B///TNXB ATF7 ATG16L1 ATG4B ATG5 ATM ATP1A3 ATP2A2 ATP2A3 ATP5G2 ATP6V0E1 ATP6V1G1 ATP8A1 ATRN ATRX ATXN3 ATXN7 AURKA AVIL AZIN1 BACH2 BAK1 BAT2L2 BAX BAZ2B BBS9 BCAS2 BCKDHB BCL2L1 BCL2L2 BEX4 BIRC2 BLCAP BLVRA BLZF1 BMP8A BMP8B BMPR1A BMS1 BNIP1 BPHL BPY2 BRD4 BRD8 BSG BTBD7 BTF3 BTF3P11 BTG1 BTG2 BTG4 BTRC BUD31 BYSL BZW1 C10orf68 C11orf24 C11orf30 C11orf41 C11orf57 C14orf1 C14orf104 C14orf147 C14orf2 C15orf39 C16orf42 C16orf68 C16orf7 C17orf101 C17orf86 C18orf10 C19orf2 C19orf26 C19orf40 C19orf42 C19orf50 C19orf60 C1orf107 C1orf123 C1orf175///TTC4 C1orf63 C1orf77 C1QBP C1S C20orf30 C22orf26 C2orf3 C2orf43 C2orf67 C4orf34 C4orf41 C5orf4 C6orf162 C6orf25 C6orf62 C6orf64 C6orf97 C8orf71 C9orf114 C9orf40 C9orf78 C9orf82 C9orf86 CA5BP CABYR CACNB3 CALB2 CALCRL CALM1 CALY CAND1 CANT1 CANX CAPN3 CAPN7 CAPRIN1 CAPZB CARD14 CASP7 CASQ2 CAST CAT CBFA2T3 CBLL1 CBX1 CCDC53 CCDC59 CCL5 CCL8 CCNI CCNL2 CCPG1 CCR8 CCT2 CCZ1 CD164 CD1C CD28 CD34 CD44 CD47 CD55 CD59 CD84 CD9 CD93 CD99 CDC14B CDC34 CDC42 CDC42EP2 CDH20 CDH6 CDIPT CDK1 CDK11A///CDK11B CDK13 CDK2 CDK6 CDKAL1 CDKN2D CDR1 CDX4 CEACAM5 CEACAM6 CEBPD CENPB CENPJ CEP110 CEP57 CEP68 CEPT1 CER1 CES1 CFB CFD CFDP1 CFH CFLAR CHD2 CHD9 CHI3L1 CHIT1 CHKB-CPT1B///CPT1B CHN2 CIZ1 CLCN4 CLCNKA///CLCNKB CLDN14 CLDND1 CLINT1 CLK1///PPIL3 CLN5 CLTA CLTB CMAH CMAS CNKSR2 CNOT2 CNR1 CNTNAP2 COL11A2 COL17A1 COL4A3 COMT COPB1 COPS8 COX11 COX4I1 COX5B COX6C COX7C CPA2 CPNE3 CPSF6 CR1 CRAT CREBZF CRELD2 CRIM1 CRIPT CRYBB2 CSDA CSDE1 CSH1 CSNK1A1 CSNK2A1 CSPP1 CSRNP2 CTAG2 CTAGE9 CTBP2 CTCF CTCFL///HMGB1 CTNNA1 CTNND2 CTTN CUL4A CUZD1 CXCL5 CYB5A CYLC1 CYLD CYP1A1 CYP1B1 CYP2A7P1 CYP3A5 CYP4A22 CYP4F3 CYP51A1 DACT1 DAZ1///DAZ2///DAZ3///DAZ4 DAZAP2 DBC1 DBH DBI DCAF16 DDC DDHD2 DDX11///DDX12///LOC642846 DDX17 DDX3X DDX3Y DDX51 DEFB1 DEFB4A DENND1A DENND1C DES DGCR14 DGKG DHRS2 DHRS4///DHRS4L2 DHRS7 DHX15 DHX34 DHX9 DIABLO DIAPH1 DICER1 DIMT1L DIO2 DIS3 DKC1 DKK3 DKK4 DLAT DLG1 DLX4 DNAH3 DNAJB1 DNAJB6///TMEM135 DNAJC12 DNMT3L DOCK1 DOCK9 DOK5 DOPEY1///LOC100509911 DPAGT1 DPH5 DPM1 DPP4 DPT DST DTNA DUOX2 DUSP1 DUSP22 DYNLRB1 E2F1 EDAR EDDM3A EDEM3 EDNRB EEF1A1 EEF1D EEF1E1 EFNA4 EFR3B EGFR EHD2 EID1 EIF1 EIF1AX EIF1AY EIF2AK1 EIF2B4 EIF2B5 EIF2C1 EIF2S2 EIF2S3 EIF3A EIF3C///EIF3CL EIF3E EIF3H EIF4B EIF4E EIF4H ELAVL1 ELAVL4 ELOVL1 ENDOD1 ENDOU ENO1 ENOX1 ENPP1 ENPP4 ENTPD4 ENTPD7 EPAS1 EPN2 EPOR EPS15L1 EPS8L1 ERAP1 ERCC3 ERGIC2 ERLIN1 ERLIN2 ESPL1 ETS2 ETV5 EVX1 EWSR1 EXOC3 EXOG EXOSC7 EXOSC9 EXT2 EZR F13A1 F13B F5 F7 FABP1 FABP7 FAHD2A FAHD2A///FAHD2B///LOC729234 FAM108A1 FAM134A FAM134B FAM149A FAM153A FAM158A FAM168B FAM188A FAM18B1 FAM193B FAM198B FAM47E///STBD1 FAM48A FAM55C FAM57A FANCG FAS FBLN1 FBN1 FBXL14 FBXL6 FBXO11 FBXO21 FCF1///LOC100507758 FCHO1 FDX1 FEM1B FEZF2 FGF1 FGF16 FGFR1 FGFR2 FGFR3 FH FIP1L1 FKBP11 FKBP1B///MFSD2B FLCN FLII FLJ10038 FLJ11292 FLJ13197 FLOT1 FMO4 FNTA FOLR1 FOXA2 FOXG1 FOXK2 FOXM1 FOXO3///FOXO3B FPGT FRY FSCN2 FUBP1 FURIN FUS FUT3 FUT9 FYB FZD2 FZD6 G3BP1 GABARAPL2 GABBR1///UBD GABRA3 GABRB2 GAD1 GADD45B GAGE1///GAGE12C///GAGE12D///GAGE12E///GAGE12F///GAGE12G///GAGE12H///GAGE12I///GAGE12J///GAGE2A///GAGE2C///GAGE2D///GAGE2E///GAGE4///GAGE5///GAGE6///GAGE7///GAGE8 GAGE1///GAGE12F///GAGE12G///GAGE12I///GAGE12J///GAGE4///GAGE5///GAGE6///GAGE7 GAGE12F///GAGE12G///GAGE12I///GAGE5///GAGE7 GAK GAL GALR3 GAP43 GAPDH GATAD1 GATM GC GCC2 GCSH///LOC100329108 GDF11 GDI1 GDI2 GEMIN4 GGA1 GGCX GGPS1 GGT1 GGTLC1 GH2 GHRHR GJA5 GK GK3P GLP1R GLRA2 GLUL GLYR1 GMCL1 GMEB2 GMPR GNAI3 GNAS GNAT3 GNB1 GNB2 GNE GNG11 GNL3L GOLGA2 GOLGA8B GOSR2 GP1BB GP2 GPBP1L1 GPN1 GPR107 GPR135 GPR172A GPR3 GPR32 GPR6 GPR63 GPR97 GRIA4 GRIK2 GRSF1 GSPT1 GSS GSTM4 GTF2H1 GTF3C3 GTPBP10 GUCA1A GUCA2B GYPB H2AFV H3F3A///LOC440926 H3F3B HAB1 HADH HAT1 HBA1///HBA2 HBBP1 HBE1 HBZ HCFC1 HCG4P6 HCG9 HDAC2 HDGFRP3 HEMK1 HERC4 HERPUD1 HES1 HFE HGD HGF HIBCH HIPK1 HIST1H2BH HIST1H2BI HIST1H3A HLA-DOA HLA-DRA HLA-DRB6 HLA-G HLTF HMGB1 HMGCS2 HMGN1 HNMT HNRNPA0 HNRNPA2B1 HNRNPA3 HNRNPC HNRNPD HNRNPF HNRNPH3 HNRNPM HNRNPU HOMER3 HOXA10///HOXA9 HOXB8 HOXC5 HPGD HRK HSP90AA1 HSP90AB1 HSP90B1 HSPA1A HSPA4L HSPA8 HSPA9 HSPB1 HSPB3 HSPC157 HSPD1 HSPH1 HTN1 HTR1B HTR1F HTR2C HTR5A HTR7 HTT HUWE1 HYOU1 IARS ICA1 ICAM3 ICMT IDH3G IDO1 IDS IFITM1 IFNA1 IFNA2 IFNA7 IGFBP6 IGHA1 IGHA1///IGHA2///IGHG1///IGHG4///IGHM///IGHV4-31 IGHA1///IGHG1///IGHM IGHD IGHG1 IGKC IGL@ IGLJ3 IHH IK///TMCO6 IKBKAP IKBKB IKZF2 IL1R2 IL21 IL21R IL24 IL27RA IL33 IL5RA IL6R IL6ST ILF3 IMMT INCENP INSL3 INVS IPO5 IQSEC2 IREB2 IRGC ISCA1 ITGA7 ITGAE ITGB3BP ITGB4 ITGBL1 ITM2B ITSN1 ITSN2 JAG1 JMJD4 JMJD6 JUND KAL1 KARS KAT5 KAZALD1 KCMF1 KCNAB1 KCNC4 KCND1 KCNIP1 KCNJ3 KCNK10 KCNMB3 KCTD12 KCTD14 KCTD3 KCTD9 KDELR1 KDELR3 KDM4B KDM5A KDM5D KHDRBS1 KHK KIAA0090 KIAA0125 KIAA0467 KIAA0748 KIAA0776 KIAA1024 KIAA1279 KIAA1609 KIDINS220 KIF15 KIF2A KIF5B KIR2DL3 KIR2DL5A KL KLC2 KLF1 KLF12 KLF6 KLHDC10 KLHL22 KLHL35 KLK15 KLRB1 KLRC1///KLRC2 KPNA5 KPNB1 KRIT1 KRT6A KYNU L1CAM LALBA LAMA4 LAMB1 LAMP1 LAMP3 LARGE LARP1 LASS6 LAT///SPNS1 LCN1 LECT2 LGALS3BP LGALS8 LHFP LHX3 LIG4 LILRA5 LIMK2 LMAN1 LMAN2L LMCD1 LMNA LMO1 LMO3 LMO4 LMO7 LOC100127972 LOC100130633///ZMYM6 LOC100131825 LOC100287515///ZNF26 LOC100288142///NBPF1///NBPF10 LOC100499177 LOC100507009 LOC100507328///LOC100508591 LOC100507619 LOC100507666///NPIPL2 LOC100509661///OVOL3 LOC100510735///RPL29 LOC145678 LOC150759 LOC155060 LOC283079 LOC389906 LOC400573 LOC51152 LOC81691 LPAL2 LPHN2 LPIN1 LPPR1 LRFN4 LRIT1 LRRC1 LRRC17 LRRC23 LRRC37A4 LRRC41 LRRC68 LSM14A LSM14B LSM2 LSM4 LSP1 LSS LTB LTB4R LTBP1 LY6D LY6G6D LYRM2 LZTR1 M6PR MACF1 MAD1L1 MAGEB1 MAGOH MALL MAN1C1 MAN2A2 MAN2B2 MANEA MANF MAP2K2 MAP2K4 MAP2K5 MAP3K4 MAP3K7 MAP4 MAP4K1 MAP7D3 MAST2 MAT2A MATN4 MATR3 MAX MBD1 MBD2 MBP MBTPS1 MC3R MCAM MCCC2 MCL1 MCM10 MCM2 MCM3AP MCTS1 MDM1 MDM4 MEAF6 MED16 MED25 MED7 MEF2A MEF2C METAP1 METAP2 METTL1 METTL13 METTL2B METTL3 MEX3C MFI2 MFN2 MFSD5 MGAT2 MGAT4B MGC29506 MGST2 MICAL2 MID1 MIR1244-1///MIR1244-2///MIR1244-3///PTMA///PTMAP5 MKI67 MKL1 MKRN1 MLH3 MLL MLLT4 MLN MMP12 MMP16 MOG MORF4///MORF4L1 MOS MOXD1 MPPED2 MPZL1 MRPL13 MRPL18 MRPL20 MRPL24 MRPL34 MRPL9 MRPS18B MRS2 MSH6 MSL3 MSRB2 MST1 MTA1 MTAP MTERFD2 MTFR1 MTMR1 MTMR8 MTNR1B MTR MTRF1L MTUS1 MTX2 MUM1 MUS81 MXD4 MYCL1 MYH3 MYH6 MYH7B MYH8 MYL2 MYL6 MYO6 MYO7B MYOF N4BP1 N4BP2L2 N6AMT1 NAA35 NAA40 NACA NAP1L1 NASP NAT1 NBAS NBPF1///NBPF10///NBPF11///NBPF12///NBPF14///NBPF15///NBPF16///NBPF24///NBPF8 NBR1 NCAM1 NCAPG2 NCK1 NCLN NCOA3 NCR2 NDRG4 NDUFAF3 NDUFB8 NEBL NEFL NEK7 NELF NENF NEU3 NEUROG2 NF2 NFYB NHLH2 NHP2L1 NID1 NIF3L1 NIPBL NKG7 NLE1 NLGN1 NLRP3 NMT1 NMT2 NOL12///TRIOBP NOL7 NOLC1 NOMO1///NOMO2///NOMO3 NONO NOTCH2NL NOVA2 NOX1 NOX4 NPFF NPIPL2///NPIPL3///PDXDC2P NPM1 NPTXR NPVF NR0B1 NR1D2 NR1H2 NR2F1 NR2F6 NR4A1 NR4A2 NR4A3 NR5A2 NRBP1 NSMCE4A NT5C NTRK3 NTS NUBPL NUCKS1 NUDT13 NUP50 NUPR1 NXT2 OGG1 OLIG2 OPRD1 OPRM1 OR1G1 OR2J2 OR2S2 OR3A3 ORAI2 OSBPL7 OSBPL8 OSBPL9 OTUB1 OXR1 PABPC1 PABPC1///RLIM PABPC3 PACS1 PACS2 PAFAH1B1 PAIP1 PANK2 PAPOLA PAPPA PAPPA2 PARP4 PATZ1 PAX5 PAXIP1 PBRM1 PBX1 PBXIP1 PCBD1 PCBP1 PCBP2 PCCA PCDHA6 PCDHB12 PCDHB13 PCF11 PCGF2 PCM1 PCMT1 PCMTD2 PCNXL2 PCYOX1 PDCD4 PDCD6 PDE4A PDE6B PDGFB PDGFRA PDIA6 PDLIM5 PDX1 PDXK PDYN PDZK1IP1 PEBP1 PER1 PEX10 PEX2 PEX26 PFDN1 PFDN6 PFKFB2 PGA3///PGA4///PGA5 PGC PGK1 PGLYRP4 PGR PGRMC1 PHACTR2 PHF10 PHF2 PHF20L1 PHF3 PI15 PI3 PI4KB PIAS2 PIK3CD PIK3CG PIN4 PIP5K1B PKLR PKN2 PKP4 PLA2G3 PLCG1 PLD1 PLEC PLEKHA1 PLGLB1///PLGLB2 PLIN1 PLSCR3 PLXNC1 PML PMS2///PMS2CL PNMA1 PNN POGLUT1 POLG POLR1C POLR2D POLR2E POM121L9P POMZP3 POP1 POP7 POU3F3 PPAP2B PPCDC PPDPF PPFIBP1 PPIB PPM1E PPP1R13L PPP1R7 PPP2R2B PPP3CC PRDM4 PRDX3 PRF1 PRIM2 PRKAR1A PRKCI PRKD3 PRKDC PRKX///PRKY PRPF4B PRPF8 PRSS16 PRSS3 PRSS50 PRUNE PSD3 PSMB1 PSPH PTBP1 PTCRA PTGER3 PTGES PTK2 PTMA PTMS PTPLAD1 PTPN11 PTPN12 PUS7 PUS7L PYGB PYY RAB14 RAB1A RAB28 RAB2A RAB3D RAB3GAP1 RAB3GAP2 RAB40B RAB4A RABEP2 RAC1 RAD51L1 RAD52 RAD54B RAG2 RALGAPB RANBP9 RAP1GAP RASA1 RASAL1 RASGRF1 RASGRP2 RBBP6 RBBP7 RBL2 RBM25 RBM28 RBM5 RBM6 RBM8A RBMS1 RBMX2 RBPMS RC3H2 RCBTB1 RCC1 RCN2 RCVRN RDX REEP1 REEP5 REG3A REL RERE RET RGL1 RHAG RHEB RIC3 RIF1 RIMS2 RMND5A RNASE1 RNASE4 RND2 RNF114 RNF130 RNF138 RNF186 RNF24 RNF8 RNFT1 ROCK2 ROD1 RORA RPA1 RPA4 RPL10 RPL10A RPL11 RPL12 RPL13 RPL14 RPL15 RPL18 RPL18A///RPL18AP3 RPL19 RPL23 RPL24 RPL26 RPL26L1 RPL29 RPL3 RPL31 RPL35 RPL35A RPL37A RPL4 RPL5 RPL6 RPL8 RPL9 RPLP0 RPLP0///RPLP0P6 RPLP2 RPN1 RPP38 RPS14P3 RPS15 RPS18 RPS2 RPS20 RPS4Y1 RPS6 RPS7 RPS8 RPS9 RRAGA RRBP1 RRM1 RRP12 RRP15 RSL24D1 RTCD1 RUNX1T1 RXRB RYBP RYK S100A7 S100A8 S100G S1PR2 SAMD14 SAP18 SART3 SC5DL SCAMP1 SCAPER SCEL SCFD1 SCGB1D1 SCO2 SCP2 SCRIB SDHB SDHC SDHD SEC14L1 SEC14L5 SEC23B SEC23IP SEC31A SEC63 SECTM1 SELT SEMA4F SENP6 SEPHS1 SEPT10 SEPT2 SEPT8 SEPX1 SERBP1 SERF2 SERINC3 SERPINB1 SERPINB13 SERPINB8 SERPINI1 SET SETD4 SF1 SF3A1 SFTPB SFTPC SGCG SGK1 SH2D1A SH3BP2 SHMT1 SHOX2 SHPK SIGLEC15 SIGLEC8 SIK3 SIM2 SIRPG SKP1 SLC11A2 SLC12A5 SLC12A7 SLC13A1 SLC14A1 SLC15A1 SLC17A3 SLC17A9 SLC22A17 SLC22A8 SLC23A2 SLC24A2 SLC24A3 SLC25A15 SLC25A5 SLC25A6 SLC26A4 SLC27A6 SLC28A2 SLC2A1 SLC30A3 SLC34A1 SLC38A2 SLC38A7 SLC39A1 SLC39A14 SLC4A7 SLC5A12 SLC5A2 SLC5A3 SLC6A2 SLC7A11 SLC7A5 SLC7A6 SLCO1B3 SLCO3A1 SLCO4C1 SLN SMAD4 SMARCA2 SMARCA4 SMARCA5 SMARCC1 SMARCD1 SMARCE1 SMC3 SMC4 SMG1 SMG6 SNAI2 SNAPC4 SNAPC5 SNCG SNRPA1 SNRPN///SNURF SNW1 SNX1 SNX13 SNX16 SNX17 SNX19 SNX29 SNX3 SNX4 SOD2 SOHLH2 SORBS3 SORL1 SOX1 SOX4 SP100 SPAG6 SPAM1 SPATA6 SPO11 SPR SPRED2 SPRR1A SPRR1B SPRR3 SPRY2 SPRY4 SPSB3 SPTBN1 SPTLC1 SPTLC2 SQLE SQSTM1 SRD5A3 SREBF2 SRPRB SRPX SRR SRRT SRSF10 SRSF2IP SRSF3 SRSF5 SRSF7 SRSF8 SRSF9 SS18 SS18L1 SSX2///SSX2B SSX3 SSX4///SSX4B ST13 ST6GALNAC5 ST8SIA1 STAG2 STAM2 STAT1 STAT2 STAT3 STAT5A STAT6 STAU1 STC1 STC2 STIP1 STK17A STK24 STK25 STK3 STK38 STRA6 STUB1 STX16 STX17 STX2 STXBP3 STYK1 SUCLG2 SUGP1 SULT2A1 SUMO1 SUMO2 SUSD5 SVEP1 SYCP2 SYNGR4 SYNJ2 SYPL1 TACSTD2 TAF1 TAF1C TAF6L TAF7L TAF9 TAP1 TATDN2 TAX1BP1 TBC1D16 TBC1D9B TBCD TBL1XR1 TBX19 TBX2 TCEA1 TCIRG1 TCP1 TCTN3 TDRD12 TEAD4 TECPR2 TERF1 TEX28 TFAP2A TFAP2C TFDP1 TFDP3 TFF1 TFG TFIP11 TFPI TFR2 TFRC TGM2 TH TH1L THAP4 THPO THRAP3 THSD7A TIMM17A TIMP1 TIMP3 TLE4 TM2D1 TM4SF1 TM9SF2 TM9SF4 TMBIM6 TMED2 TMEFF1 TMEM109 TMEM14B TMEM158 TMEM177 TMEM185B TMEM19 TMEM194A TMEM30B TMEM41B TMEM45A TMEM57 TMEM93 TMF1 TMOD1 TMOD3 TMSB15A TMX1 TNC TNF TNFAIP6 TNFRSF21 TNFRSF25 TNFSF18 TNIP1 TNKS TNPO1 TNPO2 TNRC6B TNS1 TOMM20 TOMM40 TOP2B TOP3A TOR1A TOX TOX4 TPD52 TPD52L1 TPD52L2 TPM2 TPM4 TPP2 TPPP TPR TPT1 TRADD TRAF3IP3 TRAFD1 TRAM1 TRAPPC4 TRAPPC8 TRBV10-2 TRD@ TRDN TREX1 TRHR TRIM16 TRIM37 TRMT61A TRPA1 TRPC3 TRPM2 TRPV1 TSPAN3 TSPYL5 TSR1 TSSK2 TTBK2 TTC3 TTF1 TTF2 TTR TUB TUBA3D TUBB TUBGCP5 TUFM TUG1 TWIST1 TWSG1 TXNIP TXNL4A TXNRD3 UBAP1 UBC UBE2D3 UBE2H UBE2M UBE2NL UBE3B UBL3 UBOX5 UBQLN3 UBR5 UBR7 UBXN4 UCHL1 UCP2 UGCG UGT2A1///UGT2A2 UGT2B28 UGT8 UHRF1BP1L UPF3A UQCRC2 URB2 USE1 USF2 USP10 USP16 USP19 USP4 USP46 USP48 USP7 USP9Y UTRN VAC14 VAMP3 VDAC3 VEZF1 VGLL1 VHL VPREB3 VPS41 VPS72 VSIG10 WAC WBP11 WDHD1 WDR1 WDR48 WDR74 WHSC2 WIPF2 WIPI1 WNT16 XIAP XIST XPC XPNPEP1 XRCC5 YAF2 YBX1 YIF1A YIPF5 YIPF6 YLPM1 YTHDC2 YWHAE YWHAQ YWHAZ YY1 ZBED4 ZBTB1 ZBTB38 ZBTB40 ZBTB48 ZBTB7A ZC3H15 ZC3H4 ZC3HAV1 ZDHHC17 ZFAND5 ZFHX3 ZFP106 ZFP161 ZFP36L2 ZFPL1 ZFYVE21 ZKSCAN5 ZMAT4 ZMIZ2 ZMYND11 ZMYND8 ZNF140 ZNF141 ZNF148 ZNF195 ZNF207 ZNF208 ZNF212 ZNF215 ZNF236 ZNF239 ZNF24 ZNF248 ZNF266 ZNF277 ZNF281 ZNF286A ZNF318 ZNF329 ZNF331 ZNF37A///ZNF37BP ZNF410 ZNF419 ZNF430 ZNF460 ZNF484 ZNF510 ZNF552 ZNF580 ZNF593 ZNF609 ZNF646 ZNF665 ZNF692 ZNF706 ZNF771 ZNF787 ZNF79 ZNF93 | BAL_17_A0B0 BAL_3_A1B0 BAL_26_A2B0 BAL_39_A0B1 BAL_45a_A1B0 BAL_1_A0B0 BAL_43_A0B0 BAL_13_A0B0 BAL_18_A1B0 BAL_37_A1B1 BAL_25_A0B0 BAL_33_A0B1 BAL_4_A0B0 BAL_44b_A0B0 BAL_27a_A0B0 BAL_40_A0B0 BAL_47b_A0B1 BAL_12_A1B0 BAL_45b_A2B0 BAL_42_A0B0 BAL_15a_A0B1 BAL_20b_A1B1 BAL_47c_A0B1 BAL_31_A0B1 BAL_47a_A0B0 BAL_15b_A0B0 BAL_41_A1B0 BAL_27b_A2B1 BAL_44a_A0B0 BAL_29_A1B0 |
| **3** | AAK1 ABCA7 ABCB4 ABCB9 ABCC8 ABCD3 ABHD2 ABI2 ABLIM1 ABO ACAA2 ACHE ACO2 ACOT7 ACRV1 ACSL4 ACSM2A///ACSM2B ACSM3 ACTA1 ACTN2 ACTN4 ACTR6 ADAM11 ADAM19 ADAM23 ADAM28 ADAM5P ADAM8 ADAMTS3 ADAMTS5 ADAP1 ADCY8 ADH1B ADRA1D AGAP2 AGXT AICDA AKAP1 AKAP8L ALAD ALKBH4 ALOXE3 ALPPL2 AMACR ANGPT1 ANK1 ANKRD1 ANKRD11 ANKRD36B ANKRD53 AP4E1 API5 APOA4 APOBEC3A APOC3 APOD APOE AQP6 AQP7 ARAP2 ARFRP1 ARHGAP11A ARHGAP33 ARHGDIA ARHGDIG ARHGEF12 ARID1A ARID4B ARL17A///LOC100294341 ARL3 ARL6IP4 ARMC7 ARSD ARSE ASCL1 ASGR2 ATP12A ATP1B1 ATP6V0E1 ATP8A1 ATP9A ATR ATXN2L ATXN3 AVL9 B3GALNT1 B3GALT4 B3GNT3 B4GALT1 B4GALT6 B4GALT7 BACH2 BAIAP2L2 BANK1 BARD1 BARX1 BAT2 BAT2L2 BAZ2A BAZ2B BBC3 BBS7 BBS9 BBX BCHE BCL2 BCL3 BEST1 BICC1 BLNK BLVRA BLVRB BLZF1 BMP1 BMP10 BMPR1B BNIP1 BOP1 BPTF BRD1 BRD2 BRD3 BRD4 BRWD1 BUB1 BUB1B C11orf48 C11orf57 C14orf139 C15orf29 C16orf42 C16orf58 C19orf6 C19orf60 C19orf73 C1orf109 C1orf163 C1orf68 C21orf2 C21orf7 C22orf29 C2orf3 C2orf55 C3 C3orf36 C3orf64 C4orf10 C4orf29 C5orf28 C7orf58 C7orf64 C9orf114 CABIN1 CABP1 CACNA1A CACNA1I CADM4 CALCRL CALD1 CALM1 CAMK2A CASD1 CASP2 CCDC101 CCDC106 CCDC134 CCDC22 CCDC28B CCDC76 CCDC93 CCL13 CCL21 CCL8 CCND2 CCNF CCNG2 CCNT2 CCR10 CCR2 CD22 CD300A CD47 CD58 CD93 CD96 CDA CDC25C CDC34 CDC37L1 CDC42 CDC42BPA CDC42EP4 CDC7 CDH11 CDH16 CDH4 CDH5 CDK10 CDK11A///CDK11B CDK17 CDK5R1 CDK7 CDKAL1 CDKN1C CDKN2C CEACAM8 CELF3 CEP135 CEP350 CEP57 CEP68 CES2 CFB CHAF1A CHD4 CHD7 CHIC2 CHM CHRNA1 CHRNE CHST3 CHST7 CIZ1 CLASP1 CLASP2 CLCN3 CLCN5 CLCN7 CLEC4E CLK1///PPIL3 CLN8 CLTA CNOT3 CNPY4 CNTF///ZFP91///ZFP91-CNTF COIL COL13A1 COL15A1 COL1A1 COL4A3 COL4A4 COL6A1 COL6A2 COL9A3 COLEC11 COMP COPS2 CORT COX7A1 CP CPEB3 CPLX3///LMAN1L CPNE7 CPOX CPS1 CPSF6 CR1 CREB5 CREBZF CREM CRH CRIP1 CRMP1 CROCC CRYBA2 CSAD CSAG2///CSAG3 CSDE1 CSF2RA CSF3R CSGALNACT1 CSNK1G1 CSNK1G2 CST4 CTAG2 CTAGE1 CTBP2 CTLA4 CTNND1 CTRB1///CTRB2 CTSE CTSL2 CUEDC1 CUL2 CUL3 CUL4B CUL5 CXCL14 CXCR3 CYB561 CYB5B CYBB CYLC1 CYorf15B CYP11B2 CYP27B1 CYP4F12 CYR61 DAPK3 DAZAP2 DBR1 DCAF15 DCAF16 DCAF17 DCAF7 DCLK2 DDN DDX17 DDX24 DDX27 DDX3X DDX46 DEFB4A DENND4A DHRS12 DHX58 DIAPH3 DICER1 DIMT1L DKFZP586I1420 DLEU2///DLEU2L DLG4 DLGAP4 DLX2 DLX6 DMPK DNAJB12 DNAJB9 DNAJC1 DNAJC6 DNAJC8 DNASE1L2 DNASE1L3 DNM3 DOCK4 DOCK9 DOHH DOK4 DOPEY1 DPPA4 DPY19L2P2 DPY19L4 DR1 DRD2 DST DTWD1 DUOX2 DUSP13 DUSP9 DUX2 DVL2 DYNC1LI2 ECM1 EDF1 EDN2 EDNRB EEA1 EED EEF1D EFEMP2 EFNA4 EFNB2 EGFL7 EHMT2 EIF3C///EIF3CL EIF5A2 EIF5B ELAC2 ELAVL2 ELF2 ELK4 EMILIN1 EMR3 ENC1 ENPP2 ENTPD1 ENTPD5 EPAG EPAS1 EPHA4 EPHB6 EPHX1 EPN1 EPO EPOR EPRS ERAP2 ERC1 ERI2 ESF1 ESR1 ETNK1 ETV4 EVC EVX1 EXD3 EXOC7 EXOSC8 F11R F12 F2 FABP2 FADS1 FAM115A FAM119B FAM134A FAM149B1 FAM153A FAM162A FAM164A FAM30A FAS FASLG FASTKD2 FAT4 FBLN1 FBN2 FBXL18 FBXO24 FBXO38 FBXO9 FCGBP FER1L4 FERMT2 FFAR2 FGA FGF6 FGF7 FGL2 FHL1 FICD FKBP8 FMO2 FN1 FNIP1///RAPGEF6 FOS FOXB1 FOXC2 FOXD4///FOXD4L1 FOXE1 FOXM1 FOXO4 FRS3 FSTL3 FUBP1 FUS FUT2 FUT7 FUT9 FXR1 GABBR1///UBD GABPB1 GAGE1///GAGE12F///GAGE12G///GAGE12I///GAGE12J///GAGE2A///GAGE2B///GAGE2C///GAGE2D///GAGE2E///GAGE3///GAGE4///GAGE5///GAGE6///GAGE7///GAGE8 GALK1 GALNT1 GART GAS7 GATA1 GATM GCDH GCFC1 GCM2 GDAP1L1 GFER GFRA2 GHR GIMAP6 GIN1 GINS3 GJA1 GK GK3P GLE1 GLG1 GLI1 GLP1R GLTPD1 GM2A GMCL1 GNAL GNAS GNAT1 GNRHR GOLGA2 GOLGA8A GOLIM4 GP1BB GP1BB///SEPT5 GP5 GPATCH2 GPC1 GPD1 GPLD1 GPR116 GPR144 GPR172A GPR63 GPR68 GRAMD4 GRAP2 GRIA2 GRIK1 GRIN1 GRK1 GRM6 GTDC1 GTSE1 GUCA2A GUF1 GZMM H2AFX HABP4 HAND2 HAUS5 HBA1///HBA2 HBB HBG1///HBG2 HBS1L HBZ HCG4P6 HDAC10///LOC100509694///MAPK12 HDAC7 HDGFRP3 HDHD1 HDLBP HEBP2 HEG1 HEPH HERC2 HFE HGF HIC1 HIC2 HIF3A HIPK1 HIST1H4C HLA-DQB2 HMG20B HMGB2 HMX1 HNRNPA0 HNRNPD HNRNPUL2 HOMER3 HOXA3 HOXD9 HPCA HSD17B10 HSD17B6 HSP90AA1 HTN1 HUS1 ICMT IDI1 IDO1 IFNA2 IFNA5 IFNAR2 IGF1 IGF1R IGF2AS IGF2BP3 IGFALS IGHA1///IGHA2///IGHD///IGHG1///IGHG3///IGHM///IGHV4-31///IGHV4-59///LOC100126583 IGHA1///IGHA2///LOC100126583 IGHG1///IGHG2///IGHM///IGHV4-31 IGK@///IGKC///IGKV1-5 IGKV1D-8 IGLL1 IGLL3P IGLV1-40///IGLV1-44 IGLV1-44///LOC100290481 IGLV2-18///IGLV3-19///LOC100290481 IGLV2-23 IGLV3-19 IKZF3 IL12B IL17B IL1R2 IL6R ILKAP ILVBL IMPACT INO80B INPP5K INSR INVS IQSEC2 IREB2 IRF5 ITGA4 ITGB1BP3 ITGB4 ITK ITPR1 ITPR3 ITSN1 KANK3 KAZ KCNA6 KCNC2 KCNJ12 KDELR2 KDM2A KDM3A KDM6A KEL KERA KHSRP KIAA0317 KIAA0913 KIAA1704 KIDINS220 KIF1C KIF2A KIF5B KIR2DL1///KIR2DL2///KIR2DL3///KIR2DL5A///KIR2DL5B///KIR2DS1///KIR2DS2///KIR2DS3///KIR2DS4///KIR2DS5///KIR3DP1///LOC727787 KIR2DL2 KIR3DX1 KLC1 KLF5 KLF8 KLHL20 KLHL7 KLK14 KRT13 KRT3 KRT8P12 KSR1 KTN1 L3MBTL1 LACTB2 LAMA2 LAMA4 LARGE LCN1 LCN2 LDB3 LDLR LECT2 LEPR LEPREL4 LGALS3 LGALS3BP LHB LIMD2 LIPE LMNA LMNB1 LMO4 LOC100127972 LOC100131532 LOC100133321 LOC100287927 LOC100290070 LOC100293553 LOC100506002 LOC100506517 LOC100507315///PPP2R5C LOC100507328///LOC100508591 LOC100509130 LOC100509749 LOC100510692///NAIP LOC150759 LOC254896 LOC254896///TNFRSF10C LOC388152///LOC727751///LOC727849///LOC80154 LOC389906 LOC441204 LOC55908 LOC644450 LOC647070 LOC729164 LOH3CR2A LOXL2 LPHN1 LRCH3 LRP1 LRP12 LRP8 LRRC31 LSG1 LSM14B LSM5 LSP1 LTK LUC7L3 LY6E MAGEB4 MAGOH2 MALT1 MAMLD1 MAP2K3 MAP2K7 MAP4K4 MAPK11 MAPK8IP1 MAPK8IP3 MAPT MARS MATK MBD3 MBIP MBL2 MBNL2 MBP MC1R MCM9 MDM4 ME3 MEAF6 MECP2 MED13L MED6 MED7 MEF2C MEF2D METTL4 METTL5 MFAP3L MFGE8 MFNG MGAT2 MINK1 MKNK2 MLL MLL4 MLLT1 MLLT10 MLLT4 MLXIPL MMP10 MMP12 MPHOSPH9 MPL MPP5 MRPL28 MRPS18A MRPS18B MST4 MSTN MTA2 MTAP MTDH MTF2 MTFR1 MTHFR MTM1 MTMR8 MTO1 MTRR MTX2 MUC13 MUC5B MXD4 MYCBP2 MYO1C MYO6 MYO7A MYOT N4BP2L1 NAA16 NAP1L3 NARFL NAT6 NCAPG2 NCF1///NCF1B///NCF1C NCOA2 NCOR2 NDUFA13 NDUFA4 NDUFA9 NEK1 NETO2 NFKB2 NFKBIL2 NFYA NFYC NGB NGF NHLRC2 NIPAL3 NIPSNAP3B NKTR NKX3-1 NLRP3 NMBR NMT2 NNAT NOP2 NOP56 NPAS1 NPAT NPEPPS NPRL3 NPY1R NR0B1 NR2C1 NR2E3 NR4A2 NRG1 NRP2 NRXN1 NRXN2 NSUN7 NTRK3 NUCKS1 NUFIP1 OBP2A///OBP2B OGFR OGT OIP5 OLFM4 OLIG2 OPA1 OR12D3///OR5V1 OR2B2 OR7A5 ORC2 ORC4 OSBPL10 OSBPL2 OTUD3 P2RY14 PA2G4 PABPN1 PAGE1 PAK2 PAQR3 PAQR5 PARG PAX4 PBX2 PCBP2 PCDH1 PCDHB11 PCDHGA10///PCDHGA11///PCDHGA12///PCDHGA3///PCDHGA5///PCDHGA6 PCDHGA3 PCGF2 PCIF1 PCLO PCSK2 PDCL PDE3B PDE4A PDE4B PDE8B PDGFB PDGFRB PDIA2 PDLIM1 PDLIM5 PDLIM7 PDPK1 PDZK1IP1 PER2 PEX10 PFKFB3 PFKL PGAM2 PGLYRP1 PHF16 PHF20 PHLDA1 PHLDB1 PHTF1 PIK3R1 PIM1 PIN1P1 PIN4 PITPNA PKP4 PLA2G7 PLA2R1 PLD2 PLEC PLEKHG3 PLK4 PLN PLUNC PLXNB2 PLXNB3 PLXNC1 PMM2 PNMA3 PNN PNPLA2 POFUT2 POLG POLR2D POMC PON1 POT1 POU3F1 PPAN PPBP PPDPF PPFIBP1 PPIEL PPIG PPIL6 PPP1R10 PPP2CB PPP2R1B PPP2R5A PPP2R5B PPP6R1 PQBP1 PRDM13 PRDM8 PREPL PRF1 PRICKLE3 PRKAA1 PRKCB PRKCI PRKRIP1 PRKX PRLH PROC PRPF40A PRUNE PSIP1 PSPH PSTPIP2 PTEN PTENP1 PTGES PTHLH PTMS PTPN21 PTPRF PUS3 PVRL3 QARS QRSL1 RAB11B RAB28 RAB3GAP2 RAB6A RABEP1 RAD51L3 RAE1 RAI1 RALGAPA1 RANBP3 RARB RARRES1 RASGRP2 RASSF2 RBM10 RBM25 RBM26 RBM34 RBM9 RC3H2 RCC1 RCE1 RECK RECQL5 REL REV3L RFC1 RFWD3 RGS11 RGS13 RGS6 RGS7 RHD RHEB RHOBTB2 RHOBTB3 RNF126 RNF43 RNF8 RNMT RPAP3 RPL14 RPL36 RPL5 RPLP0 RPLP2 RPP21///TRIM39///TRIM39R RPRD1A RPS14P3 RPS20 RPS6KA4 RPS6KB1 RPS6KB2 RRBP1 RRN3///RRN3P1///RRN3P2 RRP15 RRP9 RSAD2 RTCD1 RTEL1 RUFY3 RUNX1 RXRB RYR2 S100A6 S100A8 SAA1///SAA2 SAFB SAFB2 SAMD14 SAR1A SARDH SBF1 SC5DL SCAMP1 SCAPER SCARF1 SCGB1D1 SCN1A SCN2B SCN3B SCNN1A SCRN3 SCT SDCCAG3 SDHAF1 SEC14L1 SECISBP2L SELPLG SEMA3B SEMA6A SEMA6C SENP6 SEPT9 SERPINB4 SERPINB9 SESN1 SETDB1 SFRS18 SFSWAP SFTPA2 SFTPB SFTPC SGSH SH2D1A SH3GL3 SIGLEC8 SIK3 SIRT1 SLAMF1 SLC12A4 SLC12A9 SLC13A2 SLC16A1 SLC16A3 SLC16A7 SLC17A6 SLC1A6 SLC20A1 SLC22A6 SLC25A17 SLC25A36 SLC26A1 SLC26A2 SLC26A4 SLC30A10 SLC35A5 SLC35D1 SLC39A8 SLC39A9 SLC48A1 SLC4A5 SLC4A7 SLC5A3 SLC5A4 SLC6A7 SLC7A6 SLC9A3R2 SLCO1A2 SMAD4 SMAD6 SMARCA2 SMARCA4 SMARCB1 SMC3 SMCHD1 SMYD5 SNAP25 SNAPC3 SNRNP70 SNRPC SNRPD2 SNTB1 SNTB2 SOCS3 SOCS5 SOCS7 SOHLH2 SORBS1 SORD SOX13 SOX4 SP100 SP2 SPARC SPAST SPATA5L1 SPG7 SPINK1 SPP1 SPPL2B SPRED2 SPRR3 SPSB3 SQSTM1 SR140 SRC SRCAP SRP19///ZRSR1 SRPK2 SRPX SRRM2 SRSF10 SRSF2IP SSTR3 SSTR5 SSX4///SSX4B ST14 ST3GAL2 ST6GALNAC4 ST6GALNAC5 STAG1 STAT5B STATH STK11 SUGP2 SUPT6H SYCE1L SYMPK SYNJ2 SYNM SYNPO SYT1 TACO1 TACR1 TAF1 TAOK2 TAP2 TAPBPL TAS2R16 TBL1X TBX19 TCF15 TCF25 TCF3 TCF7L2 TCL6 TCN1 TCOF1 TDO2 TEF TESC TEX11 TEX264 TF TFPI TGFB1I1 TGFBR1 THAP1 THPO TIAM2 TICAM1 TLE1 TLE4 TLN2 TLX1 TM7SF3 TMCO3 TMEFF1 TMEM110 TMEM144 TMEM158 TMEM214 TMEM35 TMEM45A TMEM63A TMEM97 TMPO TNFSF10 TNIK TNK1 TNPO1 TNPO2 TNS1 TOB2 TOE1 TOMM40 TOP1 TOP2A TOP3A TOP3B TP53AIP1 TP53BP2 TP53I11 TPM2 TPMT TPPP TPR TPSAB1 TPSD1 TPTE TRA2A TRAF3IP3 TRAF5 TRAPPC2 TRAT1 TRD@ TRGV5 TRIM2 TRIM31 TRIOBP TRIP6 TRPC4 TRPV4 TRPV6 TSC22D4 TSKS TSR1 TST TTC15 TTC22 TTC3 TTC37 TTC38 TTC39A TTF1 TTK TTLL3 TTLL5 TUBB2A TUBGCP2 TWF1 TXN UBAP1 UBE2D1 UBE2D2 UBE2G2 UBE3A UBN1 UBR2 UCHL5 UCN UCP3 UGCG UHRF1BP1L ULK1 ULK2 UNC45A UNC93B1 UQCR11 UQCRQ UROD USP5 VAMP2 VCAN VCPIP1 VCX2 VDAC1 VGLL4 VIL1 VPS13A VPS13D VSIG10 VWA1 WAS WDR55 WDR59 WDR76 WDR8 WIPI2 WISP2 WNK1 WNT1 WNT6 XDH XIAP XPNPEP1 XPNPEP2 XYLT1 YAF2 YIPF4 ZAP70 ZBTB16 ZBTB17 ZBTB22 ZBTB38 ZBTB43 ZBTB7A ZEB1 ZER1 ZFP36L1 ZFPL1 ZKSCAN1 ZKSCAN4 ZKSCAN5 ZNF107 ZNF117 ZNF148 ZNF167 ZNF200 ZNF205 ZNF238 ZNF24 ZNF271 ZNF280D ZNF331 ZNF335 ZNF350 ZNF493 ZNF518A ZNF529 ZNF571 ZNF576 ZNF646 ZNF671 ZNF695 ZNF702P ZNF770 ZNF783 ZNF787 ZNF91 ZSCAN18 ZXDB | BAL_46_A2B0 BAL_27b_A2B1 BAL_26_A2B0 BAL_45b_A2B0 BAL_13_A0B0 BAL_21_A0B0 BAL_40_A0B0 BAL_45a_A1B0 BAL_17_A0B0 BAL_39_A0B1 BAL_20a_A1B1 BAL_4_A0B0 BAL_44b_A0B0 BAL_47b_A0B1 BAL_25_A0B0 BAL_3_A1B0 BAL_42_A0B0 BAL_12_A1B0 BAL_18_A1B0 BAL_37_A1B1 CurrentSmoker_1 BAL_33_A0B1 BAL_20b_A1B1 BAL_43_A0B0 BAL_15a_A0B1 BAL_29_A1B0 BAL_47a_A0B0 BAL_27a_A0B0 BAL_1_A0B0 BAL_47c_A0B1 BAL_19_A0B0 |
| **4** | AAK1 AARS ABAT ABCA1 ABCC10 ABCC8 ABCD2 ABHD6 ABL1 ACACB ACIN1 ACSF2 ACTA2 ACTL6B ACTR3 ADAM18 ADAM5P ADAM8 ADAMDEC1 ADCY3 ADM ADNP2 AHI1 AHNAK2 AHSA1 AIFM1 AK1 AK5 AKAP11 AKAP12 AKAP8L AKAP9 AKR1B10 ALDH1L1 ALDH4A1 ALDH5A1 ALG13 ALG5 ALOXE3 ALPL ANAPC2 ANAPC5 ANKFY1 ANKRD12 ANKRD6 ANXA3 AP1G2 AP1S1 AP2A2 AP3M2 APC APH1A APOBEC3A APPBP2 APPL2 APRT AQP4 AQP9 ARFGAP1 ARFGAP2 ARFIP1 ARHGAP26 ARHGAP4 ARIH2 ARL1 ARL17A///ARL17B ARL2 ARL6IP4 ARSA ASB9 ASCC1 ASMTL ASNS ASPSCR1 ATG2B ATG4B ATP11B ATP1B1 ATP2A2 ATP5C1 ATP5D ATP5J ATP6V0A1 ATP6V0A2 ATP6V0E1 ATP6V1C1 ATP6V1G1 ATP6V1G2///BAT1 ATP8A1 ATP8B1 AURKA B2M B3GAT3 B4GALT1 B4GALT5 BACH1 BAI2 BANK1 BAT3 BBS7 BCAM BCAT2 BCL3 BIRC3 BMS1 BRD1 BRD8 BRD9 BRIP1 BRWD1 BSCL2 BTG1 C10orf2 C10orf79 C11orf2 C11orf24 C12orf35 C14orf101 C14orf135 C14orf93 C15orf29 C16orf42 C17orf101 C17orf39 C17orf85 C19orf22 C19orf29 C19orf42 C19orf54 C19orf6 C19orf60 C1orf107 C1orf156 C1orf54 C1orf63 C20orf103 C20orf117 C20orf24 C21orf91 C2CD2 C2CD2L C2orf43 C3orf64 C5orf4 C6orf103 C6orf130 C6orf26///MSH5 C8orf84 C9orf114 C9orf167 C9orf53 C9orf95 CABIN1 CADM4 CALCRL CALM1 CALR CAPN3 CAPRIN2 CAPZA1 CASP2 CASP4 CASP5 CASP6 CATR1 CBR4 CBX7 CCDC130 CCDC144A CCDC51 CCDC88A CCDC91 CCL2 CCL20 CCL3///CCL3L1///CCL3L3 CCL4 CCT3 CCT8L2 CD180 CD2 CD47 CD48 CD53 CD55 CD58 CD59 CD93 CD99 CDC42BPB CDC42EP2 CDC42SE1 CDC5L CDH11 CDK5R1 CDK5RAP3 CDT1 CEACAM1 CEACAM8 CENPF CENPM CENPT CEP135 CEP76 CES1 CES1P1 CFLAR CHI3L1 CHIT1 CHKB-CPT1B///CPT1B CHMP1B CHP CHRNA2 CHRNE CHST15 CHST3 CIRBP CIT CKS2 CLASP1 CLC CLCC1 CLEC10A CLK4 CLU CMTM6 CNOT3 CNPY2 CNR1 CNTNAP2 COBLL1 COG4 COG7 COL16A1 COL3A1 COL4A3 COL4A6 COL5A3 COL6A1 COL7A1 COLQ COPG COQ7 CORO1A COX5B COX7B CPD CPSF1 CPSF3L CPT1A CR1 CREBZF CREM CRK CROCCP2 CROT CRYZL1 CSF2RB CSF3R CSNK1E CSNK1G2 CST1 CTDP1 CUL3 CUTA CWC25 CXCL1 CXCL10 CXCL11 CXCL14 CXCL9 CXCR1 CXCR2 CXorf57 CYB5B CYP1B1 CYTH1 DAXX DAZ1///DAZ2///DAZ3///DAZ4 DCAF10 DCAF16 DCAF8 DCUN1D4 DDB2 DDHD2 DDI2 DDRGK1 DDT DDX17 DDX19A DDX24 DDX27 DDX3X DDX54 DDX56 DEFA1///DEFA1B///DEFA3 DEFB1 DEFB4A DENND1A DENND4A DERL2 DGCR8 DGKG DGKZ DHRS4///DHRS4L2 DHRS7 DHX16 DHX30 DHX34 DHX35 DICER1 DIS3 DLAT DLEC1 DLGAP1 DMXL1 DNAI2 DNAJB9 DNASE2 DOK5 DOPEY1///LOC100509911 DPAGT1 DPM1 DROSHA DSCC1 DSTYK DTX3 DUOX2 DUSP6 DVL1 DYNC1H1 DYNC1LI2 DYSF ECHDC2 EDC4 EEF1D EEF2 EFEMP2 EFR3B EGR3 EHD1 EHHADH EIF2S1 EIF3G EIF4B EIF4G2 EIF5A EML3 EMP1 EMR2 ENO2 ENOSF1 ENPP2 EP400 EPAS1 ERCC1 ERG ERGIC3 ESR1 ESRRB EXOC7 EXOSC10 F5 F9 FABP6 FAHD2A FAM106A FAM111A FAM129A FAM13A FAM149B1 FAM160B2 FAM178A FAM193A FAM193B FAM3A FAM65B FAN1 FASTK FAU FBL FBXL15 FBXL18 FBXL5 FBXO22 FBXW12 FCAR FCGR2B FCN1 FECH FETUB FFAR2 FGF12 FGF20 FGF21 FGFR3 FGL2 FIS1 FKBP10 FKBP15 FLJ14107 FN1 FOLR1 FOSL2 FOXF1 FPR1 FRMD4B FRY FTH1 FTH1P5 FTL FTO FTSJ3 FUBP1 FUS FXYD2 FZD9 G0S2 GABARAPL2 GABBR1///UBD GAPVD1 GATAD1 GATC GBP1 GBX2 GCH1 GCM1 GDAP2 GEMIN4 GFAP GGA1 GGA3 GH1 GLG1 GLRX3 GLTSCR2 GLYR1 GM2A GNA11 GNAO1 GNB2L1 GNG10 GNG13 GNL3L GOLGA1 GOLGA2 GOLGA2B GOLGA6L5///GOLGA6L9 GOLGA8A GOLGA8C///GOLGA8DP///GOLGA8E///GOLGA8G///LOC653061 GOLGA8H GOLIM4 GOSR1 GPM6A GPR157 GPR44 GPR89A///GPR89B///GPR89C GPR97 GPRASP1 GPX4 GRAMD4 GRB10 GSTT2 GTF2F1 GTF2F2 GTF2H1 GTF2I GUCY1A3 GUCY1B3 GZMB H1FX H3F3B HAP1 HAUS6 HAVCR1 HCFC2 HDAC3 HDGFRP3 HERC2 HEXIM1 HFE HIC2 HIF1A HIST1H1A HIST1H2AL HIST1H3C HIST1H3E HIST1H4C HIST2H2AA3 HLA-DPA1 HMG20A HMG20B HMGN3 HNRNPA1 HNRNPD HNRNPH1 HNRNPL HNRNPM HPGD HPS4 HSD17B7 HSF1 HSP90AB1 HSP90B1 HSPA2 HTATIP2 HTRA2 ICAM1 ICAM3 ICAM4 IDH3B IDO1 IDS IFI16 IFITM1 IFITM2 IFT122 IGF1 IGF2AS IGHA1///IGHD///IGHG1///IGHG3///IGHM///IGHV3-48///IGHV4-31///LOC100291917 IGHG1///IGHM///LOC100133862 IGKC///IGKV1-5///IGKV1D-8///LOC652493///LOC652694 IGLL5///IGLV2-11 IGLV2-18///IGLV3-19///LOC100290481 IGLV3-19 IK///TMCO6 IL11RA IL17RA IL18RAP IL19 IL1B IL1R2 IL1RL2 IL1RN IL8 ILF3 IMPACT IMPDH2 INTS3 INVS IPO5 IQCE IRF3 ISG20 ISLR ITGA4 ITGB5 ITK ITM2A IVD KAL1 KAT2A KAT5 KATNB1 KCNH2 KCNJ13 KCNJ15 KCNJ2 KCTD13 KCTD2 KCTD7 KHDRBS1 KIAA0101 KIAA0146 KIAA0195 KIAA0495 KIAA1009 KIAA1109 KIR2DL3 KIR2DL4///KIR2DL5A///LOC100287534 KL KLF6 KLHL36 KLRAP1 KLRD1 KRAS KRT13 KRT15 KYNU LAP3 LARGE LARP4 LARS LAX1 LDB3 LDHB LEPR LGALS3BP LGALS8 LGSN LHX3 LILRA6///LILRB3 LILRB3 LIMK2 LIMS2 LIPE LIPF LMBR1L LMNB1 LOC100128640 LOC100132247///LOC348162///LOC613037///LOC728888///NPIPL3 LOC100505523 LOC100506168///SFPQ LOC100506517 LOC100506653 LOC100507328 LOC100507424 LOC100510626///S100A13 LOC150776///SMPD4 LOC157627 LOC339290 LOC729020///RPE LOC729164 LOC729991 LONP1 LONP2 LPCAT1 LRFN4 LRMP LRP1B LRRFIP1 LTB LTB4R LTBP3 LUC7L LUC7L3 LY6D LYN LYPLA2 LYRM4 MACF1 MAFF MAGEA11 MAGT1 MALT1 MAN1C1 MAP1A MAP2K2 MAP2K7 MARCKS MARCKSL1 MAST2 MATN1 MAU2 MBD3 MBP MBTPS1 MCL1 MCTP2 MDC1 MDFIC MDN1 ME1 ME3 MED16 MED21 MED6 MED9 METRN METT11D1 METTL7A MFAP3L MFSD10 MGAM MGC12488 MGLL MGRN1 MICAL2 MID1 MID1IP1 MIOS MKI67 MKL2 MLL4 MLPH MMADHC MMP12 MMP3 MMP9 MORC2 MPHOSPH8 MPI MRAS MRPL23 MRPL44 MRPS2 MRPS34 MTAP MTHFD2 MTO1 MTRF1L MUC5AC MUS81 MUTYH MX2 MXD1 MYH1 MYO15B MYO1E MYST1 MZF1 MZT2A NAA16 NADK NAMPT NASP NAT10 NBAS NBN NCOA4 NCOR2 NCRNA00171 NDRG1 NDST1 NDUFB7 NDUFB8 NDUFS7 NDUFV1 NEAT1 NEDD9 NEK3 NEU3 NF1 NF2 NFATC1 NFATC2IP NFATC3 NGRN NISCH NIT1 NLE1 NLRP3 NMT1 NNMT NNT NOLC1 NOV NPEPL1 NPHP4 NPHS1 NPIPL3 NPRL2 NPRL3 NPTXR NPY5R NR2C1 NR2F1 NRXN3 NSUN5 NSUN5P1 NSUN5P2 NT5C2 NTRK2 NUCKS1 NUDC NUDCD3 NUDT13 NUMA1 NUP133 NUP43 NUP50 NUP62 NUPR1 NXF2 OAZ1 OGFOD2 OGFR OGT OMD OPA1 OR2F1///OR2F2 OR2J2 ORC5 ORM1 ORM1///ORM2 OS9 OSBPL8 OSM OTUD3 OTUD4 OXTR P2RY14 P4HA2 PA2G4 PABPN1 PACS2 PADI2 PAH PAN2 PAPOLA PAQR3 PASK PAX3 PCDH9 PCDHGA8 PCM1 PCSK5 PDCD11 PDCL PDE4B PDE4D PDE6D PDK2 PDK3 PDLIM5 PDLIM7 PDPR PDX1 PDZK1IP1 PDZRN4 PEBP1 PELI1 PEX14 PFAS PFDN5 PFKFB3 PGAP1 PGD PGF PGK1 PGLS PGM3 PHF14 PHF15 PHF20 PHGDH PHKA2 PHLDA1 PHLDA2 PI3 PI4KB PIAS2 PICALM PIGQ PIGT PIH1D1 PIK3CD PIK3R1 PILRB PIP4K2A PITPNM1 PKD1P1 PKNOX2 PLA2G5 PLCG1 PLEK PLEKHO1 PLGLB1///PLGLB2 PLK3 PLN PLXNA1 PLXNA2 PLXNB2 PLXNC1 PMPCB PMS2///PMS2CL PMS2P1 PMS2P3 PNN PNO1 PNRC1 POFUT2 POGZ POLB POLI POLR2L POLRMT POP5 POR POTEKP POU3F4 PPA1 PPBP PPFIBP2 PPIA PPIF PPP1R15A PPP1R3A PPP1R7 PPP2R3A PQBP1 PRB1 PRB3 PRDX4 PRDX6 PRIM2 PRKAA1 PRKAR1A PRKCSH PRKD2 PRKD3 PRKRIP1 PRKX PROS1 PRPF8 PRPS1 PRR11 PSIP1 PSMA3 PSMB4 PSMD2 PTGES PTGFR PTGS2 PTOV1 PTP4A2 PTPN2 PTPN22 PTPRR PTTG2 QRSL1 QTRT1 RAB11FIP1 RAB2A RAB4B RAB5A RABEP2 RABGAP1 RAD1 RALGAPA1 RALGDS RANBP1 RANBP10 RANBP6 RAPGEF2 RARA RARB RARRES1 RASGRP1 RASSF2 RBCK1 RBL2 RBM10 RBM14 RBM25 RBM26 RBM42 RBM5 RBM6 RC3H2 RDBP REG1P RFNG RFX7 RFXAP RGL2 RGS12 RGS2 RHOBTB1 RHOH RIF1 RIPK2 RMND5B RNF125 RNF24 RNFT1 ROGDI RPAIN RPE65 RPGRIP1L RPL10 RPL13 RPL13A RPL14 RPL27A RPL29 RPL3 RPL38 RPL4 RPL7A RPLP2 RPP30 RPS11 RPS15 RPS3 RPS5 RPS6 RPS6KA2 RPS9 RRBP1 RREB1 RSF1 RSL1D1 RTCD1 RTN2 RUFY3 RUNX1 RUNX1T1 RUNX3 RYBP RYR2 RYR3 S100A12 S100A8 S100A9 SAP18 SART3 SATB2 SBF1 SBNO2 SCAND1 SCGB1A1 SCML1 SCN11A SCN5A SCO2 SDC4 SDCBP SEC14L1 SEC14L3 SEC61G SECTM1 SELL SEMA3B SENP3 SEPHS1 SEPHS2 SEPT4 SEPX1 SERPINB1 SERPINB6 SERPINB9 SERPINI2 SET SETD4 SETMAR SF3A2 SF3B2 SFI1 SFRS15 SFRS18 SFSWAP SFTPB SFXN3 SGSM2 SHMT1 SHMT2 SIPA1 SIVA1 SKIV2L SKP2 SLAMF7 SLC13A1 SLC18A1 SLC19A2 SLC22A3 SLC22A8 SLC23A2 SLC25A1 SLC25A31 SLC25A37 SLC25A4 SLC26A2 SLC26A4 SLC2A3 SLC2A4RG SLC2A5 SLC2A6 SLC35E1 SLC38A10 SLC39A8 SLC4A8 SLC6A16 SLC7A11 SLC9A7 SLCO1B3 SLCO3A1 SMARCA2 SMUG1 SMYD2 SNN SNRNP200 SNRNP40 SNRNP70 SNRPG SNRPN///SNURF SNX19 SNX27 SOBP SOCS5 SOD2 SON SORL1 SOX1 SP100 SPATA2 SPATA6 SPG7 SPN SPP1 SPRED2 SPRR2B SPRR3 SPRY4 SPTAN1 SPTLC2 SQLE SQRDL SQSTM1 SRC SRGN SRP54 SRPK1 SRRD SRRT SRSF10 SRSF11 SRSF5 SRSF6 SSRP1 ST20 ST3GAL1 ST3GAL4 STAG1 STARD13 STAT3 STATH STC1 STEAP3 STIP1 STK11 STOML2 STX16 STX3 STX6 SUB1 SUCLG2 SUGP2 SULT1A1 SULT1A3///SULT1A4 SULT1B1 SUMO1 SUPT3H SUPT7L SUSD5 SUZ12P SYCP1 SYMPK SYNGR3 TAF1D TAOK1 TAP1 TAP2 TAPT1 TARDBP TARS2 TBCD TBL1X TCF25 TCF3 TCF7 TCN1 TDP2 TECR TEP1 TERF1 TFCP2 THAP9 THOC6 THUMPD2 THY1 TICAM1 TIMM22 TIMM44 TIMM50 TK2 TLR2 TMCC1 TMEFF1 TMEM115 TMEM120B TMEM160 TMEM186 TMEM222 TMEM38B TMEM39B TMSB4X///TMSL3 TNF TNFAIP6 TNFRSF10C TNFRSF1B TNFRSF25 TNIP1 TNNI3 TNS1 TP53BP2 TP53I11 TPR TRADD TRAFD1 TRAK2 TRAP1 TRAPPC2L TRAV8-3 TRD@ TRDMT1 TRIM22 TRIM25 TRIM9 TRIO TRMT1 TROVE2 TRPC1 TSC22D2 TSFM TTC23 TTC28 TTC3 TTLL3 TTLL5 TUBA1B TUBD1 TUBGCP2 TUBGCP4 TUFM TULP2 TULP4 TXN TXNDC9 TXNRD1 TYMP UBA7 UBAC1 UBE2G2 UBFD1 UBR5 UCHL3 UCK2 UCKL1 UPB1 UPF3A UQCRC1 URB1 UROD USP21 USP22 USP34 USP4 USP48 USP6NL VAMP1 VAMP2 VANGL1 VAPB VARS VAX2 VCL VCP VDAC3 VEZF1 VEZT VGLL1 VGLL3 VNN2 VPS13B VPS28 VPS33B WARS WBP4 WBP5 WDHD1 WDR13 WDR18 WDR46 WNT2B WNT4 WNT5B WSB1 WTAP XIST XYLB YOD1 YRDC YWHAE YWHAZ YY2 ZBTB20 ZBTB7A ZC3H12A ZC3HAV1 ZCCHC14 ZDHHC17 ZFR ZIM2 ZMIZ2 ZMYM2 ZMYND11 ZMYND8 ZNF137P ZNF16 ZNF160 ZNF177 ZNF200 ZNF224 ZNF225 ZNF23 ZNF24 ZNF253 ZNF267 ZNF271 ZNF280D ZNF287 ZNF292 ZNF358 ZNF407 ZNF410 ZNF44 ZNF544 ZNF551 ZNF613 ZNF673 ZNF701 ZNF747 ZNF780A///ZNF780B ZNF83 ZSCAN18 ZW10 ZWINT ZXDA///ZXDB ZXDB | BAL_42_A0B0 BAL_19_A0B0 BAL_20a_A1B1 BAL_39_A0B1 BAL_44a_A0B0 BAL_20b_A1B1 BAL_46_A2B0 BAL_21_A0B0 BAL_43_A0B0 BAL_18_A1B0 BAL_29_A1B0 BAL_47b_A0B1 BAL_27b_A2B1 BAL_4_A0B0 BAL_37_A1B1 BAL_33_A0B1 BAL_12_A1B0 BAL_47a_A0B0 BAL_25_A0B0 BAL_3_A1B0 BAL_47c_A0B1 BAL_45b_A2B0 BAL_27a_A0B0 BAL_40_A0B0 BAL_15b_A0B0 BAL_45a_A1B0 BAL_13_A0B0 BAL_31_A0B1 BAL_1_A0B0 BAL_17_A0B0 BAL_15a_A0B1 BAL_44b_A0B0 BAL_26_A2B0 BAL_41_A1B0 FormerSmoker_69 CurrentSmoker_83 NeverSmoker_78 FormerSmoker_114 |
| **5** | AAK1 ABAT ABCA3 ABCB1 ABCB1///ABCB4 ABCB6 ABCC6 ABCD4 ACAA1 ACAD8 ACCN1 ACD ACHE ACO1 ACO2 ACOT7 ACOX3 ACRV1 ACSM3 ACSM5 ACTA2 ACTB ACTC1 ACTG1 ACTN2 ACTN3 ACTR3B ACVR2A ACVRL1 ADA ADAM18 ADAM2 ADAM21///ADAM21P1 ADAM23 ADAM28 ADAM3A ADAM9 ADAMTS3 ADAMTS7 ADARB1 ADCY10 ADCY2 ADCY9 ADD3 ADH7 ADRA1A ADRA2A ADRB2 ADRB3 ADRBK1 AEN AFF2 AFP AGAP1 AGER AGFG1 AGPAT1 AGPAT4 AGRN AGXT AHCY AHCYL2 AHI1 AKAP8L AKR1B10 AKR1C1 AKR1C2 AKR1C3 AKR1D1 AKR7A2 AKR7A3 ALAD ALDH1B1 ALDH3A1 ALDH3A2 ALDH4A1 ALDH5A1 ALDH6A1 ALDOAP2 ALDOB ALK ALKBH1 ALMS1 ALOX12B ALPK1 AMPD2 AMPH AMY1A///AMY1B///AMY1C///AMY2A///AMY2B ANAPC13 ANAPC5 ANG ANKRD1 ANKRD11 ANKRD12 ANKRD36B ANKRD53 ANO3 ANP32A///ANP32D ANTXR1 ANXA10 ANXA11 ANXA13 AOAH AOC2 AP1S1 AP2A2 AP2B1 AP3M2 APBA3 APBB2 APOBEC3B APOBEC3F APOBEC3F///APOBEC3G APOE APOF APOL1 APOL2 APOOL APP AQP1 AQP4 ARAP1 ARF4 ARF6 ARFGEF1 ARFGEF2 ARG2 ARHGAP1 ARHGAP11A ARHGAP12 ARHGAP15 ARHGAP25 ARHGAP26 ARHGEF12 ARHGEF2 ARHGEF7 ARID5A ARL14 ARL5A ARMC9 ASCC2 ASGR1 ASGR2 ASMT ASNS ASPH ASPSCR1 ASTE1 ASTN2 ATF3 ATF5 ATF6B ATF7IP ATF7IP2 ATG2A ATG4A ATP13A3 ATP1A2 ATP1A3 ATP1B1 ATP2A3 ATP2B2 ATP2B3 ATP5G2 ATP5J ATP6V0A1 ATP6V0A2 ATP6V1G2///BAT1 ATP8A2 ATP8B1 ATRX ATXN2L AVIL AVL9 AVP AZGP1 B3GALNT1 B4GALT3 B4GALT7 BAGE BAI3 BAIAP2L2 BAMBI BAT2L2 BATF BATF3 BAX BAZ1B BAZ2A BCAN BCAP29 BCL3 BCL7A BECN1 BEGAIN BEST1 BFSP1 BGN BHMT BICC1 BLNK BMP7 BMP8A BNIP1 BPNT1 BRD2 BRS3 BRSK2 BTBD3 BTBD7 BTC BTF3 BTF3P11 BTG2 BTK BTN2A1 BTN2A2 BTN3A1 BTNL8 BUB1 BYSL C10orf118 C10orf68 C10orf84 C11orf1 C11orf24 C11orf9 C12orf10 C12orf11 C13orf1 C14orf104 C14orf105 C14orf139 C14orf147 C14orf56 C16orf5 C16orf68 C17orf68 C17orf80 C17orf86 C17orf91 C19orf26 C19orf29 C19orf42 C19orf6 C1orf105 C1orf112 C1orf116 C1orf144 C1orf163 C1orf175 C1orf216 C1orf61 C1orf66 C20orf103 C20orf12 C21orf59 C2orf68 C3 C3orf51 C4BPA C4orf31 C5orf15 C5orf28 C5orf54 C6orf103 C6orf108 C6orf130 C6orf162 C6orf48 C6orf54 C6orf62 C7orf28B C7orf28B///CCZ1 C7orf43 C7orf44 C8B C8orf39 C9orf95 CA12 CA5BP CACNA1A CACNA1C CACNA1G CACNA1S CACYBP CADM3 CADM4 CALB1 CALCA CALD1 CALM1 CALML3 CALML5 CAMK2B CAMSAP1 CAND1 CAPN10 CASP10 CASQ1 CATR1 CAV1 CBX4 CC2D1A CCDC41 CCDC48 CCL11 CCL20 CCL25 CCL3///CCL3L1///CCL3L3 CCL4 CCL5 CCL7 CCNB1IP1 CCNT2 CCR2 CCR3 CCRL1 CD200 CD2BP2 CD3G CD55 CD6 CD80 CDA CDADC1 CDC42 CDC42BPA CDC42EP3 CDC42SE1 CDC5L CDC6 CDCA3 CDCA4 CDK1 CDK10 CDK13 CDK14 CDK5R1 CDKL5 CDKN1A CDR1 CDRT1 CDYL CEACAM1 CEACAM8 CELF3 CELSR1 CENPB CENPC1 CEP152 CEP164 CEP290 CEP57 CEP68 CGB///CGB5///CGB7 CHAF1B CHCHD7 CHD7 CHD8 CHI3L1 CHKB CHKB-CPT1B///CPT1B CHMP2A CHN2 CHRM2 CHRNA1 CHRNA3 CHST1 CHST15 CHST7 CIAPIN1 CIITA CINP CKAP4 CLCA2 CLCF1 CLCN1 CLDN5 CLEC11A CLEC4M CLIC3 CLPP CLSTN2 CLUL1 CNGA3 CNGB1 CNOT8 CNTLN CNTNAP2 COL11A2 COL1A1 COL1A2 COL21A1 COL2A1 COL4A1 COL4A3 COL6A1 COL6A2 COL8A1 COMMD3 COMMD4 COPB1 COPS8 COPZ2 COQ6 CORO7 COX11 COX5B COX8A CPEB1 CPSF7 CPT1A CRAT CRCP CREB5 CREBZF CRH CRHR1 CRIM1 CRIPT CRISP3 CRK CROCCP2 CROCCP3 CROT CRY1 CRYBA2 CSAD CSAG2///CSAG3 CSF1 CSF2RA CSF3 CSGALNACT1 CSHL1 CSNK1A1 CSNK2A1 CSPP1 CST5 CSTF2T CTAG1A///CTAG1B CTBP1 CTBP2 CTSW CTSZ CUL3 CUL4B CWH43 CXCL14 CXCL2 CXCL3 CXCL9 CXCR6 CXorf57 CYB5A CYLC1 CYLD CYP1B1 CYP2C8 CYP2E1 CYP2R1 CYP2U1 CYP51A1 CYR61 CYTH1 DAPK2 DAPP1 DAXX DAZ1///DAZ2///DAZ3///DAZ4 DBT DCAF13 DCAF15 DCAF16 DCAKD DCHS1 DCN DCTD DCTN4 DCUN1D4 DCX DDB1 DDIT4 DDO DDX11 DDX17 DDX19A DDX19A///DDX19B DDX27 DDX49 DEFB4A DEPDC5 DGCR2 DGCR6///DGCR6L DGKQ DHFR DHRS7 DHRS7B DHX30 DHX8 DIABLO DIDO1 DIP2A DISC1///TSNAX-DISC1 DIXDC1 DKK1 DKK3 DLGAP2 DLGAP4 DLGAP5 DLX6 DMD DMPK DMRT1 DNAJB6///TMEM135 DNAJC2 DNASE1L3 DNPEP DNTT DOCK6 DOCK9 DOK1 DOPEY1 DOPEY2 DPAGT1 DPH2 DPM3 DPP3 DPP4 DPP6 DPY19L1 DPY19L2P2 DPYSL3 DR1 DRD3 DSG1 DSPP DST DSTYK DTNB DUSP1 DUSP2 DUSP5 DUX1///DUX3///DUX5 DVL2 DYRK1A DYRK2 ECHDC3 EDA EDDM3B EDN1 EDNRA EDNRB EEA1 EEF1D EFCAB2 EFHC2 EGF EGR1 EGR3 EHMT2 EID1 EIF1 EIF2AK1 EIF2B4 EIF2B5 EIF2C2 EIF3C///EIF3CL EIF4A1 EIF4E2 EIF4G2 EIF5 EIF5B ELAVL2 ELF2 ELK1 ELK3 ELK4 ELL ELTD1 EML1 ENDOD1 ENO3 ENPEP ENTPD1 ENTPD4 ENTPD6 EP300 EPAG EPAS1 EPB41 EPHA3 EPHB2 EPHB6 EPM2AIP1 EPOR ERC1 ERG ERLIN2 ERO1LB ERP44 ESF1 ESM1 ESPN ESR1 ETFB ETS2 ETV5 EXOG EXOSC9 EXT1 EZH1 EZH2 EZR F2RL1 FADD FADS2 FAIM FAM115A FAM120A FAM13A FAM149A FAM149B1 FAM164C FAM169A FAM173A FAM176B FAM184A FAM186A FAM190B FAM198B FAM21A///FAM21B///FAM21C///FAM21D FAM30A FAM38B FAM47E///STBD1 FAM75A1///FAM75A2///FAM75A3///FAM75A4///FAM75A5///FAM75A6///FAM75A7 FAM82B FAM86B1 FAM8A1 FANCA FANCE FAR2 FBN1 FBXL18 FBXO46 FBXO9 FBXW12 FCAR FCER2 FCGR1A FCGR3B FCHSD2 FDPS FECH FEM1B FER1L4 FETUB FEZ2 FGB FGF12 FGF18 FGF3 FGFR3 FGFR4 FHOD3 FIP1L1 FKTN FLAD1 FLG FLJ11292 FLJ13197 FLJ21369 FLOT2 FLRT3 FLT1 FLT3 FLT3LG FMO3 FNBP4 FNIP1///RAPGEF6 FOLH1B FOLR1 FOS FOSB FOSL2 FOXA2 FOXE3 FOXJ3 FOXL2 FOXO1 FOXO3///FOXO3B FRS2 FRY FRZB FSHR FSTL1 FSTL3 FSTL4 FTCD FUBP1 FUT2 FXC1 FYCO1 G3BP2 GABBR1///UBD GABBR2 GABRA4 GABRA5///LOC100509612 GABRB1 GABRE GABRG2 GABRG3 GABRR1 GAD1 GADD45A GADD45B GAGE1///GAGE12F///GAGE12G///GAGE12I///GAGE12J///GAGE2A///GAGE2B///GAGE2C///GAGE2D///GAGE2E///GAGE3///GAGE4///GAGE5///GAGE6///GAGE7///GAGE8 GAGE12F///GAGE12G///GAGE12I///GAGE5///GAGE7 GAL GALNT11 GALNT2 GALNT3 GALR2 GAR1 GART GAS1 GAS7 GATA1 GATA6 GATAD2A GBAS GBP1 GC GCLC GCLM GCM2 GDF15 GDPD5 GEMIN4 GEMIN7 GEMIN8 GFAP GGNBP2 GIF GINS3 GJA3 GJA4 GJA9 GK GLDC GLE1 GLRA3 GLRX3 GLS GLT8D1 GLUL GLYAT GLYR1 GM2A GMDS GNA11 GNAL GNG13 GNG4 GNLY GNMT GOLGA2 GOLGA2B GOLGA6L5///GOLGA6L9 GOLGA8A///GOLGA8B GOSR1 GOSR2 GP5 GPC3 GPD1 GPD2 GPLD1 GPR116 GPR17 GPR172B GPR22 GPR3 GPR37 GPR52 GPR77 GPR89A///GPR89B///GPR89C GPX2 GPX4 GPX5 GPX7 GRB10 GREM1 GRIA1 GRIA2 GRIA3 GRIA4 GRIN1 GRK5 GRPEL1 GSTA3 GSTM1 GTF2A1 GTF2H3 GTF2H5 GTPBP10 GTSE1 GUCY1B2 GUCY1B3 GUF1 GULP1 GYG2 GYPA H2AFB1///H2AFB2///H2AFB3 H2AFJ HAUS2 HAUS5 HAVCR1 HBBP1 HBEGF HCFC2 HCG4 HCN2 HCRT HDGFRP3 HDLBP HEATR1 HELLS HERC4 HERC6 HES2 HFE HGF HIPK2 HIRA HIST1H2AI HIST1H2BG HIST1H2BH HIST1H2BJ HIST1H3B HIST1H3I HIST1H4E HK2 HLA-A HLA-DMB HLA-DOB HLA-DPA1 HLA-DPB1 HLA-DQA1 HLA-DQA1///HLA-DQA2 HLA-DQB1 HLA-DRA HLA-DRB1 HLA-DRB1///HLA-DRB3///HLA-DRB4 HLA-DRB1///HLA-DRB3///HLA-DRB4///HLA-DRB5///LOC100133661///LOC100294036///LOC100509582///LOC100510495///LOC100510519 HLA-DRB1///HLA-DRB4 HLA-DRB1///HLA-DRB4///HLA-DRB5 HLA-DRB4 HLA-DRB6 HLA-G HLCS HMG20B HMGA2 HMGCS1 HMGCS2 HMGXB4 HMP19 HNF1A HNF1B HNF4G HNRNPA0 HNRNPD HNRNPH1 HNRNPH3 HNRPDL HOOK1 HOXA2 HOXC5 HOXD1 HOXD10 HOXD13 HOXD3 HP HPGDS HPR HPS1 HRG HS3ST1 HSDL2 HSFX1///HSFX2 HSP90B1 HSPA14 HSPA1A///HSPA1B HSPA1L HSPB11 HSPC159 HSPD1 HTR1F HTR7P1 IBSP ICA1 ICAM1 ICAM2 ICMT ID4 IDO1 IER2 IER3 IFI16 IFI6 IFNA2 IFT74 IGF1 IGF1R IGF2BP3 IGFBP3 IGHA1 IGHA1///IGHA2///IGHD///IGHG1///IGHG3///IGHG4///IGHM///IGHV4-31///LOC100133862 IGHV5-78 IGK@///IGKC IGKC IGKV4-1 IGL@ IGLL3P IGLV1-44///LOC100290481 IGSF9B IHH IKZF2 IL10 IL10RA IL13RA2 IL15 IL1F7 IL1R1 IL1R2 IL1RN IL2 IL22 IL23A IL27RA IL4R IL6R IL7R ILF3 IMPA2 IMPDH2 INA INADL ING1 ING2 ING4 INPP1 INPP5K INSL3 INTS1 IPO5 IPP IQCE IRAK3 IREB2 IRF3 IRF4 IRS1 IRS2 IRX4 ISCA1 ITGA10 ITGA2B ITGA4 ITGAE ITGB1 ITGB4 ITGB5 ITGB8 ITIH2 ITK ITSN1 IVL JAK3 JRKL JUN JUNB KALRN KANK1 KAT5 KAZ KBTBD10 KCNA10 KCNAB1 KCNAB2 KCNB2 KCNG2 KCNH1 KCNH2 KCNJ1 KCNJ4 KCNJ5 KCNJ8 KCNK3 KDELR2 KDM3B KDM5B KDSR KHDRBS2 KHNYN KIAA0040 KIAA0101 KIAA0195 KIAA0467 KIAA0485 KIAA0895 KIAA0907 KIAA1024 KIAA1107 KIAA1109 KIAA1462 KIAA1539 KIAA1654 KIAA1751 KIF15 KIF20A KIF23 KIF2C KIF5A KIR2DL4 KIR3DL1///KIR3DL2///LOC727787 KIRREL KLF1 KLF13 KLF4 KLF6 KLHDC10 KLHL22 KLHL23 KLRAP1 KLRB1 KLRD1 KPNA1 KRIT1 KRT13 KRT33A KRT35 KRT6A KRT6B KSR1 KTN1 KYNU L3MBTL1 LAGE3 LAMA2 LAMB4 LAPTM5 LARGE LARP4 LARP4B LARS2 LAT///SPNS1 LCK LDHC LDLR LEFTY2 LEPREL1 LGR5 LHFP LHX2 LIFR LIG1 LILRA6///LILRB3 LILRB2 LILRP2 LIM2 LIMCH1 LIMS2 LLGL1 LMAN1L LMAN2 LMF1 LMNA LMO3 LOC100127886 LOC100130331 LOC100130741 LOC100132247///LOC348162///LOC613037///LOC728888///LOC729978///NPIPL2///NPIPL3 LOC100134822///LOC100288069 LOC100272216 LOC100272228 LOC100287076 LOC100287483 LOC100287590 LOC100287927 LOC100289410 LOC100289775///WNT7B LOC100293553 LOC100505960 LOC100506168 LOC100506571 LOC100507666///NPIPL2 LOC100509749 LOC100510712///MTX1 LOC157627 LOC202181 LOC283079 LOC284649 LOC441259///PMS2L2///PMS2P1///PMS2P6 LOC441259///PMS2P1///PMS2P6 LOC57399 LOC647070 LOC729799 LONP1 LOX LPA///PLG LPIN2 LPP LRBA LRCH1 LRMP LRRC23 LRRC41 LRRC61 LSAMP LSG1 LSM12 LSM14B LSS LTBP3 LTBR LUC7L3 LY6D LY9 LYN LYVE1 LYZL6 LZTS1 MAB21L2 MAD2L1BP MAFF MAFG MAGEA1 MAGEA9///MAGEA9B MAGEB4 MAGEL2 MAGOH MAGOH2 MAK16 MAN1A2 MAN2A1 MAN2A2 MAP2K7 MAP4 MAP7D3 MAP9 MAPK11 MAPK6 MAPK8 MAPT MARCKS MARCO MASP2 MAST2 MAT2A MAX MAZ MBTD1 MC4R MCAM MCF2 MCFD2 MCL1 MCM10 MCM3AP MCM4 MCM7 MDM1 MDM4 MEA1 MEAF6 MECOM MED13 MED27 MED8 MED9 MEF2C MEF2D MEOX2 MEP1A METTL2B METTL5 METTL7A MFAP3L MFI2 MGAT4B MGC4294 MGC87042 MGP MIA3 MICAL2 MICALL1 MIER2 MIR21///TMEM49 MIR622 MKI67 MKRN2 MLF1IP MLL MLL4 MLLT4 MLXIP MMP1 MMP11 MMP14 MMP16 MMP20 MMP24 MMP28 MN1 MNS1 MOBKL3 MON1B MORC1 MOXD1 MPDZ MPHOSPH10 MPHOSPH9 MPI MPPE1 MPZL1 MR1 MRP63 MRPL22 MRPL42 MRPL46 MRPS14 MRPS31 MS4A2 MS4A3 MSH6 MSI1 MSLN MTA1 MTAP MTHFD2L MTL5 MTM1 MTMR11 MTMR2 MTRF1L MTSS1 MTTP MTUS1 MUC2 MUC5AC MUC5B MXD1 MXD4 MXRA7 MYBBP1A MYBL2 MYC MYCBP2 MYH10 MYH11 MYH6///MYH7 MYL10 MYL6 MYO6 MYO7A MYO9A MYO9B MYOT MYST1 MYST4 N4BP2L1 NAA35 NAAA NAALAD2 NAALADL1 NAB1 NAB2 NACC2 NAP1L4 NAT1 NAT15 NAV3 NBEAL2 NBN NCDN NCF1C NCR1 NCR3 NCRNA00115 NCRNA00185 NDEL1 NDRG2 NDUFA1 NDUFB7 NDUFB8 NDUFB8///SEC31B NDUFS1 NDUFS8 NDUFV2 NEAT1 NEFM NEK1 NEK2 NEK3 NELL2 NENF NEURL NEUROG2 NF2 NFAT5 NFATC3 NFE2L1 NFE2L3 NFIB NFIC NFKB2 NFKBIA NFKBIB NFX1 NFYB NID1 NIPAL3 NKIRAS2 NKTR NKX2-1 NKX3-1 NLGN1 NLRP1 NLRP3 NME3 NMNAT2 NMT2 NOD1 NOL12 NOLC1 NOS2 NOV NOVA2 NOX1 NPAS2 NPAS3 NPBWR2 NPPC NQO1 NQO2 NR0B2 NR1D2 NR1H2 NR1I3 NR2F1 NR2F6 NR4A1 NR4A2 NR4A3 NRG1 NRL NRXN1 NSA2 NSD1 NSL1 NSUN5 NSUN5P1 NSUN5P2 NSUN6 NT5DC2 NT5E NTN3 NTRK2 NUBPL NUDC NUDT1 NUDT4 NUDT4///NUDT4P1 NUP210 NUP88 NUP98 NXF3 OAZ2 OBSL1 OGFR OLAH OLFML1 OLFML2B OPRL1 OR10C1 OR12D3///OR5V1 OR2H2 OR2J2 OR7C1 ORC4 ORC5 OSBPL10 OSBPL2 OTUD4 OTUD7B P2RY1 PA2G4 PABPN1 PACS2 PAGE1 PAIP1 PAK3 PANK2 PANX1 PAPOLA PAQR4 PARD6B PARG PARK2 PARP2 PARP8 PASK PAWR PAX6 PAX8 PAX9 PBRM1 PCBP2 PCDH11X///PCDH11Y PCDH7 PCDHA6 PCGF3 PCNXL2 PCP4 PCSK5 PCSK6 PCSK7 PDCD5 PDCD6 PDCL PDE11A PDE1B PDE3A PDE6D PDE9A PDGFA PDIA2 PDIA5 PDLIM4 PDPN PDSS2 PDZD8 PEA15 PECR PELI2 PER1 PEX10 PEX6 PGAM2 PGLYRP4 PGRMC1 PGRMC2 PGS1 PHF10 PHF15 PHIP PHOX2A PIBF1 PIGC PIGH PIGO PIGT PIK3CD PIK3CG PIK3R4 PIN1 PIN4 PIP4K2B PIR PKD1 PKIA PKNOX2 PKP2 PLA2G2E PLA2G6 PLA2R1 PLAGL1 PLAU PLCB4 PLCE1 PLCXD1 PLEKHA1 PLEKHF1 PLK3 PLK4 PLLP PLN PLUNC PLXNA2 PLXNB1 PMAIP1 PMF1 PML PMM2 PMP2 PMS2L2 PMS2P3 PNKP PNLIPRP1 PNMA2 PNMAL1 PNO1 POLH POLR2B POLR2E POLR2J///POLR2J2///POLR2J3 POLR2L POLR3G POMGNT1 POMZP3 PON3 POU1F1 PP14571 PPARA PPCDC PPFIBP1 PPIA PPIAL4A PPIG PPIL2 PPM1F PPP1R1A PPP1R2P9 PPP1R3A PPP2R1B PPP2R2B PPP2R5B PPP3CC PPP3R1 PRB3 PRDX2 PRICKLE3 PRKAA2 PRKAB1 PRKAG1 PRKAG2 PRKAR1A PRKCA PRKCB PRKDC PRKG2 PRLR PRM2 PRMT3 PRO2012 PRPF31 PRPH PRPS1 PRR11 PRR3 PRR4 PRSS1///PRSS2///PRSS3 PRSS21 PRSS53 PRUNE PRY PSD3 PSG9 PSMB1 PSMB5 PSMB9 PSMD1 PSMD13 PSPH PTAFR PTBP1 PTCD3 PTCRA PTGER4 PTGIS PTH2R PTHLH PTK6 PTP4A2 PTPN11 PTPN13 PTPN22 PTPN4 PTPRD PTPRF PTPRO PTTG3P PURA PVR PVT1 PYY QPRT QSER1 RAB11B RAB28 RAB2A RAB38 RAB3D RAB3GAP1 RAB7A RAB9A RABIF RABL2A///RABL2B RAD23A RAD50 RAD52 RAD54B RALBP1 RALGAPA1 RANBP3 RAP2A RAPGEF1 RAPGEF2 RARA RARB RASGRF1 RASL12 RASSF4 RASSF8 RAX RBBP4 RBBP6 RBBP9 RBL1 RBL2 RBM22 RBM3 RBM41 RBM6 RBM9 RBMXL2 RBMY2FP RBPMS RCE1 RCHY1 RCN2 RCN3 REL RELA REN REPS1 REPS2 RERE RFC4 RFK RFX5 RFX7 RFXANK RGPD5///RGPD6///RGPD8 RGS1 RGS10 RGS12 RGS13 RGS16 RGS4 RHD RHEB RHO RHOB RHOBTB3 RIBC2 RIMBP2 RIN3 RIOK3 RIPK1 RLN1 RNASE1 RNASEH2B RND3 RNF114 RNF220 RNF41 RNF8 RNFT2 ROR1 RORA RPAIN RPGR RPH3A RPL13P5 RPL21///RPL21P19///RPL21P28 RPL38 RPLP2 RPP40 RPRD2 RPS11 RPS14 RPS2P45 RPS6KA4 RPS6KA6 RPS6KB2 RPS8///RPS8P8 RREB1 RRP7A RTEL1 RTEL1///TNFRSF6B RTP4 RUNX1T1 RUNX3 RXFP3 RYK S100A8 S100A9 S1PR2 SAA1///SAA2 SAA4 SALL1 SAMD14 SAP130 SATB1 SBF1 SBNO1 SBNO2 SCAI SCAND2 SCAPER SCARB1 SCGB1A1 SCGB1D1 SCIN SCLY SCN11A SCN3A SCN3B SCN5A SCYL3 SDAD1 SDF2 SEC14L1 SEC62 SEC63 SECISBP2L SELE SELPLG SEMA3C SENP5 SENP7 SEPHS1 SEPT2 SEPT6 SEPT9 SERBP1 SERF1A///SERF1B SERPINA7 SERPINB1 SERPINB13 SERPINB4 SERTAD3 SETD2 SETD6 SF3B2 SF3B3 SFRP4 SFRS18 SFTPA2 SFTPB SFTPC SFTPD SGCD SGCE SH2D3C SH3GL3 SH3PXD2A SH3YL1 SHANK2 SHH SHMT2 SHPK///TRPV1 SHQ1 SIGLEC1 SIGLEC8 SIK1 SIRPA SIRT4 SIVA1 SKP2 SLC10A3 SLC11A1 SLC12A4 SLC14A2 SLC16A3 SLC17A1 SLC18A2 SLC1A1 SLC20A2 SLC22A1 SLC22A11 SLC22A17 SLC22A2 SLC22A6 SLC23A2 SLC24A1 SLC25A14 SLC25A21 SLC25A36 SLC25A6 SLC26A10 SLC26A3 SLC26A4 SLC27A6 SLC2A11 SLC35A3 SLC35D2 SLC35E1 SLC38A7 SLC46A3 SLC5A2 SLC5A3 SLC6A1 SLC6A2 SLC6A20 SLC7A11 SLC7A6 SLC7A9 SLC9A5 SLCO1A2 SLCO5A1 SLIT3 SLITRK5 SMA4 SMAD1 SMARCA4 SMC1A SMC2 SMC3 SMC6 SMCP SMEK1 SMG6 SMPD1 SMPDL3A SMPDL3B SMS SMTN SNAI1 SNAPC3 SNCG SNORA21 SNRNP25 SNRNP35 SNRPA1 SNRPD1 SNTB2 SNX13 SNX15 SNX27 SOCS1 SOCS3 SOCS5 SOCS6 SOD2 SOS2 SOX13 SOX14 SOX9 SP1 SP140L SP2 SPAG1 SPAG16 SPAG4 SPAG5 SPAG8 SPANXB1///SPANXB2///SPANXF1 SPATA2L SPATA6 SPEN SPIN2A///SPIN2B SPINK1 SPINK4 SPON2 SPP1 SPRED2 SPTAN1 SPTBN1 SQLE SRCAP SRGN SRPK2 SRPX SRRT SRSF6 SRY SS18L1 SSPN SST SSX1 SSX2///SSX2B SSX2IP SSX4///SSX4B SSX7 ST14 ST6GALNAC4 ST7 STAG3L1 STAR STAT2 STAT5A STAT5B STC1 STC2 STIL STK32B STRADA STS STT3A STX17 SUGP2 SUPT3H SUPT4H1 SUZ12P SYCE1L SYN1 SYN3 SYNE1 SYT1 SYT11 SYT13 SYT2 SYT5 TACR1 TACR3 TAF1 TAF12 TAF13 TAF4B TAF9B TAOK3 TAP1 TAPBP TAPBPL TARDBP TARP///TRGC2 TASP1 TBC1D22B TBC1D2B TBC1D9B TBL1X TBL3 TBP TBX1 TBX19 TBX6 TCEB3 TCF21 TCL6 TCOF1 TCP11 TCP11L1 TDRD3 TEK TES TESC TESK2 TET3 TEX12 TFB1M TFDP1 TFPI TFR2 TFRC TGFA TGFB2 TGFBR2 TGIF1 TGIF2 TGM2 THAP10 THAP4 THG1L THOC2 THPO THRA THSD7A THY1 THYN1 TICAM1 TIGD6 TIMM23///TIMM23B TLX2 TLX3 TM2D1 TM4SF1 TMC6 TMC7 TMCC1 TMEFF1 TMEM151B TMEM187 TMEM194A TMEM2 TMEM39A TMEM50B TMEM57 TMEM63A TMEM70 TMEM97 TMF1 TMPO TNF TNFAIP2 TNFAIP3 TNFRSF11B TNFRSF4 TNFSF18 TNFSF8 TNK2 TNNI2 TNNI3K TNP1 TNPO2 TNPO3 TOM1L1 TOMM20 TOX TOX3 TP53TG1 TP63 TPD52 TPM1 TPP2 TPR TPRA1 TRA2A TRAC///TRAJ17///TRAV20 TRADD TRAF3IP2 TRAF6 TRAPPC10 TRAV8-3 TRBC1 TRDN TRIB1 TRIM2 TRIM24 TRIM27 TRIM31 TRIM33 TRIM37 TRIM49///TRIM49L2 TRIO TROVE2 TRPC1 TRPM6 TRPV1 TSC22D2 TSKS TSPAN5 TSPYL4 TTC22 TTC3 TTC38 TTK TTLL5 TUB TUBB4Q TUBGCP2 TUG1 TULP1 TUSC2 TUSC3 TWF2 TWSG1 TXK TXLNG TXNDC3 TYMS U2AF2 UBA6 UBAP2L UBE2J1 UBE3A UBE3B UBE4B UBQLN3 UBQLN4 UBXN1 UBXN4 UBXN8 UCHL1 UCHL5 UGCG UGT2B4 ULBP2 ULK2 UNC5B UNC93A UPB1 UPF3A UQCRB USE1 USP12 USP16 USP19 USP2 USP21 USP34 USP47 USP49 USP6NL UTP14A UTY VAC14 VAMP1 VAMP2 VANGL1 VAPB VARS VASH2 VAV3 VCAN VEGFA VEGFC VEZF1 VGLL1 VPS13D VPS16 VPS33B VRK1 VSNL1 WASL WBSCR22 WDR1 WDR13 WDR18 WDR73 WDR74 WHAMM///WHAMML1///WHAMML2 WHSC1L1 WHSC2 WIF1 WIPI2 WISP2 WLS WNK1 WNT2B WNT5A WNT6 WRNIP1 WWOX WWTR1 XAB2 XPO6 XRCC2 YIPF6 YWHAZ YY1 ZBED2 ZBTB1 ZBTB40 ZBTB43 ZBTB7A ZC3H12A ZC3H7B ZCCHC14 ZDHHC11 ZEB1 ZFAND3 ZFAND5 ZFHX3 ZFP161 ZFP2 ZFP36 ZFP36L2 ZFR ZGPAT ZIC3 ZKSCAN1 ZKSCAN3 ZKSCAN4 ZKSCAN5 ZMAT3 ZMYND8 ZNF14 ZNF155 ZNF157 ZNF16 ZNF160 ZNF174 ZNF187 ZNF202 ZNF204P ZNF222 ZNF224 ZNF253 ZNF259 ZNF259P1 ZNF271 ZNF273 ZNF280A ZNF286A ZNF3 ZNF318 ZNF329 ZNF335 ZNF345 ZNF365 ZNF44 ZNF451 ZNF468 ZNF480 ZNF493 ZNF518A ZNF549 ZNF550 ZNF552 ZNF587 ZNF609 ZNF613 ZNF639 ZNF671 ZNF673 ZNF682 ZNF688 ZNF702P ZNF749 ZNF771 ZNF80 ZNF85 ZNHIT1 ZSCAN12 ZXDB | BAL_26_A2B0 BAL_13_A0B0 BAL_17_A0B0 BAL_3_A1B0 BAL_31_A0B1 BAL_45a_A1B0 BAL_44b_A0B0 BAL_1_A0B0 BAL_25_A0B0 BAL_12_A1B0 BAL_40_A0B0 BAL_39_A0B1 BAL_37_A1B1 BAL_4_A0B0 BAL_33_A0B1 BAL_18_A1B0 BAL_43_A0B0 BAL_42_A0B0 BAL_47b_A0B1 BAL_27a_A0B0 BAL_45b_A2B0 BAL_27b_A2B1 BAL_15a_A0B1 BAL_20b_A1B1 BAL_47c_A0B1 BAL_47a_A0B0 BAL_21_A0B0 BAL_15b_A0B0 BAL_29_A1B0 BAL_41_A1B0 BAL_44a_A0B0 BAL_19_A0B0 BAL_46_A2B0 BAL_20a_A1B1 NeverSmoker_38 |
| **6** | AAK1 AANAT ABAT ABCB6 ABCG4 ABHD2 ACACB ACAT2 ACE ACIN1 ACSBG2 ACSL6 ACSM3 ACTR1A ACVR1B ADAM12 ADAM23 ADCK3 ADCY1 ADD1 ADNP2 ADPRH ADRBK1 ADSL AFF3 AFP AGGF1 AHCTF1 AIF1 AKAP9 AKR1B10 AKT2 AKT3 ALDH1A1 ALDH3A2 ALDH5A1 ALDH6A1 ALG13 ALG3 ALPI ALPK1 AMBRA1 ANKRD12 ANKRD36B ANKRD7 ANO3 ANP32A///ANP32D AP2B1 AP3B1 AP3S2 APBA3 APC2 API5 APLP2 APOA1 APOBEC2 APOBEC3F AQP3 ARAF ARAP2 ARF3 ARFRP1 ARHGDIA ARHGEF38 ARID1A ARID3B ARID4B///RBM34 ARIH2 ARL4C ARL5A ARPP21 ASAP1-IT ASB4 ASB7 ASCL2 ASMTL ASPM ATG13 ATG16L1 ATG2A ATG4B ATN1 ATP13A2 ATP2A3 ATP2B2 ATP5G2 ATP5I ATP6V0A1 ATP6V0E1 ATP6V1D ATPIF1 ATR ATXN3L AVIL AZGP1P1 B4GALT1 B4GALT3 BACH2 BAIAP2 BAT2L1 BAT2L2 BATF BBS7 BCAT1 BCAT2 BCCIP BCL11B BCL2 BCL3 BCOR BFSP1 BGN BICC1 BLM BLZF1 BMP15 BMP8A BMPR1B BOP1 BRE BSG BST2 BTF3 BTN2A1 BTNL8 BUB1 C10orf137 C10orf72 C10orf76 C11orf9 C11orf95 C14orf139 C14orf156 C14orf162 C15orf28 C15orf34 C16orf5 C16orf58 C19orf2 C19orf22 C19orf26 C19orf29 C19orf40 C19orf6 C1orf105 C1orf135 C1orf144 C1orf156 C1orf216 C1orf35 C1orf54 C1orf56 C1orf77 C1orf89 C20orf20 C21orf91 C2orf18 C2orf24 C2orf34 C2orf44 C2orf49 C3 C3orf64 C5orf4 C7orf64 C8orf33 C9orf156 C9orf38 C9orf91 C9orf95 CA5A CABIN1 CACNA1G CADM3 CADM4 CALCOCO1 CALCRL CALD1 CALHM2 CALM1 CALR CALU CAMK2B CAPN3 CAPNS1 CAPZB CASP5 CASP9 CBL CBWD1///CBWD2///CBWD3///CBWD5///CBWD6///CBWD7///LOC100507355 CC2D1A CCDC72 CCDC93 CCL24 CCL25 CCL3///CCL3L1///CCL3L3 CCNB1IP1 CCND2 CCR7 CD1A CD1B CD200 CD24 CD3E CD3EAP CD4 CD40 CD47 CD5L CD6 CD9 CD93 CDC25C CDC27 CDC42BPB CDH5 CDK11A///CDK11B CDK16 CDK9 CDKN1C CDO1 CDYL CEACAM8 CEBPA CEBPE CELA2A CELA2A///CELA2B CELA2B CELF1 CENPE CENPF CEP152 CETN3 CETP CG030 CGB///CGB5///CGB7 CH25H CHCHD7 CHCHD8 CHD4 CHD7 CHI3L1 CHI3L2 CHMP4A CHN2 CHRM5 CHRNA5 CHST12 CIDEB CINP CIR1 CIZ1 CKAP4 CLC CLCA1 CLCN7 CLN5 CLN6 CLPTM1 CMAS CNOT2 CNR1 CNTF///ZFP91///ZFP91-CNTF CNTFR COL10A1 COL4A3 COPA COPS2 COPS6 COPS7A CORO1A CORO1B COX15 COX5B COX7C CPA4 CPB2 CREM CRHR1 CRIM1 CRIP1 CROCC CROCCP3 CRYGA CSE1L CSN2 CSNK1G2 CSNK2A1 CST3 CTAGE11P CTBP1 CTDSP2 CTH CTNS CTSH CXCL1 CXCL12 CXCL13 CYB5A CYBRD1 CYLC1 CYP11A1 CYP1A1 CYP1B1 CYP2B6 CYP2C9 CYP2D6 CYP2W1 CYP7A1 DAXX DBC1 DBR1 DCAF8 DCBLD2 DCTN5 DCUN1D2 DCUN1D4 DCX DDX11///DDX12///LOC642846 DDX17 DDX25 DDX49 DDX54 DDX58 DDX6 DDX60 DERL2 DHCR24 DIAPH1 DIAPH3 DICER1 DKFZp547G183 DKK1 DLGAP4 DNM2 DNMT3L DNPEP DOPEY1 DOT1L DPH1///OVCA2 DPH5 DPYSL3 DRD4 DRG2 DROSHA DSC1 DSPP DTNB DUX2 DVL3 DYNC1LI2 EBP EDDM3A EDNRA EEA1 EEF1A1 EEF1A1///EEF1A1P9 EEF1D EFHC2 EFHD2 EIF3G EIF3H EIF4E EIF4G1 EIF4G2 EIF5A ELAC2 ELAVL1 ELF1 ELK1 ELK4 ELL2 ELOVL5 ELP3 EMP1 ENTPD4 EPAG EPAS1 EPB41L1 EPM2A EPRS ERAL1 ERCC4 ERCC5 ERG ESR1 ETFDH ETV5 EXOC7 EXOSC10 EXOSC4 EXOSC8 EZR F7 FAIM3 FAM108B1 FAM119B FAM120A FAM125B FAM13B FAM182B FAM190B FAM193B FAM20B FAM55D FAM70A FAM76A FARS2 FARSA FASN FAU FBLN1 FBN1 FBXO11 FBXO46 FBXW11 FDPS FER1L4 FETUB FGF18 FGF2 FGF22 FGF5 FGFR1OP FGFR2 FGFR3 FH FIP1L1 FKBP1A FKBP1B FKBP6 FKRP FLII FLJ11292 FLJ13224 FLJ23519///RNH1 FLRT2 FLT3LG FMO4 FN1 FOXF2 FOXN2 FOXN3 FPGS FRMD4A FTSJ2 FUS FUT2 FXR1 FYB G3BP1 GABBR1 GABRE GABRG2 GABRR1 GAD1 GADD45GIP1 GAL GALR2 GANAB GAR1 GAS1 GAS6 GAS7 GATA2 GATAD1 GCLM GCN1L1 GCSH///LOC100329108 GDI1 GEMIN4 GFI1 GFI1B GGA1 GGCX GIMAP4 GINS3 GK GK2 GKN1 GLP1R GLS GLTSCR2 GMEB2 GML GNA13 GNAI2 GNAL GNAO1 GNAQ GNAZ GNB2 GNB3 GNG11 GNL1 GNLY GOLGA6L4///PML GP1BB///SEPT5 GPN2 GPR107 GPR116 GPR124 GPR17 GPR172A GPR19 GPR44 GPR45 GPR63 GPRC5B GPT GRAMD4 GRIA2 GRIK3 GRIN1 GRIN2C GRINA GRLF1 GRM1 GRM4 GRM8 GRSF1 GSN GTSE1 GUCY1A3 GYG2 GZMA GZMB HAB1 HAP1 HARS2 HBD HBG1///HBG2 HBP1 HCCS HDAC4 HDAC6 HDDC2 HERC2P2///HERC2P9 HERC6 HEXIM1 HFE HIBCH HIF3A HIGD1B HIP1 HIPK2 HIST1H2BO HIST1H3G HIST1H3J HIST3H3 HJURP HK2 HLA-A///HLA-F///HLA-J HLA-F HMGA1 HMGCR HMGCS2 HMGXB4 HNF4A HNRNPA1///HNRNPA1L2///HNRNPA1P10///LOC728643 HNRNPA2B1 HNRNPA3///HNRNPA3P1 HNRNPD HNRNPM HOMER3 HOXA5 HOXC8 HOXD10 HPR HPS1 HRH3 HSD17B14 HSD17B3 HSPB6 HTATSF1 HTR1D HTRA2 HUWE1 HYAL2 ICOS IDS IFI27 IFI44 IFI44L IFI6 IFIH1 IFIT1 IFITM1 IFNA1 IFRD1 IGF1 IGF2BP3 IGHA1///IGHA2///IGHG1///IGHG4///IGHM///IGHV4-31 IGHA1///IGHD///IGHG1///IGHG3///IGHM///IGHV3-48///IGHV4-31///LOC100291917 IGHG1 IGHM///LOC100133862 IGL@ IGLV4-60 IKBKG IL18 IL1RL1 IL2RG IL5RA IL6 ILK IMPA2 INPP4A INSR IPW IQGAP1 IREB2 IRF5 IRGQ ISG15 ISG20L2 ITGA4 ITGA7 ITGB1 ITIH2 ITK JUND KBTBD11 KCNA5 KCNAB1 KCNJ8 KCNK13 KCNK15 KCNMB1 KCNN3 KCNQ1 KCNQ4 KCNS1 KCTD13 KDR KHDC1L KIAA0101 KIAA0125 KIAA0182 KIAA0240 KIAA0368 KIAA0586 KIAA1467 KIAA1539 KIF25 KIF5A KIF5C KIR2DL2 KIR2DL3 KIR3DL1///KIR3DL2///LOC727787 KIR3DX1 KLF3 KLF9 KLHL1 KLHL18 KLHL24 KLRD1 KPNA6 KRT13 KRT19P2 KRT6A KRT9 LAG3 LAIR2 LALBA LAMB1 LAMP1 LARGE LATS1 LBH LCAT LCMT2 LDHC LECT1 LEF1 LEP LEPR LEPROT LGALS13 LGALS8 LHPP LIFR LILRA6 LILRB1 LIMD1 LIMK2 LMAN1 LMO3 LOC100272228 LOC100293553 LOC100506168///SFPQ LOC100507328 LOC100507328///LOC100508591 LOC100507630 LOC100507851///PRAMEF1///PRAMEF2 LOC100508797 LOC100510735///RPL29 LOC145678 LOC220077 LOC729991 LOC92249 LOH3CR2A LONRF3 LOR LPIN1 LPPR2 LRP10 LRP1B LRRC16A LRRC31 LRRC37A///LRRC37A2///LRRC37A3///LRRC37A4 LRRC59 LRRK1 LSG1 LSM12 LSP1 LTB4R LTBP1 LUC7L3 LY9 MAB21L1 MAF MAGEA12 MAGEA4 MAGEB1 MAGOH MAGT1 MAL MAML3 MAMLD1 MAP7D1 MAPK13 MAPK7 MAPKAPK2 MAPRE3 MAPT MARCKS MAS1 MAZ MBOAT7 MBP MCC MCM3AP MDC1 MECOM MED20 MEFV MEGF6 MEN1 METT10D MEX3D MFI2 MFN2 MGA MGAT4B MIA2 MIA3 MICA///MICB MICALL1 MID1IP1 MINA MINK1 MKI67 MKKS MKL1 MMP1 MMP11 MMP14 MMP24 MOCS1 MRPL18 MRPL20 MRPL22 MRPL40 MRPS14 MRPS22 MRS2 MSMB MSR1 MT1E MT1F MT1M MT1X MT2A MTA1 MTAP MTDH MTMR1 MTMR11 MTMR2 MTMR8 MTR MX1 MXD1 MYH14 MYH6///MYH7 MYL3 MYO16 MYO1A MYO1C MYO6 MYO7A MYO9B MYOZ1 MYST1 MYST2 MYST3 MZT2B N4BP3 NAA10 NAA35 NAAA NADSYN1 NAP1L1 NAPA NASP NAV3 NCAPD2 NCAPD3 NCOA3 NCR1 NCR2 NDRG3 NDRG4 NDST1 NDUFA2 NDUFA4 NDUFA4L2 NDUFA6 NDUFB2 NDUFB7 NEFL NEK1 NEK3 NF2 NFATC3 NFE2L1 NFIB NFKB2 NFU1 NGDN NGLY1 NHEJ1 NIPA2 NIPSNAP1 NMT2 NNAT NOP56 NOTCH2NL NPAT NPEPPS NPFF NQO2 NR0B1 NR2F6 NR4A2 NR5A1 NRG2 NRGN NRIP2 NSMCE4A NT5C NTRK3 NUCB1 NUCB2 NUDT18 NUDT4 NUDT6 NUDT9 NUP133 NUP160 NUP210 NUPR1 NXT1 OAZ2 OGFR OGG1 OGT OMD OR12D3///OR5V1 OR1A1 OR2A20P///OR2A9P OR7A5 ORAI2 ORM1 ORM1///ORM2 OSBPL1A OTC OTOF OTUD4 P2RX4 PABPN1 PAFAH1B1 PAICS PAK3 PAK7 PALM PAMR1 PAN2 PAOX PARL PARP6 PARVB PAX3 PAX8 PBXIP1 PCBP2 PCDH7 PCGF1 PCSK7 PDCD5 PDE12 PDE4C PDE4DIP PDK2 PDLIM4 PDLIM7 PDX1 PELO PET112L PEX10 PEX11B PEX26 PEX3 PFDN5 PGD PGLS PGLYRP1 PHB PHF16 PHF3 PI4KB PIAS2 PIGF PIK3R4 PIN4 PKIA PKM2 PKNOX1 PKNOX2 PKP2 PLAGL1 PLAGL2 PLCB1 PLCE1 PLCH2 PLD1 PLD3 PLK2 PLN PLUNC PML PMP22 PMS2///PMS2CL PMS2P1 PMS2P3 PNMA2 PNMT PNN PNO1 PNP PNPLA2 PNPLA3 PNPO PODNL1 POFUT1 POLE2 POLQ POLR1B POLR1E POM121 POM121///POM121C POM121L9P PON1 PON2 POPDC3 POU2F1 POU3F1 POU4F2 POU5F1P4 PPAP2B PPDPF PPFIA1 PPFIBP1 PPIP5K1 PPP1R2P9 PPP3CC PPP5C PPP6C PRDX6 PRKAB2 PRKACA PRKCG PRKCSH PRKX PROS1 PRPF3 PRPF40A PRPF8 PRSS1///PRSS2///PRSS3 PRUNE PRUNE2 PSMB1 PSMB4 PSMD1 PSMD4 PSME2 PSME3 PSME4 PTCD1 PTCD2 PTCRA PTEN PTGDR PTGER3 PTN PTPN12 PTPN18 PTPN2 PTPRO PTRH2 PUS1 PVR PVT1 PYGB PYY2 QKI QPCT QPRT RAB11B RAB2A RAB9BP1 RABEPK RABIF RAD23A RANBP3 RANBP6 RANGAP1 RAPGEF2 RARRES1 RASA2 RASA4 RBM23 RBM25 RBM4B RBM5 RBMS3 RBMX2 RCN1 RECK RENBP REPS1 REST REV1 RFC1 RFC5 RFTN1 RGS1 RGS6 RHEB RIC8B RMND5B RNASE1 RNASEH2B RNF121 RNF126 RNF19B RNF216 RNF5 ROCK2 ROS1 RPL10A RPL12 RPL13 RPL14 RPL15 RPL17 RPL18 RPL18A///RPL18AP3 RPL22 RPL23 RPL26L1 RPL27A RPL28 RPL3 RPL32 RPL34 RPL35 RPL35A RPL37 RPL37A RPL38 RPL4 RPL5 RPL7A RPL9 RPLP2 RPS10 RPS10///RPS10P7 RPS11 RPS12 RPS15A RPS16 RPS17 RPS18 RPS19 RPS2 RPS20 RPS21 RPS26 RPS3 RPS3A RPS4X RPS6KA6 RPS8///RPS8P8 RPS9 RRAD RREB1 RRH RSAD2 RUNX1 RUSC1 RXRB RYBP S100A7 S100A8 SBF1 SBNO1 SBNO2 SCAMP2 SCAND2 SCEL SCGB1A1 SCML2 SCN2A SCN4A SCNN1D SDC2 SDF4 SDR39U1 SEC14L3 SEC23IP SECTM1 SEMA6A SENP6 SEPX1 SERPINB2 SERPINB3 SERPINB3///SERPINB4 SERPINB9 SERPIND1 SETD3 SETD4 SETX SF3A2 SFRS15 SFTPA2 SFTPB SFTPC SGCG SGSH SH3BGRL3 SH3GL3 SHC1 SHC3 SHOX2 SIRPG SIX3 SIX5 SLC11A2 SLC12A5 SLC14A2 SLC16A3 SLC16A7 SLC17A5 SLC19A1 SLC1A1 SLC1A4 SLC22A7 SLC25A1 SLC25A36 SLC26A3 SLC2A4RG SLC30A5 SLC35D1 SLC35E1 SLC38A10 SLC38A7 SLC39A8 SLC41A3 SLC4A1AP SLC7A1 SLC7A4 SLC8A2 SLCO1A2 SLCO1B1 SLCO3A1 SLIT3 SLPI SLX1A///SLX1B SMARCA4 SMARCB1 SMARCD1 SMARCE1 SMC1A SMPD1 SNAPC5 SNCA SNRPA1 SNRPG SNRPN///SNURF SNUPN SNX1 SNX27 SOBP SOD2 SOD3 SOLH SORBS2 SORL1 SOS2 SOX18 SOX21 SPANXB1///SPANXB2///SPANXF1 SPATA2L SPHK1 SPI1 SPIN2A///SPIN2B SPN SPON1 SPRR1A SPRR1B SPRR3 SPRY4 SPSB1 SPTAN1 SPTBN1 SRP72 SRPR SRRM1 SRRM2 SRSF4 SSBP3 SSTR5 SSX2///SSX2B SSX7 ST6GALNAC4 ST7 STC1 STK17A STK19 STK3 STK39 STMN4 STOM STRADA STRN4 STS STX2 STXBP2 SUGP1 SUPT6H TAAR3 TACR3 TADA3 TAF10 TAGLN2 TAOK2 TAP1 TAPT1 TAS2R8 TAX1BP1 TBC1D10B TBL1X TBR1 TBXA2R TCEB2 TCF3 TCF4 TCF7 TCF7L2 TCP10 TDRD3 TDRKH TEP1 TEX12 TFPT TGFB2 THAP4 THBS1 THRAP3 THTPA TIMM8B TIMP1 TINAGL1 TK2 TLX2 TM4SF1 TM4SF4 TMED7 TMEFF1 TMEM180 TMEM206 TMEM214 TMEM8A TMOD3 TMPO TMX2 TNC TNFAIP2 TNFRSF14 TNNI3K TNXB TOP3A TOX TP63 TPP1 TPP2 TPR TPSAB1 TPX2 TRAC///TRAJ17///TRAV20 TRAF3 TRAK2 TRBC2 TREX2 TRIM24 TRIM26 TRIM3 TRIM31 TRIM37 TRIM44 TRIM8 TRPC2 TRPM6 TSC22D1 TSHR TSKS TSN TSPAN4 TSR1 TTC17 TTC3 TTC33 TTC39A TTTY9A///TTTY9B TUBB TUBGCP2 TUBGCP4 TWF1 UAP1 UBAP1 UBE2L3 UBE3A UBL5 UBN1 UBQLN4 UBXN4 UCP2 ULK1 UNG UQCRC1 USE1 USF2 USP12 USP18 USP20 USP21 USP34 USP6 UTP18 UTP20 UTS2 UXS1 VAC14 VAMP2 VCAM1 VCAN VCL VDAC3 VPS53 WAS WASF1 WBP4 WDR43 WDR45 WDR55 WDR67 WDR74 WFDC1 WIPI1 WIZ WNK1 WNT10B WNT6 WSCD1 XPA XRCC4 XYLB YARS2 YKT6 YTHDF1 YWHAE ZBTB16 ZC3H13 ZC3H14 ZER1 ZFP161 ZFP36L1 ZFP36L2 ZHX3 ZIC3 ZKSCAN5 ZMYM1 ZMYM2 ZMYM5 ZMYND8 ZNF107 ZNF132 ZNF133 ZNF16 ZNF160 ZNF175 ZNF205 ZNF221///ZNF230 ZNF280B ZNF280D ZNF287 ZNF32 ZNF330 ZNF365 ZNF407 ZNF43 ZNF440 ZNF442 ZNF451 ZNF467 ZNF507 ZNF528 ZNF529 ZNF592 ZNF611 ZNF721 ZNF74 ZSCAN2 ZYX | BAL_46_A2B0 BAL_44a_A0B0 BAL_15b_A0B0 BAL_19_A0B0 BAL_21_A0B0 BAL_45a_A1B0 BAL_33_A0B1 BAL_47c_A0B1 BAL_37_A1B1 BAL_45b_A2B0 BAL_47a_A0B0 BAL_44b_A0B0 BAL_20a_A1B1 BAL_12_A1B0 BAL_4_A0B0 BAL_43_A0B0 BAL_27a_A0B0 BAL_26_A2B0 BAL_42_A0B0 BAL_25_A0B0 BAL_13_A0B0 BAL_29_A1B0 BAL_1_A0B0 BAL_27b_A2B1 BAL_15a_A0B1 BAL_40_A0B0 BAL_3_A1B0 BAL_20b_A1B1 BAL_41_A1B0 BAL_31_A0B1 BAL_17_A0B0 BAL_47b_A0B1 BAL_18_A1B0 BAL_39_A0B1 FormerSmoker_69 |
| **7** | AADAC AAK1 ABAT ABCD2 ABCD3 ABHD6 ABO ACAA1 ACAP2 ACIN1 ACTB ACTG1 ACYP1 ADAM17 ADAM28 ADAM8 ADCY3 ADD3 ADH5 ADM ADORA2A///SPECC1L ADRM1 AGAP1 AGK AGL AGPAT2 AHCYL2 AKAP1 AKAP7 AKAP8L AKAP9 AKR1B10 AKR1C2 AKR1C3 ALCAM ALDH3A2 ALDH9A1 ALG13 ALOXE3 ALPL AMD1 AMMECR1 AMPD2 ANAPC10 ANAPC5 ANGEL2 ANKHD1///ANKHD1-EIF4EBP3 ANKRA2 ANKRD36B AOC3 AP1G2 API5 APOBEC3B APOBEC3C APOBEC3G APOOL APPL1 ARF1 ARHGAP10 ARHGAP26 ARHGEF7 ARID3A ARL5A ARMC1 ASF1A ASL ASNSD1 ATF1 ATF3 ATF4 ATG2A ATG4A ATM ATMIN ATN1 ATP1B1 ATP2C1 ATP5C1 ATP5G2 ATP5S ATP8B3 ATRN ATRX B4GALT6 BAHD1 BANP BASP1 BCL2L11 BCL3 BCLAF1 BDH2 BLVRA BPGM BPTF BRD2 BRD3 BRD8 BRWD1 BTBD3 BTG2 BTN3A1 BTN3A3 BTNL8 C10orf68 C11orf17 C11orf71 C15orf39 C16orf42 C16orf57 C16orf7 C17orf101 C17orf81 C18orf1 C19orf22 C1orf112 C1orf50 C21orf33 C22orf9 C2orf43 C2orf72 C3orf64 C5 C5orf15 C6orf105 C6orf162 C6orf64 C8orf44///SGK3 C9orf16 C9orf82 C9orf95 CA2 CACNG1 CACYBP CALCB CALM1 CAMK2G CAMP CAMTA1 CASP8AP2 CBR1 CCDC101 CCDC6 CCDC88A CCDC90B CCDC91 CCL24 CCL3///CCL3L1///CCL3L3 CCL4 CCL5 CCNB1IP1 CCND1 CCR3 CD2 CD200 CD22 CD2AP CD300A CD55 CD6 CD84 CD93 CDA CDC16 CDC42BPA CDC73 CDCA4 CDH20 CDK10 CDK11A CDK11A///CDK11B CDK12 CDK13 CDK2AP2 CDK9 CDKN1A CDYL CEACAM1 CEACAM3 CEBPD CENPE CEP152 CEP350 CETN3 CHCHD7 CHERP CHI3L1 CHORDC1 CHRNG CHST15 CIR1 CKAP2 CKMT2 CLC CLCN3 CLEC10A CLEC4E CLEC4M CLIP1 CLMN CLN3 CLPX CMAH CNGA1 CNOT8 CNTN5 COBLL1 COG5 COL1A1 COL5A3 COMMD8 COPE CORO2A COX16 COX7A2 COX7C CPNE1 CPNE3 CR1 CRHR1 CRIM1 CRYBB2 CSE1L CSF2RA CSF3R CSH1 CSNK1G3 CST2 CSTF2T CTBP2 CTSO CUL4A CUX1 CXCL1 CXCL10 CXCL2 CXCL9 CXCR1 CXCR2 CXCR5 CXCR6 CYB5A CYP1A1 CYP1B1 CYP26B1 CYP3A5 CYP51A1 CYR61 CYTH4 DAXX DBF4B DCAF15 DCTN1 DCTN3 DDAH2 DDX17 DDX18 DDX28 DDX3Y DEFA1///DEFA1B///DEFA3 DENND3 DENND4C DENR DERA DES DFFA DHFR DHX29 DLG1 DLGAP4 DMD DNAH3 DNAJB1 DNAJC13 DOCK6 DOCK9 DPYD DSG3 DSPP DUOX2 DUS4L DUSP1 DUSP11 DUSP2 DUSP5 DYNLL1 DYRK2 EBNA1BP2 ECE1 EDA EDNRB EEF1D EGR1 EGR3 EGR4 EHD1 EHHADH EID1 EIF1AX EIF4E2 EIF4G1 EIF5A EIF5B ELK3 ELMO2 ELSPBP1 EMR2 ENOX2 ENPP2 EPB41L3 EPHA5 EPHA7 EPM2A EPOR EPRS ERAP1 ERAP2 ERG ERGIC2 ERI2 ERLIN2 ERMAP ETS2 EXOSC2 EXOSC4 EXT1 FAM134B FAM178A FAM20B FAM3C FAM50A FAM65B FAM69A FAM82B FANCL FAR2 FBRS FBXL18 FBXO11 FBXO3 FBXO42 FBXO9 FBXW11 FCAR FCGBP FCGR3B FCN1 FECH FEZ1 FFAR2 FGFR3 FHL1 FKTN FLOT1 FLRT2 FMO5 FOS FOSB FOSL2 FOXA2 FPGS FPR1 FRAT2 FRZB FSCN1 FTH1 FTO FUBP3 FUT8 FYN FZR1 G0S2 GABARAP GABBR1///UBD GADD45B GBAS GCC1 GCLC GCSH///LOC100329108 GDF1///LASS1 GDF15 GEMIN6 GM2A GNAL GNB2 GNE GNL3L GOLGA8A GOSR1 GP1BB///SEPT5 GPAA1 GPN3 GPR116 GPR183 GPR32 GPR97 GPS2 GRIA1 GRLF1 GRSF1 GTF2F2 GTF2H5 GTPBP1 GTPBP10 GTPBP4 GUK1 GUSBP3 GZMA H3F3B HADH HAMP HAPLN2 HDGFRP3 HEATR1 HEATR6 HERC6 HFE HIST1H2BG HIST1H2BJ HIST1H4C HIST1H4E HIST2H2AA3///HIST2H2AA4 HIVEP2 HLA-DQA1 HLA-DQA1///HLA-DQA2 HLA-DQB1 HLTF HLX HMHA1 HN1L HNRNPA3 HNRNPH1 HOXA5 HOXB2 HPGD HRH2 HS1BP3 HSD11B1 HSP90AA1 HSPA13 HSPA1A///HSPA1B HSPB1 HSPB11 IARS IBTK ICA1 ICAM1 ICAM3 ICK IDI1 IDO1 IER2 IER3 IFI27 IFI44L IFITM1 IFITM2 IFITM3 IFNA1 IGHA1///IGHA2///LOC100126583 IGHG1///IGHG2///IGHM///IGHV4-31 IGJ IGK@///IGKC IGK@///IGKC///IGKV1-5 IGKC IGL@ IGLC7///IGLV1-44///LOC100290481 IGLV1-44///LOC100290481 IL17RA IL18R1 IL18RAP IL1B IL1R1 IL1R2 IL1RN IL21R IL27RA IL2RA IL4 IL7 IL8 IMPDH1 INO80B INPP4A INPP5A INSR IPO5 IPO7 IQCJ-SCHIP1///SCHIP1 IQGAP1 IRAK3 IRF1 ISCA1 ISG20 ITGB4 JAKMIP2 JMJD6 JUN JUNB JUND KANK2 KARS KAZ KCNAB1 KCNB2 KCNJ1 KCNJ15 KCNJ2 KCNN4 KDM5C KDM6B KHDC1L KIAA0040 KIAA0415 KIAA0494 KIAA0776 KIAA0913 KIAA0913///SAT1 KIAA1109 KIAA1704 KIF16B KIF1C KIR3DX1 KL KLF2 KLF3 KLF6 KLHDC10 KLHDC2 KLRB1 KLRK1 KRIT1 KRT17 KRT84 KRTAP1-1 KTN1 LAG3 LAMB1 LAMP1 LARP4 LARS LCAT LCMT2 LEPR LILRA1 LILRA3 LILRB2 LIMK2 LIN7C LIPT1 LMNA LMNB1 LMO4 LOC100128640 LOC100506076///LOC100506123 LOC100507424 LOC100507804///TPSAB1 LOC100508797 LOC100509130 LOC220594 LOC283079 LOC389906 LPCAT1 LRBA LRRFIP1 LRRK1 LSM4 LSS LTF LUC7L3 LY75 LY9 MAD2L1 MAF MAFF MAGEA4 MAGEC3 MAGEL2 MAN1A2 MAP2K2 MAP4 MAPKAPK2 MARCKS MBD4 MBNL2 MCAM MECP2 MED1 MED15 MED22 MED31 MEGF9 MEOX1 METAP1 METTL1 METTL2B MFNG MIIP MKL2 MLL MMD MMP3 MOBKL1B MOCOS MORC3 MOS MOSPD1 MPP2 MPPE1 MPZ MPZL1 MPZL2 MRPL46 MRPL52 MRPS17///ZNF713 MRPS22 MRPS30 MSH3 MSRB2 MTCP1NB MTF1 MTHFD1 MTIF2 MTO1 MTOR MTR MTUS1 MUC5AC MUDENG MUT MXD1 MYCBP MYH3 MYL6 MYLIP MYO3A MYO6 MYST3 N4BP2L1 N6AMT1 NAA35 NAB2 NADK NAT2 NBEAL2 NCAPD2 NCAPG NCF1///NCF1B///NCF1C NCF1C NCOA3 NCRNA00115 NDEL1 NDRG1 NDUFA2 NDUFA4 NDUFA6 NDUFA8 NDUFA9 NDUFC1 NDUFS4 NEDD9 NEK1 NEK4 NF1 NF2 NFATC2IP NFE2 NFKB1 NFKB2 NFKBIA NFKBIE NFS1 NFYC NHP2 NINJ1 NIPBL NLGN1 NLRP3 NME6 NOL10 NOS1 NOTCH1 NPVF NTRK2 NTRK3 NUCKS1 NUDT4 NUP160 NUP188 NUP210 NUP50 NUP88 NXF3 OAT OGDH OLFM1 OPRL1 OPTN OR12D3 OR2J2 OR2W1 ORC5 ORM1 ORM1///ORM2 OS9 OSBPL1A OSM OXR1 OXSM P2RY14 P4HB PABPN1 PAF1 PAIP1 PAK3 PAM PANK3 PAPOLA PAPOLG PARP1 PBXIP1 PCDH11Y PCM1 PCSK6 PDCD6 PDE3A PDE4B PDE7B PDE8A PDLIM5 PDLIM7 PELI1 PELO PER1 PEX14 PEX6 PFKFB3 PGLS PGRMC2 PHACTR2 PHACTR4 PHF20 PHIP PI3 PIGH PIK3C2A PIK3R1 PIM1 PIM2 PISD PITPNA PKM2 PKNOX2 PLCB3 PLCB4 PLEC PLEK PLK3 PLLP PLN PLUNC PLXNC1 PMAIP1 PNMA1 PNRC1 POGLUT1 POLR2E POLR2K POLR3G POT1 PPARA PPARD PPARG PPARGC1A PPCDC PPFIBP2 PPIF PPIG PPIP5K2 PPM1F PPP1CC PPP1R10 PPP1R14B PPP1R15A PPP2R2D PPP2R5A PRB3 PRKACB PRKAR2B PRKCI PRKCSH PRLH PRPF19 PRPF4B PRR4 PRUNE PSD3 PSMB1 PSMD8 PSPH PTER PTGES2 PTGFR PTGS2 PTMS PTP4A2 PTPN22 PTPRF PTPRR PTPRS PVALB PXN PYROXD1 QSOX1 RAB2A RAB35 RAB38 RAB4A RAB4A///SPHAR RAB7L1 RABEP2 RABEPK RABGAP1L RABGGTB RAD51L1 RALBP1 RALGDS RANGAP1 RARA RASA4///RASA4P RASGRP1 RASGRP2 RASSF2 RBL2 RBM25 RBM41 RBM5 RCBTB1 RCN2 RDH11 RECK RECQL RELA RELB REST RFK RFX5 RGS2 RHCG RHD RHOBTB3 RHOH RIF1 RIOK3 RLF RNF8 RNFT2 RNGTT RORA RPA3 RPL10 RPL10A RPL26 RPL29 RPL35A RPL36 RPN1 RPRD1A RPS11 RPS25 RPS4Y1 RPS6KA2 RREB1 RRN3 RSL1D1 RTN2 RUFY3 RYBP S100A12 S100A6 S100A8 S100A9 SAA1///SAA2 SAMD4A SASH1 SBNO2 SCAMP3 SCARF1 SCGB1A1 SCML1 SCRIB SDCCAG1 SDF2L1 SDS SEC14L1 SEC22A SEC61A1 SEC62 SEC63 SECISBP2L SELL SEMA3C SEMA3G SENP5 SERPINB4 SERPINB9 SET SETD6 SF1 SF3A1 SF3B1 SFTPB SGK1 SGMS1 SGSH SH2D1A SH3GL3 SH3YL1 SHB SIGLEC1 SIGLEC6 SKAP2 SKP1 SLC16A3 SLC16A6 SLC19A1 SLC22A3 SLC25A1 SLC25A36 SLC25A37 SLC26A4 SLC35A3 SLC38A2 SLC38A4 SLC39A8 SLC47A1 SLC7A10 SLC7A8 SLCO1A2 SMAD4 SMAD5 SMARCA1 SMARCA2 SMARCAL1 SMC5 SMG1 SMS SNCA SNN SNRPA1 SNRPD2 SNX1 SNX24 SNX4 SNX7 SOAT1 SOCS3 SOD2 SON SORD SORL1 SOX30 SP140 SP2 SPAG8 SPARCL1 SPEN SPICE1 SPIN1 SPIN2A///SPIN2B SPP1 SPTLC2 SQSTM1 SRF SRI SRP72 SRSF6 SSB ST18 ST20 ST6GALNAC5 STAC STAG3L1 STAP1 STAT1 STAT2 STAT3 STAT5B STATH STEAP4 STK25 STK39 STRN4 STS STX3 SUGP2 SUOX SUPT6H SYNCRIP SYNJ2BP SYPL1 SYT1 TACC1 TAF10 TAF11 TAGLN2 TBC1D12 TBC1D5 TBL1X TBRG4 TBXA2R TCEB3 TCF3 TCF4 TEX14 TEX2 TF TFB2M TFCP2 TFIP11 TGFA TGM2 TGM3 TH1L THBS1 THOC2 THSD7A THUMPD1 TIA1 TIMELESS TIMM44 TIMP1 TJP2 TLE3 TLR7 TLX2 TM2D1 TMBIM4 TMED5 TMEFF1 TMEM106B TMEM123 TMEM131 TMEM144 TMEM149 TMEM183A TMEM209 TMEM38B TMEM5 TMEM8A TMEM93 TNFAIP3 TNFAIP6 TNFRSF10C TNFRSF14 TNFRSF1A TNFRSF1B TNFSF10 TNFSF15 TNFSF4 TNIK TNIP1 TNK2 TNPO3 TOM1 TOP1 TOP2B TPD52 TPRA1 TPSAB1 TPSAB1///TPSB2 TPSB2 TRAK2 TRBC1 TRBC2 TRD@ TRIB1 TRIM14 TRIM25 TRIM28 TRIM31 TRIM36 TRIM44 TRIM68 TRIP11 TRMT112 TRNAU1AP TRPC1 TRPC6 TRPM6 TSPAN31 TSPYL4 TSR1 TTBK2 TTC19 TTC3 TTC33 TTC37 TUBB2A TWF1 TXN TXNL4A UBA1 UBAP2 UBASH3A UBE2K UBFD1 UBR5 UBR7 UBXN1 UBXN4 UFSP2 UGCG UGDH UHRF1BP1L UPF3A UQCR11 UQCRQ USP1 USP20 USP29 USP8 UTP18 UTP20 VAMP1 VAMP2 VAMP4 VAV3 VDAC3 VEGFA VHL VNN2 VRK2 WARS2 WASL WBP4 WDR11 WDR12 WDR43 WDR44 WDR61 WNK1 WRB WSB1 WSB2 WWP2 XIST XPNPEP1 XPNPEP3 XPO6 YES1 YIPF2 YKT6 YME1L1 YTHDC2 YWHAQ ZBTB24 ZBTB25 ZBTB7A ZC3H12A ZC3H14 ZDHHC17 ZDHHC18 ZFP161 ZFP36 ZFP36L2 ZFYVE26 ZKSCAN1 ZMYM2 ZMYND11 ZNF155 ZNF157 ZNF217 ZNF24 ZNF264 ZNF271 ZNF273 ZNF276 ZNF304 ZNF329 ZNF44 ZNF443 ZNF451 ZNF518A ZNF652 ZNF702P ZNF813 ZNF83 ZNF91 ZYX | BAL_39_A0B1 BAL_26_A2B0 BAL_40_A0B0 BAL_43_A0B0 BAL_17_A0B0 BAL_13_A0B0 BAL_45a_A1B0 BAL_33_A0B1 BAL_47b_A0B1 BAL_44b_A0B0 BAL_25_A0B0 BAL_37_A1B1 BAL_12_A1B0 BAL_1_A0B0 BAL_18_A1B0 BAL_4_A0B0 BAL_3_A1B0 BAL_27b_A2B1 BAL_15a_A0B1 BAL_27a_A0B0 BAL_45b_A2B0 BAL_46_A2B0 BAL_31_A0B1 BAL_20b_A1B1 BAL_15b_A0B0 BAL_47c_A0B1 BAL_47a_A0B0 BAL_41_A1B0 BAL_21_A0B0 BAL_29_A1B0 BAL_44a_A0B0 NeverSmoker_70 FormerSmoker_72 CurrentSmoker_67 CurrentSmoker_81 NeverSmoker_96 BAL_19_A0B0 FormerSmoker_95 |
| **8** | AASS ABCA7 ABCB6 ABCC2 ABCC5 ABCC9 ABHD5 ABL2 ABLIM1 ABO ACADVL ACAP1 ACD ACVR1B ADAM17 ADCK2 ADCY2 ADCY3 ADD3 ADH1C ADH6 ADH7 ADI1 ADK ADNP2 ADORA2B AGFG1 AGMAT AGPAT1 AHCTF1 AHCY AHI1 AHNAK AHNAK2 AHR AKAP12 AKAP3 ALDH1A1 ALDH3A2 ALDH3B1 ALDH6A1 ALDH7A1 ALDOC ALG13 ALPK1 ALPK3 AMY1A///AMY1B///AMY1C///AMY2A///AMY2B ANAPC13 ANK3 ANKRD12 ANKRD36 ANKRD36B ANKRD36BP2 ANKRD6 ANXA4 ANXA6 ANXA8///ANXA8L1///ANXA8L2 APC APOBEC3F APOL1 APOL2 APP AQP3 AREG ARG2 ARHGAP26 ARHGAP6 ARHGEF10L ARID5B ARL1 ARL17A///ARL17B ARL4C ARMC8 ARSD ASAP1-IT ASCC2 ASCL1 ASCL2 ASMTL ASRGL1 ATM ATP10A ATP11A ATP1A1 ATP1B1 ATP2A2 ATP6V0E1 ATP6V0E2 ATP7A ATP8A1 ATP8B1 ATP8B4 ATXN10 ATXN7 AUTS2 AVIL AZI2 AZIN1 B3GALNT1 B3GALT1 BACH2 BAG1 BAG4 BAG5 BATF3 BAZ1B BBOX1 BBS7 BCAM BCAP29 BCL11A BCL11B BCL2 BCL3 BCLAF1 BGN BLMH BMP5 BNIP3 BNIP3L BPNT1 BRWD1 BST2 BTBD2 BTF3 BTN2A1 BTN2A3 BTN3A2 BZW2 C10orf116 C10orf84 C11orf2 C12orf10 C12orf47 C13orf18 C14orf102 C14orf132 C14orf166 C15orf39 C15orf63///SERF2 C16orf7 C17orf101 C17orf60 C17orf86 C17orf91 C18orf10 C19orf10 C19orf29 C19orf42 C1orf107 C1orf112 C1orf115 C1orf116 C1orf159 C1orf175///TTC4 C1orf77 C20orf111 C20orf117 C21orf33 C22orf9 C2orf18 C3orf14 C3orf32 C3orf63 C4orf46///TOMM7 C6orf120 C6orf130 C6orf48 C8A C8B C8orf33 C8orf4 C9orf16 C9orf31 CA2 CALM1 CAMK1 CAMSAP1L1 CASC3 CASP6 CASP7 CAV1 CAV2 CBX1 CBX5 CBX6 CBX7 CCDC47 CCDC72 CCDC81 CCDC85C CCDC88A CCL20 CCL5 CCL8 CCNB1IP1 CCND2 CCNT1 CCR2 CCR7 CCT3 CD164 CD2 CD200 CD207 CD247 CD27 CD36 CD3D CD3E CD47 CD55 CD6 CD68 CD7 CD8A CD93 CD96 CD99 CDADC1 CDC16 CDC42 CDC45 CDK16 CDK8 CDKN2A CDKN2C CDYL CEACAM5 CELA2A CELSR1 CENPF CENPT CEP135 CEP68 CES1 CFB CFLAR CH25H CHAF1B CHD7 CHD9 CHIT1 CHKB-CPT1B///CPT1B CHP2 CHST15 CIAO1 CIITA CIRBP CIZ1 CLC CLCA1 CLCA2 CLDN18 CLDN8 CLEC16A CLNS1A CLSTN1 CNBP CNOT1 CNOT4 COBRA1 COG8///PDF COIL COL4A3 COL4A5 COL4A6 COL6A1 COL7A1 COL8A1 COMMD8 COPA COPS6 CORO1A COX11 COX16 COX4I1 COX5B COX6C CPEB3 CPPED1 CREM CRYBB2P1 CSF3 CSGALNACT1 CSNK2A2 CSPG4 CST6 CTDSPL CTNNAL1 CTSH CTSK CTSZ CUL5 CWH43 CX3CR1 CXCL13 CXCL14 CXCL2 CXCL3 CXCL5 CXCL6 CXCL9 CXCR2 CXCR3 CXCR6 CXorf27 CYB561D2 CYB5A CYBA CYBRD1 CYC1 CYFIP2 CYLD CYP1B1 CYP24A1 CYP26A1 CYP2A6 CYP2B6 CYP2D6 CYP2F1 CYP2J2 CYP3A5 CYP4A11 CYP4F3 D4S234E DAPP1 DAZ1///DAZ2///DAZ3///DAZ4 DBF4 DBT DCN DDC DDIT4 DDX11///DDX12///LOC642846 DDX17 DDX3Y DDX42 DDX50 DDX58 DDX60 DEFB1 DEFB4A DEPDC6 DEXI DGUOK DHRS11 DHRS12 DHX34 DIAPH2 DIAPH3 DICER1 DIXDC1 DKFZp547G183 DKK3 DMBT1 DMC1 DNAJC1 DNAJC10 DNAJC12 DNAJC2 DNAJC4 DNASE2B DNPEP DOCK10 DOCK6 DPH5 DPP4 DPYSL3 DSPP DST DSTN DTNB DUOX2 DUSP1 DUSP5 DYRK2 EAPP EBAG9 EDA EDC3 EDEM3 EDNRA EDNRB EED EEF1A1 EEF1A1///EEF1A1P9 EEF1B2 EEF1D EEF1G EEF2 EFEMP1 EFEMP2 EFHD1 EFHD2 EFNA2 EFNB3 EGFL6 EGFR EGLN3 EGR2 EGR3 EHBP1 EHD1 EHMT2 EID1 EIF3B EIF3D EIF3E EIF3F EIF3K EIF3L EIF4A1 EIF4B ELF2 ELF5 ELK3 ELN EML2 ENDOD1 ENTPD4 EP300 EPAS1 EPHB6 EPHX1 EPM2A EPM2AIP1 EPOR EPS15L1 ERAP1 ERBB4 ERLIN2 ERO1L ERO1LB ERP44 ESM1 ESRRG ETV6 EVI5 EVX1 EZH1 F13A1 FABP3 FADS1 FAF2 FAIM2 FAIM3 FAM107A FAM117A FAM118A FAM125B FAM129A FAM131A FAM134B FAM168B FAM184A FAM186A FAM20B FAM46A FAM46C FAM50B FAM55C FAM65B FAM69A FAM70A FAM75C2 FAM86B1 FAT2 FAU FBL FBXO11 FBXO4 FBXO42 FCER1A FCGBP FCGR3B FCN1 FFAR3 FGFR2 FGFR3 FKBP9 FKSG49 FLJ13224 FLRT3 FMO2 FNDC3A FOLR1 FOLR3 FOSB FOSL2 FOXB1 FOXG1 FOXL2 FOXN3 FPR1 FRMD4A FRYL FSCN1 FTO FTSJ1 FUBP3 FXC1 FXR1 FXYD1 FYN FZD6 FZR1 G0S2 G3BP1 GABARAP GABBR1///UBD GABRA4 GAD1 GADD45A GADD45B GAGE1///GAGE12C///GAGE12D///GAGE12E///GAGE12F///GAGE12G///GAGE12H///GAGE12I///GAGE12J///GAGE2A///GAGE2C///GAGE2D///GAGE2E///GAGE4///GAGE5///GAGE6///GAGE7///GAGE8 GAGE1///GAGE12F///GAGE12G///GAGE12I///GAGE12J///GAGE2A///GAGE2B///GAGE2C///GAGE2D///GAGE2E///GAGE3///GAGE4///GAGE5///GAGE6///GAGE7///GAGE8 GAGE1///GAGE12F///GAGE12G///GAGE12I///GAGE12J///GAGE4///GAGE5///GAGE6///GAGE7 GAGE12C///GAGE12D///GAGE12E///GAGE12F///GAGE12G///GAGE12H///GAGE12I///GAGE2A///GAGE2C///GAGE4///GAGE5///GAGE6///GAGE7 GAGE12F///GAGE12G///GAGE12I///GAGE5///GAGE7 GAGE3 GAK GALNT6 GAPDH GAPVD1 GART GAS2L1 GAS6 GATAD2A GATM GBP1 GBP2 GCLC GCLM GCN1L1 GDF15 GEMIN4 GFI1 GIPC2 GJA1 GJA4 GK///GK3P GK3P GLP1R GLTSCR2 GLUD2 GLUL GMNN GNA15 GNAS GNB2L1 GNE GNL3L GNLY GOLT1B GPD1 GPD1L GPR116 GPR125 GPR157 GPR171 GPR25 GPR56 GPR75 GPRASP1 GREB1 GRP GSTA1 GSTA3 GSTA4 GSTM1 GSTM2 GSTT1 GTF2A1L///STON1-GTF2A1L GTF2H1 GTF2H2///GTF2H2B///GTF2H2C///GTF2H2D GTF2H3 GTPBP1 GTPBP10 GUCY1B2 GULP1 GYPE GZMA GZMB GZMK H1F0 H1FX H2AFX H2BFS H3F3B HAB1 HAUS4 HAX1 HBP1 HCRTR2 HDAC5 HDGFRP3 HERC6 HEY2 HFE HHLA3 HINT1 HIST1H2BG HIST1H2BK HIST1H3C HIST1H4C HIVEP1 HK2 HLA-B HLA-C HLA-DQA1 HLA-DQB1 HLA-DRB4 HLA-DRB6 HLA-E HLA-F HLA-G HLF HMBOX1 HMGB1 HMGN3 HNMT HNRNPA0 HNRNPA1 HNRNPA1///HNRNPA1L2///HNRNPA1P10 HNRNPA1///HNRNPA1L2///HNRNPA1P10///LOC728643 HNRNPC HNRNPD HNRNPH1 HNRNPU HNRNPUL1 HOPX HOXB6 HP HP///HPR HRH4 HS3ST3A1 HSD17B8 HSPA2 HTR1D HTR2C ICOS IDO1 IFI27 IFI30 IFI35 IFI44 IFI44L IFI6 IFIH1 IFIT1 IFIT3 IFITM1 IFITM3 IFNA14 IGBP1 IGF1 IGF2BP3 IGFBP2 IGFBP4 IGFBP5 IGFBP7 IGHA1///IGHG1///IGHG3///IGHM///IGHV4-31///LOC100510678 IKBKAP IKZF1 IL13RA1 IL17RA IL17RB IL1A IL1B IL1R1 IL1RN IL22RA1 IL24 IL2RB IL33 IL6 IL7 IL8 IMPDH2 INF2 ING1 INHA INHBA INSIG1 IPO5 IPO9 IRAK3 IRAK4 IRF1 IRF7 IRS2 ISCA1 ISG15 ISL1 ISLR ITGA2B ITGB1 ITGB1BP1 ITGB5 ITGB6 ITGBL1 ITIH4///MUSTN1 ITIH5 ITK ITM2A ITM2C ITSN1 JAG2 JAKMIP2 JHDM1D KAL1 KCNA3 KCNAB1 KCNJ1 KCNJ12///KCNJ18///LOC100290070 KCNJ16 KCNJ2 KCNJ5 KCNJ6 KCNS3 KCTD12 KCTD7///RABGEF1 KDM3B KDM5B KDR KDSR KIAA1024 KIAA1033 KIAA1731 KIF16B KIF20A KIF23 KL KLF4 KLHDC2 KLHL20 KLHL22 KLHL24 KLK11 KLK13 KLRB1 KLRC1///KLRC2 KLRD1 KLRF1 KLRG1 KLRK1 KMO KPNA1 KRAS KRT15 KRT4 KRT5 KRT6A KRT6A///KRT6B///KRT6C KRT6B KSR1 KTN1 KYNU L1TD1 LAG3 LAMB1 LANCL1 LAP3 LARP4 LARP6 LCK LDHB LDLR LDLRAP1 LEF1 LETM1 LGALS2 LGALS8 LHX2 LIG4 LILRA2 LILRA6///LILRB3 LIMK1 LMNA LMO2 LMO4 LNPEP LOC100127972 LOC100287927 LOC100505503///RPS17 LOC100505584///MT1E LOC100506076///LOC100506123 LOC100507328///LOC100508591 LOC100507804///TPSAB1 LOC100507851///PRAMEF1///PRAMEF2 LOC100510735///RPL29 LOC150759 LOC282997 LOC285359///PDCL3 LOC285830 LOC51152 LOC730101 LONP2 LRFN4 LRIG1 LRMP LRP12 LRP8 LRPPRC LRRC42 LRRFIP1 LSG1 LSM14B LST1 LTBP3 LTF LYZ MAFF MAGEH1 MAGEL2 MAGI2 MAN1C1 MANEA MAOB MAP2K6 MAP7 MARCKS MASP2 MATR3 MB MBNL1 MCAM MCC MCF2L2 MCM4 MDC1 MDFIC MDM2 MECP2 MEF2C MEGF9 MEIS3P1 METAP1 METRN METTL7A MFAP3L MGC13053 MIA MICAL2 MICALL1 MIOS MKKS MKL2 MKRN1 MKRN3 MLF1 MLL MLLT4 MMP1 MMP10 MMP13 MMS19 MOG MPHOSPH8 MPHOSPH9 MR1 MRFAP1L1 MRPL28 MRPL40 MRPL9 MS4A1 MSL3 MST1P9 MT1F MT1G MT1H MT1P2 MT1X MT3 MTCP1NB MTHFD2 MTMR2 MTRF1L MTSS1 MUC13 MX1 MX2 MXI1 MXRA7 MYH1 MYH10 MYH7 MYL6B MYO1B MYO7B MYOZ2 MYST3 MZT2B N4BP2L1 NACA NADSYN1 NAMPT NAP1L1 NBPF10 NCAM1 NCL NCR1 NCRNA00094 NDRG1 NDRG4 NDUFA1 NDUFA10 NDUFA6 NDUFC2 NDUFS4 NEAT1 NELL2 NETO2 NF1 NFATC3 NFIB NFIC NFX1 NID1 NINL NISCH NLGN1 NLRP3 NMBR NMT2 NOL11 NPAT NPEPPS NPFF NPIPL3 NPM1 NPRL3 NR2F1 NR3C2 NR4A1 NR4A2 NR4A3 NRL NSA2 NSFL1C NTS NUBPL NUCKS1 NYNRIN OAS1 OAS2 OAS3 OAZ2 OBFC1 OGT OLFML2B OPCML OR2A20P///OR2A9P OR2H2 OR2J2 OR2S2 OR6A2 ORAI3 ORC3 ORC5 ORM1 ORM1///ORM2 OSBPL10 OSBPL3 OXTR P2RX1 P2RY11///PPAN-P2RY11 PABPC4 PAK6 PAQR5 PARP12 PAWR PAX8 PBRM1 PBX1 PBX2 PBXIP1 PCCA PCDH7 PCGF2 PCGF3 PCNX PCNXL2 PCSK1 PCSK2 PCSK7 PDAP1 PDCD4 PDCL PDE4B PDE4DIP PDE6H PDE8A PDGFA PDK2 PDLIM5 PDS5A PDZK1IP1 PEBP1 PER3 PERP PEX11A PFDN5 PGC PGRMC1 PHACTR1 PHACTR2 PHF1 PHF14 PHF8 PHIP PHLDA2 PHYHIP PI3 PIAS2 PIK3CB PIK3CG PIK3IP1 PIK3R1 PIN4 PIP PISD PITPNC1 PKN2 PKP1 PKP4 PLA2R1 PLAC4 PLAGL1 PLAGL2 PLCB3 PLCG1 PLD1 PLEKHA5 PLGLB1///PLGLB2 PLK1S1 PLK2 PLK4 PLP2 PLS3 PLSCR1 PLSCR3 PLTP PLUNC PLXNA1 PMAIP1 PML PMP2 PMVK PNLIPRP2 PNPLA3 POGZ POLDIP3 POLH POLR1D POLR2C POLR3B PON3 POSTN POU2AF1 PPIA PPIC PPIE PPIH PPL PPP1R16B PPP2R3A PPPDE1 PRB3 PRB4 PRDX6 PRF1 PRKAB1 PRKCA PRKCB PRKX///PRKY PRNP PROS1 PRPF8 PRPS1 PRPSAP1 PRR4 PRR5 PRSS21 PRUNE PSG9 PSIP1 PSMB10 PSMB7 PSMB9 PSME2 PSME4 PTBP2 PTDSS1 PTEN PTGER2 PTGFR PTGS2 PTHLH PTN PTP4A1 PTPN1 PTPN11 PTPN12 PTPN20A///PTPN20B PTPN21 PTPRH PTPRZ1 PTRF PTX3 PUM2 PURA PVRIG RAB3B RAB3GAP2 RAB40B RAB5C RABGAP1 RABGAP1L RABL3 RAD52 RAPGEF2 RASAL2 RASGRP1 RBBP5 RBM14///RBM4 RBM23 RBM25 RBM4B RBMS3 RBP1 RECQL REL REPS1 RETSAT REV1 RFC1 RFC5 RFTN1 RFX7 RGL1 RGS12 RGS17 RGS2 RGS4 RHOQ RIF1 RIMS2 RMI1 RNASE1 RNASEH1 RND2 RNF113A RNF114 RNF187 RNF40 RNF8 RNFT1 RNFT2 ROS1 RPIA RPL10 RPL10A RPL11 RPL12 RPL13 RPL13A RPL13A///RPL13AP5///RPL13AP6 RPL14 RPL15 RPL17 RPL18 RPL18A///RPL18AP3 RPL19 RPL21 RPL22 RPL23 RPL23A RPL24 RPL26 RPL27 RPL27A RPL28 RPL29 RPL3 RPL30 RPL31 RPL32 RPL34 RPL35 RPL35A RPL36 RPL36A RPL37 RPL37A RPL38 RPL39 RPL4 RPL5 RPL6 RPL7 RPL7A RPL8 RPL9 RPLP0 RPLP0///RPLP0P6 RPLP1 RPLP2 RPS10 RPS10///RPS10P7 RPS11 RPS12 RPS13 RPS14 RPS14P3 RPS15 RPS15A RPS16 RPS17 RPS18 RPS19 RPS2 RPS20 RPS21 RPS23 RPS24 RPS25 RPS26 RPS27 RPS27A RPS28 RPS29 RPS3 RPS3A RPS4X RPS5 RPS6 RPS6KA5 RPS7 RPS8 RPS9 RPSA RRAS2 RREB1 RRM1 RRN3 RRP15 RRP8 RS1 RSAD1 RTEL1 RTEL1///TNFRSF6B RTP4 RUNX1 RUNX3 RWDD1 RYR1 RYR3 S100A8 S100A9 SAA1///SAA2 SAA4 SAMHD1 SARS SATB1 SBF1 SBNO2 SCAMP1 SCGB1A1 SCN11A SCN2A SCO2 SDF2 SDHAF1 SDHC SEC14L3 SEC22A SEC24D SECTM1 SEL1L SEMA5A SEMG1 SEPP1 SEPT11 SEPT4 SERP1 SERPINA1 SERPINB1 SERPINB3 SERPING1 SET SETBP1 SETD5 SF3A3 SF3B2 SF3B3 SFTPA2 SFTPB SFTPC SFTPD SH2B2 SH2D1A SH3BP2 SH3BP4 SHANK2 SIAH1 SIK1 SIX2 SKAP1 SKP1 SLC13A1 SLC13A3 SLC15A2 SLC17A9 SLC1A1 SLC22A4 SLC23A2 SLC24A1 SLC25A12 SLC25A31 SLC25A6 SLC26A4 SLC27A2 SLC2A6 SLC30A1 SLC35E2B SLC35F5 SLC37A1 SLC38A1 SLC39A8 SLC4A7 SLC6A2 SLC9A2 SLCO3A1 SLK SMA4 SMAD4 SMAD5 SMAD6 SMARCA2 SMARCA4 SMARCC1 SMEK2 SMPDL3A SMU1 SMYD3 SNAI2 SNCA SNED1 SNRNP200 SNRPD2 SNRPN///SNURF SNX7 SOCS3 SOD2 SORD SOX11 SP110 SP140L SPARCL1 SPATA2L SPATS2L SPG21 SPINLW1 SPN SPOCK2 SPRR1A SPRR1B SPRY1 SPTBN1 SQLE SRD5A1 SRGAP3 SRGN SRP14 SRPK1 SRSF6 SS18 SSBP1 SSBP2 SSBP3 SSH3 ST20 ST3GAL2 ST3GAL6 STAG3 STAM2 STARD8 STAT1 STAT2 STATH STEAP1 STEAP3 STK16 STK17A STK3 STMN2 STS STX11 SUCLG1 SULT1A1 SULT1A2 SULT1A3///SULT1A4 SUSD4 SUZ12P SYNE1 SYNE2 SYNGR3 SYNJ2 SYNM SYPL1 SYT1 TACC1 TACO1 TADA3 TAF6 TAF6L TAF7 TAF9B TANC2 TAOK2 TAP1 TAPBPL TAPT1 TBC1D13 TBC1D30 TBX1 TBX10 TBXA2R TCEA1 TCEA2 TCHH TCIRG1 TF TFB2M TFCP2 TFEB TFIP11 TFPI TGM2 THAP10 THBS1 THBS4 THOC7 THYN1 TIAL1 TIMP3 TLE1 TLR5 TM2D1 TM4SF20 TMCO6 TMED5 TMED7 TMEFF1 TMEM106B TMEM131 TMEM144 TMEM14A TMEM156 TMEM19 TMEM204 TMEM53 TMEM63A TMEM80 TMEM97 TMSB10 TNFAIP2 TNFAIP3 TNFRSF10B TNFRSF10C TNFRSF9 TNFSF14 TNFSF8 TNIP3 TNNI2 TNNI3 TNS1 TOMM20 TOP2B TOX TP53TG1 TP63 TPSAB1 TPSAB1///TPSB2 TPSB2 TPT1 TRAC TRAC///TRAJ17///TRAV20 TRAF5 TRAF6 TRBC1 TRBC1///TRBC2 TRD@ TRDV3 TRHDE TRHR TRIB1 TRIM10 TRIM14 TRIM22 TRIM31 TRIO TRMT2B TRPC2 TRPV4 TRRAP TSC22D1 TSC22D2 TSPAN6 TSPAN9 TSPYL1 TSPYL4 TSPYL5 TST TTC17 TTC18 TTF2 TUBA4A TUBB4Q TUBD1 TUBGCP4 TYMS UBA52 UBAP2L UBASH3A UBE2D2 UBE2H UBE2I UBE2J1 UBE2L6 UBL3 UBL4A UBQLN4 UBR7 UCHL1 UGCG UGT1A1///UGT1A10///UGT1A3///UGT1A4///UGT1A5///UGT1A6///UGT1A7///UGT1A8///UGT1A9 UGT1A1///UGT1A10///UGT1A4///UGT1A6///UGT1A8///UGT1A9 UGT2A1///UGT2A2 UNC45A UPF3A UQCRB USH1C USP13 USP18 USP27X USP34 USP36 USP6NL UTP14A VAMP1 VANGL1 VAV3 VDAC3 VEGFA VENTXP1 VEZF1 VEZT VKORC1 VNN1 VNN2 VPS13B VPS53 VPS72 VTCN1 VWA5A WARS WBP4 WDR59 WFDC8 WHAMML1///WHAMML2 WIZ WSB1 WSB2 XAF1 XCL1 XPC XPNPEP1 XRCC2 XRCC4 XYLB YARS2 YBX1///YBX1P2 YIPF3 YTHDC2 ZC3H12A ZCCHC11 ZCCHC14 ZCCHC4 ZDHHC4 ZFAND5 ZFP36 ZFP36L1 ZFPL1 ZFR ZFX ZHX3 ZMAT3 ZMYM5 ZMYND8 ZNF155 ZNF160 ZNF167 ZNF189 ZNF22 ZNF221 ZNF224 ZNF23 ZNF250 ZNF263 ZNF264 ZNF318 ZNF330 ZNF623 ZNF702P ZNF721 ZNF780A///ZNF780B ZNF814 ZSCAN18 | BAL_46_A2B0 FormerSmoker_18 BAL_19_A0B0 BAL_17_A0B0 BAL_42_A0B0 BAL_21_A0B0 BAL_18_A1B0 BAL_27b_A2B1 BAL_43_A0B0 CurrentSmoker_54 BAL_47b_A0B1 BAL_26_A2B0 BAL_33_A0B1 BAL_20a_A1B1 BAL_44a_A0B0 BAL_31_A0B1 BAL_4_A0B0 BAL_40_A0B0 BAL_44b_A0B0 BAL_25_A0B0 BAL_1_A0B0 BAL_3_A1B0 BAL_39_A0B1 BAL_12_A1B0 BAL_15a_A0B1 BAL_45a_A1B0 BAL_47c_A0B1 BAL_13_A0B0 BAL_27a_A0B0 BAL_47a_A0B0 BAL_15b_A0B0 BAL_41_A1B0 BAL_29_A1B0 BAL_37_A1B1 BAL_20b_A1B1 CurrentSmoker_37 NeverSmoker_38 CurrentSmoker_12 FormerSmoker_69 CurrentSmoker_29 BAL_45b_A2B0 CurrentSmoker_36 NeverSmoker_52 FormerSmoker_27 |
| **9** | ABAT ABCA1 ABCB1 ABCD2 ABHD2 ABLIM1 ABO ACIN1 ACSM1 ADAM20 ADAM22 ADAMTS3 ADAMTS5 ADCK2 ADCY3 ADH1B ADORA1 ADORA2A///SPECC1L AFF1 AGGF1 AGT AGXT AHNAK AIMP2 AKAP17A AKAP2///PALM2-AKAP2 AKT2 ALDH5A1 ALG3 ALPK3 ANKRD36 ANKRD36B ANKRD6 ANXA10 AP1S1 APC AQP4 ARAP1 AREG ARF4 ARGLU1 ARHGAP32 ARHGAP5 ARID3A ARID4B ARL4C ARMC8 ASB13 ASB4 ASPHD1 ATF3 ATP2A2 ATP5SL ATP6V0A2 ATP6V1G2 ATP6V1G2///BAT1 ATP8A2 ATRX ATXN8OS AVPR1A AXIN1 BAIAP2L2 BANF1 BAP1 BAT2 BAT2L2 BAZ1A BAZ2B BBS7 BBX BCHE BCL2L11 BCL3 BCLAF1 BEST1 BEST2 BIN1 BIRC2 BIRC5 BMP2 BMP6 BPI BPTF BRWD1 BUB1 C10orf18 C12orf10 C14orf139 C16orf42 C17orf101 C17orf91 C18orf25 C19orf2 C19orf29 C19orf42 C1orf107 C1orf144 C1orf63 C1orf9 C20orf103 C20orf117 C2orf24 C3orf37 C4BPB C4orf31 C6orf145 C6orf35 C6orf54 C7orf58 C9orf16 CA12 CA5BP CACYBP CALD1 CALM1 CALR CAMKK2 CAPRIN2 CASP2 CASR CAV1 CBWD1///CBWD2///CBWD3///CBWD5///CBWD6///CBWD7///LOC100507355 CCDC86 CCKAR CCL1 CCL13 CCL16 CCL2 CCL22 CCL4 CCL8 CCNF CCR2 CD36 CD6 CD7 CD84 CDC20 CDC42BPA CDH10 CDHR5 CDK2 CDK5 CDSN CEACAM3 CECR1 CENPB CEP135 CEP290 CEP350 CHCHD7 CHKB-CPT1B///CPT1B CHM CHODL CHRNA6 CHST15 CIDEA CIITA CLC CLCN3 CLDN18 CLEC10A CLEC11A CLEC2D CLEC4M CLK4 CLOCK CLPP CNOT4 CNP CNTF///ZFP91///ZFP91-CNTF COBLL1 COL2A1 COL3A1 COL4A1 CORT CR1 CREBZF CREM CRIM1 CRYBB2///CRYBB2P1 CSAG2///CSAG3 CSE1L CSGALNACT1 CSH1 CSH2 CSNK1G3 CST1 CTAGE1 CTNS CTRB2 CUL3 CX3CR1 CXCL13 CXCL2 CXorf27 CYP1A1 CYP1B1 CYP24A1 CYP4F2 CYR61 DCLRE1A DDIT3 DDX11///DDX12///LOC642846 DDX17 DDX24 DDX3Y DDX5 DEFA6 DHCR7 DHRS2 DHRS7 DHX15 DIAPH2 DICER1 DKFZp547G183 DLST DLX5 DNAJB14 DNAJC22 DOC2A DPYS DSCR4 DST DUOX2 DUSP2 DUSP5 DUSP6 E2F3 EDNRB EFHC2 EFNB1 EHMT1 EIF1 EIF3C///1EIF3CL EIF5A ELK1 ELK3 ELOVL6 EMP1 EMR2 EN2 ENDOD1 ENO2 ENOSF1 ENOX1 EP300 EPAG EPB41L2 EPB41L4A EPM2AIP1 ERC2 ERCC4 ERI3 ESR1 EVC EXOC7 EXOSC8 F11R F12 FADS1 FAF1 FAM120A FAM129A FAM134A FAM155A FAM64A FAM8A1 FASTKD2 FBN2 FBXL15 FCAR FCGR2B FCN1 FCN2 FDFT1 FDPS FERMT2 FGB FGF18 FGF22 FGFR2 FHOD3 FLRT2 FLRT3 FMO1 FNBP4 FOSB FOXA2 FOXJ3 FOXRED2 FZD1 FZD5 G0S2 GABRA4 GABRB3 GALR2 GAS1 GATM GCH1 GDAP2 GDF15 GEMIN4 GFPT2 GHRHR GHSR GLG1 GLT8D2 GNAT1 GNG11 GNLY GOLGA1 GOLGA2 GOLGA6L4///PML GOLGA8A GOLGA8B GOLGA8H GPC4 GPN2 GPR176 GPR31 GPR52 GPR89A///GPR89B///GPR89C GPRC5D GPX4 GRAMD3 GREB1 GREB1L GRK5 GRM6 GRSF1 GSN GSPT2 GSTM3 GTDC1 GTF2A1 GTF2A1L///STON1-GTF2A1L GTF2H2B GTPBP10 GTSE1 GUCA1A GUCY1B3 GZMA GZMH HAS2 HBA1///HBA2 HBB HBS1L HCG4 HERPUD1 HGF HIGD1B HIGD2A HIPK2 HIST1H1D HIST1H2BE HIST1H2BF HIST1H3C HLA-DOA HMGN1 HNF4A HNRNPD HNRNPH1 HNRNPH3 HNRNPU HOPX HOXA11 HOXB5 HOXD13 HRASLS2 HRH3 HSD17B12 HSF2 HSPC159 HTR2C HTR7P1 HYAL4 ICAM1 ICAM3 IER3 IFNA1 IGF1 IGF2BP3 IGHA1///IGHG1///IGHG2///IGHG3///IGHM///LOC100126583///LOC100290036 IGHG1///IGHM///LOC100133862 IGL@ IL12A IL19 IL1R1 IL1R2 IL1RL1 IL2RA IL3 IL6ST IL7 IL8 INADL ING2 ING3 INSL6 IRGC ITGB5 ITK ITM2A ITM2B ITSN1 IVD IVL IVNS1ABP JAKMIP2 JHDM1D JUN KAT2B KAT5 KATNB1 KBTBD11 KBTBD4 KCNAB1 KCNE1 KCNJ15 KCNJ5 KCNMA1 KCNQ1 KDM2A KDM6B KIAA0368 KIAA0509 KIAA0649 KIAA0776 KIAA0907 KIAA0913///SAT1 KIAA1024 KIAA1109 KIAA1539 KIF14 KIF4A KIR3DS1 KLF2 KLF8 KLHDC10 KLHDC2 KLHL23 KLK3 KPNA6 KPNB1 KPTN KRAS KREMEN2 KRT12 KRT37 KRT6A KRTAP1-3 L3MBTL1 LAMA4 LARP1 LEPR LHPP LILRB4 LIM2 LIMCH1 LMO3 LOC100170939 LOC100287927 LOC100505650 LOC100507851///PRAMEF1///PRAMEF2 LOC100509749 LOC26102 LOC51152 LOC653513///PDE4DIP LOC91316 LONP2 LOX LOXL2 LPAR1 LPHN3 LRP1 LRRC23 LTB4R LUC7L3 LUZP1 LY9 LYPD1 LYST MAB21L2 MAGEA4 MAL MAP2K7 MAP9 MAPK8 MARCKS MBP MCPH1 MED13 MED14 MED23 MEG3 MERTK METAP2 METTL5 MFN1 MGAT2 MGC12488 MID2 MKI67 MKRN2 MLC1 MLL2 MMP26 MMP27 MOBKL3 MOSC2 MPHOSPH8 MPPE1 MPRIP MPZ MRPL20 MRPL44 MRPS11 MSH6 MSL1 MTHFSD MTUS1 MUC2 MUT MYCBP2 MYL10 MYO1A MYO6 MYRIP MYST3 MYST4 NACA2 NBPF10 NBPF10///NBPF11///NBPF12///NBPF15///NBPF16///NBPF24///NBPF8///NBPF9 NCAM1 NCAPD2 NCLN NCOA2 NCR3 NCRNA00081 NDUFS8 NEAT1 NEBL NEDD9 NEK1 NEK7 NEK9 NF2 NFAT5 NFATC3 NFKB2 NKTR NLRP3 NLRX1 NMBR NOL12 NOS3 NOTCH2NL NPEPPS NPFF NPY NR2E1 NR4A2 NR4A3 NRXN1 NRXN2 NSUN6 NTRK1 NTRK3 NTSR2 NUP155 NUP50 NUP98 OGFR OLFML3 ONECUT1 OR2B2 OR2J2 OR7E19P ORM1 ORM1///ORM2 OSBPL2 OSBPL3 OSBPL7 OTC OTUD3 OXTR P2RY14 PADI4 PAFAH1B3 PAFAH2 PALLD PAPSS1 PCBP2 PCDH8 PCDHA2 PCDHGA3 PCDHGC3 PCIF1 PCLO PDCD6 PDE4A PDE4C PDE4D PDE4DIP PDGFA PDGFB PDLIM4 PDLIM5 PELI1 PELI2 PEX11A PEX3 PFDN4 PFDN6 PGA3///PGA4///PGA5 PGLS PGPEP1 PGS1 PHC1 PHF11 PHF20L1 PHKA2 PI15 PI4K2A PIGG PIK3C3 PIK3R1 PITPNC1 PKD2L2 PKLR PKNOX2 PKP4 PLA2G2E PLCB1 PLK4 PLXNA2 PLXNC1 PMAIP1 PMS1 PNLIPRP2 PNN POLDIP2 POLI POLR3E POLR3G POMGNT1 POP5 POU3F1 PPARD PPIA PPIG PPP1R15A PPP2R1B PQBP1 PRDM5 PRKCI PRKD1 PRM2 PRNP PROS1 PROX1 PRR4 PRUNE PSG2 PSMD5 PSPH PTGDS PTGER2 PTGIS PTGS2 PTPLB PTPN21 PTPN4 PTPRD PURA PWP1 R3HDM1 RAB11FIP3 RAB27A RAB3GAP1 RAB7A RALGAPB RANBP3 RANBP6 RAPGEF2 RASA3 RASGRF1 RASGRP1 RASSF1 RASSF2 RB1CC1 RBL1 RBM19 RBM22 RBM39 RBM5 RBMY2FP RCN3 REEP4 REEP5 REV1 RFPL1S///RFPL3S RGS1 RGS2 RGS6 RGS7 RIF1 RIMS3 RIT2 RNASEH2A RNF103 RNF115 RNF138 RNF185 RNF19A RNF24 RNF8 RPL14 RPL23AP53 RPL28 RPS4Y1 RRS1 RUNX1T1 RYBP S100A12 S100A5 S100A8 SCAMP1 SCAND2 SCEL SCLY SCNN1A SCO2 SCT SEC14L1 SEC24D SEC62 SECISBP2L SEL1L3 SELL SENP3 SERP1 SERPINB13 SERPINB9 SERPIND1 SF3A1 SFRS18 SFTPA2 SFTPB SFTPC SH3BP2 SHOX SIK3 SKIL SLC10A3 SLC15A1 SLC16A1 SLC16A7 SLC17A3 SLC22A3 SLC25A36 SLC26A4 SLC2A3 SLC2A4 SLC30A1 SLC30A4 SLC35C1 SLC35E2B SLC38A4 SLC38A7 SLC39A6 SLC39A8 SLC5A3 SLC5A4 SLC6A12 SLC7A11 SLC7A6 SLC8A2 SLC9A3 SMA4///SMA5 SMAD5 SMARCC1 SMC3 SMCHD1 SMPD1 SMUG1 SNN SNRK SNTB2 SNX19 SOD2 SON SORBS1 SORL1 SOX13 SPAG11B SPANXB1///SPANXB2///SPANXF1 SPN SPON1 SPP1 SPRR1A SPRR1B SPTAN1 SR140 SRC SREBF2 SREK1 SRF SRPK2 SRRM2 SRSF2 SRSF3 SRSF5 SRSF6 SS18L1 SSTR5 SSX2IP ST6GALNAC4 STAB1 STEAP4 STK17A STK3 STS SULT2A1 SUV420H1 SYNGR1 SYNM SYNPO2L SYT5 TAB2 TBC1D13 TBC1D29 TBP TBPL1 TBX10 TCEB3 TCF7 TCL6 TDRKH TEX28 TF TFCP2L1 TFE3 TFR2 TGM5 THAP9 THG1L THY1 TIAM2 TICAM1 TLE2 TMCC1 TMEM106C TMEM45A TMEM63A TMF1 TNFAIP3 TNFAIP6 TNFRSF10C TNFRSF4 TNIP1 TNKS TNPO1 TNS1 TOB1 TOP2A TOX TOX4 TP63 TPM1 TPR TRA2A TRA2B TRAPPC10 TRIB1 TRIOBP TROAP TRPM1 TRPV5 TSPAN2 TSPY1 TSR1 TTC19 TTC28 TTC3 TTF1 TTLL4 TTTY2 TUB TYMP UBE2L3 UBXN4 UFSP2 UROD USE1 USP34 USP39 USP4 USP46 USP6NL VAMP1 VCAN VCP VDAC3 VEGFA VENTXP1 VGLL1 VPRBP VPS13C VPS53 WBP4 WDFY3 WDR33 WIF1 WIPI1 WIZ WSB2 WWC3 XCL1///XCL2 XYLB YARS2 YTHDC1 ZBTB38 ZC3H15 ZC3H7A ZDHHC11 ZDHHC17 ZFAND3 ZFR ZFR2 ZMYM2 ZMYND8 ZNF117 ZNF134 ZNF140 ZNF177 ZNF222 ZNF225 ZNF23 ZNF238 ZNF266 ZNF273 ZNF274 ZNF34 ZNF358 ZNF407 ZNF451 ZNF510 ZNF592 ZNF638 ZNF667 ZNF673 ZNF696 ZNF79 ZNF814 ZNF835 | BAL_39_A0B1 BAL_17_A0B0 BAL_45b_A2B0 BAL_4_A0B0 BAL_26_A2B0 BAL_18_A1B0 BAL_43_A0B0 BAL_3_A1B0 BAL_40_A0B0 BAL_20b_A1B1 BAL_42_A0B0 BAL_33_A0B1 BAL_13_A0B0 BAL_47b_A0B1 BAL_37_A1B1 BAL_25_A0B0 BAL_15a_A0B1 BAL_44b_A0B0 BAL_45a_A1B0 BAL_27b_A2B1 CurrentSmoker_37 BAL_27a_A0B0 BAL_12_A1B0 FormerSmoker_34 BAL_1_A0B0 BAL_47a_A0B0 BAL_29_A1B0 BAL_15b_A0B0 BAL_31_A0B1 BAL_47c_A0B1 BAL_41_A1B0 BAL_44a_A0B0 NeverSmoker_38 |
| **10** | ABCA4 ABHD2 ABHD8 ABO ACAP1 ACTL6B ADAMTS5 ADCK2 ADCY3 ADK ADM ADRA1D AGA AGXT AKAP8L AKAP9 ALMS1 ALOX5 ALPK1 ALPP///ALPPL2 AMPD2 AMPD3 ANK1 ANKRD12 ANKRD36B ANKRD36BP2 ANKRD5 ANP32A///ANP32D AP1S1 APLP2 APOBEC3G AQP4 ARL17A///LOC100294341 ARPC4 ARTN ATF6B ATM ATP5C1 ATP6V0E1 ATP8B1 ATXN7L1 B2M BASP1 BAT2L2 BCL11B BCL2L11 BCL3 BCL6 BIN1 BLVRB BPTF BRSK2 BTBD2 BTF3P11 BTG1 BTG2 C11orf51 C14orf101 C14orf105 C14orf139 C14orf156 C15orf39 C18orf1 C18orf10 C19orf2 C19orf26 C19orf6 C4orf29 C6orf54 C8G C9orf114 CA5BP CACNA1A CAMK2B CAPZB CASP4 CC2D1A CCDC76 CCK CCL3///CCL3L1///CCL3L3 CCL4 CCL5 CCL8 CCNE1 CCNL1 CCT6A CD27 CD28 CD3D CD55 CD7 CDC5L CDCP1 CDK16 CDK19 CDK2AP2 CDK9 CDKN1A CDKN2D CEACAM5 CELA3A CELF1 CEP350 CEP76 CFLAR CHCHD8 CHI3L1 CHST15 CLDN14 CLEC4E CLIC3 CLN8 CMTM6 CNOT4 COL6A1 COQ3 COX5B COX6C COX7C CPT2 CRTC1 CSF1 CSF3R CST7 CSTF2T CTSB CTSZ CUX1 CWC25 CX3CR1 CXCL1 CXCL11 CXCL13 CXCR2 CXorf21 CYP1A2 CYP1B1 CYP2C9 CYP2E1 CYP3A4 CYP4F8 DAPK3 DAPP1 DAZ1///DAZ2///DAZ3///DAZ4 DBT DCK DCLRE1C DDT DDX28 DDX3Y DDX5 DDX54 DEF6 DEFB1 DGKA DGKQ DHODH DICER1 DIP2A DNAJC4 DNTTIP2 DOK3 DOT1L DPP4 DRD3 DSG1 DUSP1 DUSP5 DYRK2 E2F2 ECSIT EDNRB EEA1 EEF1A2 EEF1D EGR3 EHD1 EIF1 EIF4A1 ELN EMR2 EPB41 EPOR EPS8L1 ERCC6L ERN2 ESRRB ETV1 EXOG EXOSC5 F11R FAIM3 FAM115A FAM129A FAM162A FAM49B FAM86C FANCE FBXO5 FBXW12 FCER1A FCGR3B FER1L4 FFAR2 FGF8 FGFR1 FKSG49 FLCN FLRT2 FLT3LG FOSL2 FOXB1 FOXO3///FOXO3B FPR1 FUT9 FXR1 FYCO1 FZD8 G0S2 GADD45B GADD45G GADD45GIP1 GALK2 GAS1 GAS7 GATAD1 GBP1 GCNT2 GDF15 GFI1B GIMAP6 GJA1 GJA3 GK GLG1 GLRA3 GLUL GNG11 GNLY GOLGA2 GOLGA4 GPN1 GPN3 GPR171 GPR27 GPR97 GPS1 GPT GRIA3 GRIK5 GRIN1 GRIN2D GTF2F1 GTF2H3 GTSE1 GUK1 GUSBP3 GZMA GZMB GZMH GZMK GZMM H3F3A///LOC440926 H3F3B HAUS2 HCG2P7 HDAC2 HDAC6 HES2 HIPK1 HIST1H1E HIST1H2AG HLA-A HNF4A HNRNPD HNRNPH1 HOMER3 HOPX HOXB7 HOXD10 HOXD3 HR44 HRH2 HSD11B1 HSF1 HSPA1A HSPA1A///HSPA1B HTR7 HTR7///HTR7P1 HUWE1 ICAM1 ICOSLG ICT1 ID2///ID2B IER3 IFITM2 IFNA14 IFNA16 IFNAR2 IGHA1 IGHA1///IGHG1///IGHG2///IGHG3///IGHM///LOC100126583///LOC100290036 IGHG1///IGHM///LOC100133862 IGHG1///ZCWPW2 IGKV1-5 IK///TMCO6 IL12RB1 IL1A IL1B IL1R2 IL1RN IL2RA IL32 IL6R IL8 IPO5 IREB2 IRF5 IRGC ITGA4 ITK ITM2A ITM2B IVNS1ABP JAK3 JUNB KCNAB1 KCNE1 KCNF1 KCNJ15 KCNJ2 KCNK12 KDM2A KDM6B KDSR KIAA0101 KIAA0146 KIAA0415 KIAA0894 KIAA1659 KIAA1967 KIF16B KIF22 KIR2DL5A KLF13 KLHL1 KLHL22 KLHL24 KLK15 KLRC1///KLRC2 KLRC3 KLRD1 KLRG1 KRAS KRIT1 KRR1 KRT81 KYNU LATS1 LBX1 LCK LEF1 LEPROT LGMN LILRB5 LIMK1 LIN7C LITAF LMNB1 LMO3 LOC100132247///LOC348162///LOC613037///LOC728888///NPIPL3 LOC647070 LOC728855 LOC729991-MEF2B///MEF2B LPCAT1 LRRC37A3 LRRC40 LRRFIP1 LSM3 LTBP4 LTC4S LY6D LYN LYST LZTS1 MAF MAFF MANEA MAP2K7 MAP4K1 MARCKS MARK4 MBOAT7 MBP MERTK METTL7A MFI2 MICAL2 MIR21///TMEM49 MLL2 MLLT10 MLLT4 MMP12 MMP17 MPHOSPH9 MPZL1 MRAS MRM1 MRP63 MRPL17 MRPL22 MRPL44 MRPS12 MRPS7 MTCP1NB MTMR1 MYH11 MYH14 MYL6 MYL9 MYO19 MYO1C MZT2B NADK NBN NBPF10 NBPF10///NBPF11///NBPF12///NBPF15///NBPF16///NBPF24///NBPF8///NBPF9 NBPF10///NBPF12///NBPF15///NBPF16///NBPF8///NBPF9 NCBP1 NCOA3 NDUFS8 NEURL NEUROG2 NF1 NF2 NFAT5 NFE2L2 NFKB2 NFKBIA NFKBIB NID1 NIPSNAP3B NKTR NMBR NME1 NOL11 NOL3 NOP16 NOTCH2NL NPIPL3 NPLOC4 NPPC NR4A2 NT5C2 NUP155 NXF1 OBSCN OBSL1 ODF2 OMP ORM1///ORM2 OSGEP OTOF P2RY11///PPAN-P2RY11 P2RY14 PACSIN2 PAFAH1B3 PAGE4 PAPPA2 PAQR3 PAX8 PCBP2 PCDHB6 PCDHGA3 PCNXL2 PDAP1 PDE4C PDE4DIP PDGFA PDGFB PELI1 PEX16 PGF PHACTR1 PHF20L1 PI3 PINK1 PITPNM3 PKD1 PKNOX1 PKP3 PLCB1 PLEK PLK3 PLLP PMAIP1 PNLIPRP2 PNO1 PNRC1 POLR1B POLR2A POLR2F POP7 POSTN PPAP2A PPAP2B PPFIBP1 PPID PPIF PPP1R15A PPPDE1 PRB1 PRB4 PRDM12 PRDM2 PRDX2 PRF1 PRKAR1A PRKCI PROS1 PRR11 PRR4 PRRG4 PSMF1 PTAFR PTCD1 PTCH1 PTGER2 PTGS2 PTH1R PTMS PTP4A3 PTPN20A///PTPN20B PTPN22 PTPRA PTPRB PVR PYY RAB14 RAB40C RAB6A RAB6B RAD51L1 RAD51L3 RALGDS RASGRP1 RBM12 RBP3 RBP4 RCC1 RCN3 RECK REL RERGL RGS9 RIOK3 RNGTT RPAIN RPL10L RPL14 RPL23 RPL27A RPL30 RPL34 RPL35A RPL36A RPL39L RPLP2 RPS11 RPS6 RRAS RREB1 RRP1B RSL1D1 SAFB SAP30L SBF1 SCARB2 SCD5 SCLY SCML2 SCUBE3 SEC14L1 SECISBP2 SECISBP2L SELL SEMA3B SEPT6 SERINC3 SERP1 SERPINB1 SETDB1 SF1 SF3B1 SFTPA2 SFTPB SFTPC SFTPD SH2B2 SH2D1A SHANK1 SIGLEC6 SIGLEC8 SIX3 SLC16A3 SLC17A1 SLC19A1 SLC20A1 SLC22A3 SLC22A7 SLC24A6 SLC25A16 SLC25A36 SLC25A37 SLC26A2 SLC2A4RG SLC30A9 SLC35E1 SLC35F5 SLC39A4///SLC39A7 SLC39A9 SMA4 SMARCB1 SMG6 SMPD1 SNCA SNX24 SOCS1 SOD2 SORL1 SOX21 SPARC SPEN SPG21 SPN SPP1 SPRR1A SPTBN1 SPTLC2 SQSTM1 SRC SRR SRSF11 SRSF2IP SRSF5 SSH1 SSSCA1 SSX3 ST8SIA2 STAG2 STARD13 STATH STK17A STX16 SUPV3L1 TAF13 TAF7L TAF9B TAOK1 TAS2R16 TBRG4 TCF3 TCF7 TDRD3 TELO2 TFAM TFDP1 TFE3 THAP7 THBS1 THPO THRAP3 TIAL1 TIGD1L TLL1 TLX2 TLX3 TMEM187 TMEM63A TNF TNFAIP3 TNFAIP6 TNFRSF10C TNFRSF1A TNPO1 TOP3B TPO TPR TRA2A TRAC TRAC///TRAJ17///TRAV20 TRAF3IP3 TRBC1///TRBC2 TRDV3 TREH TRIB1 TROVE2 TSR1 TTC17 TTLL5 TUG1 TXN U2AF2 UBAP2 UBE4B UBIAD1 UBQLN4 UCHL5 UCN UGCG UGT2A3 UNC45A UQCRQ URB1 UROS USP10 USP34 UTF1 UTP14A VAMP1 VEGFA VGLL1 VIPR2 VNN2 WASL WDHD1 WDR18 WDR44 WDR6 WDR8 WIPI2 WNK1 WNT6 WSB1 WWP1 XCL1 XCL1///XCL2 XPNPEP3 XRCC2 ZBED4 ZBTB20 ZBTB43 ZC3H11A ZC3H12A ZC3H7B ZFP36 ZFP36L1 ZNF131 ZNF136 ZNF160 ZNF239 ZNF24 ZNF292 ZNF44 ZNF528 ZNF587 ZNF611 ZNF721 ZNF79 ZNF80 ZNF816 ZNF835 ZNF91 ZP2 | CurrentSmoker_54 Sample_43_never_smoker CurrentSmoker_39 NeverSmoker_52 Sample_44_never_smoker FormerSmoker_27 CurrentSmoker_37 Sample_40_former_smoker_without_cancer NeverSmoker_45 FormerSmoker_41 CurrentSmoker_10 Sample_48_never_smoker BAL_19_A0B0 CurrentSmoker_12 BAL_27b_A2B1 CurrentSmoker_36 CurrentSmoker_2 NeverSmoker_42 NeverSmoker_38 BAL_44a_A0B0 FormerSmoker_18 BAL_21_A0B0 NeverSmoker_3 FormerSmoker_28 FormerSmoker_34 CurrentSmoker_29 CurrentSmoker_1 Sample_47_never_smoker Sample_46_never_smoker BAL_44b_A0B0 BAL_46_A2B0 BAL_12_A1B0 BAL_25_A0B0 BAL_13_A0B0 BAL_39_A0B1 BAL_15b_A0B0 BAL_26_A2B0 BAL_37_A1B1 BAL_40_A0B0 BAL_31_A0B1 BAL_45a_A1B0 BAL_1_A0B0 FormerSmoker_76 BAL_33_A0B1 BAL_3_A1B0 BAL_45b_A2B0 BAL_17_A0B0 |
| **11** | ABCA6 ABCB1 ABCD2 ABHD6 ACADVL ACAP2 ACO2 ACSM1 ACTA2 ADAM21///ADAM21P1 ADAM28 ADAMDEC1 ADAMTSL2 ADCYAP1 ADORA3 ADRM1 AFF2 AHSA1 AKAP8L AKR1B10 AKR1C1 AKR1C2 AKR1C3 ALDH7A1 ALDOA ALK ANGPT1 ANK3 ANKMY2 ANKRD11 ANKRD12 ANKRD36B ANKRD6 ANXA1 ANXA10 ANXA11 ANXA2 ANXA2P2 ANXA7 AOX1 AP2A2 APBB2 APLP2 APOE APOLD1 AQR ARHGEF12 ARL6IP4 ART3 ASB8 ASL ASNA1 ATF3 ATP10B ATP12A ATP5C1 ATP5G2 ATP6V0E1 ATP6V1D ATP7B ATP8A1 ATPAF2 ATR ATRX BAIAP2L2 BANF1 BAT2L2 BAT3 BBX BCL2 BIRC3 BLOC1S1 BLVRB BMP15 BMP6 BSCL2 BTD BUB1 C10orf118 C11orf17///NUAK2 C15orf39 C15orf5 C16orf3 C19orf26 C1orf135 C20orf117 C9orf156 C9orf16 CACNA1A CACNB3 CADM1 CALM1 CALM3 CALY CASQ2 CBR3 CCDC28A CCL5 CCNA2 CCND1 CCND2 CCR7 CCR8 CCT2 CCT3 CCT8 CD177 CD27 CD3E CD59 CD7 CD84 CD97 CD99 CDC42BPA CDC6 CDH17 CDH6 CDK2 CDK7 CEACAM1 CEP68 CES1 CETN2 CFDP1 CHCHD2 CHCHD8 CHIT1 CHMP2A CIT CLC CLCA4 CLEC1A CLTA CNPY2 COL4A6 COMMD3 COMMD4 COPB2 COPE COPG COQ9 COX5B COX6A1 COX6B1 CR1 CRH CRIP1 CRP CTNNA1 CTSE CTSH CXCL13 CXCL9 CYLC1 CYLD CYP2A7 CYP2B6 CYP4F8 D4S234E DAD1 DCAF16 DCAF17 DDX1 DDX17 DDX41 DEFB126 DGCR11 DHPS DHX16 DIAPH2 DKFZP434C153 DMD DNAJC10 DNTTIP2 DOK5 DSCR6 DSTN DUSP6 DYNLRB1 EEA1 EFS EGFR EHF EIF2AK1 EIF3G EIF4E2 EIF5B ELAVL2 ELF5 ELK4 EMG1 ENSA EPAG EPAS1 ERAP1 ERP29 ETV1 FAM107A FAM46C FARP1 FBXL15 FCGBP FETUB FGF3 FGFR1OP FGFR2 FGL1 FKSG49 FLRT3 FMO1 FOLR1 FOS FOXN3 FTH1 FTL FUBP1 G6PD GABRA5///LOC100509612 GAD2 GALNT6 GAP43 GAS2L1 GATM GDF15 GGTLC2 GIMAP5 GMCL1 GML GMPR GNB2 GNGT1 GP1BB///SEPT5 GPATCH8 GPR64 GPRC5B GPX4 GRM1 GRM6 GRP GSTA1 GSTK1 GTDC1 HAB1 HAX1 HBA1///HBA2 HBB HCN2 HERC2P3 HGF HIST1H3E HIST1H3H HIVEP2 HLA-DOB HLA-DQA1 HLF HMGN5 HOPX HOXC6 HPCAL4 HSBP1 HSD3B1 HSP90AB1 HSP90B1 HSPA1A///HSPA1B HSPA4 HSPA8 HSPD1 HYMAI IAPP IDH2 IDO1 IDS IFITM1 IFNA10 IFNA14 IFNA16 IFNA4 IFT20 IGHA1///IGHA2///LOC100126583 IGHG1///IGHG2///IGHM///IGHV4-31 IGK@///IGKC IGKC IGL@ IGLC7///IGLV1-44///LOC100290481 IGLV1-44///LOC100290481 IKBKB IKZF2 IL1RL1 IL2RB IL32 IL33 IL6ST IMPA2 ING2 INHBC IPCEF1 IPW IRF1 ISL1 ISLR ITGB1BP1 ITM2A JTB JUND KALRN KAT5 KATNB1 KCNA5 KCNJ15 KCNJ2 KCNMB3 KDM5A KIAA0776 KIF5A KIFAP3 KIR2DS2 KLF4 KLRC1///KLRC2 KLRD1 KRT13 KRT2 KRT6A KRTAP4-7 KTN1 LAMC1 LARP4B LATS1 LEPR LHFP LMAN2 LMNA LMNB1 LMO4 LOC100170939 LOC100507619 LOC100507630 LOC100507804///TPSAB1 LOC100507851///PRAMEF1///PRAMEF2 LOH3CR2A LPAR6 LPIN1 LRCH1 LRRN3 LSM4 LSM5 LSM7 LTF LUC7L3 MAGEA12 MAGEA3 MAGEA9///MAGEA9B MAGEC2 MAN1C1 MAPT MARCKS MBD5 MBNL2 MCOLN3 MDM4 ME1 MED13L MED7 MEX3D MGC4859 MIER2 MIF MLL MLLT11 MMP10 MORC1 MPRIP MRPL18 MRPS17///ZNF713 MRPS7 MS4A1 MST1///MST1P2///MST1P9 MTF2 MTMR7 MUC5AC MUC7 MYCBP MYH11 MYH3 MYNN MYO1B MYO6 MYST2 MYST3 MYST4 N4BP3 NADSYN1 NCR3 NDUFAB1 NDUFB2 NEDD8 NFAT5 NFKB2 NHLH2 NIPSNAP3B NIT1 NIT2 NKTR NKX3-1 NLGN4Y NOMO1///NOMO2///NOMO3 NOS2 NPAT NQO2 NR0B1 NR2F1 NR2F6 NRG1 NSDHL NT5E NTNG1 NTRK3 NTS NUBPL NUCKS1 NUFIP1 NUP188 NUPR1 OGDHL OMG OPA1 OR2B2 OR6A2 OTUB1 P4HA2 PADI2 PAK3 PAM PCSK6 PDE1B PDE8B PDIA4 PDP1 PDXK PELO PEX11A PFDN5 PFKL PHF20L1 PHOX2B PKP4 PLA2G16 PLCB1 PLEKHA1 PLUNC PLXDC1 PNKP PNN POLD3 POLRMT POSTN POU4F2 PPAP2B PPBP PPIB PPID PPIG PPP1R15A PPP1R3A PPP1R7 PPP2R1B PPP3R1 PRB1 PRB3 PRDX1 PRDX4 PREX2 PRKAA2 PRKCA PRKCI PRO2012 PRPF8 PRR14 PRR4 PRUNE2 PRY PSMB3 PSMB5 PSMB6 PSMC4 PSMD1 PSMD4 PSMD6 PSMD8 PTGFR PTH2R PTPRZ1 PUS7 RAB35 RABAC1 RAD51L3 RANBP1 RARRES1 RBM25 RBM4 RBM5 RCHY1 RFTN1 RFX1 RGS4 RHOBTB3 RIMBP2 RNASEH2B RNF8 RNPEP RPL27A RPL38 RPL39 RPLP2 RPN1 RPN2 RPS11 RPS6KA6 RPS8 RPS8///RPS8P8 RRAS2 RTN3 RUFY1 RUNX1T1 S100A10 S100A11 S100A8 SAE1 SAMM50 SAP18 SC5DL SCCPDH SCD5 SCN3A SENP6 SEPX1 SERF2 SERPINB6 SERPINB9 SF1 SF3B2 SF3B5 SFRP1 SFRS18 SFTPB SH2D2A SH3GL3 SIKE1 SKP1 SLC16A7 SLC25A36 SLC25A37 SLC26A2 SLC30A10 SLC30A3 SLC30A5 SLC39A8 SLC4A4 SLC4A7 SLCO1A2 SLCO1C1 SMARCA1 SMARCA4 SMC3 SMC5 SMYD2 SNX1 SNX15 SORBS2 SOX2 SPEN SPRR1B SSSCA1 SSX2IP ST18 STATH STC1 STIP1 STK17B STMN4 STOML2 STX10 STX18 SULT1A1 SUPT7L SVEP1 SYT1 TAAR2 TAAR3 TAF4B TAF9B TAGLN2 TALDO1 TAPBP TAZ TBC1D8B TBCB TBCD TCEB2 TFCP2L1 TFPT TGFBR2 TIAM2 TIPARP TLR5 TMED10 TMEM111 TMEM147 TMEM5 TNC TNFAIP6 TNFSF10 TNNI3 TOP1 TOR3A TOX TP63 TPR TPSAB1 TPSAB1///TPSB2 TRAC TRIM23 TRIM48 TRIM49///TRIM49L2 TRMT112 TSFM TSPAN3 TTC3 TTLL4 TTTY15 TTTY2 TUBA1B TUBA1C TUBB TXK TXN TXNDC9 TXNL4A TXNRD1 UBXN4 UCHL1 UCHL3 UCP2 UGT1A10///UGT1A7///UGT1A8 UGT2A1///UGT2A2 ULBP1 USF2 UTP18 VAMP8 VAPA VAV3 VCP VDAC3 VEGFC VOPP1 VPS13A VPS37C VPS52 WASL WBP4 WHSC1L1 WHSC2 WNK1 WNT2 WSB1 XYLT1 YIF1A YIPF3 YY1 ZAK ZBTB16 ZBTB43 ZBTB44 ZBTB7A ZCCHC4 ZFPM2 ZNF131 ZNF174 ZNF192 ZNF22 ZNF236 ZNF264 ZNF286A ZNF287 ZNF423 ZNF507 ZNF654 | BAL_17_A0B0 BAL_1_A0B0 BAL_44b_A0B0 BAL_15a_A0B1 BAL_3_A1B0 BAL_33_A0B1 BAL_26_A2B0 BAL_45a_A1B0 BAL_43_A0B0 BAL_27a_A0B0 BAL_25_A0B0 BAL_39_A0B1 BAL_4_A0B0 BAL_40_A0B0 BAL_13_A0B0 BAL_31_A0B1 BAL_12_A1B0 BAL_37_A1B1 BAL_15b_A0B0 BAL_47a_A0B0 BAL_18_A1B0 BAL_45b_A2B0 BAL_41_A1B0 BAL_44a_A0B0 CurrentSmoker_1 BAL_47c_A0B1 BAL_47b_A0B1 BAL_42_A0B0 FormerSmoker_34 CurrentSmoker_10 BAL_29_A1B0 BAL_20b_A1B1 |
| **12** | ABCC9 ACP1 ACP6 ACSL3 ACSM3 ACTR5 ADA ADAM23 ADAM8 ADAMTS2 ADAMTS6 ADRA1B AGFG2 AGT AHCYL1 AK1 AKR1B10 AKR1C2 AKR1C3 ALDH6A1 ALG13 AMACR ANP32A///ANP32D ANXA2 AP3D1 APBB2 APOC3 APOL2 APP AQP1 AQP4 ARF4 ARHGAP19 ARHGDIG ARSA ATF1 ATF4 ATP1B1 ATP6V0E2 AZGP1 B2M B3GALT2 B4GALT1 B4GALT7 BAMBI BAT2L2 BBOX1 BCAT1 BCAT2 BCL2L1 BHLHB9 BIRC3 BMP5 BMP6 BNIP1 BRD4 BST2 BTF3 BTNL2 BUB1 C13orf1 C14orf139 C16orf42 C16orf59 C16orf88 C19orf10 C19orf2 C19orf56 C20orf117 C21orf91 C2orf47 C9orf167 CADM4 CALM2 CAPN10 CARD14 CCDC41 CCDC85B CD226 CD27 CD59 CD6 CD63 CD7 CDC5L CDH13 CDK5RAP1 CDKN3 CEACAM3 CEACAM5 CENPC1 CEP70 CFB CFLAR CHST3 CHST8 CKB CLDN10 CLDN17 CLDN18 CLSPN COBLL1 COL6A1 COL9A2 COPB1 COPB2 COPG COQ6 COX5B COX6A1 COX6C COX7C CRELD2 CRYAA CSH2 CSNK1A1 CSTA CSTF2T CTNNA1 CTNND1 CTSL2 CXCL13 CXCR6 CYB561D2 CYP1A1 CYP1B1 CYP3A5 DAD1 DAZAP2 DCAF13 DCN DCTN2 DDX6 DKK3 DLG4 DNAJC10 DNAJC12 DPP4 DTNA DUOX2 ECHDC3 EEA1 EEF1A1 EEF2 EFR3B EGR3 EIF3M EIF4A2 EMX2 EPHA3 ERLIN1 EZR FAM117A FAM36A///HOXA7 FAM70A FASTKD5 FBXL18 FBXO17///SARS2 FBXO22 FBXO46 FCGBP FDFT1 FECH FKSG2 FLRT3 FOLH1 FOLH1B FOLR3 FTSJ2 FUBP1 FYN GABBR2 GAGE1 GAL3ST4 GALNT12 GALNT3 GALNT7 GALR2 GAPDH GDF2 GLUL GM2A GMDS GNA15 GNE GNL3L GPR153 GRK5 GSPT2 GSTM3 GTF2A1L///STON1-GTF2A1L GTF2F2 GTPBP4 GTSE1 GYPB H1FX H3F3A///LOC440926 H3F3B HAB1 HAO2 HAX1 HDGFRP3 HEXIM1 HIST1H2BI HLA-A HLA-DQA1 HLA-G HMGN1 HMP19 HNRNPA2B1 HNRNPH1 HOMER3 HOPX HS1BP3 HSD11B1 HSD17B14 HSP90AA1 HSPA8 HTR2A HUWE1 ICAM3 ICOS ID2///ID2B IDH1 IDO1 IFI6 IFIH1 IFITM3 IFNA14 IL1RAPL1 IL2RA JAK3 JTB KDELR2 KDM1A KDM4B KIAA1109 KIR2DL1 KIR3DX1 KLHL11 KRT13 KRT6A KRT6A///KRT6B///KRT6C LAG3 LAIR2 LAMB1 LAMP1 LEF1 LGALS3BP LMBR1L LOC100290070 LOC100505523 LOC100507328 LOC100507328///LOC100508591 LOC150776///SMPD4 LOC220077 LPCAT3 LPP LPPR3 LSS LTB4R LTBP4 LY6D LYZ MAGEA4 MAGED4///MAGED4B MANF MAPKAPK2 MC4R MCM4 MCM5 MGAT2 MGC4859 MGRN1 MMADHC MMP12 MPHOSPH9 MPI MPZL1 MS4A1 MTAP MTMR3 MUC5AC MUT MYH11 MYL12A///MYL12B MYNN MYT1L NDRG2 NDUFV2 NF1 NGDN NIT1 NOLC1 NPIPL3 NPM1 NRG2 NRL NRN1 NRXN2 NSUN5 NTRK2 NTRK3 NUP50 OAS1 OAT OBSL1 OR7A17 ORAI2 ORM1///ORM2 OS9 OSBPL3 P4HB PADI2 PAM PARG PAX3 PCDHGC3 PDE4DIP PDE7B PDIA3 PDPK1 PDXDC1 PER1 PEX7 PHB PHC3 PHF15 PHF21A PHLPP2 PIP4K2A PIP5K1A PKLR PLCG1 PLCXD1 PLXDC1 PLXNC1 PNKP PNN POLA1 POLR3C PORCN POU2F1 PPFIBP1 PPIA PPIB PPIC PPIH PPP1R15A PPP1R3A PPP2R1B PPP2R2B PPP2R5A PPP3CC PRB1 PRKCB PRPS2 PRSS1 PSG1 PTMA PYCRL RAB11A RAB3GAP2 RABEPK RALGDS RARA RASAL1 RASGRP1 RDH8 RETSAT RFC5 RGS3 RHD RHOBTB3 RNF144A RNF40 RNF6 RNPEP///TMEM189///TMEM189-UBE2V1///UBE2V1 RPL23AP32 RPL27 RPL27A RPL3 RPLP0 RPN1 RPN2 RPS11 RPS24 RPS3 RPS3A RPS6 RTEL1 SCAMP4 SCAND2 SCGB1D1 SEC22B SERF2 SETD2 SFTPB SFTPC SH3BP4 SIPA1 SKP1 SLC12A5 SLC15A1 SLC25A12 SLC27A6 SLC2A1 SLC30A3 SLC30A5 SLC35B1 SLC39A2 SLC7A11 SLC7A6 SLIT3 SMA4///SMA5 SMARCD1 SNAPC1 SNRPD2 SNRPF SNX15 SORL1 SOX4 SPATA2 SPATA2L SPIN2A///SPIN2B SPRR1A SPRR1B SPRR3 SQSTM1 SREK1IP1 SRP14 SRPRB SRSF4 SSPN ST3GAL2 STAT1 STAT2 STATH STAU1 STRN4 TACR2 TCF12 TCF7L2 TCN1 TDP1 TEX13A TFPI TGM2 TGM4 TICAM1 TIMP1 TLE1 TMBIM6 TMED10 TMED9 TMEFF1 TMEM80 TMSB4X///TMSL3 TMSB4Y TNFRSF9 TNFSF10 TPD52L1 TPMT TPR TPT1 TRAFD1 TRANK1 TRAPPC2L TRIM14 TROAP TRPA1 TRPM3 TSC22D2 TSFM TSPAN3 TTC23 TTC28 TTC3 TXN UBA52 UCKL1 UGGT2 UMOD UMPS UPK1B UQCC USP20 USP34 UTP18 VAMP1 VAPB VGLL4 WARS WDR70 WHSC1 WSCD2 XBP1 XYLB YWHAE ZBTB48 ZNF141 ZNF16 ZNF197 ZNF211 ZNF224 ZNF282 ZNF652 ZNF771 ZSCAN18 ZXDC | BAL_39_A0B1 BAL_1_A0B0 BAL_3_A1B0 BAL_47b_A0B1 BAL_47c_A0B1 BAL_27a_A0B0 BAL_45a_A1B0 BAL_17_A0B0 BAL_15a_A0B1 BAL_25_A0B0 BAL_18_A1B0 BAL_43_A0B0 BAL_31_A0B1 BAL_40_A0B0 BAL_37_A1B1 BAL_33_A0B1 BAL_47a_A0B0 BAL_4_A0B0 BAL_41_A1B0 BAL_13_A0B0 BAL_29_A1B0 BAL_26_A2B0 BAL_12_A1B0 BAL_20b_A1B1 BAL_44b_A0B0 BAL_42_A0B0 CurrentSmoker_12 CurrentSmoker_111 FormerSmoker_114 CurrentSmoker_83 BAL_15b_A0B0 BAL_45b_A2B0 BAL_44a_A0B0 CurrentSmoker_113 |
| **13** | ABCB6 ABHD2 ABLIM1 ACSL3 ADAM28 ADD3 AGAP1 AGRN AKAP1 AKAP2///PALM2-AKAP2 AKAP8L ALCAM ALDH5A1 ALOX15B ANGPT2 ANKRD11 ANKRD12 ANKRD36 ANKRD36B ASCC1 ATF3 ATF5 ATN1 ATP2B1 ATP6V1D ATP6V1G1 ATPAF2 ATRX ATXN2L ATXN7L3B B4GALT5 BAG3 BAI3 BAT2L2 BAZ1A BAZ1B BBS4 BBX BCL6 BCLAF1 BDH2 BMP6 BPTF BRD2 BRD4 BTBD3 C11orf24 C11orf9 C1orf77 C2orf43 C3 C7orf44 CACNA1B CAND1 CASP9 CAST CCNA1 CCND1 CCNF CCNI CCT6A CD2 CD6 CDC20 CDC25A CDC25C CDC42 CDC42BPA CDK11A///CDK11B CENPT CEP290 CEP350 CFLAR CHD4 CHD9 CIR1 CKAP4 CLCN3 CLUAP1 CNNM3 CNOT3 COIL COL4A3BP COL6A1 COQ7 CORT CROCCP3 CSDA CSNK1A1 CSPP1 CSTF2T CTBP2 CUEDC1 CUX1 CXCL13 CXCL5 CXorf56 CYC1 CYLD CYP2B6 DAXX DBT DCTN2 DDOST DDX17 DDX24 DDX27 DDX3Y DGKQ DHRS2 DHRS7B DHX38 DICER1 DLAT DNAJC16 DNAJC17 DPY19L1 DUOX2 DUSP5 DYNC1LI2 DYRK2 EBNA1BP2 ECSIT EFNB2 EID1 EIF3A EIF4A1 EIF4G1 ELF1 ENC1 EPRS ESF1 EZR F11R FAM120A FAM129A FAM13A FBXO42 FBXW12 FDPS FGFR3 FKBP1B///MFSD2B FLJ13197 FLRT3 FNBP1 FOXO3///FOXO3B FTH1 FUT7 FXC1 G0S2 G3BP2 GABBR1///UBD GABRA4 GALK2 GAP43 GATAD1 GBP1 GCLM GDF1///LASS1 GLG1 GNAL GNL3 GOLGA2 GOLGA4 GPR44 GUSBP3 H3F3B HAB1 HBA1///HBA2 HBB HBE1 HBG1///HBG2 HIPK1 HIST1H2BE HMGB1 HMGXB4 HNF4A HNRNPA3 HNRNPA3///HNRNPA3P1 HNRNPH3 HNRNPM HNRNPR HOPX HSD17B7 HSP90AB1 HSPA1A///HSPA1B HSPH1 IDI1 IDO1 IDS IFI16 IGFBP3 IGHA1///IGHA2///IGHD///IGHG1///IGHG3///IGHG4///IGHM///IGHV4-31///LOC100133862 IGHA1///IGHA2///LOC100126583 IKBKAP IL27RA IL6ST IL7 IL7R IL8 ILF3 IMPDH1 IQCK IQGAP1 JUN KATNB1 KCNJ5 KDM2A KDM4B KDM6B KIAA0020 KIAA0562 KIAA1033 KIAA1109 KIAA1659 KIF3A KLF6 KLHDC10 KLHL1 KPNA3 KPNB1 KRAS KRT33A KRT86///LOC100509764 KYNU LIMS1 LOC100506076///LOC100506123 LOC100507804///TPSAB1 LOC100509749 LOC100510525///SUZ12///SUZ12P LOC150759 LOC441259///PMS2L2///PMS2P1///PMS2P6 LONP2 LRRC14 LSS LTBP4 LTF LUC7L3 MAB21L1 MAGI2 MAP2K5 MAP9 MARCKS MBD4 MBP MED13L MID1 MLEC MLL MLL2 MMP7 MPDZ MPHOSPH10 MPPE1 MRPS31 MSH3 MTF1 MTUS1 MTUS2 MYH10 MYH14 MYL10 MYO1A MYO6 MYOF N4BP2L2 NAA15 NAA40 NAB1 NBPF10 NCOA3 NCOA6 NEK1 NF1 NF2 NFAT5 NGLY1 NKTR NOL12///TRIOBP NOS2 NR2C1 NR4A2 NUP160 OAS2 OAZ2 OSBPL1A OTUD4 PA2G4 PAFAH1B3 PALLD PARG PCBP2 PCDH7 PCGF1 PCM1 PDAP1 PDE4D PDP1 PEX11A PFDN2 PGLYRP1 PIK3CD PIK3R1 PIP5K1A PITPNM3 PLCB4 PLEKHB2 PLUNC PMAIP1 PMS2L2 PMS2P1 PMS2P3 POU4F2 PPAP2B PPBP PPIG PPP1R10 PPP1R12A PPP1R7 PPP4R2 PRICKLE4///TOMM6 PRKCI PRPSAP2 PRR11 PRR4 PRRC1 PRSS3 PSMD7 PTAFR PTMS PTP4A2 PTPN11 PTPRC PWP1 RAB27A RAD23A RALBP1 RALGDS RANBP2 RARRES1 RBL2 RBM25 RBMX2 RGP1 RHEB RHOBTB3 RHOF RNF115 RNF19A RNF40 RORA RTEL1///TNFRSF6B RUFY3 RYBP SAA1///SAA2 SART3 SEC14L1 SEC14L3 SEC22B SEC23B SEC23IP SECISBP2L SEL1L SENP3 SERBP1 SERINC3 SERPINB4 SF1 SF3B1 SFRS18 SFTPB SH3BP1 SH3GLB2 SIDT2 SIKE1 SIVA1 SLC22A7 SLC23A2 SLC25A36 SLC25A37 SLC26A2 SLC26A4 SLC7A2 SLC9A3R2 SMARCA2 SMARCA4 SMARCC1 SMC3 SMG1 SMOX SNAPC1 SNCA SOD2 SON SORD SPEN SPG21 SPPL2B SRPK2 SRRM2 SRSF2IP SSB SSH1 SSRP1 STAG2 STC1 STRN4 STX2 SUCLG2 SUV420H1 SYNCRIP SYNJ2BP SYNPO2L TAOK1 TAX1BP3 TBL1X TBX2 TCF25 TCF3 TCF4 TES TF TFAM TFDP1 TFIP11 TGFA THAP4 THOC2 THRAP3 TLK2 TM2D1 TM4SF1 TM9SF3 TMBIM4 TMEM45A TMEM5 TMEM57 TMEM66 TMEM97 TNFAIP3 TNFSF10 TNPO1 TOP1 TOP3B TOX4 TPM2 TPR TRA2A TRA2B TRAF3IP1 TRAK1 TREML2 TRIM13 TTC3 U2AF1 U2AF2 UBE2H UBN1 UBXN4 UGCG USF2 USP1 USP13 USP2 USP27X VRK3 WASL WDR77 WIPI2 WNK1 WSB1 YTHDC1 YTHDF3 YWHAE YY1 ZBTB43 ZC3H11A ZDHHC11 ZKSCAN5 ZMYND8 ZNF136 ZNF160 ZNF222 ZNF292 ZNF44 ZNF576 ZSCAN18 | Sample_47_never_smoker CurrentSmoker_29 FormerSmoker_28 BAL_17_A0B0 NeverSmoker_45 FormerSmoker_27 Sample_46_never_smoker Sample_40_former_smoker_without_cancer NeverSmoker_42 CurrentSmoker_36 FormerSmoker_18 BAL_18_A1B0 Sample_48_never_smoker BAL_47b_A0B1 BAL_25_A0B0 BAL_4_A0B0 BAL_43_A0B0 BAL_3_A1B0 NeverSmoker_52 BAL_42_A0B0 BAL_1_A0B0 BAL_33_A0B1 NeverSmoker_38 BAL_27a_A0B0 Sample_44_never_smoker BAL_37_A1B1 BAL_12_A1B0 FormerSmoker_34 BAL_26_A2B0 BAL_13_A0B0 CurrentSmoker_10 BAL_44b_A0B0 BAL_15a_A0B1 FormerSmoker_41 BAL_20b_A1B1 BAL_45b_A2B0 BAL_40_A0B0 NeverSmoker_3 BAL_29_A1B0 BAL_47a_A0B0 BAL_45a_A1B0 CurrentSmoker_39 BAL_31_A0B1 BAL_44a_A0B0 Sample_43_never_smoker BAL_41_A1B0 BAL_15b_A0B0 BAL_47c_A0B1 BAL_39_A0B1 CurrentSmoker_68 CurrentSmoker_1 CurrentSmoker_2 BAL_27b_A2B1 NeverSmoker_70 FormerSmoker_72 CurrentSmoker_67 |
| **14** | ABCA1 ABCA7 ABHD14A ABHD2 ABO ACAP1 ACTN1 ACTR6 ADD1 ADORA1 AGPAT1 AGPAT2 AGR2 AKAP12 AKAP8L AKR1B10 ALDH1A2 ALPK3 ANKLE2 ANKRD36 ANP32E ANXA3 AP1G2 AP3S2 ARHGDIA ARHGEF3 ARSD ASAH1 ASTN2 ATF3 ATN1 ATP5E ATP5O ATP6V0E2 ATP6V1G2///BAT1 AXL B4GALT7 BAT3 BCL11B BTBD2 BTG3 C12orf35 C19orf26 C19orf66 C2 C6orf26///MSH5 CALCOCO1 CALM1 CALR CAPN3 CASP3 CBLB CBX7 CC2D1A CCDC144A CCDC22 CCDC99 CCL13 CCL2 CCL5 CCL8 CCR2 CCR5 CCS CD2 CD3D CD7 CD81 CD8A CDC20 CDC42BPB CDK1 CDK10 CDK2AP1 CDRT1 CDYL CEACAM5 CEACAM6 CECR1 CENPT CHKB-CPT1B///CPT1B CKS2 CLDN18 CLPTM1 CLSTN2 CNOT3 COL6A1 COL9A2 COMMD3 COPB1 COPB2 CORO1A CORO2A COX5B COX7B CPSF1 CR1 CST3 CST7 CSTA CTBP1 CTSD CTSH CXCL11 CXCL13 CXCL9 CXCR6 CXXC1 CYP1A1 CYP1B1 CYR61 DCLRE1A DHRS12 DHX30 DIAPH3 DNAJC2 DUSP5 DUSP6 EEF1D EHBP1L1 EIF3M EIF4B EIF4G3 EIF5B EML3 EMP1 ENPP2 ENTPD6 EPB41L1 ERCC2 F13A1 FAF2 FAHD2A FAIM3 FAM129A FAM65B FCGR2B FCN1 FGFR1OP FGL2 FHOD3 FKBP3 FLOT2 FOLR1 FOXK2 FRG1 FYN G0S2 GALNT7 GCHFR GCLM GGA1 GINS2 GJA1 GLT25D1 GNB2 GNL3 GNLY GPD1 GPN1 GPR171 GPR183 GPX3 GPX4 GRINA GTF2H2///GTF2H2B///GTF2H2C///GTF2H2D GTSE1 GZMA GZMB GZMH GZMK HBA1///HBA2 HBB HBG1///HBG2 HGS HIPK2 HIST1H2AC HIST1H2BK HLA-DQB2 HMGCS1 HNRNPA3///HNRNPA3P1 HOPX HP///HPR HPSE HSD17B14 HSP90B1 HSPA1A///HSPA1B HSPA4 IDE IFITM1 IL1R2 IL32 IL6ST ILVBL INTS8 ITGA6 ITIH4 ITIH4///MUSTN1 ITK KAZ KCNAB1 KIAA0467 KLRB1 KLRC1///KLRC2 KLRG1 KRAS KRT13 KRT6A LAIR2 LAMB1 LAMP3 LARP1 LARP4B LCK LEF1 LGALS3BP LGMN LIMK2 LMO2 LOC100507328 LOC100507630 LOC150759 LOC150776///SMPD4 LRP1 LTB4R LY6E MAD1L1 MAF MAN2C1 MAPK6 MAST3 MCL1 ME1 MECP2 MELK MERTK METTL9 MFSD10 MGRN1 MIIP MINK1 MLF1IP MLL MLL4 MOSPD3 MPHOSPH10 MRPL9 MSMB MSR1 MST4 MUC5AC MUTED///TXNDC5 MYCBP MYCBP2 MYO15B N4BP1 NAPA NCOA3 NCOR2 NDUFB3 NDUFS8 NEDD9 NKG7 NPEPPS NPM1 NPRL3 NR1H2 NUCB1 NUP62 NUP98 NXF1 OAT ORM1 PACS2 PCDH9 PDE4A PDIA6 PEBP1 PEX14 PHKA2 PHLDA2 PI4KB PIGO PKN1 PLA2G16 PLCB2 PLD3 PLEC PLUNC PLXNA3 PLXNC1 PMAIP1 PNPLA2 PNPLA6 POMZP3 POR PPP1R15A PPP5C PRF1 PRKCD PROS1 PRPF8 PRSS21 PSMC1 PTK2B PTMS PTPN4 PTTG1 QSOX1 RAB11A RAB27A RARA RASGRP1 RASSF2 RBM17 RBM42 RELB REXO2 RHOBTB3 RNF41 RORA RPL14 RPL27A RPL38 RPS11 RRBP1 RYR1 S100A8 S100P SAR1B SCAP SCEL SDAD1 SEC14L3 SENP3 SEPT4 SERPINB9 SFTPA2 SFTPC SH2B2 SH2D1A SH3TC1 SIN3B SLC19A2 SLC22A13 SLC25A1 SLC25A11 SLC29A1 SLC35A5 SLC36A1 SLC4A7 SLC6A1 SLC7A11 SLC7A8 SLCO3A1 SLIT1 SLIT2 SMAD5 SMG7 SMPDL3A SNCA SNRPE SNRPG SORBS3 SOX13 SPOCK2 SPRR1B SREBF2 SRPX2 SRSF1 ST8SIA4 STAP1 STAT1 STAT4 STAT5A STEAP3 STK17A TACSTD2 TAF10 TAGLN TAP1 TBC1D10B TBC1D17 TCN1 TGFB1 TM4SF1 TMEM45A TNFSF10 TNFSF12-TNFSF13///TNFSF13 TNNI2 TPP1 TPR TRAC TRAC///TRAJ17///TRAV20 TRBC1 TRBC1///TRBC2 TRD@ TRIM28 TSC2 TSPAN13 TWF1 TXN TXNDC9 TXNRD2 UBE2G2 UBE2L3 UCHL1 USP11 VAMP2 VCAN VDR VEGFB WBP2 WSB2 WWP1 XPNPEP1 YARS2 YWHAE ZBTB16 ZBTB38 ZCCHC24 ZFHX3 | BAL_31_A0B1 BAL_41_A1B0 BAL_44b_A0B0 BAL_1_A0B0 BAL_44a_A0B0 BAL_47c_A0B1 BAL_12_A1B0 FormerSmoker_34 BAL_17_A0B0 BAL_15b_A0B0 BAL_15a_A0B1 BAL_27a_A0B0 Sample_43_never_smoker BAL_13_A0B0 BAL_45a_A1B0 BAL_25_A0B0 BAL_29_A1B0 BAL_26_A2B0 BAL_47a_A0B0 Sample_44_never_smoker BAL_3_A1B0 BAL_21_A0B0 BAL_40_A0B0 Sample_40_former_smoker_without_cancer NeverSmoker_45 BAL_19_A0B0 NeverSmoker_52 BAL_27b_A2B1 BAL_18_A1B0 NeverSmoker_42 BAL_33_A0B1 BAL_20a_A1B1 NeverSmoker_38 BAL_42_A0B0 Sample_47_never_smoker FormerSmoker_18 BAL_4_A0B0 BAL_37_A1B1 NeverSmoker_3 CurrentSmoker_39 CurrentSmoker_1 CurrentSmoker_54 BAL_43_A0B0 BAL_47b_A0B1 Sample_48_never_smoker BAL_46_A2B0 NeverSmoker_103 FormerSmoker_28 FormerSmoker_41 BAL_20b_A1B1 |
| **15** | ABCA5 ABCD3 ABHD2 ACAD8 ACSL3 ACTR6 ADAM28 ADAMTSL4 ADD3 ADH7 ADM AGFG1 AGGF1 AGL AHCYL2 AKAP10 AKAP13 AKR1B10 AKR1C1 ALDH18A1 ALOX15 ALPL AMFR AMMECR1 ANKRD11 ANKRD12 ANP32B ANXA3 AP1G2 AP3D1 APOL1 APOO APPL2 ARG2 ARGLU1 ARHGAP32 ARHGAP5 ARHGEF12 ARID5B ASAP1-IT ASPH ATF3 ATP12A ATP13A3 ATP1B1 ATP2A2 ATP2B4 ATP6V0A4 ATP7B ATR ATRX ATXN10 AZGP1 AZIN1 B4GALT4 B4GALT5 BAALC BAT2L2 BATF BBS9 BBX BCL3 BCL6 BIRC3 BMPR1A BPTF BRCC3 BTBD3 C10orf57 C14orf1 C14orf104 C16orf80 C18orf10 C19orf22 C3 C6 C6orf97 C8orf4 CA12 CABYR CALM1 CASP10 CCL5 CD2AP CD59 CDC14B CDC42BPA CEACAM1 CEACAM5 CEBPD CEP350 CEP57 CEP68 CERK CES1 CFB CFH///CFHR1 CFI CHL1 CHST15 CLCA2 CLCA4 CLCN3 CLDN10 CLIC5 CLINT1 CLMN CLN8 CMAH CNN3 COIL COL21A1 COL4A3BP COL4A5 CP CP110 CPA3 CPD CREB1 CRELD2 CRIP1 CROCCP2 CSNK1A1 CSNK2A1 CSPP1 CTGF CTNND1 CXCL1 CXCL10 CXCL6 CXCL9 CXCR6 CYB5A CYLD CYP1A1 CYP1B1 CYP4F3 CYR61 DAAM1 DAP DCAF7 DDX17 DDX24 DEFB4A DHRS7B DHX35 DHX40 DHX9 DLG1 DMD DNAJB4 DNAJC10 DNAJC12 DNAJC16 DNAJC3 DPY19L4 DPYSL3 DSC2 DSG2 DSP DST DUOX2 DUSP5 DYNLL1 DYRK2 DZIP3 EGR1 EHBP1 EI24 EID1 EIF2AK3 EIF2C3 EIF3A EIF4B EIF4G1 ELF1 ELK3 EPRS EPS8 ERBB3 ERCC3 ESF1 ETNK1 F2RL1 F3 FADS1 FADS3 FAM120A FAM129A FAM13A FAM172A FAM179B FAM18B1 FAM36A///HOXA7 FAM3C FAM60A FAM65B FAU FBLN5 FBXO3 FKBP5 FMO3 FMO5 FNBP1 FNBP1L FOSB FUT3 FUT6 FXR1 FZD6 GABBR1///UBD GAD1 GAGE1///GAGE12F///GAGE12G///GAGE12I///GAGE12J///GAGE2A///GAGE2B///GAGE2C///GAGE2D///GAGE2E///GAGE3///GAGE4///GAGE5///GAGE6///GAGE7///GAGE8 GALNT1 GALNT3 GALNT7 GAS7 GBP1 GCH1 GCLC GCLM GCNT3 GDF15 GFPT1 GIPC2 GLB1L GLG1 GLT8D1 GMCL1 GMDS GMEB1 GNAL GNE GOLGA6L5///GOLGA6L9 GOLGA8A GOLIM4 GPX2 GRAMD3 GSK3B GULP1 GZMA GZMB H2BFS HCFC1R1 HCP5 HEBP2 HERC6 HIPK1 HIST1H2AC HIST1H2BD HIST1H2BE HIST1H2BK HIST1H4C HIST2H2BE HLA-DQA1 HMGA1 HNRNPD HNRNPR HS3ST1 HSP90B1 HSPA1A///HSPA1B ID1 IDH2 IDO1 IER2 IER3 IFI16 IFITM1 IFITM2 IFITM3 IGFBP3 IGFBP7 IGHG1///IGHG2///IGHM///IGHV4-31 IGK@///IGKC IL1A IL32 IL33 IL6ST IL7R ILF3 INPP1 INPP4B IPO8 IQCB1 IQGAP1 IRF1 IRF9 ITGA6 ITM2A JAG1 KATNB1 KBTBD4///PTPMT1 KCNG2 KCNJ15 KCTD12 KDM2A KDM5A KDM5B KIAA0562 KIAA0776 KIAA0907 KIAA1033 KIAA1659 KIFAP3 KIT KLF5 KLF9 KLHDC10 KLHL29 KPNA3 KPNA6 KRAS KRT10 KRT13 KRT23 KRT6A L1TD1 LAMC1 LAMP3 LAPTM4B LARP1 LBH LDLR LEPROT LIMS1 LMNA LOC100288142///NBPF1///NBPF10 LOC100506076///LOC100506123 LOC100506168 LOC100507328 LOC100507804///TPSAB1 LOC150759 LOX LRBA LRRC16A LRRFIP1 LUC7L3 LXN LY6D LYST MACF1 MAPRE3 MARCKS MARCKSL1 MBD4 MBOAT7 MBP MCM9 MDM1 MED1 MED24 MICAL2 MLEC MLLT10 MLXIP MMP10 MORF4L2 MPZL1 MPZL2 MRPS31 MSH3 MST4 MTA1 MTUS1 MUC1 MUC13 MUC2 MUC4 MUC5AC MUC5B MXI1 MYCBP MYO1B MYO1D MYO6 MYST4 NAA15 NAT1 NBEA NBPF10 NCOA2 NCOA3 NEBL NEK1 NEK4 NET1 NFAT5 NFE2L2 NFX1 NKTR NQO1 NR1D2 NR2F2 NUDT15 NUP50 NUP62 OAT OR7E14P OSBPL1A OSBPL3 P4HB PA2G4 PAIP1 PALLD PAM PAWR PBXIP1 PCBP2 PCM1 PDE4DIP PDXDC1 PDZK1IP1 PECI PER2 PERP PEX5 PFDN5 PHACTR2 PHACTR4 PHF3 PHLDA3 PHTF1 PI3 PIBF1 PIGR PIK3R1 PIM2 PIP5K1B PIR PKN2 PKP4 PLAT PLEKHB1 PLS1 PLTP PLUNC PMAIP1 PNMA1 PNPLA4 PPBP PPFIA1 PPIG PPM1H PPP1R12A PPP1R3D PPP3CB PPPDE1 PRKCI PRMT5 PRPF40A PRPF6 PRR4 PRRC1 PRRG4 PSD3 PSENEN PSPH PTK2 PTK6 PTN PTP4A2 PTPN13 PTPRZ1 PWP1 RAB11A RAB2A RABGAP1L RALBP1 RAP2A///RAP2B RAPGEF2 RARRES1 RBL2 RBM25 RBMX2 RBPMS RCBTB1 RCN2 RHOBTB3 RNASE4 RNF103 RNF19A RPLP2 RPS20 RPS21 RPS4Y1 RPS6KA2 RSRC1 RTEL1///TNFRSF6B RUFY3 RWDD1 RYBP SAA1///SAA2 SAA4 SART3 SCAMP1 SEC14L1 SECISBP2L SEL1L3 SEPT11 SERBP1 SERINC3 SERPINB13 SERPINB4 SF3B1 SGPL1 SH3BP4 SH3GLB2 SIGLEC9 SIPA1L1 SIPA1L3 SLC20A1 SLC25A36 SLC25A37 SLC26A4 SLC27A2 SLC34A2 SLC35A3 SLC4A4 SLC6A14 SLC7A1 SLC7A11 SLC7A6 SLC7A8 SLC9A8 SLCO4C1 SMAD1 SMAD5 SMC3 SMC5 SMYD2 SNRPA1 SNX27 SOD2 SON SORD SORL1 SOX4 SPAG1 SPAG16 SPAG9 SPEN SPIN1 SPRY2 SQLE SRD5A1 SREK1 SRI SRPK1 SRPK2 SRPX2 SRRM2 SRSF2IP SSH1 SSPN ST14 ST6GAL1 STAG2 STAT1 STEAP1 STK38 STS SYNCRIP SYNE2 SYNGR1 SYNJ2 TAP1 TASP1 TBL1X TCF25 TCF7L2 TCN1 TES TFAM TFPI TGFA TGOLN2 THUMPD1 TJP2 TM4SF1 TM9SF1 TMBIM6 TMEM131 TMEM30B TMEM45A TMPRSS2 TNC TNFRSF21 TNFSF10 TNIP1 TNPO1 TOB1 TOP1 TOX3 TOX4 TPD52 TPM1 TPR TPSAB1 TPSAB1///TPSB2 TPSB2 TRAK2 TRBC1 TRBC1///TRBC2 TRIB2 TRIM16 TRIM2 TRIM31 TRIM33 TRIM68 TSC2 TSEN34 TSPAN3 TSPAN6 TSPYL1 TSPYL4 TTC3 TTC30A TTF1 TUFT1 TUG1 TWF1 UAP1 UBN1 UBXN4 UCP2 UGCG UGDH UPF3A UPK1B USP1 USP33 VAV3 VEGFA VNN1 VPS37B VWA5A WASL WBP5 WDR45L WDR52 WLS WNK1 WRB WSB1 XBP1 XIST YES1 YIPF6 YY1 ZBTB38 ZBTB43 ZC3H11A ZC3H12A ZC3H15 ZFP36L1 ZMYM2 ZNF124 ZNF148 ZNF238 ZNF273 ZNF323 ZSCAN18 | BAL_18_A1B0 BAL_43_A0B0 BAL_44a_A0B0 BAL_47b_A0B1 BAL_31_A0B1 BAL_46_A2B0 BAL_1_A0B0 BAL_15b_A0B0 CurrentSmoker_54 BAL_41_A1B0 BAL_19_A0B0 BAL_37_A1B1 BAL_33_A0B1 BAL_29_A1B0 BAL_47a_A0B0 BAL_17_A0B0 NeverSmoker_52 BAL_4_A0B0 BAL_20b_A1B1 BAL_27a_A0B0 BAL_3_A1B0 BAL_47c_A0B1 BAL_25_A0B0 Sample_43_never_smoker BAL_12_A1B0 BAL_15a_A0B1 BAL_21_A0B0 BAL_20a_A1B1 BAL_42_A0B0 NeverSmoker_103 FormerSmoker_27 BAL_39_A0B1 BAL_40_A0B0 BAL_13_A0B0 BAL_45a_A1B0 NeverSmoker_45 BAL_44b_A0B0 BAL_26_A2B0 Sample_46_never_smoker CurrentSmoker_37 NeverSmoker_105 FormerSmoker_76 Sample_48_never_smoker NeverSmoker_100 CurrentSmoker_10 |
| **16** | ABCA1 ABCA7 ABCF2 ACAA1 ACTN4 ADAM8 ADRM1 AKAP13 AKT2 ALDH1A1 ALDH3A2 ALDOA ANP32A///ANP32D ANXA11 AP2S1 APPL2 APRT APTX ARAP1 ARHGDIA ARID1A ARL6IP4 ARPC2 ARSD ATF4 ATG2A ATOX1 ATP5D AUP1 B2M B3GALT4 BAZ1B BCAP31 BCL3 BLOC1S1 BNIP3 BOLA2///BOLA2B BRD2 BRD3 C11orf2 C11orf48 C12orf44 C18orf10 C19orf60 C1orf156 C2orf24 C9orf16 C9orf6 CALM1 CALM3 CALR CAPZB CBLB CCL3///CCL3L1///CCL3L3 CCT3 CD151 CD247 CD3D CDC34 CDH1 CDH7 CDKN1A CEP350 CERK CHMP1A CHMP4A CKAP4 CLC CLINT1 CMAH CNGB1 CNPY3 COBRA1 CORO1A CORO1B COX6B1 CRIP1 DCAKD DDX23 DDX27 DDX51 DKFZP586I1420 DNAJC7 DPAGT1 EDF1 EEF1D EHD1 EIF2B5 EIF3B EIF3C///EIF3CL EIF4E2 EIF6 ENTPD1 EZR F13B FAM134A FAM134C FAM50A FAU FBXO42 FBXO46 FBXO5 FLOT2 FOLR1 FOSL2 FPGS G6PD GAK GALNT4///POC1B GALNT6 GATAD2A GBAP1 GLTSCR2 GMPPA GNA15 GOLGA3 GOSR1 GRM8 GSS GUK1 HGS HIPK1 HMHA1 HNRNPUL1 HSP90AB1 HSPA8 HSPD1 HSPH1 HTR5A ICAM3 ID2///ID2B IER3 INADL INPP5K IRAK1 KCNQ4 KDM4B KDM5A KHSRP KIAA0240 KLF6 KPNB1 KRT13 KRT6A LHX1 LLGL1 LMNA LOC150776///SMPD4 MAN2B1 MAP2K2 MAP2K3 MATN4 MBD3 MBTPS1 MCM4 MEAF6 MED16 MFSD5 MICAL1 MLF1 MLLT4 MMP3 MOCS3 MTDH MTUS1 MVP MYL6 MYO18A///TIAF1 NANS NAT15 NDUFA13 NDUFB2 NDUFB7 NDUFS8 NET1 NF2 NFATC2IP NFKBIA NME1-NME2///NME2 NOP56 NOS3 NPRL3 NR2F6 NUCB1 NUPR1 OAT P4HB PAK1 PDE4D PDE6G PDSS2 PDXK PEX5 PFKL PGLS PGRMC1 PHTF1 PIGR PKM2 PMM2 POLR2E POU4F2 PPIB PPIF PPP1R14B PPP2R1B PRDM2 PRKAR1A PRKAR1B PSMB1 PSMB10 PSMB6 PSMD13 PSMD2 PSMD3 PSMD9 PSME2 PTPRA PTPRF PVRL1 PYY2 QARS QSOX1 RABAC1 RABEP2 RAD51C RALGDS RASA3 RBM10 RBM14///RBM4 RBM22 RBM42 RERE RPL13 RPL35 RPLP2 RPN2 RPS10 RPS15 RPS9 RRM1 RRP7A RRP8 RUNX3 S100A13 S100A9 SAE1 SAMM50 SART1 SCAND1 SCGB1D1 SEC14L3 SEC24C SEMA4G SEPT9 SETD5 SF3A2 SF3B5 SLC16A1 SLC16A3 SLC25A44 SLC36A1 SLC39A8 SLC4A7 SLC7A6 SMARCA2 SMARCA4 SMG7 SND1 SNX6 SOX21 SP140L SPOP SPRR1B SPRR3 SPTAN1 STS SUPT6H TALDO1 TAPBPL TAX1BP3 TBL1X TCF25 TCIRG1 TFPT TIMM13 TIMM17A TIMM8B TIMP1 TMED3 TMED9 TMEM208 TMSB10 TNIP2 TNPO1 TOM1 TP53 TPI1 TPPP TSPAN3 TTC15 TYMP UBA7 UBE2J1 UBIAD1 UBQLN3 UBXN1 UCP2 USP10 VARS VGLL1 VPS28 VPS4A WIPF2 WSCD1 YWHAE ZFP36L2 ZNF148 | BAL_15a_A0B1 BAL_3_A1B0 BAL_17_A0B0 BAL_26_A2B0 BAL_45b_A2B0 BAL_4_A0B0 BAL_1_A0B0 BAL_40_A0B0 BAL_44b_A0B0 BAL_47a_A0B0 BAL_27a_A0B0 BAL_13_A0B0 BAL_20b_A1B1 BAL_45a_A1B0 BAL_47c_A0B1 CurrentSmoker_54 BAL_25_A0B0 BAL_41_A1B0 FormerSmoker_104 BAL_29_A1B0 BAL_33_A0B1 BAL_12_A1B0 BAL_18_A1B0 BAL_37_A1B1 BAL_47b_A0B1 CurrentSmoker_111 NeverSmoker_103 BAL_42_A0B0 FormerSmoker_61 BAL_39_A0B1 NeverSmoker_105 CurrentSmoker_102 FormerSmoker_84 NeverSmoker_100 FormerSmoker_107 CurrentSmoker_82 BAL_43_A0B0 CurrentSmoker_109 FormerSmoker_89 FormerSmoker_87 BAL_31_A0B1 CurrentSmoker_106 CurrentSmoker_67 |
| **17** | ABCF3 ABHD2 ACP1 ADD3 ADH7 AHNAK AKAP1 AKAP2///PALM2-AKAP2 AKR1B10 AKR1C1 AKR1C2 AKR1C3 AKR7A2 ALCAM ALDH1A1 ALDH1A2 ALDH1A3 ALDH3A1 ALDH3B2 ALDOA ANKRD11 ANKRD36B ANXA3 AOX1 AP3D1 APOC1 APP ARHGDIA ARID1A ASB7 ASPH ATP13A2 ATP1B1 ATP2B4 ATRX B4GALT4 BAT2L2 BCL2L1 BCLAF1 BEST1 BPTF BRD2 BRD4 BTBD2 C10orf26 C11orf24 C18orf10 C19orf6 C1orf105 C8B CALML3 CALR CAPNS1 CASP5 CAV2 CBLB CBR1 CBR3 CCL3///CCL3L1///CCL3L3 CCL4 CCL5 CCL8 CD151 CD40 CDC14B CDC42BPA CDH1 CDK16 CEACAM5 CEACAM6 CEP110 CEP350 CETP CHD4 CHI3L1 CKAP4 CLCN3 CLDN10 CLINT1 CLPTM1 CNOT3 COMMD3 COMT COPE CPNE3 CROCC CSNK2A1 CSRNP2 CSTA CTBP1 CXCL13 CXCL14 CXCL2 CYC1 CYP1A1 CYP1B1 CYP3A5 DCAF11 DDIT3 DDX17 DGKA DHX9 DKFZP586I1420 DLX4 DUOX2 DUSP1 DYRK2 EDNRB EGFL6 EGR1 EHF EID1 EIF2AK3 EIF4G1 EIF6 EMP1 EMR1 EPB41 EPHX1 EPRS EREG ERGIC2 EXOC7 EZR F11R FABP4 FAF2 FAIM3 FAM108A1 FAM111A FBXO21 FGFBP1 FKTN FOLR1 FOS FUT3 FUT4 FUT6 G0S2 GADD45B GALNT1 GALNT6 GALNT7 GAPDH GATAD1 GCLC GCLM GDF15 GFPT1 GLG1 GLTP GMDS GMNN GNAL GNE GNG11 GOLGA2 GOLGA8H GOLIM4 GOSR2 GOT2 GPR109B GPS1 GPX2 GSN GSR GSTA1 GSTM3 HEBP2 HIST1H4C HLA-DRB4///LOC100509582 HMG20B HMGCS1 HNRNPL HNRNPUL1 HOPX HP HP///HPR HS3ST1 HSD11B1 HSP90B1 HSPA1A///HSPA1B HTR4 IDO1 IFI16 IL1A IL1B IL8 ILF3 IMPA2 INTS7 IQGAP1 IQSEC3 JUNB KCNAB1 KDELR1 KDM5B KIAA0090 KIAA1009 KLC1 KLF5 KLRC1///KLRC2 KPNA3 KPTN KRAS KRT13 KRT14 KRT4 KRT6A KRT6A///KRT6B///KRT6C KRT6B KTN1 LCK LEPROT LILRA6///LILRB3 LIMCH1 LIMS1 LOC100288142///NBPF1///NBPF10 LOC100293553 LOC100506168///SFPQ LOC100507804///TPSAB1 LOC150759 LOC642869///SET LPCAT4 LUC7L3 LY6D MACF1 MAP9 MARCKS MARK2 MAZ MBD5 MDM4 ME1 MED27 MEX3C MGAT4B MIF MLEC MMP14 MRPS12 MRPS18A MSMB MTMR2 MTUS1 MUC1 MUC5AC MUTED///TXNDC5 MYO6 NAA15 NAA35 NBPF10 NCOA3 NDUFS8 NEK1 NET1 NFAT5 NFKBIA NFX1 NHLH2 NKG7 NKTR NKX3-1 NMU NOMO1///NOMO2///NOMO3 NOP16 NPEPPS NQO1 NR0B1 NR4A2 NR4A3 NXF3 OGDH OTUB1 P4HB PA2G4 PALLD PCBP2 PCDHB12 PCYOX1 PDP1 PEA15 PER1 PERP PFKFB3 PGLS PGS1 PIK3IP1 PIR PLA2G3 PLCB4 PLS3 PMAIP1 PML PMS1 PMS2P3 PON2 PPDPF PPIB PPIF PPP2R5D PPP4C PPP5C PPPDE1 PRDX1 PRF1 PRKCI PRKCSH PROS1 PRPF6 PRR4 PRRC1 PTBP1 PTGS2 PURA RAB14 RAB2A RALBP1 RALY RANBP3 RASGRP1 RBM25 RGS1 RGS2 RHOBTB3 RHOC RNASE1 RNF115 RNMTL1 RRAS2 RRBP1 RYBP S100A14 SBF1 SCAMP1 SCD5 SCEL SCT SDF4 SEC14L1 SEC61G SEC63 SECISBP2L SERBP1 SERPINA1 SERPINB13 SERPINB5 SEZ6L SFN SFRS18 SFTPA2 SFTPB SH3GL3 SH3GLB2 SIRPB1 SLC22A13 SLC25A15 SLC25A36 SLC26A2 SLC2A3 SLC35A2 SLC35A3 SLC35E1 SLC7A11 SMARCB1 SMC3 SMC5 SNRPB SOD2 SON SOS2 SPEN SPRR1A SPRR1B SPRR3 SRRM2 SRSF2IP ST8SIA4 STAB1 STATH SYNCRIP SYNJ2 TBL1X TCF25 TCF4 TCN1 TES TGOLN2 TLR3 TM4SF1 TMEM183A///TMEM183B TMEM45A TMEM50B TNFAIP1 TNFAIP6 TNFRSF21 TNFSF10 TNPO1 TOP1 TOP2A TOX4 TPD52 TPR TPSAB1 TPSAB1///TPSB2 TPSB2 TRBC1 TRIM16 TTC3 TXN TXN2 TXNRD1 UBE2M UBE2S UBXN4 UCHL1 UCP2 UGCG UGT1A1///UGT1A10///UGT1A3///UGT1A4///UGT1A5///UGT1A6///UGT1A7///UGT1A8///UGT1A9 UGT1A1///UGT1A10///UGT1A4///UGT1A6///UGT1A8///UGT1A9 UPK1B USO1 USP1 VAC14 VCP VNN2 VPS37B VRK1 WASL WIF1 WIPI2 YES1 YWHAB YWHAE ZBTB17 ZBTB38 ZC3H11A ZC3H15 ZFP36L1 ZFP36L2 ZSCAN18 | BAL_39_A0B1 BAL_20a_A1B1 BAL_44a_A0B0 BAL_19_A0B0 BAL_46_A2B0 BAL_27b_A2B1 BAL_29_A1B0 BAL_47c_A0B1 BAL_47a_A0B0 BAL_40_A0B0 BAL_47b_A0B1 BAL_18_A1B0 BAL_20b_A1B1 BAL_21_A0B0 BAL_43_A0B0 BAL_25_A0B0 BAL_41_A1B0 BAL_42_A0B0 BAL_15a_A0B1 NeverSmoker_93 BAL_33_A0B1 BAL_13_A0B0 BAL_44b_A0B0 BAL_37_A1B1 NeverSmoker_103 BAL_15b_A0B0 BAL_31_A0B1 BAL_45a_A1B0 NeverSmoker_105 BAL_27a_A0B0 BAL_26_A2B0 BAL_17_A0B0 BAL_4_A0B0 BAL_12_A1B0 BAL_1_A0B0 BAL_3_A1B0 CurrentSmoker_98 FormerSmoker_84 NeverSmoker_5 NeverSmoker_78 CurrentSmoker_29 NeverSmoker_100 CurrentSmoker_115 NeverSmoker_94 NeverSmoker_52 FormerSmoker_104 |
| **18** | ABCA8 ABCB6 ABCC1 ABCD1 ACAP1 ACTR5 ADH7 AKR1B1 AKR1B10 AKR1C1 AKR1C2 AKR1C3 ALDH3A1 ALOXE3 ANKRD1 ANXA6 APOL1 APOL3 ATF3 ATP11A ATP6V1D AZGP1 B2M BLZF1 BRIP1 BTN3A2 BTN3A2///BTN3A3 C14orf139 C15orf34 C19orf40 C3 C4A///C4B///LOC100509001 C7orf64 C9orf114 CABYR CALCRL CALM1 CALML3 CBR1 CBR3 CCL5 CCL8 CCND2 CD2 CD247 CD38 CD3D CD40 CD48 CD59 CD74 CDH5 CDH6 CDKN2C CEACAM5 CES1 CFB CH25H CIITA CLDN10 CLMN CNGB1 COPG2IT1 CORO1A CPS1 CSF1 CSTA CXCL10 CXCL11 CXCL13 CXCL9 CYP1A1 CYP1B1 CYP4F3 CYR61 DEFB1 DHX35 DIAPH2 DNMT1 DPP6 DSG3 DYRK2 EGFL6 EGR1 EGR3 ENOX2 ENPP2 FAM120A FAM13A FAM21A///FAM21B///FAM21C FASTKD5 FEM1B FGFBP1 FLT3LG FOLR1 FOSB GABARAPL1 GABBR1///UBD GAGE1///GAGE12F///GAGE12G///GAGE12I///GAGE12J///GAGE2A///GAGE2B///GAGE2C///GAGE2D///GAGE2E///GAGE3///GAGE4///GAGE5///GAGE6///GAGE7///GAGE8 GAGE1///GAGE12F///GAGE12G///GAGE12I///GAGE12J///GAGE4///GAGE5///GAGE6///GAGE7 GAGE12C///GAGE12D///GAGE12E///GAGE12F///GAGE12G///GAGE12H///GAGE12I///GAGE2A///GAGE2C///GAGE4///GAGE5///GAGE6///GAGE7 GBP1 GCLC GCLM GDF15 GFPT2 GFRA1 GHRH GNLY GPC4 GPLD1 GPR171 GPX2 GRID2 GRSF1 GZMA GZMB GZMH GZMM H3F3A///LOC440926 HBEGF HGD HLA-A HLA-B HLA-C HLA-DPA1 HLA-E HLA-F HLA-G HSP90AA1 IDO1 IER3 IFI27 IFITM1 IFITM3 IFNA7 IFNG IGF1 IGK@///IGKC IGKC IGL@ IGLC7///IGLV1-44///LOC100290481 IGLL3P IGLV1-44///LOC100290481 IL32 IL7 IL8 IQSEC3 IRF1 ITGA7 ITGB6 KCNMA1 KCTD12 KIAA1467 KIF2A KLRD1 KRT13 KRT14 KRT6A LAG3 LAP3 LCK LCN2 LOC100507804///TPSAB1 LPIN1 LPPR4 LSS LTB LTF MAP2K6 MBL2 MICAL2 MLL MLLT4 MPHOSPH9 MPPE1 MPPED2 MRC1///MRC1L1 MUC2 MUC5AC MUC5B MYCL1 MYST3 NAA35 NCRNA00081 NDC80 NDEL1 NHLH2 NKG7 NKX3-1 NQO1 NR0B1 NR4A1 NR4A3 NTN3 OSGIN1 OTUD7B PBRM1 PDE7B PDK3 PDZK1IP1 PECI PHF16 PIR PIWIL2 PLCXD1 PMAIP1 PNMA2 PP14571 PPP1R16B PPP1R7 PRDX1 PRF1 PROS1 PRR4 PSMB10 PSMB8 PSMB9 PSMD1 PSME2 PTK6 PVRIG RARRES1 RARRES3 RAVER2 RCAN1 REG1P RNF8 ROD1 RTEL1 RTEL1///TNFRSF6B RXRB SAA1///SAA2 SAA4 SCAND2 SCARB1 SCGB1A1 SERPINB13 SERPINB5 SFTPA2 SFTPB SFTPC SH2B2 SIGLEC8 SIRPG SLAMF1 SLC19A1 SLC25A42 SLC26A4 SLC27A6 SLC29A1 SLC30A1 SLC34A2 SLC3A2 SLC4A7 SLC5A3 SLC7A11 SMPX SORL1 SOX30 SPARCL1 SPP1 SPRR1A SPRR1B SPRR3 SRPX2 SRRM1 STAT1 STC2 STX2 SUV420H1 SYNGR1 TALDO1 TAP1 TAP2 TAS2R13 TCF3 TCN1 TESK1 TEX12 TFF1 TGM2 TIMP3 TKT TM4SF1 TMEM176A TMEM45A TNFAIP6 TNFRSF11A TNFSF10 TNXA///TNXB TOP2B TOX TPSAB1 TPSAB1///TPSB2 TPSB2 TRAC TRAC///TRAJ17///TRAV20 TRAF3IP3 TRBC1 TRBC1///TRBC2 TRD@ TRIM16 TRIM26 TRIP10 TRPC6 TXN TXNRD1 UBAP2L UBIAD1 UCHL1 UGT1A1///UGT1A10///UGT1A3///UGT1A4///UGT1A5///UGT1A6///UGT1A7///UGT1A8///UGT1A9 UGT1A1///UGT1A10///UGT1A4///UGT1A6///UGT1A8///UGT1A9 UPK1B USP18 VPS13D VPS37B VRK1 WARS WDHD1 XCL1 XCL1///XCL2 ZFP30 ZNF12 ZNF323 | CurrentSmoker_54 CurrentSmoker_1 BAL_46_A2B0 BAL_20a_A1B1 BAL_19_A0B0 CurrentSmoker_29 BAL_21_A0B0 BAL_44a_A0B0 CurrentSmoker_2 NeverSmoker_52 BAL_47c_A0B1 BAL_29_A1B0 BAL_41_A1B0 BAL_31_A0B1 CurrentSmoker_36 BAL_47a_A0B0 BAL_18_A1B0 CurrentSmoker_37 BAL_27b_A2B1 NeverSmoker_3 BAL_15b_A0B0 BAL_12_A1B0 BAL_1_A0B0 BAL_43_A0B0 BAL_47b_A0B1 BAL_15a_A0B1 BAL_44b_A0B0 BAL_42_A0B0 BAL_25_A0B0 NeverSmoker_103 BAL_40_A0B0 BAL_45a_A1B0 NeverSmoker_38 BAL_27a_A0B0 NeverSmoker_93 BAL_33_A0B1 CurrentSmoker_12 BAL_17_A0B0 FormerSmoker_69 BAL_20b_A1B1 FormerSmoker_41 BAL_3_A1B0 BAL_4_A0B0 CurrentSmoker_115 BAL_13_A0B0 CurrentSmoker_10 BAL_26_A2B0 BAL_39_A0B1 FormerSmoker_84 BAL_37_A1B1 NeverSmoker_105 FormerSmoker_104 CurrentSmoker_98 CurrentSmoker_39 FormerSmoker_76 FormerSmoker_87 Sample_40_former_smoker_without_cancer Sample_48_never_smoker Sample_43_never_smoker NeverSmoker_100 FormerSmoker_61 |
| **19** | AAK1 ACAP1 AKAP1 ANKRD12 ARG2 ARL4C ASXL1 ATP5E ATP5I ATP5O ATP6V0E1 ATP6V1D ATXN1 ATXN10 AZIN1 B4GALT1 BANF1 BAT2L2 BNIP3 BRCA1 BTBD1 BTD C14orf104 C14orf156 C14orf2 C16orf42 C16orf80 C1orf25 C4orf27 C4orf46///TOMM7 CALM1 CALM2 CAMLG CD300A CD59 CD6 CDC5L CDK11A///CDK11B CEACAM1 CEACAM21 CEP57 CES1P1 CETN3 CFDP1 CH25H CIRBP CLCC1 CLDN8 COPS8 COQ6 COX5B COX6C COX7C CPSF1 CRISP2 CRLF2 CTBP1 CTNNB1 CXCR6 CYP2D6 DALRD3 DDX54 DHX40 DKFZp686O1327 DNAJC4 DUSP22 DUT DUX1///DUX3///DUX5 DYNLL1 EDC3 EED EEF1D EGR3 EIF1 EIF2C4 EIF5A ERAP1 ESD FAM13A FAM65B FBXO40 FBXW2 FDFT1 FGF8 FNBP4 FOLR1 FTO FZR1 GAGE1 GALC GALNT10 GCLC GCSH///LOC100329108 GDF10 GNAS GNLY GOLGA8A GP1BB///SEPT5 GPR172A GTF2H4 GTF3C5 GYPC GZMK H3F3A///LOC440926 HBA1///HBA2 HEATR1 HGD HINT1 HIST1H2BJ HMGCR HMGN1 HMGN3 HNRNPA0 HNRNPL HSP90AA1 HSP90B1 HSPA1A///HSPA1B HSPB11 HSPH1 IFITM3 IGFBP7 IK///TMCO6 IL7 IPO9 ITK KBTBD11 KCNJ2 KCTD12 KIAA0182 KIAA0232 KTN1 LAD1 LCK LMO2 LOC100507315///PPP2R5C LOC100507328 LOC100509558///LOC100510047 LOC150776///SMPD4 LPCAT3 LSM4 LSM5 LY75 LZTFL1 MAP4K1 MARCKS MARK2 MCL1 ME3 MLH3 MORF4L2 MRP63 MRPL52 MRPS31 MTCP1NB NANS NAP1L1 NAT1 NCRNA00081 NDUFB1 NDUFB3 NFKB2 NFU1 NIPA2 NIPBL NKIRAS2 NME7 NPC1L1 NR1D2 NRXN2 NUCKS1 NXF1 OLA1 OLIG2 ORM1///ORM2 P4HB PAIP1 PCDHGA10///PCDHGA11///PCDHGA12///PCDHGA3///PCDHGA5///PCDHGA6 PDIA4 PEBP1 PGRMC1 PMAIP1 POLR1D PPP3CA PRDX6 PRKAR1A PROS1 PRPS1 PSPH PTPN21 PVR RBBP4 RBM5 RDH14 REEP5 RFTN1 RLF RND3 RPL14 RPL23 RPL27A RPL34 RPL35A RPL37A RPL38 RPLP2 RPS11 RPS3A RPS7 RUFY3 RYBP S100A8 S100A9 SC4MOL SCARB1 SDHC SEC14L1 SECISBP2L SERP1 SET SFXN3 SIRT7 SKP1 SLC19A1 SLC35A3 SLC39A6 SLC39A8 SMARCA2 SMG1 SNRPE SOBP SOD2 SPRR3 SQLE SRI STAG2 STATH STAU1 STOM STUB1 SUMO2 TAS2R13 TAX1BP1 TBC1D1 TBCA TCFL5 TCP1 TFAM TK2 TMEM63A TNFRSF10C TNPO1 TPR TRAC TRAF3IP2 TRAF5 TRIM14 TRIM31 TROVE2 TSC2 TSPYL4 TTBK2 TUBBP5 TUBGCP2 TXN TYMP UBIAD1 UBL5 UGDH VAPA VPS13B WRB YIPF6 YPEL5 YWHAE ZBED2 ZNF202 ZNF225 ZNF816 | NeverSmoker_52 BAL_20a_A1B1 NeverSmoker_3 CurrentSmoker_54 BAL_44a_A0B0 CurrentSmoker_37 CurrentSmoker_12 FormerSmoker_18 BAL_15b_A0B0 FormerSmoker_69 CurrentSmoker_2 BAL_19_A0B0 BAL_46_A2B0 CurrentSmoker_36 CurrentSmoker_29 BAL_45b_A2B0 NeverSmoker_45 BAL_31_A0B1 Sample_44_never_smoker BAL_41_A1B0 FormerSmoker_27 BAL_47c_A0B1 BAL_47a_A0B0 Sample_40_former_smoker_without_cancer Sample_43_never_smoker BAL_29_A1B0 Sample_48_never_smoker CurrentSmoker_1 FormerSmoker_28 NeverSmoker_42 BAL_21_A0B0 BAL_44b_A0B0 BAL_12_A1B0 BAL_25_A0B0 BAL_37_A1B1 BAL_13_A0B0 BAL_20b_A1B1 NeverSmoker_5 NeverSmoker_93 BAL_27b_A2B1 BAL_33_A0B1 BAL_26_A2B0 FormerSmoker_114 Sample_47_never_smoker BAL_27a_A0B0 BAL_1_A0B0 BAL_4_A0B0 |
| **20** | ABCA1 ABCB1///ABCB4 ACACB ACAP1 ACP6 ADAM19 ADAM8 ADAMDEC1 ADM ADORA2A///SPECC1L AKR1C3 ALPK3 ANXA6 AOAH AP1S2 AP2A2 APOBEC3A APOBEC3F///APOBEC3G APOBEC3G APOL3 AQP1 AQP4 ARHGDIA ARHGEF1 ARHGEF40 ARL4A ARL4C ATP1B1 ATP2B4 ATP8A1 ATP8B4 BATF BCAT1 BCL11B BCL2 BCL3 BIRC3 BTG2 BTN3A3 BUB1 C14orf139 CAV1 CCL13 CCL18 CCL2 CCL3///CCL3L1///CCL3L3 CCL4 CCL5 CCL7 CCL8 CCND2 CCR2 CCR5 CCR7 CD2 CD247 CD27 CD28 CD300A CD36 CD3D CD3E CD48 CD6 CD7 CD84 CD8A CD8B CD9 CD93 CD96 CFLAR CFP CHI3L1 CHIT1 CHST15 CLC CLDN18 CLEC2D CORO1A CPE CREB5 CSF3R CSGALNACT1 CST7 CSTA CTLA4 CX3CR1 CXCL10 CXCL11 CXCL13 CXCL9 CXCR2 CXCR4 CXCR6 CXCR7 CYFIP2 CYTH4 DEFB1 DENND3 DGKA DHRS1 DOCK4 DPP4 DUSP2 DUSP5 DUSP6 DYSF EGR3 EHD1 EIF5A EMP1 EMR2 ENPP2 ENTPD1 EXD2 F13A1 FAIM3 FAM162A FAM65B FAS FCAR FCGR2B FCGR3B FCN1 FDFT1 FFAR2 FGL2 FLT3LG FPR1 FPR3 FUS FYB FYN G0S2 GABBR1///UBD GADD45B GALNT12 GBP1 GCH1 GIMAP5 GIMAP6 GJA1 GM2A GNG12 GNLY GPR171 GPR18 GPR183 GRAMD4 GRK5 GZMA GZMB GZMH GZMK HCP5 HIP1 HLA-C HLA-E HMHA1 HOMER3 HOPX HPSE HS3ST2 HSPA6 ICAM1 ICAM3 ID2///ID2B IDO1 IER3 IFITM1 IFITM2 IFITM3 IFNG IGHM IGLV1-44///LOC100290481 IL18R1 IL18RAP IL1B IL1R1 IL1R2 IL21R IL2RB IL32 IL6R IL7R IL8 INPP5D IRF1 ISG20 ITGA4 ITK ITM2A KAZ KCNAB1 KCNJ15 KCNJ2 KDM6B KIAA0040 KLRB1 KLRC1///KLRC2 KLRD1 KLRK1 LAG3 LAMP3 LAT///SPNS1 LBH LCK LCP2 LEF1 LGALS2 LGMN LILRA3 LILRB1 LILRB2 LILRB4 LMNB1 LOC100507804///TPSAB1 LPCAT1 LTB MAF MAL MARCKS MAU2 MEF2C MERTK MGAT3 MLPH MMP1 MMP12 MREG MST4 MTX2 MXD1 MYBL1 MYLIP MYO6 MYO7A NEDD9 NFKB2 NINJ1 NKG7 NLRP3 NR1H3 NRGN NUMA1 NUP62 OLFML2B ORM1 ORM1///ORM2 OSM P2RY13 P2RY14 PAK1 PAPSS2 PBXIP1 PDE4B PEG10 PEX11A PI3 PIK3CD PIK3R5 PIM1 PIM2 PLA2G7 PLEKHO1 PLK3 PLTP PLXNC1 PMAIP1 PPIC PPIF PPP1R16B PRF1 PRKCB PRKCQ PSMB9 PSTPIP1 PSTPIP2 PTGER2 PTPN7 PTPRCAP PVRIG PXN QPCT RAB27A RALGDS RASGRP1 RASSF2 RELB RFTN1 RHBDF2 RHOF RHOH RNASE1 RNASE2 RSAD2 RUNX3 S100A12 S100A13 S1PR1 SBNO2 SC4MOL SDC2 SDS SEC14L1 SELL SEMA4D SEPP1 SERPINB9 SFTPA2 SFTPB SFTPC SFTPD SH2D1A SH2D2A SH3BP4 SIRPG SKAP1 SLAMF1 SLC25A37 SLC38A1 SLC39A8 SLC7A5 SMCHD1 SMPDL3A SNAP23 SOBP SOD2 SPOCK2 SPP1 SRSF6 ST8SIA4 STAB1 STAT1 STAT4 STAT5B STEAP4 STK17A SYNE1 TAP1 TBX21 TIMP1 TLE3 TLR2 TMEM140 TMEM149 TMEM176B TMEM2 TNF TNFAIP3 TNFAIP6 TNFRSF10C TNFRSF1B TNFSF14 TNFSF8 TNIP1 TOX TPD52L1 TPSAB1 TPSAB1///TPSB2 TPSB2 TRAC TRAC///TRAJ17///TRAV20 TRAF1 TRAF3IP3 TRAF5 TRAT1 TRBC1 TRBC1///TRBC2 TRD@ TRDV3 TSPAN3 TXK TYMP UBASH3A VAMP5 VCAN VEGFA XCL1 XCL1///XCL2 ZAP70 ZBED2 ZEB1 ZFP36L2 | BAL_20a_A1B1 BAL_45b_A2B0 CurrentSmoker_54 BAL_42_A0B0 BAL_20b_A1B1 BAL_15b_A0B0 CurrentSmoker_10 NeverSmoker_38 NeverSmoker_52 NeverSmoker_103 CurrentSmoker_12 FormerSmoker_69 NeverSmoker_93 Sample_43_never_smoker CurrentSmoker_1 FormerSmoker_18 BAL_31_A0B1 BAL_41_A1B0 NeverSmoker_78 NeverSmoker_105 FormerSmoker_76 NeverSmoker_100 BAL_37_A1B1 BAL_4_A0B0 CurrentSmoker_29 FormerSmoker_27 NeverSmoker_5 CurrentSmoker_98 CurrentSmoker_115 BAL_29_A1B0 Sample_44_never_smoker BAL_44a_A0B0 CurrentSmoker_112 NeverSmoker_3 FormerSmoker_104 NeverSmoker_45 Sample_40_former_smoker_without_cancer FormerSmoker_110 BAL_19_A0B0 BAL_45a_A1B0 BAL_12_A1B0 BAL_47c_A0B1 FormerSmoker_28 NeverSmoker_42 CurrentSmoker_73 BAL_13_A0B0 BAL_26_A2B0 BAL_47a_A0B0 Sample_48_never_smoker FormerSmoker_114 |

*BAL- Human Lung transplant. The numeric part of the sample name is an arbitrary identifier for individual patients. Bronchoalveolar lavage samples obtained from lung transplant recipients whose biopsies had a perivascular score (A) of between 0 and 2, and a bronchiolar score (B) of between 0 and 1. A combined A and B score of 2 or more represents an acute rejection [46].

**Lung epithelial cell transcriptome study of 34 current smokers, 18 former smokers, and 23 subjects who had never smoked [47].
