# Supplementary material for: Suppressed Expression of T-Box Transcription Factors Is Involved in Senescence in Chronic Obstructive Pulmonary Disease
Source: PLoS Comput Biol. 2012 Jul 19;8(7):e1002597. doi: 10.1371/journal.pcbi.1002597 (PMC3400575; doi:10.1371/journal.pcbi.1002597)
Supplement: Table S5 — Details of CLR Execution that Generated Results Found in Figure S1 and Table S1. (DOC) [file pcbi.1002597.s009.doc]

**Table S5. Details of CLR Execution that Generated Results Found in Figure S1 and Table S1.**

|  |  | **U133A (109 Arrays)** | |  | **U133Plus_2 (49 Arrays)** | |
| --- | --- | --- | --- | --- | --- | --- |
|  | **Number of Probe sets** | **Median Number of Bins (SD)** | **Likelihood Estimate Threshold Corresponding to 0.05 FDR** | **Number of Probe sets** | **Median Number of Bins (SD)** | **Likelihood Estimate Threshold Corresponding to 0.05 FDR** |
| Apoptosis Probe sets | 902 | 8 (2.65) | 3.95 | 1219 | 5 (1.82) | 3.47 |
| Inflammatory Response Probe sets | 248 | 8 (4.1) | 2.73 | 326 | 6 (2.53) | 3.11 |
| Response to Oxidative Stress Probe sets | 115 | 7 (3.2) | 3.36 | 153 | 5 (1.86) | 2.71 |

SD= Standard deviation; FDR= False discovery rate
